# 1,2-Disubstituted bicyclo[2.1.1]hexanes as saturated bioisosteres of *ortho*-substituted benzene†

**DOI:** 10.1039/d3sc05121h

**Published:** 2023-10-27

**Authors:** Aleksandr Denisenko, Pavel Garbuz, Yelyzaveta Makovetska, Oleh Shablykin, Dmytro Lesyk, Galeb Al-Maali, Rodion Korzh, Iryna V. Sadkova, Pavel K. Mykhailiuk

**Affiliations:** a Enamine Ltd Winston Churchill st. 78 02094 Kyiv Ukraine www.mykhailiukchem.org Pavel.Mykhailiuk@gmail.com; b V. P. Kukhar Institute of Bioorganic Chemistry and Petrochemistry of the NAS of Ukraine 02094 Kyiv Ukraine; c Bienta Winston Churchill st. 78 02094 Kyiv Ukraine; d Institute of Botany of the National Academy of Sciences of Ukraine 02094 Kyiv Ukraine

## Abstract

Bicyclo[2.1.1]hexanes have been synthesized, characterized, and biologically validated as saturated bioisosteres of the *ortho*-substituted benzene ring. The incorporation of the 1,2-disubstituted bicyclo[2.1.1]hexane core into the structure of fungicides boscalid (BASF), bixafen (Bayer CS), and fluxapyroxad (BASF) gave saturated patent-free analogs with high antifungal activity.

## Abstract 2

1,2-Disubstituted bicyclo[2.1.1]hexanes have been synthesized, characterized, and biologically validated as saturated bioisosteres of the *ortho*-substituted benzene ring.

## Graphical abstract

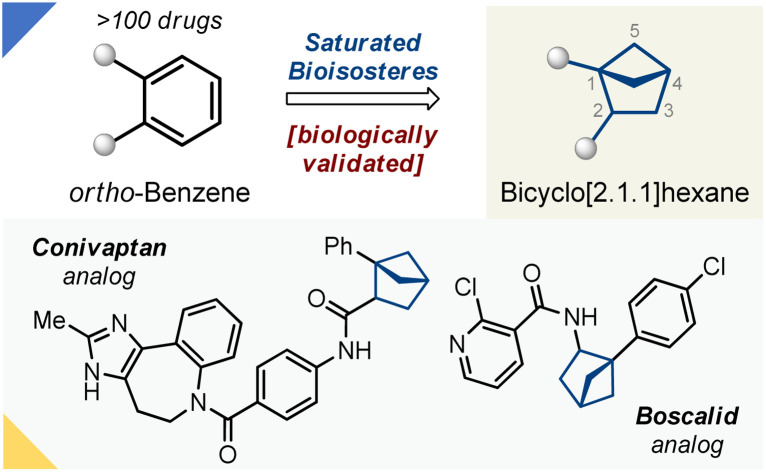

## Introduction

The benzene ring is a basic structural unit in chemistry, and we learn about it in school. It is the most popular ring in natural products^1^ and bioactive compounds.^2,3^

**Fig. 1:**
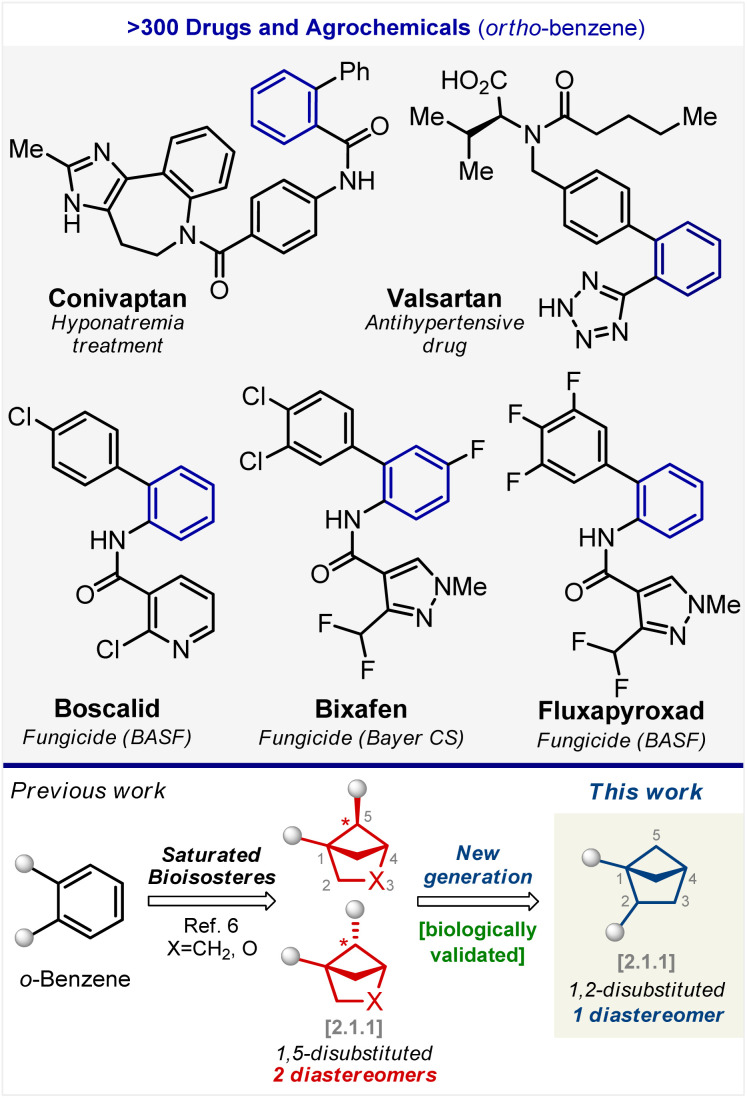
Bicyclo[2.1.1]hexanes as saturated bioisosteres of the *ortho*-substituted benzene ring: state of the art.

The *ortho*-substituted benzene ring, in particular, is found in the structure of more than three hundred drugs and agrochemicals (Fig. 1).^4,5^ For example, the well-known drug aspirin contains an *ortho*-substituted benzene ring. Recently, we discovered that 1,5-disubstituted bicyclo[2.1.1]hexanes and their oxa-containing analogs can mimic the *ortho*-substituted benzene ring in bioactive compounds (Fig. 1).^6^ These scaffolds were synthesized as a mixture of two diastereomers. Some of them were subsequently separated by crystallization or column chromatography. In many cases, however, the separation of diastereomers failed, and the desired products were not obtained.

Pleasingly, these studies were well received by the scientific community, and the groups of Glorius,^7^ Brown,^8^ Procter,^9^ Li,^10^ Wang,^11^ and Studer^12^ subsequently developed alternative approaches to di- and poly-substituted bicyclo[2.1.1]hexanes based on the functionalization of bicyclo[1.1.0]butanes.^13–15^

In this work, we have synthesized, characterized, and biologically validated 1,2-disubstituted bicyclo[2.1.1]hexanes as a new generation of saturated bioisosteres of *ortho*-substituted benzenes. These structural cores exist as single diastereomers. Bicyclo[2.1.1]hexanes were incorporated into the structure of fungicides boscalid (BASF), bixafen (Bayer CS), and fluxapyroxad (BASF) to provide saturated patent-free analogs with high antifungal activity.

## Results and discussion

### Design

**Fig. 2:**
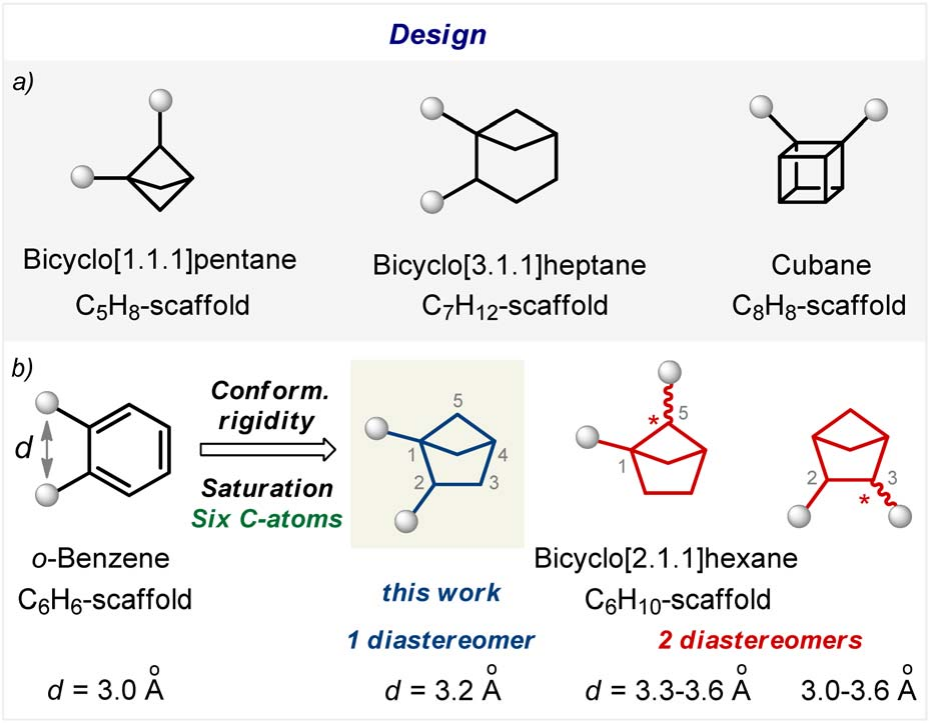
Design of saturated bioisosteres of the *ortho*-substituted benzenes. (a) Disubstituted bicyclo[1.1.1]pentanes, bicyclo[3.1.1]heptanes, and cubanes. (b) Disubstituted bicyclo[2.1.1]hexanes.

Benzene (C_6_H_6_; M.W. = 78) is an aromatic molecule. Among the conformationally rigid bi(poly)cyclic saturated scaffolds, – bicyclo[1.1.1]pentane,^16^ bicyclo[3.1.1]heptane,^17^ cubane,^18^ and bicyclo[2.1.1]hexane (Fig. 2), – only the latter has the same number of carbon atoms and a similar molecular weight (C_6_H_10_; M.W. = 82; Fig. 2b).^19,20^ Three types of substitution patterns in bicyclo[2.1.1]pentane can potentially mimic the *ortho*-benzene ring geometry: 1,2-, 1,5-, and 2.3- (Fig. 2b). While two latter cores exist as two diastereomers, the 1,2-disubstituted bicyclo[2.1.1]pentanes exist as only one diastereomer (Fig. 2). Moreover, this scaffold was recently proposed to mimic the *ortho*-substituted benzene ring; however, biological validation of this hypothesis was not made.^9^ In this work, we report a practical approach to 1,2-disubstituted bicyclo[2.1.1]hexanes and show with biological experiments that this core indeed can act as a bioisostere of the *ortho*-substituted benzene ring in bioactive compounds.

### Synthesis

**Scheme 1:**
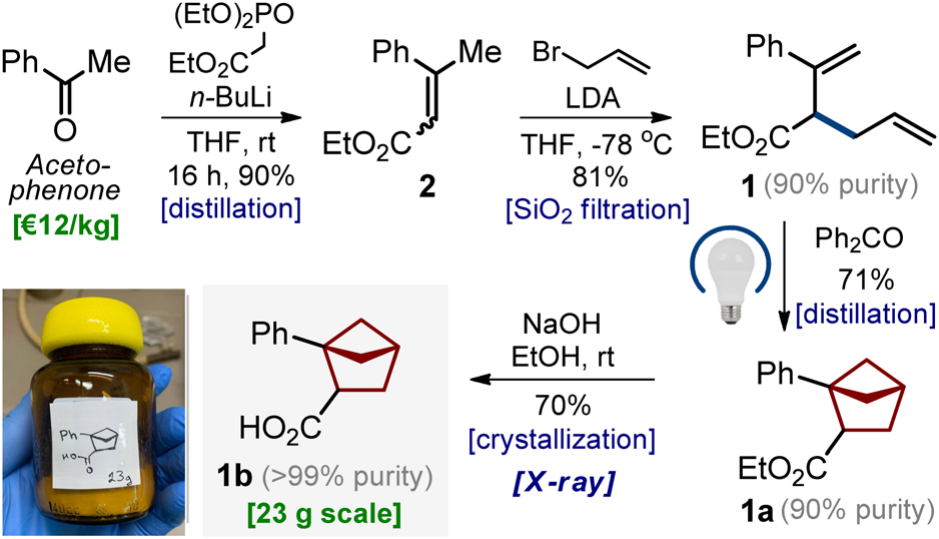
Gram-scale synthesis of bicyclo[2.1.1]hexane 1b from acetophenone.

Despite numerous studies on the topic,^7–15^ we needed a practical approach to bicyclo[2.1.1]hexanes with only two substituents (two exit vectors) at the 1- and 2-positions of the core (Scheme 1) without additional (poly)substitution at other positions. Moreover, one substituent should be (hetero)aromatic, and another one should be the carboxylic group, which is needed for the subsequent modifications of the core *via* amide coupling.^21,22^

In light of our previous experience,^6^ we wondered if diene 1 (easily obtained from acetophenone, please see Scheme 1) would undergo an intramolecular photocycloaddition into the desired bicyclo[2.1.1]hexane core. Direct irradiation of diene 1 in acetonitrile at different wavelengths gave only traces of products (entries 1–4, Table 1). Irradiation with a broad wavelength mercury lamp gave the target product in 35% yield along with the formation of unidentified side products (entry 5). We also tried available organic ketones for the triplet sensitization of the styrene moiety. Cleaner formation of the desired bicyclo[2.1.1]hexane 1a was observed (entries 6–10). The best yield of 76% was obtained with benzophenone (entry 8), whereas thioxanthone also worked well (entry 7). Among all tested solvents (entries 11–14), the best result was obtained in acetonitrile. Without irradiation, the reaction did not take place (entry 15).

**Table 1:** Optimization of the synthesis of bicyclo[2.1.1]hexane 1a

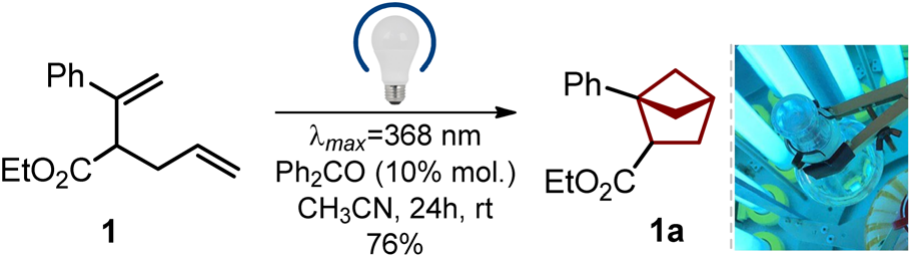		
Entry	Conditions	Yield^a^^,^^b^ (%)
1	450 nm, CH_3_CN	n.r.
2	368 nm, CH_3_CN	<10
3	313 nm, CH_3_CN	<10
4	254 nm, CH_3_CN	<20
5	Broad wavelength Hg lamp, CH_3_CN	35
6	368 nm, CH_3_CN, acetophenone	43
7	368 nm, CH_3_CN, thioxanthone	54
8	368 nm, CH_3_CN, benzophenone	(82)76^c^
9	368 nm, CH_3_CN, (*p*-NO_2_C_6_H_4_)_2_CO	31
10	368 nm, CH_3_CN, (*p*-Me0C_6_H_4_)_2_CO	62
11	368 nm, CH_2_Cl_2_ Ph_2_CO	47
12	368 nm, Me_2_CO, Ph_2_CO	45
13	368 nm, PhMe, Ph_2_CO	31
14	368 nm, EtOAc, Ph_2_CO	43
15	In the dark, rt	n.r.

a 100 mmol scale.

b ^1^H NMR yield (CH_2_Br_2_ as an internal standard).

c Isolated yield after column chromatography. See the ESI for details. n.r.: no reaction.

### Scalable synthesis

**Scheme 2:**
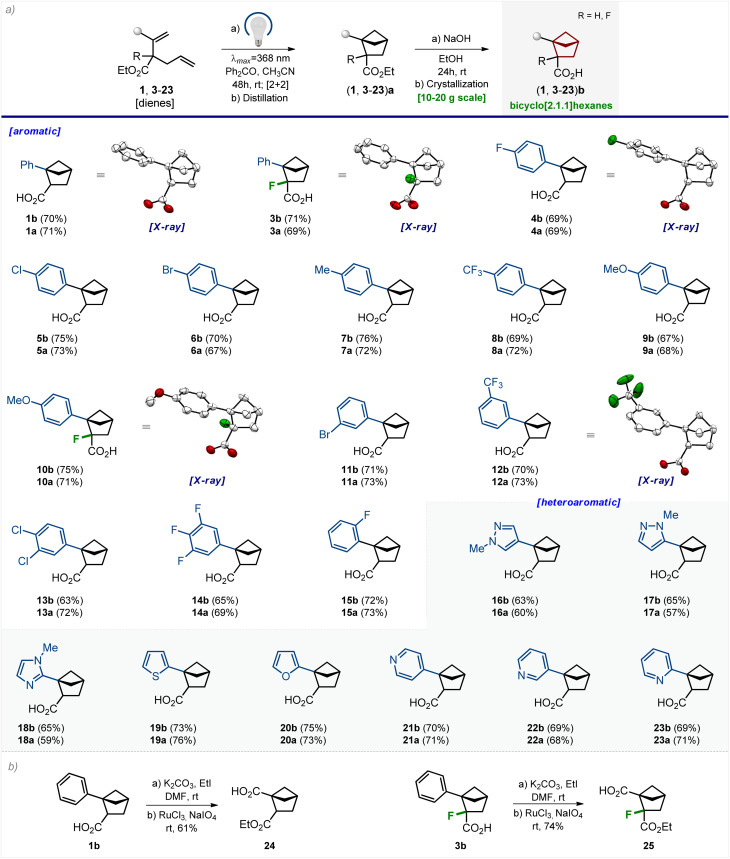
(a) Scope of the reaction. X-ray crystal structure of compounds 1b, 3b, 4b, 10b, and 12b (carbon – white, oxygen – red, and fluorine – green). Hydrogen and chlorine atoms are omitted for clarity. Ellipsoids are shown at a 50% probability level. (b) Synthesis of linkers 24 and 25.

The entire optimized synthetic protocol is shown in Scheme 1. It was important for us to elaborate on a modular method that would provide bicyclo[2.1.1]hexanes employing only available and inexpensive starting materials. The synthesis started from acetophenone. The Horner–Wadsworth–Emmons reaction of acetophenone smoothly gave alkene 2 in 90% yield after distillation. Treatment of the latter with LDA in THF at −78 °C followed by the addition of allyl bromide gave diene 1 in 81% yield (*ca.* 90% purity). The compound contained *ca.* 10% of the isomeric diene, as the undesired alkylation at the methyl group also took place (please see the ESI,† p. S5–S6). An analytically pure sample of diene 1 was obtained by purification with HPLC. In the next step, however, we used the crude material. An intramolecular photocycloaddition of diene 1 proceeded smoothly on scale to provide the desired bicyclo[2.1.1]hexane 1a in 71% yield (*ca.* 90% purity) after distillation. Saponification of the ester group in 1a followed by crystallization from hexane-*t*BuOMe gave pure carboxylic acid 1b as a white crystalline solid in 70% yield. The structure of compound 1b was confirmed by X-ray crystallographic analysis (Scheme 2).^23^

It is important to note that following this optimized sequence, we easily synthesized 23 g of product 1b in one run. No column chromatography was involved at any step.

### Scope

We studied the scope of the developed method. The photochemical reaction tolerated various substituents at the aromatic core (Scheme 2). Among them were an alkyl group (7); fluorine (4, 14, and 15), chlorine (5 and 13) and bromine atoms (6 and 11); methoxy (9 and 10) and trifluoromethyl groups (8 and 12). All three substitution patterns of the benzene ring, – *para* (4–10), *meta* (11–14), and *ortho* (15), – gave the corresponding bicyclo[2.1.1]hexanes 4a–15a in 67–73% yield. The photocycloaddition was also compatible with the fluorine atom directly attached to the diene structure (3 and 10). Various medicinal chemistry-relevant heterocycles, such as pyrazole (16 and 17), imidazole (18), thiophene (19), furane (20), and pyridine (21–23), provided the desired bicyclo[2.1.1]hexanes 16a–23a in 57–76% yield. All products 3a–23a were purified by distillation. Saponification of the ester group in 3a–23a followed by crystallization gave solid carboxylic acids 3b–23b in 63–76% yield.

All final products were synthesized in gram quantities. The structures of bicyclo[2.1.1]hexanes 1b, 3b, 4b, 10b, and 12b were confirmed by X-ray crystallographic analysis (Scheme 2).^23^

### Modifications

Compounds 1b and 3b–23b possess one functional group (–CO_2_H). We aimed to perform some representative modifications of these bicyclo[2.1.1]hexanes to obtain linkers for medicinal chemistry – compounds with two functional groups.

Esterification of carboxylic acid 1b, followed by oxidation of the phenyl group with NaIO_4_/RuCl_3_ gave linker 24 (Scheme 2). The fluorine-substituted linker 25 was synthesized analogously from compound 3b. Both linkers 24 and 25 provide a way to synthesize various 1,2-disubstituted bicyclo[2.1.1]hexanes by standard stepwise modifications of carboxylic groups (amide synthesis, heterocyclizations, radical couplings, *etc.*).^24^

### Crystallographic analysis

**Fig. 3:**
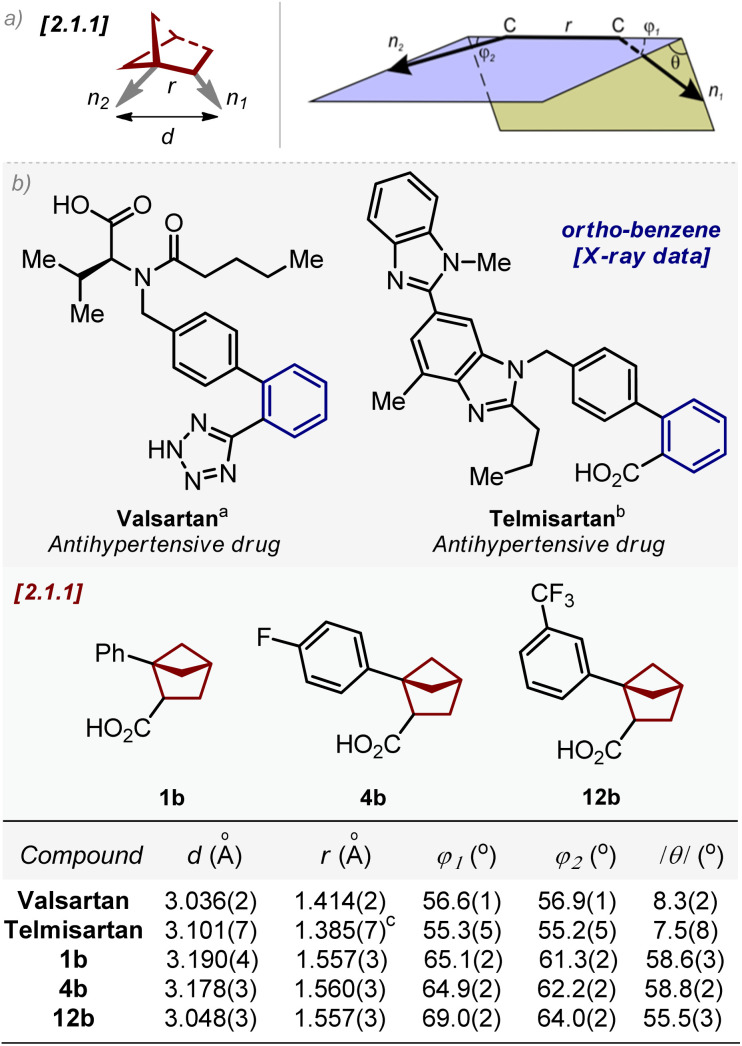
(a) Definition of vectors *n*_1_ and *n*_2_, and geometric parameters *d*, *r*, *φ*_1_, *φ*_2_ and *θ*. 1,2-Disubstituted bicyclo[2.1.1]hexane is shown as an example. (b) Geometric parameters *d*, *r*, *φ*_1_, *φ*_2,_ and |*θ*| for *ortho*-substituted benzenes (*valsartan* and *telmisartan*); and bicyclo[2.1.1]hexanes 1b, 4b, and 12b. ^*a*^Data are taken from ref. 25a. ^*b*^Data are taken from ref. 25b. ^*c*^Two molecules of telmisartan are present in the crystalline lattice with *r* = 1.385(7)Å and 1.395(7)Å (ref. 25b).

Next, we compared the geometric parameters of 1,2-disubstituted bicyclo[2.1.1]hexanes with those of the *ortho*-substituted benzene ring. For this, we employed the exit vector plot tool. In this method, two substituents on the scaffold were simulated by two exit vectors *n*_1_ and *n*_2_ (Fig. 3). The relative spatial arrangement of the vectors is described by four geometric parameters: the distance between C-atoms *r*, the plane angles *φ*_1_ (between vectors *n*_1_ and C–C) and *φ*_2_ (between vectors *n*_2_ and C–C), and the dihedral angle *θ* defined by vectors *n*_1_, C–C and *n*_2_. An additional representative parameter – the distance *d* between two carbon substituents (Fig. 3) – was also measured.

We calculated the values of *d*, *r*, *φ*_1_, *φ*_2_, and /*θ*/of 1,2-disubstituted bicyclo[2.1.1]hexanes from the X-ray data of compounds 1b, 4b, and 12b.^23^

The corresponding parameters for *ortho*-substituted benzene were obtained from the reported crystal data of two antihypertensive drugs – valsartan and telmisartan (Fig. 3).^25^ Analysis of these data revealed that the geometric properties of 1,2-disubstituted bicyclo[2.1.1]hexanes in general were similar to those of *ortho*-substituted benzene. In particular, the distance *d* in bicyclo[2.1.1]hexanes was only 0.1 Å longer than that in the *ortho*-benzene ring: 3.05–3.19 Å (1b, 4b, and 12b) *vs.* 3.04–3.10 Å (*ortho*-benzene).

The distance *r* in bicyclo[2.1.1]hexane was *ca.* 0.1–0.2 Å longer than that in the *ortho*-benzene ring: 1.56 Å (1b, 4b, and 12b) *vs.* 1.39–1.41 Å (*ortho*-benzene).

Angles *φ*_1_ and *φ*_2_ were similar in both scaffolds: 61–65° (1b, 4b, and 12b) *vs.* 55–57° (*ortho*-benzene). The difference in planarity, however, between the saturated scaffold and the benzene ring was significant: while *ortho*-benzene was almost flattened, bicyclo[2.1.1]hexanes had a significant three-dimensional character:/θ/= 56–59° (1b, 4b, and 12b) *vs.* 7–8° (*ortho*-benzene).

In general, both distances *r* and *d* and angles *φ*_1_ and *φ*_2_ of 1,2-disubstituted bicyclo[2.1.1]hexanes were similar to those of the *ortho*-substituted benzene ring.

### Incorporation into bioactive compounds

**Scheme 3:**
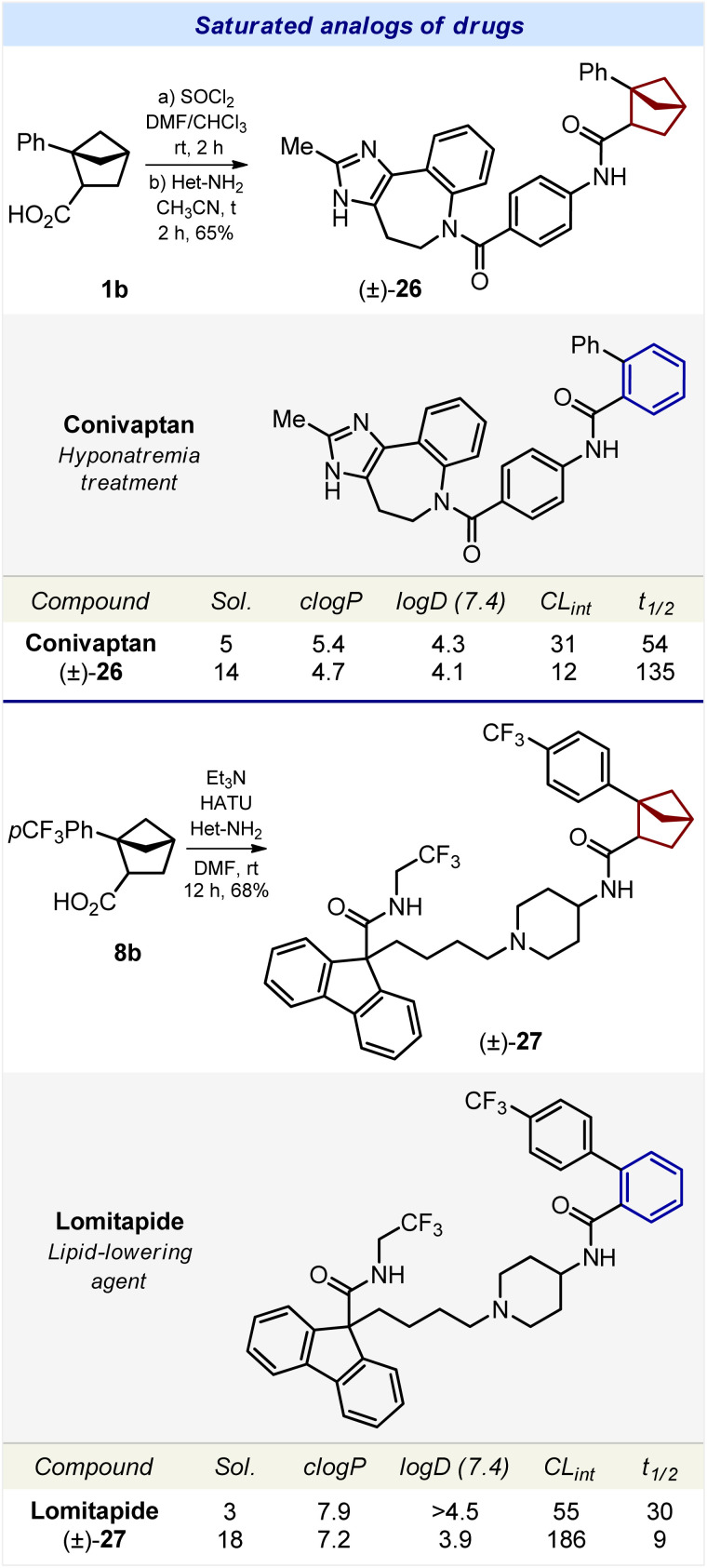
Synthesis and properties of compounds 26 and 27 – saturated analogs of drugs conivaptan and lomitapide, correspondingly. HATU: hexafluorophosphate azabenzotriazole tetramethyl uronium. Sol.: the experimental kinetic solubility in phosphate-buffered saline, pH 7.4 (μM). *c* log *P*: the calculated lipophilicity. log *D* (7.4): the experimental distribution coefficient in *n*-octanol/phosphate-buffered saline, pH 7.4. Reliable log *D* values could be obtained within a range of 1.0–4.5. CL_int_: the experimental metabolic stability in human liver microsomes (μL min^−1^ mg^−1^). *t*_1/2_ (min): the experimental half-time of metabolic decomposition.

**Scheme 4:**
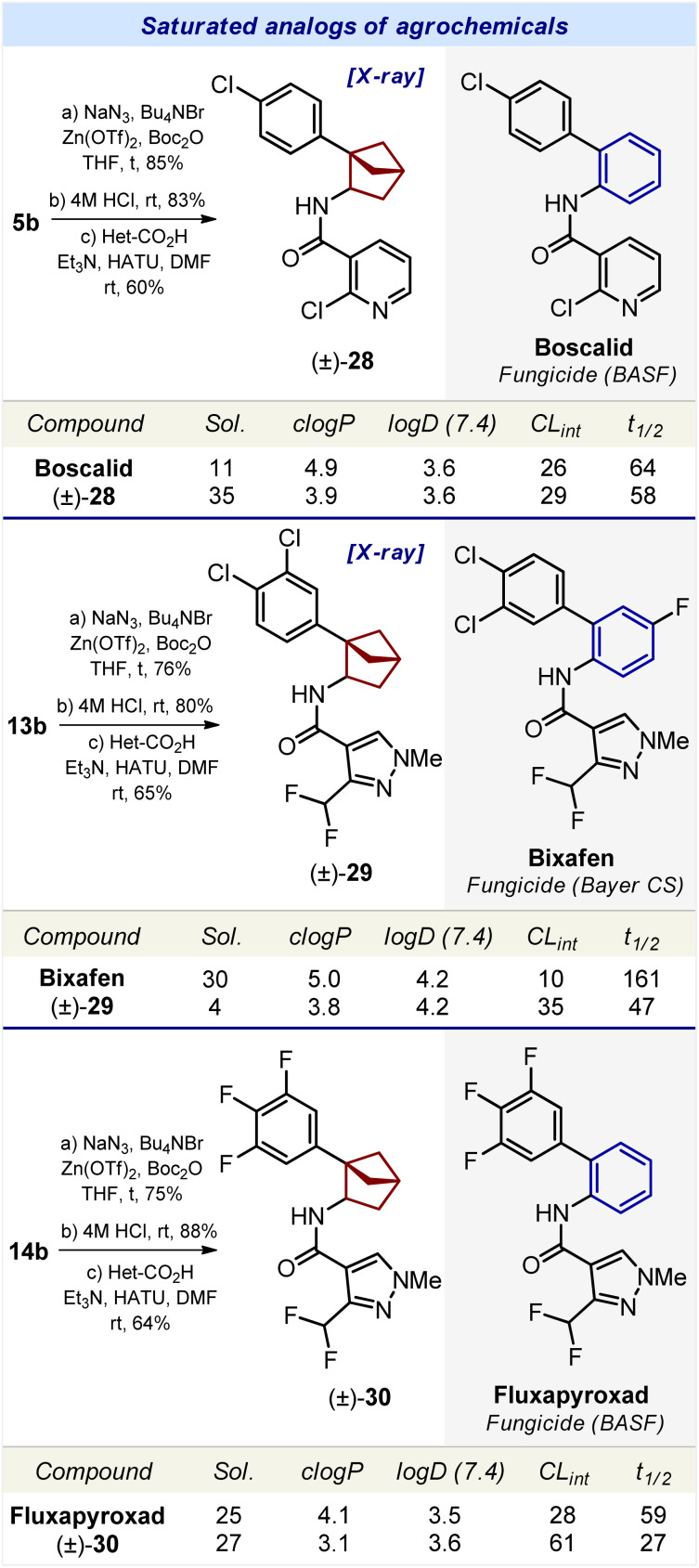
Synthesis and properties of compounds 28–30 – saturated analogs of agrochemicals boscalid, bixafen, and fluxapyroxad, correspondingly. Sol.: the experimental kinetic solubility in phosphate-buffered saline, pH 7.4 (μM). *c* log *P*: the calculated lipophilicity. log *D* (7.4): the experimental distribution coefficient in *n*-octanol/phosphate-buffered saline, pH 7.4. Reliable log *D* values could be obtained within a range of 1.0–4.5. CL_int_, clearance intrinsic (μL min^−1^ mg^−1^): experimental metabolic stability in human liver microsomes. *t*_1/2_ (min): the experimental half-time of metabolic decomposition.

The incorporation of the bicyclo[2.1.1]hexane scaffold into bioactive compounds was then attempted. We chose five drugs and agrochemicals with an *ortho*-substituted benzene ring: agent for hyponatremia treatment conivaptan, lipid-lowering agent lomitapide (Scheme 3); fungicides boscalid, bixafen*,* and fluxapyroxad (Scheme 4).

Synthesis of the saturated analog of conivaptan was performed from carboxylic acid 1b (Scheme 3). Amide coupling with the corresponding *para*-substituted aniline gave the desired compound 26. Compound 27, – a saturated analog of lomitapide, – was obtained analogously from carboxylic acid 8b (Scheme 3).

Synthesis of the saturated analog of boscalid was performed from carboxylic acid 5b (Scheme 4). The Curtius reaction followed by acylation of the intermediate amine with 2-chloropyridine-3-carboxylic acid gave compound 28. Using an analogous strategy, compound 29, – a saturated analog of bixafen, – was obtained from carboxylic acid 13b (Scheme 4). The saturated analog of fluxapyroxad, compound 30, was synthesized from carboxylic acid 14b.

The structures of bicyclo[2.1.1]hexanes 28 and 29 were confirmed by X-ray crystallographic analysis.^23^

### Physicochemical parameters

We studied the effect of the replacement of the *ortho*-substituted benzene ring by bicyclo[2.1.1]hexanes on the physicochemical properties of bioactive compounds (Schemes 3 and 4).

#### Water solubility

Replacement of the *ortho*-benzene ring in conivaptan by bicyclo[2.1.1]hexane (26) increased the solubility by threefold: 5 μM (conivaptan) *vs.* 14 μM (26) (Scheme 3). An analogous trend was observed with lomitapide. Replacement of the benzene ring in lomitapide with bicyclo[2.1.1]hexane (27) led to a dramatic sixfold increase in solubility: 3 μM (lomitapide) *vs.* 18 μM (27).

Replacement of the *ortho*-benzene ring by bicyclo[2.1.1]hexane in agrochemicals boscalid, bixafen, and fluxapyroxad produced conflicting results. In boscalid, such a replacement led to a significant threefold increase in solubility: 11 μM (boscalid) *vs.* 35 μM (28). In bixafen, the opposite effect was observed, and the solubility was reduced: 30 μM (bixafen) *vs.* 4 μM (29).^26^ In fluxapyroxad, such a replacement resulted in a slight increase in solubility: 25 μM (fluxapyroxad) *vs.* 27 μM (30).

In conclusion, in four out of the five bioactive compounds, the replacement of the *ortho*-benzene ring by bicyclo[2.1.1]hexane led to an enhanced water solubility.

#### Lipophilicity

To estimate the influence of the replacement of the *ortho*-benzene ring with bicyclo[2.1.1]hexane on lipophilicity, we used two parameters: calculated lipophilicity (*c* log *P*)^27^ and experimental lipophilicity (log *D*).

Replacement of the *ortho*-benzene ring in conivaptan, lomitapide, boscalid, bixafen, and fluxapyroxad with bicyclo[2.1.1]hexane (26–30) led to a decrease in *c* log *P* by 0.7–1.2 units.

Replacement of the *ortho*-benzene ring with bicyclo[2.1.1]hexane had only a small effect on the logD index. In four bioactive compounds (conivaptan, boscalid, bixafen, and fluxapyroxad) such a replacement almost did not affect log *D*. Only in lomitapide, the saturated analog 27 had a significantly lower log *D* index: >4.5 (lomitapide) *vs.* 3.9 μM (27).

In summary, in all five bioactive compounds, replacement of the *ortho*-benzene ring by bicyclo[2.1.1]hexane led to a decrease in calculated lipophilicity (*c* log *P*) by 0.7–1.2 units; and in four of them it had little effect on the experimental lipophilicity (log *D*).

#### Metabolic stability

The effect of bicyclo[2.1.1]hexane on the metabolic stability of bioactive compounds was more complex. In conivaptan, the incorporation of bicyclo[2.1.1]hexane (26) increased the metabolic stability: CL_int_ (μL min^−1^ mg^−1^) = 31 (conivaptan) *vs.* 12 (26). In lomitapide, bixafen*,* and fluxapyroxad the incorporation of bicyclo[2.1.1]hexane (27, 29, and 30) dramatically decreased the metabolic stability by two (30) to three (27, 29) times, as measured by using *t*_1/2_ (min). In boscalid, such a replacement led to a slight decrease in metabolic stability: CL_int_ (μL min^−1^ mg^−1^) = 26 (boscalid) *vs.* 29 (28) (Schemes 3 and 4).

In brief summary, in four out of the five bioactive compounds, replacement of the *ortho*-benzene ring with bicyclo[2.1.1]hexane decreased the metabolic stability.

### Bioactivity

**Fig. 4:**
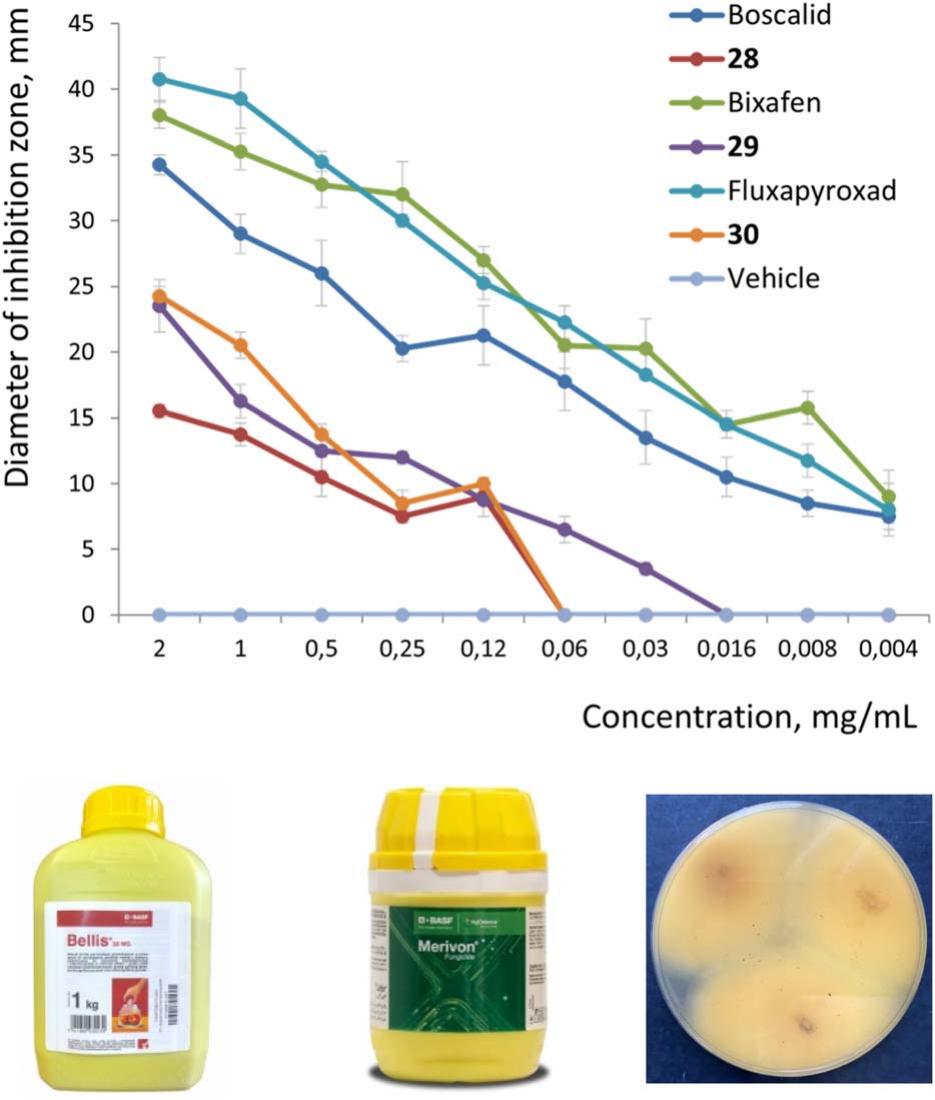
Inhibition of growth of *Aspergillus niger* (strain VURV-F 822; measured as a diameter of the inhibition zone, mm) with boscalid, bixafen, fluxapyroxad, and their analogs 28–30 at different concentrations after 48 h of incubation. Fungicides boscalid (@Bellis) and bixafen (@Merivon) are shown. Inhibition of fungal growth by compound 29.

Finally, we wanted to answer the key question, – can 1,2-disubstituted bicyclo[2.1.1]hexanes indeed act as bioisosteres of the *ortho*-benzene ring in bioactive compounds?^9^ Therefore, we measured the antifungal activity of the marketed fungicides boscalid (BASF), bixafen (Bayer CS), fluxapyroxad (BASF), and their saturated analogs 28–30. In strict contrast to medicinal chemistry, the use of racemic mixtures in agrochemistry is common;^5^ therefore for the validation of the proof-of-concept, we directly studied the biological activity of the available racemic compounds 28–30 (Fig. 4).

We measured the antifungal activity of all six compounds against the fungal strain *Aspergillus niger*, – using the disk diffusion method (ESI, p. S327–S333†). Even though the original agrochemicals were more potent, all three saturated analogs 28–30 were active and showed a high inhibition of the growth of *Aspergillus niger* compared to the vehicle (Fig. 4; and ESI, p. S327–S333†).

## Conclusions

In this work, we have synthesized, characterized, and biologically validated 1,2-disubstituted bicyclo[2.1.1]hexanes as saturated bioisosteres of the *ortho*-substituted benzenes. These structures were obtained from readily available and inexpensive starting materials (acetophenone) on a multigram scale (Schemes 1 and 2). Physicochemical and geometric properties of bicyclo[2.1.1]hexanes were measured and compared to those of the *ortho*-substituted benzenes (Fig. 3). The incorporation of the bicyclo[2.1.1]hexane core into the structure of agrochemicals boscalid (BASF), bixafen (Bayer CS), and fluxapyroxad (BASF) gave the saturated patent-free analogs 28–30 with high antifungal activity.

We believe that given the commonplace of the phenyl group in chemistry, its saturated bioisosteres developed here will become common in medicinal chemistry in the coming years.

## Data availability

The ESI† contains method description, product characterization data, and NMR spectra.

## Author contributions

A. D. and P. K. M. designed the project. A. D., P. G., Y. M., O. S., G. A.-M., and R. K. carried out the experiments. I. V. S. and P. K. M. wrote the manuscript and all authors provided comments.

## Conflicts of interest

A. D., P. G., Y. M., O. S., G. A.-M., R. K., I. V. S., and P. K. M. are employees of a chemical supplier Enamine.

## Supplementary Material

SC-014-D3SC05121H-s001

SC-014-D3SC05121H-s002
