# Supplementary material for: 1,2-Disubstituted bicyclo[2.1.1]hexanes as saturated bioisosteres of ortho-substituted benzene
Source: Chem Sci. 2023 Oct 27;14(48):14092–9. doi: 10.1039/d3sc05121h (PMC10718076; doi:10.1039/d3sc05121h)
Supplement: SC-014-D3SC05121H-s001 [file SC-014-D3SC05121H-s001.pdf]

Electronic Supplementary Material (ESI) for Chemical Science.
This journal is © The Royal Society of Chemistry 2023

Table of Contents

Experimental Section. Data description and procedures S4
Copies of ^{1}H, ^{13}C{^{1}H} and ^{19}F{^{1}H} spectra S50
Compound 1 .... S52
Compound (±)-1a S54
Compound (±)-1b S56
Compound 3 .... S61
Compound (±)-3a S64
Compound (±)-3b S67
Compound 4 .... S73
Compound (±)-4a S76
Compound (±)-4b S79
Compound 5 .... S84
Compound (±)-5b S88
Compound 6 .... S92
Compound (±)-6a S94
Compound (±)-6b S96
Compound 7 .... S100
Compound (±)-7a S102
Compound (±)-7b S104
Compound 8 .... S109
Compound (±)-8a S112
Compound (±)-8b S115
Compound 9 .... S120
Compound (±)-9a S122
Compound (±)-9b S124
Compound 10 .... S129
Compound (±)-10a S132
Compound (±)-10b S135
Compound 11 .... S140
Compound (±)-11a S142
Compound (±)-11b S144
Compound 12 .... S149

Compound (±)-12a S152
Compound (±)-12b S155
Compound 13 .... S160
Compound (±)-13a S162
Compound (±)-13b S164
Compound 14 .... S169
Compound (±)-14a S172
Compound (±)-14b S175
Compound 15 .... S181
Compound (±)-15a S184
Compound (±)-15b S187
Compound 16 .... S192
Compound (±)-16a S194
Compound (±)-16b S196
Compound 17 .... S200
Compound (±)-17a S202
Compound (±)-17b S204
Compound 18 .... S208
Compound (±)-18a S210
Compound (±)-18b S212
Compound 19 .... S216
Compound (±)-19a S218
Compound (±)-19b S220
Compound (±)-20a S224
Compound (±)-20b S226
Compound 21 .... S230
Compound (±)-21a S232
Compound (±)-21b S234
Compound 22 .... S238
Compound (±)-22a S240
Compound (±)-22b S242
Compound 23 .... S246
Compound (±)-23a S248
Compound (±)-23b S250
Compound (±)-24 .... S252

Compound (±)-25 .... S254
Compound (±)-26 .... S257
Compound (±)-27 .... S259
Compound (±)-28 .... S266
Compound (±)-29 .... S272
Compound (±)-30 .... S280
Crystallographic data (X-Ray) S283
Analysis of Aqueous Solubility S304
Determination of Distribution Coefficient (LogD, pH 7.4) S314
Assessment of Metabolic Stability in Human Liver Microsomes S318
Bioactivity S327

Experimental Section. Data description and procedures

General Considerations. All chemicals were provided by Enamine Ltd. (www.enamine.net). All solvents were treated according to standard methods. All reactions were monitored by thinlayer chromatography (TLC) and were visualized using UV light. Product purification was performed using HPLC: AGILENT 1260 INFINITY, a column Chromatorex C18 SMB 100-5T, 100 × 19 mm, 5 microm; PuriFlash XS420 Plus or by distillation under a reduce pressure. ^{1}H NMR spectra were recorded at 400, 500 or 600 MHz (Varian); ^{19}F-NMR spectra were recorded at 376 MHz (Varian) and ^{13}C NMR spectra were recorded at 100, 126 or 151 MHz (Varian). ^{1}H NMR chemical shifts are calibrated using residual undeuterated solvents $CHCl_3$ (δ = 7.26 ppm) or DMSO (δ = 2.50 ppm). ^{13}C-NMR chemical shifts for ^{13}C-NMR are reported relative to the central $CHCl_3$ (δ = 77.16 ppm) or DMSO (δ = 39.52 ppm). Coupling constants are given in Hz. High-resolution mass spectra (HRMS) were recorded on an Agilent LC/MSD TOF mass spectrometer by electrospray ionization time of flight reflectron experiments

General procedure A (ethyl-3-phenylbut-2-enoate (2) as an example)

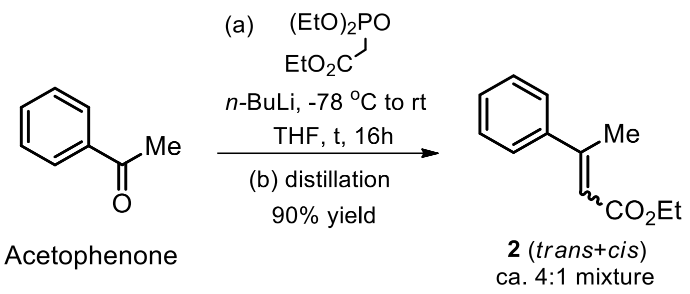

Ethyl-3-phenylbut-2-enoate (2)

To a solution of ethyl 2-(diethoxyphosphoryl)acetate (100.00 g, 0.51 mol, 1.33 equiv) in THF (700 mL) was added dropwise *n*-BuLi (2.5 M, 204 mL, 0.51 mol, 1.33 equiv) at -40 °C under argon over 15 min. The resulting mixture was stirred for 15 min at the same temperature, and then a solution of acetophenone (45.60 g, 0.38 mol, 1.00 equiv) in THF (100 mL) was added dropwise at the same temperature over 15 min. The mixture was warmed to room temperature and left at this temperature for 16 h. The mixture was concentrated under reduced pressure and diluted with water (300 mL). The solution was extracted with MeO*t*Bu (2 × 300 mL). The combined organic layers were dried over Na_2SO_4, filtered and concentrated under reduced procure. The final product was purified by distillation (b.p. = 55-56 °C, 0.1 mmHg). Yield: 64.60 g, 0.34 mol, 90%, colorless oil. A (*trans*+*cis*)-mixture of isomers: ~4:1. ^{1}H NMR (500 MHz, $CDCl_3$): δ 7.74 – 7.40 (m, 2H), 7.48 – 6.91 (m, 3H), 6.14 (s), 5.91 (s) 1H, 4.22 (q, J = 7.1 Hz), 4.00 (q, J = 7.1 Hz) 2H, 2.58 (s), 2.18 (s) 3H, 1.32 (t, J = 7.1 Hz), 1.08 (t, J = 7.1 Hz) 3H ppm. ^{13}C{^{1}H} NMR (101 MHz, $CDCl_3$): δ 167.0, 155.6, 155.5, 142.4, 129.1, 128.6, 128.0, 127.9, 126.9, 126.4, 117.9, 117.3, 60.0, 59.9, 27.3, 18.1, 14.5, 14.1 ppm. HRMS (ESI-TOF) *m/z*: $[M + H]^+$ calcd for $C_{12}H_{15}O_2$, 191.1072; found 191.1066.

General procedure B (1 as an example)

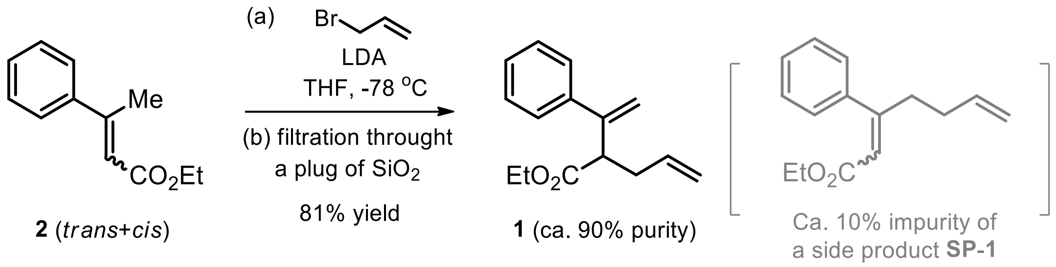

Ethyl 2-(1-phenylvinyl)pent-4-enoate (1)

To freshly prepared LDA (*n*-BuLi, 2.5 M, 144 mL, 0.36 mol, 1.25 equiv and DIPA 36.36 g, 0.36 mol, 1.25 equiv in THF (150 mL)) was added ethyl-3-phenylbut-2-enoate (**2**) (55.10 g, 0.29 mol, 1.00 equiv) dropwise at – 78 °C under argon over 15 min. The mixture was warmed to -10 °C, then

cooled again to -78 °C and 3-bromoprop-1-ene (36.84 g, 0.30 mol, 1.05 equiv) was added dropwise at the same temperature over 15 min. The mixture was allowed to warm slowly to 10 °C, and a solution of citric acid (50 g in 300 mL of water) was added to the mixture. THF was removed under reduced pressure. The residue was extracted with hexane (2 × 300 mL). The combined organic layers were washed with water (2 × 500 mL), dried over Na_2SO_4, filtered through a SiO_2 pad (~5 cm, h = 15 cm). The solvent was removed on a rotary evaporator, and the crude product was used in a next step without further purification. Yield: 54.05 g, purity ~90%, 0.235 mol, 81%, yellow oil.

An analytically pure sample of product **1** was obtained by purification of the sample of the crude mixture by HPLC: Rt = 0-6 min, water/acetonitrile, 50-80%, flow 30 mL/min (loading pump 4 mL/min), column: Chomatorex 18 SMB100-5T, 100 × 19 mm, 5 µm. ^{1}H NMR (500 MHz, $CDCl_3$): δ 7.40 (d, *J* = 7.2 Hz, 2H), 7.33 (t, *J* = 7.3 Hz, 2H), 7.28 (t, *J* = 7.2 Hz, 1H), 5.85 – 5.72 (m, 1H), 5.41 (s, 1H), 5.29 (s, 1H), 5.07 (dd, *J* = 17.1, 1.4 Hz, 1H), 5.01 (d, *J* = 10.0 Hz, 1H), 4.14 (q, *J* = 7.1 Hz, 2H), 3.61 (dd, *J* = 8.9, 6.1 Hz, 1H), 2.71 – 2.61 (m, 1H), 2.44 (dt, *J* = 13.0, 6.3 Hz, 1H), 1.20 (t, *J* = 7.1 Hz, 3H) ppm. ^{13}C{^{1}H} NMR (151 MHz, $CDCl_3$): δ 173.3, 146.6, 141.4, 135.6, 128.4, 127.8, 126.7, 116.9, 115.0, 60.8, 50.4, 36.3, 14.3 ppm. LCMS (M+H): 231. HRMS (ESI-TOF) *m/z*: [M + H]$^+$ calcd for $C_{15}H_{19}O_2$, 231.1385; found 231.1381.

General procedure C for photocyclization (compound 1a as an example)

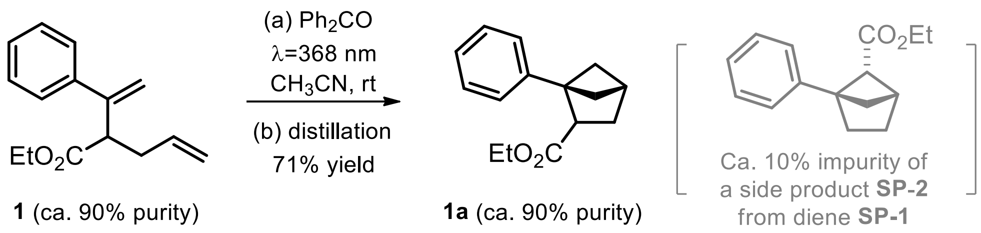

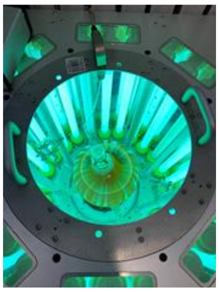

(±)-Ethyl 1-phenylbicyclo[2.1.1]hexane-2-carboxylate (1a)

The solution of ethyl 2-(1-phenylvinyl)pent-4-enoate (52.90 g, purity 90%, 0.23 mol, 1.00 equiv) from the previous step and benzophenone (4.19 g, 0.023 mol, 0.10 equiv) in dry CH_3CN (4 L) was degassed by the bubbling of argon for 15 min. The flask was closed by a septum and irradiated with luminescent UV lamps, 368 nm (24 lamps: Sylvania 368 Blacklight F25/T8/18/BL3368; each lamp has power 25 W; total power is 600 W), under stirring at room temperature for 48 h. The reaction mixture was concentrated under reduced pressure to provide the crude product. The final product was purified by distillation (b.p. = 85-86 °C, 0.1 mmHg). Yield: 37.49 g, purity ~90%, 0.163 mol, 71%, colorless oil.

An analytically pure sample of product **1a** was obtained by purification of the sample of the crude mixture by HPLC: Rt = 0-6 min, water/MeOH, 50-80%, flow 30 mL/min (loading pump 4 mL/min), column: XBridge BEH C18, 100 × 19 mm, 5 µm. ^{1}H NMR (500 MHz, DMSO-d_6): δ 7.27 (t, J = 7.4 Hz, 2H), 7.18 (t, J = 7.3 Hz, 1H), 7.13 (d, J = 7.1 Hz, 2H), 3.94 – 3.74 (m, 2H), 3.03 (dd, J = 8.3, 3.6 Hz, 1H), 2.48 (br. s, 1H), 2.11 (t, J = 9.7 Hz, 1H), 2.04 (dd, J = 9.5, 6.8 Hz, 1H), 1.94 (d, J = 10.7 Hz, 1H), 1.79 – 1.57 (m, 3H), 0.86 (t, J = 7.1 Hz, 3H) ppm. ^{13}C{^{1}H} NMR (126 MHz, DMSO-d_6): δ 174.3, 141.8, 127.9, 126.2, 125.7, 59.3, 57.9, 47.5, 45.9, 37.4, 34.5, 33.4, 13.8 ppm. LCMS (M+H): 231. HRMS (ESI-TOF) *m/z*: $[M + H]^+$ calcd for $C_{15}H_{19}O_2$, 231.1385; found 231.1377.

Irradiation with 368 nm was performed using 24 lamps (25W each)
“Sylvania 368 Blacklight F25/T8/18/BL3368”
https://www.sylvania-lighting.com/product/en-int/products/0002166/
Irradiation was performed until the disappearance of the starting material (ca. 48h).

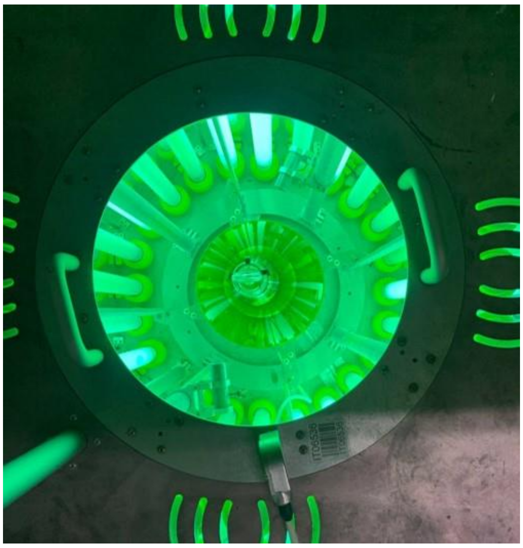
Photochemical step.

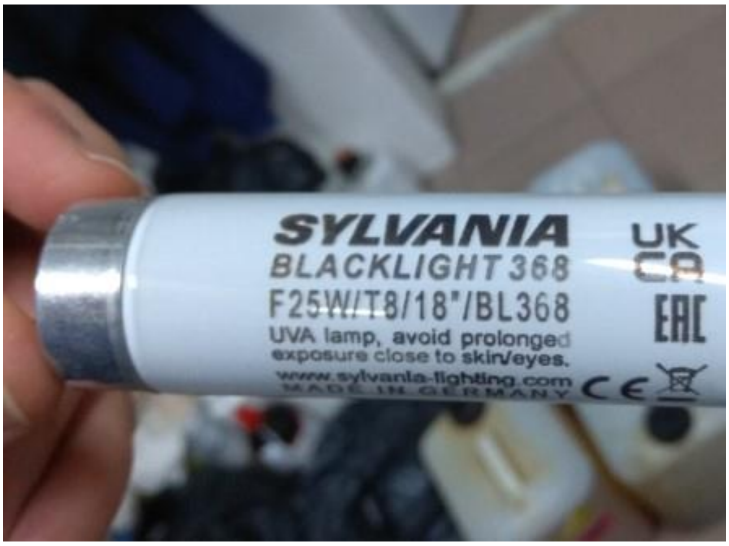

Type of lamp (368 nm)

Mark of lamp: 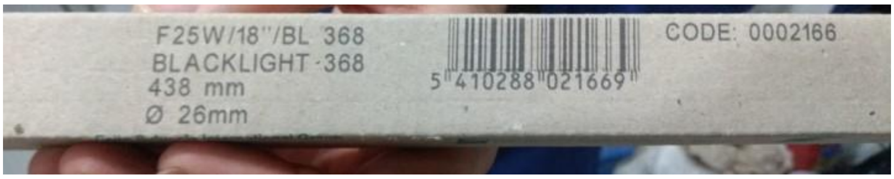

General procedure D (1b as an example)

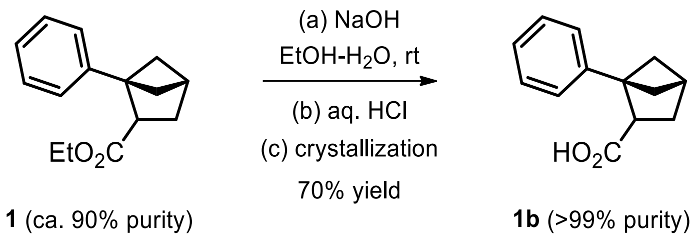

(±)-1-Phenylbicyclo[2.1.1]hexane-2-carboxylic acid (1b)

To a cold solution of NaOH (13.04 g, 0.326 mol, 2.00 equiv) in 100 mL of EtOH/H_2O (85/15; v/v) was added a solution of crude **1a** (37.49 g, purity ~90%, 0.163 mol, 1.00 equiv) obtained in a previous step in EtOH (300 mL). The reaction mixture was stirred at room temperature for 12 h, and then the solvents were removed under reduced pressure. The residue was dissolved in 200 mL of water and washed with CH_2Cl_2 (2 × 100 mL). An aqueous layer was acidified with concentrated HCl to pH ~ 2 and extracted with CH_2Cl_2 (3 × 150 mL). The organic layers were combined, dried over Na_2SO_4, filtered and evaporated to dryness. The crude product was recrystallized from a hexane-MeO*t*Bu mixture ~9:1. Yield: 23.03 g, 0.114 mol, 70%, white solid, m.p. = 119-120 °C. ^{1}H NMR (500 MHz, DMSO-d$_6$): δ 11.81 (s, 1H), 7.25 (t, *J* = 7.6 Hz, 2H), 7.16 (d, *J* = 7.3 Hz, 3H), 2.96 (dd, *J* = 8.5, 3.7 Hz, 1H), 2.44 (s, 1H), 2.11 (t, *J* = 9.9 Hz, 1H), 2.07 (dd, *J* = 9.7, 6.7 Hz, 1H), 1.89 (d, *J* = 10.7 Hz, 1H), 1.78 – 1.73 (m, 1H), 1.69 – 1.63 (m, 1H), 1.59 (dd, *J* = 9.6, 6.5 Hz, 1H) ppm. ^{13}C{^{1}H} NMR (126 MHz, DMSO-d$_6$): δ 176.1, 142.1, 127.9, 126.1, 125.9, 57.5, 47.2, 46.3, 37.6, 34.5, 33.9 ppm. LCMS (M-H): 201. HRMS (ESI-TOF) *m/z*: $[M - H]^-$ calcd for $C_{13}H_{13}O_2$, 201.0916; found 201.0919.

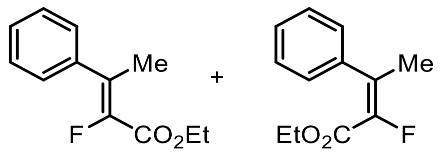

Ethyl-2-fluoro-3-phenylbut-2-enoate

General procedure A was used with $(EtO)_2(O)P-CHF(CO_2Et)$. The final product was purified by distillation (b.p. = 57-58 °C, 0.1 mmHg). Yield: 64.06 g, 0.308 mol, 77%, colorless oil. A mixture of *cis*+*trans*-isomers: ~4:1. ^{1}H NMR (500 MHz, $CDCl_3$): δ 7.42 – 7.27 (m, 3H), 7.22 – 7.08 (m, 2H), 4.34 (q, *J* = 7.1 Hz), 4.05 (q, *J* = 7.1 Hz) 2H, 2.45 (d, *J* = 3.5 Hz), 2.15 (d, *J* = 4.4 Hz) 3H, 1.38 (t, *J* = 7.1 Hz), 1.03 (t, *J* = 7.1 Hz) 3H ppm. ^{13}C{^{1}H} NMR (151 MHz, $CDCl_3$): δ 162.0 (d, *J* = 34.6 Hz), 160.8 (d, *J* = 36.2 Hz), 144.4 (d, *J* = 251.6 Hz), 138.7 (d, *J* = 5.4 Hz), 131.5 (d, *J* = 17.0 Hz), 130.8 (d, *J* = 11.3 Hz), 128.5, 128.4, 128.2, 128.1 (d, *J* = 4.0 Hz), 127.9, 127.5 (d, *J* = 3.0 Hz), 61.5, 61.1, 19.5 (d, *J* = 6.5 Hz), 18.4, 14.3, 13.8 ppm. ^{19}F{^{1}H} NMR (376 MHz, $CDCl_3$): δ -124.5 (s), -

126.4 (s) ppm. LCMS (M+H): 209. HRMS (ESI-TOF) *m/z*: $[M + H]^+$ calcd for $C_{12}H_{14}FO_2$, 209.0978; found 209.0971.

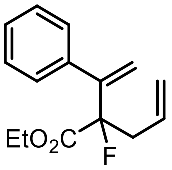

Ethyl 2-fluoro-2-(1-phenylvinyl)pent-4-enoate (3)

General procedure B was used. Yield: 41.17 g, purity ~90%, 0.166 mol, 83%, colorless oil.

An analytically pure sample of the product was obtained by purification of the sample of the crude mixture by HPLC: Rt = 0-7 min, water/acetonitrile, 40-65%, flow 30 mL/min (loading pump 4 mL/min), column: Chomatorex 18 SMB100-5T, 100×19 mm, 5 µm. ^1H NMR (500 MHz, $CDCl_3$): δ 7.31 (s, 5H), 5.89 – 5.77 (m, 1H), 5.61 (d, *J* = 2.3 Hz, 1H), 5.42 (s, 1H), 5.18 (s, 1H), 5.15 (d, *J* = 6.3 Hz, 1H), 4.27 – 4.10 (m, 2H), 2.88 (t, *J* = 8.0 Hz, 1H), 2.84 (d, *J* = 6.8 Hz, 1H), 1.19 (t, *J* = 7.1 Hz, 3H) ppm. $^{13}C\{^1H\}$ NMR (126 MHz, $CDCl_3$): δ 169.6 (d, *J* = 26.7 Hz), 146.6 (d, *J* = 20.2 Hz), 138.5, 130.8 (d, *J* = 3.2 Hz), 128.5, 128.2, 128.0, 120.0, 118.4 (d, *J* = 8.6 Hz), 96.8 (d, *J* = 189.3 Hz), 61.9, 40.7 (d, *J* = 22.3 Hz), 14.1 ppm. $^{19}F\{^1H\}$ NMR (376 MHz, $CDCl_3$): δ -155.7 (s) ppm. HRMS (ESI-TOF) *m/z*: $[M + H]^+$ calcd for $C_{15}H_{18}FO_2$, 249.1291; found 249.1282.

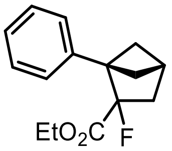

(±)-Ethyl 2-fluoro-1-phenylbicyclo[2.1.1]hexane-2-carboxylate (3a)

General procedure C was used. The final product was purified by distillation (b.p. = 80-81 °C, 0.1 mmHg). Yield: 17.11 g, purity ~90%, 0.069 mol, 69%, colorless oil.

An analytically pure sample of the product was obtained by purification of the sample of the crude mixture by HPLC: Rt = 0-2-9 min, water/acetonitrile, 42-50-80%, flow 30 mL/min (loading pump 4 mL/min), column: Chomatorex 18 SMB100-5T, 100×19 mm, 5 µm. ^1H NMR (500 MHz, $CDCl_3$): δ 7.30 – 7.22 (m, 3H), 7.17 (d, *J* = 7.5 Hz, 2H), 4.12 – 3.98 (m, 2H), 2.65 (t, *J* = 14.3 Hz, 1H), 2.56 (s, 1H), 2.44 – 2.37 (m, 1H), 2.32 – 2.27 (m, 1H), 2.17 (ddd, *J* = 27.0, 12.2, 3.8 Hz, 1H), 2.01 – 1.93 (m, 2H), 1.01 (t, *J* = 7.1 Hz, 3H) ppm. $^{13}C\{^1H\}$ NMR (126 MHz, $CDCl_3$): δ 171.3 (d, *J* = 28.8 Hz), 138.6, 128.1, 127.1, 126.7, 101.2 (d, *J* = 204.6 Hz), 63.1 (d, *J* = 21.6 Hz), 61.4, 43.1 (d, *J* = 5.0 Hz), 42.7, 42.6, 42.5 (d, *J* = 3.3 Hz), 33.3, 14.1 ppm. $^{19}F\{^1H\}$ NMR (376 MHz, $CDCl_3$): δ -159.5 (s) ppm. HRMS (ESI-TOF) *m/z*: $[M + H]^+$ calcd for $C_{15}H_{18}FO_2$, 249.1291; found 249.1283.

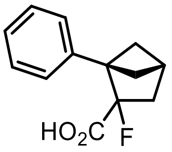

(±)-2-Fluoro-1-phenylbicyclo[2.1.1]hexane-2-carboxylic acid (3b)

General procedure D was used. Yield: 10.12 g, 0.046 mol, 71%, white solid, m.p. = 133-134 °C. ^{1}H NMR (500 MHz, DMSO-d$_6$): δ 13.08 (s, 1H), 7.29 (t, *J* = 7.3 Hz, 2H), 7.23 (t, *J* = 7.2 Hz, 1H), 7.15 (d, *J* = 7.1 Hz, 2H), 2.51 – 2.43 (m, 2H), 2.41 – 2.32 (m, 1H), 2.18 – 2.02 (m, 2H), 2.01 – 1.86 (m, 2H) ppm. ^{13}C{^{1}H} NMR (126 MHz, DMSO-d$_6$): δ 172.2 (d, *J* = 29.9 Hz), 138.6, 127.9, 126.8, 126.6, 100.2 (d, *J* = 201.6 Hz), 62.1 (d, *J* = 21.8 Hz), 42.7 (d, *J* = 4.8 Hz), 42.6, 42.4, 42.4, 32.6 ppm. ^{19}F{^{1}H} NMR (376 MHz, DMSO-d$_6$): δ -155.8 (s) ppm. LCMS (M-H): 219. HRMS (ESI-TOF) *m/z*: [M - H]$^-$ calcd for $C_{13}H_{12}FO_2$, 219.0821; found 219.0817.

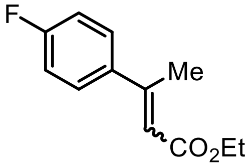

Ethyl-3-(4-fluorophenyl)but-2-enoate

General procedure A was used. The final product was purified by distillation (b.p. = 59-60 °C, 0.1 mmHg). Yield: 73.08 g, 0.348 mol, 87%, colorless oil. A mixture of *cis*+*trans*-isomers: ~4:1. ^{1}H NMR (500 MHz, CDCl$_3$): δ 7.52 – 7.40 (m, 2H), 7.23 – 7.14 (m), 7.11 – 6.99 (m) 2H, 6.09 (s), 5.91 (s) 1H, 4.21 (q, *J* = 7.1 Hz), 4.01 (q, *J* = 7.1 Hz) 2H, 2.55 (d, *J* = 1.1 Hz), 2.16 (d, *J* = 1.3 Hz) 3H, 1.31 (t, *J* = 7.1 Hz), 1.11 (t, *J* = 7.1 Hz) 3H ppm. ^{13}C{^{1}H} NMR (151 MHz, CDCl$_3$): δ 166.9, 165.9, 164.2, 162.5, 161.7, 154.4, 154.3, 138.4 (d, *J* = 3.3 Hz), 128.9 (d, *J* = 8.1 Hz), 128.2 (d, *J* = 8.3 Hz), 118.2, 117.3, 115.6 (d, *J* = 21.5 Hz), 115.0 (d, *J* = 21.6 Hz), 60.03, 59.97, 27.3, 18.1, 14.5, 14.2 ppm. ^{19}F{^{1}H} NMR (376 MHz, CDCl$_3$): δ -113.1 (s), -114.8 (s) ppm. LCMS (M+H): 209. HRMS (ESI-TOF) *m/z*: [M + H]$^+$ calcd for $C_{12}H_{14}FO_2$, 209.0978; found 209.0971.

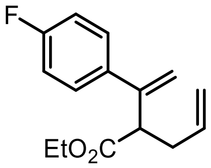

Ethyl 2-(1-(4-fluorophenyl)vinyl)pent-4-enoate (4)

General procedure B was used. Yield: 39.18 g, purity ~90%, 0.158 mol, 79%, colorless oil.

An analytically pure sample of the product was obtained by purification of the sample of the crude mixture by HPLC: Rt = 0-2-9 min, acetonitrile/water, 38-45-70%, flow 30 mL/min (loading pump 4 mL/min), column: Chomatorex 18 SMB100-5T, 100×19 mm, 5 µm. ^{1}H NMR (500 MHz, CDCl$_3$): δ

7.40 – 7.32 (m, 2H), 7.01 (t, *J* = 8.7 Hz, 2H), 5.83 – 5.70 (m, 1H), 5.36 (s, 1H), 5.27 (s, 1H), 5.07 (dd, *J* = 17.1, 1.5 Hz, 1H), 5.02 (d, *J* = 10.2 Hz, 1H), 4.13 (q, *J* = 7.1 Hz, 2H), 3.55 (dd, *J* = 8.6, 6.4 Hz, 1H), 2.70 – 2.59 (m, 1H), 2.48 – 2.35 (m, 1H), 1.19 (t, *J* = 7.1 Hz, 3H) ppm. $^{13}C\{^1H\}$ NMR (151 MHz, $CDCl_3$): δ 173.2, 162.6 (d, *J* = 246.7 Hz), 145.6, 137.4, 135.4, 128.4 (d, *J* = 8.0 Hz), 117.0, 115.3 (d, *J* = 21.4 Hz), 115.1, 60.9, 50.5, 36.1, 14.3 ppm. $^{19}F\{^1H\}$ NMR (376 MHz, $CDCl_3$): δ -115.4 (s) ppm. HRMS (ESI-TOF) *m/z*: $[M + H]^+$ calcd for $C_{15}H_{18}FO_2$, 249.1291; found 249.1287.

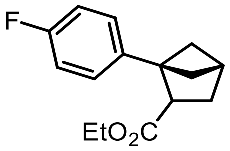

(±)-Ethyl 1-(4-fluorophenyl)bicyclo[2.1.1]hexane-2-carboxylate (4a)

General procedure C was used. The final product was purified by distillation (b.p. = 83-84 °C, 0.1 mmHg). Yield: 17.11 g, purity ~90%, 0.069 mol, 69%, colorless oil.

An analytically pure sample of the product was obtained by purification of the sample of the crude mixture by HPLC: Rt = 0-9 min, water/acetonitrile, 50-80%, flow 30 mL/min (loading pump 4 mL/min), column: Chomatorex 18 SMB100-5T, 100×19 mm, 5 µm. A mixture of isomers: ~9:1 (the sample contains ca. 10% of the isomeric 1,5-disubstituted bicyclo[2.1.1]hexane). ^1H NMR (500 MHz, $CDCl_3$): δ 7.41 – 7.08 (m, 2H), 7.03 – 6.91 (m, 2H), 4.16 – 3.87 (m, 2H), 3.03 – 2.93 (m, 1H), 2.63 – 2.50 (m, 1H), 2.20 – 2.04 (m, 3H), 1.83 – 1.76 (m, 2H), 1.62 (dd, *J* = 9.8, 6.7 Hz, 1H), 1.23 (t, *J* = 7.1 Hz), 0.97 (t, *J* = 7.1 Hz) 3H ppm. $^{13}C\{^1H\}$ NMR (126 MHz, $CDCl_3$): δ 175.2, 161.6 (d, *J* = 244.0 Hz), 138.0 (d, *J* = 3.0 Hz), 128.4 (d, *J* = 7.9 Hz), 127.6 (d, *J* = 7.9 Hz), 114.9 (d, *J* = 21.1 Hz), 114.9 (d, *J* = 21.2 Hz), 60.1, 57.9, 56.7, 53.3, 48.7, 46.6, 41.8, 39.9, 38.2, 35.2, 34.0, 30.2, 26.7, 14.4, 14.2 ppm. $^{19}F\{^1H\}$ NMR (376 MHz, $CDCl_3$): δ -117.2 (s), -117.3 (s) ppm. LCMS (M+H): 249. HRMS (ESI-TOF) *m/z*: $[M + H]^+$ calcd for $C_{15}H_{18}FO_2$, 249.1291; found 249.1283.

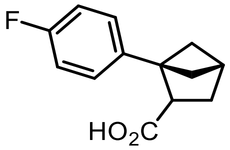

(±)-1-(4-Fluorophenyl)bicyclo[2.1.1]hexane-2-carboxylic acid (4b)

General procedure D was used. Yield: 8.80 g, 0.04 mol, 69%, white solid, m.p. = 117-118 °C. ^1H NMR (500 MHz, DMSO-d_6): δ 11.85 (s, 1H), 7.20 (t, *J* = 6.9 Hz, 2H), 7.09 (t, *J* = 8.8 Hz, 2H), 2.97 (dd, *J* = 8.8, 3.9 Hz, 1H), 2.45 (s, 1H), 2.12 (t, *J* = 9.7 Hz, 1H), 2.04 (dd, *J* = 9.4, 6.8 Hz, 1H), 1.90 (d, *J* = 10.5 Hz, 1H), 1.76 (d, *J* = 5.6 Hz, 1H), 1.70 – 1.63 (m, 1H), 1.60 (dd, *J* = 9.3, 6.7 Hz, 1H) ppm. $^{13}C\{^1H\}$ NMR (151 MHz, DMSO-d_6): δ 175.9, 160.7 (d, *J* = 241.7 Hz), 138.3 (d, *J* = 2.9 Hz),

127.8 (d, *J* = 8.0 Hz), 114.6 (d, *J* = 21.0 Hz), 56.8, 47.2, 46.3, 37.7, 34.4, 33.8 ppm. $^{19}F\{^1H\}$ NMR (376 MHz, DMSO-d$_6$): δ -117.4 (s) ppm. LCMS (M-H): 219. HRMS (ESI-TOF) *m/z*: $[M - H]^-$ calcd for $C_{13}H_{12}FO_2$, 219.0821; found 219.0819.

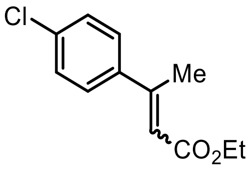

Ethyl-3-(4-chlorophenyl)but-2-enoate

General procedure A was used. The final product was purified by distillation (b.p. = 83-84 °C, 0.1 mmHg). Yield: 0.32 mol, 71.68 g, 80%, colorless oil. A mixture of *cis*+*trans*-isomers: ~4:1. ^1H NMR (500 MHz, $CDCl_3$): δ 7.40 (d, *J* = 8.5 Hz), 7.31 (d, *J* = 8.7 Hz) 2H, 7.34 (d, *J* = 8.6 Hz), 7.14 (d, *J* = 8.4 Hz) 2H, 6.11 (s), 5.91 (s) 1H, 4.21 (q, *J* = 7.1 Hz), 4.01 (q, *J* = 7.1 Hz) 2H, 2.54 (s), 2.15 (s) 3H, 1.31 (t, *J* = 7.1 Hz), 1.12 (t, *J* = 7.1 Hz) 3H ppm. $^{13}C\{^1H\}$ NMR (101 MHz, $CDCl_3$): δ 166.8, 165.8, 154.3, 154.1, 140.7, 139.3, 135.1, 133.8, 128.8, 128.5, 128.3, 127.7, 118.4, 117.7, 60.1, 60.0, 27.2, 17.9, 14.5, 14.1 ppm. LCMS (M+H): 225. HRMS (ESI-TOF) *m/z*: $[M + H]^+$ calcd for $C_{12}H_{14}ClO_2$, 225.0682; found 225.0674.

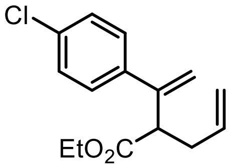

Ethyl 2-(1-(4-chlorophenyl)vinyl)pent-4-enoate (5)

General procedure B was used. Yield: 38.54 g, purity ~90%, 0.146 mol, 73%, colorless oil.

An analytically pure sample of the product was obtained by purification of the sample of the crude mixture by HPLC: Rt = 0-5 min, water/acetonitrile, 50-80%, flow 30 mL/min (loading pump 4 mL/min), column: Chomatorex 18 SMB100-5T, 100×19 mm, 5 µm. ^1H NMR (500 MHz, $CDCl_3$): δ 7.34 – 7.29 (m, 2H), 7.12 – 7.06 (m, 2H), 5.78 – 5.65 (m, 1H), 5.02 – 4.91 (m, 2H), 4.25 (q, *J* = 7.1 Hz, 2H), 2.88 (d, *J* = 5.4 Hz, 2H), 2.22 (s, 3H), 1.32 (t, *J* = 7.1 Hz, 3H) ppm. $^{13}C\{^1H\}$ NMR (126 MHz, $CDCl_3$): δ 169.2, 144.5, 141.3, 135.9, 133.3, 128.67, 128.65, 115.9, 60.6, 35.5, 23.3, 14.4 ppm. HRMS (ESI-TOF) *m/z*: $[M + H]^+$ calcd for $C_{15}H_{18}ClO_2$, 265.0995; found 265.0988.

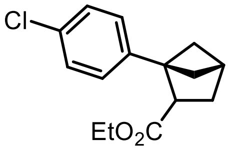

(±)-Ethyl 1-(4-chlorophenyl)bicyclo[2.1.1]hexane-2-carboxylate (5a)

General procedure C was used. The final product was purified by distillation (b.p. = 102-103 °C, 0.1 mmHg). Yield: 19.30 g, purity ~90%, 0.073 mol, 73%, colorless oil.

An analytically pure sample of the product was obtained by purification of the sample of the crude mixture by HPLC: Rt = 0-5 min, water/acetonitrile, 50-80%, flow 30 mL/min (loading pump 4 mL/min), column: XBridge BEH C18, OBD 30×100, 5 µm. ^{1}H NMR (500 MHz, DMSO-d_6): δ 7.33 (d, *J* = 8.3 Hz, 2H), 7.16 (d, *J* = 8.3 Hz, 2H), 3.92 – 3.82 (m, 2H), 3.05 (dd, *J* = 8.8, 4.0 Hz, 1H), 2.48 (s, 1H), 2.12 (t, *J* = 9.8 Hz, 1H), 1.98 (dd, *J* = 9.5, 6.8 Hz, 1H), 1.93 (d, *J* = 10.8 Hz, 1H), 1.77 (d, *J* = 5.3 Hz, 1H), 1.70 – 1.62 (m, 2H), 0.89 (t, *J* = 7.1 Hz, 3H) ppm. ^{13}C{^{1}H} NMR (151 MHz, $CDCl_3$): δ 175.0, 136.5 (d, *J* = 1312.2 Hz), 128.2, 127.5, 60.1, 57.9, 48.7, 46.6, 38.1, 35.3, 34.1, 14.2 ppm. LCMS (M+H): 265. HRMS (ESI-TOF) *m/z*: $[M + H]^+$ calcd for $C_{15}H_{18}ClO_2$, 265.0995; found 265.1003.

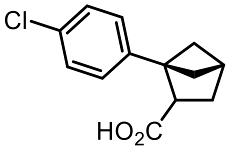

(±)-1-(4-Chlorophenyl)bicyclo[2.1.1]hexane-2-carboxylic acid (5b)

General procedure D was used. Yield: 10.62 g, 0.045 mol, 75%, white solid, m.p. = 124-125 °C. ^{1}H NMR (500 MHz, DMSO-d_6): δ 11.89 (s, 1H), 7.33 (d, *J* = 8.4 Hz, 2H), 7.19 (d, *J* = 8.4 Hz, 2H), 3.02 – 2.95 (m, 1H), 2.46 (s, 1H), 2.13 (t, *J* = 9.9 Hz, 1H), 2.02 (dd, *J* = 9.6, 6.7 Hz, 1H), 1.91 (d, *J* = 10.7 Hz, 1H), 1.79 – 1.74 (m, 1H), 1.69 – 1.64 (m, 1H), 1.60 (dd, *J* = 9.6, 6.5 Hz, 1H) ppm. ^{13}C{^{1}H} NMR (126 MHz, DMSO-d_6): δ 175.8, 141.2, 130.7, 127.9, 127.9, 56.8, 47.2, 46.2, 37.6, 34.5, 33.8 ppm. LCMS (M-H): 235. HRMS (ESI-TOF) *m/z*: $[M - H]^-$ calcd for $C_{13}H_{12}ClO_2$, 235.0526; found 235.0526.

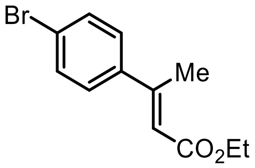

Ethyl-3-(4-bromophenyl)but-2-enoate

General procedure A was used. The final product was purified by distillation (b.p. = 94-95 °C, 0.1 mmHg). Yield: 80.70 g, 0.30 mol, 76%, yellow oil.^{1}H NMR (500 MHz, $CDCl_3$): δ 7.49 (d, *J* = 8.5 Hz, 2H), 7.34 (d, *J* = 8.5 Hz, 2H), 6.11 (s, 1H), 4.21 (q, *J* = 7.1 Hz, 2H), 2.54 (s, 3H), 1.31 (t, *J* = 7.1 Hz, 3H) ppm. ^{13}C{^{1}H} NMR (101 MHz, $CDCl_3$): δ 166.8, 154.2, 141.2, 131.8, 128.0, 123.3, 117.7, 60.1, 17.9, 14.5 ppm. LCMS (M+H): 269. HRMS (ESI-TOF) *m/z*: $[M + H]^+$ calcd for $C_{12}H_{14}BrO_2$, 271.0157; found 271.0148.

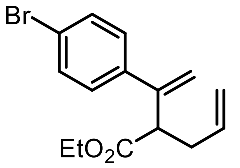

Ethyl 2-(1-(4-bromophenyl)vinyl)pent-4-enoate (6)

General procedure B was used. Yield: 49.44 g, purity 90%, 0.16 mol, 80%, yellow oil.

An analytically pure sample of the product was obtained by purification of the sample of the crude mixture by HPLC: Rt = 0-5 min, water/acetonitrile, 50-90%, flow 60 mL/min (loading pump 4 mL/min), column: XBridge OBD 30×100, 5 µm. ^{1}H NMR (500 MHz, $CDCl_3$): δ 7.49 – 7.40 (m, 2H), 7.30 – 7.21 (m, 2H), 5.80 – 5.69 (m, 1H), 5.40 (s, 1H), 5.30 (s, 1H), 5.06 (dq, *J* = 17.1, 1.6 Hz, 1H), 5.03 – 4.98 (m, 1H), 4.13 (q, *J* = 7.1 Hz, 2H), 3.54 (dd, *J* = 8.7, 6.3 Hz, 1H), 2.69 – 2.59 (m, 1H), 2.45 – 2.37 (m, 1H), 1.19 (t, *J* = 7.1 Hz, 3H) ppm. ^{13}C{^{1}H} NMR (126 MHz, $CDCl_3$): δ 173.1, 145.5, 140.3, 135.3, 131.6, 128.4, 121.9, 117.1, 115.7, 61.0, 50.2, 36.1, 14.3 ppm. LCMS (M+H): 311. HRMS (ESI-TOF) *m/z*: $[M + H]^+$ calcd for $C_{15}H_{18}BrO_2$, 311.0470; found 311.0464.

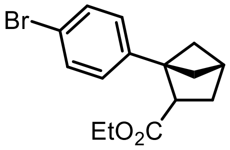

(±)-Ethyl 1-(4-bromophenyl)bicyclo[2.1.1]hexane-2-carboxylate (6a)

General procedure C was used. The final product was purified by distillation (b.p. = 112-113 °C, 0.1 mmHg). Yield: 20.70 g, purity 90%, 0.067 mol, 67%, yellow oil.

An analytically pure sample of the product was obtained by purification of the sample of the crude mixture by HPLC: Rt = 0-5 min, water/acetonitrile, 50-100%, flow 60 mL/min (loading pump 4 mL/min), column: XBridge OBD 30×100, 5 µm. ^{1}H NMR (500 MHz, $CDCl_3$): δ 7.39 (d, *J* = 8.4 Hz, 2H), 7.03 (d, *J* = 8.4 Hz, 2H), 3.93 (q, *J* = 7.1 Hz, 2H), 3.00 – 2.91 (m, 1H), 2.53 (s, 1H), 2.22 – 2.04 (m, 3H), 1.81 – 1.72 (m, 2H), 1.62 (dd, *J* = 9.8, 6.7 Hz, 1H), 0.99 (t, *J* = 7.1 Hz, 3H) ppm. ^{13}C{^{1}H} NMR (126 MHz, $CDCl_3$): δ 175.0, 141.3, 131.2, 127.9, 120.2, 60.1, 57.9, 48.6, 46.5, 38.1, 35.3, 34.1, 14.2 ppm. LCMS (M+H): 309. HRMS (ESI-TOF) *m/z*: $[M + H]^+$ calcd for $C_{15}H_{18}BrO_2$, 311.0470; found 311.0465.

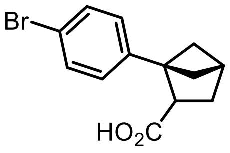

(±)-1-(4-Bromophenyl)bicyclo[2.1.1]hexane-2-carboxylic acid (6b)

General procedure D was used. Yield: 11.76 g, 0.042 mol, 70%, white solid, m.p. = 132-133 °C. ^{1}H NMR (500 MHz, DMSO-d_6): δ 11.90 (s, 1H), 7.46 (d, *J* = 8.3 Hz, 2H), 7.13 (d, *J* = 8.4 Hz, 2H),

2.98 (dd, J = 8.6, 3.7 Hz, 1H), 2.45 (s, 1H), 2.12 (t, J = 9.8 Hz, 1H), 2.01 (dd, J = 9.5, 6.7 Hz, 1H), 1.90 (d, J = 10.7 Hz, 1H), 1.78 – 1.74 (m, 1H), 1.69 – 1.65 (m, 1H), 1.60 (dd, J = 9.6, 6.5 Hz, 1H) ppm. $^{13}C\{^1H\}$ NMR (101 MHz, DMSO-d_6): δ 175.8, 141.6, 130.8, 128.3, 56.8, 47.2, 46.2, 37.5, 34.5, 33.8 ppm. LCMS (M+H): 281. HRMS (ESI-TOF) *m/z*: $[M - H]^-$ calcd for $C_{13}H_{12}BrO_2$, 279.0021; found 279.0017.

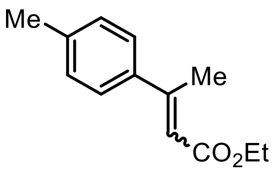

Ethyl-3-(*p*-tolyl)but-2-enoate

General procedure A was used. The final product was purified by distillation (b.p. = 72-73 °C, 0.1 mmHg). Yield: 64.46 g, 0.316 mol, 79%, colorless oil. A mixture of *cis*+*trans*-isomers: ~4:1. ^1H NMR (500 MHz, $CDCl_3$): δ 7.39 (d, J = 8.1 Hz), 7.16 (d, J = 8.1 Hz) 2H, 7.18 (d, J = 8.0 Hz), 7.12 (d, J = 8.0 Hz) 2H, 6.14 (s), 5.89 (s) 1H, 4.21 (q, J = 7.1 Hz), 4.02 (q, J = 7.1 Hz) 2H, 2.57 (s, 2H), 2.37 (s), 2.17 (s) 3H, 1.32 (t, J = 7.1 Hz), 1.12 (t, J = 7.1 Hz) 3H ppm. $^{13}C\{^1H\}$ NMR (126 MHz, $CDCl_3$): δ 167.1, 166.1, 155.7, 155.5, 139.4, 139.2, 137.9, 137.7, 129.3, 128.7, 127.0, 126.3, 117.5, 116.4, 59.9, 59.8, 27.3, 21.4, 21.3, 17.9, 14.5, 14.2 ppm. LCMS (M+H): 205. HRMS (ESI-TOF) *m/z*: $[M + H]^+$ calcd $C_{13}H_{17}O_2$, 205.1229; found 205.1229.

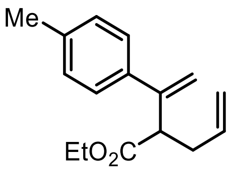

Ethyl 2-(1-(*p*-tolyl)vinyl)pent-4-enoate (7)

General procedure B was used. Yield: 39.04 g, purity 90%, 0.16 mol, 90%, colorless oil.

An analytically pure sample of the product was obtained by purification of the sample of the crude mixture by HPLC: Rt = 0-6 min, water/acetonitrile, 50-85%, flow 30 mL/min (loading pump 4 mL/min), column: Chomatorex 18 SMB100-5T, 100×19 mm, 5 μm. ^1H NMR (500 MHz, $CDCl_3$): δ 7.30 (d, J = 8.0 Hz, 2H), 7.14 (d, J = 7.9 Hz, 2H), 5.86 – 5.72 (m, 1H), 5.39 (s, 1H), 5.24 (s, 1H), 5.07 (d, J = 17.1 Hz, 1H), 5.01 (d, J = 10.2 Hz, 1H), 4.14 (q, J = 7.1 Hz, 2H), 3.60 (dd, J = 8.9, 6.1 Hz, 1H), 2.73 – 2.58 (m, 1H), 2.50 – 2.38 (m, 1H), 2.35 (s, 3H), 1.20 (t, J = 7.1 Hz, 3H) ppm. $^{13}C\{^1H\}$ NMR (126 MHz, $CDCl_3$): δ 173.5, 146.3, 138.4, 137.6, 135.7, 129.1, 126.5, 116.8, 114.2, 60.8, 50.3, 36.3, 21.2, 14.3 ppm. HRMS (ESI-TOF) *m/z*: $[M + H]^+$ calcd for $C_{16}H_{21}O_2$, 245.1542; found 245.1532.

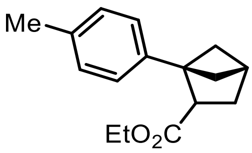

(±)-Ethyl 1-(*p*-tolyl)bicyclo[2.1.1]hexane-2-carboxylate (7a)

General procedure C was used. Yield: 17.57 g, purity 90%, 0.072 mol, 72%, colorless oil. The final product was purified by distillation (b.p. = 98-99 °C, 0.1 mmHg).

An analytically pure sample of the product was obtained by purification of the sample of the crude mixture by HPLC: Rt = 0-5 min, water/acetonitrile, 40-90%, flow 60 mL/min (loading pump 4 mL/min), column: XBridge OBD 30×100, 5 µm. ^{1}H NMR (500 MHz, $CDCl_3$): δ 7.10 – 7.05 (m, 4H), 3.99 – 3.89 (m, 2H), 3.00 – 2.94 (m, 1H), 2.52 (s, 1H), 2.31 (s, 3H), 2.19 – 2.07 (m, 3H), 1.81 – 1.74 (m, 2H), 1.62 (dd, *J* = 9.8, 6.7 Hz, 1H), 0.98 (t, *J* = 7.1 Hz, 3H) ppm. ^{13}C{^{1}H} NMR (126 MHz, $CDCl_3$): δ 175.4, 139.2, 135.9, 128.8, 125.9, 60.0, 58.3, 48.6, 46.6, 38.1, 35.3, 34.2, 21.2, 14.2 ppm. LCMS (M+H): 245. HRMS (ESI-TOF) *m/z*: $[M + H]^+$ calcd for $C_{16}H_{21}O_2$, 245.1542; found 245.1531.

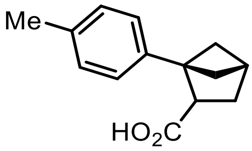

(±)-1-(*p*-Tolyl)bicyclo[2.1.1]hexane-2-carboxylic acid (7b)

General procedure D was used. Yield: 9.07 g, 0.042 mol, 76%, white solid, m.p. = 122-123 °C. ^{1}H NMR (500 MHz, DMSO-d_6): δ 11.81 (s, 1H), 7.07 (s, 4H), 2.94 (dd, *J* = 8.6, 3.8 Hz, 1H), 2.44 (s, 1H), 2.25 (s, 3H), 2.16 – 2.01 (m, 2H), 1.89 (d, *J* = 10.6 Hz, 1H), 1.74 – 1.69 (m, 1H), 1.66 – 1.60 (m, 1H), 1.57 (dd, *J* = 9.6, 6.5 Hz, 1H) ppm. ^{13}C{^{1}H} NMR (126 MHz, DMSO-d_6): δ 176.1, 139.0, 135.0, 128.5, 125.8, 57.3, 47.1, 46.4, 37.6, 34.5, 33.9, 20.7 ppm. LCMS (M-H): 215. HRMS (ESI-TOF) *m/z*: $[M + H]^+$ calcd for $C_{14}H_{17}O_2$, 217.1229; found 217.1227.

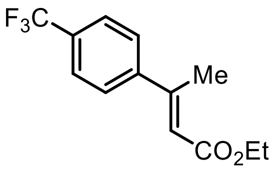

Ethyl-3-(4-(trifluoromethyl)phenyl)but-2-enoate

General procedure A was used. The final product was purified by distillation (b.p. = 63-64 °C, 0.1 mmHg). Yield: 87.72 g, 0.34 mol, 85%, yellow oil. ^{1}H NMR (500 MHz, $CDCl_3$): δ 7.63 (d, *J* = 8.2 Hz, 2H), 7.56 (d, *J* = 8.2 Hz, 2H), 6.14 (s, 1H), 4.23 (q, *J* = 7.1 Hz, 2H), 2.57 (s, 3H), 1.32 (t, *J* = 7.1 Hz, 3H) ppm. ^{13}C{^{1}H} NMR (126 MHz, $CDCl_3$): δ 166.6, 153.9, 146.0, 130.9 (dd, *J* = 65.2, 32.6 Hz), 126.8, 125.6 (q, *J* = 3.7 Hz), 124.1 (q, *J* = 272.2 Hz), 119.1, 60.3, 18.1, 14.4 ppm.

$^{19}F\{^1H\}$ NMR (376 MHz, $CDCl_3$): δ -63.2 (s) ppm. HRMS (ESI-TOF) *m/z*: $[M + H]^+$ calcd for $C_{13}H_{14}F_3O_2$, 259.0946; found 259.0939.

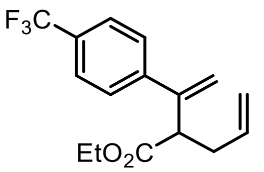

Ethyl 2-(1-(4-(trifluoromethyl)phenyl)vinyl)pent-4-enoate (8)

General procedure B was used. Yield: 48.87 g, purity 90%, 0.164 mol, 82%, yellow oil. An analytically pure sample of the product was obtained by purification of the sample of the crude mixture by column chromatography, SiO_2, hexane/MeO*t*Bu, 9:1. ^1H NMR (500 MHz, $CDCl_3$): δ 7.59 (d, *J* = 8.2 Hz, 2H), 7.50 (d, *J* = 8.1 Hz, 2H), 5.83 – 5.69 (m, 1H), 5.47 (s, 1H), 5.39 (s, 1H), 5.07 (dd, *J* = 17.1, 1.3 Hz, 1H), 5.03 (d, *J* = 10.2 Hz, 1H), 4.14 (q, *J* = 7.1 Hz, 2H), 3.59 (dd, *J* = 8.4, 6.6 Hz, 1H), 2.73 – 2.61 (m, 1H), 2.48 – 2.37 (m, 1H), 1.19 (t, *J* = 7.1 Hz, 3H) ppm. $^{13}C\{^1H\}$ NMR (151 MHz, $CDCl_3$): δ 173.0, 145.5, 145.0, 135.2, 129.9 (q, *J* = 32.5 Hz), 127.1, 125.4 (q, *J* = 3.7 Hz), 124.3 (q, *J* = 272.0 Hz), 117.3, 117.0, 61.0, 50.3, 36.1, 14.2 ppm. $^{19}F\{^1H\}$ NMR (376 MHz, $CDCl_3$): δ -63.1 (s) ppm. LCMS (M+H): 299. HRMS (ESI-TOF) *m/z*: $[M + H]^+$ calcd for $C_{16}H_{18}F_3O_2$, 299.1259; found 299.1253.

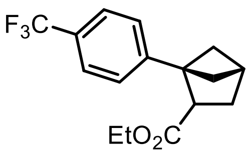

(±)-Ethyl 1-(4-(trifluoromethyl)phenyl)bicyclo[2.1.1]hexane-2-carboxylate (8a)

General procedure C was used. The final product was purified by distillation (b.p. = 94-95 °C, 0.1 mmHg). Yield: 21.46 g, purity ~90%, 0.072 mol, 72%, colorless oil.

An analytically pure sample of the product was obtained by purification of the sample of the crude mixture by HPLC: Rt = 0-7 min, water/acetonitrile, 50-80%, flow 30 mL/min (loading pump 4 mL/min), column: Chomatorex 18 SMB100-5T, 100×19 mm, 5 μm. A mixture of isomers: ~9: (the sample contains ca. 10% of the isomeric 1,5-disubstituted bicyclo[2.1.1]hexane). ^1H NMR (500 MHz, $CDCl_3$): δ 7.52 (d, *J* = 8.1 Hz, 1H), 7.27 (d, *J* = 8.4 Hz, 1H), 4.17 – 4.07 (m), 3.96 – 3.86 (m) 2H, 3.01 (dd, *J* = 8.6, 4.1 Hz, 1H), 2.56 (s, 1H), 2.24 – 2.05 (m, 3H), 1.87 – 1.77 (m, 2H), 1.67 (dd, *J* = 9.7, 6.7 Hz, 1H), 1.23 (t, *J* = 7.1 Hz), 0.94 (t, *J* = 7.1 Hz) 3H ppm. $^{13}C\{^1H\}$ NMR (126 MHz, $CDCl_3$): δ 174.8, 171.0, 146.5, 128.7 (q, *J* = 32.3 Hz), 127.2, 126.5, 125.1 (q, *J* = 3.8 Hz), 124.5 (q, *J* = 271.8 Hz), 60.2, 58.0, 56.9, 53.2, 48.8, 46.6, 41.7, 40.1, 38.1, 35.5, 34.0, 30.3, 26.7, 14.4, 14.1 ppm. $^{19}F\{^1H\}$ NMR (376 MHz, $CDCl_3$): δ -62.9 (s) ppm. HRMS (ESI-TOF) *m/z*: $[M + H]^+$ calcd for $C_{16}H_{18}F_3O_2$, 299.1259; found 299.1249.

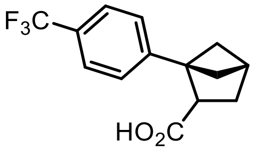

(±)-1-(4-(Trifluoromethyl)phenyl)bicyclo[2.1.1]hexane-2-carboxylic acid (8b)

General procedure D was used. Yield: 10.26 g, 0.038 mol, 69%, white solid, m.p. = 106-107 °C. ^{1}H NMR (500 MHz, DMSO-d$_6$): δ 11.93 (s, 1H), 7.64 (d, *J* = 8.1 Hz, 2H), 7.40 (d, *J* = 8.0 Hz, 2H), 3.06 (dd, *J* = 8.7, 3.8 Hz, 1H), 2.16 (t, *J* = 9.9 Hz, 1H), 2.04 (dd, *J* = 9.5, 6.7 Hz, 1H), 1.93 (d, *J* = 10.7 Hz, 1H), 1.86 – 1.79 (m, 1H), 1.74 – 1.69 (m, 1H), 1.66 (dd, *J* = 9.5, 6.5 Hz, 1H) ppm. ^{13}C{^{1}H} NMR (151 MHz, DMSO-d$_6$): δ 175.6, 147.0, 127.0 (d, *J* = 33.0 Hz), 126.8, 124.8 (q, *J* = 3.8 Hz), 124.4 (q, *J* = 271.9 Hz), 56.9, 47.3, 46.2, 37.6, 34.7, 33.7 ppm. ^{19}F{^{1}H} NMR (376 MHz, DMSO-d$_6$): δ -61.2 (s) ppm. LCMS (M-H): 269. HRMS (ESI-TOF) *m/z*: [M - H]$^-$ calcd for $C_{14}H_{12}F_3O_2$, 269,0789; found 269.0784.

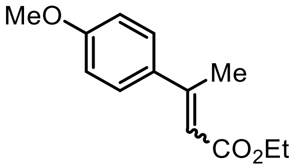

Ethyl-3-(4-methoxyphenyl)but-2-enoate

General procedure A was used. The final product was purified by distillation (b.p. = 94-95 °C, 0.1 mmHg). Yield: 78.32 g, 0.356 mol, 89%, colorless oil. A mixture of *cis*+*trans*-isomers: ~4:1. ^{1}H NMR (500 MHz, CDCl$_3$): δ 7.45 (d, *J* = 8.8 Hz), 7.19 (d, *J* = 8.7 Hz) 2H, 6.89 (d, *J* = 8.8 Hz, 1H), 6.87 (d, *J* = 8.4 Hz) 2H, 6.11 (s), 5.87 (s) 1H, 4.21 (q, *J* = 7.1 Hz), 4.03 (q, *J* = 7.1 Hz) 2H, 3.83 (s), 3.81 (s) 3H, 2.56 (s), 2.16 (s) 3H, 1.31 (t, *J* = 7.1 Hz), 1.14 (t, *J* = 7.1 Hz) 3H ppm. ^{13}C{^{1}H} NMR (151 MHz, CDCl$_3$): δ 167.2, 166.3, 160.6, 159.5, 155.00, 154.98, 134.5, 132.8, 128.7, 127.8, 117.2, 115.5, 114.0, 113.4, 59.8, 55.5, 55.3, 31.0, 27.2, 19.4, 17.8, 14.5, 14.2 ppm. LCMS (M+H): 221. HRMS (ESI-TOF) *m/z*: [M + H]$^+$ calcd for $C_{13}H_{17}O_3$, 221.1178; found 221.1171.

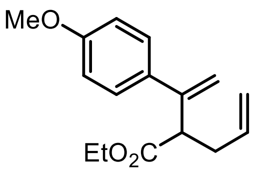

Ethyl 2-(1-(4-methoxyphenyl)vinyl)pent-4-enoate (9)

General procedure B was used. Yield: 43.16 g, purity ~90%, 0.166 mol, 83%, colorless oil. An analytically pure sample of the product was obtained by purification of the sample of the crude mixture by column chromatography, SiO_2, hexane/MeO*t*Bu, 9:1. ^{1}H NMR (500 MHz, CDCl$_3$): δ

7.34 (d, J = 8.7 Hz, 2H), 6.86 (d, J = 8.7 Hz, 2H), 5.84 – 5.73 (m, 1H), 5.35 (s, 1H), 5.20 (s, 1H), 5.07 (dd, J = 17.1, 1.4 Hz, 1H), 5.01 (d, J = 10.1 Hz, 1H), 4.14 (q, J = 7.1 Hz, 2H), 3.81 (s, 3H), 3.58 (dd, J = 8.9, 6.1 Hz, 1H), 2.74 – 2.60 (m, 1H), 2.47 – 2.37 (m, 1H), 1.20 (t, J = 7.1 Hz, 3H) ppm. $^{13}C\{^1H\}$ NMR (151 MHz, $CDCl_3$): δ 173.4, 159.4, 145.9, 135.7, 133.7, 127.8, 116.8, 113.8, 113.6, 60.8, 55.4, 50.4, 36.3, 14.3 ppm. LCMS (M+H): 261. HRMS (ESI-TOF) *m/z*: $[M + H]^+$ calcd for $C_{16}H_{21}O_3$, 261.1491; found 261.1482.

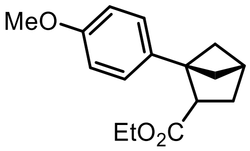

(±)-Ethyl 1-(4-methoxyphenyl)bicyclo[2.1.1]hexane-2-carboxylate (9a)

General procedure C was used. The final product was purified by distillation (b.p. = 110-111 °C, 0.1 mmHg). Yield: 17.68 g, purity 90%, 0.068 mol, 68%, colorless oil.

An analytically pure sample of the product was obtained by purification of the sample of the crude mixture by HPLC: Rt = 0-7 min, water/MeOH, 40-90%, flow 30 mL/min (loading pump 4 mL/min), column: Chomatorex 18 SMB100-5T, 100×19 mm, 5 μm. A mixture of isomers: 9:1 (the sample contains ca. 10% of the isomeric 1,5-disubstituted bicyclo[2.1.1]hexane). ^1H NMR (500 MHz, $CDCl_3$): δ 7.32 (d, J = 8.7 Hz), 7.09 (d, J = 8.7 Hz) 2H, 6.86 (d, J = 8.7 Hz), 6.82 (d, J = 8.6 Hz) 2H, 4.16 – 3.89 (m, 2H), 3.79 (s), 3.78 (s) 3H, 3.02 – 2.87 (m, 1H), 2.58 (s), 2.51 (s) 1H, 2.21 – 2.02 (m, 3H), 1.86 – 1.71 (m, 2H), 1.65 – 1.49 (m, 1H), 1.23 (t, J = 7.1 Hz), 0.99 (t, J = 7.1 Hz) 3H ppm. $^{13}C\{^1H\}$ NMR (126 MHz, $CDCl_3$): δ 175.4, 158.2, 134.4, 127.9, 127.1, 113.6, 113.5, 60.0, 59.9, 58.1, 56.8, 55.4, 53.3, 48.6, 46.6, 41.8, 39.8, 38.2, 35.2, 34.1, 30.0, 26.7, 14.4, 14.2 ppm. LCMS (M+H): 261. HRMS (ESI-TOF) *m/z*: $[M + H]^+$ calcd for $C_{16}H_{21}O_3$, 261.1491; found 261.1475.

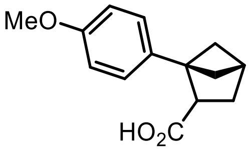

(±)-1-(4-Methoxyphenyl)bicyclo[2.1.1]hexane-2-carboxylic acid (9b)

General procedure D was used. Yield: 8.58 g, 0.037 mol, 67%, white solid, m.p. = 156-157 °C. ^1H NMR (500 MHz, DMSO-d_6): δ 11.80 (s, 1H), 7.09 (d, J = 8.5 Hz, 2H), 6.83 (d, J = 8.5 Hz, 2H), 3.71 (s, 3H), 2.93 (d, J = 4.8 Hz, 1H), 2.43 (br s, 1H), 2.15 – 2.00 (m, 2H), 1.89 (d, J = 10.4 Hz, 1H), 1.73 (br s, 1H), 1.62 (br s, 1H), 1.59 – 1.50 (m, 1H) ppm. $^{13}C\{^1H\}$ NMR (126 MHz, DMSO-d_6): δ 176.2, 158.6, 157.6, 134.0, 127.0, 113.3, 57.1, 55.0, 47.1, 46.4, 37.7, 34.4, 33.9 ppm. LCMS (M-H): 231. HRMS (ESI-TOF) *m/z*: $[M - H]^-$ calcd for $C_{14}H_{15}O_3$, 231.1021; found 231.1015.

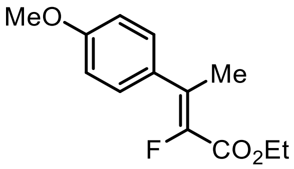

Ethyl-2-fluoro-3-(4-methoxyphenyl)but-2-enoate

General procedure A was used with $(EtO)_2(O)P\text{-}CHF(CO_2Et)$. The final product was purified by distillation (b.p. = 92-93 °C, 0.1 mmHg). Yield: 76.16 g, 0.32 mol, 80%, colorless oil. ^{1}H NMR (500 MHz, $CDCl_3$): δ 7.12 (d, *J* = 8.6 Hz, 2H), 6.88 (d, *J* = 8.6 Hz, 2H), 4.08 (q, *J* = 7.1 Hz, 2H), 3.82 (s, 3H), 2.13 (d, *J* = 4.5 Hz, 3H), 1.10 (t, *J* = 7.1 Hz, 3H) ppm. ^{13}C{^{1}H} NMR (126 MHz, $CDCl_3$): δ 160.9 (d, *J* = 35.7 Hz), 159.4, 144.3 (d, *J* = 251.4 Hz), 131.4 (d, *J* = 17.1 Hz), 130.6 (d, *J* = 5.6 Hz), 129.0 (d, *J* = 2.8 Hz), 113.6, 61.1, 55.4, 19.5 (d, *J* = 6.6 Hz), 13.9 ppm. ^{19}F{^{1}H} NMR (376 MHz, $CDCl_3$): δ -124.2 (s) ppm. HRMS (ESI-TOF) *m/z*: $[M + H]^+$ calcd for $C_{13}H_{16}FO_3$, 239.1083; found 239.1078.

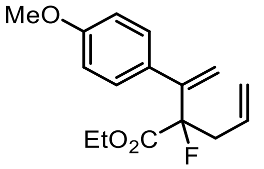

Ethyl 2-fluoro-2-(1-(4-methoxyphenyl)vinyl)pent-4-enoate (10)

General procedure B was used. Yield: 43.37 g, purity ~90%, 0.156 mol, 78%, colorless oil.

An analytically pure sample of the product was obtained by purification of the sample of the crude mixture by column chromatography, SiO_2, MeO*t*Bu/hexane, 9:1. ^{1}H NMR (500 MHz, $CDCl_3$): δ 7.26 (d, *J* = 8.4 Hz, 2H), 6.84 (d, *J* = 8.6 Hz, 2H), 5.89 – 5.76 (m, 1H), 5.54 (d, *J* = 2.1 Hz, 1H), 5.38 (s, 1H), 5.17 (s, 1H), 5.14 (d, *J* = 5.7 Hz, 1H), 4.24 – 4.12 (m, 2H), 3.80 (s, 3H), 2.87 (t, *J* = 6.0 Hz, 1H), 2.83 (d, *J* = 7.0 Hz, 1H), 1.19 (t, *J* = 7.1 Hz, 3H) ppm. ^{13}C{^{1}H} NMR (126 MHz, $CDCl_3$): δ 169.7 (d, *J* = 26.6 Hz), 159.5, 146.0 (d, *J* = 20.0 Hz), 130.9, 129.7 (d, *J* = 1.4 Hz), 119.9, 117.6 (d, *J* = 8.5 Hz), 113.6, 96.9 (d, *J* = 188.9 Hz), 61.9, 55.4, 40.6 (d, *J* = 22.4 Hz), 14.2 ppm. ^{19}F{^{1}H} NMR (376 MHz, $CDCl_3$): δ -155.7 (s) ppm. HRMS (ESI-TOF) *m/z*: $[M + H]^+$ calcd for $C_{16}H_{20}FO_3$, 279.1396; found 279.1392.

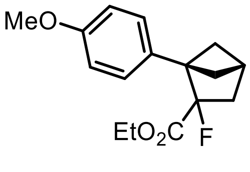

(±)-Ethyl 2-fluoro-1-(4-methoxyphenyl)bicyclo[2.1.1]hexane-2-carboxylate (10a)

General procedure C was used. The final product was purified by distillation (b.p. = 106-107 °C, 0.1 mmHg). Yield: 19.74 g, purity 90%, 0.071 mol, 71%, colorless oil.

An analytically pure sample of the product was obtained by purification of the sample of the crude mixture by HPLC: Rt = 0-7 min, water/MeOH, 40-90%, flow 30 mL/min (loading pump 4 mL/min), column: Chomatorex 18 SMB100-5T, 100×19 mm, 5 μm. ^{1}H NMR (500 MHz, $CDCl_3$): δ 7.10 (d, *J* = 8.6 Hz, 2H), 6.81 (d, *J* = 8.6 Hz, 2H), 4.13 – 4.01 (m, 2H), 3.78 (s, 3H), 2.68 – 2.59 (m, 1H), 2.54 (s, 1H), 2.41 – 2.35 (m, 1H), 2.28 – 2.22 (m, 1H), 2.15 (ddd, *J* = 27.0, 12.2, 3.7 Hz, 1H), 1.99 – 1.88 (m, 2H), 1.06 (t, *J* = 7.1 Hz, 3H) ppm. ^{13}C{^{1}H} NMR (126 MHz, $CDCl_3$): δ 171.4 (d, *J* = 28.9 Hz), 158.8, 130.8, 127.8, 113.6, 101.2 (d, *J* = 203.9 Hz), 62.6 (d, *J* = 21.7 Hz), 61.4, 55.4, 43.2 (d, *J* = 5.0 Hz), 42.8, 42.6 (d, *J* = 3.4 Hz), 33.2, 14.1 ppm. ^{19}F{^{1}H} NMR (376 MHz, $CDCl_3$): δ -159.6 (s) ppm. GCMS (M): 278. HRMS (ESI-TOF) *m/z*: $[M + H]^+$ calcd for $C_{16}H_{20}FO_3$, 279.1396; found 279.1384.

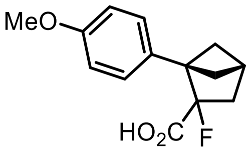

(±)-2-Fluoro-1-(4-methoxyphenyl)bicyclo[2.1.1]hexane-2-carboxylic acid (10b)

General procedure D was used. Yield: 10.00 g, 0.040 mol, 75%, beige solid, m.p. = 139-140 °C. ^{1}H NMR (500 MHz, DMSO-d_6): δ 13.03 (br s, 1H), 7.07 (d, *J* = 8.4 Hz, 2H), 6.85 (d, *J* = 8.5 Hz, 2H), 3.72 (s, 3H), 2.50 – 2.41 (m, 2H), 2.37 – 2.28 (m, 1H), 2.14 – 1.98 (m, 2H), 1.97 – 1.78 (m, 2H) ppm. ^{13}C{^{1}H} NMR (126 MHz, DMSO-d_6): δ 172.3 (d, *J* = 29.9 Hz), 158.2, 130.6, 127.7, 113.4, 100.2 (d, *J* = 201.0 Hz), 61.7 (d, *J* = 21.9 Hz), 55.0, 42.8 (d, *J* = 4.9 Hz), 42.6, 42.4, 32.5 ppm. ^{19}F{^{1}H} NMR (376 MHz, DMSO-d_6): δ -155.8 (s) ppm. LCMS (M-H): 249. HRMS (ESI-TOF) *m/z*: $[M - H]^-$ calcd for $C_{14}H_{14}FO_3$, 249.0927; found 249.0919.

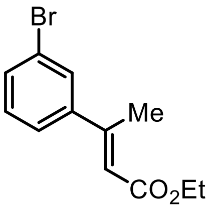

Ethyl-3-(3-bromophenyl)but-2-enoate

General procedure A was used. The final product was purified by distillation (b.p. = 94-95 °C, 0.1 mmHg). Yield: 78.26 g, 0.292 mol, 73%, colorless oil. ^{1}H NMR (500 MHz, $CDCl_3$): δ 7.60 (s, 1H), 7.48 (d, *J* = 7.8 Hz, 1H), 7.38 (d, *J* = 7.6 Hz, 1H), 7.24 (td, *J* = 7.8, 2.9 Hz, 1H), 6.10 (s, 1H), 4.27 – 4.15 (m, 2H), 2.53 (s, 2H), 1.40 – 1.24 (m, 3H) ppm. ^{13}C{^{1}H} NMR (126 MHz, $CDCl_3$): δ 166.6,

153.9, 144.5, 132.0, 130.2, 129.5, 125.1, 122.8, 118.4, 60.2, 18.0, 14.5 ppm. LCMS (M+H): 269. HRMS (ESI-TOF) *m/z*: $[M + H]^+$ calcd for $C_{12}H_{14}BrO_2$, 269.0177; found 269.0171.

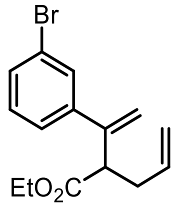

Ethyl 2-(1-(3-bromophenyl)vinyl)pent-4-enoate (11)

General procedure B was used. Yield: 50.06 g, purity ~90%, 0.162 mol, 81%, yellow oil.

An analytically pure sample of the product was obtained by purification of the sample of the crude mixture by HPLC: Rt = 0-5 min, water/acetonitrile, 50-100%, flow 60 mL/min (loading pump 4 mL/min), column: Chomatorex 18 SMB100-5T, 100×19 mm, 5 µm. ^1H NMR (500 MHz, $CDCl_3$): δ 7.54 (s, 1H), 7.41 (d, J = 7.8 Hz, 1H), 7.31 (d, J = 7.6 Hz, 1H), 7.20 (t, J = 7.8 Hz, 1H), 5.84 – 5.69 (m, 1H), 5.41 (s, 1H), 5.32 (s, 1H), 5.07 (d, J = 17.1 Hz, 1H), 5.02 (d, J = 10.1 Hz, 1H), 4.14 (q, J = 6.9 Hz, 1H), 3.54 (t, J = 6.9 Hz, 1H), 2.70 – 2.59 (m, 1H), 2.48 – 2.37 (m, 1H), 1.20 (t, J = 7.0 Hz, 3H) ppm. $^{13}C\{^1H\}$ NMR (126 MHz, $CDCl_3$): δ 173.0, 159.2, 145.3, 143.6, 135.3, 130.8, 129.9, 125.4, 122.6, 117.2, 116.3, 61.0, 50.2, 36.2, 14.2 ppm. HRMS (ESI-TOF) *m/z*: $[M + H]^+$ calcd for $C_{15}H_{18}BrO_2$, 309.0490; found 309.0481.

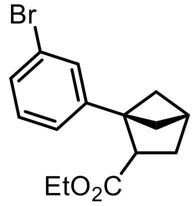

(±)-Ethyl 1-(3-bromophenyl)bicyclo[2.1.1]hexane-2-carboxylate (11a)

General procedure C was used. The final product was purified by distillation (b.p. = 118-119 °C, 0.1 mmHg). Yield: 22.63 g, purity 90%, 0.073 mol, 73%, colorless oil.

An analytically pure sample of the product was obtained by purification of the sample of the crude mixture by HPLC: Rt = 1-7 min, water/acetonitrile, 50-80%, flow 30 mL/min (loading pump 4 mL/min), column: SunFire, 100×19 mm, 5 µm. ^1H NMR (500 MHz, $CDCl_3$): δ 7.35 – 7.30 (m, 1H), 7.29 (t, J = 1.7 Hz, 1H), 7.14 (t, J = 7.7 Hz, 1H), 7.10 – 7.06 (m, 1H), 4.00 – 3.86 (m, 2H), 2.97 (ddd, J = 8.4, 4.6, 1.4 Hz, 1H), 2.57 – 2.50 (m, 1H), 2.21 – 2.09 (m, 3H), 1.82 – 1.75 (m, 2H), 1.63 (dd, J = 9.8, 6.7 Hz, 1H), 0.99 (t, J = 7.1 Hz, 3H) ppm. $^{13}C\{^1H\}$ NMR (126 MHz, $CDCl_3$): δ 174.9, 144.7, 129.7, 129.5, 129.3, 124.7, 122.3, 60.2, 57.9, 48.7, 46.5, 38.1, 35.3, 33.9, 14.2 ppm. LCMS (M+H): 309. HRMS (ESI-TOF) *m/z*: $[M + H]^+$ calcd for $C_{15}H_{18}BrO_2$, 311.0470; found 311.0465.

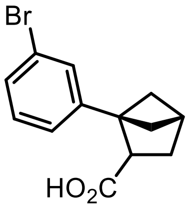

(±)-1-(3-Bromophenyl)bicyclo[2.1.1]hexane-2-carboxylic acid (11b)

General procedure D was used. Yield: 10.93 g, 0.039 mol, 71%, white solid, m.p. = 130-131 °C. ^{1}H NMR (500 MHz, DMSO-d$_6$): δ 11.92 (s, 1H), 7.38 (d, *J* = 8.6 Hz, 1H), 7.34 (s, 1H), 7.25 (t, *J* = 7.7 Hz, 1H), 7.18 (d, *J* = 7.6 Hz, 1H), 3.01 (dd, *J* = 8.8, 3.8 Hz, 1H), 2.46 (s, 1H), 2.13 (t, *J* = 9.9 Hz, 1H), 2.02 (dd, *J* = 9.5, 6.7 Hz, 1H), 1.90 (d, *J* = 10.7 Hz, 1H), 1.83 – 1.75 (m, 1H), 1.70 – 1.66 (m, 1H), 1.63 (dd, *J* = 9.5, 6.5 Hz, 1H) ppm. ^{13}C{^{1}H} NMR (101 MHz, DMSO-d$_6$): δ 175.8, 130.2, 129.0, 128.8, 125.2, 121.4, 56.8, 47.2, 46.2, 37.6, 34.6, 33.8 ppm. LCMS (M-H): 279. HRMS (ESI-TOF) *m/z*: [M - H]$^-$ calcd for $C_{13}H_{12}BrO_2$, 279.0021; found 279.0017.

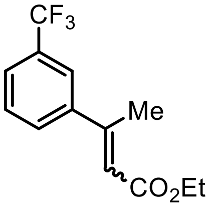

Ethyl-3-(3-(trifluoromethyl)phenyl)but-2-enoate

General procedure A was used. The final product was purified by distillation (b.p. = 69-70 °C, 0.1 mmHg). Yield: 92.56 g, 0.356 mol, 89%, colorless oil. A mixture of *cis*+*trans*-isomers: ~4:1. ^{1}H NMR (500 MHz, DMSO-d$_6$): δ 7.87 (d, *J* = 12.1 Hz), 7.76 (d, *J* = 7.7 Hz) 2H, 7.71 – 7.46 (m, 2H), 6.24 (s), 6.04 (s) 1H, 4.16 (q, *J* = 7.1 H), 3.90 (q, *J* = 7.1 Hz) 2H, 2.54 (s), 2.18 (s) 2H, 1.24 (t, *J* = 7.0 Hz), 0.99 (t, *J* = 7.1 Hz) 3H ppm. ^{13}C{^{1}H} NMR (151 MHz, DMSO-d$_6$): δ 165.7, 164.8, 153.4, 153.0, 142.3, 141.6, 131.0, 130.4, 129.7, 129.4 (q, *J* = 31.7 Hz), 128.9, 125.7 (q, *J* = 3.6 Hz), 124.23 (q, *J* = 3.8 Hz), 124.16 (q, *J* = 272.6 Hz), 124.0 (q, *J* = 272.5 Hz), 123.6 (q, *J* = 3.8 Hz), 122.8 (q, *J* = 3.7 Hz), 118.4, 118.1, 59.6, 59.3, 26.3, 17.3, 14.1, 13.7 ppm. ^{19}F{^{1}H} NMR (376 MHz, DMSO-d$_6$): δ -61.50 (s), -61.53 (s) ppm. LCMS (M+H): 259. HRMS (ESI-TOF) *m/z*: [M + H]$^+$ calcd for $C_{13}H_{14}F_3O_2$, 259.0946; found 259.0937.

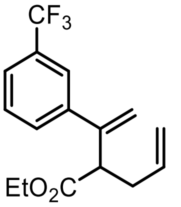

Ethyl 2-(1-(3-(trifluoromethyl)phenyl)vinyl)pent-4-enoate (12)

General procedure B was used. Yield: 23.84 g, purity ~90%, 0.08 mol, 80%, colorless oil.

An analytically pure sample of the product was obtained by purification of the sample of the crude mixture by HPLC: Rt = 1-7 min, water/acetonitrile, 50-80%, flow 30 mL/min (loading pump 4 mL/min), column: Chomatorex 18 SMB100-5T, 100×19 mm, 5 µm. ^{1}H NMR (500 MHz, $CDCl_3$): δ 7.65 (s, 1H), 7.60 (dd, *J* = 13.3, 7.9 Hz, 2H), 7.50 (t, *J* = 7.7 Hz, 1H), 6.05 (s, 1H), 5.85 – 5.66 (m, 1H), 5.03 – 4.88 (m, 2H), 4.23 (q, *J* = 7.1 Hz, 1H), 3.30 – 3.13 (m, 2H), 2.26 – 2.08 (m, 2H), 1.32 (t, *J* = 7.1 Hz, 3H) ppm. ^{13}C{^{1}H} NMR (126 MHz, $CDCl_3$): δ 166.1, 158.1, 142.3, 137.4, 131.2 (q, *J* = 32.4 Hz), 130.2, 129.3, 125.6 (q, *J* = 3.7 Hz), 123.7 (q, *J* = 3.8 Hz), 124.1 (q, *J* = 272.4 Hz), 119.5, 115.4, 60.3, 32.9, 30.4, 14.4 ppm. ^{19}F{^{1}H} NMR (376 MHz, $CDCl_3$): δ -63.2 (s) ppm. LCMS (M+H): 299. HRMS (ESI-TOF) *m/z*: $[M + H]^+$ calcd for $C_{16}H_{18}F_3O_2$, 299.1259; found 299.1250.

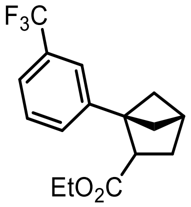

(±)-Ethyl 1-(3-(trifluoromethyl)phenyl)bicyclo[2.1.1]hexane-2-carboxylate (12a)

General procedure C was used. The final product was purified by distillation (b.p. = 81-82 °C, 0.1 mmHg). Yield: 21.75 g, purity ~90%, 0.073 mol, 73%, white solid, m.p. = 132-133 °C.

An analytically pure sample of the product was obtained by purification of the sample of the crude mixture by HPLC: Rt = 1-7 min, water/acetonitrile, 50-80%, flow 30 mL/min (loading pump 4 mL/min), column: Chomatorex 18 SMB100-5T, 100×19 mm, 5 µm. A mixture of isomers: 9:1 (the sample contains ca. 10% of the isomeric 1,5-disubstituted bicyclo[2.1.1]hexane). ^{1}H NMR (500 MHz, $CDCl_3$): δ 7.47 – 7.34 (m, 4H), 4.19 – 4.07 (m), 3.95 – 3.87 (m) 2H, 3.08 – 2.95 (m, 1H), 2.57 (s, 1H), 2.21 – 2.10 (m, 3H), 1.85 – 1.80 (m, 2H), 1.68 (dd, *J* = 9.8, 6.7 Hz, 1H), 1.24 (t, *J* = 7.1 Hz), 0.93 (t, *J* = 7.1 Hz) 3H ppm. ^{13}C{^{1}H} NMR (126 MHz, $CDCl_3$): δ 174.9, 171.0, 143.3, 143.3, 130.5 (q, *J* = 32.0 Hz), 129.5, 128.6, 126.6 (q, *J* = 272.4 Hz), 123.7 (q, *J* = 4.1 Hz), 123.4 (q, *J* = 3.7 Hz), 123.3 (q, *J* = 3.8 Hz), 122.9 (q, *J* = 3.6 Hz), 60.2, 58.0, 56.9, 53.2, 48.7, 46.5, 41.6, 39.9, 38.1, 35.4, 33.9, 30.4, 26.7, 14.3, 14.0 ppm. ^{19}F{^{1}H} NMR (376 MHz, $CDCl_3$): δ -63.02 (s), -63.07 (s) ppm. LCMS (M-H): 299. HRMS (ESI-TOF) *m/z*: $[M + H]^+$ calcd for $C_{16}H_{18}F_3O_2$, 299.1259; found 299.1250.

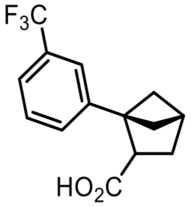

(±)-1-(3-(Trifluoromethyl)phenyl)bicyclo[2.1.1]hexane-2-carboxylic acid (12b)

General procedure D was used. Yield: 10.43 g, 0.0385 mol, 70%, colorless oil. ^{1}H NMR (500 MHz, DMSO-d$_6$): δ 11.94 (s, 1H), 7.58 – 7.45 (m, 4H), 3.07 (dd, *J* = 8.9, 3.3 Hz, 1H), 2.49 (s, 1H), 2.15 (t, *J* = 9.9 Hz, 1H), 2.05 (dd, *J* = 9.4, 6.7 Hz, 1H), 1.96 – 1.88 (m, 1H), 1.85 – 1.80 (m, 1H), 1.75 – 1.71 (m, 1H), 1.68 (dd, *J* = 9.4, 6.5 Hz, 1H) ppm. ^{13}C{^{1}H} NMR (126 MHz, DMSO-d$_6$): δ 175.7, 143.6, 130.3, 129.0, 128.7 (q, *J* = 31.3 Hz), 124.3 (q, *J* = 272.3 Hz), 122.9 (q, *J* = 3.8 Hz), 122.4 (q, *J* = 3.8 Hz), 56.8, 47.3, 46.1, 37.6, 34.6, 33.7 ppm. ^{19}F{^{1}H} NMR (376 MHz, DMSO-d$_6$): δ -61.4 (s) ppm. HRMS (ESI-TOF) *m/z*: [M + H]$^+$ calcd for $C_{14}H_{14}F_3O_2$, 271.0946; found 271.0939.

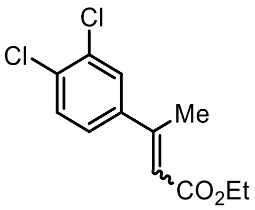

Ethyl-3-(3,4-dichlorophenyl)but-2-enoate

General procedure A was used. The final product was purified by distillation (b.p. = 107-109 °C, 0.1 mmHg). Yield: 91.85 g, 0.356 mol, 89%, white oil. A mixture of *cis*+*trans*-isomers: ~7:3. ^{1}H NMR (500 MHz, $CDCl_3$): δ 7.55 (s, 1H), 7.44 (d, *J* = 8.3 Hz), 7.41 (d, *J* = 9.0 Hz) 1H, 7.30 (d, *J* = 8.6 Hz), 7.04 (d, *J* = 8.2 Hz) 1H, 6.11 (s), 5.93 (s) 1H, 4.22 (q, *J* = 7.0 Hz), 4.03 (q, *J* = 7.0 Hz) 1H, 2.53 (s), 2.14 (s) 1H, 1.31 (t, *J* = 7.0 Hz), 1.13 (t, *J* = 7.0 Hz) 3H ppm. ^{13}C{^{1}H} NMR (151 MHz, $CDCl_3$): δ 166.5, 165.5, 152.72, 152.70, 142.2, 140.9, 133.2, 132.9, 132.2, 131.9, 130.6, 130.1, 129.1, 128.4, 126.7, 125.7, 119.2, 118.6, 60.3, 60.2, 27.0, 26.8, 17.9, 14.5, 14.2 ppm. LCMS (M+H): 259. HRMS (ESI-TOF) *m/z*: [M + H]$^+$ calcd for $C_{12}H_{13}Cl_2O_2$, 259.0293; found 259.0287.

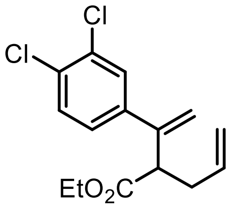

Ethyl 2-(1-(3,4-dichlorophenyl)vinyl)pent-4-enoate (13)

General procedure B was used. Yield: 47.24 g, purity 90%, 0.158 mol, 79%, colorless oil.

An analytically pure sample of the product was obtained by purification of the sample of the crude mixture by HPLC: Rt = 1-9 min, water/acetonitrile, 50-80%, flow 30 mL/min (loading pump 4 mL/min), column: Chomatorex 18 SMB100-5T, 100×19 mm, 5 μm. ^{1}H NMR (500 MHz, $CDCl_3$): δ 7.48 (d, *J* = 1.9 Hz, 1H), 7.39 (d, *J* = 8.4 Hz, 1H), 7.22 (dd, *J* = 8.3, 1.9 Hz, 1H), 5.85 – 5.68 (m, 1H), 5.42 (s, 1H), 5.34 (s, 1H), 5.07 (dd, *J* = 17.1, 1.2 Hz, 1H), 5.03 (d, *J* = 10.2 Hz, 1H), 4.14 (q, *J* = 7.1 Hz, 2H), 3.51 (dd, *J* = 8.3, 6.8 Hz, 1H), 2.74 – 2.59 (m, 1H), 2.51 – 2.32 (m, 1H), 1.21 (t, *J* = 7.1 Hz, 3H) ppm. ^{13}C{^{1}H} NMR (126 MHz, $CDCl_3$): δ 172.9, 144.5, 141.4, 135.1, 132.6, 131.8,

130.4, 128.8, 126.1, 117.4, 116.7, 61.1, 50.1, 36.1, 14.3 ppm. LCMS (M+H): 299. HRMS (ESI-TOF) *m/z*: $[M + H]^+$ calcd for $C_{15}H_{17}Cl_2O_2$, 299.0606; found 299.0600.

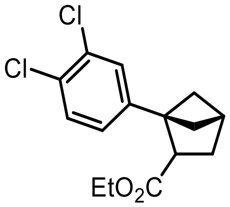

(±)-Ethyl 1-(3,4-dichlorophenyl)bicyclo[2.1.1]hexane-2-carboxylate (13a)

General procedure C was used. The final product was purified by distillation (b.p. = 125-126 °C, 0.1 mmHg). Yield: 21.53 g, purity 90%, 0.072 mol, 72%, yellow oil.

An analytically pure sample of the product was obtained by purification of the sample of the crude mixture by HPLC: Rt = 0-5 min, water/acetonitrile, 50-100%, flow 60 mL/min (loading pump 4 mL/min), column: Chomatorex 18 SMB100-5T, 100×19 mm, 5 µm. ^{1}H NMR (500 MHz, DMSO-d_6): δ 7.54 (d, *J* = 8.2 Hz, 1H), 7.37 (s, 1H), 7.14 (d, *J* = 8.2 Hz, 1H), 3.98 – 3.81 (m, 2H), 3.11 (dd, *J* = 8.7, 3.9 Hz, 1H), 2.47 (s, 1H), 2.11 (t, *J* = 9.8 Hz, 1H), 2.00 – 1.90 (m, 2H), 1.80 (br s, 1H), 1.75 – 1.62 (m, 2H), 0.91 (t, *J* = 7.0 Hz, 3H) ppm. ^{13}C{^{1}H} NMR (126 MHz, DMSO-d_6): δ 173.9, 143.1, 130.6, 130.1, 128.7, 128.1, 126.5, 59.5, 56.6, 47.3, 45.8, 37.5, 34.6, 33.3, 13.9 ppm. LCMS (M+H): 299. HRMS (ESI-TOF) *m/z*: $[M + H]^+$ calcd for $C_{15}H_{17}Cl_2O_2$, 299.0606; found 299.0586.

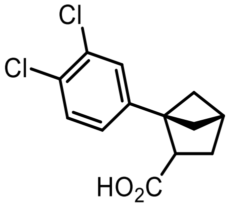

(±)-1-(3,4-Dichlorophenyl)bicyclo[2.1.1]hexane-2-carboxylic acid (13b)

General procedure D was used. Yield: 9.18 g, 0.034 mol, 63%, white solid, m.p. = 108-109 °C. ^{1}H NMR (500 MHz, DMSO-d_6): δ 11.96 (s, 1H), 7.54 (d, *J* = 8.3 Hz, 1H), 7.40 (d, *J* = 1.6 Hz, 1H), 7.17 (dd, *J* = 8.2, 1.6 Hz, 1H), 3.04 (dd, *J* = 8.7, 3.7 Hz, 1H), 2.46 (s, 1H), 2.13 (t, *J* = 9.8 Hz, 1H), 1.97 (dd, *J* = 9.3, 6.9 Hz, 1H), 1.91 (d, *J* = 10.6 Hz, 1H), 1.81 (br s, 1H), 1.72 – 1.66 (m, 1H), 1.63 (dd, *J* = 9.4, 6.6 Hz, 1H) ppm. ^{13}C{^{1}H} NMR (151 MHz, DMSO-d_6): δ 175.6, 143.5, 130.6, 130.1, 128.6, 128.1, 126.6, 56.2, 47.1, 46.1, 37.6, 34.6, 33.6 ppm. LCMS (M-H): 269. HRMS (ESI-TOF) *m/z*: $[M - H]^-$ calcd for $C_{13}H_{11}Cl_2O_2$, 269.0136; found 269.0130.

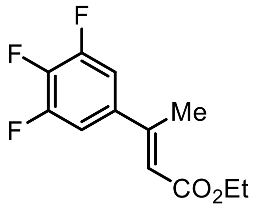

Ethyl-3-(3,4,5-trifluorophenyl)but-2-enoate

General procedure A was used. The final product was purified by distillation (b.p. = 56-57 °C, 0.1 mmHg). Yield: 81.01 g, 0.332 mol, 83%, white solid, m.p. = 62-63 °C. ^{1}H NMR (500 MHz, $CDCl_3$): δ 7.17 – 6.99 (m, 2H), 6.08 (s, 1H), 4.22 (q, *J* = 7.1 Hz, 2H), 2.50 (s, 3H), 1.31 (t, *J* = 7.1 Hz, 3H) ppm. ^{13}C{^{1}H} NMR (151 MHz, $CDCl_3$): δ 166.3, 151.8, 151.3 (ddd, *J* = 250.4, 10.1, 4.1 Hz), 140.1 (dt, *J* = 254.2, 15.5 Hz), 138.3 (q, *J* = 4.7 Hz), 119.0, 110.7 (dd, *J* = 17.3, 4.6 Hz), 60.4, 17.7, 14.4 ppm. ^{19}F{^{1}H} NMR (376 MHz, $CDCl_3$): δ -134.2 (d, *J* = 20.4 Hz), -159.8 (t, *J* = 20.4 Hz) ppm. HRMS (ESI-TOF) *m/z*: $[M + H]^+$ calcd for $C_{12}H_{12}F_3O_2$, 245.0789; found 245.0782.

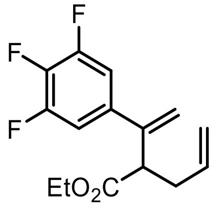

Ethyl 2-(1-(3,4,5-trifluorophenyl)vinyl)pent-4-enoate (14)

General procedure B was used. Yield: 45.44 g, purity ~90%, 0.16 mol, 80%, colorless oil.

An analytically pure sample of the product was obtained by purification of the sample of the crude mixture by HPLC: Rt = 1-7 min, water/acetonitrile, 50-80%, flow 30 mL/min (loading pump 4 mL/min), column: Chomatorex 18 SMB100-5T, 100×19 mm, 5 µm. ^{1}H NMR (500 MHz, $CDCl_3$): δ 7.06 – 6.92 (m, 2H), 5.83 – 5.64 (m, 1H), 5.41 (s, 1H), 5.35 (s, 1H), 5.11 – 5.00 (m, 2H), 4.14 (q, *J* = 7.1 Hz, 2H), 3.55 – 3.34 (m, 1H), 2.75 – 2.54 (m, 1H), 2.45 – 2.32 (m, 1H), 1.21 (t, *J* = 7.1 Hz, 3H) ppm. ^{13}C{^{1}H} NMR (151 MHz, $CDCl_3$): δ 172.7, 151.1 (ddd, *J* = 249.7, 10.6, 4.6 Hz), 144.0, 139.4 (dt, *J* = 251.6, 15.7 Hz), 137.5 (m), 134.9, 117.5, 117.1, 111.0 (d, *J* = 4.9 Hz), 110.9 (d, *J* = 4.9 Hz), 61.2, 50.1, 36.0, 14.2 ppm. ^{19}F{^{1}H} NMR (376 MHz, $CDCl_3$): δ -134.9 (d, *J* = 20.7 Hz), -162.1 (t, *J* = 20.8 Hz) ppm.HRMS (ESI-TOF) *m/z*: $[M + H]^+$ calcd for $C_{15}H_{16}F_3O_2$, 285.1102; found 285.1091.

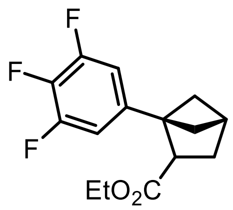

(±)-Ethyl 1-(3,4,5-trifluorophenyl)bicyclo[2.1.1]hexane-2-carboxylate (14a)

General procedure C was used. The final product was purified by distillation (b.p. = 87-88 °C, 0.1 mmHg). Yield: 19.60 g, purity 90%, 0.069 mol, 69%, colorless oil.

An analytically pure sample of the product was obtained by purification of the sample of the crude mixture by HPLC: Rt = 1-7 min, water/acetonitrile, 50-80%, flow 30 mL/min (loading pump 4 mL/min), column: Chomatorex 18 SMB100-5T, 100×19 mm, 5 µm. ^{1}H NMR (500 MHz, $CDCl_3$): δ 6.78 – 6.70 (m, 2H), 4.02 – 3.94 (m, 2H), 2.99 – 2.90 (m, 1H), 2.54 (s, 1H), 2.23 – 2.14 (m, 1H), 2.13 – 2.03 (m, 2H), 1.80 – 1.72 (m, 2H), 1.59 (dd, *J* = 9.7, 6.8 Hz, 1H), 1.05 (t, *J* = 7.1 Hz, 3H) ppm. ^{13}C{^{1}H} NMR (126 MHz, $CDCl_3$): δ 174.6, 151.1 (ddd, *J* = 249.7, 10.2, 4.1 Hz), 138.8 (m), 138.4 (dd, *J* = 264.8, 15.2 Hz), 110.3 (dd, *J* = 16.1, 4.7 Hz), 60.3, 57.2, 48.4, 46.5, 38.2, 35.1, 34.1, 14.2 ppm. ^{19}F{^{1}H} NMR (376 MHz, $CDCl_3$): δ -135.7 (d, *J* = 20.5 Hz), -164.3 (t, *J* = 20.4 Hz) ppm. LCMS (M+H): 285. HRMS (ESI-TOF) *m/z*: $[M + H]^+$ calcd for $C_{15}H_{16}F_3O_2$, 285.1102; found 285.1093.

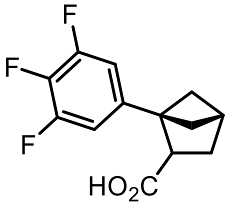

(±)-1-(3,4,5-Trifluorophenyl)bicyclo[2.1.1]hexane-2-carboxylic acid (14b)

General procedure D was used. Yield: 9.22 g, 0.036 mol, 65%, white solid, m.p. = 103-104 °C. ^{1}H NMR (500 MHz, DMSO-d_6): δ 12.00 (s, 1H), 7.12 (dd, *J* = 8.9, 6.8 Hz, 2H), 3.06 (dd, *J* = 9.0, 3.3 Hz, 1H), 2.45 (s, 1H), 2.12 (t, *J* = 9.9 Hz, 1H), 1.95 – 1.87 (m, 2H), 1.84 – 1.77 (m, 1H), 1.69 – 1.65 (m, 1H), 1.61 (dd, *J* = 9.5, 6.5 Hz, 1H) ppm. ^{13}C{^{1}H} NMR (151 MHz, DMSO-d_6): δ 175.5, 149.9 (ddd, *J* = 246.8, 9.5, 3.6 Hz), 139.8 (m), 137.1 (dt, *J* = 247.0, 15.6 Hz), 110.8 (dd, *J* = 16.4, 3.6 Hz), 56.2, 46.9, 46.2, 37.7, 34.3, 33.6 ppm. ^{19}F{^{1}H} NMR (376 MHz, DMSO-d_6): δ -136.7 (d, *J* = 21.6 Hz), -165.5 (t, *J* = 21.7 Hz) ppm. LCMS (M-H): 255. HRMS (ESI-TOF) *m/z*: $[M - H]^-$ calcd for $C_{13}H_{10}F_3O_2$, 255.0633; found 255.0627.

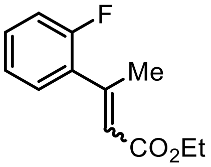

Ethyl-3-(2-fluorophenyl)but-2-enoate

General procedure A was used. The final product was purified by distillation (b.p. = 59-60 °C, 0.1 mmHg). Yield: 72.38 g, 0.348 mol, 87%, colorless oil. A mixture of *cis*+*trans*-isomers: ~3:2. ^{1}H NMR (500 MHz, $CDCl_3$): δ 7.39 – 7.19 (m, 2H), 7.15 – 6.96 (m, 2H), 6.02 (s), 6.00 (s) 1H, 4.22 (q, *J* = 7.1 Hz), 4.01 (q, *J* = 7.1 Hz) 2H, 2.53 (s), 2.17 (s) 3H, 1.31 (t, *J* = 7.1 Hz), 1.08 (t, *J* = 7.1 Hz)

3H ppm. $^{13}C\{^1H\}$ NMR (101 MHz, $CDCl_3$): δ 166.5, 165.4, 159.7 (d, J = 249.3 Hz), 158.6 (d, J = 245.9 Hz), 152.1, 149.4, 131.1 (d, J = 13.4 Hz), 130.1 (d, J = 8.5 Hz), 129.3 (d, J = 3.6 Hz), 128.8 (d, J = 3.8 Hz), 124.3 (d, J = 3.5 Hz), 123.8 (d, J = 3.4 Hz), 120.7 (d, J = 2.6 Hz), 120.3, 116.2 (d, J = 22.5 Hz), 115.5 (d, J = 22.1 Hz), 60.1, 60.0, 26.4 (d, J = 1.1 Hz), 19.5 (d, J = 3.6 Hz), 14.4, 14.1 ppm. $^{19}F\{^1H\}$ NMR (376 MHz, $CDCl_3$): δ -114.9 (s), -116.7 (s) ppm. LCMS (M+H): 209. HRMS (ESI-TOF) *m/z*: $[M + H]^+$ calcd for $C_{12}H_{14}FO_2$, 209.0978; found 209.0973.

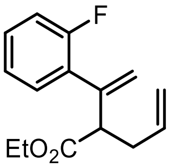

Ethyl 2-(1-(2-fluorophenyl)vinyl)pent-4-enoate (15)

General procedure B was used. Yield: 39.18 g, purity 90%, 0.158 mol, 79%, colorless oil.

An analytically pure sample of the product was obtained by column chromatography, SiO_2, hexane/MeOtBu, 9:1. ^1H NMR (500 MHz, $CDCl_3$): δ 7.28 – 7.19 (m, 2H), 7.09 (t, J = 7.5 Hz, 1H), 7.07 – 7.01 (m, 1H), 5.85 – 5.73 (m, 1H), 5.46 (s, 1H), 5.33 (s, 1H), 5.08 (dd, J = 17.1, 1.4 Hz, 1H), 5.02 (d, J = 10.2 Hz, 1H), 4.17 – 4.05 (m, 2H), 3.55 (dd, J = 9.1, 5.8 Hz, 1H), 2.67 – 2.55 (m, 1H), 2.53 – 2.39 (m, 1H), 1.17 (t, J = 7.1 Hz, 3H) ppm. $^{13}C\{^1H\}$ NMR (151 MHz, $CDCl_3$): δ 173.0, 159.8 (d, J = 247.0 Hz), 142.0, 135.5, 130.6 (d, J = 3.8 Hz), 129.5 (d, J = 14.7 Hz), 129.3 (d, J = 8.3 Hz), 124.1 (d, J = 3.5 Hz), 118.3 (d, J = 1.5 Hz), 117.0, 115.8 (d, J = 22.6 Hz), 60.8, 51.1 (d, J = 2.0 Hz), 35.8, 13.9 (d, J = 86.4 Hz) ppm. $^{19}F\{^1H\}$ NMR (376 MHz, $CDCl_3$): δ -115.4 (s) ppm. LCMS (M+H): 249. HRMS (ESI-TOF) *m/z*: $[M + H]^+$ calcd for $C_{15}H_{18}FO_2$, 249.1291; found 249.1287.

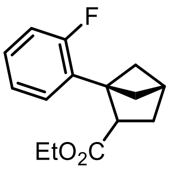

(±)-Ethyl 1-(2-fluorophenyl)bicyclo[2.1.1]hexane-2-carboxylate (15a)

General procedure C was used. The final product was purified by distillation (b.p. = 87-88 °C, 0.1 mmHg). Yield: 18.10 g, purity ~90%, 0.073 mol, 73%, colorless oil.

An analytically pure sample of the product was obtained by purification of the sample of the crude mixture by HPLC: Rt = 0-2-9 min, acetonitrile/water, 52-60-80%, flow 30 mL/min (loading pump 4 mL/min), column: XBridge BEH C18, 100×19 mm, 5 μm. ^1H NMR (500 MHz, $CDCl_3$): δ 7.22 – 6.92 (m, 4H), 3.95 – 3.78 (m, 2H), 3.18 (dd, J = 8.6, 4.1 Hz, 1H), 2.59 – 2.46 (m, 1H), 2.20 – 2.05 (m, 3H), 1.89 – 1.73 (m, 3H), 0.89 (t, J = 7.1 Hz, 3H) ppm. $^{13}C\{^1H\}$ (151 MHz, $CDCl_3$): δ 174.9, 161.4 (d, J = 246.4 Hz), 129.2 (d, J = 15.4 Hz), 128.9 (d, J = 5.6 Hz), 128.2 (d, J = 8.1 Hz), 123.7 (d, J = 3.3 Hz), 115.2 (d, J = 21.6 Hz), 60.0, 55.3, 47.1, 46.6, 38.4, 35.9, 33.3, 14.0 ppm. $^{19}F\{^1H\}$

NMR (376 MHz, $CDCl_3$): δ -116.5 (s) ppm. HRMS (ESI-TOF) *m/z*: $[M + H]^+$ calcd for $C_{15}H_{18}FO_2$, 249.1291; found 249.1286.

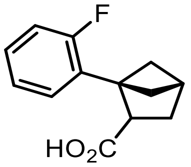

(±)-1-(2-Fluorophenyl)bicyclo[2.1.1]hexane-2-carboxylic acid (15b)

General procedure D was used. Yield: 8.58 g, 0.039 mol, 72%, white solid, m.p. = 110-111 °C. ^1H NMR (500 MHz, DMSO-d_6): δ 11.81 (s, 1H), 7.35 – 6.95 (m, 4H), 3.04 (dd, *J* = 8.4, 3.5 Hz, 1H), 2.14 – 1.93 (m, 3H), 1.83 – 1.73 (m, 2H), 1.69 (t, *J* = 6.9 Hz, 1H) ppm. $^{13}C\{^1H\}$ NMR (151 MHz, DMSO-d_6): δ 175.4, 160.6 (d, *J* = 244.8 Hz), 129.1 (d, *J* = 5.7 Hz), 128.8 (d, *J* = 15.3 Hz), 128.3 (d, *J* = 8.1 Hz), 123.9 (d, *J* = 3.1 Hz), 115.0 (d, *J* = 21.5 Hz), 54.2, 46.2 (d, *J* = 49.2 Hz), 37.9, 35.2, 33.1 ppm. $^{19}F\{^1H\}$ NMR (376 MHz, DMSO-d_6): δ -116.6 (s) ppm. LCMS (M-H): 219. HRMS (ESI-TOF) *m/z*: $[M - H]^-$ calcd for $C_{13}H_{12}FO_2$, 219.0821; found 219.0820.

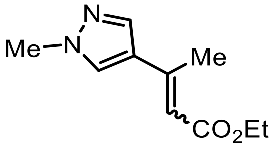

Ethyl-3-(1-methyl-1*H*-pyrazol-4-yl)but-2-enoate

General procedure A was used. The final product was purified by distillation (b.p. = 78-79 °C, 0.1 mmHg). Yield: 53.54 g, 0.276 mol, 69%, colorless oil. A mixture of *cis*+*trans*-isomers: ~1:1. ^1H NMR (500 MHz, $CDCl_3$): δ 8.34 (s), 7.75 (s) 1H, 7.67 (s), 7.52 (s) 1H, 6.08 (d, *J* = 1.2 Hz), 5.69 (d, *J* = 1.1 Hz) 1H, 4.25 – 4.04 (m, 2H), 3.90 (s), 3.89 (s) 2H, 2.46 (d, *J* = 1.1 Hz), 2.20 (d, *J* = 1.1 Hz) 3H, 1.38 – 1.21 (m, 3H) ppm. $^{13}C\{^1H\}$ NMR (126 MHz, $CDCl_3$): δ 167.3, 166.5, 146.9, 144.2, 140.2, 137.3, 132.8, 128.7, 124.8, 119.5, 113.9, 112.4, 59.8, 59.7, 39.3, 39.2, 26.2, 17.3, 14.5, 14.4 ppm. LCMS (M+H): 195. HRMS (ESI-TOF) *m/z*: $[M + H]^+$ calcd for $C_{10}H_{15}N_2O_2$, 195.1134; found 195.1131.

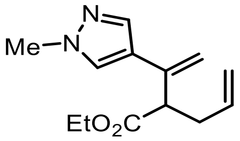

Ethyl 2-(1-(1-methyl-1*H*-pyrazol-4-yl)vinyl)pent-4-enoate (16)

General procedure B was used. The final product was purified by column chromatography, SiO_2, MeO*t*Bu/MeCN, 9:1. Yield: 30.89 g, 0.132 mol, 66%, colorless oil. ^1H NMR (500 MHz, $CDCl_3$): δ 7.56 (s, 1H), 7.43 (s, 1H), 5.84 – 5.70 (m, 1H), 5.36 (s, 1H), 5.08 – 5.04 (m, 2H), 5.01 (d, *J* = 10.2

Hz, 1H), 4.13 (q, J = 7.1 Hz, 2H), 3.86 (s, 3H), 3.37 (dd, J = 8.4, 6.7 Hz, 1H), 2.72 – 2.61 (m, 1H), 2.50 – 2.39 (m, 1H), 1.20 (t, J = 7.1 Hz, 3H) ppm. $^{13}C\{^1H\}$ NMR (126 MHz, $CDCl_3$): δ 173.3, 137.2, 136.9, 135.5, 127.5, 122.4, 116.8, 111.3, 61.0, 51.1, 39.1, 35.4, 14.3 ppm. LCMS (M+H): 235. HRMS (ESI-TOF) *m/z*: $[M + H]^+$ calcd for $C_{13}H_{19}N_2O_2$, 235.1447; found 235.1441.

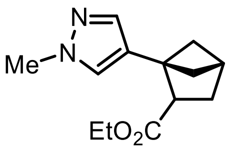

(±)-Ethyl 1-(1-methyl-1*H*-pyrazol-4-yl)bicyclo[2.1.1]hexane-2-carboxylate (16a)

General procedure C was used. The final product was purified by distillation (b.p. = 102-103 °C, 0.1 mmHg). Yield: 14.04 g, purity 90%, 0.06 mol, 60%, colorless oil.

An analytically pure sample of the product was obtained by purification of the sample of the crude mixture by HPLC: Rt = 1-7 min, water/acetonitrile, 40-65%, flow 30 mL/min (loading pump 4 mL/min), column: Chomatorex 18 SMB100-5T, 100×19 mm, 5 µm. A mixture of isomers: 9:1 (the sample contains ca. 10% of the isomeric 1,5-disubstituted bicyclo[2.1.1]hexane). ^1H NMR (500 MHz, $CDCl_3$): δ 7.39 (s), 7.31 (s) 1H, 7.28 (s), 7.18 (s) 1H, 4.13 – 3.97 (m, 2H), 3.85 (s), 3.82 (s) 3H, 2.92 (dd, J = 9.0, 4.2 Hz, 1H), 2.72 (s), 2.46 (s) 1H, 2.17 – 1.81 (m, 3H), 1.78 – 1.57 (m, 2H), 1.42 (dd, J = 9.9, 6.7 Hz, 1H), 1.22 (t, J = 6.9 Hz), 1.15 (t, J = 7.1 Hz) 3H ppm. $^{13}C\{^1H\}$ NMR (126 MHz, $CDCl_3$): δ 175.7, 137.9, 137.8, 128.1, 127.8, 122.5, 60.2, 59.9, 54.0, 50.6, 47.6, 47.1, 40.2, 40.0, 38.9, 38.9, 35.8, 34.2, 14.4, 14.3 ppm. LCMS (M+H): 235. HRMS (ESI-TOF) *m/z*: $[M + H]^+$ calcd for $C_{13}H_{19}N_2O_2$, 235.1447; found 235.1437.

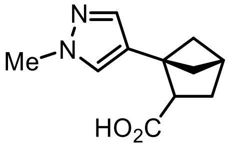

(±)-1-(1-Methyl-1*H*-pyrazol-4-yl)bicyclo[2.1.1]hexane-2-carboxylic acid (16b)

General procedure D was used. Yield: 0.714 g, 0.00346 mol, 63%, white solid. ^1H NMR (500 MHz, DMSO-d_6): δ 11.94 (s, 1H), 7.45 (s, 1H), 7.24 (s, 1H), 3.75 (s, 3H), 2.84 (dd, J = 8.9, 4.0 Hz, 1H), 2.39 (s, 1H), 2.08 – 2.02 (m, 1H), 1.95 (dd, J = 9.7, 6.6 Hz, 1H), 1.81 (d, J = 10.7 Hz, 1H), 1.71 – 1.63 (m, 2H), 1.35 (dd, J = 9.8, 6.5 Hz, 1H) ppm. $^{13}C\{^1H\}$ (126 MHz, DMSO-d_6): δ 176.5, 136.8, 128.1, 121.8, 49.9, 47.2, 45.9, 38.3, 34.8, 33.8 ppm. LCMS (M+H): 207. HRMS (ESI-TOF) *m/z*: $[M + H]^+$ calcd for $C_{11}H_{15}N_2O_2$, 207.1134; found 207.1125.

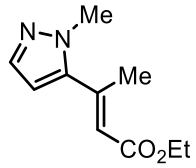

Ethyl-3-(1-methyl-1*H*-pyrazol-5-yl)but-2-enoate

General procedure A was used. The final product was purified by column chromatography, SiO_2, MeO*t*Bu/hexane, 1:9. Yield: 54.32 g, 0.28 mol, 70%, yellow oil. ^{1}H NMR (500 MHz, $CDCl_3$): δ 7.43 (d, *J* = 1.8 Hz, 1H), 6.29 (d, *J* = 1.8 Hz, 1H), 5.93 (d, *J* = 1.1 Hz, 1H), 4.21 (q, *J* = 7.1 Hz, 2H), 3.91 (s, 3H), 2.48 (d, *J* = 1.0 Hz, 3H), 1.31 (t, *J* = 7.1 Hz, 3H) ppm. ^{13}C{^{1}H} NMR (151 MHz, $CDCl_3$): δ 166.1, 144.7, 144.4, 138.5, 120.4, 106.8, 60.3, 38.5, 19.4, 14.4 ppm. LCMS (M+H): 195. HRMS (ESI-TOF) *m/z*: $[M + H]^+$ calcd for $C_{10}H_{15}N_2O_2$, 195.1134; found 195.1127.

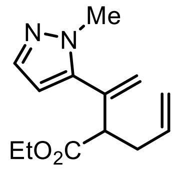

Ethyl 2-(1-(1-methyl-1*H*-pyrazol-5-yl)vinyl)pent-4-enoate (17)

General procedure B was used. The final product was purified by column chromatography, SiO_2, hexane/EtOAc, 9:1. Yield: 32.76 g, 0.14 mol, 70%, colorless oil. ^{1}H NMR (500 MHz, $CDCl_3$): δ 7.41 (d, *J* = 1.8 Hz, 1H), 6.15 (d, *J* = 1.8 Hz, 1H), 5.80 – 5.63 (m, 1H), 5.56 (s, 1H), 5.29 (s, 1H), 5.07 – 5.00 (m, 2H), 4.11 (q, *J* = 7.1 Hz, 2H), 3.83 (s, 3H), 3.38 (t, *J* = 7.5 Hz, 1H), 2.68 – 2.54 (m, 1H), 2.48 – 2.31 (m, 1H), 1.18 (t, *J* = 7.1 Hz, 3H) ppm. ^{13}C{^{1}H} NMR (126 MHz, $CDCl_3$): δ 172.4, 142.2, 138.3, 136.4, 135.0, 119.7, 117.3, 105.4, 61.0, 51.9, 37.5, 35.3, 14.2 ppm. LCMS (M+H): 235. HRMS (ESI-TOF) *m/z*: $[M + H]^+$ calcd for $C_{13}H_{19}N_2O_2$, 235.1447; found 235.1440.

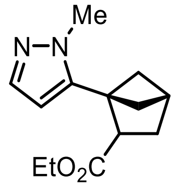

(±)-Ethyl 1-(1-methyl-1*H*-pyrazol-5-yl)bicyclo[2.1.1]hexane-2-carboxylate (17a)

A General procedure C was used. The final product was purified by distillation (b.p. = 103-104 °C, 0.1 mmHg). Yield: 13.34 g, purity 80%, 0.057 mol, 57%, colorless oil.

An analytically pure sample of the product was obtained by purification of the sample of the crude mixture by HPLC: Rt = 1-7 min, water/acetonitrile, 20-45%, flow 30 mL/min (loading pump 4 mL/min), column: Chomatorex 18 SMB100-5T, 100×19 mm, 5 μm. A mixture of isomers: ~4:1 (the sample contains ca. 20% of the isomeric 1,5-disubstituted bicyclo[2.1.1]hexane). ^{1}H NMR (500 MHz, DMSO-d_6): δ 7.25 (s), 7.22 (s) 1H, 6.15 (s), 5.90 (s) 1H, 4.05 (q, *J* = 7.0 Hz), 3.93 – 3.81 (m)

2H, 3.79 (s), 3.73 (s) 3H, 3.19 (dd, J = 8.4, 3.9 Hz, 1H), 2.88 (s), 2.72 (s) 1H, 2.06 (t, J = 9.7 Hz, 1H), 2.02 – 1.91 (m, 2H), 1.88 – 1.76 (m, 2H), 1.73 (dd, J = 9.6, 6.7 Hz, 1H), 1.16 (t, J = 7.1 Hz), 0.91 (t, J = 7.1 Hz) 3H ppm. $^{13}C\{^1H\}$ NMR (151 MHz, $CDCl_3$): δ 174.3, 170.5, 143.2, 142.7, 137.8, 137.8, 106.0, 105.0, 60.4, 60.3, 52.7, 51.00, 50.0, 46.7, 45.8, 41.7, 40.5, 39.0, 38.1, 37.7, 36.4, 32.9, 28.7, 26.1, 14.4, 14.1 ppm. LCMS (M+H): 235. HRMS (ESI-TOF) m/z: $[M + H]^+$ calcd for $C_{13}H_{19}N_2O_2$, 235.1447; found 235.1437.

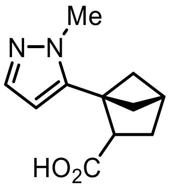

(±)-1-(1-Methyl-1*H*-pyrazol-5-yl)bicyclo[2.1.1]hexane-2-carboxylic acid (17b)

General procedure D was used. The final product was purified by column chromatography, SiO_2, hexane/EtOAc, 4:1. Yield: 7.42 g, 0.036 mol, 65%, white solid, m.p. = 231-232 °C. ^1H NMR (500 MHz, DMSO-d_6): δ 12.01 (s, 1H), 7.22 (s, 1H), 5.94 (s, 1H), 3.73 (s, 3H), 3.09 (dd, J = 8.6, 3.7 Hz, 1H), 2.50 – 2.47 (m, 1H), 2.07 (t, J = 9.8 Hz, 1H), 2.02 (dd, J = 9.5, 6.8 Hz, 1H), 1.95 (d, J = 10.7 Hz, 1H), 1.87 – 1.82 (m, 1H), 1.81 – 1.76 (m, 1H), 1.69 (dd, J = 9.5, 6.6 Hz, 1H) ppm. $^{13}C\{^1H\}$ NMR (126 MHz, DMSO-d_6): δ 175.4, 137.0, 104.6, 50.0, 45.8, 45.1, 38.6, 37.4, 35.6, 32.8 ppm. LCMS (M+H): 207. HRMS (ESI-TOF) m/z: $[M + H]^+$ calcd for $C_{11}H_{15}N_2O_2$, 207.1134; found 207.1128.

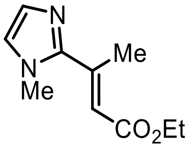

Ethyl-3-(1-methyl-1*H*-imidazol-2-yl)but-2-enoate

General procedure A was used. The final product was purified by distillation (b.p. = 87-88 °C, 0.1 mmHg). Yield: 55.10 g, 0.284 mol, 71%, colorless oil. ^1H NMR (500 MHz, DMSO-d_6): δ 7.27 (s, 1H), 6.99 (s, 1H), 6.10 (s, 1H), 4.15 (q, J = 7.1 Hz, 2H), 3.75 (s, 3H), 1.24 (t, J = 7.1 Hz, 3H) ppm. $^{13}C\{^1H\}$ NMR (151 MHz, $CDCl_3$): δ 166.3, 147.9, 144.8, 128.7, 123.9, 120.1, 60.3, 35.4, 18.3, 14.4 ppm. LCMS (M+H): 195. HRMS (ESI-TOF) m/z: $[M + H]^+$ calcd for $C_{10}H_{15}N_2O_2$, 195.1134; found 195.1133.

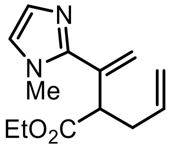

Ethyl 2-(1-(1-methyl-1*H*-imidazol-2-yl)vinyl)pent-4-enoate (18)

General procedure B was used. The product was purified by column chromatography, SiO_2, hexane/EtOAc, 9:1. Yield: 32.29 g, 0.138 mol, 69%, yellow oil. ^{1}H NMR (500 MHz, $CDCl_3$): δ 7.02 (s, 1H), 6.84 (s, 1H), 5.85 – 5.72 (m, 1H), 5.57 (s, 1H), 5.33 (s, 1H), 5.05 (dd, *J* = 17.1, 1.2 Hz, 1H), 4.99 (d, *J* = 10.1 Hz, 1H), 4.10 (q, *J* = 7.0 Hz, 2H), 3.66 (s, 3H), 2.69 – 2.63 (m, 1H), 2.57 – 2.50 (m, 1H), 1.16 (t, *J* = 7.1 Hz, 3H) ppm. ^{13}C{^{1}H} NMR (126 MHz, $CDCl_3$): δ 173.1, 147.1, 136.7, 135.6, 128.0, 122.3, 117.9, 116.8, 60.8, 50.1, 35.6, 34.6, 14.3 ppm. HRMS (ESI-TOF) *m/z*: $[M + H]^+$ calcd for $C_{13}H_{19}N_2O_2$, 235.1447; found 235.1439.

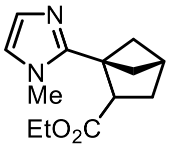

(±)-Ethyl 1-(1-methyl-1*H*-imidazol-2-yl)bicyclo[2.1.1]hexane-2-carboxylate (18a)

General procedure C was used. The final product was purified by distillation (b.p. = 124-125 °C, 0.1 mmHg). Yield: 13.81 g, purity ~90%, 0.059 mol, 59%, colorless oil.

An analytically pure sample of the product was obtained by purification of the sample of the crude mixture by HPLC: Rt = 1-7 min, water/acetonitrile, 25-50%, flow 30 mL/min (loading pump 4 mL/min), column: Chomatorex 18 SMB100-5T, 100×19 mm, 5 µm. ^{1}H NMR (500 MHz, $CDCl_3$): δ 6.88 (d, *J* = 0.9 Hz, 1H), 6.71 (d, *J* = 0.7 Hz, 1H), 4.02 – 3.86 (m, 2H), 3.60 (s, 3H), 3.18 (dd, *J* = 8.5, 4.8 Hz, 1H), 2.53 (s, 1H), 2.14 – 2.03 (m, 4H), 1.99 – 1.85 (m, 1H), 1.76 – 1.66 (m 1H), 1.01 (t, *J* = 7.1 Hz, 3H) ppm. ^{13}C{^{1}H} NMR (126 MHz, $CDCl_3$): δ 174.4, 147.5, 127.3, 121.1, 60.4, 52.1, 47.0, 45.8, 38.7, 36.3, 33.5, 32.5, 14.1 ppm. LCMS (M+H): 235. HRMS (ESI-TOF) *m/z*: $[M + H]^+$ calcd for $C_{13}H_{19}N_2O_2$, 235.1447; found 235.1438.

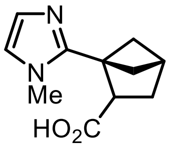

(±)-1-(1-Methyl-1*H*-imidazol-2-yl)bicyclo[2.1.1]hexane-2-carboxylic acid (18b)

General procedure D was used. The product was purified by column chromatography, SiO_2, hexane/EtOAc, 4:1. Yield: 7.42 g, 0.036 mol, 65%, yellow solid, m.p. = 172-173 °C. ^{1}H NMR (500 MHz, DMSO-d_6): δ 12.47 (br s, 1H), 6.97 (d, *J* = 0.8 Hz, 1H), 6.70 (d, *J* = 0.9 Hz, 1H), 3.60 (s, 3H), 3.12 (dd, *J* = 9.3, 3.8 Hz, 1H), 2.44 (s, 1H), 2.09 – 1.93 (m, 3H), 1.90 (dd, *J* = 9.6, 7.0 Hz, 1H), 1.87 – 1.81 (m, 1H), 1.61 (dd, *J* = 9.6, 6.6 Hz, 1H) ppm. ^{13}C{^{1}H} NMR (126 MHz, DMSO-d_6): δ 175.4, 146.8, 125.7, 121.8, 51.6, 45.9, 45.2, 38.3, 35.4, 33.2, 32.2 ppm. LCMS (M+H): 207. HRMS (ESI-TOF) *m/z*: $[M + H]^+$ calcd for $C_{11}H_{15}N_2O_2$, 207.1134; found 207.1127.

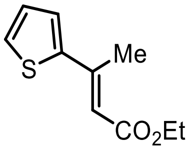

Ethyl-3-(thiophen-2-yl)but-2-enoate

General procedure A was used. The final product was purified by distillation (b.p. = 62-63 °C, 0.1 mmHg). Yield: 62.72 g, 0.32 mol, 80%, colorless oil. ^{1}H NMR (500 MHz, $CDCl_3$): δ 7.32 (s, 1H), 7.31 (s, 1H), 7.04 (t, *J* = 4.4 Hz, 1H), 6.25 (s, 1H), 4.20 (q, *J* = 7.1 Hz, 2H), 2.61 (s, 3H), 1.31 (t, *J* = 7.1 Hz, 3H) ppm. $^{13}C\{^1H\}$ NMR (126 MHz, $CDCl_3$): δ 166.9, 147.9, 145.7, 128.0, 127.2, 126.8, 114.4, 60.0, 17.4, 14.5 ppm. HRMS (ESI-TOF) *m/z*: $[M + H]^+$ calcd for $C_{10}H_{13}O_2S$, 197.0636; found 197.0628.

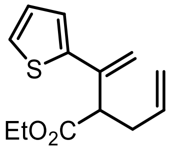

Ethyl 2-(1-(thiophen-2-yl)vinyl)pent-4-enoate (19)

General procedure B was used. Yield: 33.98 g, purity ~90%, 0.144 mol, 72%, colorless oil.

An analytically pure sample of the product was obtained by purification of the sample of the crude mixture by HPLC: Rt = 0-6 min, water/acetonitrile, 40-80%, flow 60 mL/min (loading pump 4 mL/min), column: XBridge OBD 30×100, 5 μm. ^{1}H NMR (500 MHz, $CDCl_3$): δ 7.32 (d, *J* = 5.1 Hz, 1H), 7.31 (d, *J* = 3.6 Hz, 1H), 7.05 (dd, *J* = 4.9, 3.9 Hz, 1H), 6.22 (s, 1H), 5.96 – 5.85 (m, 1H), 5.06 (dd, *J* = 17.1, 1.5 Hz, 1H), 4.98 (d, *J* = 10.1 Hz, 1H), 4.20 (q, *J* = 7.1 Hz, 2H), 3.23 – 3.08 (m, 2H), 2.34 (dd, *J* = 15.2, 7.3 Hz, 2H), 1.31 (t, *J* = 7.1 Hz, 3H) ppm. $^{13}C\{^1H\}$ NMR (126 MHz, $CDCl_3$): δ 166.4, 152.0, 144.8, 137.8, 128.1, 127.3, 126.8, 115.1, 114.6, 60.0, 33.9, 30.8, 14.5 ppm. HRMS (ESI-TOF) *m/z*: $[M + H]^+$ calcd for $C_{13}H_{17}O_2S$, 237.0949; found 237.0941.

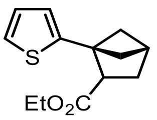

(±)-Ethyl 1-(thiophen-2-yl)bicyclo[2.1.1]hexane-2-carboxylate (19a)

General procedure C was used. The final product was purified by distillation (b.p. = 88-89 °C, 0.1 mmHg). Yield: 17.94 g, purity ~90%, 0.076 mol, 76%, colorless oil.

An analytically pure sample of the product was obtained by purification of the sample of the crude mixture by HPLC: Rt = 0-5 min, water/acetonitrile, 40-90%, flow 60 mL/min (loading pump 4 mL/min), column: XBridge OBD 30×100, 5 μm. ^{1}H NMR (500 MHz, $CDCl_3$): δ 7.14 (d, *J* = 5.1 Hz, 1H), 6.90 (dd, *J* = 4.9, 3.6 Hz, 1H), 6.81 (d, *J* = 3.4 Hz, 1H), 4.17 – 3.90 (m, 2H), 3.04 (dd, *J* =

8.9, 4.2 Hz, 1H), 2.51 (s, 1H), 2.22 (dd, *J* = 9.8, 7.0 Hz, 1H), 2.16 (t, *J* = 10.0 Hz, 1H), 2.09 – 1.99 (m, 1H), 1.92 – 1.87 (m, 2H), 1.57 (dd, *J* = 9.8, 6.7 Hz, 1H), 1.10 (t, *J* = 7.1 Hz, 3H) ppm. $^{13}C\{^1H\}$ NMR (126 MHz, $CDCl_3$): δ 175.2, 145.6, 126.7, 123.7, 123.6, 60.3, 54.6, 48.6, 48.2, 40.6, 35.7, 34.4, 14.2 ppm. HRMS (ESI-TOF) *m/z*: $[M + H]^+$ calcd for $C_{13}H_{17}O_2S$, 237.0949; found 237.0941.

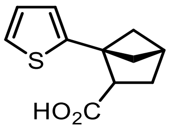

(±)-1-(Thiophen-2-yl)bicyclo[2.1.1]hexane-2-carboxylic acid (19b)

General procedure D was used. Yield: 8.35 g, 0.04 mol, 73%, beige solid, m.p. = 103-104 °C. ^1H NMR (500 MHz, DMSO-d_6): δ 12.07 (s, 1H), 7.33 (d, *J* = 4.6 Hz, 1H), 6.95 – 6.91 (m, 1H), 6.85 (s, 1H), 3.02 – 2.95 (m, 1H), 2.44 (s, 1H), 2.19 – 2.07 (m, 2H), 1.86 (d, *J* = 10.6 Hz, 1H), 1.83 – 1.73 (m, 2H), 1.53 (t, *J* = 7.6 Hz, 1H) ppm. $^{13}C\{^1H\}$ NMR (126 MHz, DMSO-d_6): δ 176.0, 145.3, 126.7, 123.9, 123.7, 53.4, 48.2, 46.9, 40.3, 34.8, 34.1 ppm. LCMS (M+H): 209. HRMS (ESI-TOF) *m/z*: $[M - H]^-$ calcd for $C_{11}H_{11}O_2S$, 207.0480; found 207.0480.

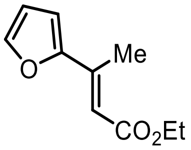

Ethyl-3-(furan-2-yl)but-2-enoate

General procedure A was used. The final product was purified by distillation (b.p. = 39-40 °C, 0.1 mmHg). Yield: 58.32 g, 0.324 mol, 81%, colorless oil. ^1H NMR (500 MHz, $CDCl_3$): δ 7.43 (d, *J* = 1.3 Hz, 1H), 6.63 (d, *J* = 3.4 Hz, 1H), 6.45 (dd, *J* = 3.4, 1.8 Hz, 1H), 6.36 (s, 1H), 4.19 (q, *J* = 7.1 Hz, 2H), 2.45 (d, *J* = 1.1 Hz, 3H), 1.30 (t, *J* = 7.1 Hz, 3H) ppm. $^{13}C\{^1H\}$ NMR (126 MHz, $CDCl_3$): δ 167.3, 154.5, 144.0, 142.2, 112.6, 112.1, 111.3, 59.9, 14.9, 14.5 ppm. HRMS (ESI-TOF) *m/z*: $[M + H]^+$ calcd for $C_{10}H_{13}O_3$, 181.0865; found 181.0857.

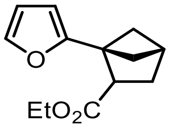

(±)-Ethyl 1-(furan-2-yl)bicyclo[2.1.1]hexane-2-carboxylate (20a)

General procedure C was used. The final product was purified by distillation (b.p. = 69-70 °C, 0.1 mmHg). Yield: 16.06 g, purity ~90%, 0.073 mol, 73%, colorless oil.

An analytically pure sample of the product was obtained by purification of the sample of the crude mixture by HPLC: Rt = 0-2-9 min, acetonitrile/water, 32-40-65%, flow 30 mL/min (loading pump 4 mL/min), column: Chomatorex 18 SMB100-5T, 100×19 mm, 5 μm. ^1H NMR (500 MHz, $CDCl_3$): δ

7.30 (s, 1H), 7.26 (s, 1H), 6.27 (dd, *J* = 3.0, 1.8 Hz, 1H), 6.06 (d, *J* = 3.1 Hz, 1H), 4.11 – 3.99 (m, 2H), 3.09 (dd, *J* = 8.8, 3.3 Hz, 1H), 2.49 (s, 1H), 2.14 – 2.00 (m, 3H), 1.96 – 1.89 (m, *J* = 10.3, 3.9 Hz, 2H), 1.45 (dd, *J* = 9.8, 6.7 Hz, 1H), 1.15 (t, *J* = 7.1 Hz, 3H) ppm. $^{13}C\{^1H\}$ NMR (151 MHz, $CDCl_3$): δ 175.3, 155.4, 141.2, 110.1, 105.3, 60.3, 52.4, 46.4, 45.7, 39.1, 35.8, 33.7, 14.3 ppm. HRMS (ESI-TOF) *m/z*: $[M + H]^+$ calcd for $C_{13}H_{17}O_3$, 221.1178; found 221.1173.

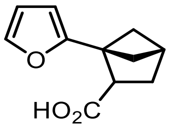

(±)-1-(Furan-2-yl)bicyclo[2.1.1]hexane-2-carboxylic acid (20b)

General procedure D was used. Yield: 7.92 g, 0.041 mol, 75%, yellow solid, m.p. = 79-80 °C. ^1H NMR (500 MHz, DMSO-d_6): δ 12.08 (s, 1H), 7.51 (s, 1H), 6.35 (dd, *J* = 2.8, 1.7 Hz, 1H), 6.12 (d, *J* = 3.0 Hz, 1H), 2.99 (dd, *J* = 8.9, 3.2 Hz, 1H), 2.43 (s, 1H), 2.10 (t, *J* = 9.8 Hz, 1H), 1.96 (dd, *J* = 9.6, 6.7 Hz, 1H), 1.90 – 1.77 (m, 3H), 1.38 (dd, *J* = 9.5, 6.5 Hz, 1H) ppm. $^{13}C\{^1H\}$ NMR (126 MHz, DMSO-d_6): δ 176.1, 154.9, 141.4, 110.3, 105.2, 51.3, 46.0, 44.4, 34.9, 33.4 ppm. LCMS (M-H): 191. HRMS (ESI-TOF) *m/z*: $[M + H]^+$ calcd for $C_{11}H_{13}O_3$, 193.0865; found 193.0858.

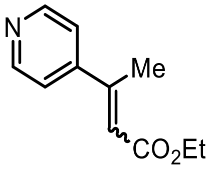

Ethyl-3-(pyridin-4-yl)but-2-enoate

General procedure A was used. The final product was purified by distillation (b.p. = 72-73 °C, 0.1 mmHg). Yield: 55.77 g, 0.292 mol, 73%, yellow oil. A mixture of *cis*+*trans*-isomers: ~7:3. ^1H NMR (500 MHz, DMSO-d_6): δ 8.61 (d, *J* = 6.1 Hz), 8.54 (d, *J* = 5.9 Hz) 2H, 7.55 (d, *J* = 6.1 Hz), 7.20 (d, *J* = 5.9 Hz) 2H, 6.32 (d, *J* = 1.1 Hz), 6.04 (d, *J* = 1.2 Hz) 1H, 4.16 (q, *J* = 7.1 Hz), 3.92 (q, *J* = 7.1 Hz) 2H, 2.49 (d, *J* = 1.0 Hz), 2.14 (d, *J* = 1.2 Hz) 3H, 1.24 (t, *J* = 7.1 Hz), 1.02 (t, *J* = 7.1 Hz) 3H ppm. $^{13}C\{^1H\}$ NMR (126 MHz, DMSO-d_6): δ 165.6, 164.6, 152.6, 151.7, 150.1, 149.2, 148.1, 121.8, 120.7, 119.0, 118.5, 59.8, 59.5, 25.8, 16.7, 14.1, 13.7 ppm. LCMS (M+H): 192. HRMS (ESI-TOF) *m/z*: $[M + H]^+$ calcd for $C_{11}H_{14}NO_2$, 192.1025; found 192.1019.

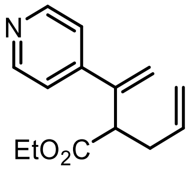

Ethyl 2-(1-(pyridin-4-yl)vinyl)pent-4-enoate (21)

General procedure B was used. Yield: 31.88 g, purity 90%, 0.138 mol, 69%, colorless oil. ^{1}H NMR (500 MHz, $CDCl_3$): δ 8.57 (s, 2H), 7.31 (d, J = 5.0 Hz, 2H), 5.81 – 5.67 (m, 1H), 5.60 (s, 1H), 5.46 (s, 1H), 5.07 (d, J = 17.1 Hz, 1H), 5.03 (d, J = 10.3 Hz, 1H), 4.14 (q, J = 7.1 Hz, 2H), 3.56 (t, J = 6.9 Hz, 1H), 2.74 – 2.61 (m, 1H), 2.49 – 2.35 (m, 1H), 1.20 (t, J = 7.1 Hz, 3H) ppm. $^{13}C\{^1H\}$ NMR (151 MHz, $CDCl_3$): δ 172.8, 150.0, 148.7, 144.2, 135.0, 121.3, 118.0, 117.4, 61.1, 49.4, 36.0, 14.2 ppm. GCMS (M): 231. HRMS (ESI-TOF) m/z: $[M + H]^+$ calcd for $C_{14}H_{18}NO_2$, 232.1338; found 232.1334.

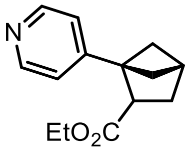

(±)-Ethyl 1-(pyridin-4-yl)bicyclo[2.1.1]hexane-2-carboxylate (21a)

General procedure C was used. The final product was purified by distillation (b.p. = 98-99 °C, 0.1 mmHg). Yield: 16.40 g, purity ~90%, 0.071 mol, 71%, colorless oil.

An analytically pure sample of the product was obtained by purification of the sample of the crude mixture by HPLC: Rt = 0-1-5 min, water/acetonitrile, 30-30-80%, flow 60 mL/min (loading pump 4 mL/min), column: XBridge OBD 30×100, 5 μm. ^{1}H NMR (500 MHz, $CDCl_3$): δ 8.48 (d, J = 4.9 Hz, 2H), 7.07 (d, J = 5.5 Hz, 2H), 3.92 (q, J = 7.1 Hz, 2H), 3.02 (dd, J = 8.7, 4.1 Hz, 1H), 2.56 (s, 1H), 2.17 (t, J = 10.0 Hz, 1H), 2.14 – 2.08 (m, 2H), 1.83 – 1.78 (m, 2H), 1.64 (dd, J = 9.7, 6.8 Hz, 1H), 0.97 (t, J = 7.1 Hz, 3H) ppm. $^{13}C\{^1H\}$ NMR (126 MHz, $CDCl_3$): δ 174.6, 151.1, 149.6, 121.5, 60.3, 57.2, 48.3, 46.5, 37.8, 35.6, 33.9, 14.1 ppm. LCMS (M+H): 232. HRMS (ESI-TOF) m/z: $[M + H]^+$ calcd for $C_{14}H_{18}NO_2$, 232.1338; found 232.1330.

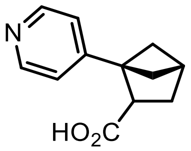

(±)-1-(Pyridin-4-yl)bicyclo[2.1.1]hexane-2-carboxylic acid (21b)

General procedure D was used. Yield: 7.92 g, 0.039 mol, 70%, yellow solid, m.p. = 197-198 °C. ^{1}H NMR (500 MHz, DMSO-d_6): δ 12.02 (br s, 1H), 8.73 – 8.26 (m, 2H), 7.32 – 7.00 (m, 2H), 3.14 – 3.01 (m, 1H), 2.50 – 2.44 (m, 1H), 2.16 (t, J = 9.8 Hz, 1H), 2.03 – 1.88 (m, 2H), 1.84 (br s, 1H), 1.76 – 1.58 (m, 2H) ppm. $^{13}C\{^1H\}$ NMR (126 MHz, DMSO-d_6): δ 175.6, 150.8, 149.2, 121.6, 56.2, 46.8, 46.2, 37.3, 34.8, 33.6 ppm. LCMS (M+H): 204. HRMS (ESI-TOF) m/z: $[M - H]^-$ calcd for $C_{12}H_{12}NO_2$, 202.0868; found 202.0868.

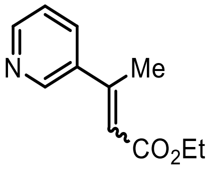

Ethyl-3-(pyridin-3-yl)but-2-enoate

General procedure A was used. The final product was purified by distillation (b.p. = 68-69 °C, 0.1 mmHg). Yield: 53.48 g, 0.28 mol, 70%, colorless oil. A mixture of *cis*+*trans*-isomers: ~3:2. ^{1}H NMR (500 MHz, DMSO-d$_6$): δ 8.78 (d, *J* = 1.8 Hz), 8.41 (d, *J* = 1.5 Hz) 1H, 8.59 (dd, *J* = 4.7, 1.3 Hz), 8.50 (dd, *J* = 4.8, 1.4 Hz) 1H, 8.01 – 7.98 (m), 7.68 – 7.64 (m) 1H, 7.44 (dd, *J* = 7.6, 4.8 Hz), 7.38 (dd, *J* = 7.7, 4.8 Hz) 1H, 6.24 (d, *J* = 1.1 Hz), 6.06 (d, *J* = 1.3 Hz) 1H, 4.16 (q, *J* = 7.1 Hz), 3.93 (q, *J* = 7.1 Hz) 2H, 3.32 (s), 3.29 (s) 3H, 2.53 (d, *J* = 1.0 Hz), 2.18 (d, *J* = 1.3 Hz) 3H, 1.25 (t, *J* = 7.1 Hz), 1.03 (t, *J* = 7.1 Hz) 3H ppm. ^{13}C{^{1}H} NMR (126 MHz, $CDCl_3$): δ 165.7, 164.8, 152.1, 151.8, 150.0, 148.6, 147.6, 147.2, 136.6, 136.1, 134.5, 133.8, 123.5, 122.9, 118.6, 117.7, 59.7, 59.4, 26.3, 17.1, 14.2, 13.8 ppm. LCMS (M+H): 192. HRMS (ESI-TOF) *m/z*: $[M + H]^+$ calcd for $C_{11}H_{14}NO_2$, 192.1025; found 192.1025.

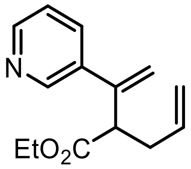

Ethyl 2-(1-(pyridin-3-yl)vinyl)pent-4-enoate (22)

General procedure B was used. Yield: 32.34 g, purity 90%, 0.14 mol, 70%, colorless oil.

An analytically pure sample of the product was obtained by purification of the sample of the crude mixture by column chromatography, SiO_2, hexane/EtOAc, 9:1. ^{1}H NMR (500 MHz, DMSO-d$_6$): δ 8.61 (s, 1H), 8.50 (d, *J* = 4.6 Hz, 1H), 7.81 (d, *J* = 7.9 Hz, 1H), 7.38 (dd, *J* = 7.9, 4.8 Hz, 1H), 5.84 – 5.69 (m, 1H), 5.54 (s, 1H), 5.33 (s, 1H), 5.03 (dd, *J* = 25.8, 13.7 Hz, 2H), 4.10 – 3.97 (m, 2H), 3.77 – 3.68 (m, 1H), 2.58 – 2.51 (m, 1H), 2.44 – 2.35 (m, 1H), 1.06 (t, *J* = 7.1 Hz, 3H) ppm. ^{13}C{^{1}H} NMR (126 MHz, DMSO-d$_6$): δ 172.1, 148.8, 147.4, 142.9, 135.9, 135.3, 133.7, 123.3, 117.1, 116.6, 60.3, 48.8, 35.1, 13.9 ppm. LCMS (M+H): 232. HRMS (ESI-TOF) *m/z*: $[M + H]^+$ calcd for $C_{14}H_{18}NO_2$, 232.1338; found 232.1331.

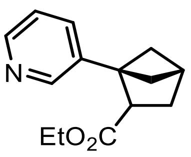

(±)-Ethyl 1-(pyridin-3-yl)bicyclo[2.1.1]hexane-2-carboxylate (22a)

General procedure C was used. The final product was purified by distillation (b.p. = 98-99 °C, 0.1 mmHg). Yield: 15.71 g, purity ~90%, 0.068 mol, 68%, colorless oil.

An analytically pure sample of the product was obtained by purification of the sample of the crude mixture by HPLC: Rt = 0-1-6 min, water/acetonitrile, 30-60-70%, flow 60 mL/min (loading pump 4 mL/min), column: XBridge OBD 30×100, 5 µm. ^{1}H NMR (500 MHz, DMSO-d_6): δ 8.39 (d, *J* = 4.7 Hz, 1H), 8.36 (s, 1H), 7.57 – 7.52 (m, 1H), 7.29 (dd, *J* = 7.8, 4.8 Hz, 1H), 3.92 – 3.75 (m, 2H), 3.29 (s, 1H), 3.12 (dd, *J* = 8.8, 4.0 Hz, 1H), 2.12 (t, *J* = 9.9 Hz, 1H), 1.99 – 1.89 (m, 2H), 1.89 – 1.81 (m, 1H), 1.77 – 1.70 (m, 1H), 1.68 (dd, *J* = 9.5, 6.6 Hz, 1H), 0.85 (t, *J* = 7.1 Hz, 3H) ppm. ^{13}C{^{1}H} NMR (151 MHz, $CDCl_3$): δ 174.7, 147.6 (d, *J* = 14.3 Hz), 137.7, 134.0, 123.1, 60.2, 56.0, 48.5, 46.5, 38.1, 35.9, 33.9, 14.1 ppm. LCMS (M+H): 232. HRMS (ESI-TOF) *m/z*: $[M + H]^+$ calcd for $C_{14}H_{18}NO_2$, 232.1338; found 232.1333.

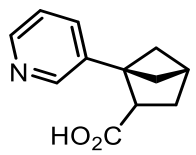

(±)-1-(Pyridin-3-yl)bicyclo[2.1.1]hexane-2-carboxylic acid (22b)

General procedure D was used. Yield: 7.71 g, 0.038 mol, 69%, beige solid, m.p. = 129-130 °C. ^{1}H NMR (500 MHz, DMSO-d_6): δ 11.99 (br s, 1H), 8.41 (d, *J* = 8.6 Hz, 2H), 7.59 (d, *J* = 6.2 Hz, 1H), 7.30 (s, 1H), 3.07 (br s, 1H), 2.49 – 2.32 (m, 1H), 2.15 (t, *J* = 8.7 Hz, 1H), 2.00 (t, *J* = 7.4 Hz, 1H), 1.93 (d, *J* = 8.9 Hz, 1H), 1.86 (br s, 1H), 1.72 (br s, 1H), 1.69 – 1.58 (m, 1H) ppm. ^{13}C{^{1}H} NMR (126 MHz, DMSO-d_6): δ 175.7, 158.6, 147.5, 147.3, 133.7, 123.1, 55.1, 47.0, 46.1, 37.5, 35.0, 33.6 ppm. LCMS (M+H): 204. HRMS (ESI-TOF) *m/z*: $[M + H]^+$ calcd for $C_{12}H_{14}NO_2$, 204.1025; found 204.1019.

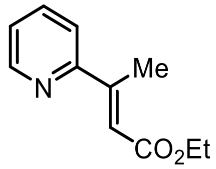

Ethyl-3-(pyridin-2-yl)but-2-enoate

General procedure A was used. The final product was purified by distillation (b.p. = 67-68 °C, 0.1 mmHg). Yield: 55.77 g, 0.292 mol, 73%, colorless oil. ^{1}H NMR (500 MHz, $CDCl_3$): δ 8.63 (d, *J* = 4.6 Hz, 1H), 7.70 (td, *J* = 7.7, 1.4 Hz, 1H), 7.54 (d, *J* = 7.9 Hz, 1H), 7.25 (dd, *J* = 7.6, 5.1 Hz, 1H), 6.68 (d, *J* = 0.7 Hz, 1H), 4.22 (q, *J* = 7.1 Hz, 2H), 2.61 (s, 3H), 1.31 (t, *J* = 7.1 Hz, 3H) ppm. ^{13}C{^{1}H} NMR (126 MHz, $CDCl_3$): δ 167.1, 158.2, 153.1, 149.4, 136.8, 123.6, 121.0, 119.4, 60.1, 16.1, 14.4 ppm. LCMS (M+H): 192. HRMS (ESI-TOF) *m/z*: $[M + H]^+$ calcd for $C_{11}H_{14}NO_2$, 192.1025; found 192.1018.

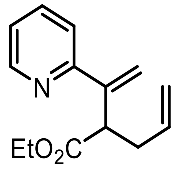

Ethyl 2-(1-(pyridin-2-yl)vinyl)pent-4-enoate (23)

General procedure B was used. Yield: 30.95 g, 0.134 mol, 67%, colorless oil.

An analytically pure sample of the product was obtained by purification of the sample of the crude mixture by column chromatography, SiO_2, hexane/EtOAc, 9:1. ^{1}H NMR (500 MHz, $CDCl_3$): δ 8.55 (d, *J* = 4.3 Hz, 1H), 7.63 (dt, *J* = 7.8, 1.7 Hz, 1H), 7.53 (d, *J* = 8.0 Hz, 1H), 7.15 (dd, *J* = 6.9, 5.3 Hz, 1H), 5.84 (s, 1H), 5.84 – 5.75 (m, 1H), 5.46 (s, 1H), 5.05 (dd, *J* = 17.1, 1.5 Hz, 1H), 4.99 (d, *J* = 10.1 Hz, 1H), 4.12 (qd, *J* = 7.1, 1.3 Hz, 2H), 4.05 (t, *J* = 7.4 Hz, 1H), 2.77 – 2.63 (m, 1H), 2.58 – 2.42 (m, 1H), 1.16 (t, *J* = 7.1 Hz, 3H) ppm. ^{13}C{^{1}H} NMR (126 MHz, $CDCl_3$): δ 173.8, 157.5, 148.8, 146.0, 136.4, 136.0, 122.4, 120.6, 116.8, 116.6, 60.6, 48.0, 35.8, 14.2 ppm. LCMS (M+H): 232. HRMS (ESI-TOF) *m/z*: $[M + H]^+$ calcd for $C_{14}H_{18}NO_2$, 232.1338; found 232.1330.

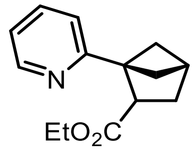

(±)-Ethyl 1-(pyridin-2-yl)bicyclo[2.1.1]hexane-2-carboxylate (23a)

General procedure C was used. The final product was purified by distillation (b.p. = 95-94 °C, 0.1 mmHg). Yield: 16.40 g, purity ~90%, 0.071 mol, 71%, colorless oil.

An analytically pure sample of the product was obtained by purification of the sample of the crude mixture by HPLC: Rt = 0-1-5 min, water/acetonitrile, 30-30-70%, flow 60 mL/min (loading pump 4 mL/min), column: XBridge OBD 30×100, 5 µm. ^{1}H NMR (500 MHz, $CDCl_3$): δ 8.52 (d, *J* = 4.2 Hz, 1H), 7.57 (td, *J* = 7.7, 1.7 Hz, 1H), 7.14 (d, *J* = 7.8 Hz, 1H), 7.12 – 7.04 (m, 1H), 3.99 – 3.85 (m, 2H), 3.27 – 3.18 (m, 1H), 2.52 (d, *J* = 1.2 Hz, 1H), 2.18 (t, *J* = 10.0 Hz, 1H), 2.11 – 2.05 (m, 2H), 1.94 – 1.84 (m, 2H), 1.69 (dd, *J* = 9.7, 6.8 Hz, 1H), 0.96 (t, *J* = 7.1 Hz, 3H) ppm. ^{13}C{^{1}H} NMR (126 MHz, $CDCl_3$): δ 175.1, 161.5, 149.1, 136.0, 121.4, 121.0, 60.0, 59.4, 47.5, 46.3, 38.1, 35.3, 33.7, 14.1 ppm. LCMS (M+H): 232. HRMS (ESI-TOF) *m/z*: $[M + H]^+$ calcd for $C_{14}H_{18}NO_2$, 232.1338; found 232.1330.

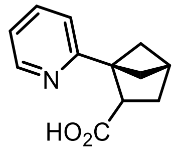

(±)-1-(Pyridin-2-yl)bicyclo[2.1.1]hexane-2-carboxylic acid (23b)

General procedure D was used. Yield: 7.70 g, 0.038 mol, 69%, beige solid, m.p. = 107-108 °C. ^{1}H NMR (500 MHz, DMSO-d$_6$): δ 11.96 (br s, 1H), 8.46 (d, *J* = 2.5 Hz, 1H), 7.69 (dd, *J* = 10.8, 4.4 Hz, 1H), 7.26 (d, *J* = 7.6 Hz, 1H), 7.23 – 7.12 (m, 1H), 3.16 (dd, *J* = 8.6, 3.5 Hz, 1H), 2.45 (s, 1H), 2.15 (t, *J* = 9.8 Hz, 1H), 2.02 – 1.81 (m, 3H), 1.79 – 1.72 (m, 1H), 1.55 (s, 1H) ppm. ^{13}C{^{1}H} NMR (126 MHz, DMSO-d$_6$): δ 175.9, 160.8, 148.5, 136.1, 121.4, 121.0, 58.7, 46.3, 45.9, 37.7, 34.5, 33.6 ppm. LCMS (M+H): 204. HRMS (ESI-TOF) *m/z*: [M - H]$^-$ calcd for $C_{12}H_{12}NO_2$, 202.0868; found 202.0869.

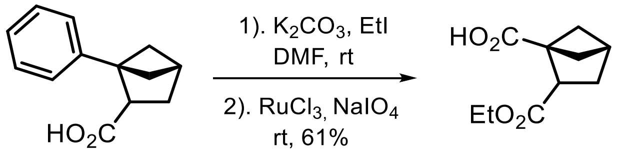

1b **24**

2-(Ethoxycarbonyl)bicyclo[2.1.1]hexane-1-carboxylic acid (24)

To a solution of 1-phenylbicyclo[2.1.1]hexane-2-carboxylic acid (**1b**) (20.20 g, 0.10 mol, 1.00 equiv) and K_2CO_3 (27.60 g, 0.20 mol, 2.00 equiv) in 200 mL of DMF was added EtI (46.80 g, 0.30 mol, 3.00 equiv) dropwise at 0 °C over 15 min. The resulting mixture was stirred overnight at room temperature. The solution was diluted with water (400 mL) and extracted with EtOAc (3 × 200 mL). The combined layers were washed with water (1 × 200 mL), brine (1 × 200 mL), dried over Na_2SO_4, filtered and concentrated under reduced pressure. The crude product was used without purification. The residue was dissolved in a mixture of H_2O (90 mL), CH_3CN (60 mL) and CH_2Cl_2 (60 mL). $RuCl_3$ × H_2O (0.62 g, 0.003 mol, 0.03 equiv) and NaOH (16.00 g, 0.40 mol, 4.00 equiv) were added to the mixture. Then $NaIO_4$ (64.20 g, 0.30 mol, 3.00 equiv) was added in portions at 0 °C. The mixture was vigorously stirred overnight at room temperature. Then the mixture was filtered and washed with water. The layers were partitioned. An aqueous layer was washed with MeO*t*Bu (2 × 100 mL). The aqueous layer was acidified with 5M HCl to pH = 2 and extracted with EtOAc (4 × 100 mL). The combined organic phases were dried over Na_2SO_4, filtered and concentrated under reduced pressure to give the desired product. Yield over 2 steps: 12.08 g, 0.061 mol, 61%, yellow oil. ^{1}H NMR (500 MHz, CDCl$_3$): δ 9.90 (br s, 1H), 4.22 – 4.08 (m, 2H), 3.17 – 3.13 (m, 1H), 2.44 (s, 1H), 2.17 (t, *J* = 10.3 Hz, 1H), 2.03 – 1.89 (m, 3H), 1.66 (dd, *J* = 9.7, 7.4 Hz, 1H), 1.48 (dd, *J* = 9.7, 6.8 Hz, 1H), 1.24 (t, *J* = 7.1 Hz, 3H) ppm. ^{13}C{^{1}H} NMR (126 MHz, CDCl$_3$): δ 178.0, 174.5, 60.9, 54.8, 45.1, 44.8, 38.2, 35.6, 33.1, 14.2 ppm. HRMS (ESI-TOF) *m/z*: [M + H]$^+$ calcd for $C_{10}H_{15}O_4$, 199.0970; found 199.0963.

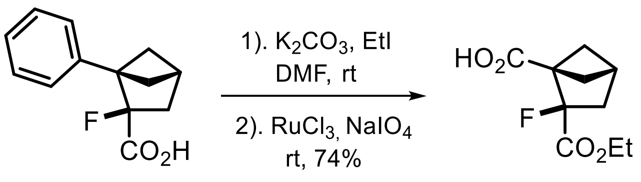

(±)-2-(Ethoxycarbonyl)-2-fluorobicyclo[2.1.1]hexane-1-carboxylic acid (25)

The same procedure as for **24** was used. Yield: 7.93 g, 0.0367 mol, 74%, yellow oil. ^{1}H NMR (500 MHz, $CDCl_3$): δ 9.98 (br s, 1H), 4.46 – 4.23 (m, 2H), 2.54 – 2.41 (m, 2H), 2.31 – 1.99 (m, 5H), 1.31 (t, *J* = 7.1 Hz, 3H) ppm. ^{13}C{^{1}H} NMR (126 MHz, $CDCl_3$): δ 175.3, 170.7 (d, *J* = 28.6 Hz), 98.4 (d, *J* = 205.2 Hz), 62.1, 60.9 (d, *J* = 23.0 Hz), 44.0, 42.6 (d, *J* = 21.0 Hz), 41.1 (d, *J* = 3.8 Hz), 33.6, 14.1 ppm. ^{19}F{^{1}H} NMR (376 MHz, $CDCl_3$): δ -158.1 (s) ppm. HRMS (ESI-TOF) *m/z*: [M + H]$^+$ calcd for $C_{10}H_{14}FO_4$, 217.0876; found 217.0869.

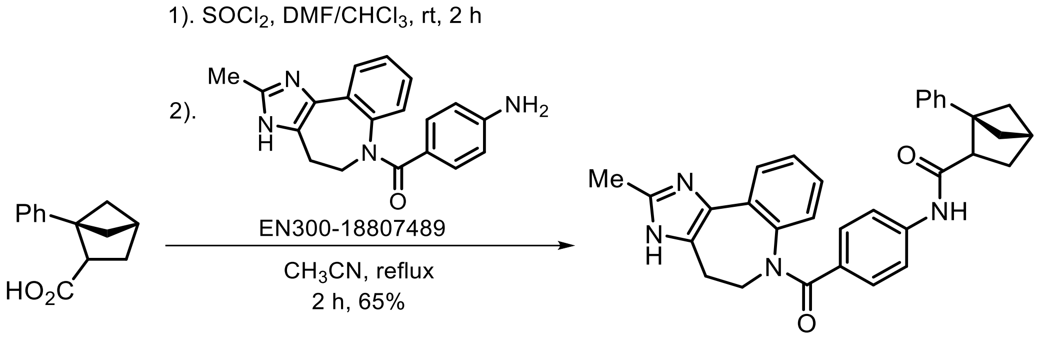

***N*-(4-(2-methyl-3,4,5,6-tetrahydrobenzo[b]imidazo[4,5-d]azepine-6-carbonyl)phenyl)-1-phenylbicyclo[2.1.1]hexane-2-carboxamide ((±)-26)**

DMF (1 drop) and thionyl chloride (0.59 g, 4.90 mmol, 1.96 equiv) were added to a solution of 1-phenylbicyclo[2.1.1]hexane-2-carboxylic acid (**1b**) (0.50 g, 2.50 mmol, 1.00 equiv) in $CHCl_3$ (3 mL) at room temperature. The resulting mixture was stirred for 2 h at this temperature, then concentrated in *vacuo*. The resulting residue was diluted with $CHCl_3$ (3 mL) and concentrated again. CH_3CN (5 mL) was added to the residue, and the mixture was poured into a suspension of (4-aminophenyl)(2-methyl-4,5-dihydrobenzo[*b*]imidazo[4,5-d]azepin-6(1*H*)-yl)methanone (EN300-18807489) (0.71 g, 2.20 mmol, 0.88 equiv) and pyridine (0.59 g, 7.40 mmol, 2.96 equiv) in CH_3CN (10 mL) at room temperature. The mixture was heated at reflux for 2 h, and then cooled to room temperature. The solvent was evaporated under reduced pressure. The final product was purified by HPLC: Rt = 0-1-5 min, water/acetonitrile/0.1%NH_4OH, 35-35-60%, flow 30 mL/min (loading pump 4 mL/min), column: XBridge BEH C18, 100 × 19 mm, 5 μm. Yield: 0.72 g, 1.43 mmol, 65%, white solid. ^{1}H NMR (500 MHz, DMSO-d_6): δ 11.91 (s, 1H), 9.65 (s, 1H), 8.10 (d, *J* = 7.6 Hz, 1H), 7.22 – 6.93 (m, 7H), 6.91 – 6.54 (m, 4H), 4.93 (d, *J* = 11.3 Hz, 1H), 3.17 (d, *J* = 5.2 Hz, 1H), 3.16 – 2.74 (m, 4H), 2.45 (s, 1H), 2.40 – 2.19 (m, 4H), 2.09 (t, *J* = 9.4 Hz, 1H), 1.93 (d, *J* = 9.5 Hz, 1H),

1.70 (br s, 2H), 1.61 – 1.53 (m, 1H) ppm. $^{13}C\{^1H\}$ NMR (151 MHz, DMSO-d$_6$): δ 161.0, 150.7, 150.6, 149.0, 144.3, 139.20, 139.17, 137.9, 136.3, 132.5, 116.0, 111.2, 110.94, 110.91, 110.83, 110.80, 109.6, 108.1, 57.0, 52.1, 44.3, 37.8, 36.7, 34.1 ppm. LCMS (M+H): 503. HRMS (ESI-TOF) *m/z*: $[M + H]^+$ calcd for $C_{32}H_{31}N_4O_2$, 503.2447; found 503.2448.

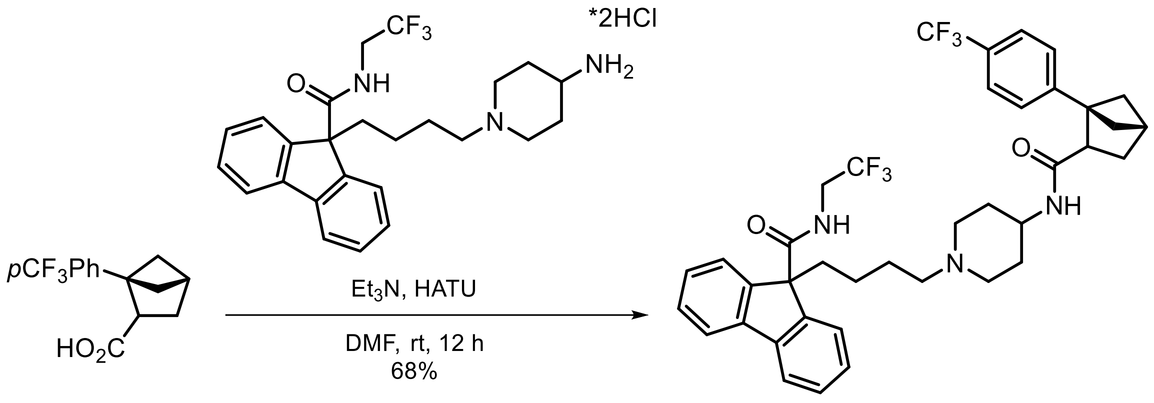

8b (±)-**27**

***N*-(2,2,2-Trifluoroethyl)-9-(4-(4-(1-(4-(trifluoromethyl)phenyl)bicyclo[2.1.1]hexane-2-carboxamido)piperidin-1-yl)butyl)-9*H*-fluorene-9-carboxamide ((±)-27)**

1-(4-(Trifluoromethyl)phenyl)bicyclo[2.1.1]hexane-2-carboxylic acid **8b** (0.20 g, 0.40 mmol, 1.00 equiv), 9-(4-(4-aminopiperidin-1-yl)butyl)-*N*-(2,2,2-trifluoroethyl)-9*H*-fluorene-9-carboxamide dihydrochloride (0.12 g, 0.44 mmol, 1.10 equiv) and Et_3N (0.27 g, 2.70 mmol, 6.75 equiv) were dissolved in DMF (2 mL). The mixture was cooled to 0 °C and HATU (0.21 g, 0.50 mmol, 1.25 equiv) was added. The resulting mixture was stirred for 12 h at room temperature. The solution was poured in 10 mL (5% aq.) citric acid and extracted with MeO*t*Bu (3 × 10 mL). The combined organic layers were dried over Na_2SO_4, filtered and concentrated under reduced pressure. The final product was purified by HPLC: Rt = 0-1-5 min, water/acetonitrile/0.1%NH_4OH, 55-55-90%, flow 30 mL/min (loading pump 4 mL/min), column: XBridge BEH C18, 100 × 19 mm, 5 μm. Yield: 0.19 g, 0.27 mmol, 68%, white solid. ^1H NMR (500 MHz, DMSO-d$_6$): δ 7.88 (d, *J* = 7.5 Hz, 2H), 7.60 (d, *J* = 7.9 Hz, 2H), 7.47 – 7.26 (m, 9H), 7.23 (t, *J* = 6.1 Hz, 1H), 3.74 – 3.64 (m, 2H), 2.82 (br s, 1H), 2.44 (br s, 2H), 2.39 – 2.33 (m, 1H), 2.30 – 2.15 (m, 3H), 1.99 – 1.83 (m, 4H), 1.75 – 1.55 (m, 5H), 1.49 – 1.40 (m, 1H), 1.16 – 1.02 (m, 4H), 0.80 – 0.71 (m, 1H), 0.50 (br s, 2H) ppm. $^{13}C\{^1H\}$ NMR (151 MHz, DMSO-d$_6$): δ 172.7, 172.3, 147.1, 145.6, 140.8, 124.6 (d, *J* = 3.5 Hz), 124.0, 123.7 (m), 120.8 (t, *J* = 296.6 Hz), 61.7, 57.7, 57.3, 51.6, 51.4, 48.0, 45.8, 45.4, 37.4, 36.0, 34.4, 32.9, 31.2, 31.1, 26.6, 21.2 ppm. $^{19}F\{^1H\}$ NMR (376 MHz, DMSO-d$_6$): δ -61.3 (s), -71.1 (s) ppm. LCMS (M+H): 698. HRMS (ESI-TOF) *m/z*: $[M + H]^+$ calcd for $C_{39}H_{42}F_6N_3O_2$, 698.3181; found 698.3185.

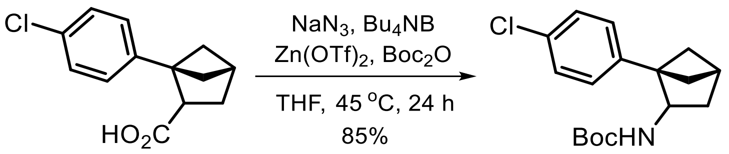

5b **SI-1**

Tert-butyl (1-(4-chlorophenyl)bicyclo[2.1.1]hexan-2-yl)carbamate (SI-1)

To a stirring solution of 1-(4-chlorophenyl)bicyclo[2.1.1]hexane-2-carboxylic acid (**5b**) (1.50 g, 6.30 mmol, 1.00 equiv) in THF (40 mL) was added NaN_3 (1.44 g, 22.2 mmol, 3.50 equiv), followed by tetrabutyl ammonium bromide (Bu_4NBr) (0.31 g, 1.00 mmol) and $Zn(OTf)_2$ (0.12 g, 0.30 mmol), and the reaction mixture was heated to 40 °C. Then Boc_2O (2.07 g, 9.50 mmol, 1.50 equiv) was added at once, and the reaction was heated at 45 °C overnight. The reaction was cooled to 0 °C and was quenched with a 10% aq. solution of $NaHCO_3$ (180 mL). THF was evaporated, and the aqueous layer was extracted with EtOAc (3 × 30 mL). The combined organic layers were washed with a 5% aq. solution of $NaHCO_3$ (20 mL), brine (30 mL), dried over Na_2SO_4, filtered and concentrated to dryness to yield a crude product which which was purified by flash chromatography (SiO_2, gradient, hexanes/EtOAc, 0-90%). Yield: 1.65 g, 5.37 mmol, 85%, beige solid, m.p. = 93-94 °C. ^{1}H NMR (500 MHz, DMSO-d$_6$): δ 7.28 (d, *J* = 8.0 Hz, 2H), 7.14 (d, *J* = 8.0 Hz, 2H), 6.87 (d, *J* = 8.8 Hz, 1H), 4.08 (t, *J* = 6.9 Hz, 1H), 2.36 (s, 1H), 2.20 (t, *J* = 9.5 Hz, 1H), 1.95 – 1.84 (m, 1H), 1.70 – 1.54 (m, 3H), 1.45 (d, *J* = 10.8 Hz, 1H), 1.24 (s, 9H) ppm. ^{13}C{^{1}H} NMR (151 MHz, DMSO-d$_6$): δ 155.1, 141.0, 130.4, 128.0, 127.5, 77.3, 57.1, 53.9, 43.8, 37.8, 36.8, 34.1, 28.1 ppm. HRMS (ESI-TOF) *m/z*: $[M + Na]^+$ calcd for $C_{17}H_{22}ClNNaO_2$, 330.1237; found 330.1233.

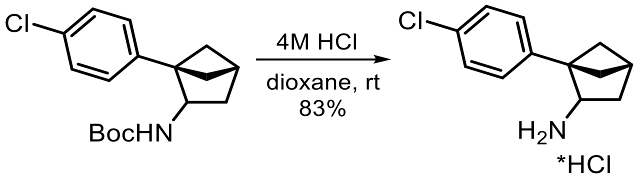

SI-1 **SI-2**

1-(4-Chlorophenyl)bicyclo[2.1.1]hexan-2-amine hydrochloride (SI-2)

Tert-butyl (1-(3,4-dichlorophenyl)bicyclo[2.1.1]hexan-2-yl)carbamate (1.30 g, 4.23 mmol) was dissolved in 4M HCl in dioxane (20 mL). The resulting solution was stirred at 20-25 °C for 12 h, and then Et_2O (20 mL) was added. The suspension was stirred for 1 h, filtered and dried. Yield: 0.86 g, 3.53 mmol, 83%, white solid, m.p. = 260-262 °C. ^{1}H NMR (500 MHz, DMSO-d$_6$): δ 8.11 (br s, 3H), 7.42 (d, *J* = 8.4 Hz, 2H), 7.26 (d, *J* = 8.4 Hz, 2H), 3.75 (br s, 1H), 2.47 (br s, 1H), 2.29 (t, *J* = 9.8 Hz, 1H), 2.13 – 2.06 (m, 1H), 1.86 (d, *J* = 6.6 Hz, 1H), 1.76 – 1.69 (m, 2H), 1.67 (d, *J* = 11.7 Hz, 1H) ppm. ^{13}C{^{1}H} NMR (151 MHz, DMSO-d$_6$): δ 138.4, 131.5, 128.4, 128.3, 56.2, 54.0, 44.6, 37.1, 35.2, 34.7 ppm. LCMS (M+H): 208. HRMS (ESI-TOF) *m/z*: $[M + H]^+$ calcd for $C_{12}H_{15}ClN$, 208.0893; found 208.0884.

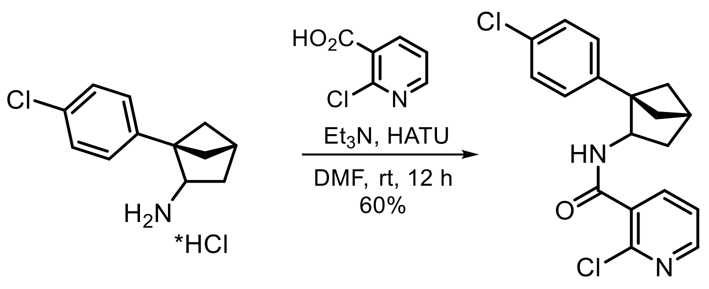

2-Chloro-*N*-(1-(4-chlorophenyl)bicyclo[2.1.1]hexan-2-yl)nicotinamide ((±)28)

2-Chloronicotinic acid (0.39 g, 2.5 mmol, 1.00 equiv), 1-(4-chlorophenyl)bicyclo[2.1.1]hexan-2-amine hydrochloride (0.6 g, 2.5 mmol, 1.00 equiv) and Et_3N (1.24 g, 12.5 mmol, 5.00 equiv) were dissolved in DMF (5 mL). The mixture was cooled to 0 °C, and HATU (1.31 g, 3.4 mmol, 1.36 equiv) was added. The mixture was stirred for 12 h at room temperature. Then the solution was poured in 50 mL (5% aq.) citric acid and extracted with MeO*t*Bu (3 × 30 mL). The combined organic layers were dried over Na_2SO_4, filtered and concentrated under reduced pressure. The crude mixture was purified by HPLC: Rt = 0-1-6 min, water/acetonitrile/0.1%FA, 35-35-80%, flow 30 mL/min (loading pump 4 mL/min), column: Chomatorex 18 SMB100-5T, 100×19 mm, 5 µm. Yield: 0.52 g, 1.50 mmol, 60%, white solid. ^{1}H NMR (500 MHz, DMSO-d$_6$): δ 8.55 (d, *J* = 9.1 Hz, 1H), 8.41 (dd, *J* = 4.7, 1.8 Hz, 1H), 7.60 (dd, *J* = 7.5, 1.8 Hz, 1H), 7.44 (dd, *J* = 7.5, 4.8 Hz, 1H), 7.34 (d, *J* = 8.4 Hz, 2H), 7.25 (d, *J* = 8.4 Hz, 2H), 4.64 (t, *J* = 7.3 Hz, 1H), 2.46 (s, 1H), 2.37 – 2.30 (m, 1H), 1.96 – 1.88 (m, 1H), 1.84 – 1.78 (m, 1H), 1.75 – 1.67 (m, 2H), 1.56 (d, *J* = 11.0 Hz, 1H) ppm. ^{13}C{^{1}H} NMR (151 MHz, DMSO-d$_6$): δ 164.7, 149.9, 146.4, 140.4, 137.7, 133.4, 130.7, 128.3, 127.7, 122.9, 57.2, 52.5, 44.6, 37.8, 37.2, 34.4 ppm. LCMS (M+H): 348. HRMS (ESI-TOF) *m/z*: $[M + H]^+$ calcd for $C_{18}H_{17}Cl_2N_2O$, 347.0718; found 347.0726.

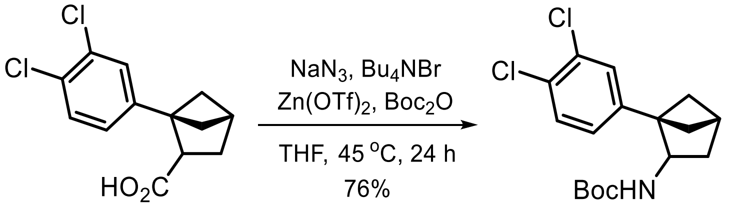

Tert-butyl (1-(3,4-dichlorophenyl)bicyclo[2.1.1]hexan-2-yl)carbamate (SI-3)

The same procedure as for **SI-1** was used. Yield: 1.63 g, 4.78 mmol, 76%, beige solid. ^{1}H NMR (500 MHz, DMSO-d$_6$): δ 7.49 (d, *J* = 8.2 Hz, 1H), 7.32 (s, 1H), 7.11 (d, *J* = 8.2 Hz, 1H), 7.00 (d, *J* = 9.3 Hz, 1H), 4.06 (s, 1H), 2.37 (s, 1H), 2.19 (t, *J* = 9.7 Hz, 1H), 1.91 – 1.86 (m, 1H), 1.70 – 1.60 (m, 3H), 1.45 (d, *J* = 10.8 Hz, 1H), 1.24 (s, 9H) ppm. ^{13}C{^{1}H} NMR (101 MHz, DMSO-d$_6$): δ

155.1, 143.3, 130.3, 129.8, 128.3, 128.2, 126.7, 77.5, 56.9, 54.0, 43.4, 37.9, 36.4, 34.1, 28.1 ppm. HRMS (ESI-TOF) *m/z*: $[M + Na]^+$ calcd for $C_{17}H_{21}Cl_2NNaO_2$, 364.0847; found 364.0840.

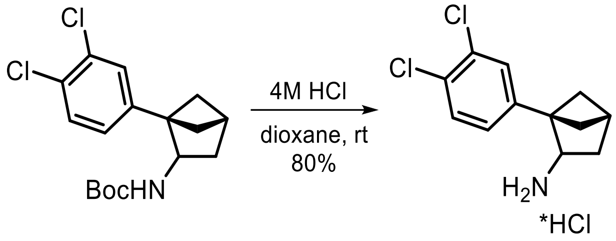

SI-3 **SI-4**

1-(3,4-Dichlorophenyl)bicyclo[2.1.1]hexan-2-amine hydrochloride (SI-4)

The same procedure as for **SI-2** was used. Yield: 0.72 g, 2.59 mmol, 80%, beige solid, m.p. = 244-246 °C. ^{1}H NMR (500 MHz, DMSO-d$_6$): δ 8.17 (br s, 3H), 7.60 (d, *J* = 8.2 Hz, 1H), 7.48 (d, *J* = 1.5 Hz, 1H), 7.20 (dd, *J* = 8.2, 1.6 Hz, 1H), 3.78 (d, *J* = 6.1 Hz, 1H), 2.46 (s, 1H), 2.26 (t, *J* = 9.9 Hz, 1H), 2.14 – 2.03 (m, 1H), 1.87 (d, *J* = 6.6 Hz, 1H), 1.77 – 1.70 (m, 2H), 1.66 (d, *J* = 11.6 Hz, 1H) ppm. ^{13}C{^{1}H} (151 MHz, DMSO-d$_6$): δ 140.6, 131.1, 130.5, 129.5, 128.8, 127.1, 55.9, 54.0, 44.5, 37.0, 35.1, 34.8 ppm. HRMS (ESI-TOF) *m/z*: $[M + H]^+$ calcd for $C_{12}H_{14}Cl_2N$, 242.0503; found 242.0498.

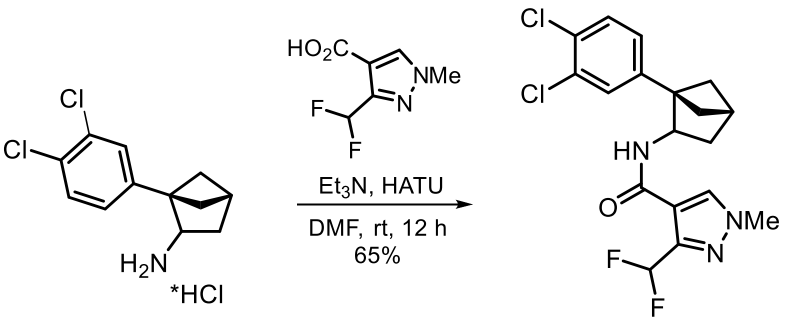

SI-4 **(±)29**

***N*-(1-(3,4-Dichlorophenyl)bicyclo[2.1.1]hexan-2-yl)-3-(difluoromethyl)-1-methyl-1*H*-pyrazole-4-carboxamide ((±)29)**

The same procedure as for **(±)28** was used. The crude mixture was purified by HPLC: Rt = 0-1-6 min, water/MeOH, 50-50-90%, flow 30 mL/min (loading pump 4 mL/min), column: Chomatorex 18 SMB100-5T, 100×19 mm, 5 μm. Yield: 0.66 g, 1.65 mmol, 65%, white solid. ^{1}H NMR (500 MHz, DMSO-d$_6$): δ 8.29 (s, 1H), 7.88 (d, *J* = 8.9 Hz, 1H), 7.47 (d, *J* = 8.3 Hz, 1H), 7.38 (s, 1H), 7.14 (d, *J* = 8.3 Hz, 1H), 7.08 (t, *J* = 54.3 Hz, 1H), 4.62 (t, *J* = 7.4 Hz, 1H), 3.89 (s, 1H), 2.47 (s, 1H), 2.30 (t, *J* = 9.7 Hz, 1H), 2.01 (t, *J* = 7.6 Hz, 1H), 1.86 – 1.80 (m, 1H), 1.79 – 1.73 (m, 1H), 1.70 (dd, *J* = 8.8, 7.2 Hz, 1H), 1.58 (d, *J* = 11.2 Hz, 1H) ppm. ^{13}C{^{1}H} NMR (151 MHz, DMSO-d$_6$): δ 161.0, 144.3 (t, *J* = 23.0 Hz), 142.9, 132.4, 130.5, 129.9, 128.5, 128.3, 126.7, 116.0 (t, *J* = 3.5

Hz), 109.6 (t, *J* = 234.3 Hz), 56.9, 52.1, 44.3, 37.9, 36.9, 34.3 ppm. LCMS (M+H): 401. HRMS (ESI-TOF) *m/z*: $[M + H]^+$ calcd for $C_{18}H_{18}Cl_2F_2N_3O$, 400.0795; found 400.0801.

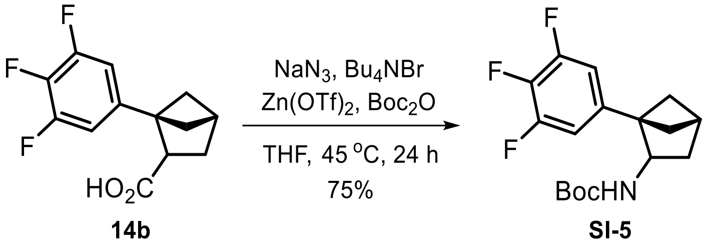

Tert-butyl (1-(3,4,5-trifluorophenyl)bicyclo[2.1.1]hexan-2-yl)carbamate (SI-5)

The same procedure as for **S-1** was used. Yield: 1.55 g, 4.74 mmol, 75%, beige solid. ^1H NMR (500 MHz, DMSO-d$_6$): δ 7.09 – 6.90 (m, 2H), 4.04 (s, 1H), 2.36 (s, 1H), 2.17 (t, *J* = 9.6 Hz, 1H), 1.88 – 1.83 (m, 1H), 1.71 – 1.58 (m, 3H), 1.45 (d, *J* = 9.2 Hz, 1H), 1.25 (s, 9H) ppm. $^{13}C\{^1H\}$ NMR (151 MHz, DMSO-d$_6$): δ 155.2, 149.8 (m), 139.6, 137.01 (m), 110.7 (d, *J* = 16.3 Hz), 77.5, 56.9, 54.0, 43.4, 37.8, 36.2, 33.8, 28.0 ppm. $^{19}F\{^1H\}$ NMR (376 MHz, DMSO-d$_6$): δ -137.33 (d, *J* = 22.2 Hz), -166.47 (t, *J* = 22.1 Hz) ppm. HRMS (ESI-TOF) *m/z*: $[M + Na]^+$ calcd for $C_{17}H_{20}F_3NNaO_2$, 350.1344; found 350.1337.

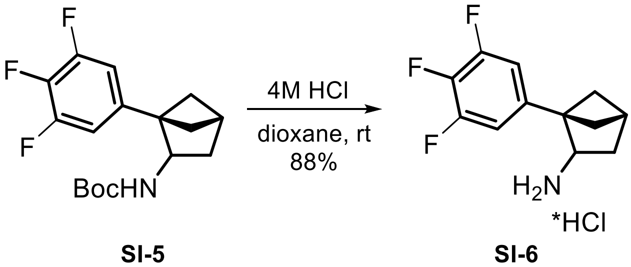

1-(3,4,5-Trifluorophenyl)bicyclo[2.1.1]hexan-2-amine hydrochloride (SI-6)

The same procedure as for **SI-2** was used. Yield: 0.67 g, 2.54 mmol, 88%, white solid. ^1H NMR (500 MHz, DMSO-d$_6$): δ 8.14 (br s, 3H), 7.25 – 7.11 (m, 2H), 3.78 (d, *J* = 6.3 Hz, 1H), 2.45 (s, 1H), 2.25 (t, *J* = 10.0 Hz, 1H), 2.05 (t, *J* = 8.2 Hz, 1H), 1.88 (d, *J* = 7.0 Hz, 1H), 1.75 – 1.67 (m, 2H), 1.64 (d, *J* = 11.7 Hz, 1H) ppm. $^{13}C\{^1H\}$ NMR (151 MHz, DMSO-d$_6$): δ 150.2 (ddd, *J* = 246.8, 9.3, 3.3 Hz), 137.7 (dt, *J* = 231.2, 15.8 Hz), 136.8 (m), 111.6 (dd, *J* = 16.7, 3.6 Hz), 56.0, 54.0, 44.5, 37.0, 35.0, 34.5 ppm. $^{19}F\{^1H\}$ NMR (376 MHz, DMSO-d$_6$): δ -136.2 (d, *J* = 21.6 Hz), -164.9 (t, *J* = 21.6 Hz) ppm. HRMS (ESI-TOF) *m/z*: $[M + H]^+$ calcd for $C_{12}H_{13}F_3N$, 228.1000; found 228.0999.

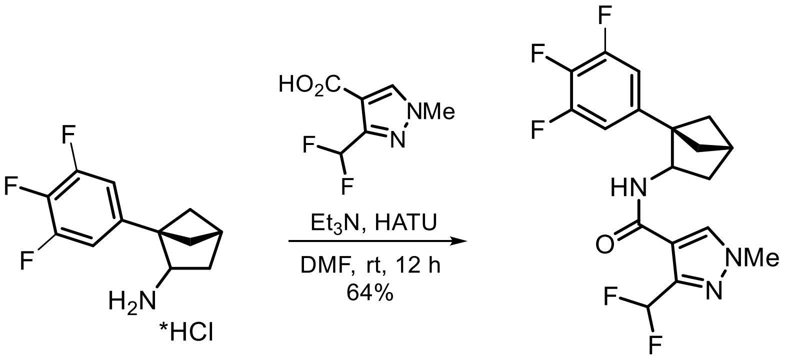

SI-6 **(±)30**

3-(Difluoromethyl)-1-methyl-*N*-(1-(3,4,5-trifluorophenyl)bicyclo[2.1.1]hexan-2-yl)-1*H*-pyrazole-4-carboxamide ((±)30)

The same procedure as for **28** was used. The crude mixture was purified by HPLC: Rt = 0-1-6 min, water/MeOH, 55-55-75%, flow 30 mL/min (loading pump 4 mL/min), column: Chomatorex 18 SMB100-5T, 100×19 mm, 5 µm. Yield: 0.62 g, 1.61 mmol, 64%, white solid. ^{1}H NMR (500 MHz, DMSO-d$_6$): δ 8.28 (s, 1H), 7.87 (d, J = 8.9 Hz, 1H), 7.19 – 6.96 (m, 3H), 4.58 (br s, 1H), 3.87 (s, 3H), 2.44 (s, 1H), 2.26 (t, J = 9.6 Hz, 1H), 1.97 – 1.91 (m, 1H), 1.81 (s, 1H), 1.73 – 1.64 (m, 2H), 1.57 (d, J = 10.6 Hz, 1H) ppm. ^{13}C{^{1}H} NMR (151 MHz, DMSO-d$_6$): δ 173.2, 143.5, 142.0, 131.7, 130.7, 130.3, 128.7, 127.9, 126.5, 126.3, 126.1, 125.8, 117.9, 58.2, 48.7, 46.1, 37.7, 34.3, 34.0, 13.8 ppm. ^{19}F{^{1}H} NMR (376 MHz, DMSO-d$_6$): δ -114.5 (d, J = 16.1 Hz), -136.9 (d, J = 22.3 Hz), -165.9 (t, J = 22.3 Hz) ppm. LCMS (M+H): 386. HRMS (ESI-TOF) *m/z*: [M + H]$^+$ calcd for $C_{18}H_{17}F_5N_3O$, 386.1292; found 386.1295.

Copies of ^{1}H, $^{13}C\{^{1}H\}$ and $^{19}F\{^{1}H\}$ spectra

Ethyl-3-phenylbut-2-enoate (2)

^{1}H NMR (500 MHz, $CDCl_3$)

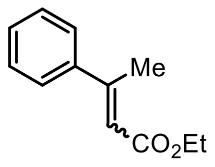

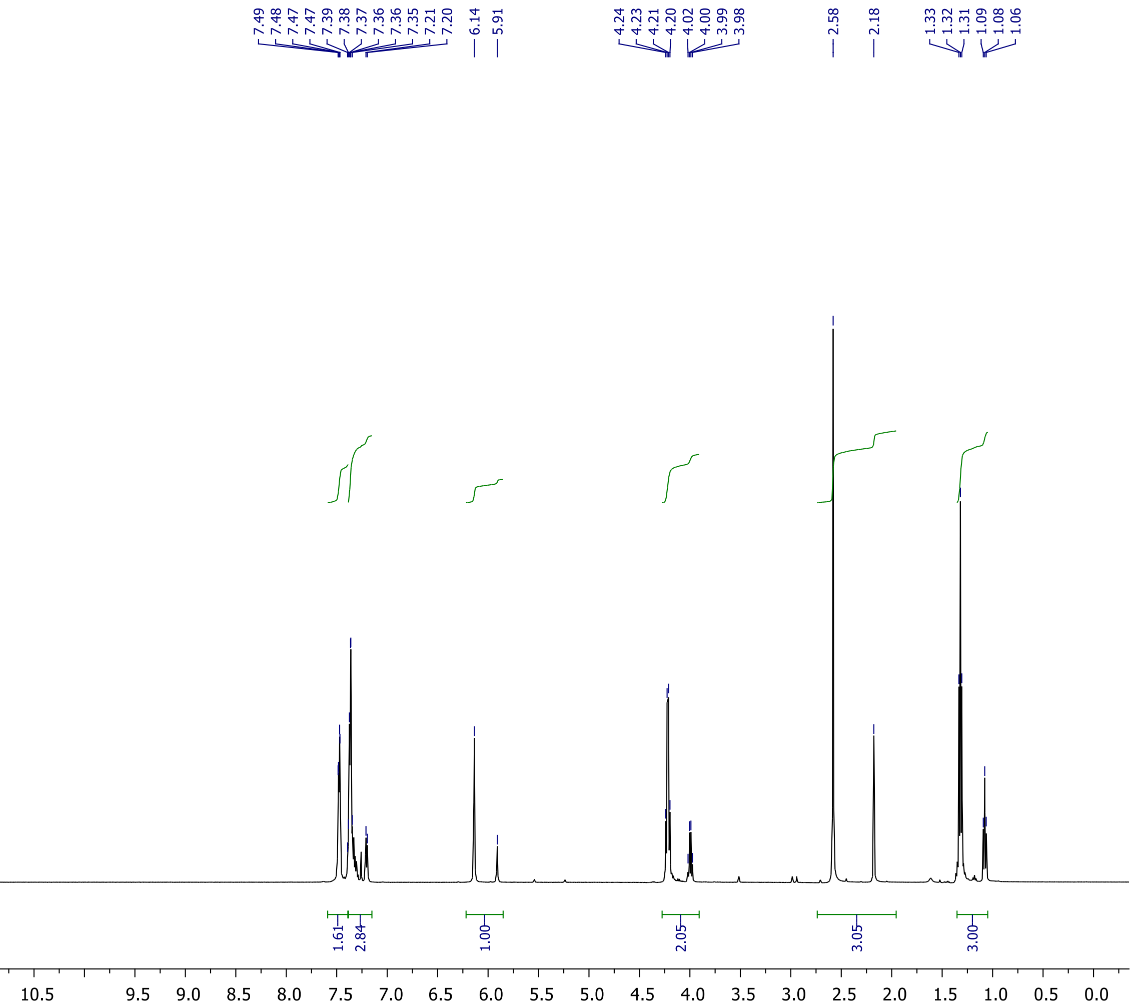

$^{13}C\{^1H\}$ NMR (101 MHz, $CDCl_3$)

R3069160_C13

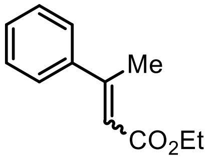

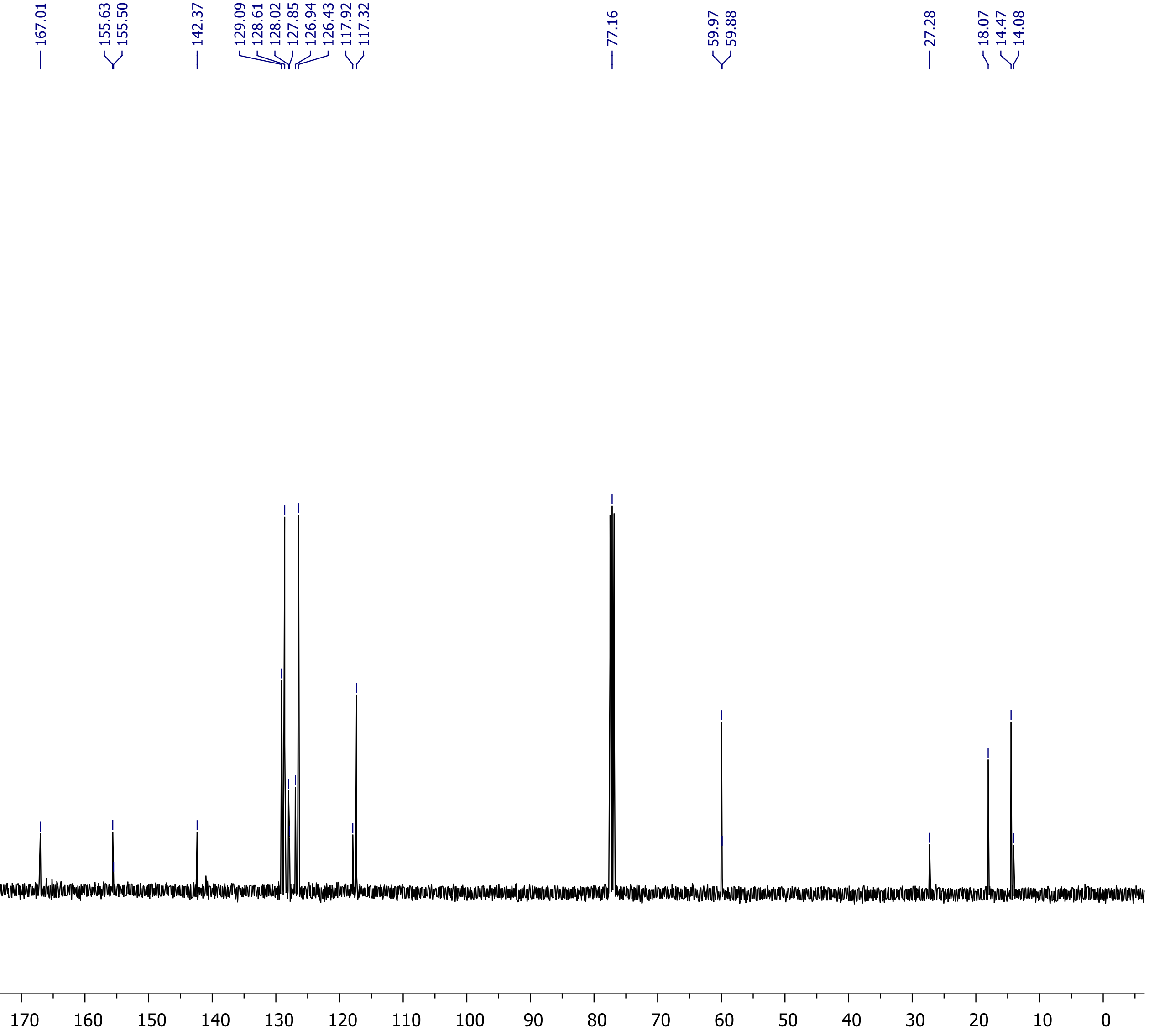

Compound 1

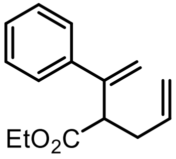

^{1}H NMR (500 MHz, $CDCl_3$)

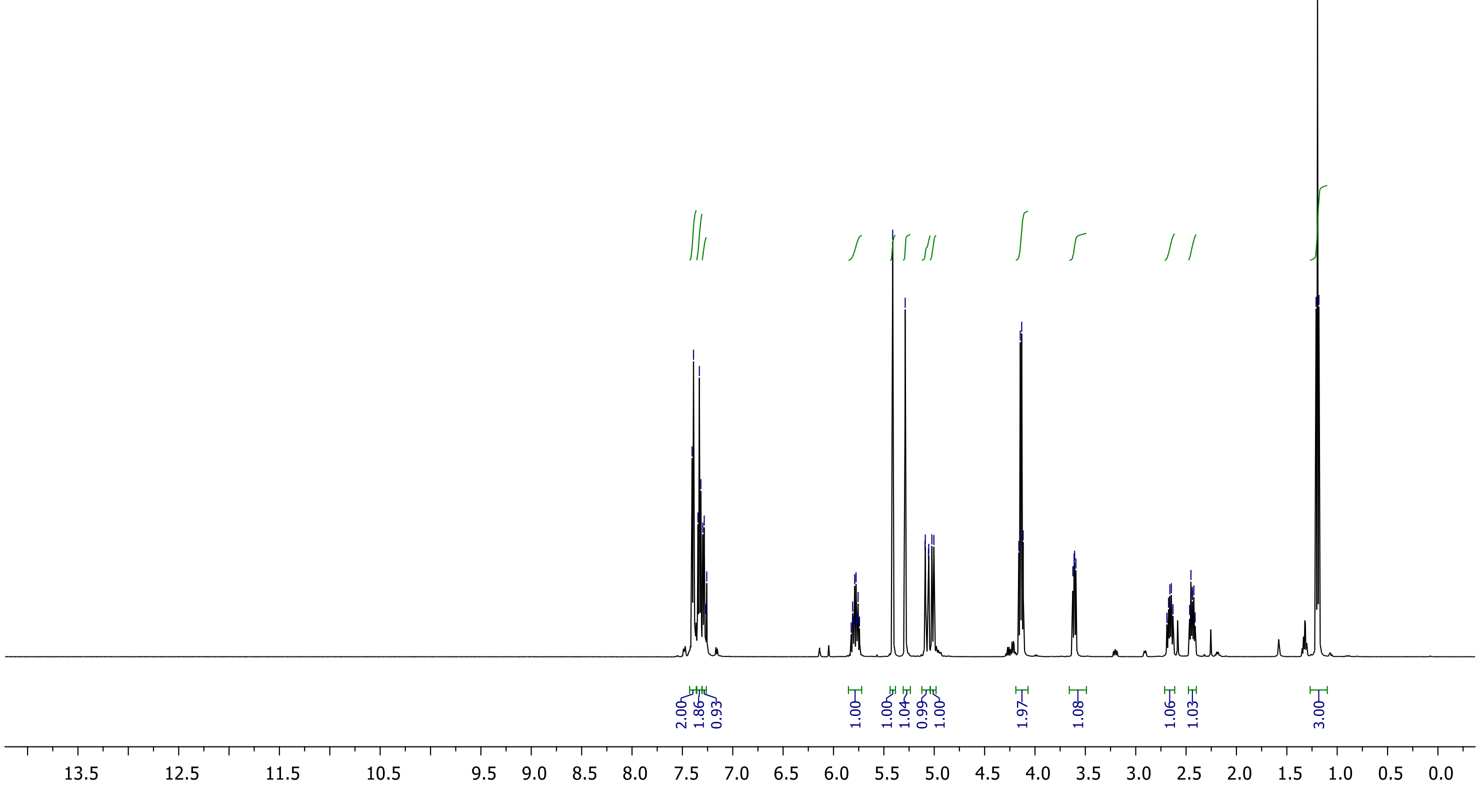

$^{13}C\{^1H\}$ NMR (151 MHz, $CDCl_3$)

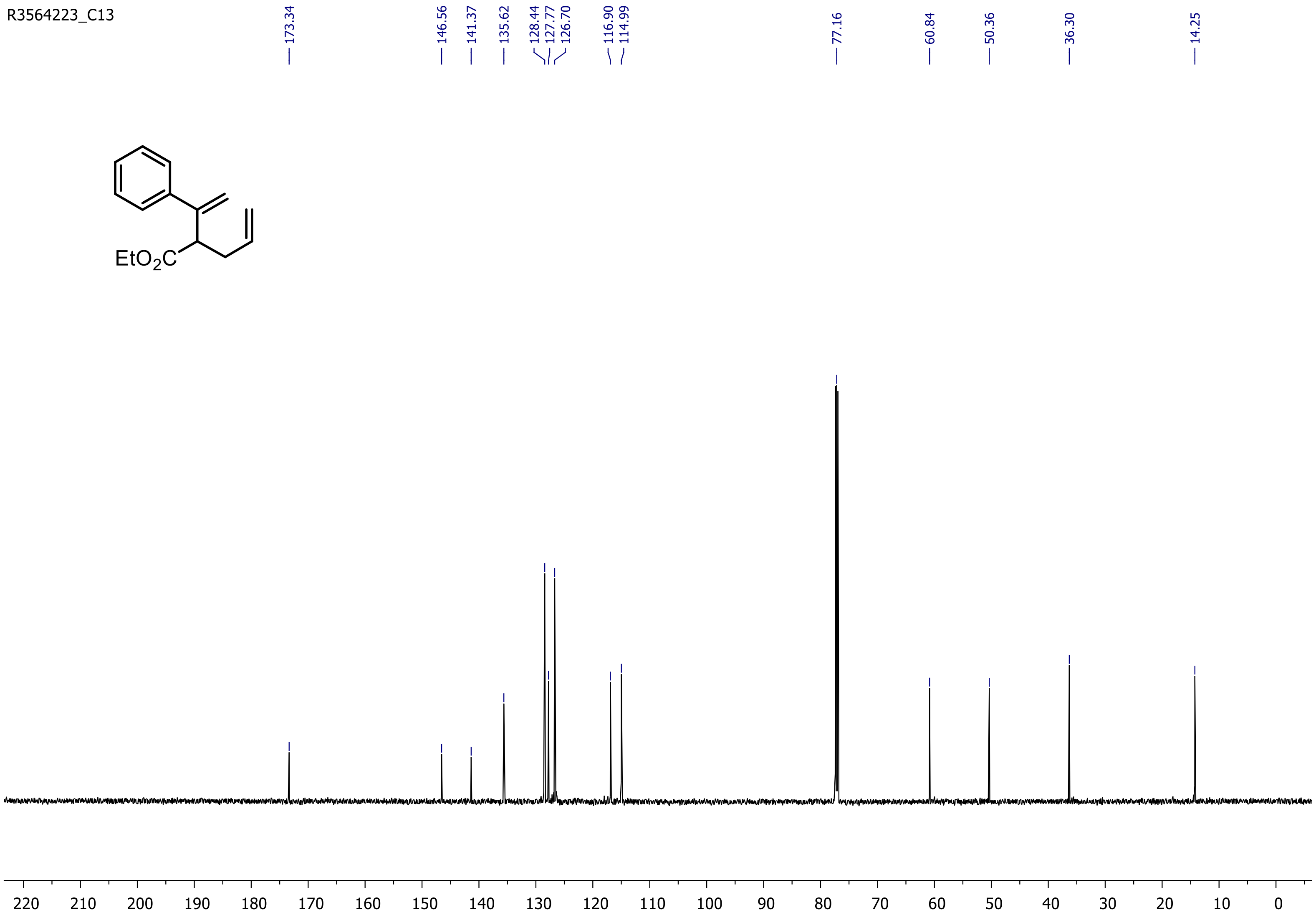

Compound (±)-1a

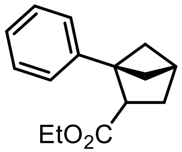

EtO2C

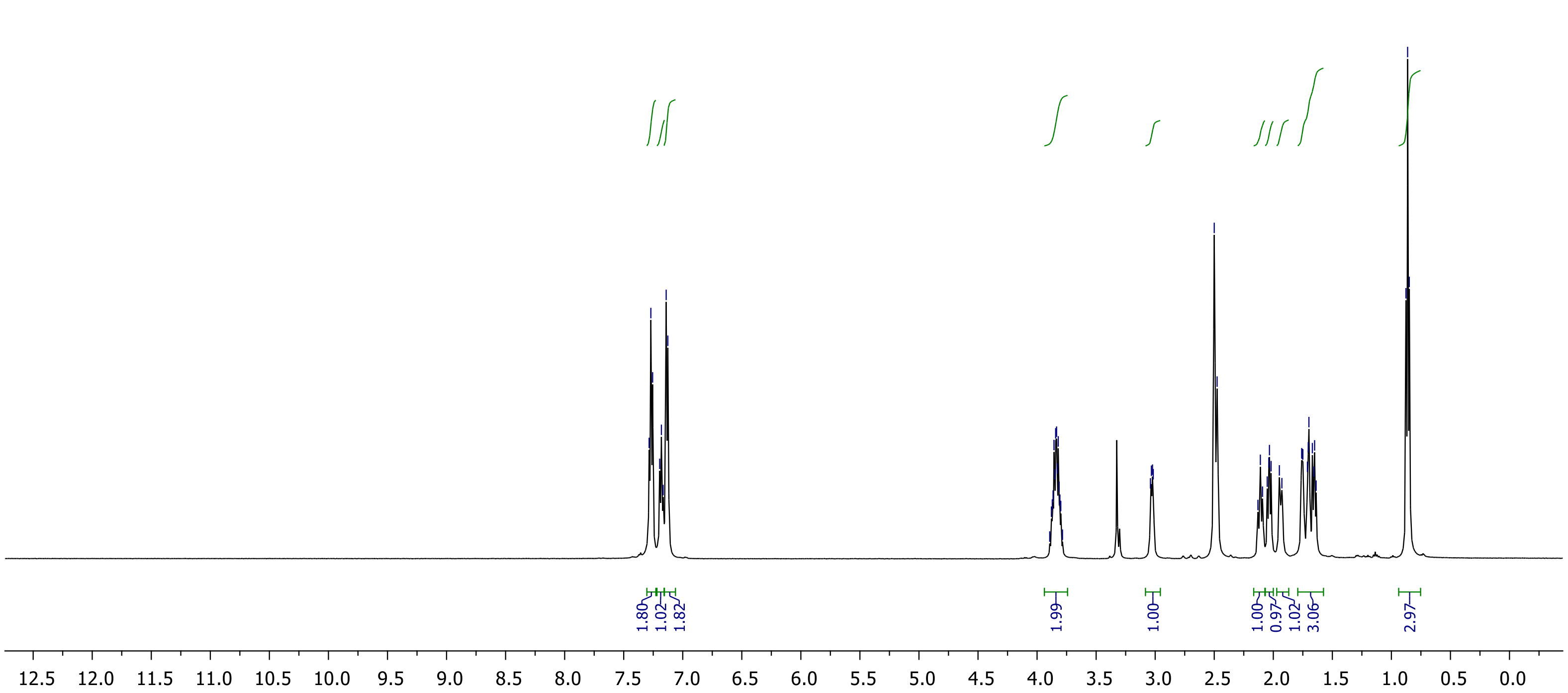

1H NMR (500 MHz, DMSO-d6)
R3073569
7.28 7.27 7.26 7.20 7.18 7.17 7.14 7.13
3.89 3.88 3.87 3.86 3.85 3.84 3.83 3.83 3.82 3.81 3.81 3.80 3.78
3.04 3.03 3.02 3.02
2.50 2.48
2.03 1.76 1.75 1.70 1.70 1.67 1.65
0.88 0.86 0.85
1.80 1.02 1.82 1.99 1.00 1.00 0.97 1.02 3.06 2.97
12.5 12.0 11.5 11.0 10.5 10.0 9.5 9.0 8.5 8.0 7.5 7.0 6.5 6.0 5.5 5.0 4.5 4.0 3.5 3.0 2.5 2.0 1.5 1.0 0.5 0.0

$^{13}C\{^{1}H\}$ NMR (126 MHz, DMSO-d_6)

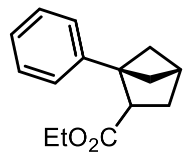

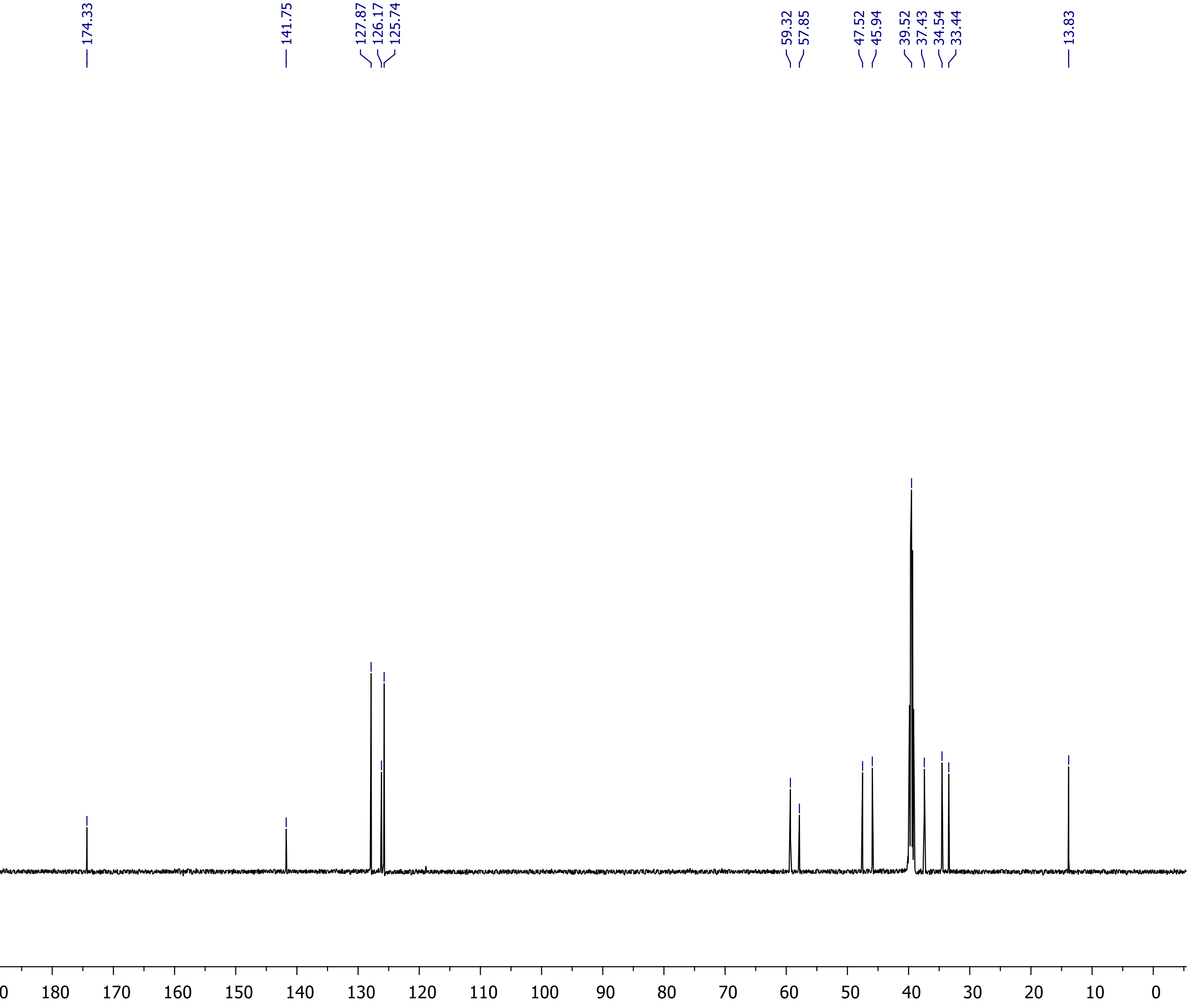

Compound (±)-1b

^{1}H NMR (500 MHz, DMSO-d_6)

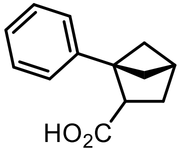

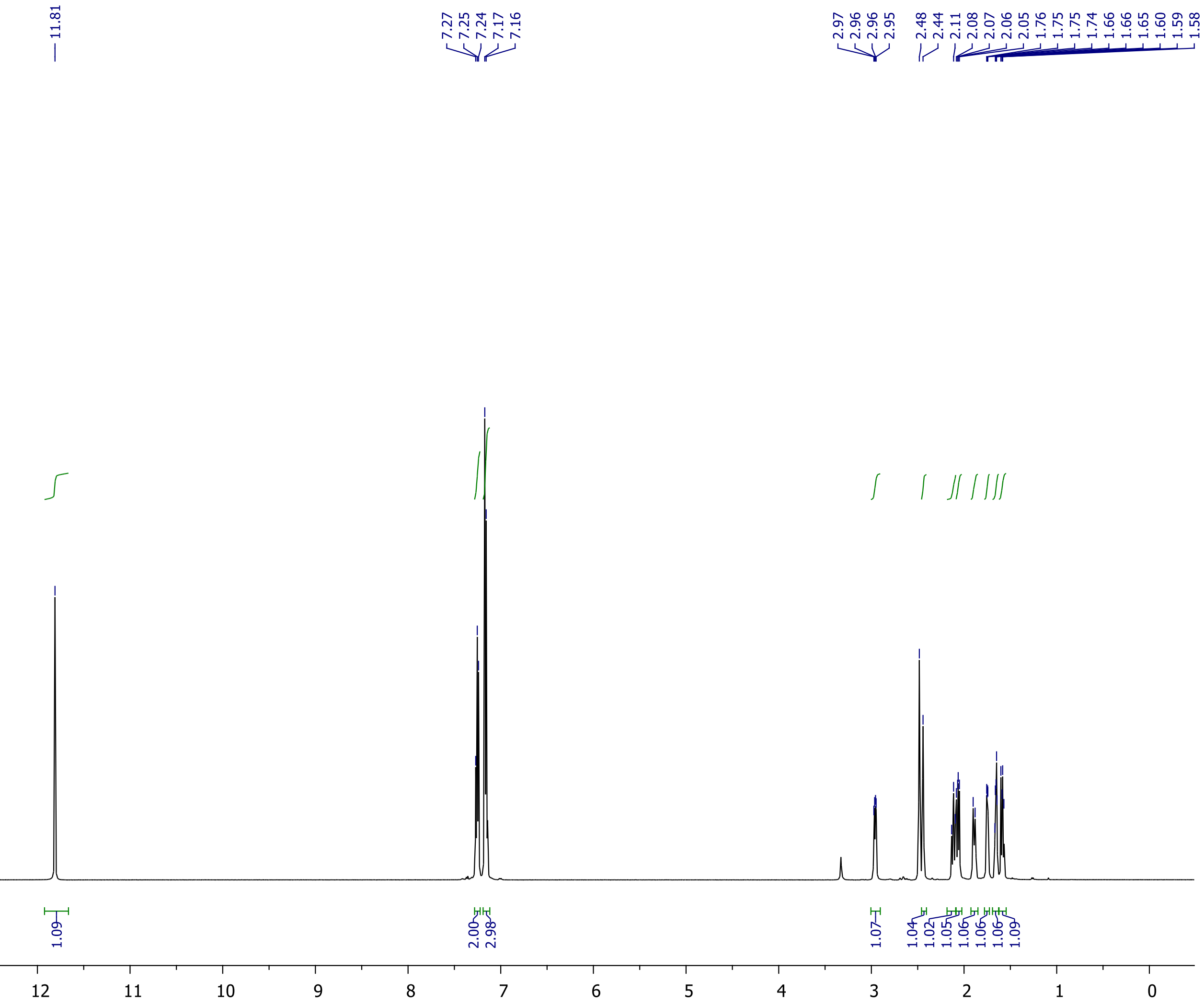

$^{13}C\{^{1}H\}$ NMR (126 MHz, DMSO-d$_6$)

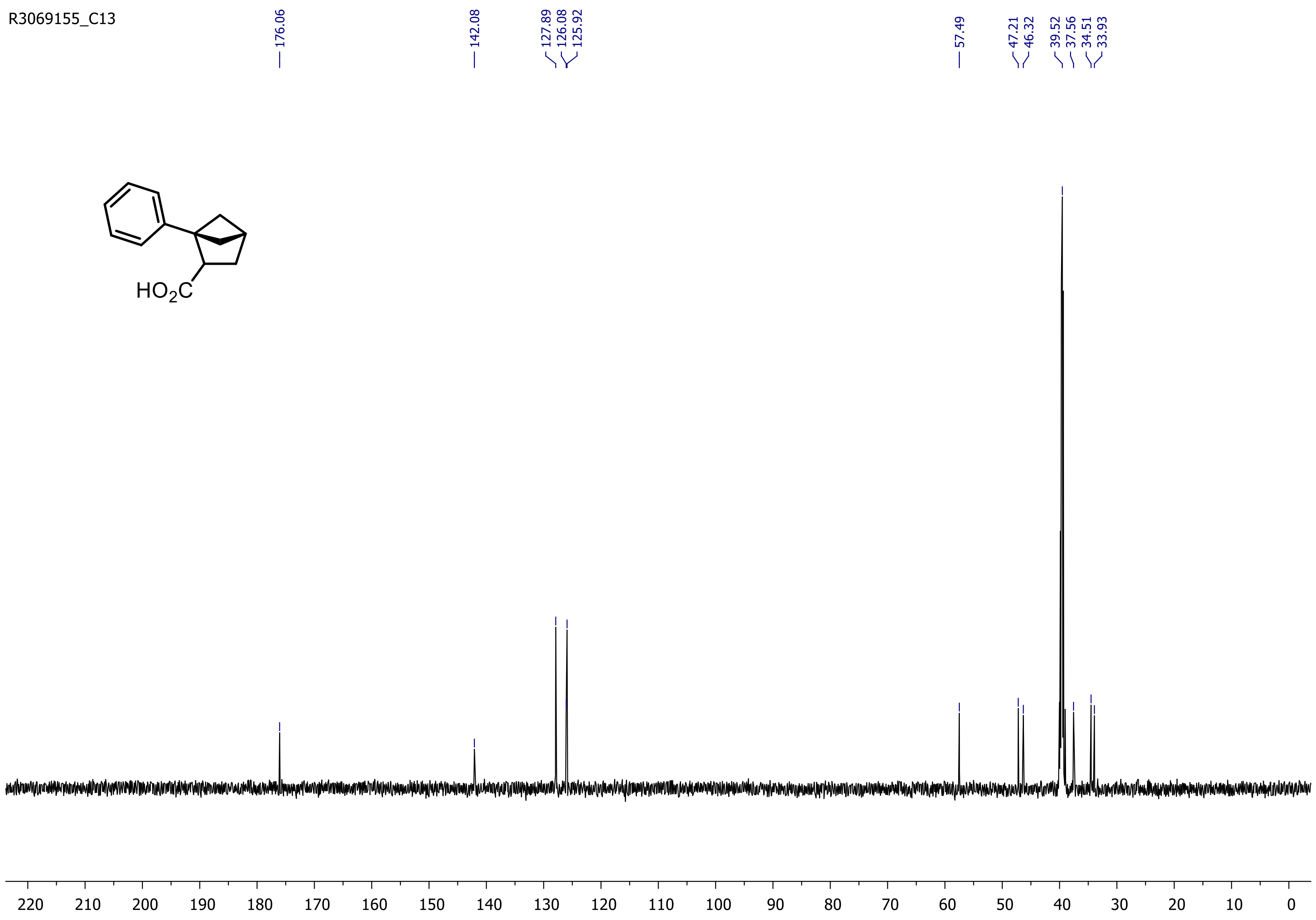

Ethyl-2-fluoro-3-phenylbut-2-enoate

^{1}H NMR (500 MHz, $CDCl_3$)

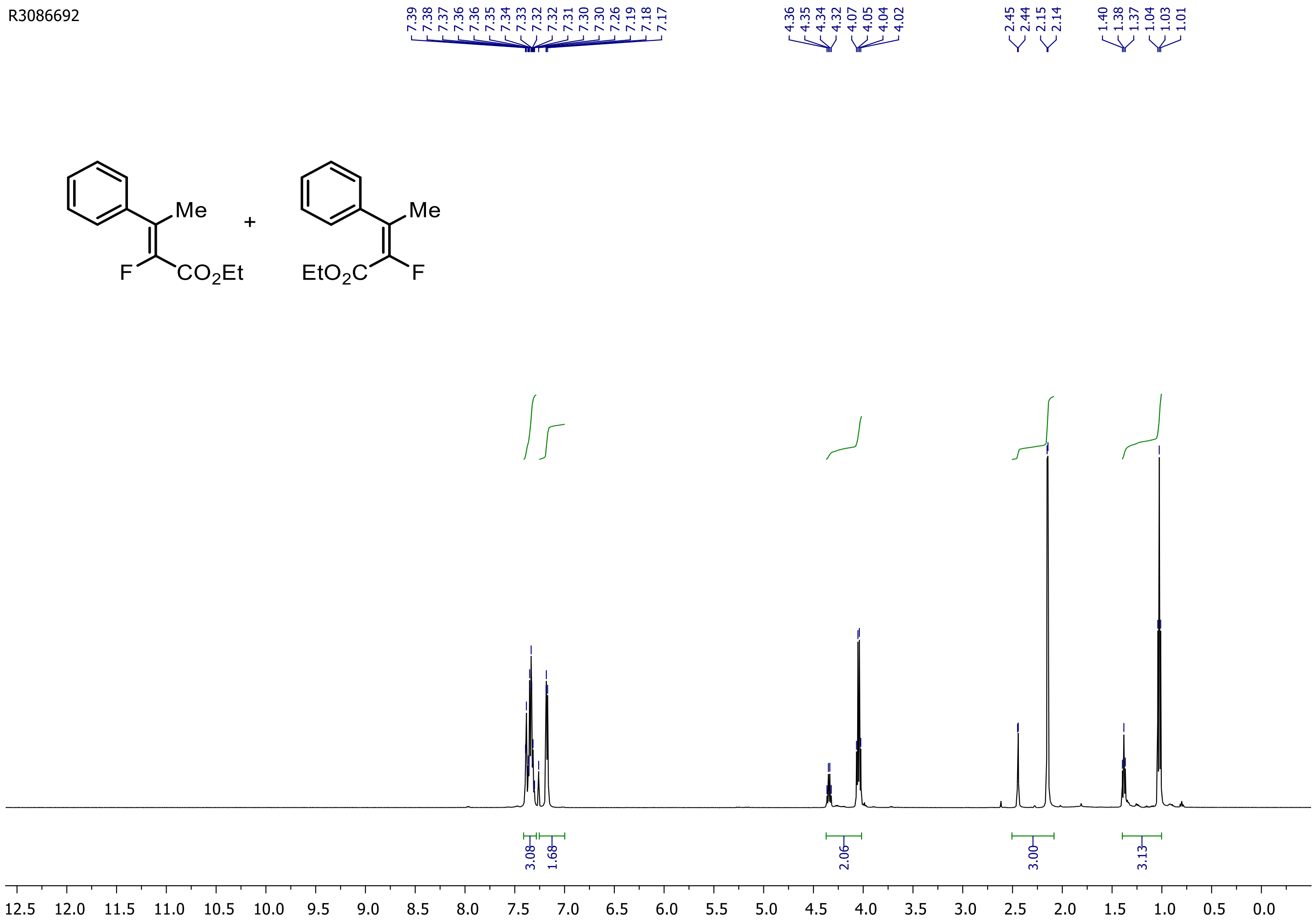

$^{13}C\{^1H\}$ NMR (151 MHz, $CDCl_3$)

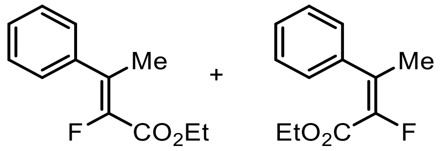

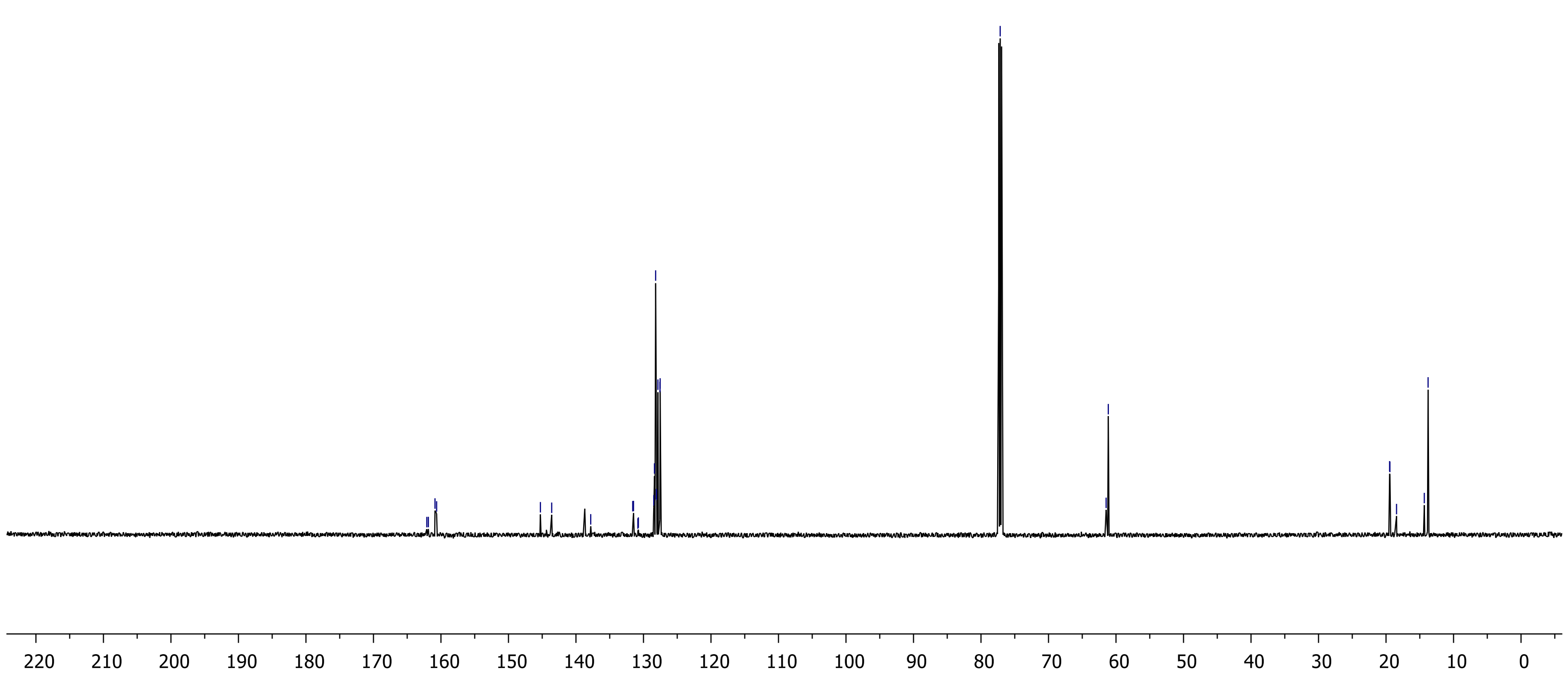

$^{19}F\{^1H\}$ NMR (376 MHz, $CDCl_3$)

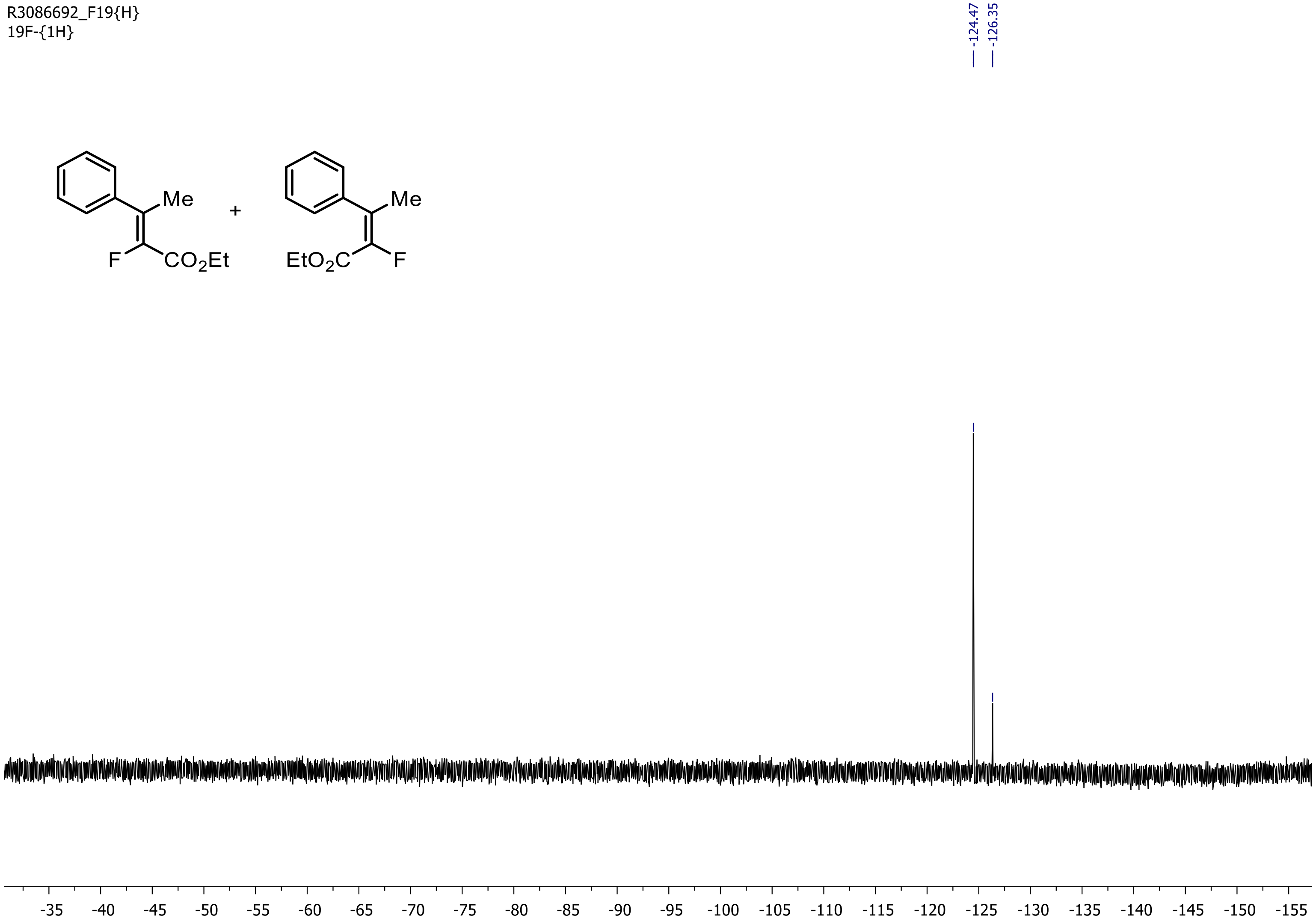

Compound 3

^{1}H NMR (500 MHz, $CDCl_3$)

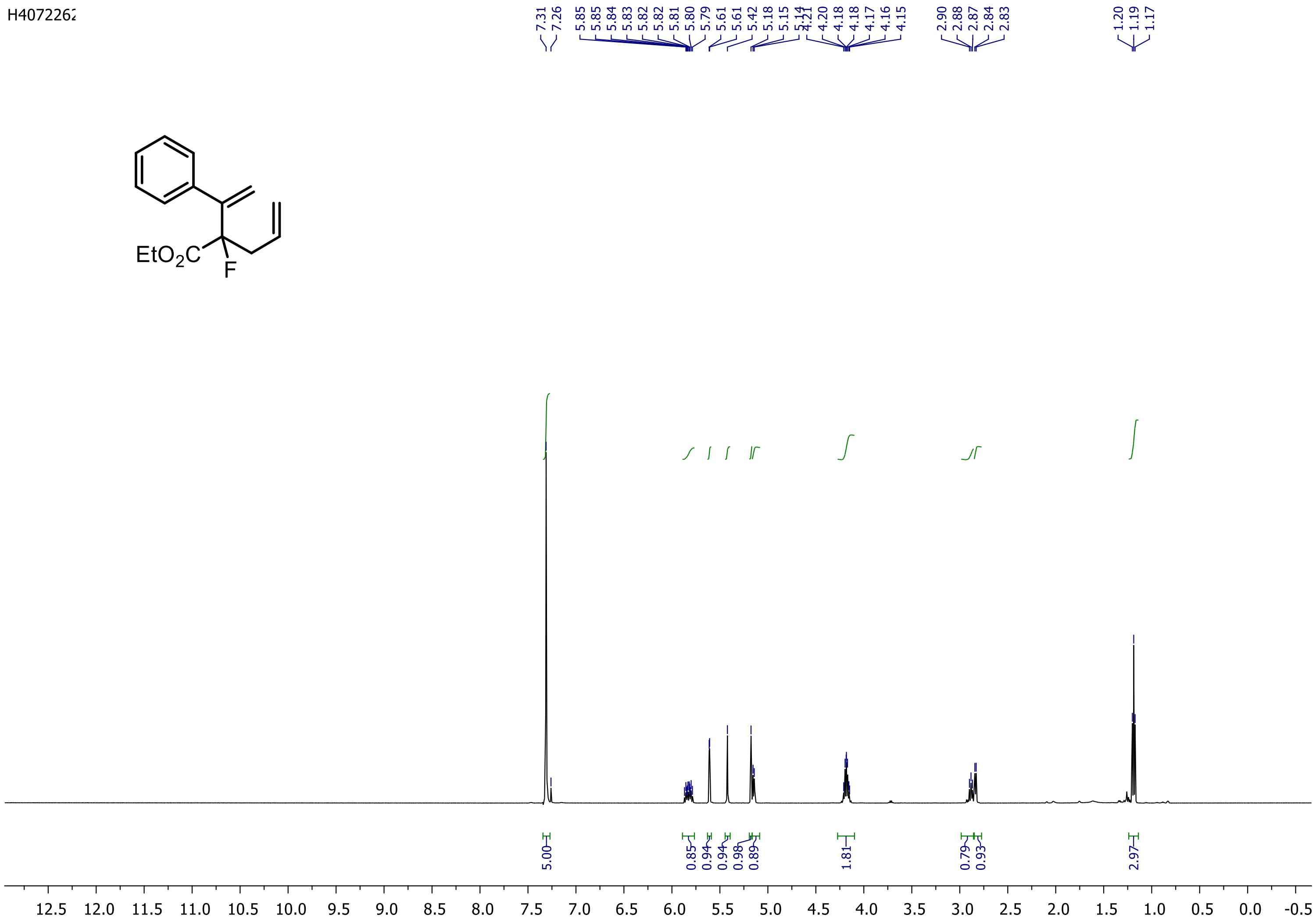

$^{13}C\{^{1}H\}$ NMR (126 MHz, $CDCl_3$)

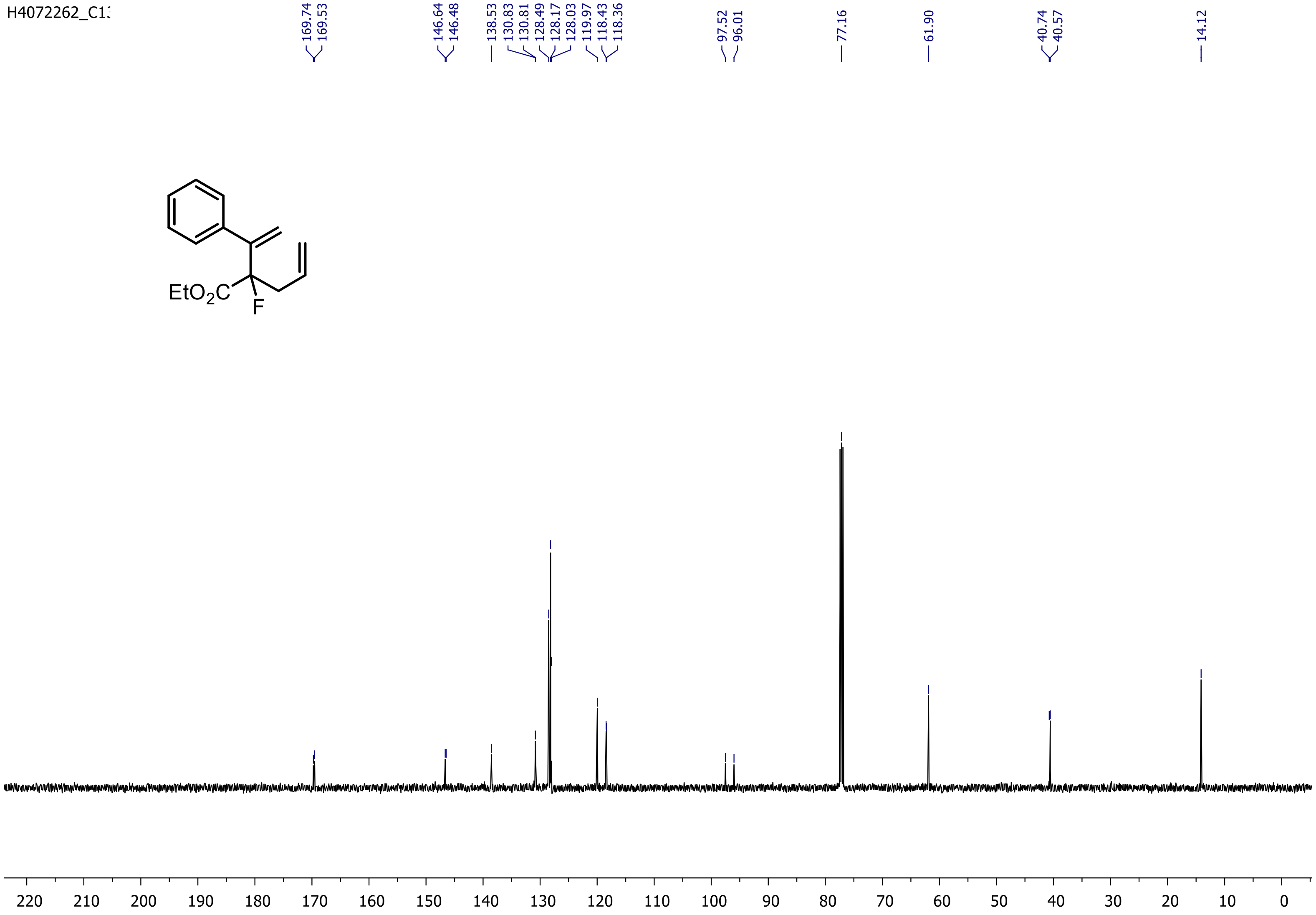

$^{19}F\{^{1}H\}$ NMR (376 MHz, $CDCl_3$)

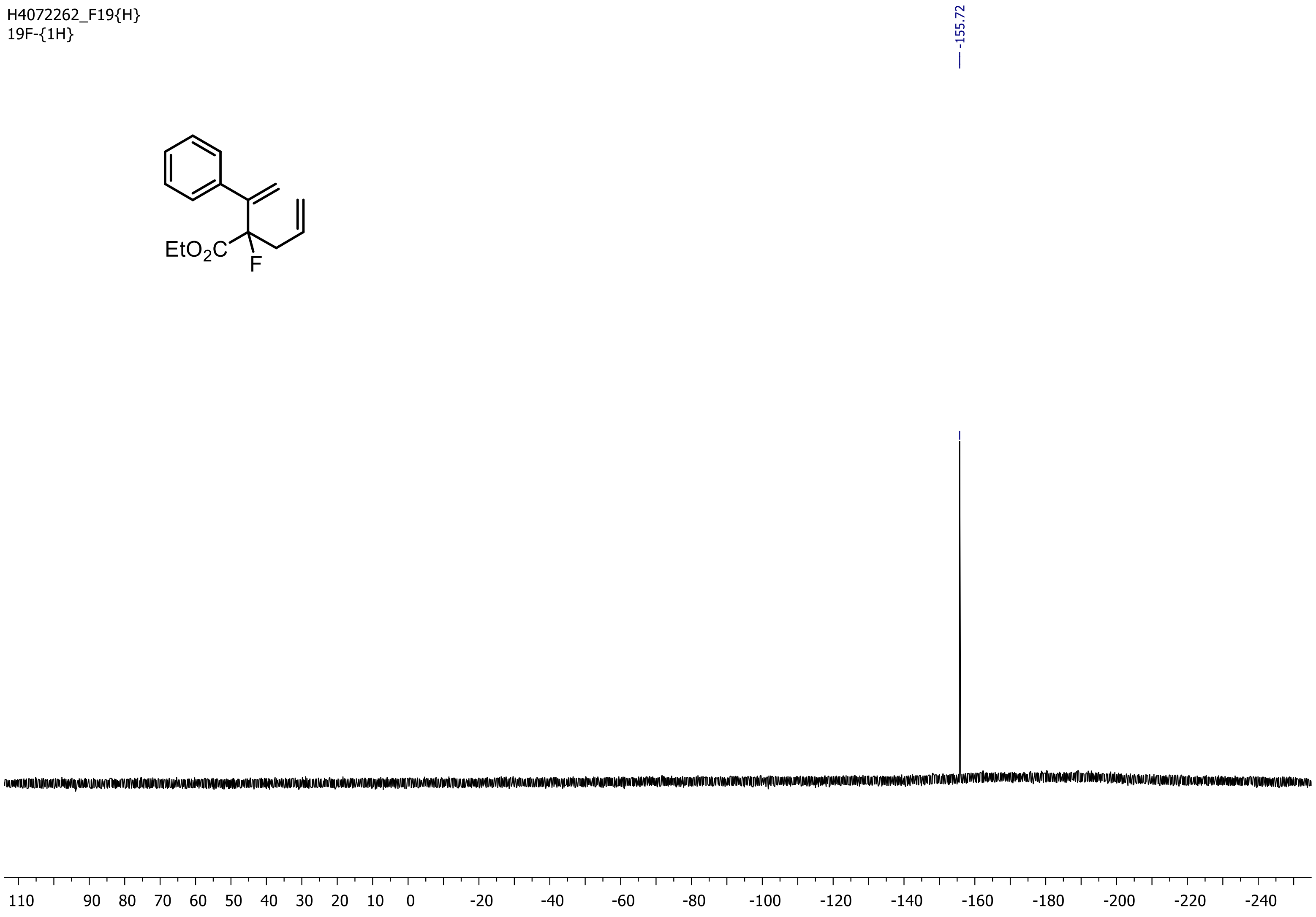

Compound (±)-3a

^{1}H NMR (500 MHz, $CDCl_3$)

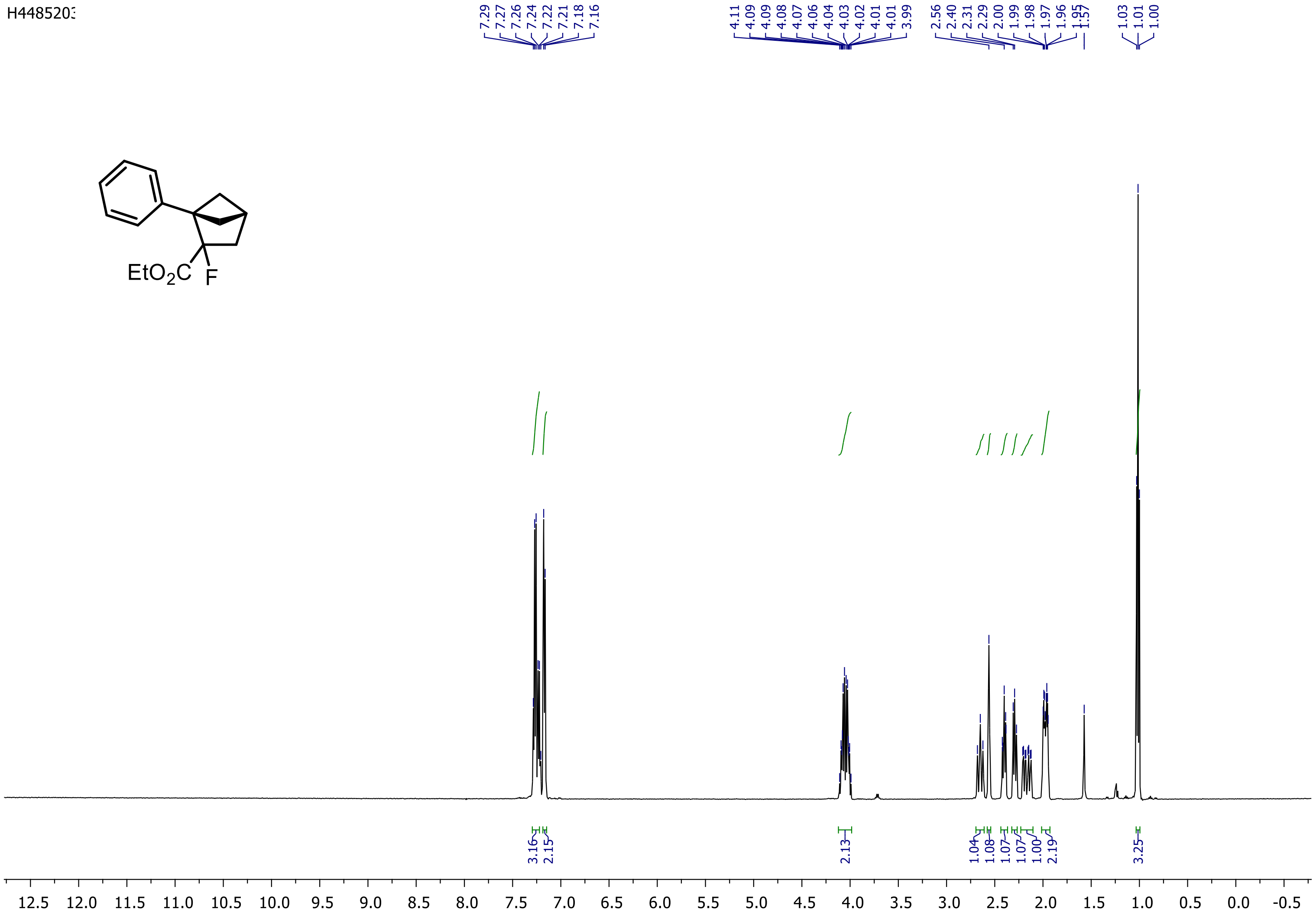

$^{13}C\{^1H\}$ NMR (126 MHz, $CDCl_3$)

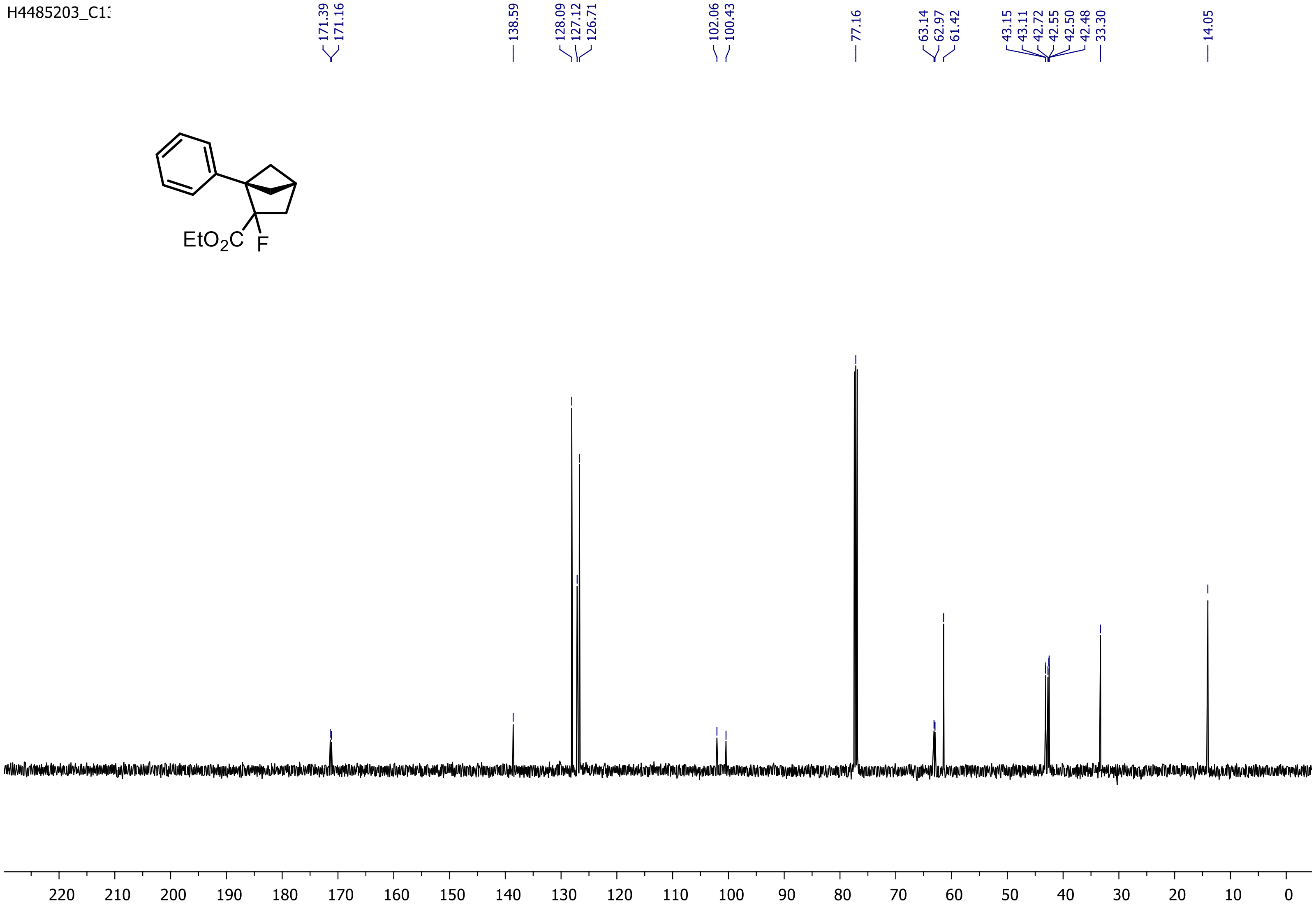

$^{19}F\{^1H\}$ NMR (376 MHz, $CDCl_3$)

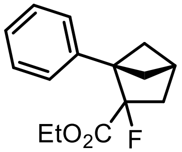

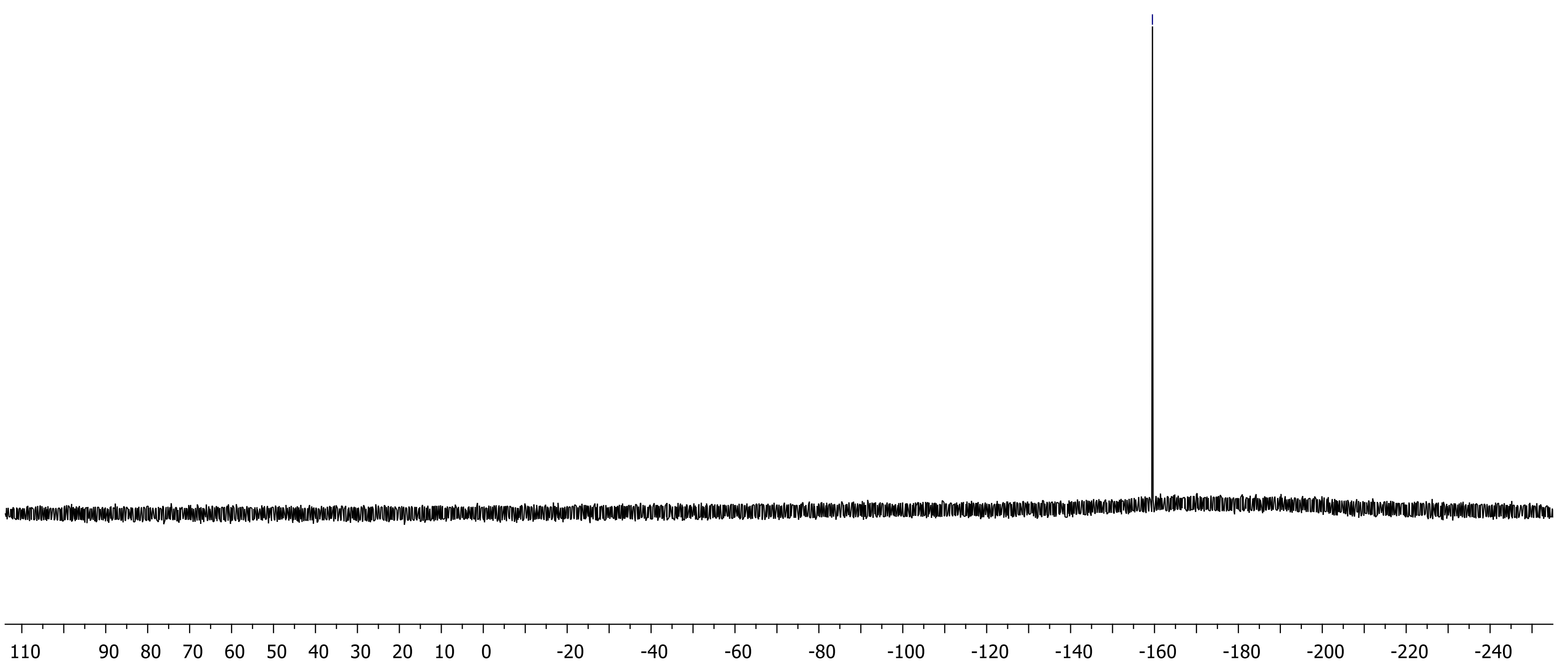

Compound (±)-3b

^{1}H NMR (500 MHz, DMSO-d_6)

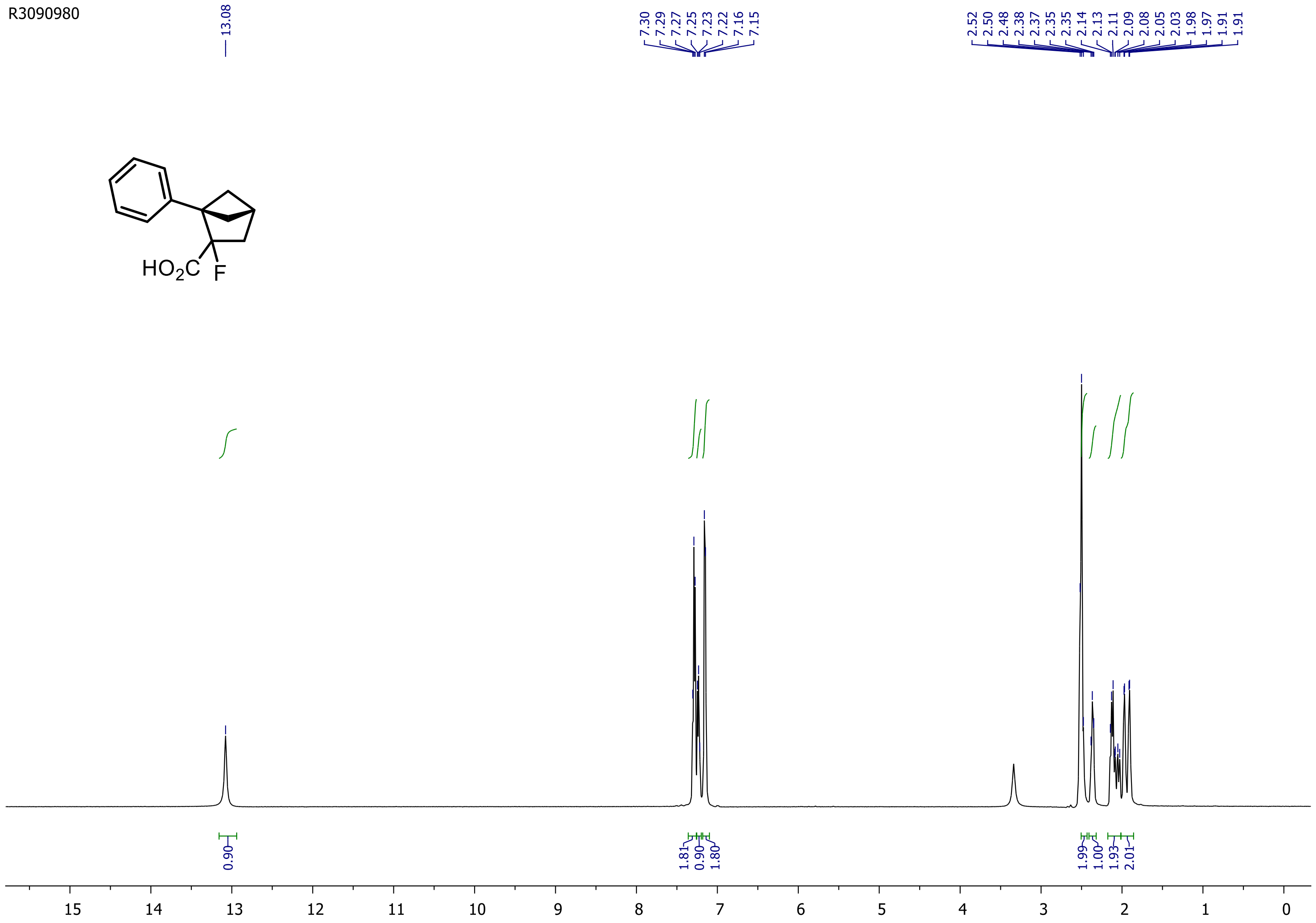

$^{13}C\{^{1}H\}$ NMR (126 MHz, DMSO-d_6)

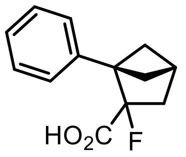

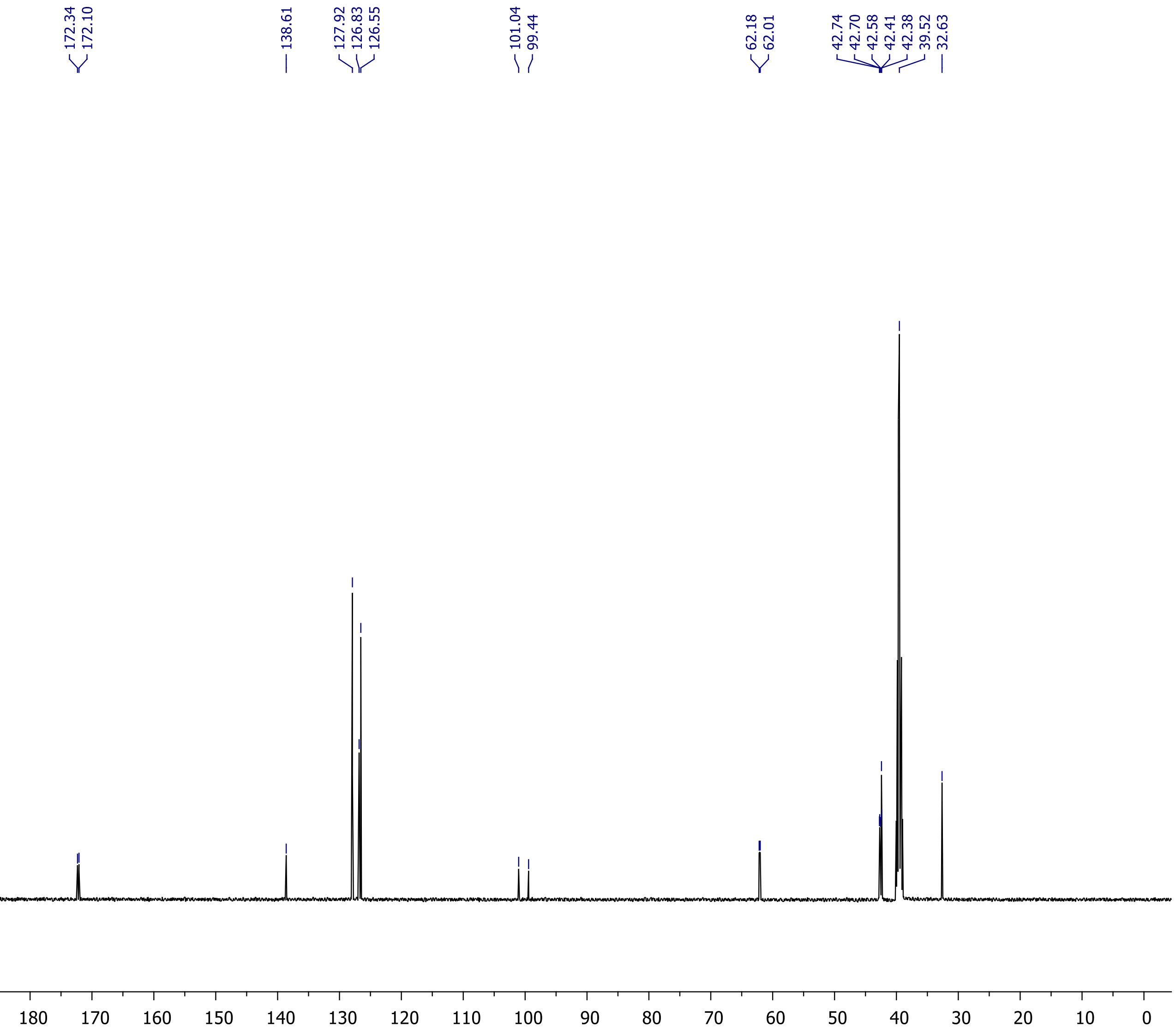

$^{19}F\{^{1}H\}$ NMR (376 MHz, DMSO-d_6)

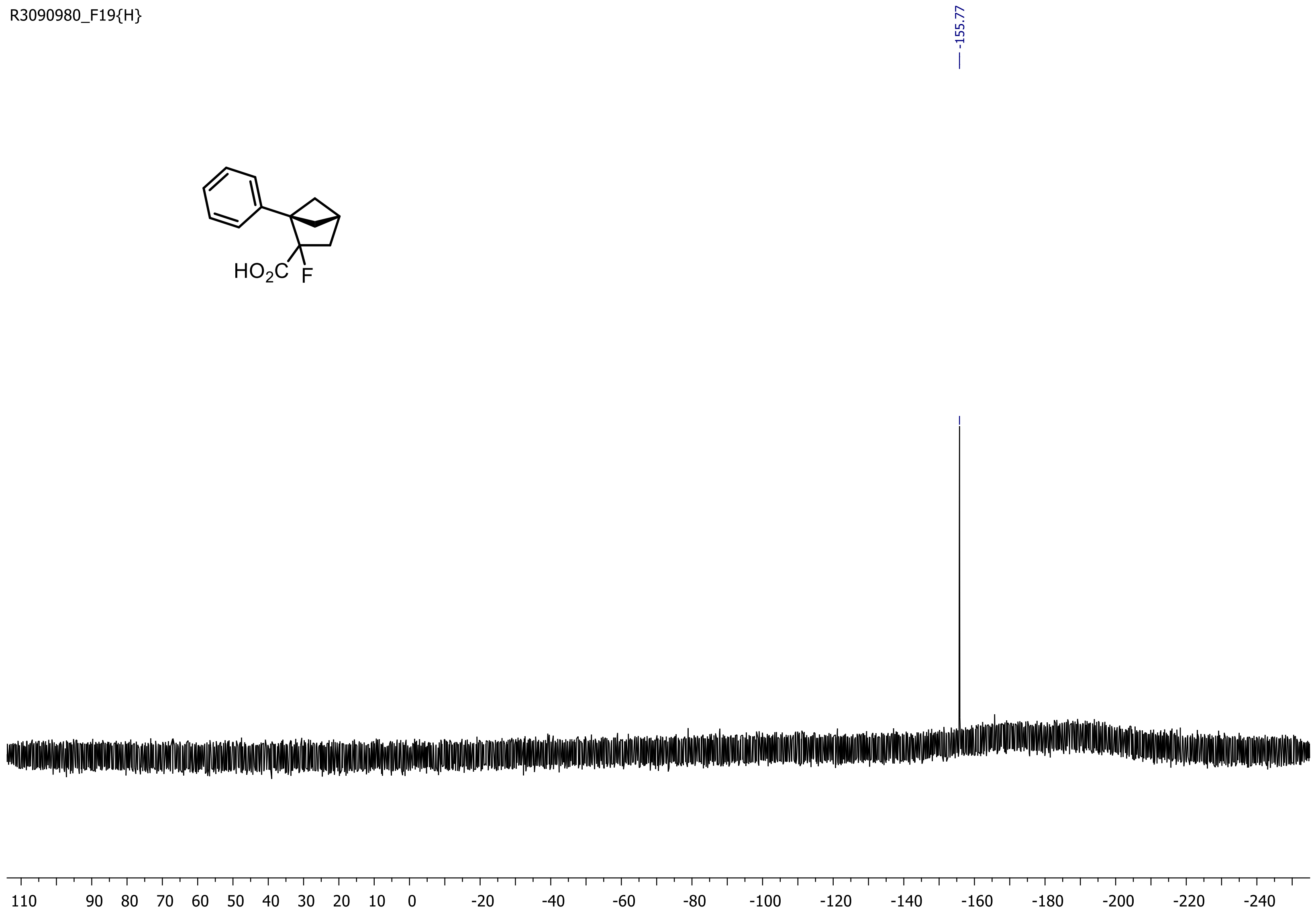

Ethyl-3-(4-fluorophenyl)but-2-enoate

^{1}H NMR (500 MHz, $CDCl_3$)

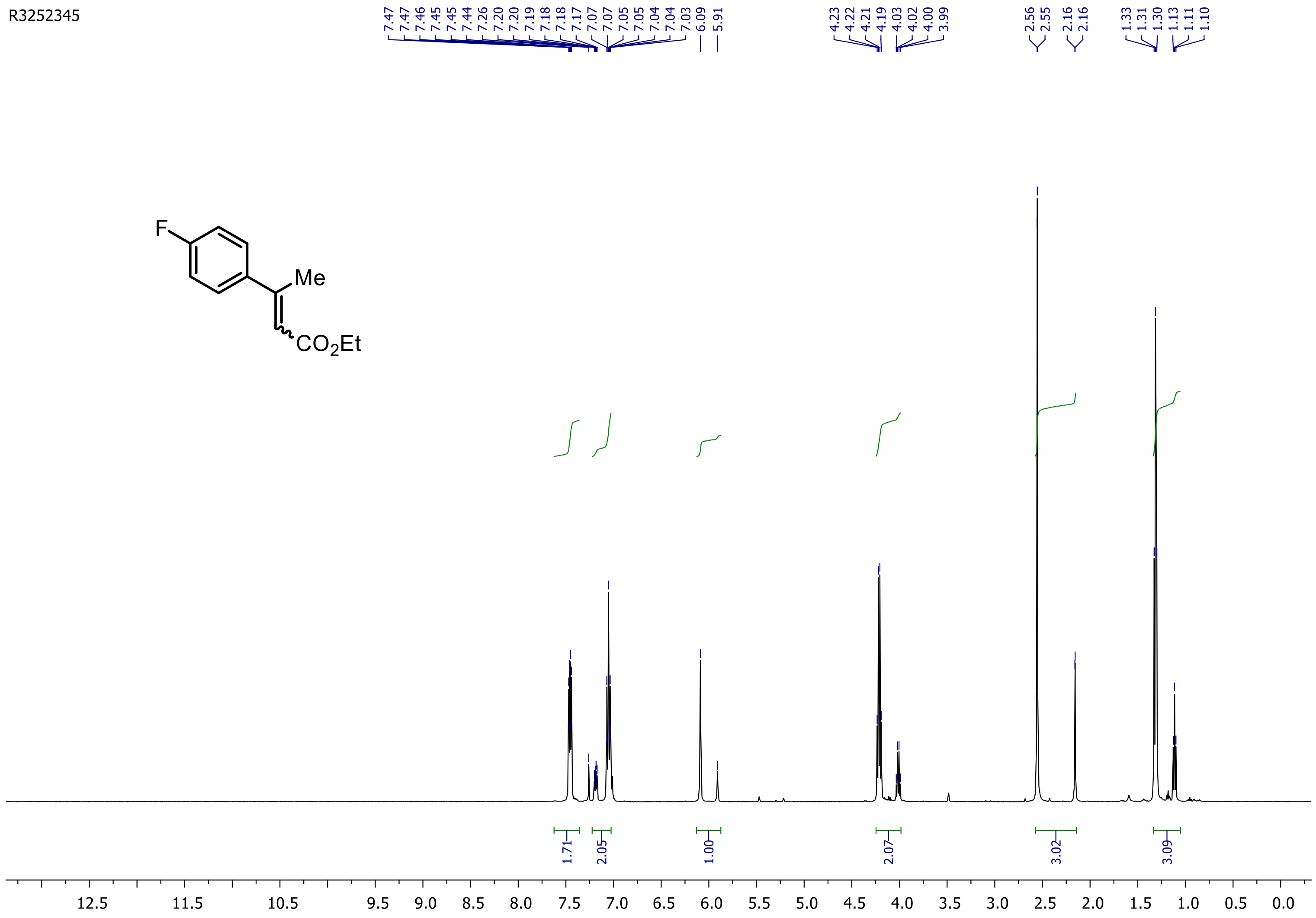

$^{13}C\{^{1}H\}$ NMR (151 MHz, $CDCl_3$)

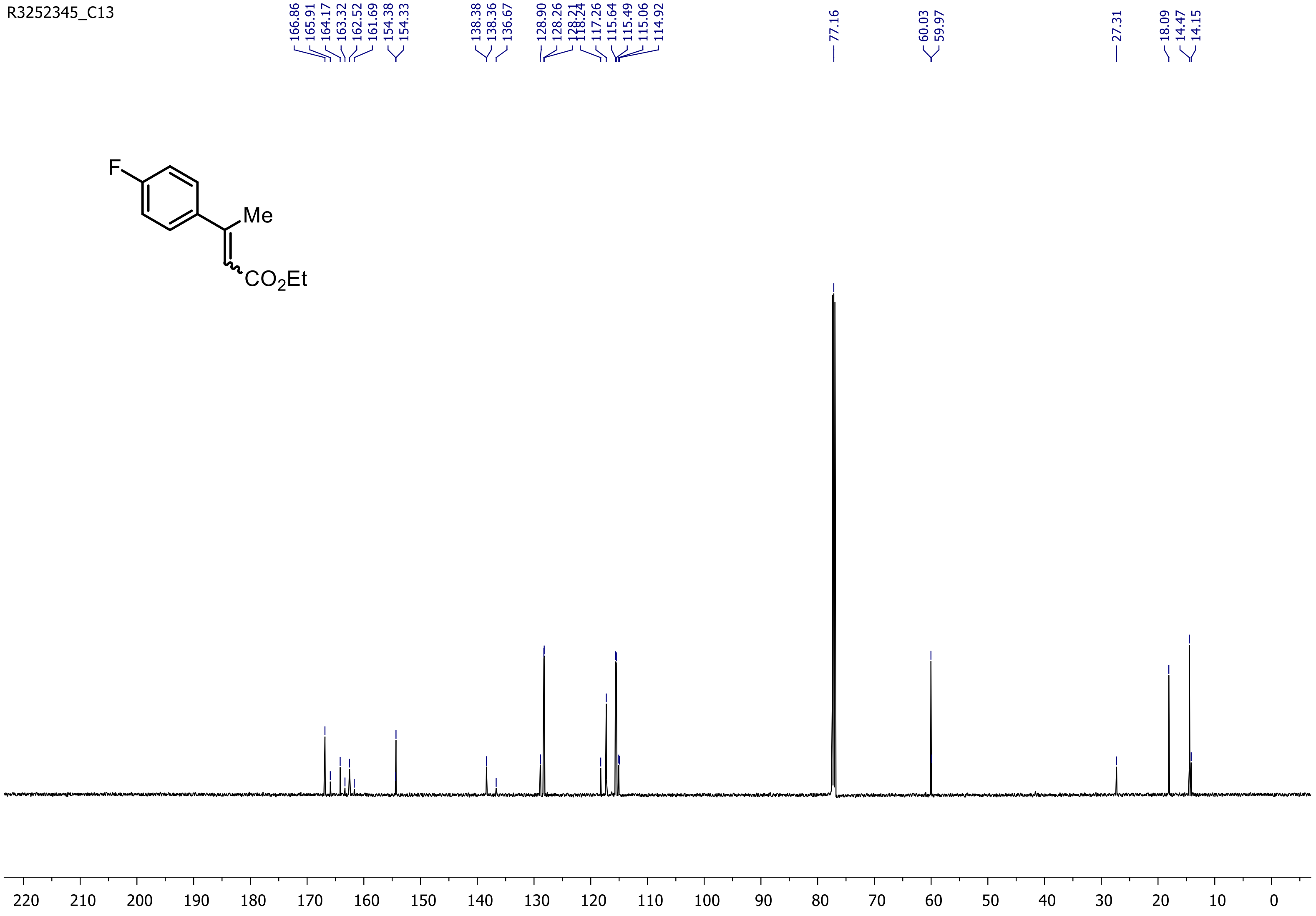

$^{19}F\{^{1}H\}$ NMR (376 MHz, $CDCl_3$)

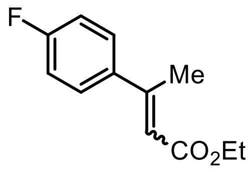

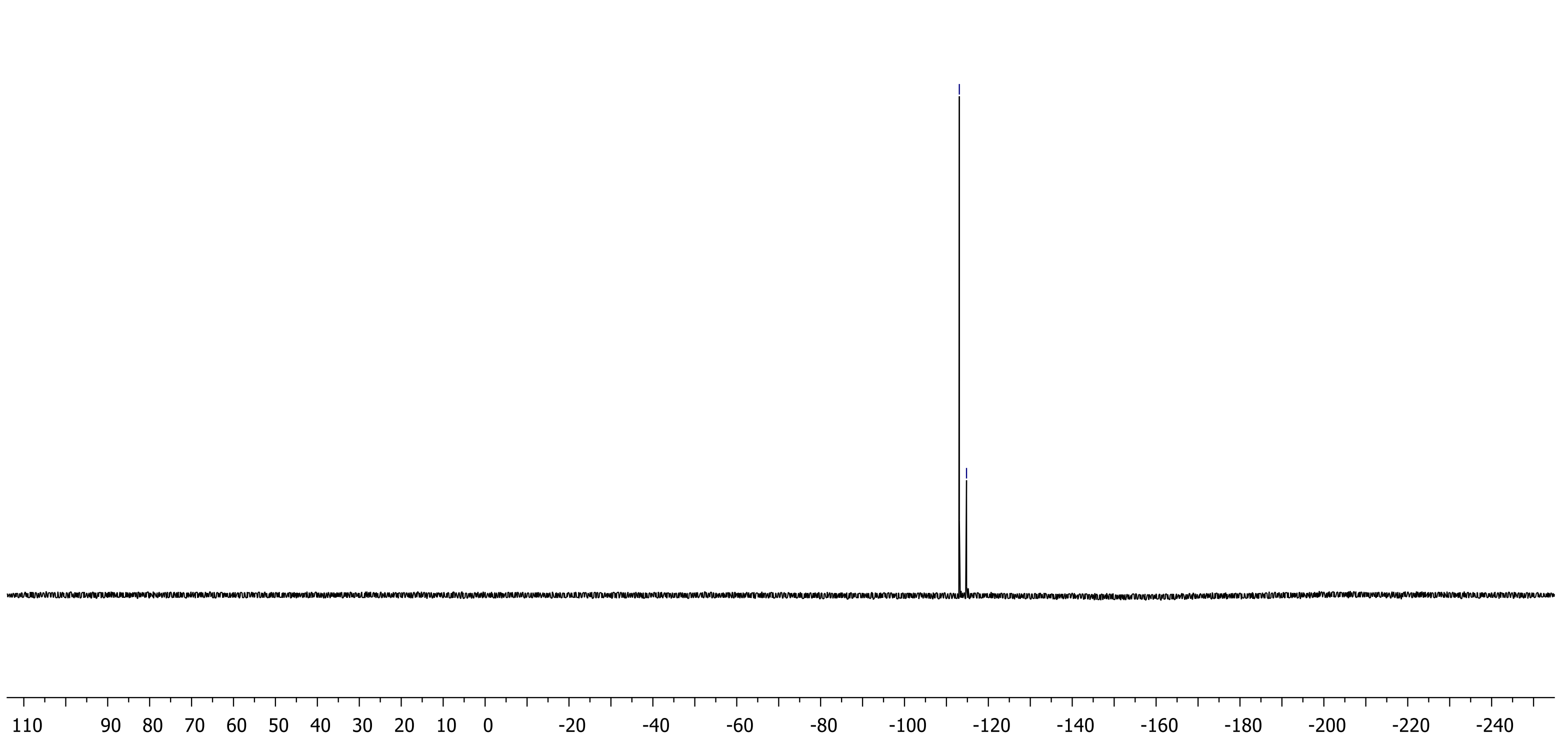

Compound 4

^{1}H NMR (500 MHz, $CDCl_3$)

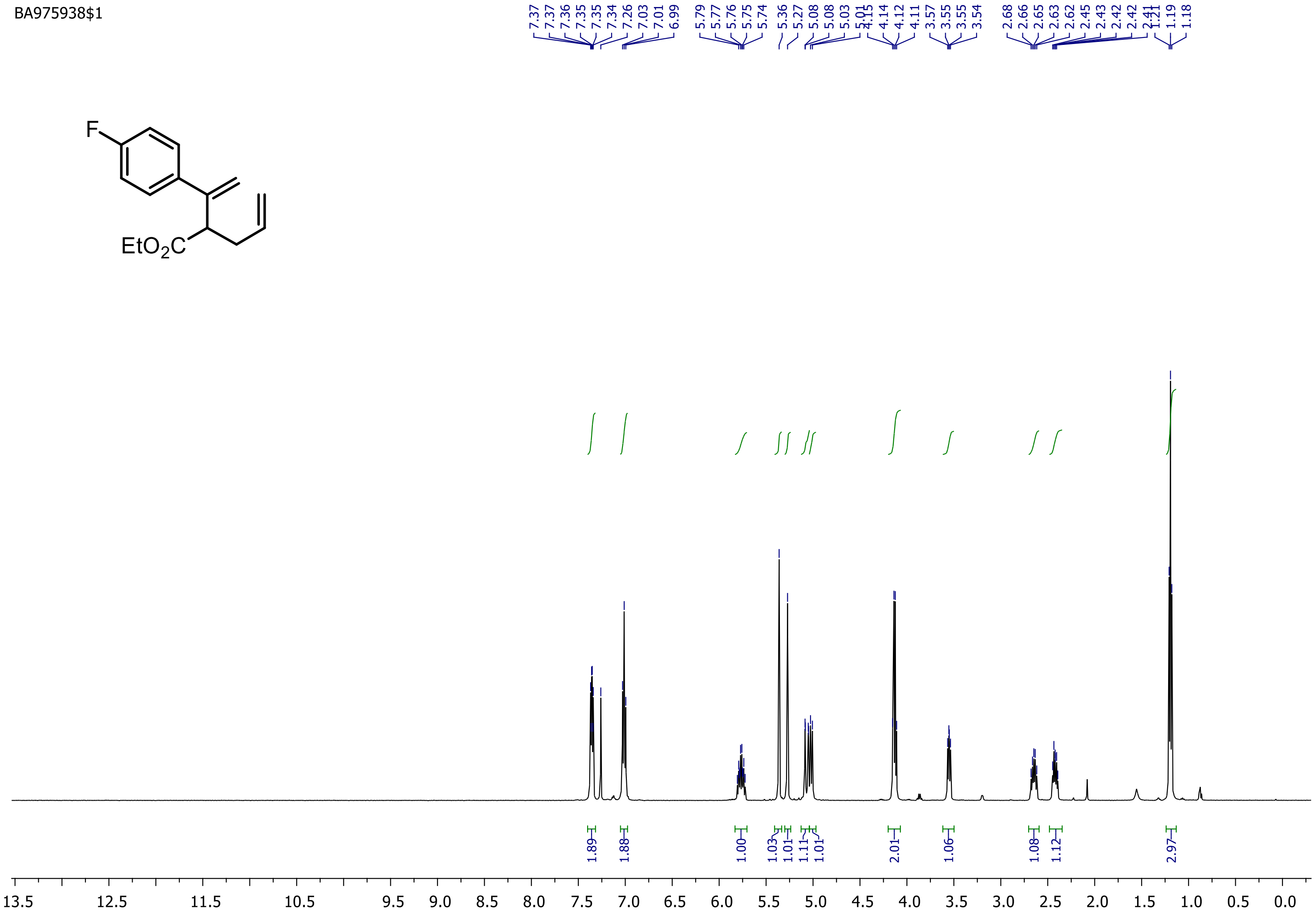

$^{13}C\{^{1}H\}$ NMR (151 MHz, $CDCl_3$)

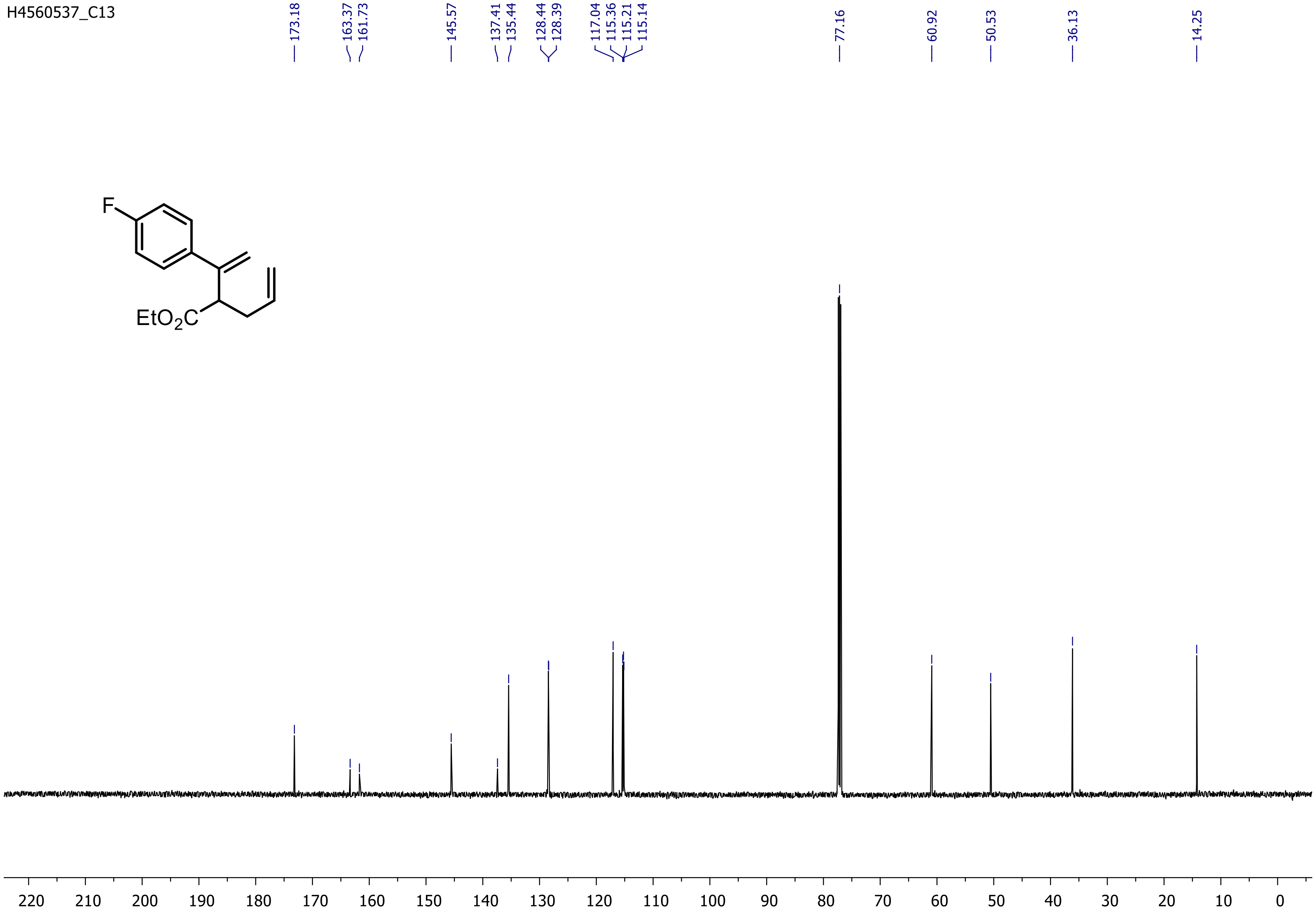

$^{19}F\{^1H\}$ NMR (376 MHz, $CDCl_3$)

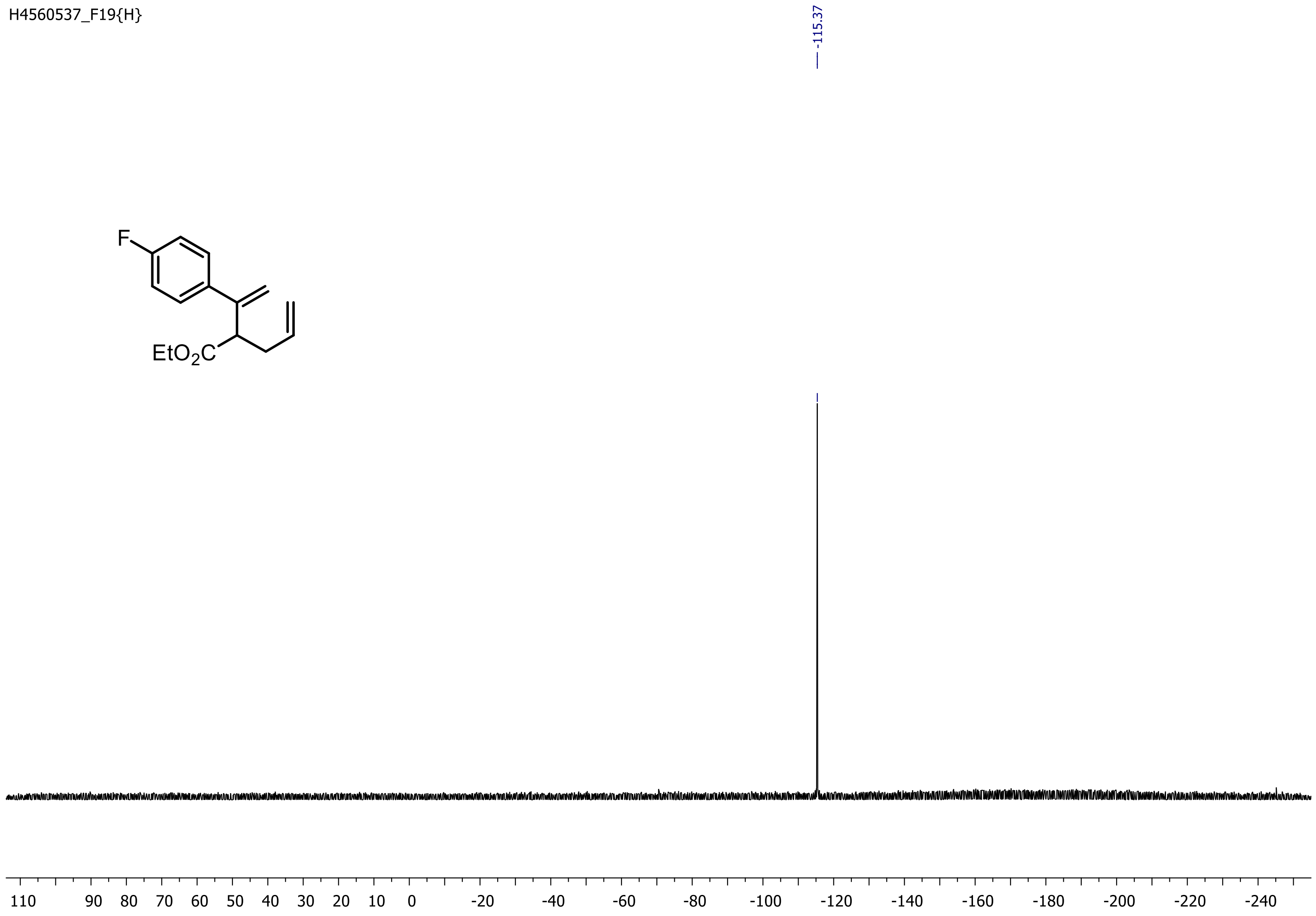

Compound (±)-4a (the sample contains ca. 10% of the isomeric 1,5-disubstituted bicyclo[2.1.1]hexane)

^{1}H NMR (500 MHz, $CDCl_3$)

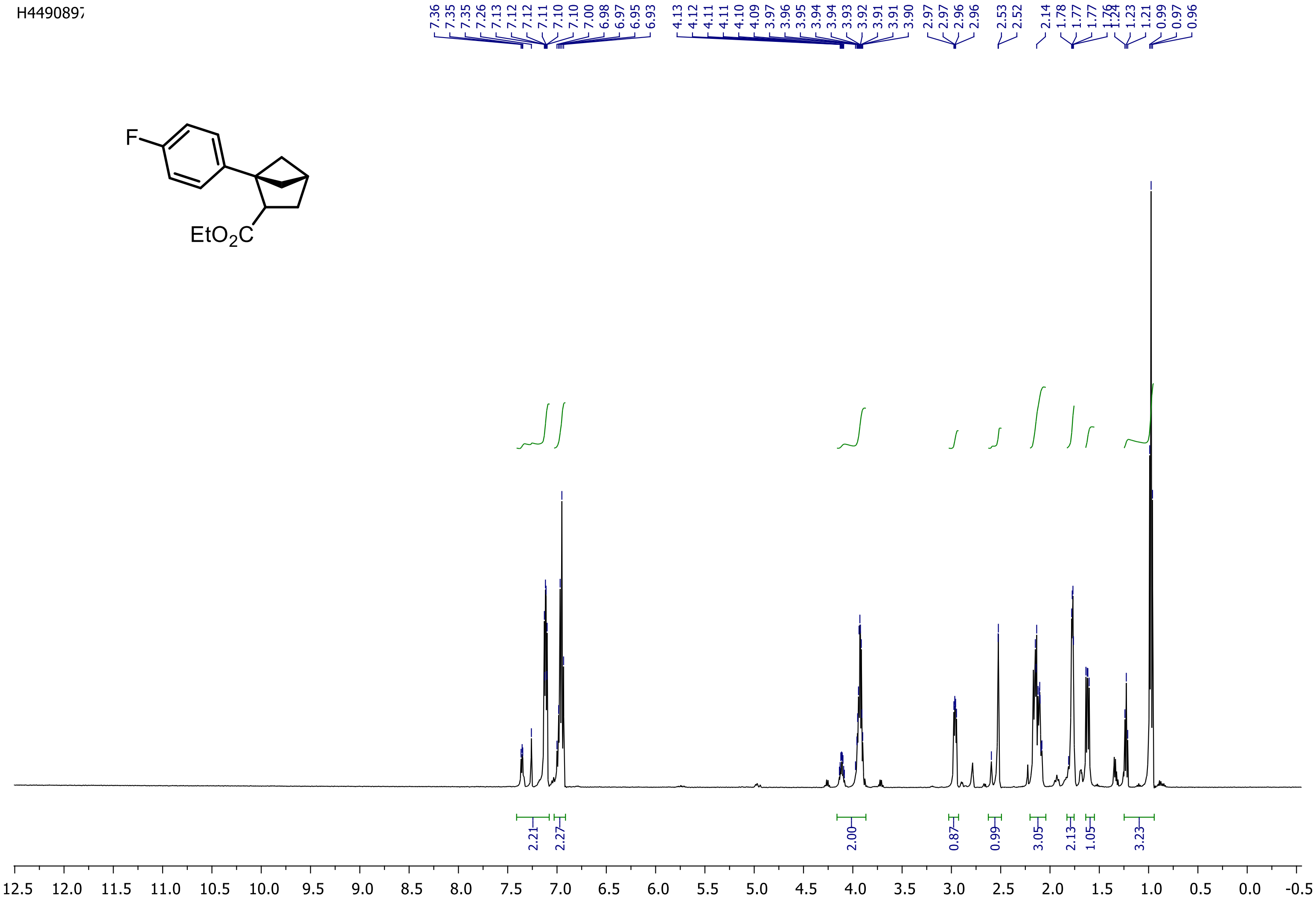

$^{13}C\{^1H\}$ NMR (126 MHz, $CDCl_3$)

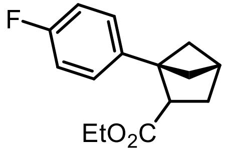

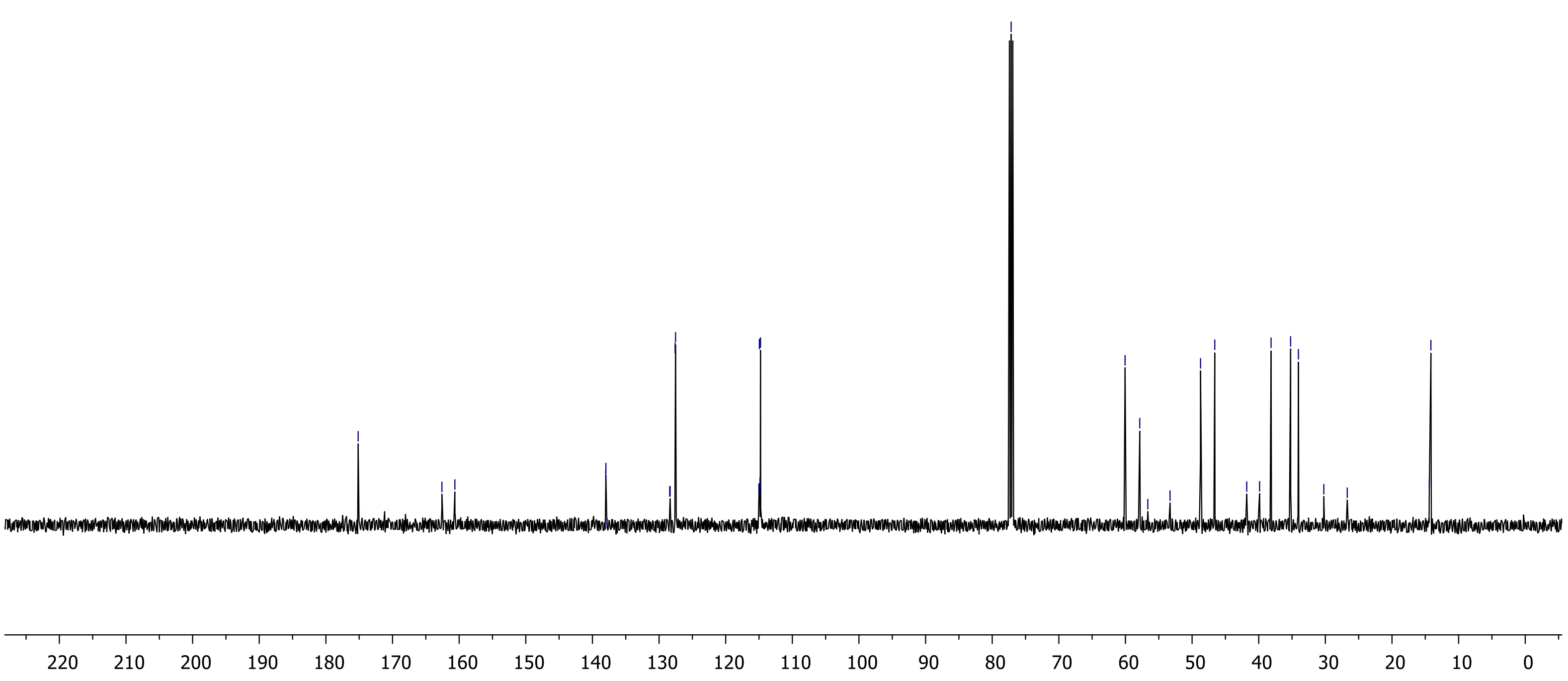

$^{19}F\{^{1}H\}$ NMR (376 MHz, $CDCl_3$)

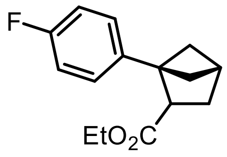

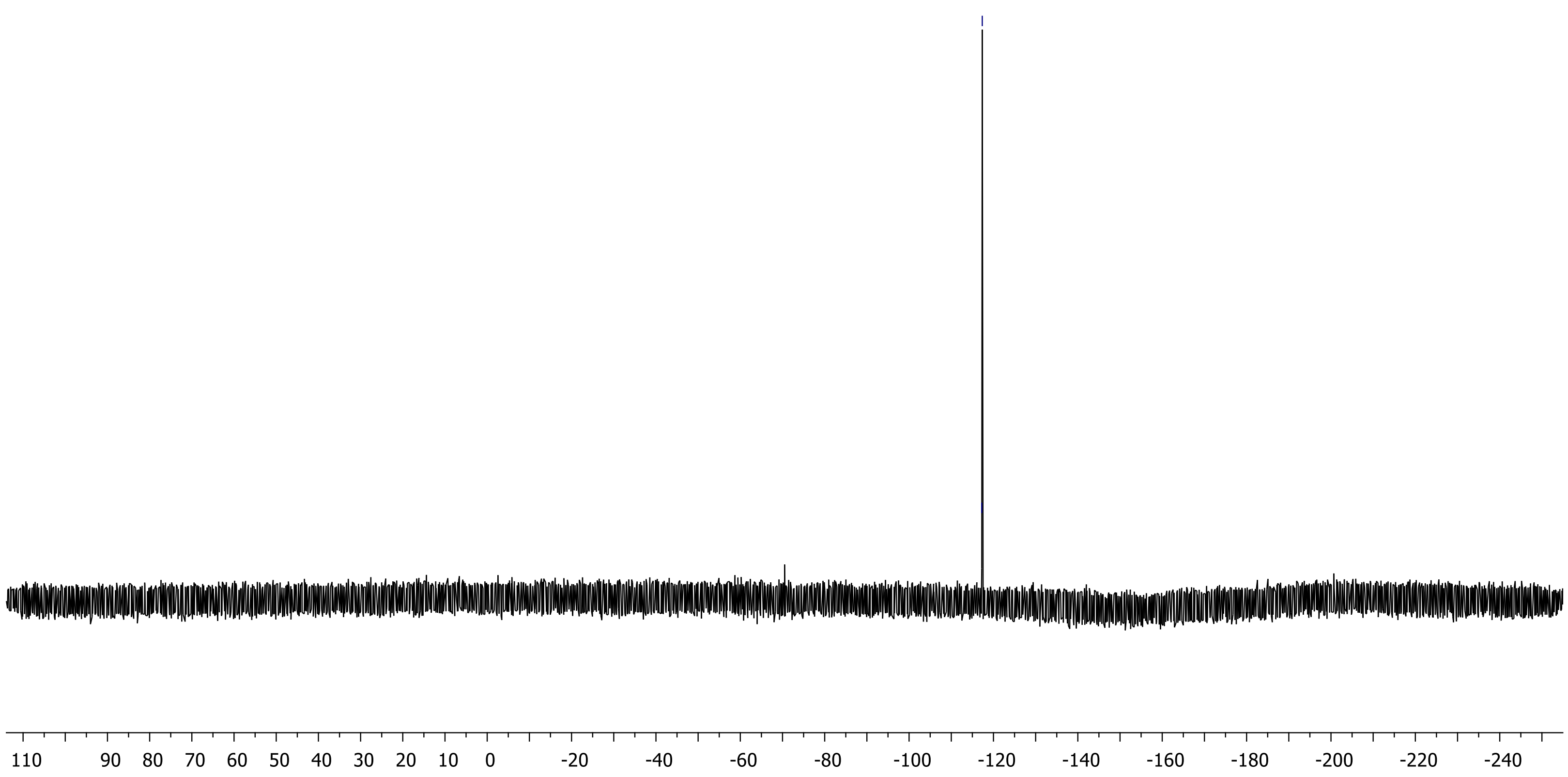

Compound (±)-4b

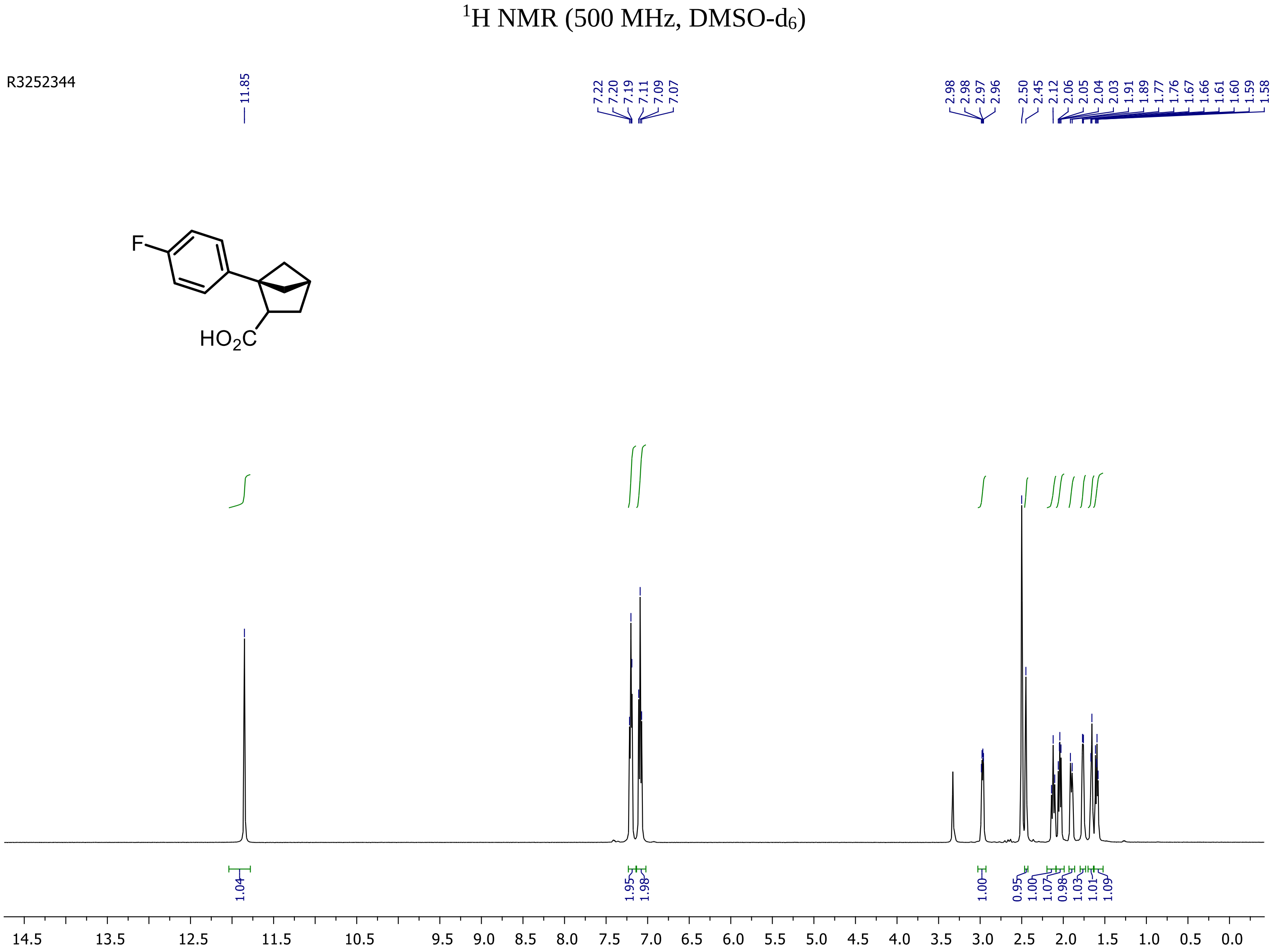
F
HO2C
^{1}H NMR (500 MHz, DMSO-d$_6$)
R3252344
11.85
7.22
7.20
7.19
7.11
7.09
7.07
2.98
2.98
2.97
2.96
2.50
2.45
2.12
2.06
2.05
2.04
2.03
1.91
1.89
1.77
1.76
1.67
1.66
1.61
1.60
1.59
1.58
1.04
1.95
1.98
1.00
0.95
1.00
1.07
0.98
1.03
1.01
1.09
14.5
13.5
12.5
11.5
10.5
9.5
9.0
8.5
8.0
7.5
7.0
6.5
6.0
5.5
5.0
4.5
4.0
3.5
3.0
2.5
2.0
1.5
1.0
0.5
0.0

$^{13}C\{^1H\}$ NMR (151 MHz, DMSO-d_6)

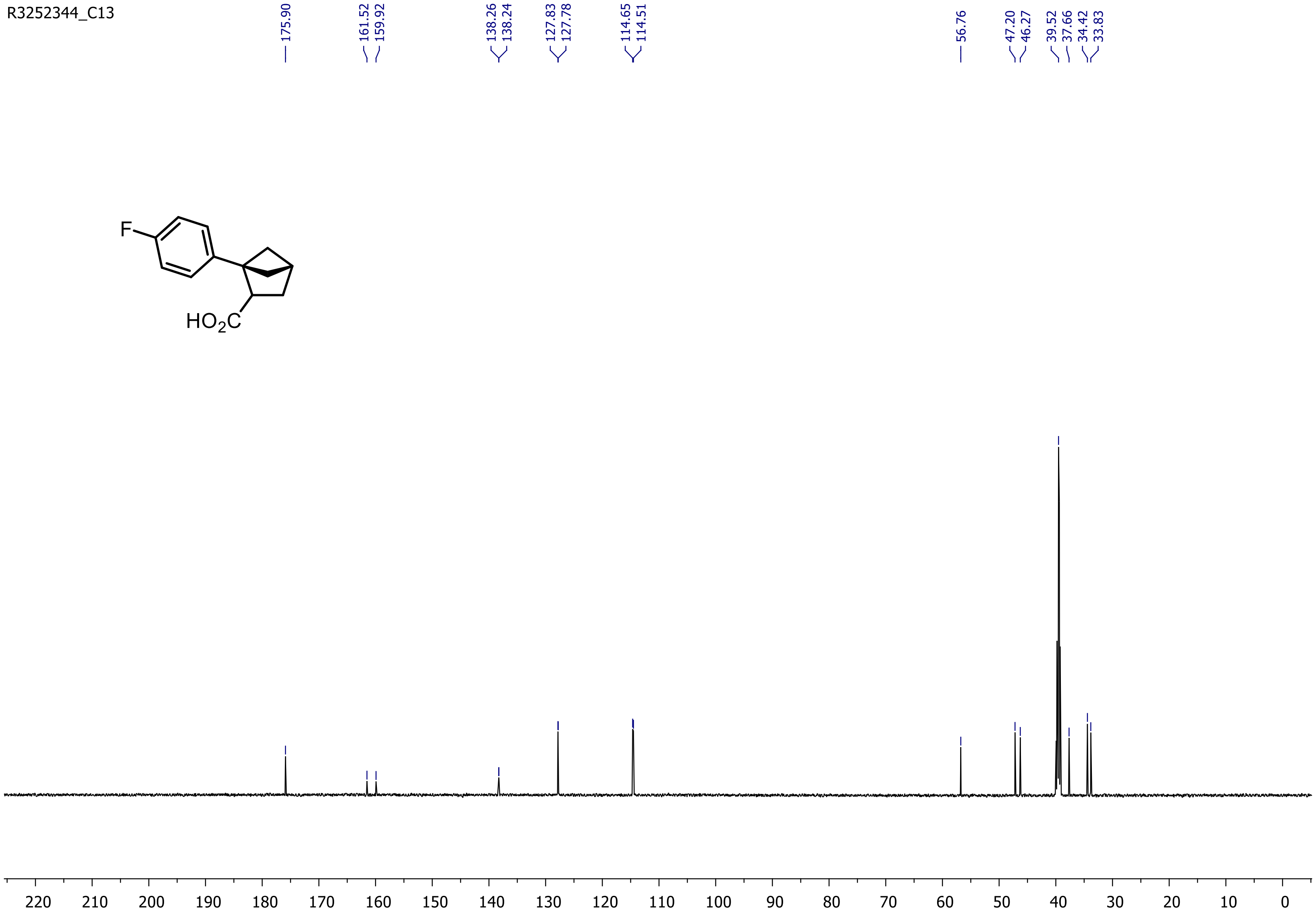

$^{19}F\{^{1}H\}$ NMR (376 MHz, DMSO-d$_6$)

R3252344_F19{H}

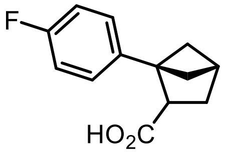

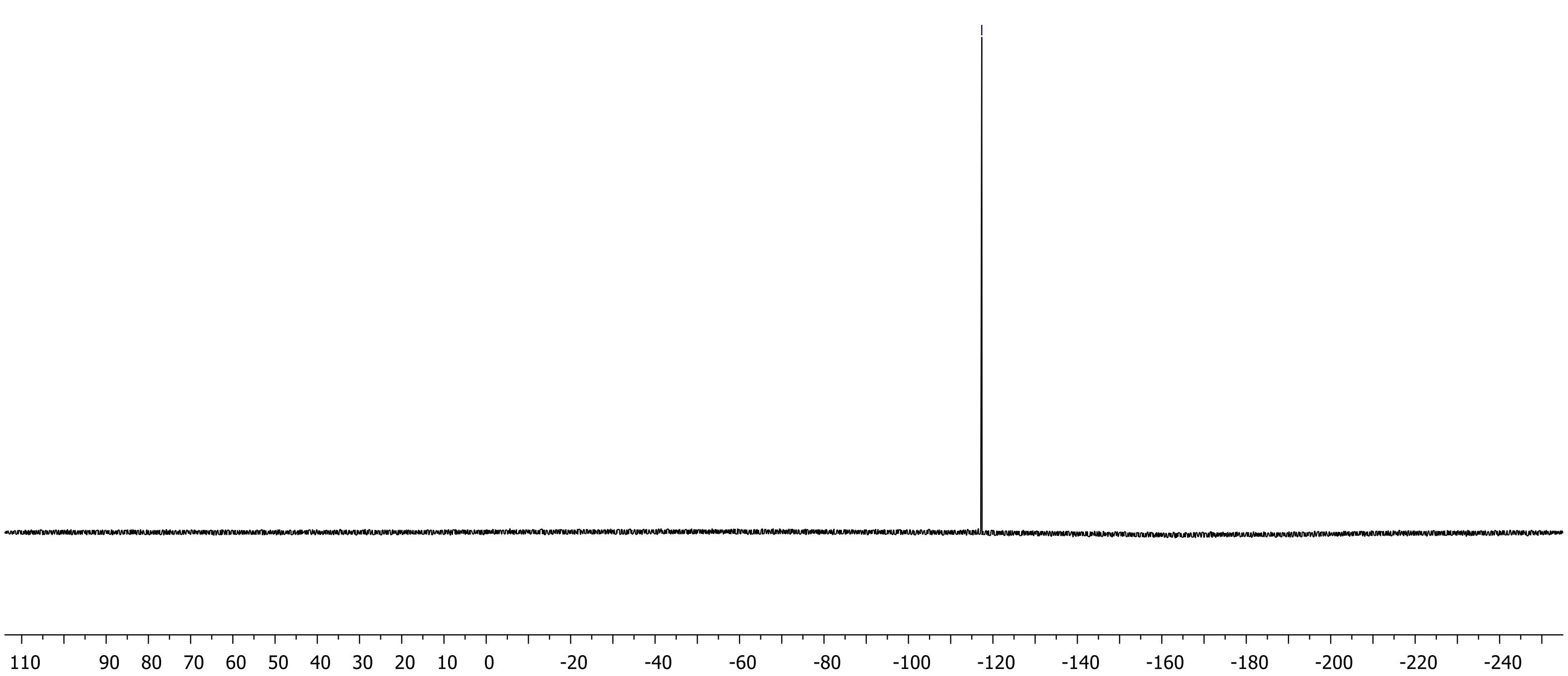

Ethyl-3-(4-chlorophenyl)but-2-enoate

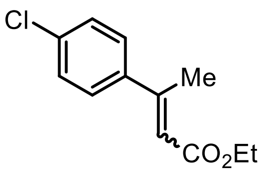

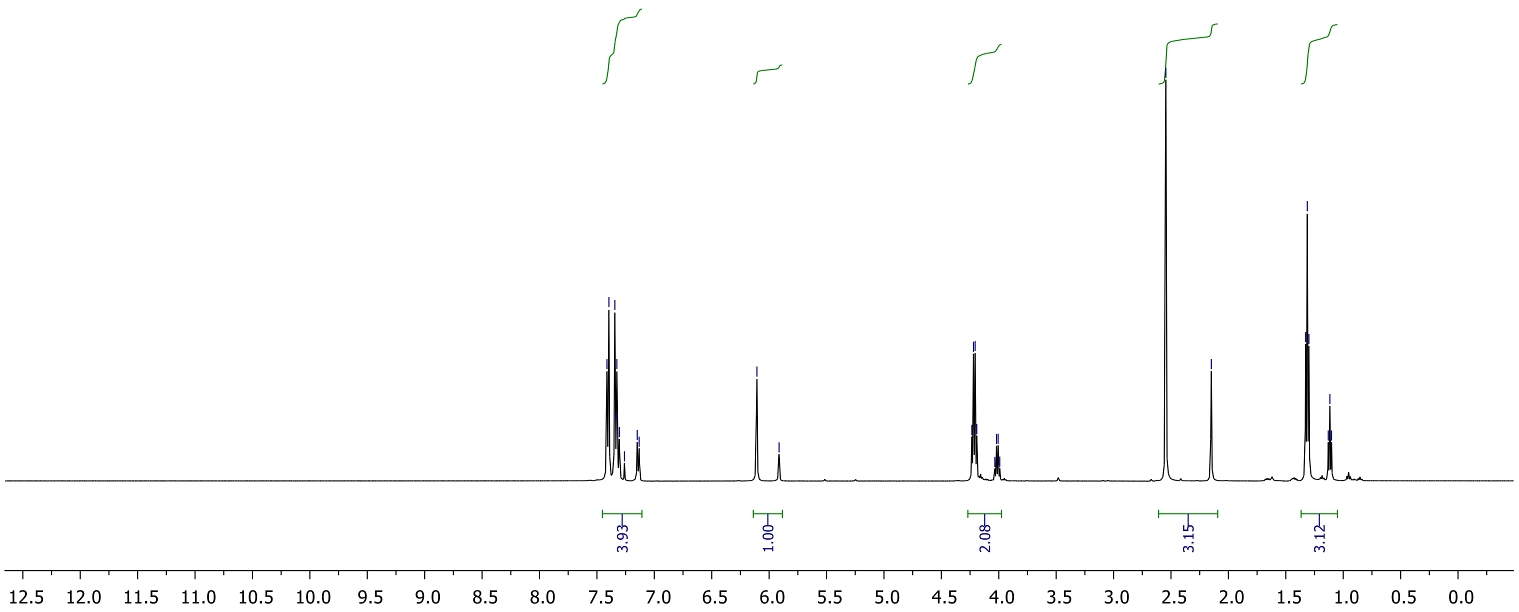

$^{13}C\{^1H\}$ NMR (101 MHz, $CDCl_3$)

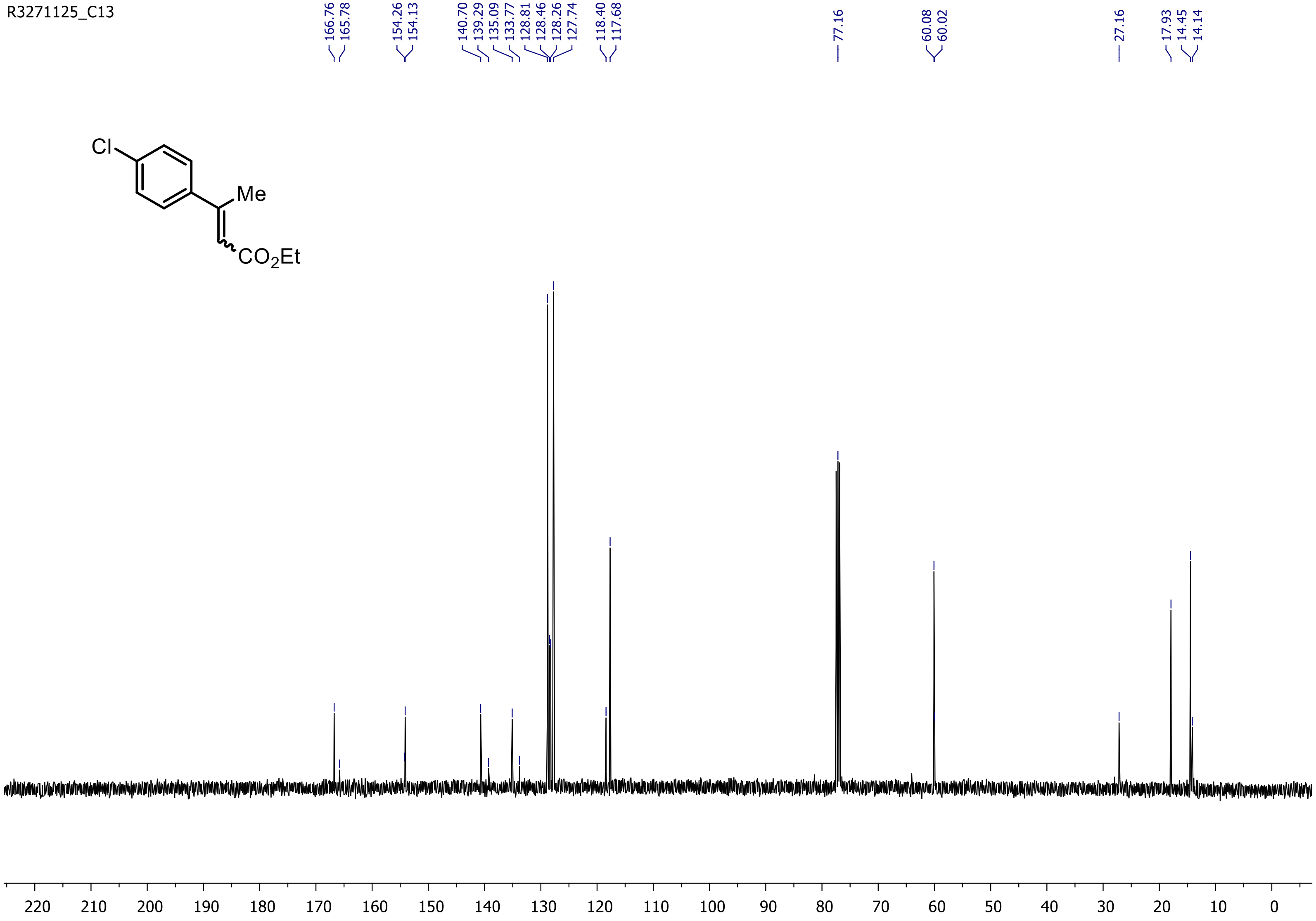

Compound 5

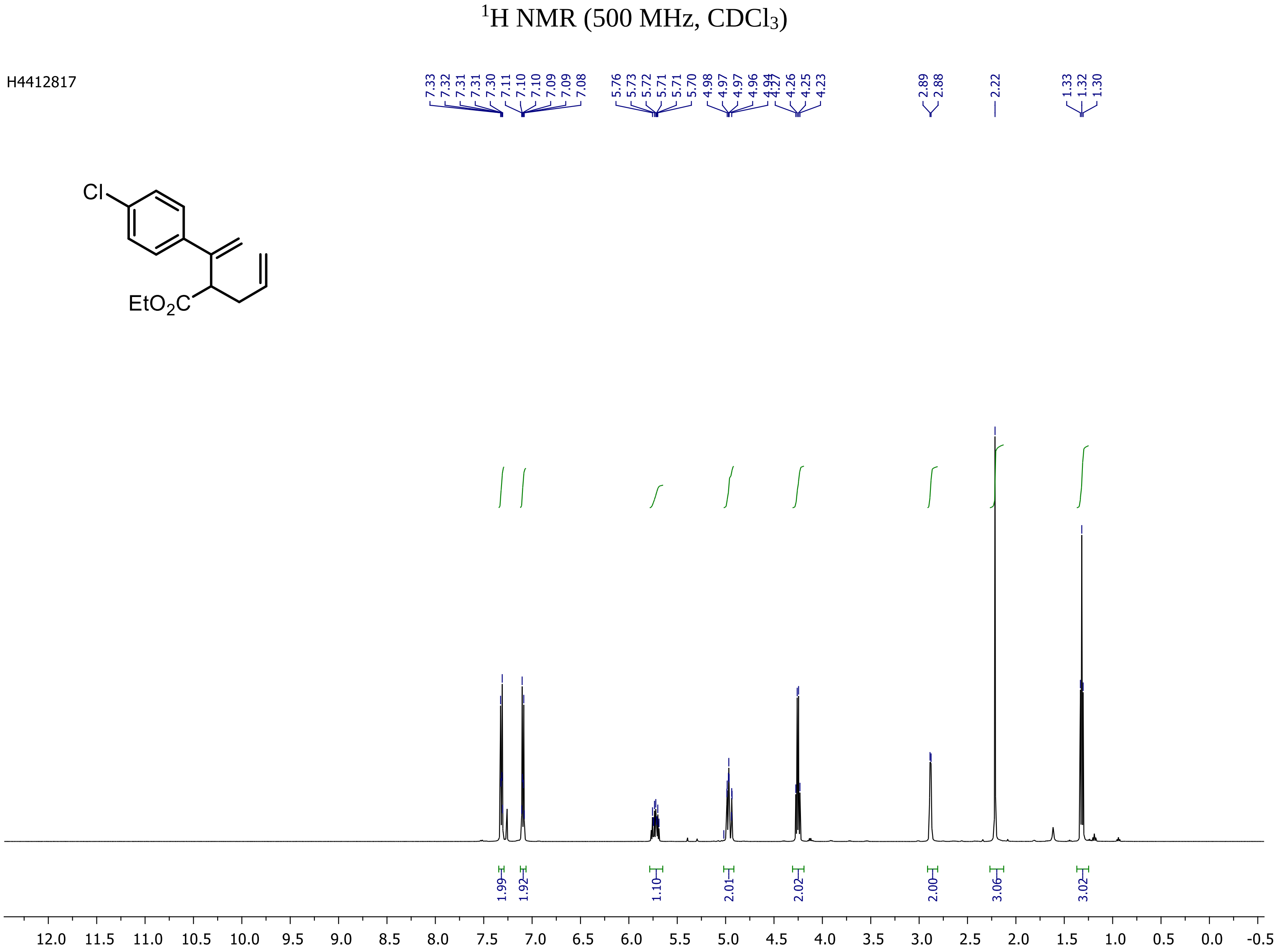
Cl
EtO2C
^{1}H NMR (500 MHz, CDCl$_3$)
H4412817
7.33
7.32
7.31
7.31
7.30
7.11
7.10
7.10
7.09
7.09
7.08
5.76
5.73
5.72
5.71
5.71
5.70
4.98
4.97
4.97
4.96
4.94
4.27
4.26
4.25
4.23
2.89
2.88
2.22
1.33
1.32
1.30
1.99
1.92
1.10
2.01
2.02
2.00
3.06
3.02
12.0
11.5
11.0
10.5
10.0
9.5
9.0
8.5
8.0
7.5
7.0
6.5
6.0
5.5
5.0
4.5
4.0
3.5
3.0
2.5
2.0
1.5
1.0
0.5
0.0
-0.5

$^{13}C\{^{1}H\}$ NMR (126 MHz, $CDCl_3$)

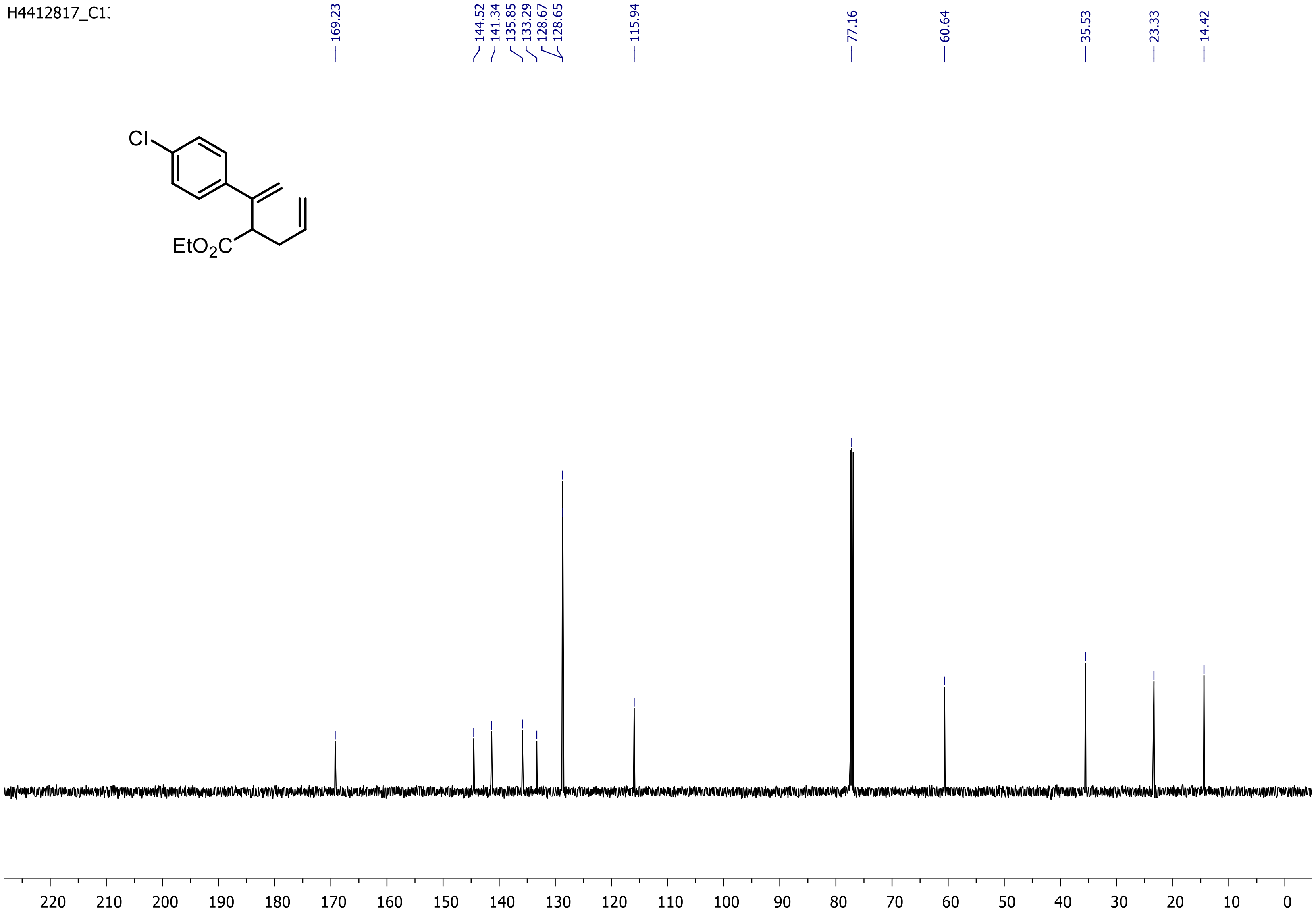

Compound (±)-5a

^{1}H NMR (500 MHz, DMSO-d_6)

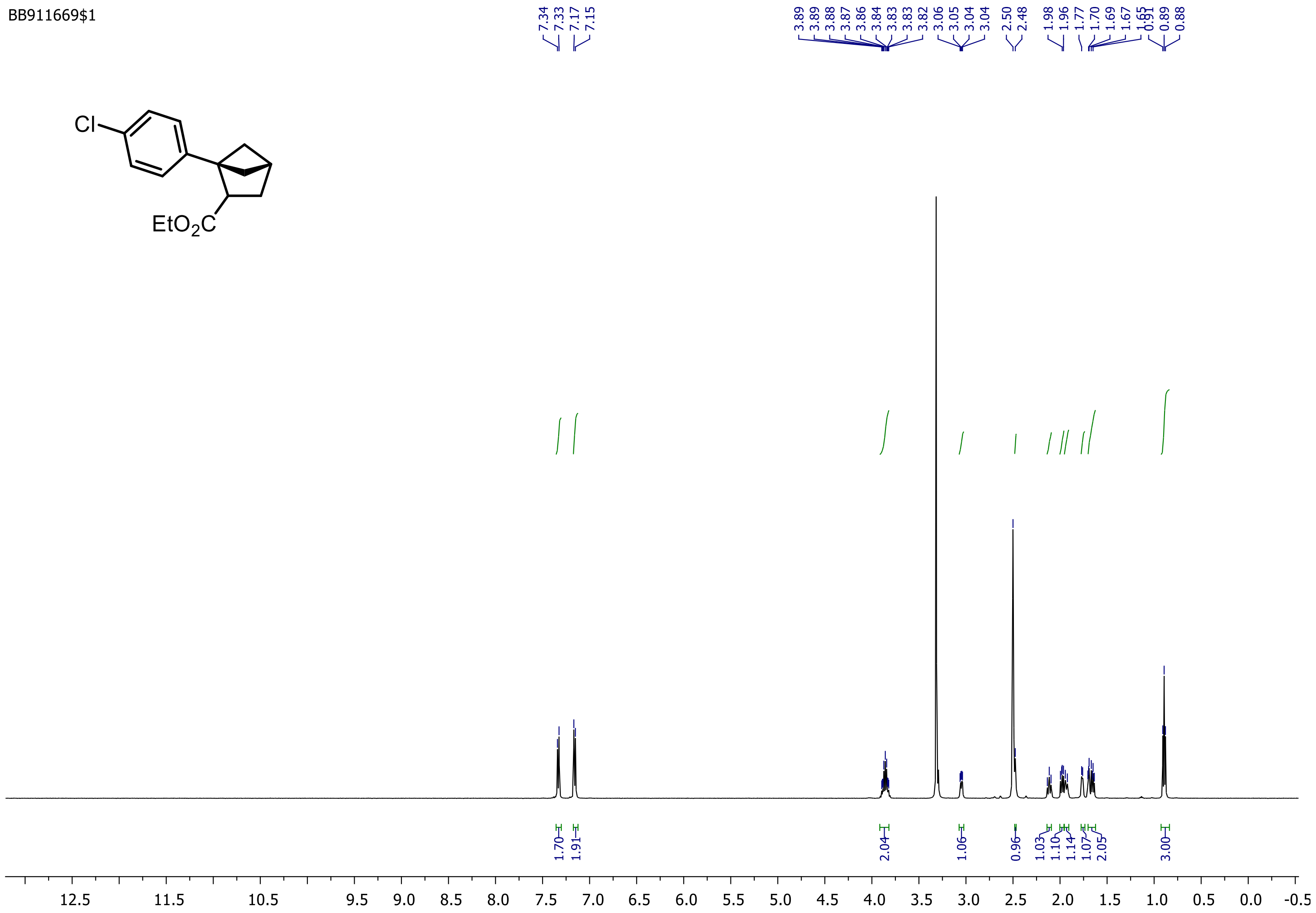

$^{13}C\{^1H\}$ NMR (151 MHz, $CDCl_3$)

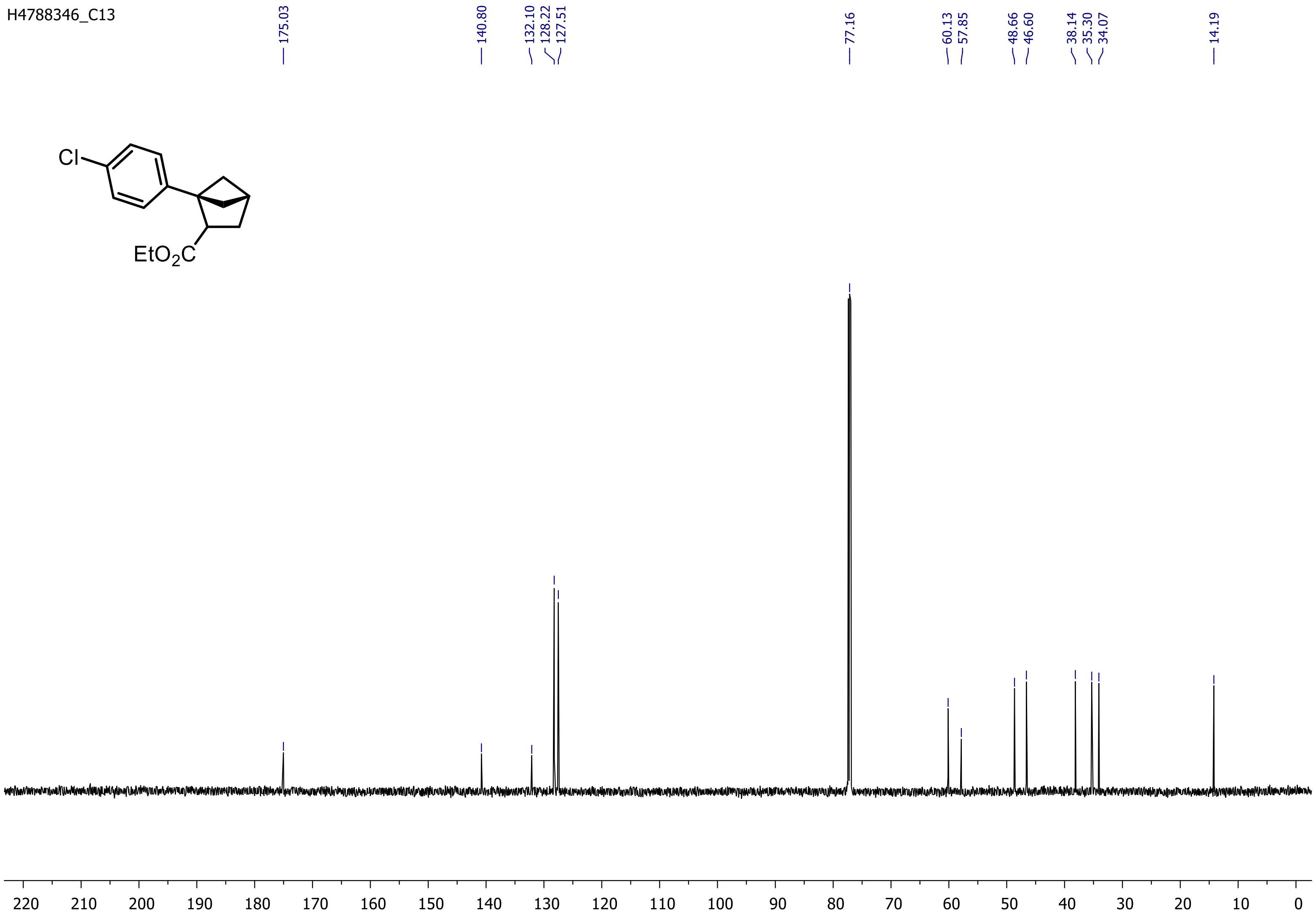

Compound (±)-5b

^{1}H NMR (500 MHz, DMSO-d_6)

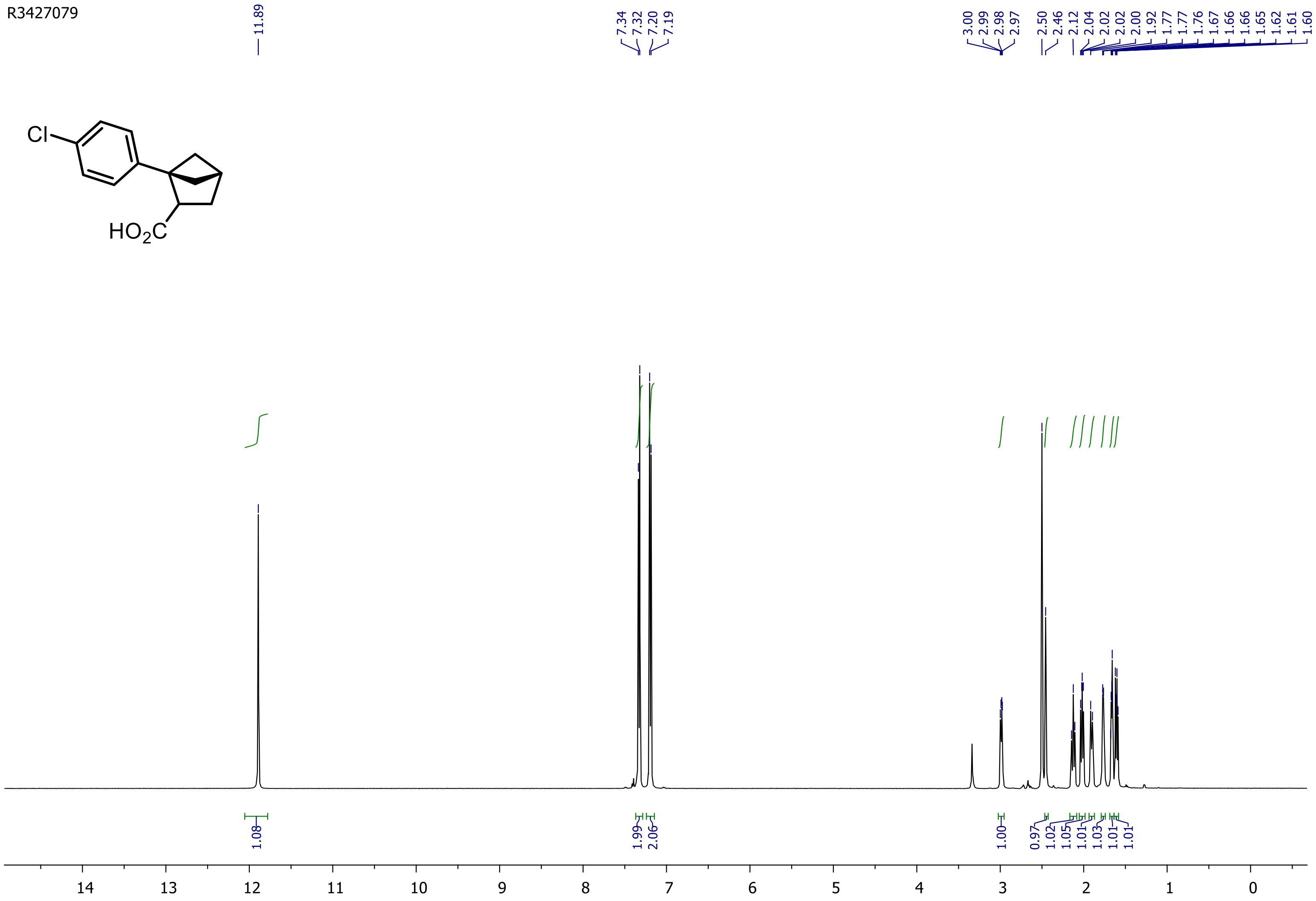

$^{13}C\{^{1}H\}$ NMR (126 MHz, DMSO-d_6)

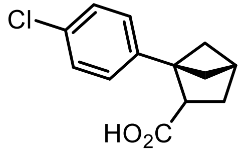

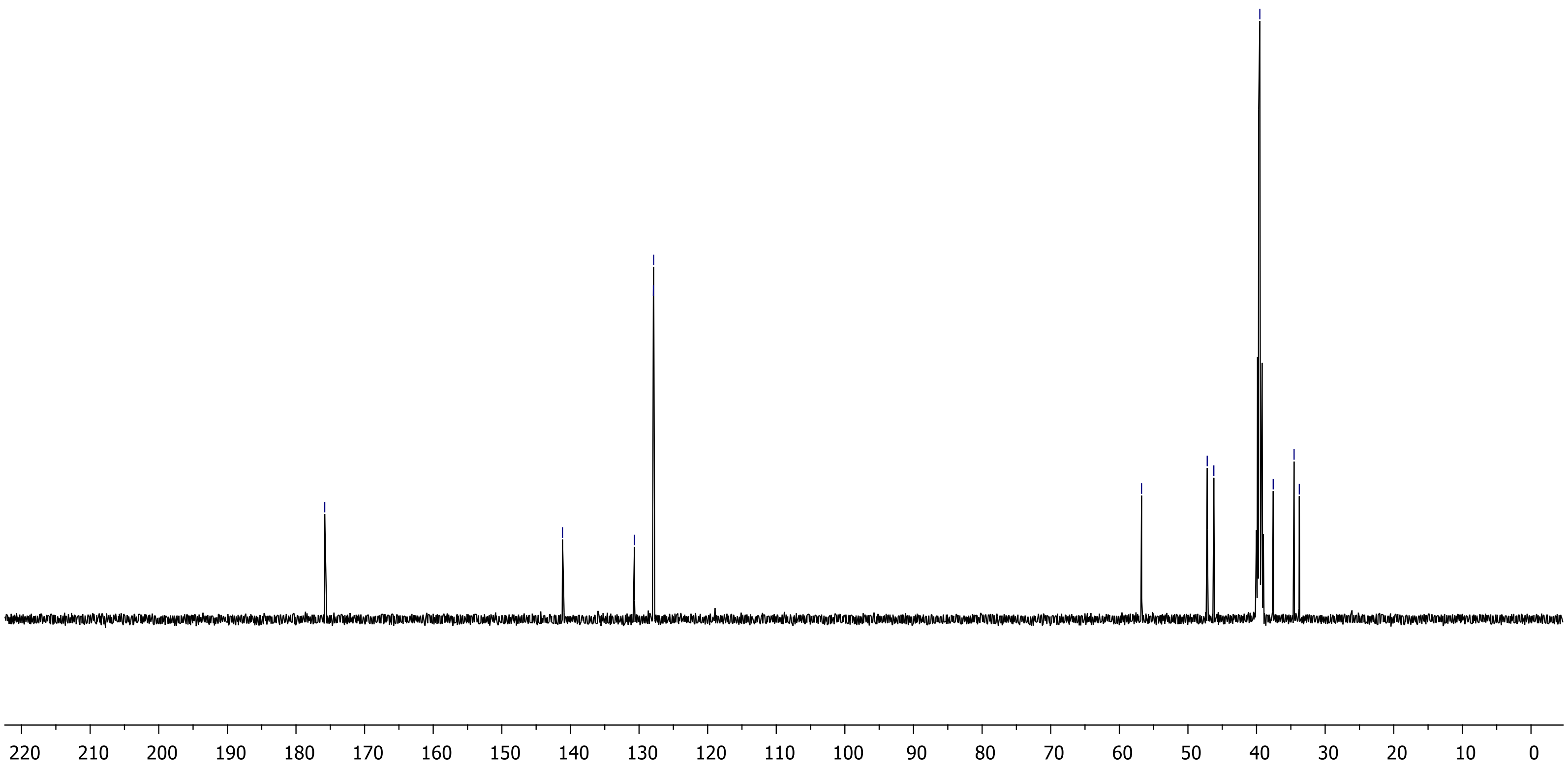

Ethyl-3-(4-bromophenyl)but-2-enoate

^{1}H NMR (500 MHz, $CDCl_3$)

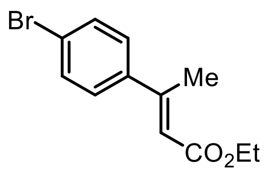

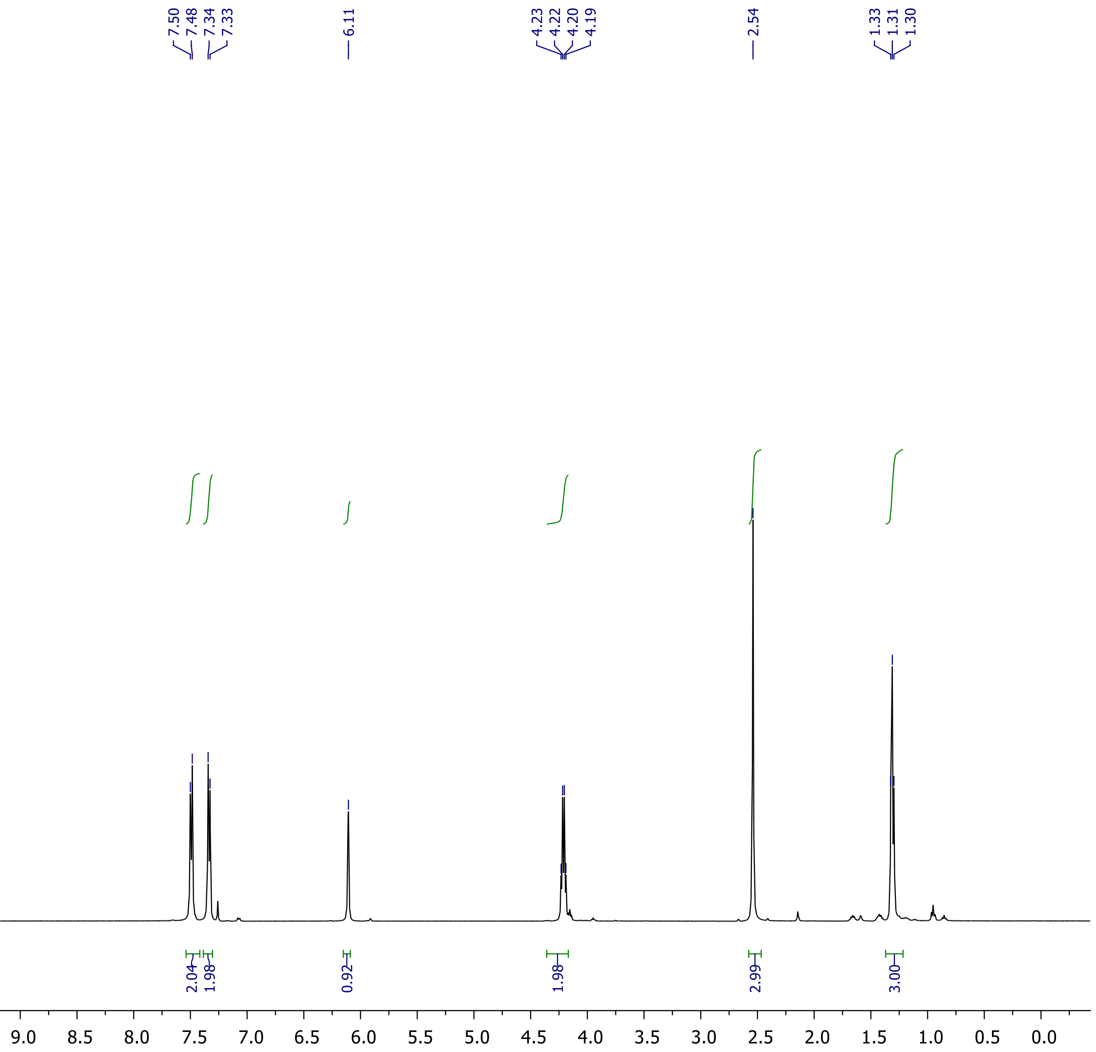

$^{13}C\{^{1}H\}$ NMR (101 MHz, $CDCl_3$)

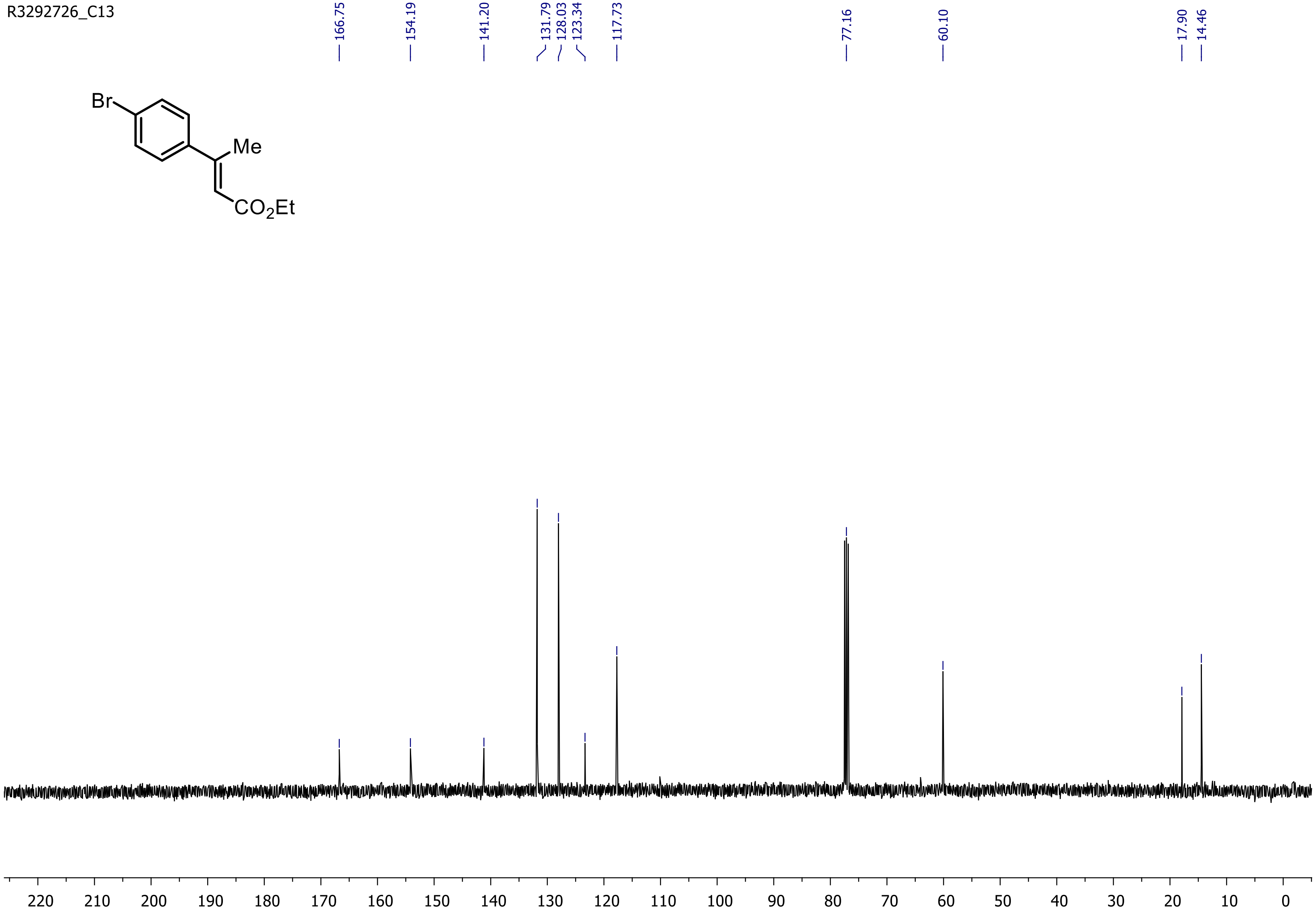

Compound 6

^{1}H NMR (500 MHz, CDCl$_3$)

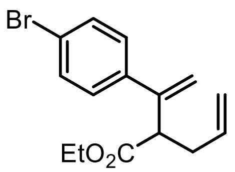

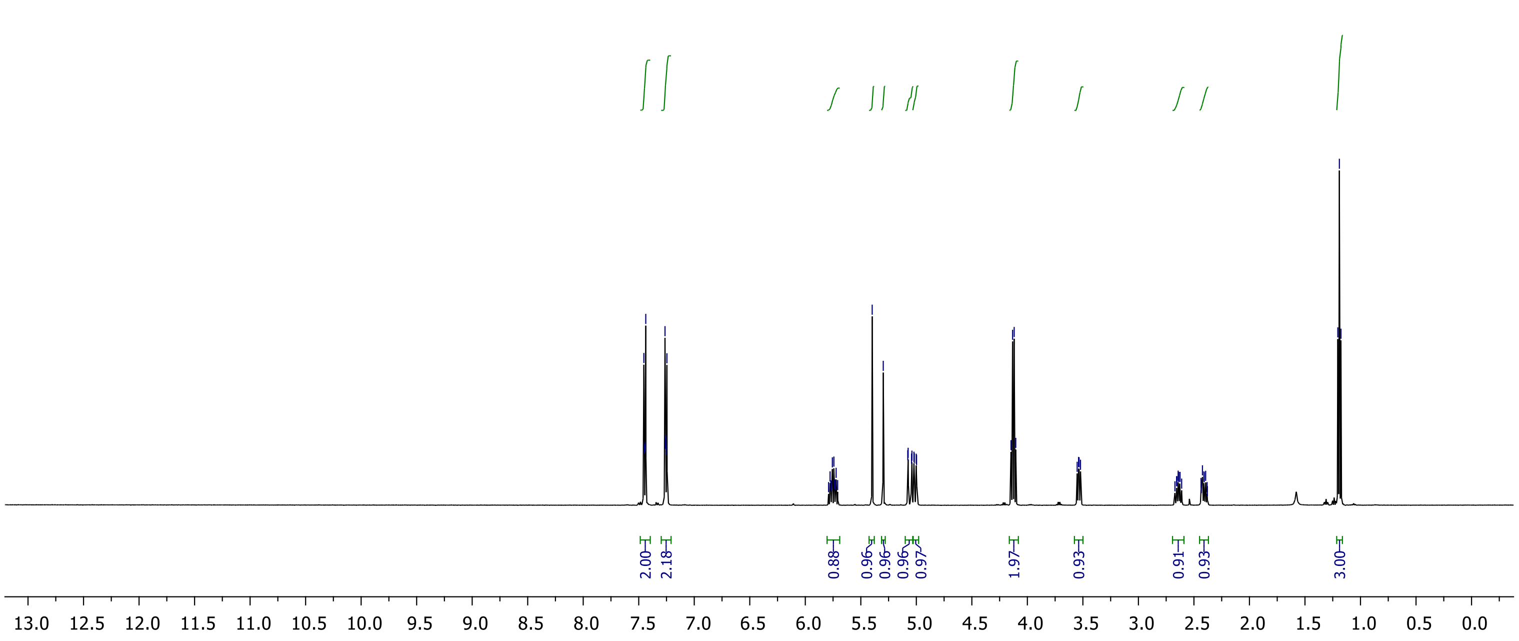

$^{13}C\{^{1}H\}$ NMR (126 MHz, $CDCl_3$)

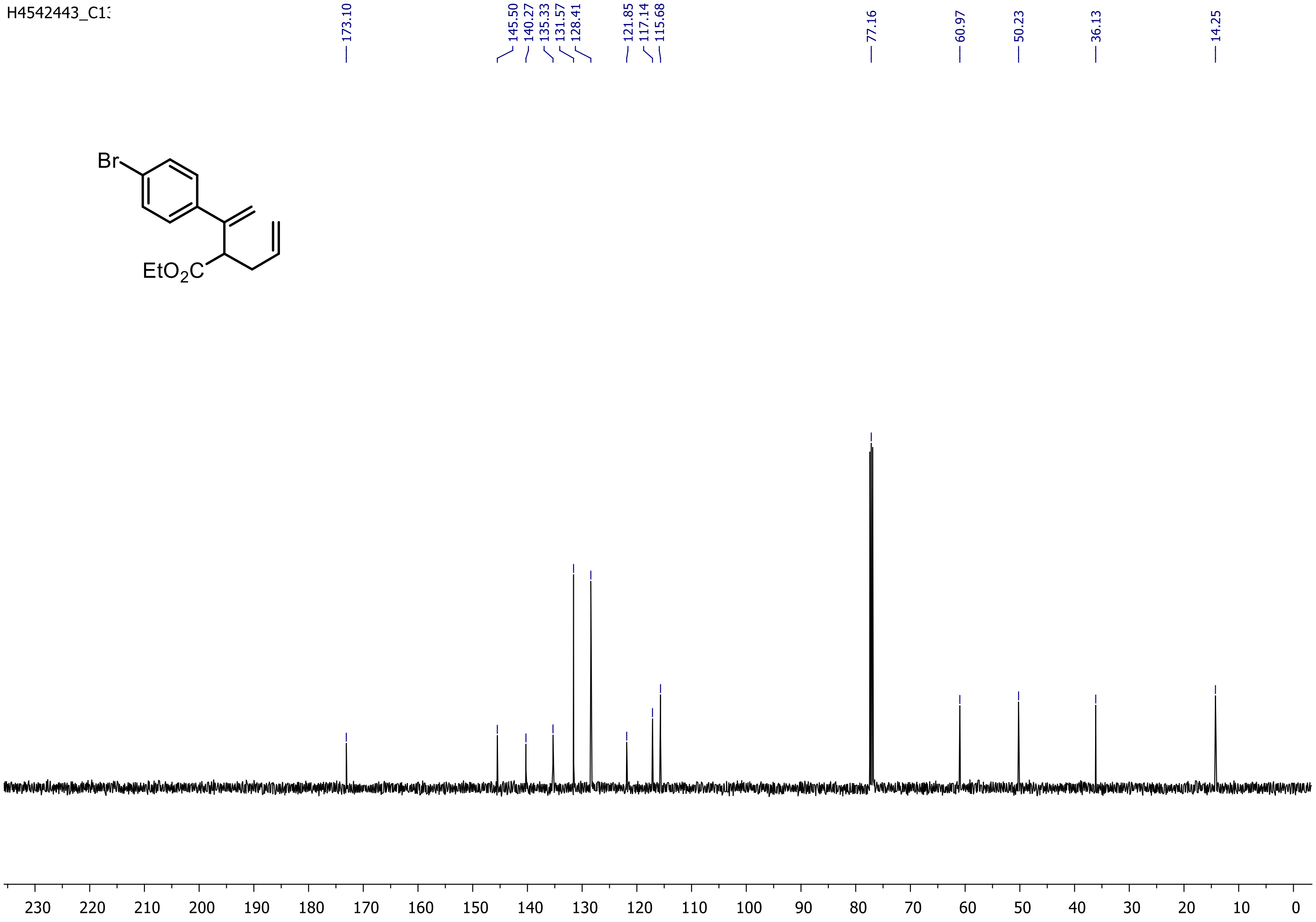

Compound (±)-6a

^{1}H NMR (500 MHz, $CDCl_3$)

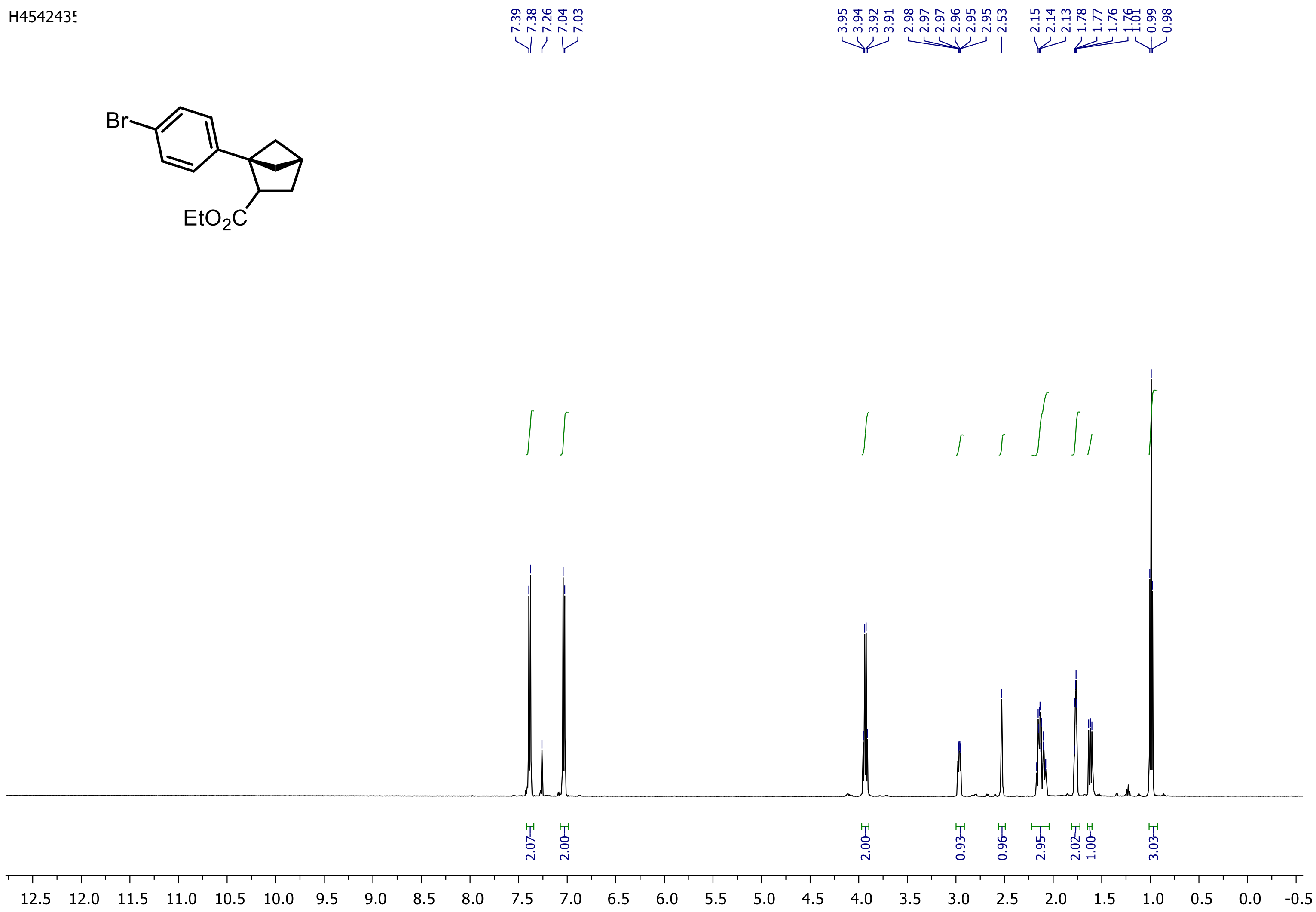

$^{13}C\{^{1}H\}$ NMR (126 MHz, $CDCl_3$)

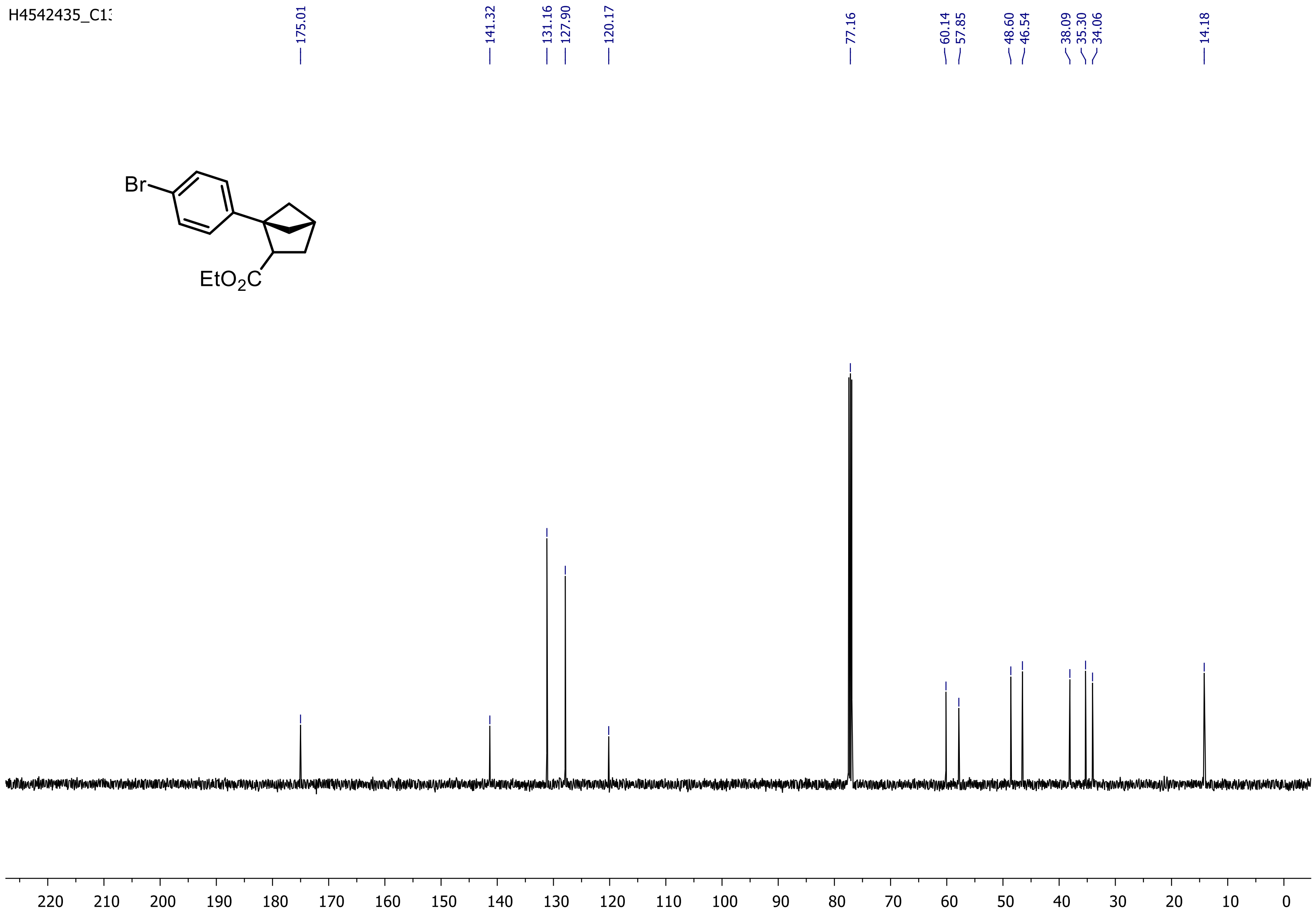

Compound (±)-6b

^{1}H NMR (500 MHz, DMSO-d$_6$)

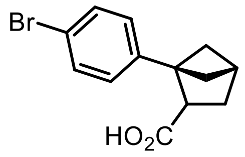

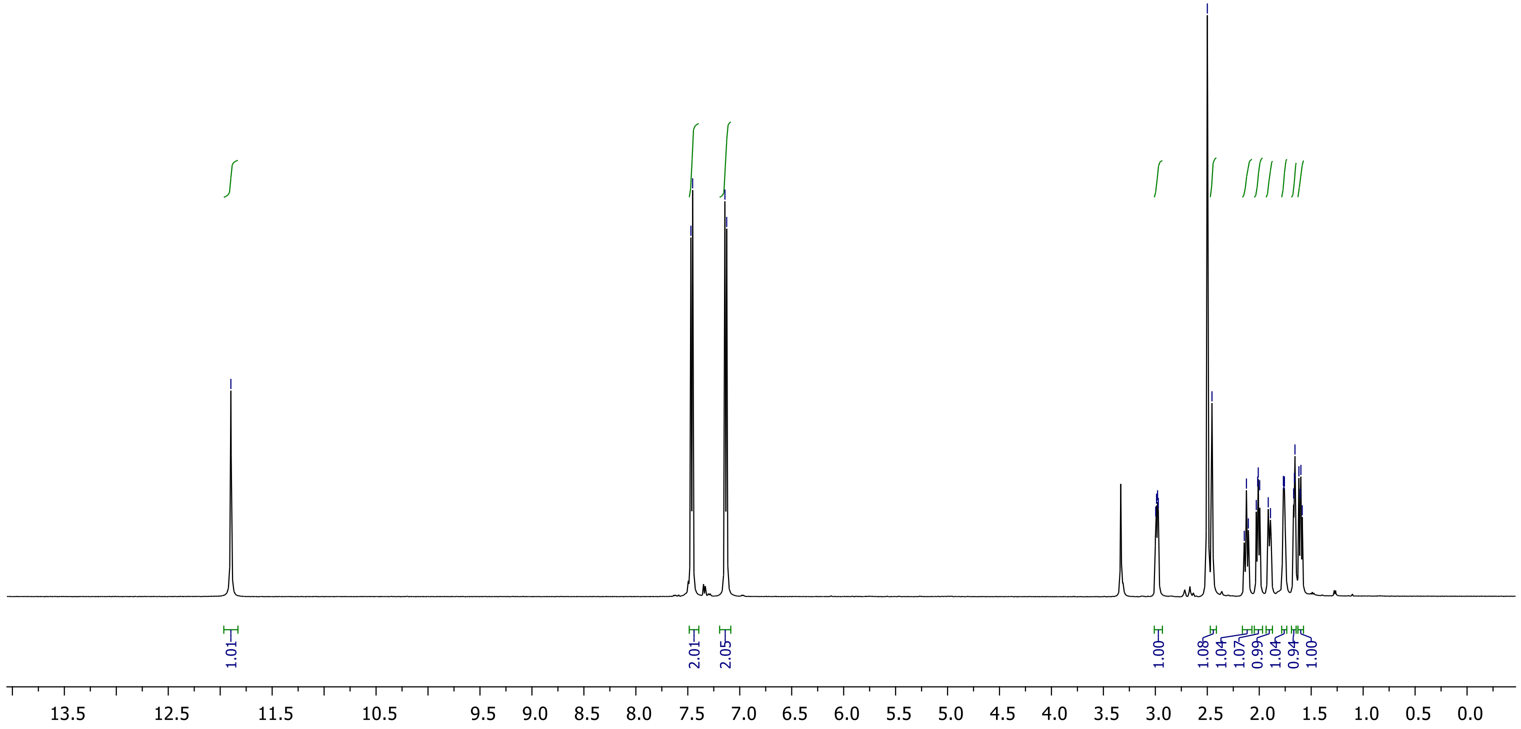

$^{13}C\{^1H\}$ NMR (101 MHz, DMSO-d_6)

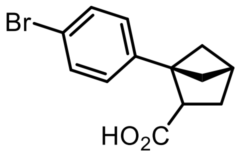

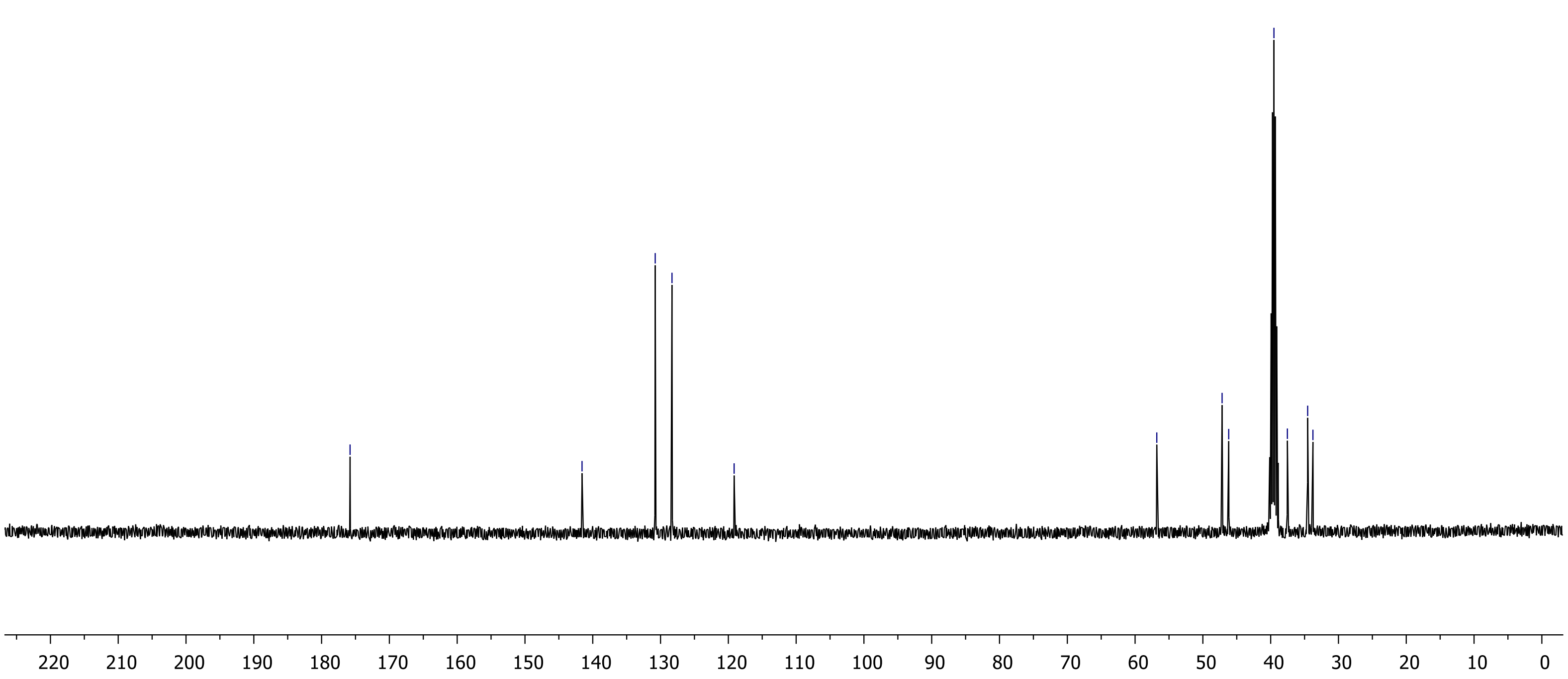

Ethyl-3-(*p*-tolyl)but-2-enoate

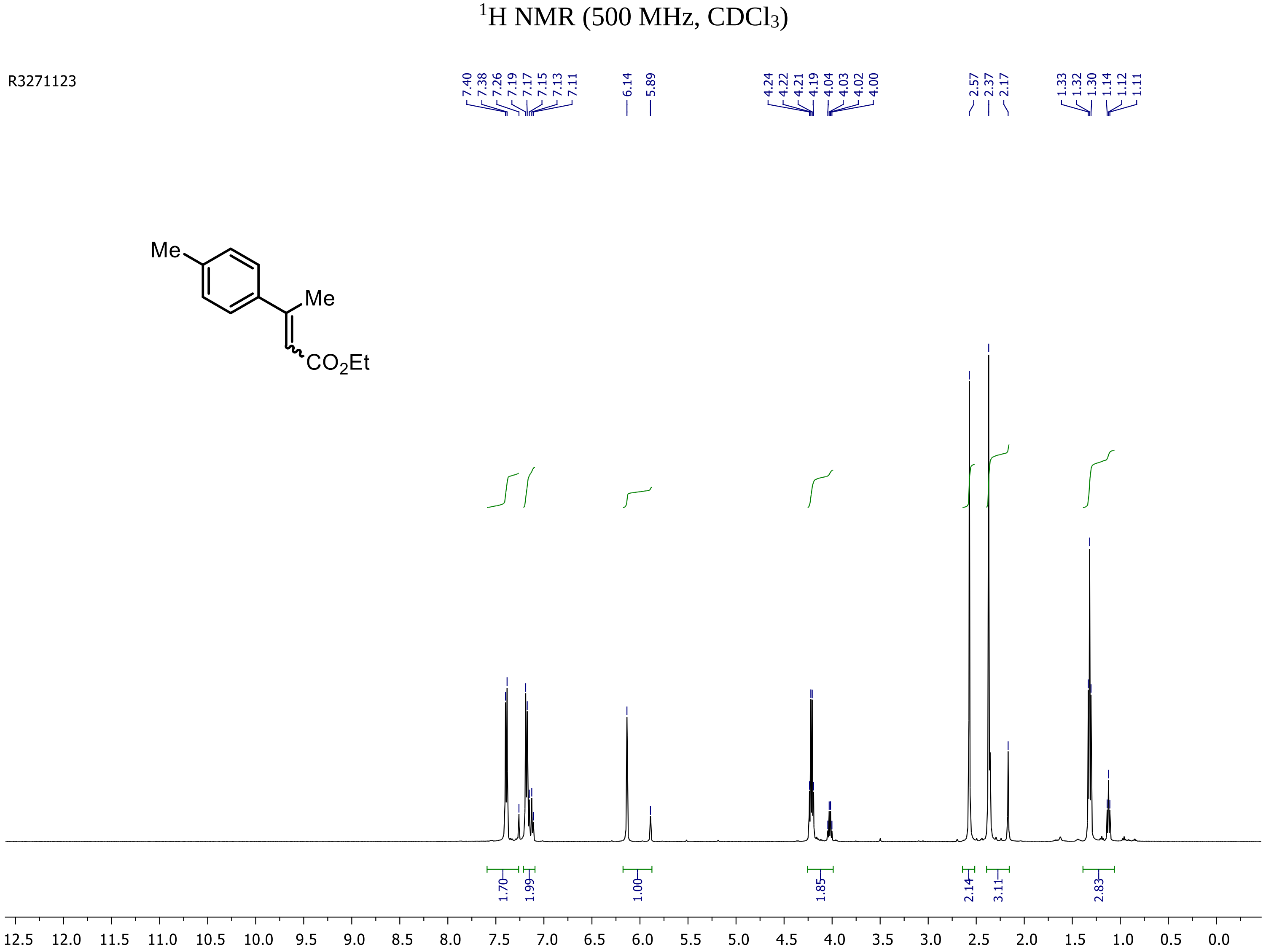

$^{13}C\{^1H\}$ NMR (126 MHz, $CDCl_3$)

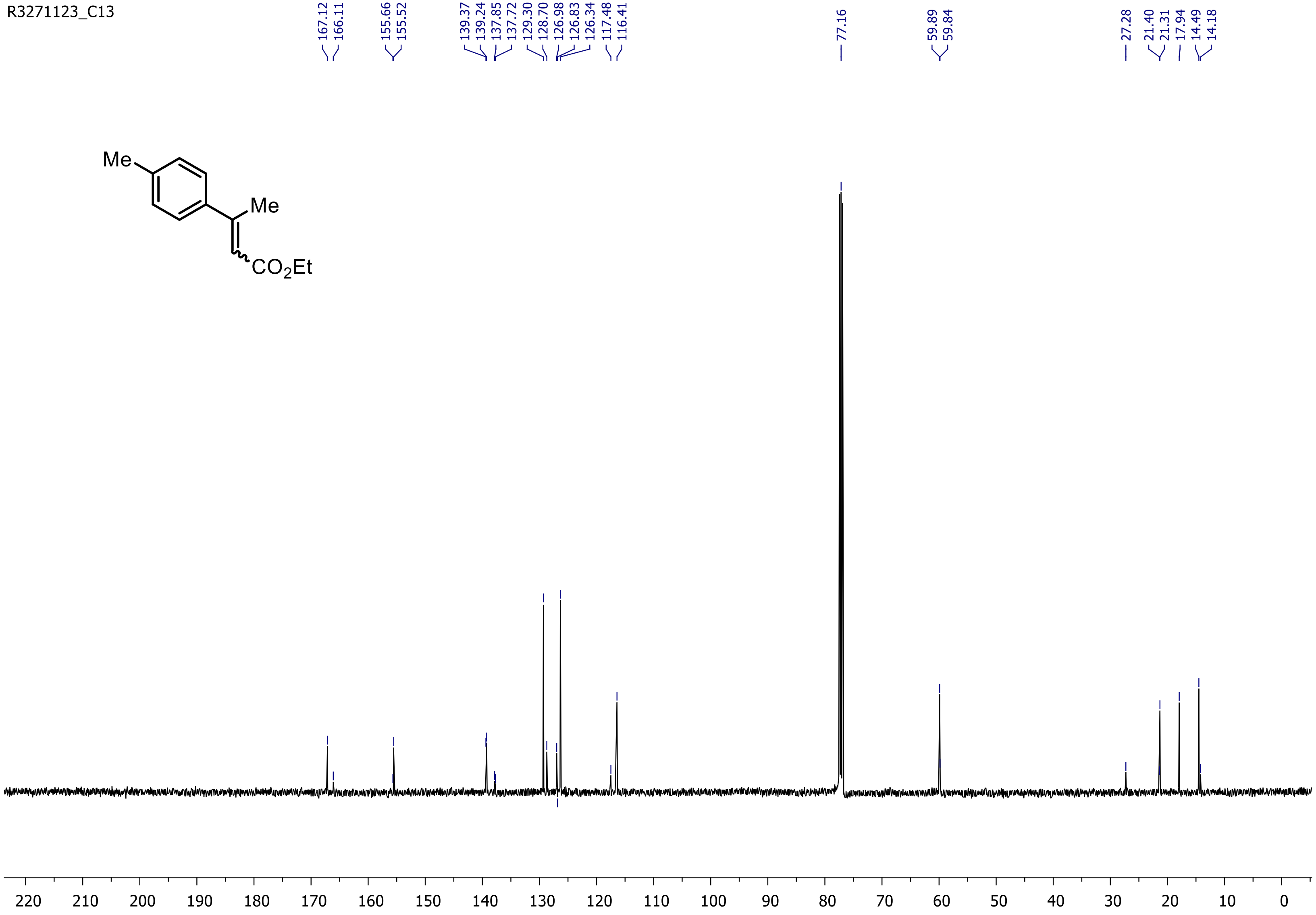

Compound 7

^{1}H NMR (500 MHz, $CDCl_3$)

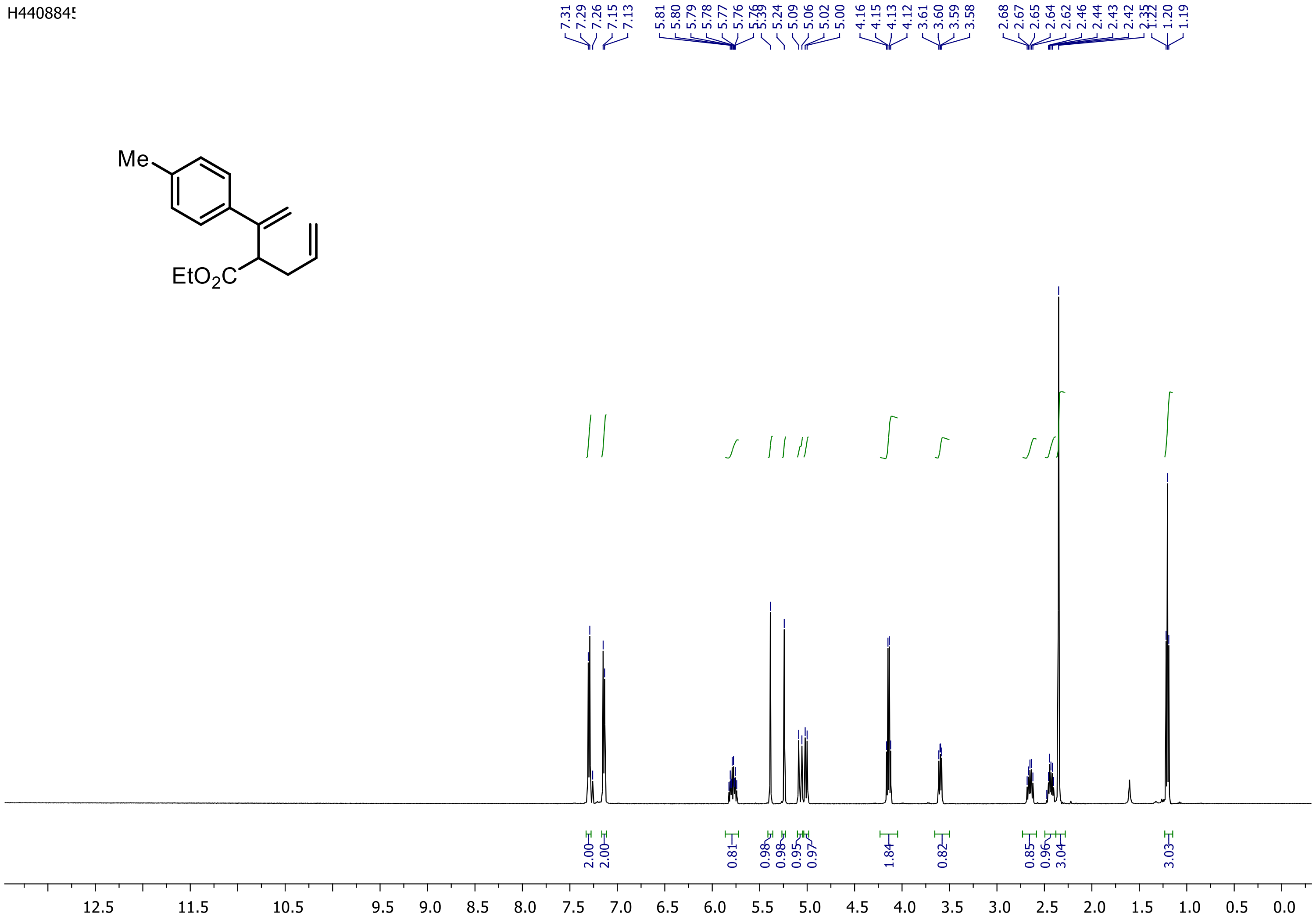

$^{13}C\{^{1}H\}$ NMR (126 MHz, $CDCl_3$)

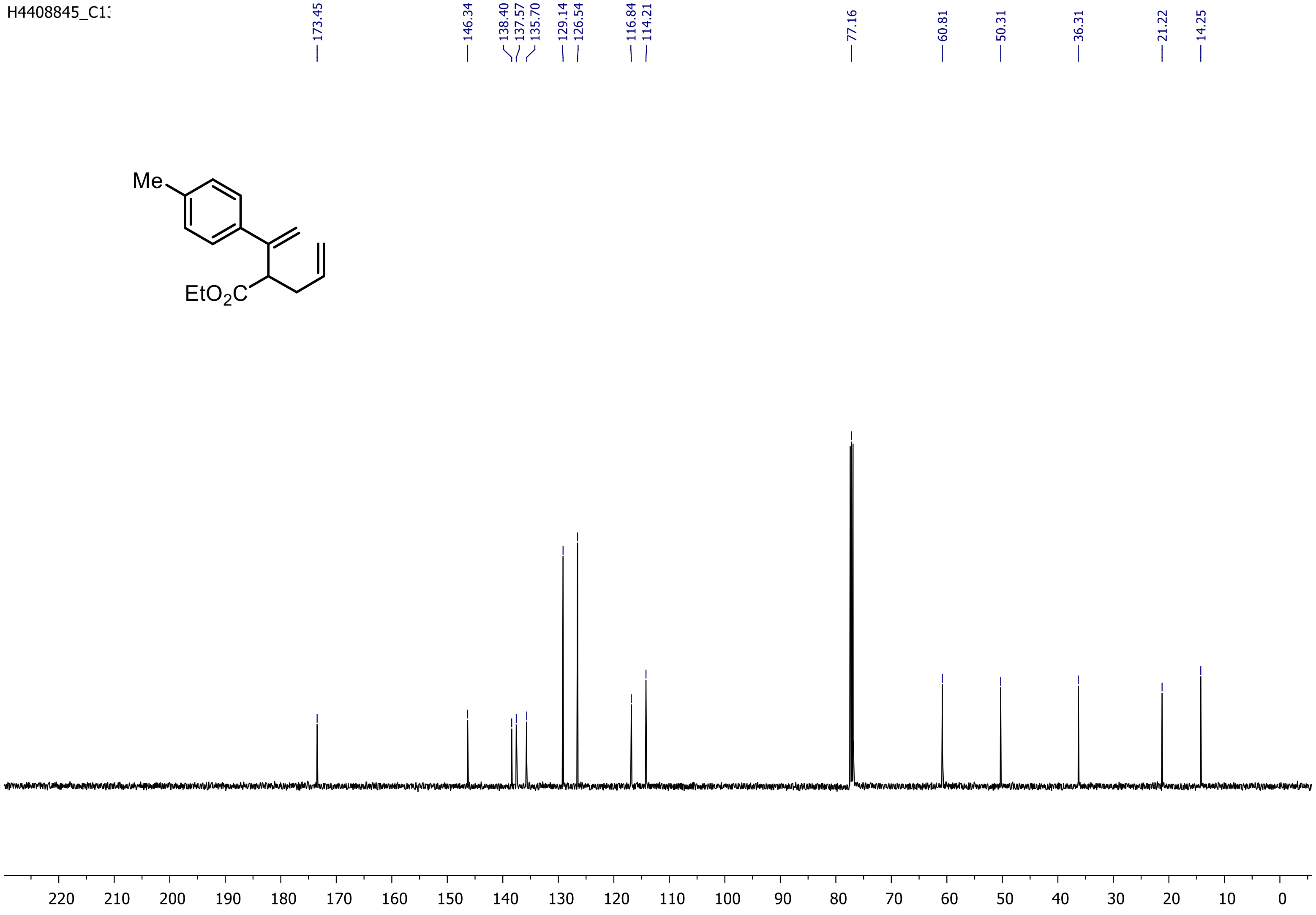

Compound (±)-7a

^{1}H NMR (500 MHz, $CDCl_3$)

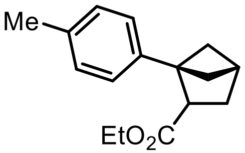

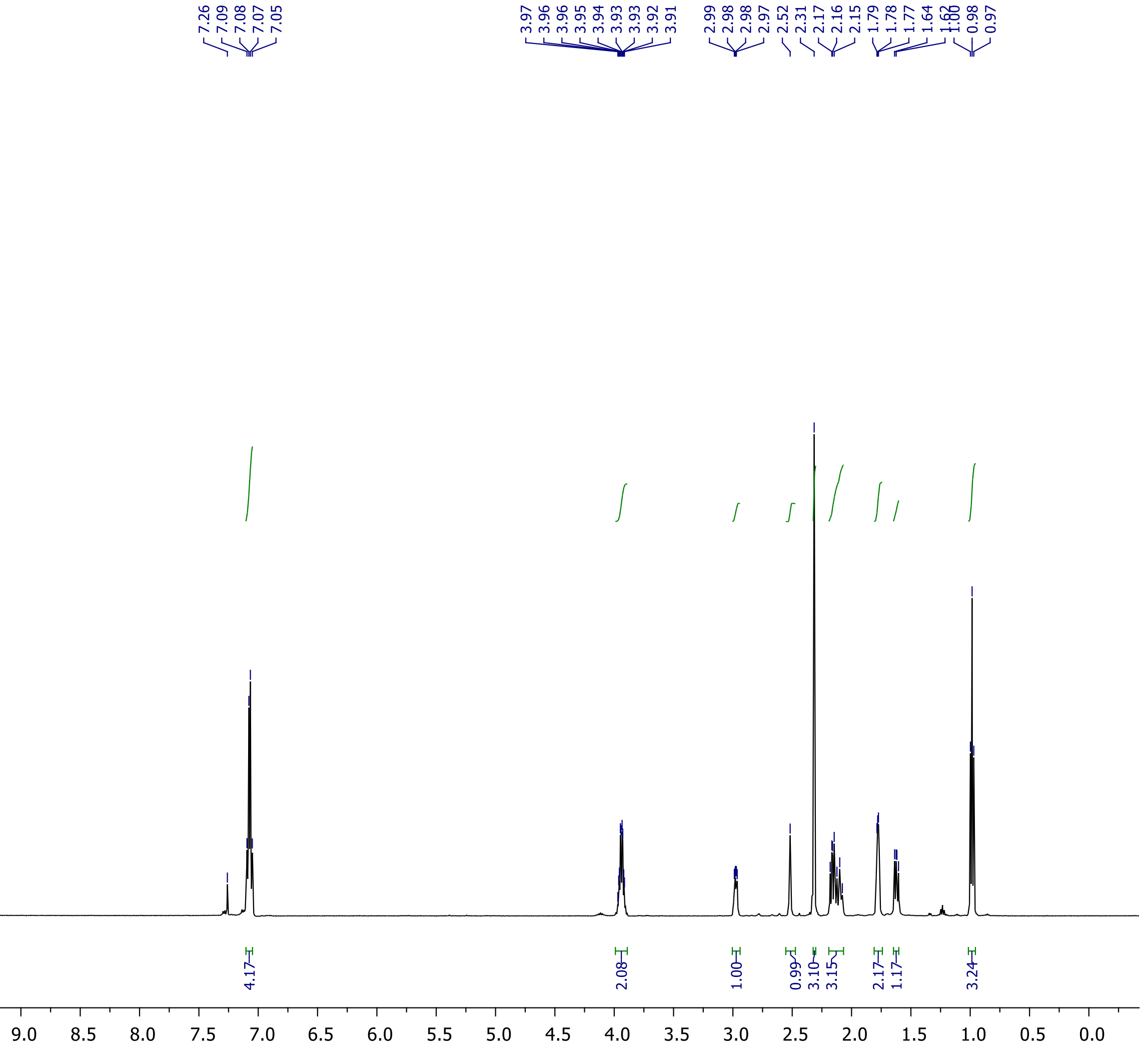

$^{13}C\{^{1}H\}$ NMR (126 MHz, $CDCl_3$)

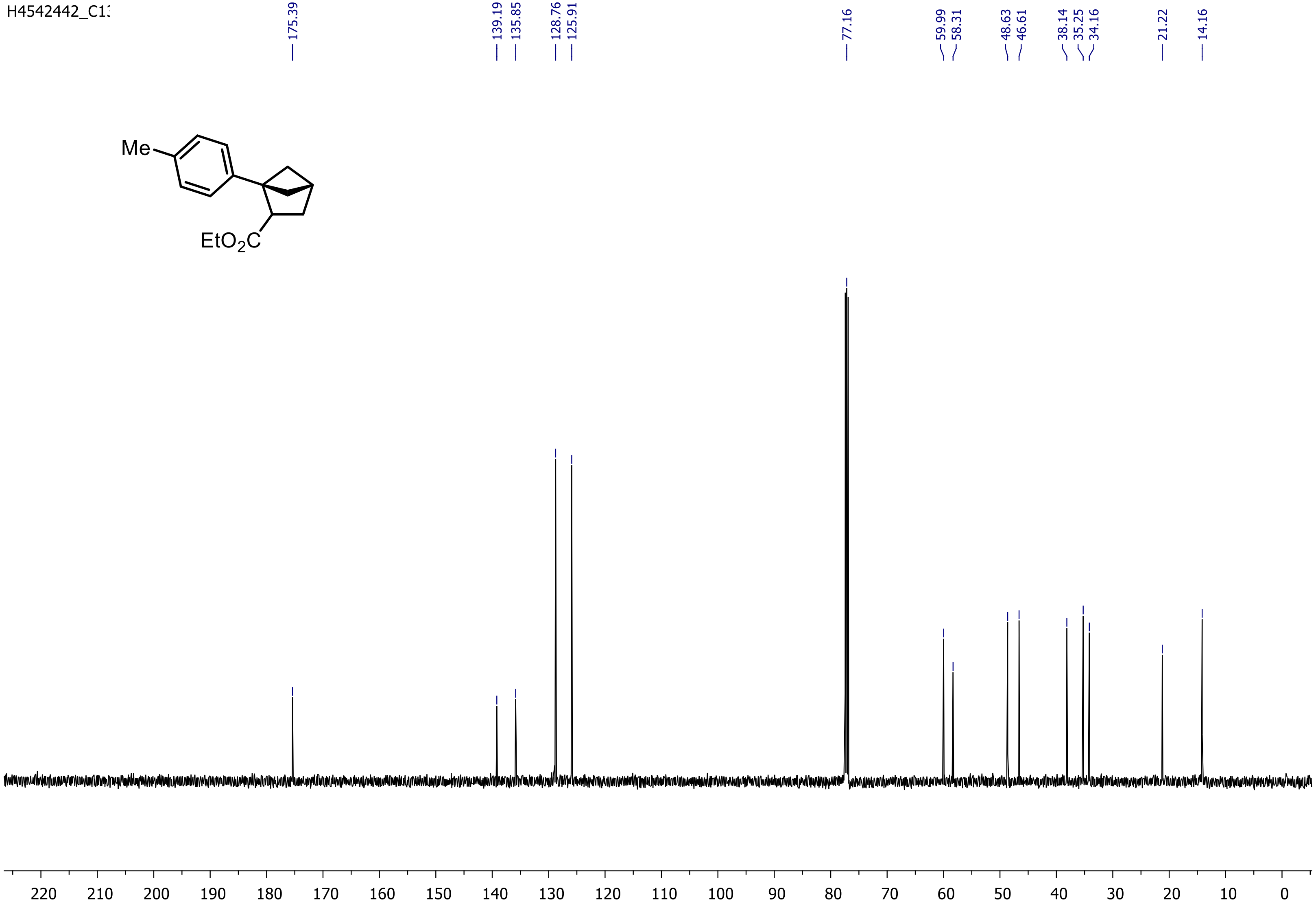

Compound (±)-7b

^{1}H NMR (500 MHz, DMSO-d$_6$)

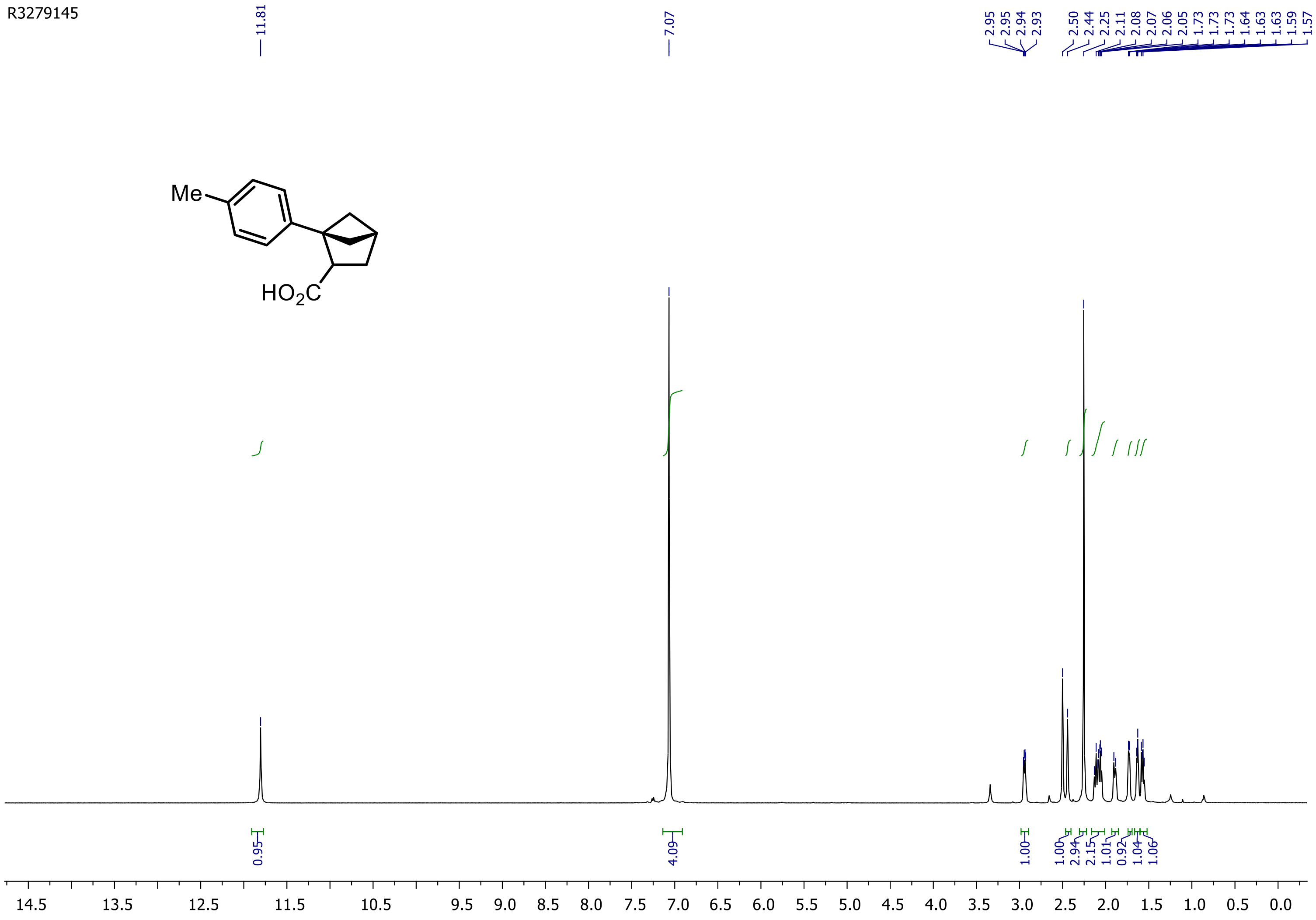

$^{13}C\{^{1}H\}$ NMR (126 MHz, DMSO-d_6)

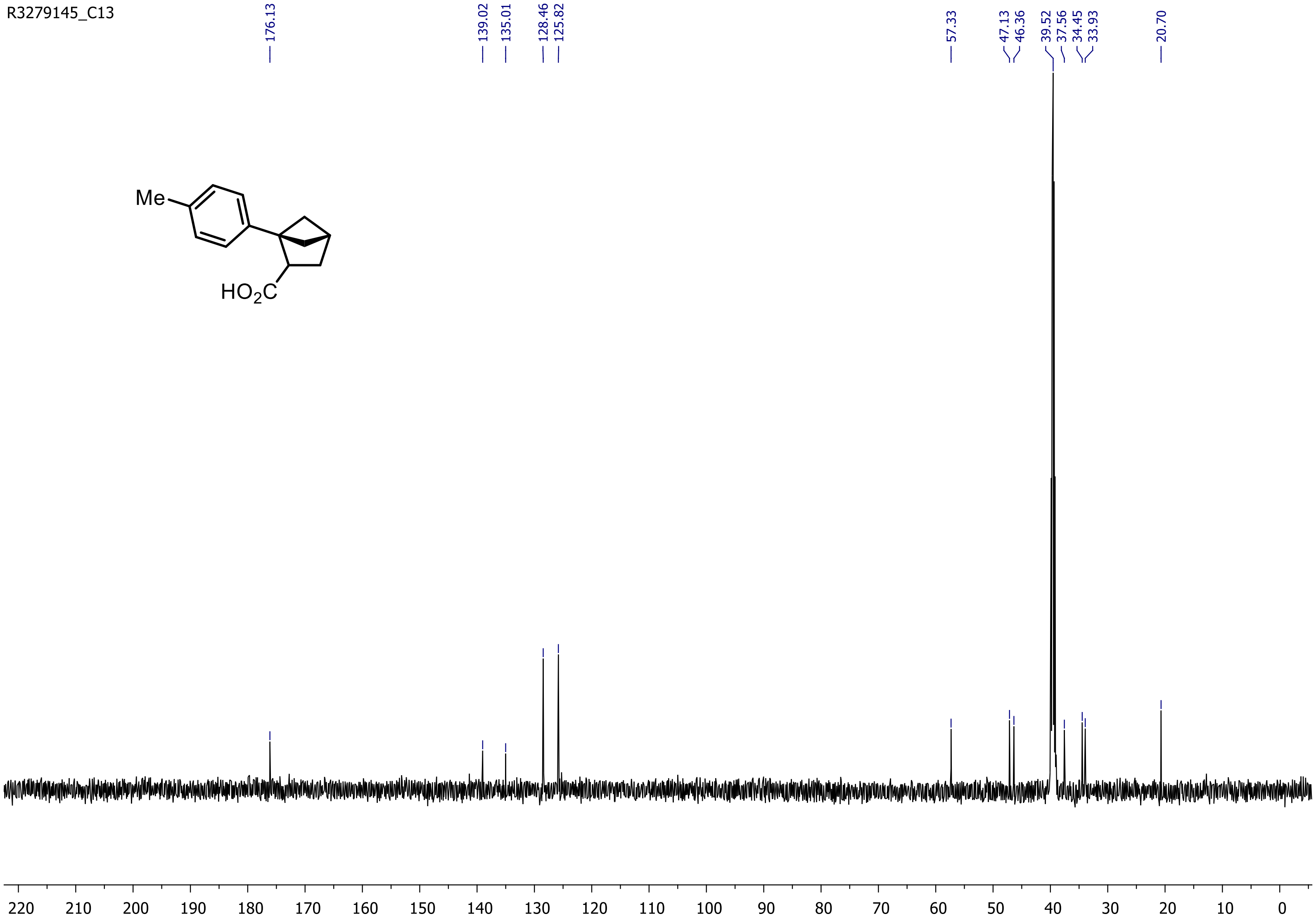

Ethyl-3-(4-(trifluoromethyl)phenyl)but-2-enoate

^{1}H NMR (500 MHz, $CDCl_3$)

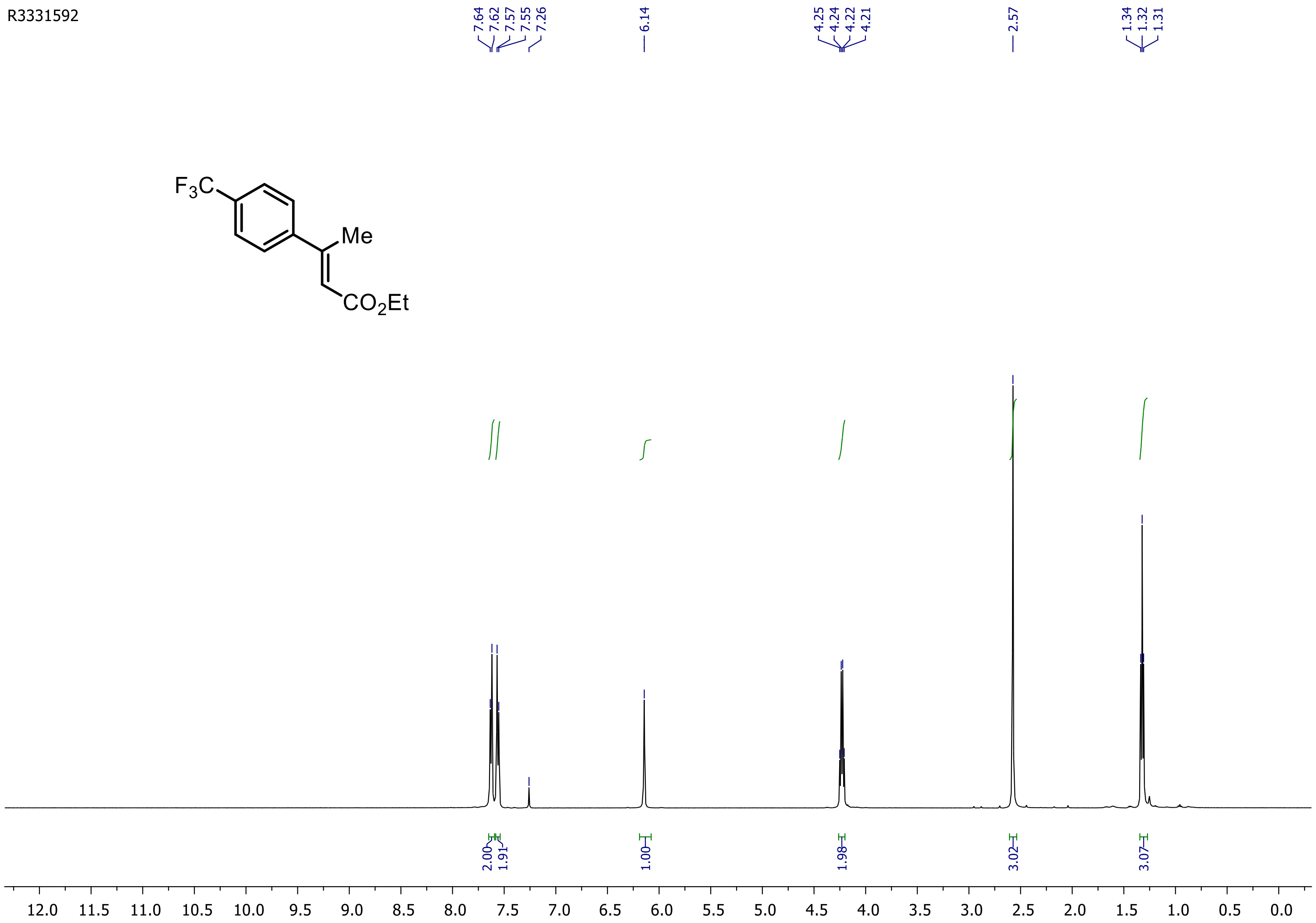

$^{13}C\{^1H\}$ NMR (126 MHz, $CDCl_3$)

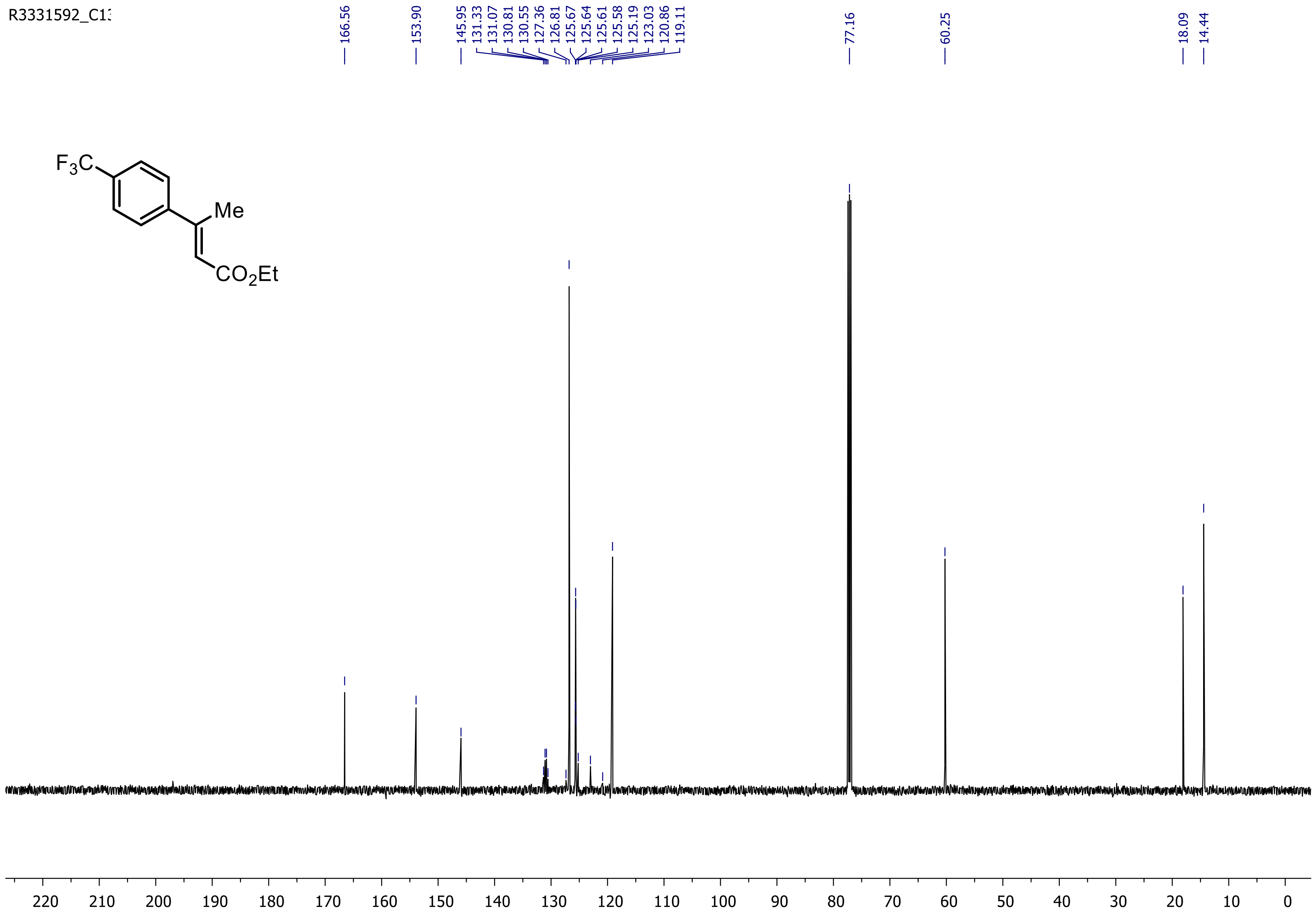

$^{19}F\{^{1}H\}$ NMR (376 MHz, $CDCl_3$)

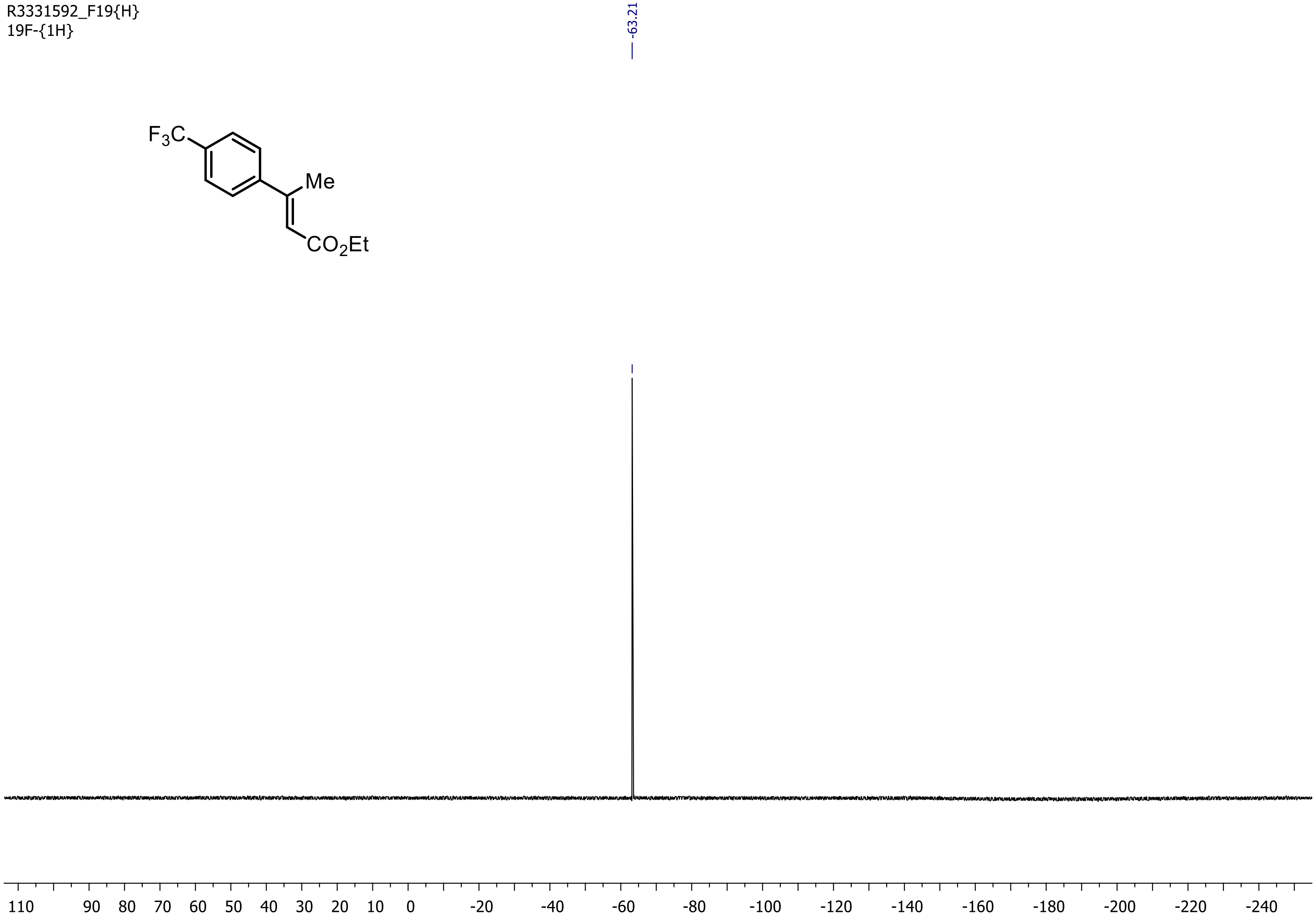

Compound 8

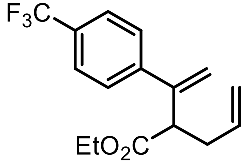

^{1}H NMR (500 MHz, $CDCl_3$)

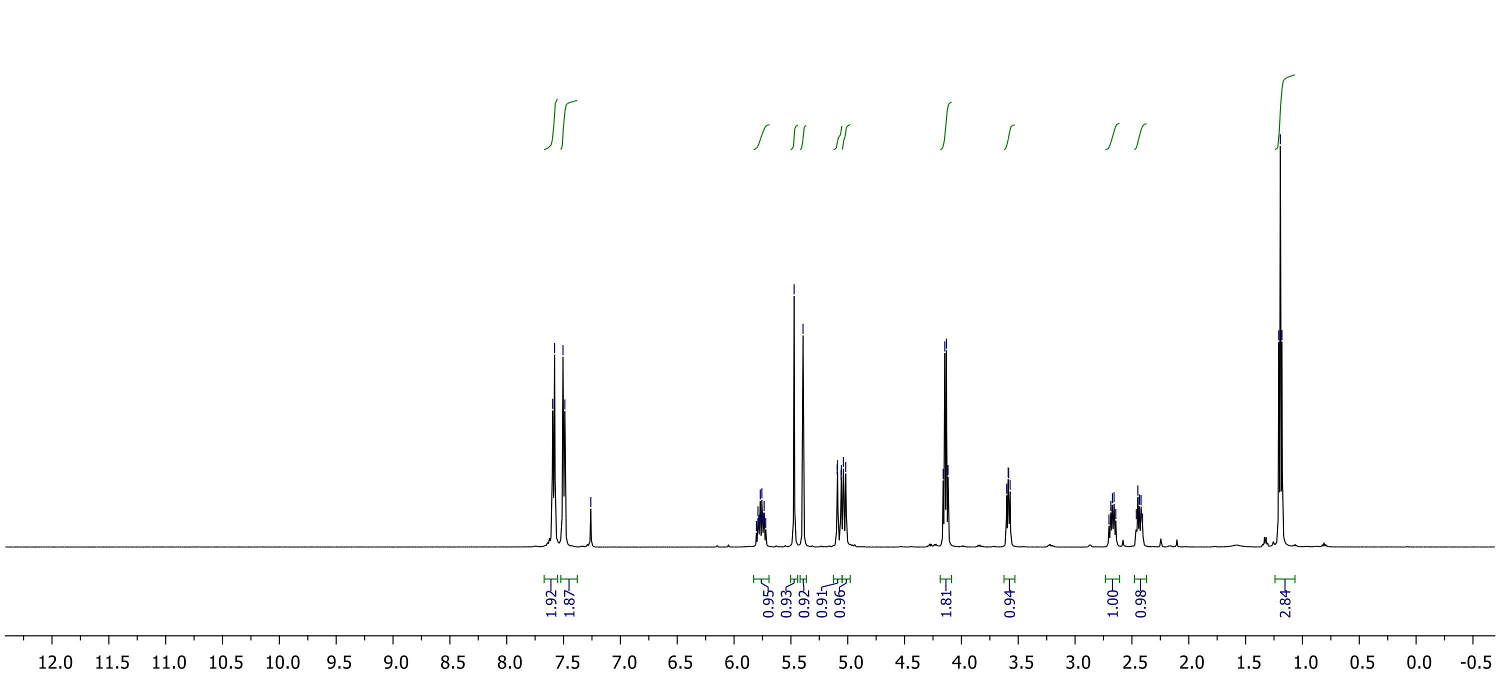

$^{13}C\{^1H\}$ NMR (151 MHz, $CDCl_3$)

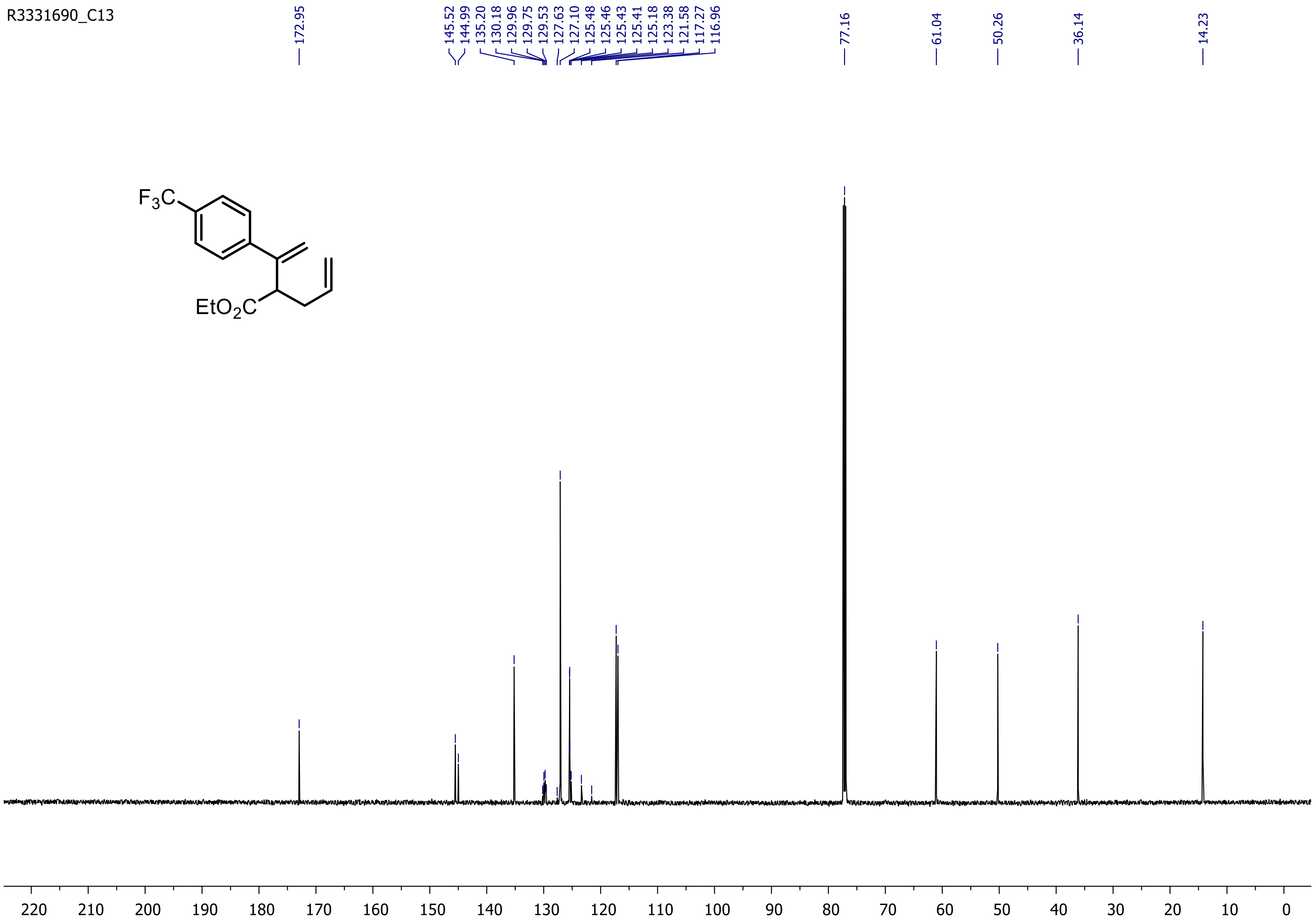

$^{19}F\{^{1}H\}$ NMR (376 MHz, $CDCl_3$)

R3331690_F19{H}
19F-{1H}

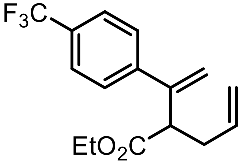

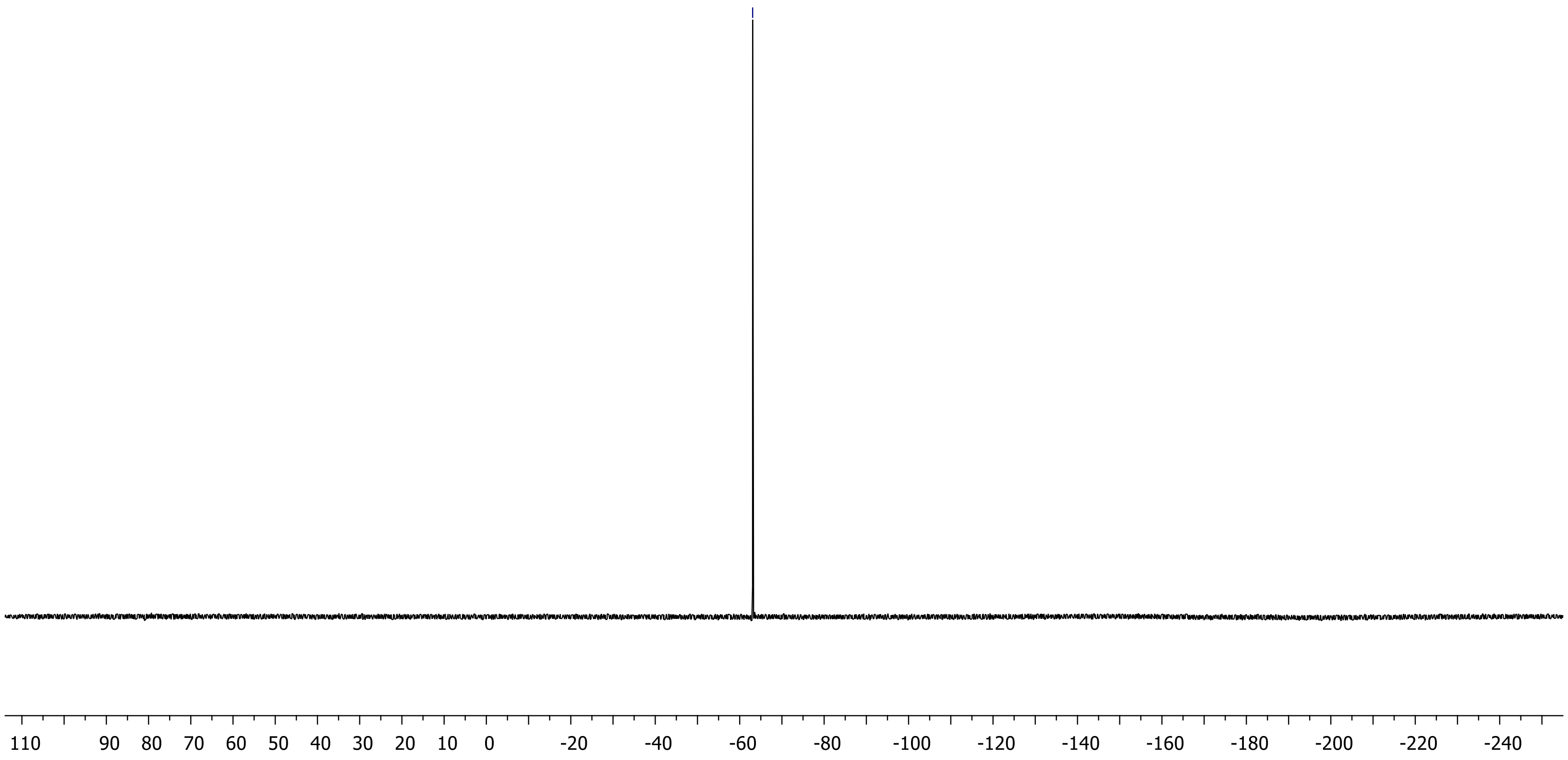

Compound (±)-8a (the sample contains ca. 10% of the isomeric 1,5-disubstituted bicyclo[2.1.1]hexane)

^{1}H NMR (500 MHz, $CDCl_3$)

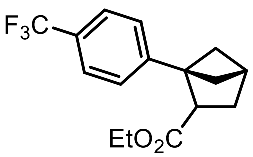

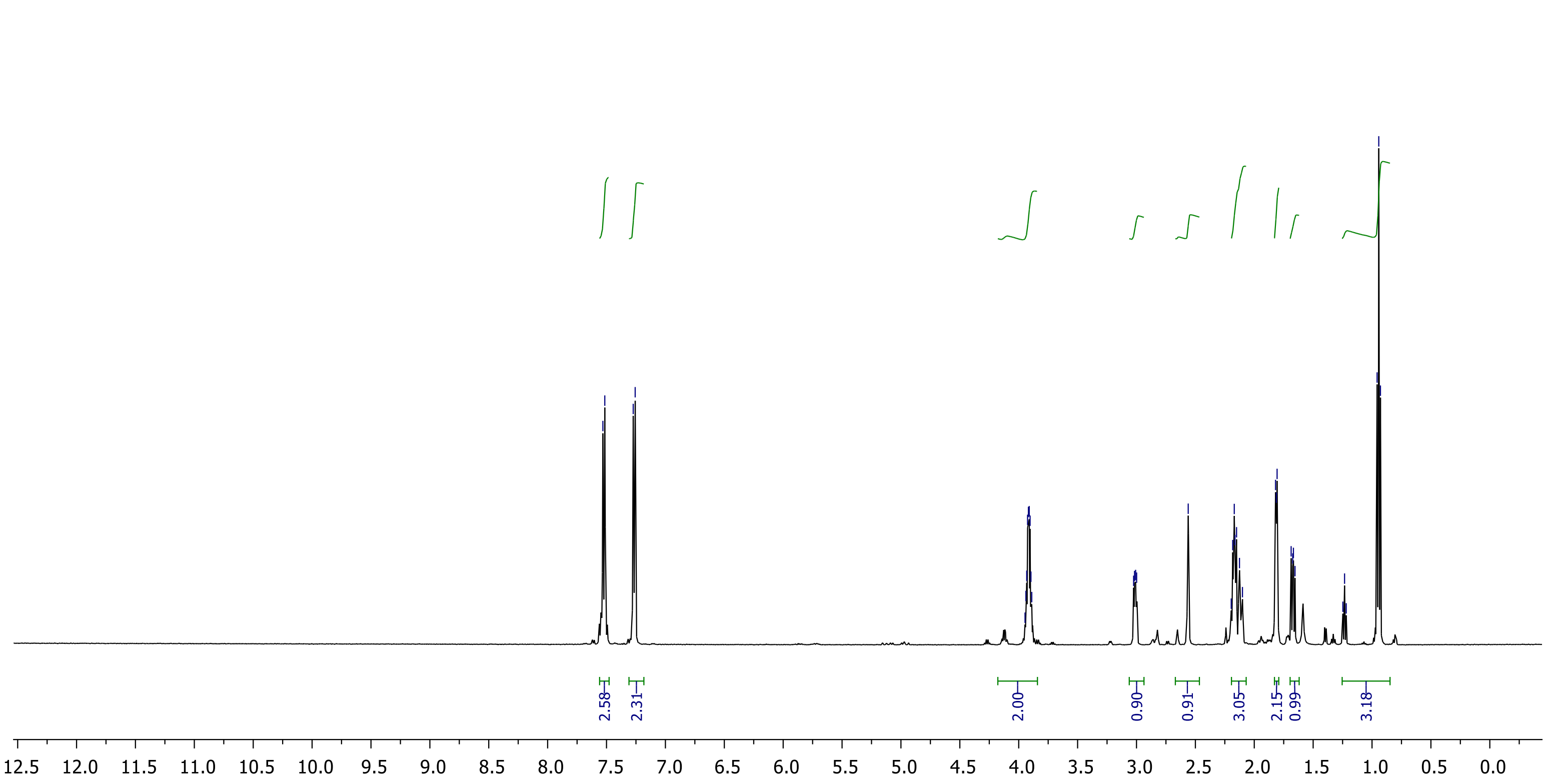

$^{13}C\{^1H\}$ NMR (126 MHz, $CDCl_3$)

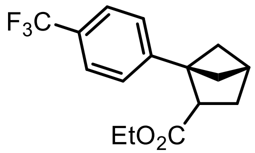

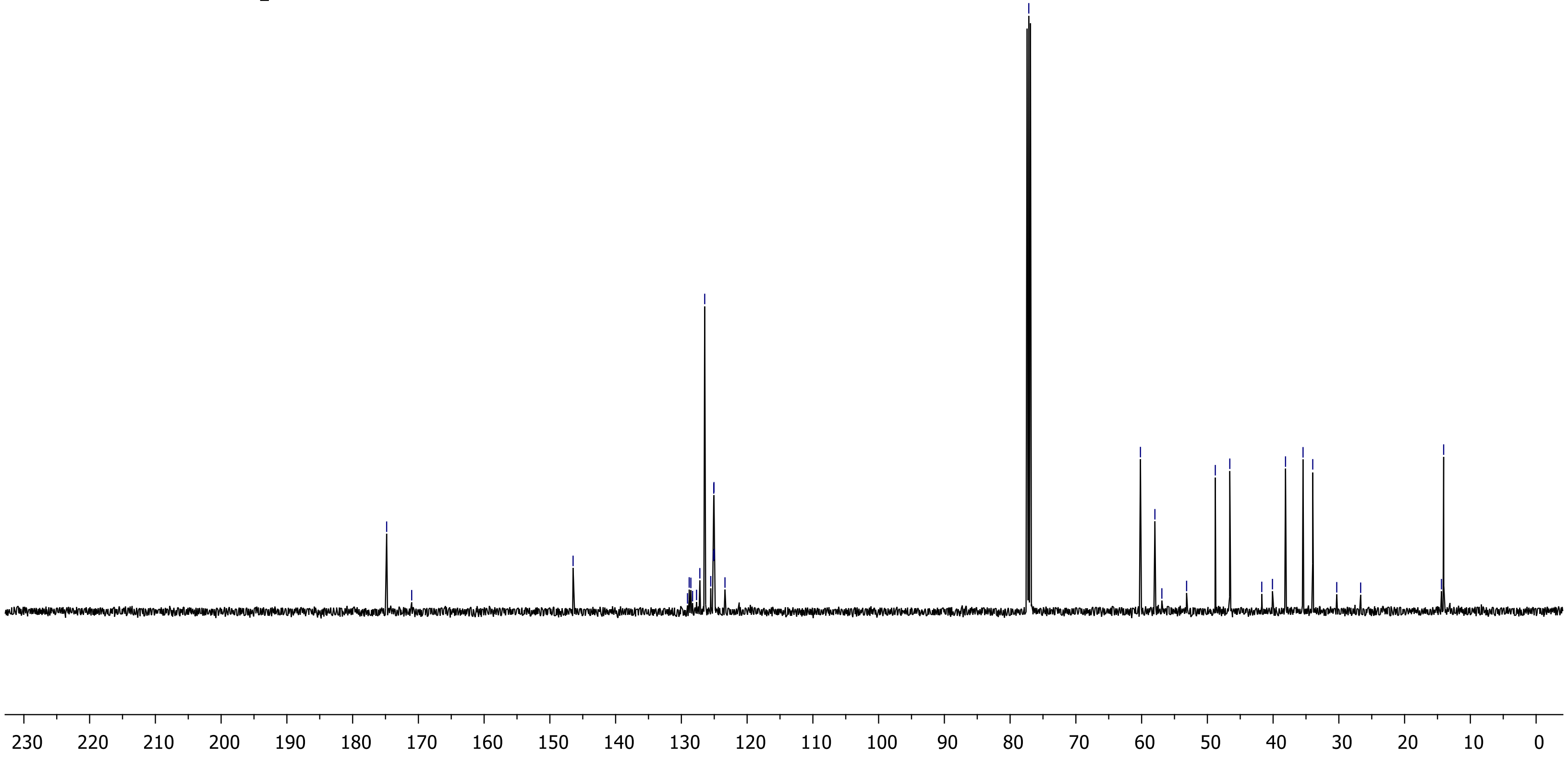

$^{19}F\{^{1}H\}$ NMR (376 MHz, $CDCl_3$)

H4412819_F19{H}
19F-{1H}

-62.90

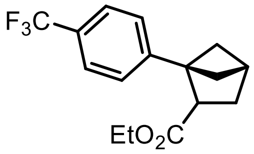

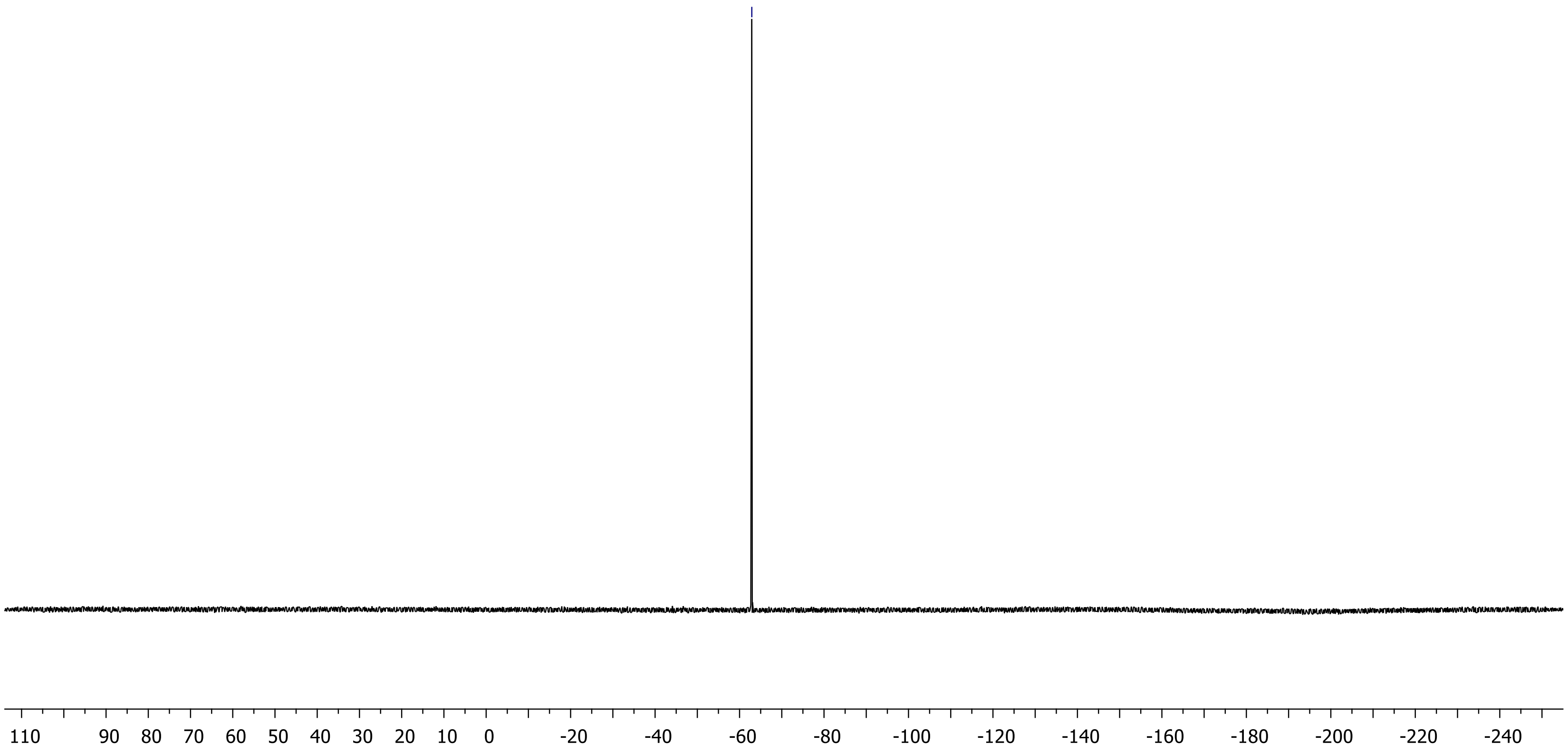

Compound (±)-8b

^{1}H NMR (500 MHz, DMSO-d_6)

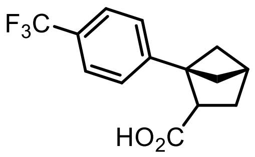

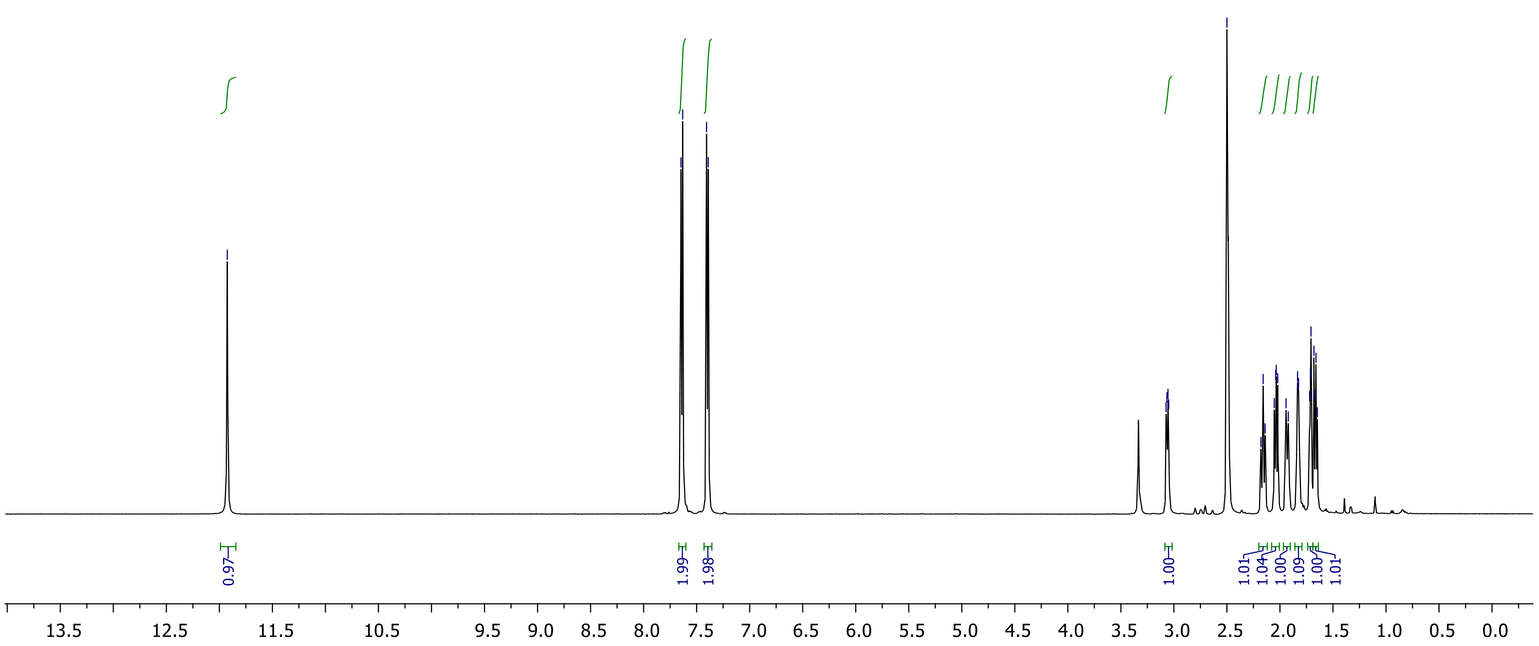

$^{13}C\{^{1}H\}$ NMR (151 MHz, DMSO-d$_6$)

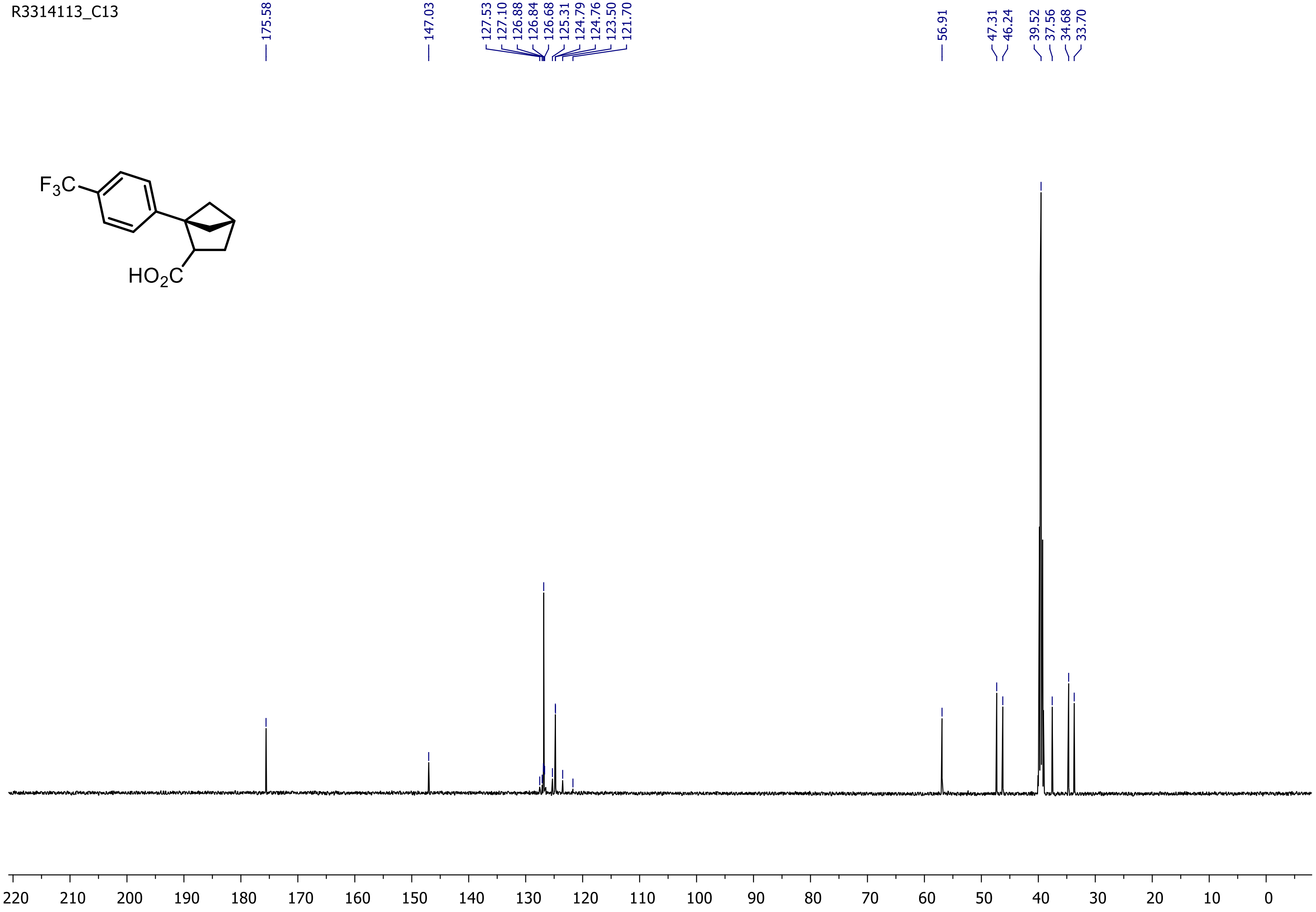

$^{19}F\{^{1}H\}$ NMR (376 MHz, DMSO-d_6)

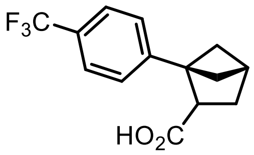

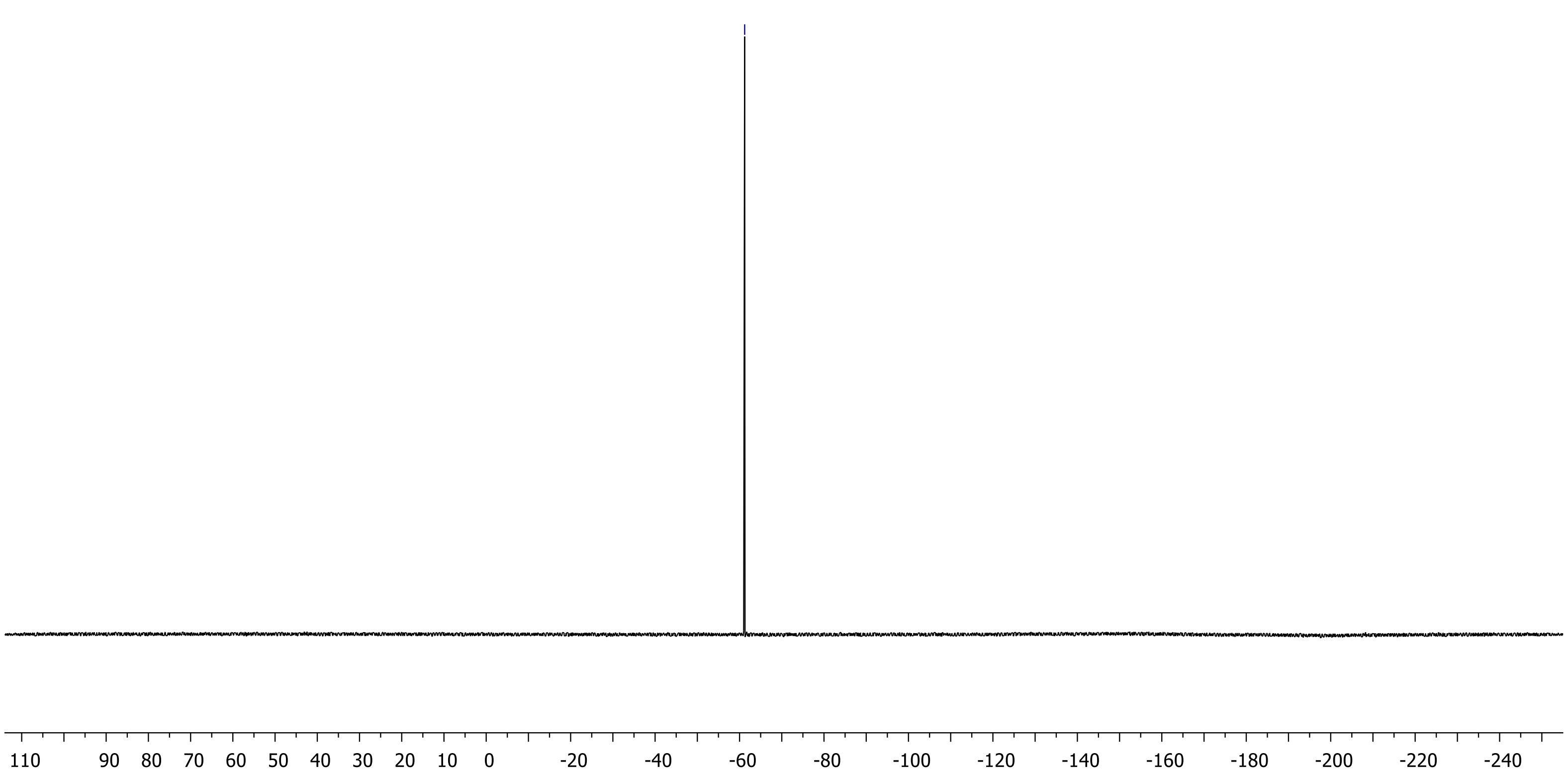

Ethyl-3-(4-methoxyphenyl)but-2-enoate

^{1}H NMR (500 MHz, $CDCl_3$)

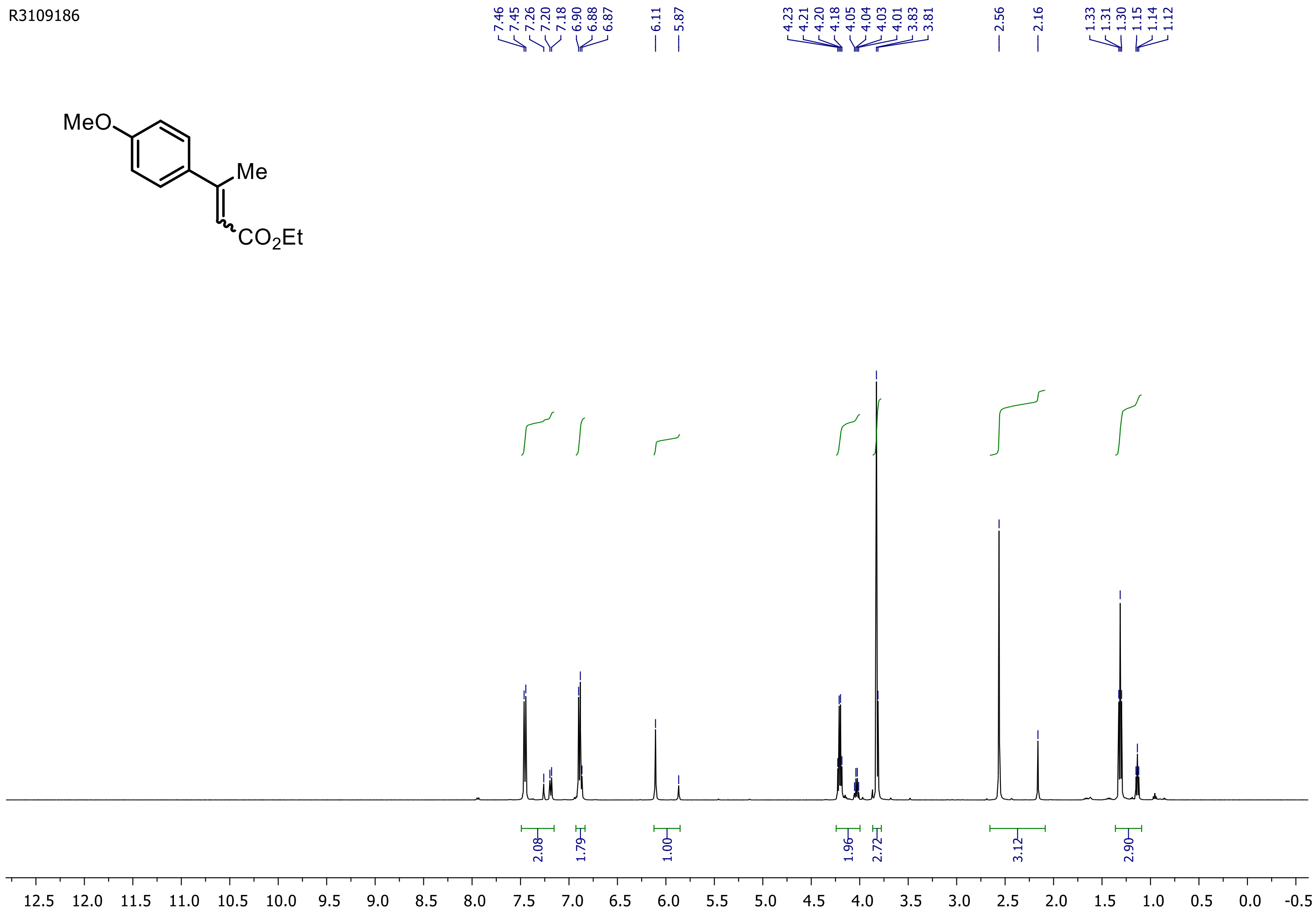

$^{13}C\{^1H\}$ NMR (151 MHz, $CDCl_3$)

R3109186_C13

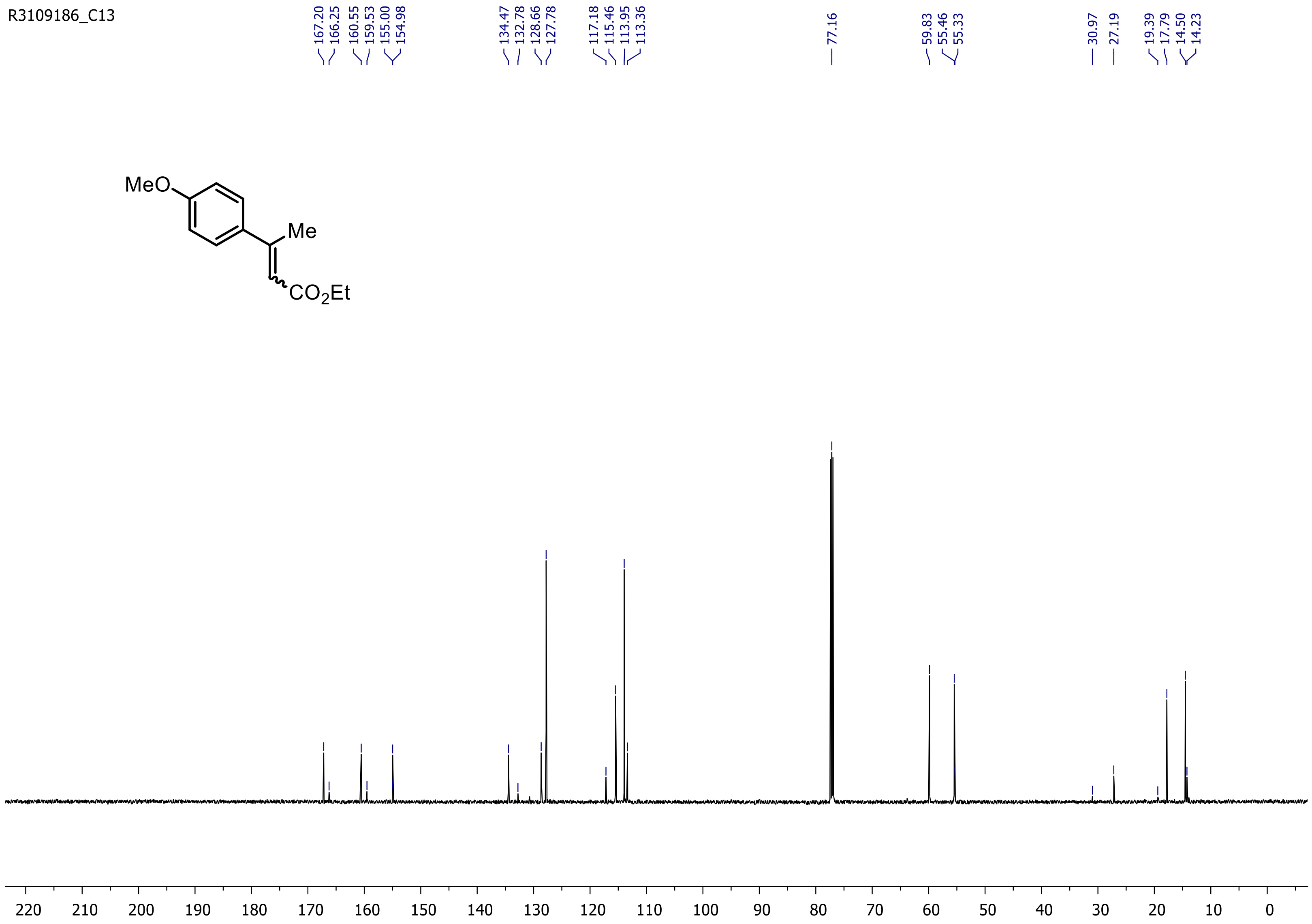

Compound 9

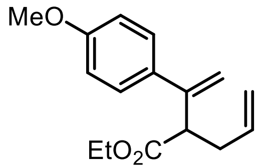

^1H NMR (500 MHz, $CDCl_3$)

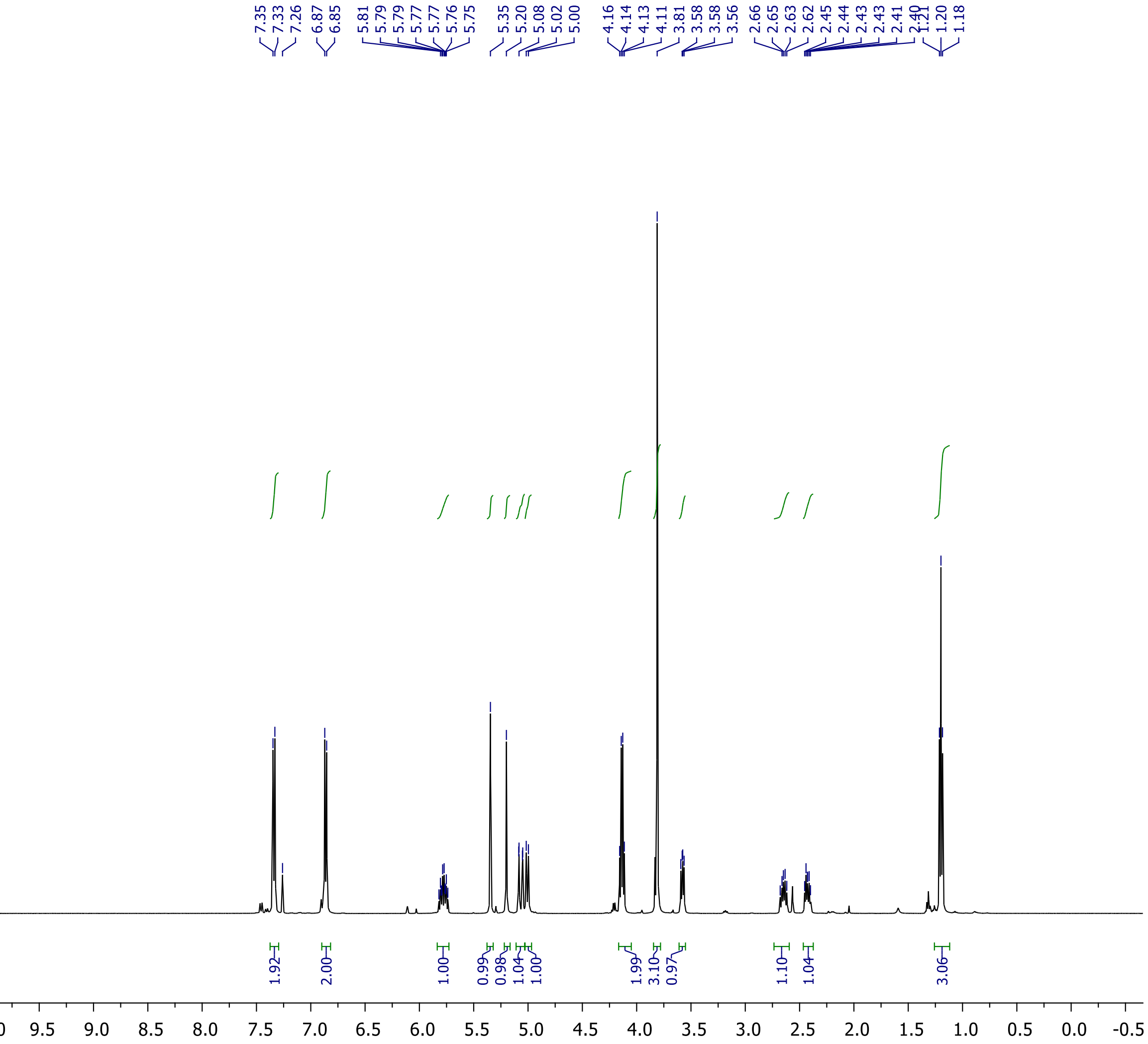

$^{13}C\{^1H\}$ NMR (151 MHz, $CDCl_3$)

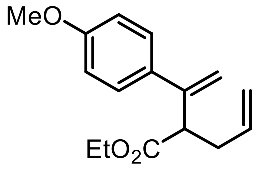

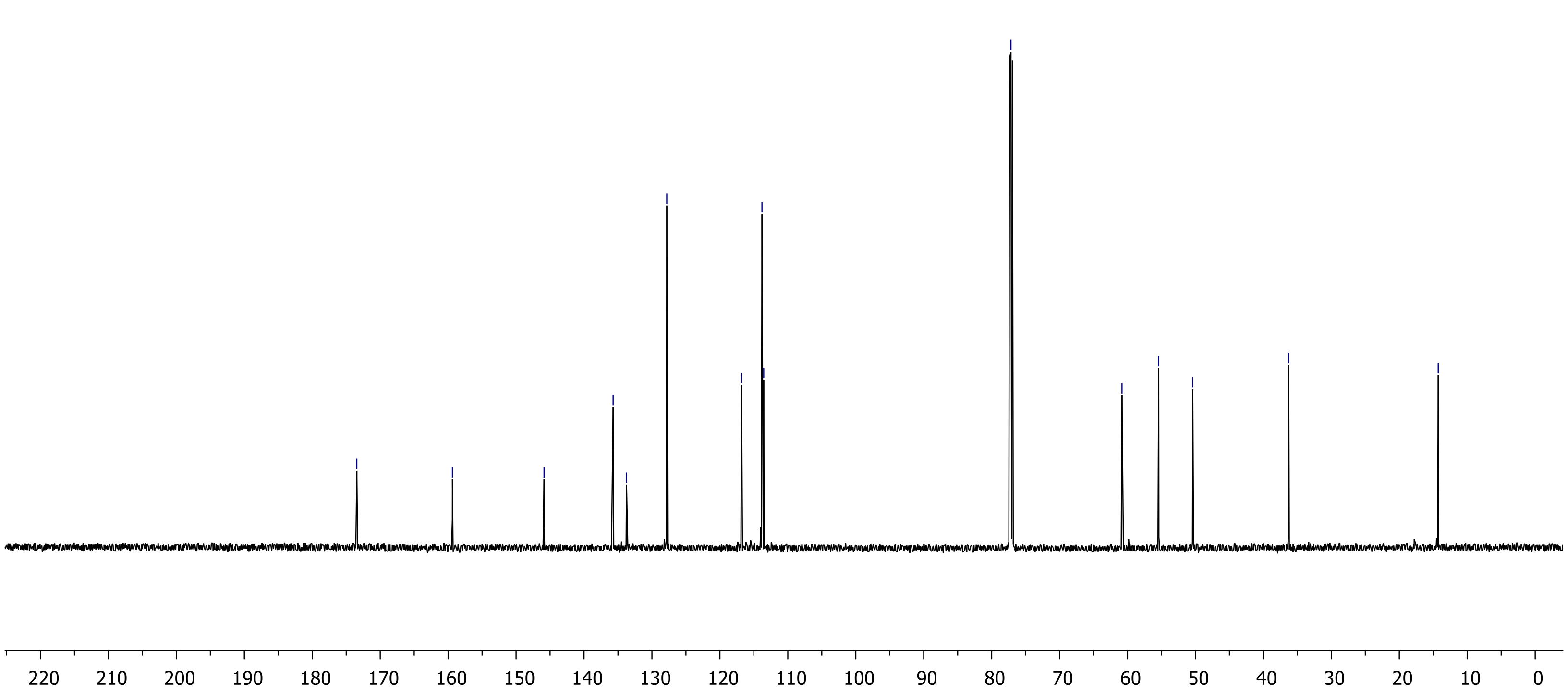

Compound (±)-9a (the sample contains ca. 10% of the isomeric 1,5-disubstituted bicyclo[2.1.1]hexane)

^{1}H NMR (500 MHz, $CDCl_3$)

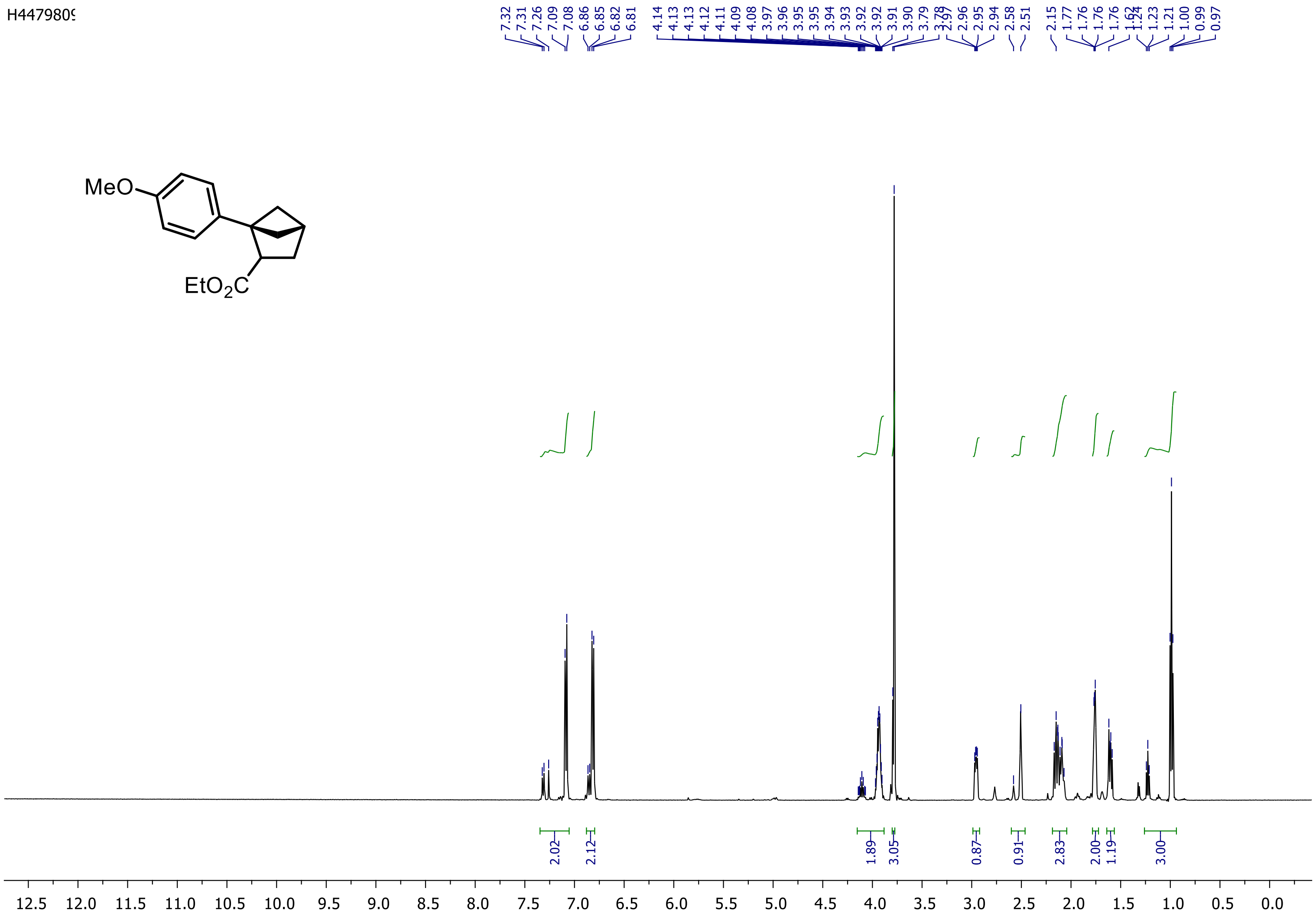

$^{13}C\{^1H\}$ NMR (126 MHz, $CDCl_3$)

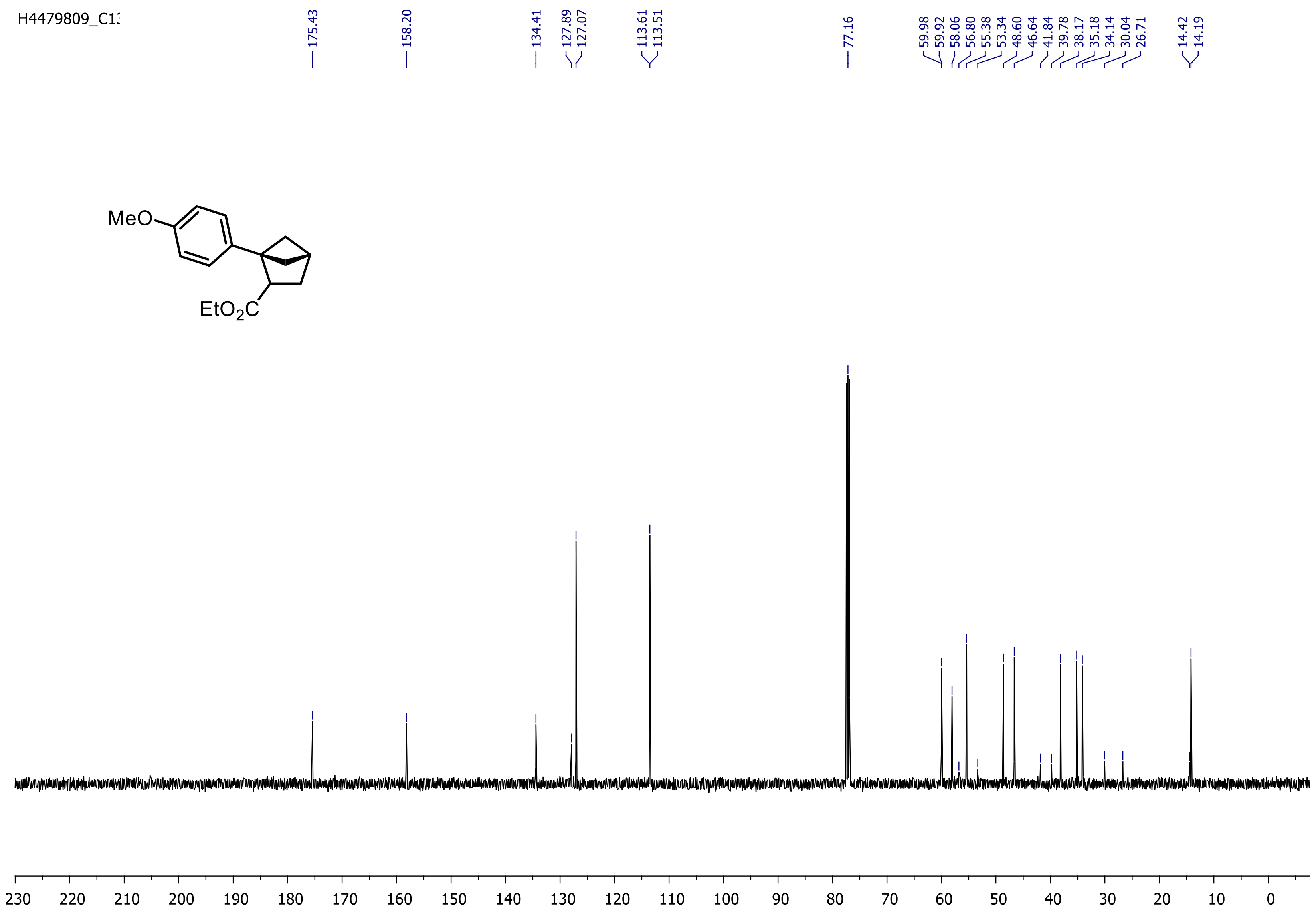

Compound (±)-9b

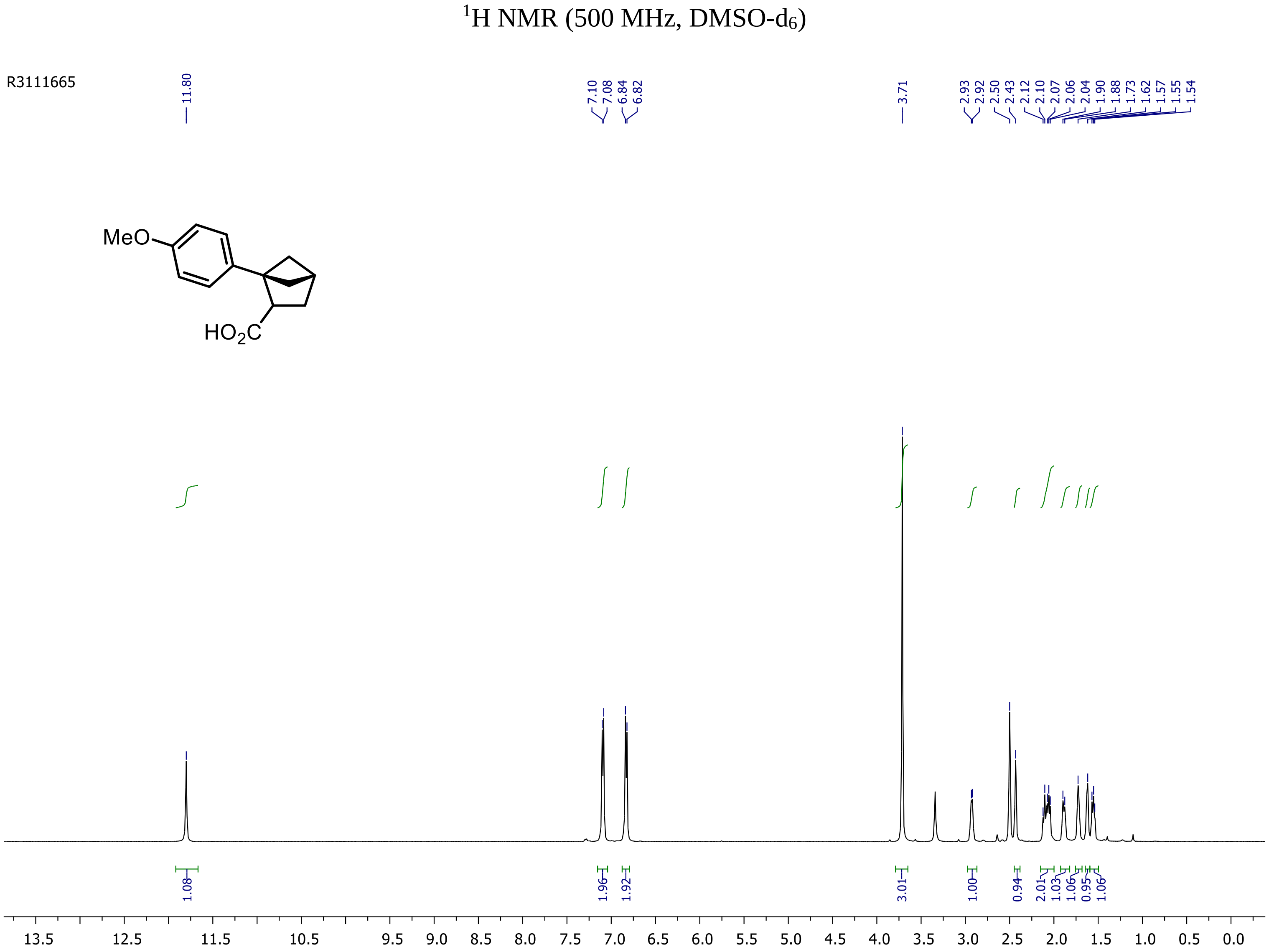
1H NMR (500 MHz, DMSO-d6)
R3111665
MeO
HO2C
11.80
7.10
7.08
6.84
6.82
3.71
2.93
2.92
2.50
2.43
2.12
2.10
2.07
2.06
2.04
1.90
1.88
1.73
1.62
1.57
1.55
1.54
1.08
1.96
1.92
3.01
1.00
0.94
2.01
1.03
1.06
0.95
1.06
13.5
12.5
11.5
10.5
9.5
9.0
8.5
8.0
7.5
7.0
6.5
6.0
5.5
5.0
4.5
4.0
3.5
3.0
2.5
2.0
1.5
1.0
0.5
0.0

$^{13}C\{^{1}H\}$ NMR (126 MHz, DMSO-d_6)

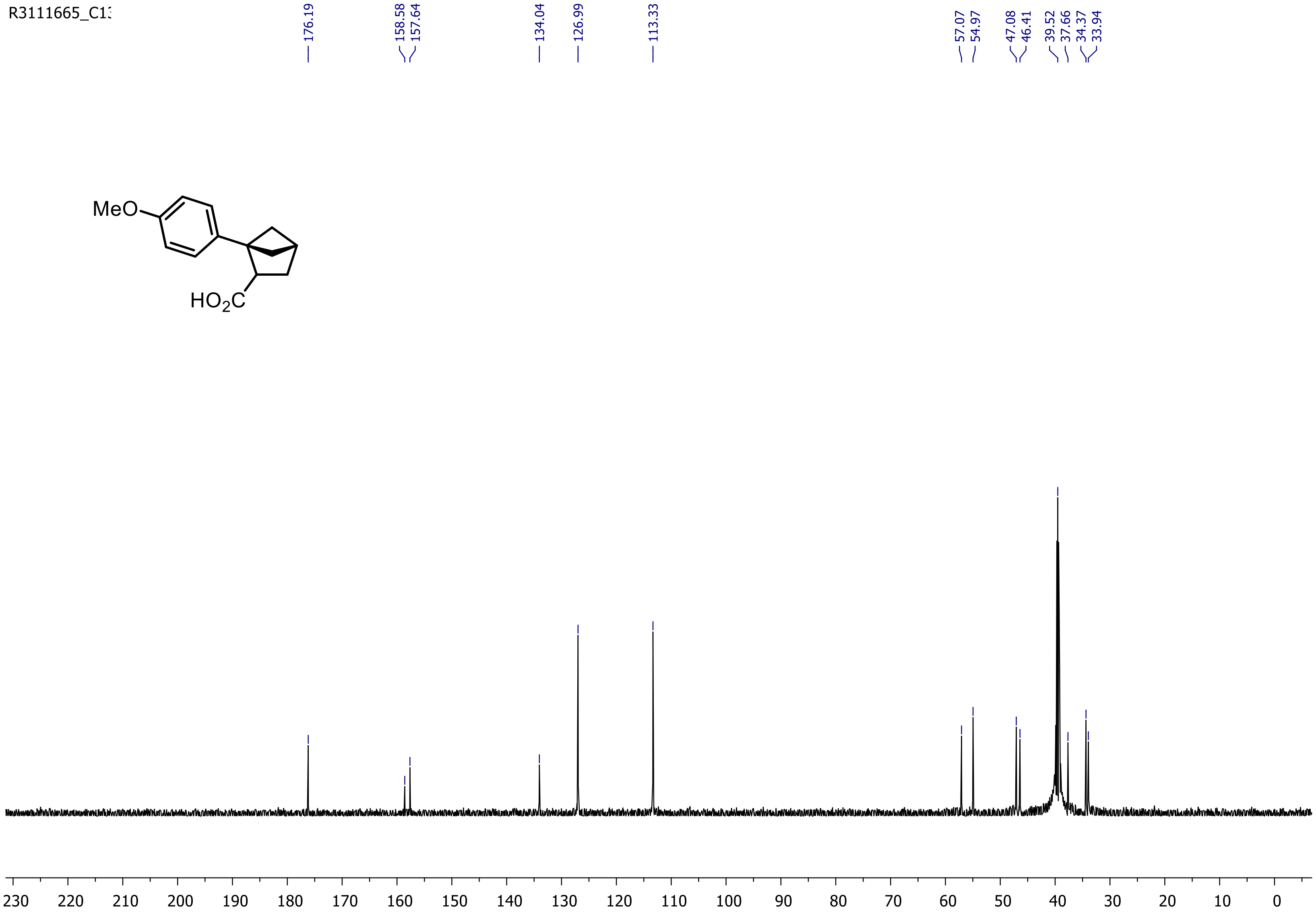

Ethyl-2-fluoro-3-(4-methoxyphenyl)but-2-enoate

^{1}H NMR (500 MHz, $CDCl_3$)

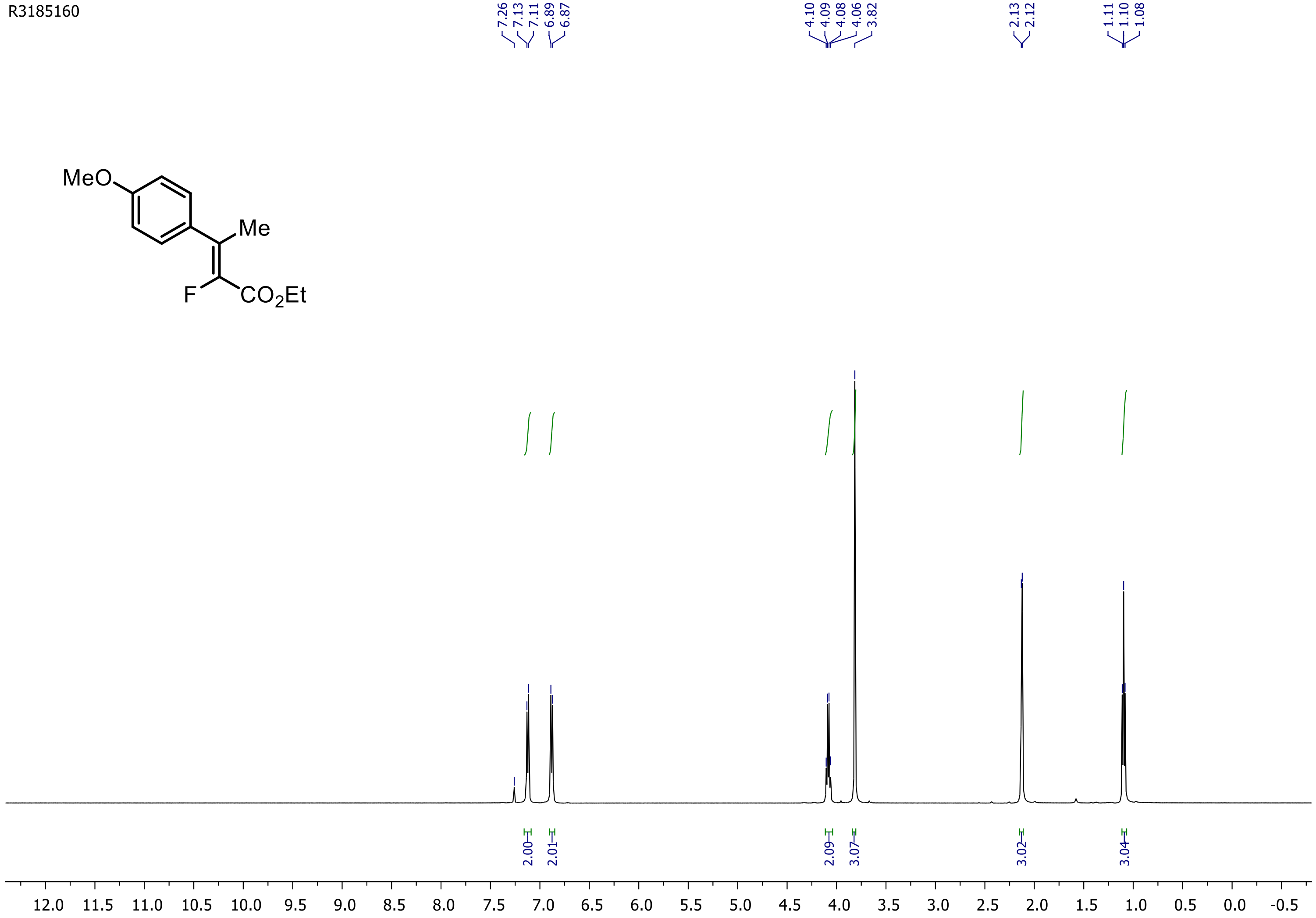

$^{13}C\{^1H\}$ NMR (126 MHz, $CDCl_3$)

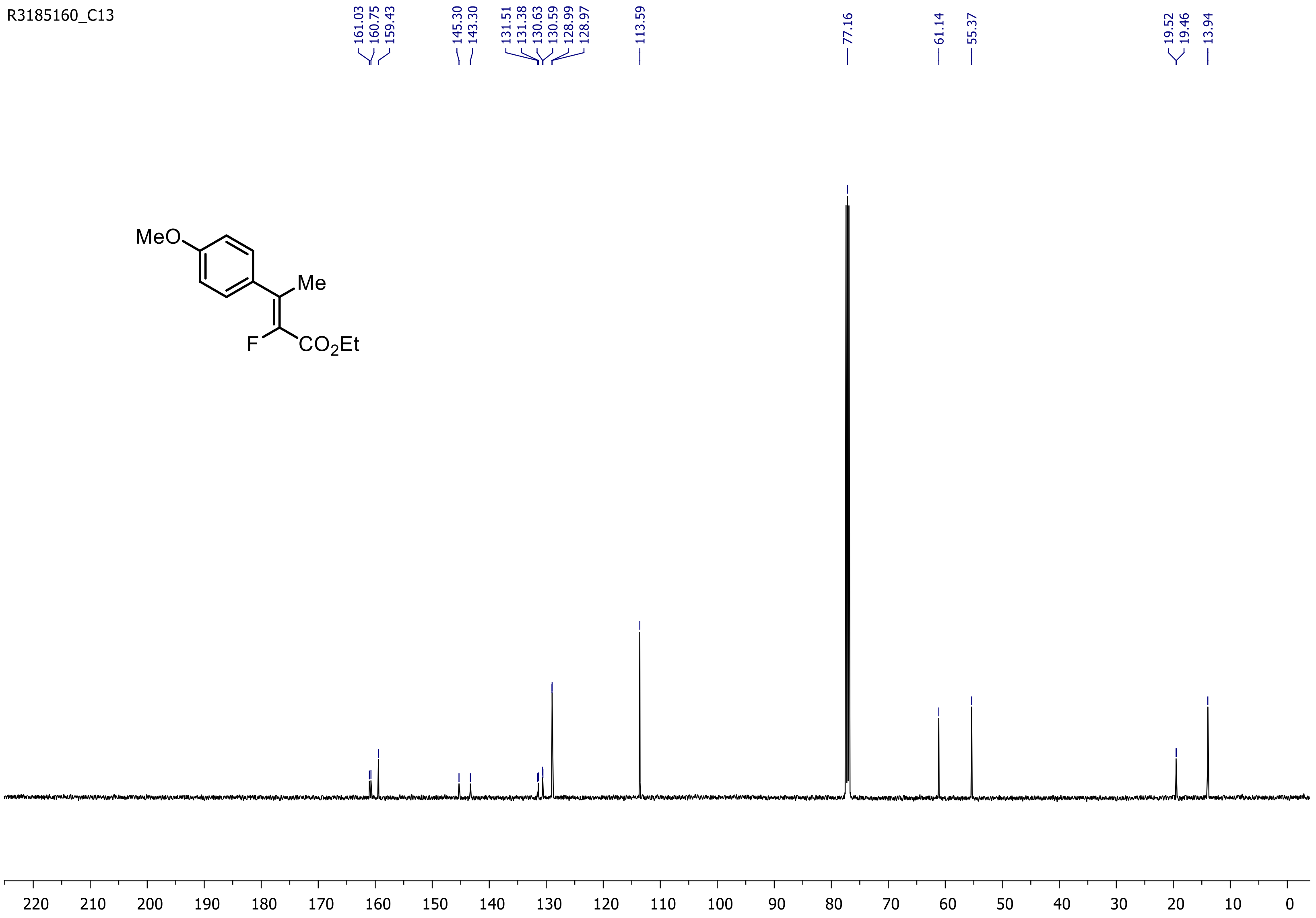

$^{19}F\{^1H\}$ NMR (376 MHz, $CDCl_3$)

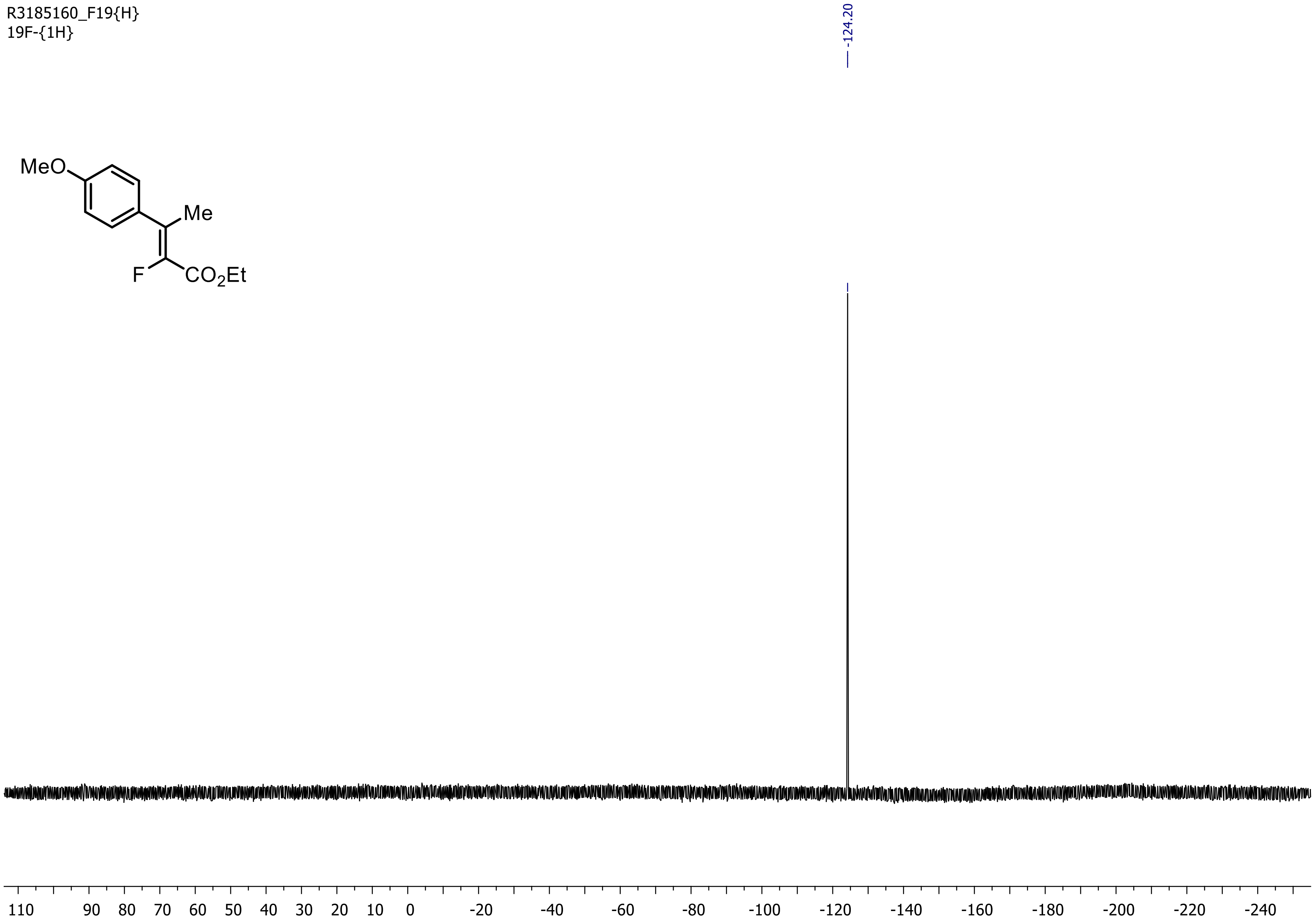

Compound 10

^{1}H NMR (500 MHz, $CDCl_3$)

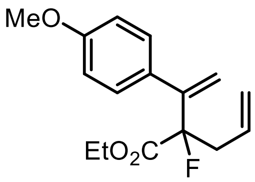

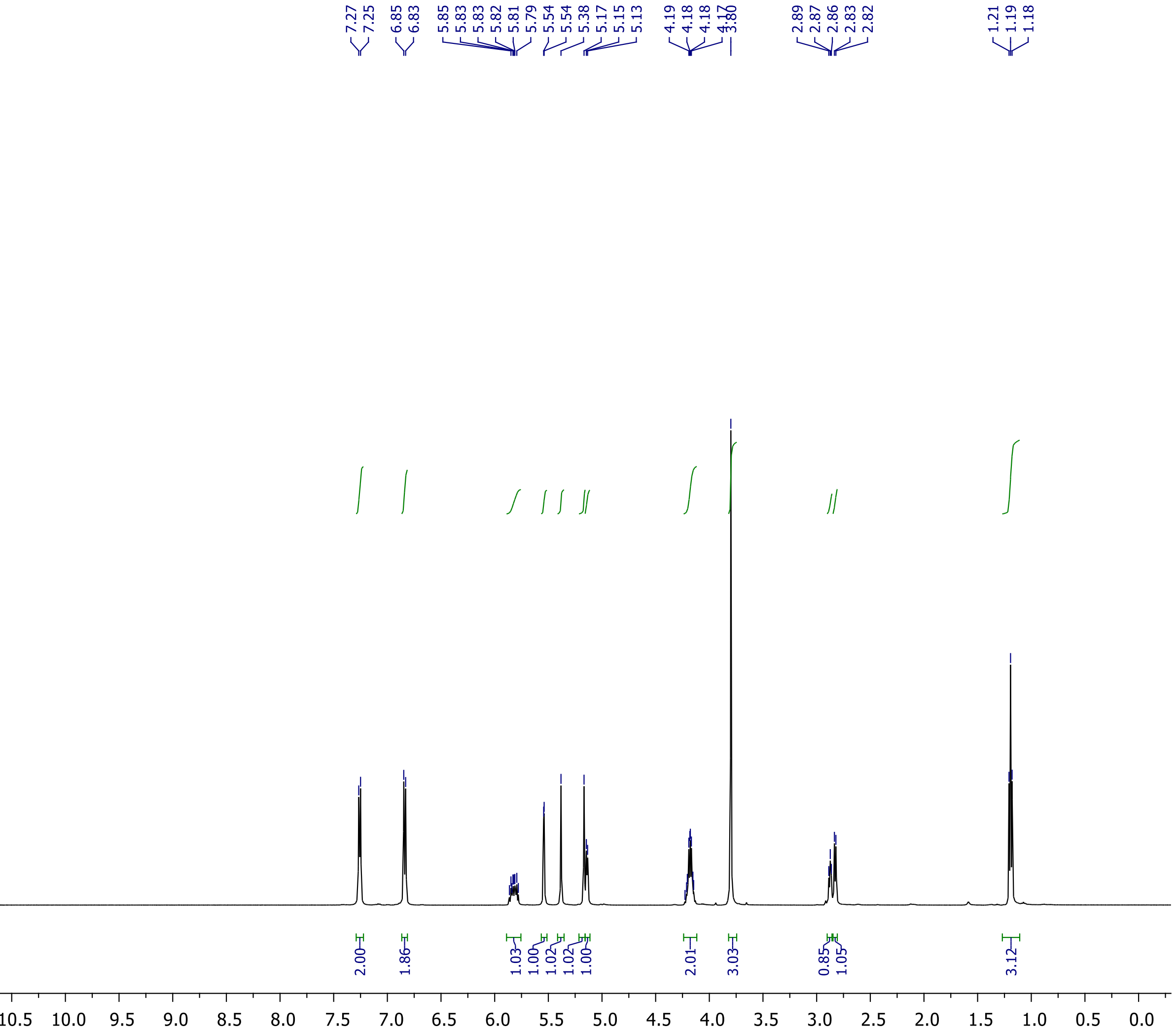

$^{13}C\{^{1}H\}$ NMR (126 MHz, $CDCl_3$)

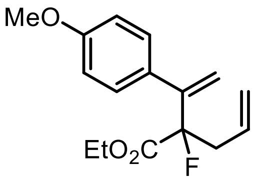

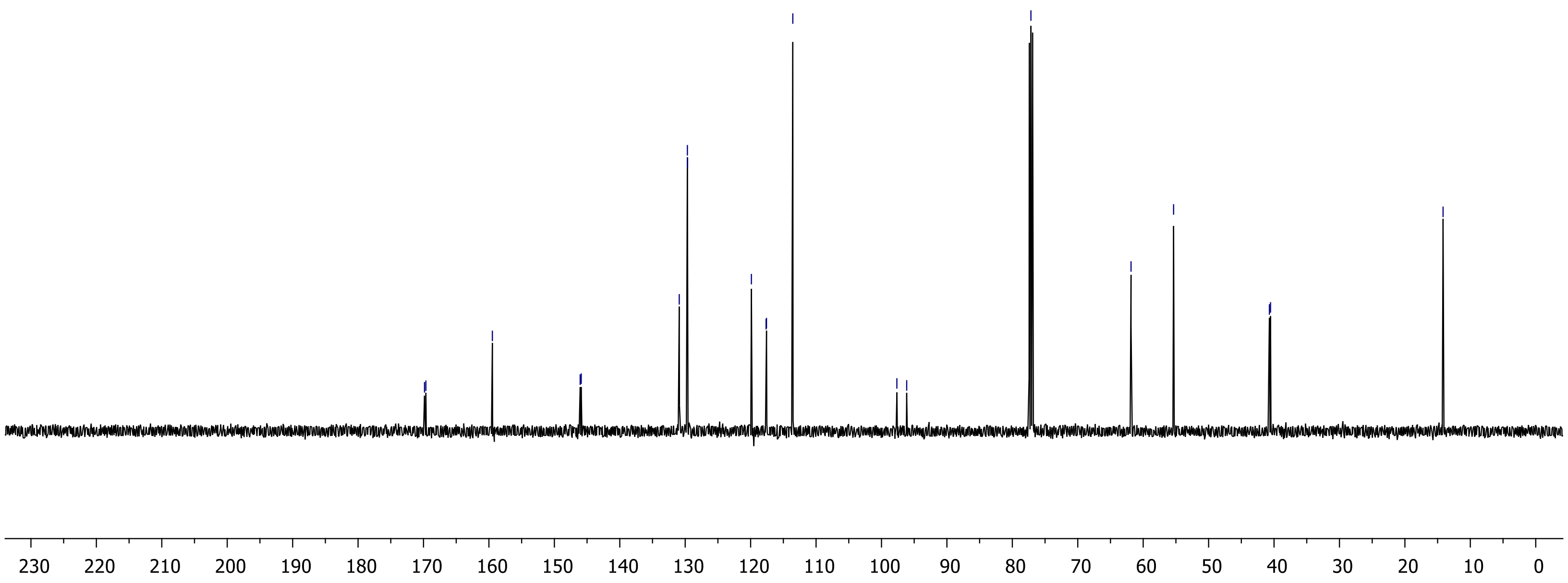

$^{19}F\{^{1}H\}$ NMR (376 MHz, $CDCl_3$)

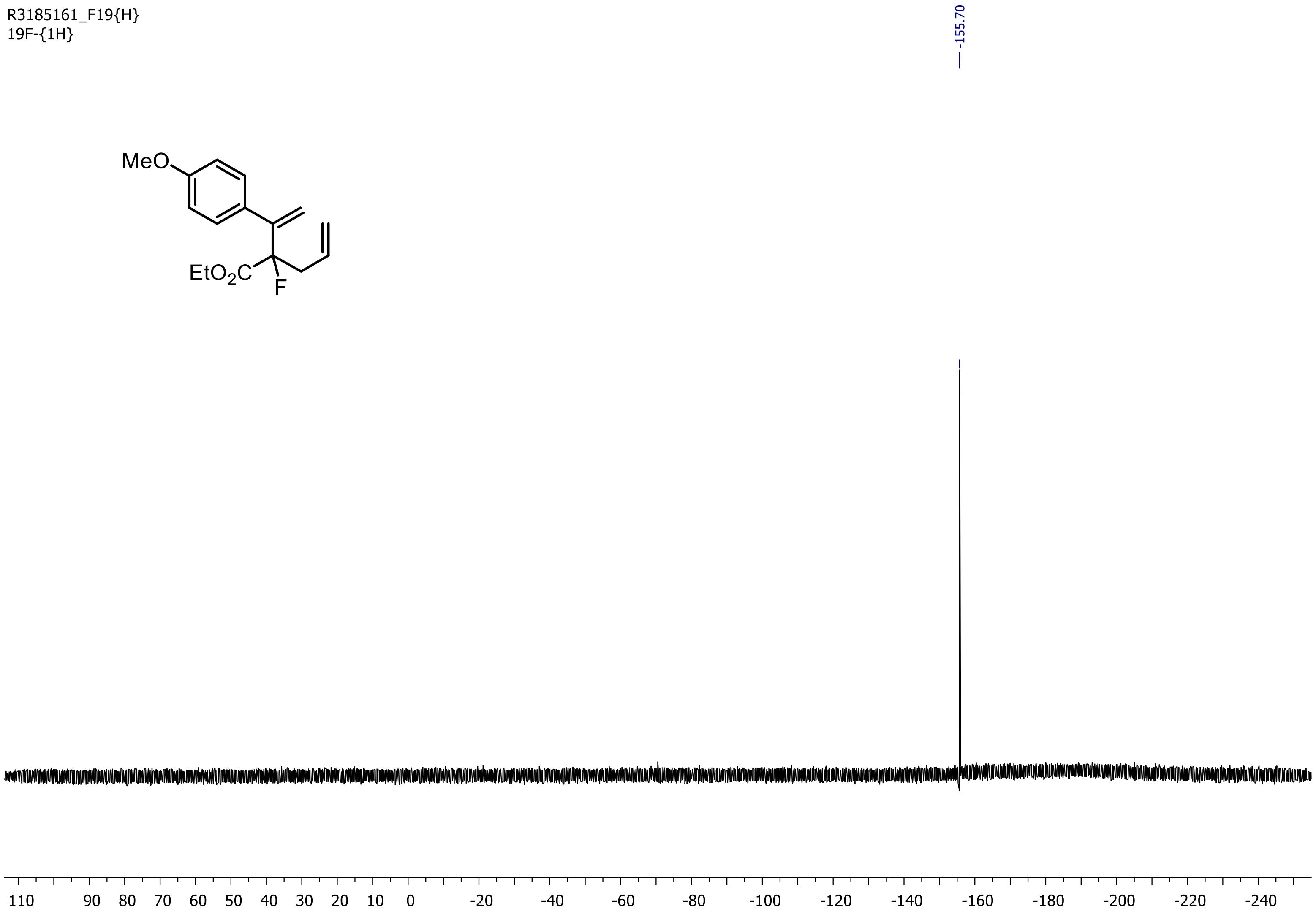

Compound (±)-10a

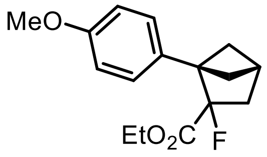

^{1}H NMR (500 MHz, $CDCl_3$)

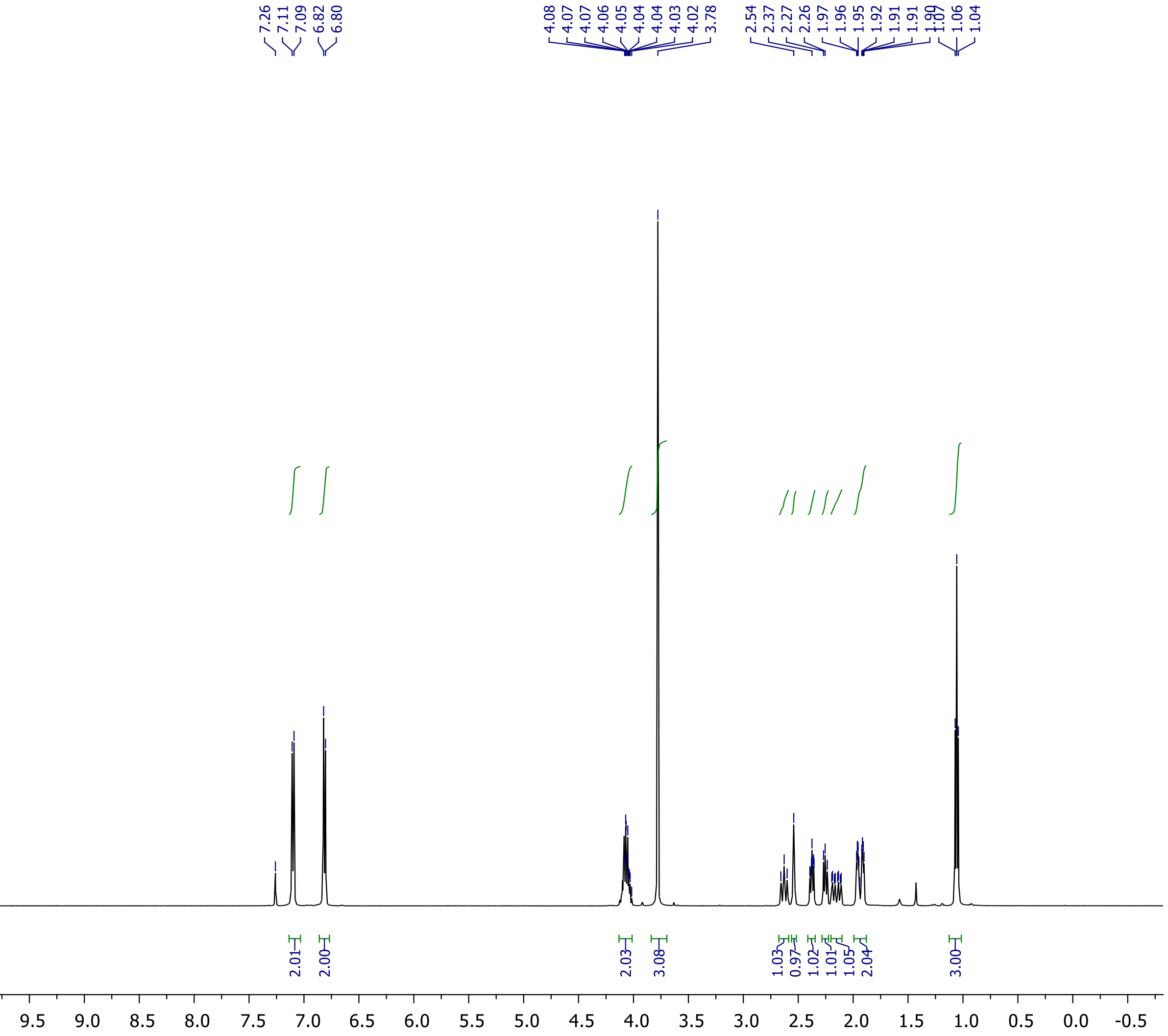

$^{13}C\{^{1}H\}$ NMR (126 MHz, $CDCl_3$)

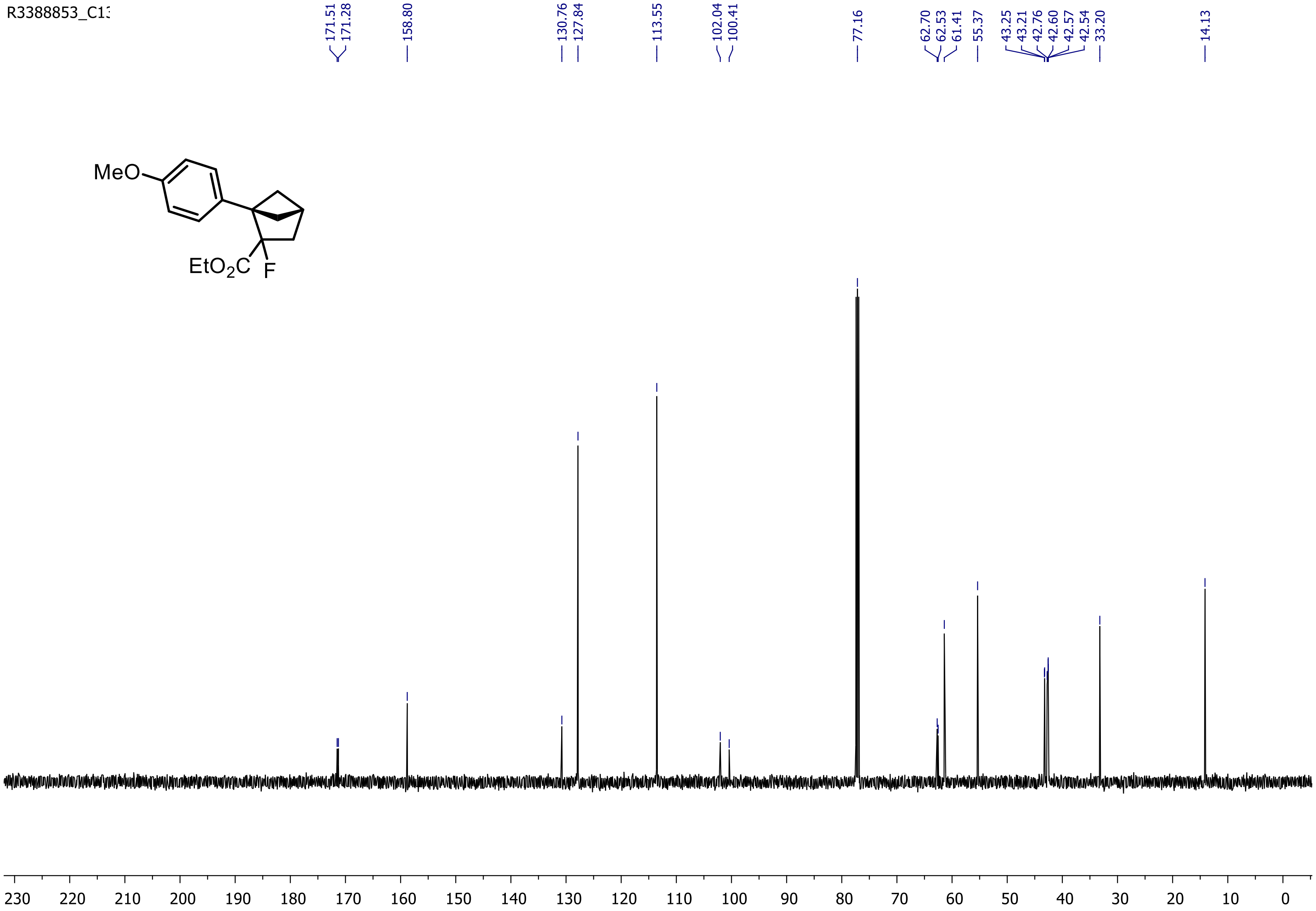

$^{19}F\{^{1}H\}$ NMR (376 MHz, $CDCl_3$)

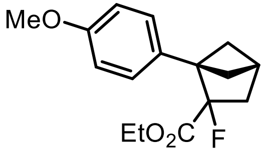

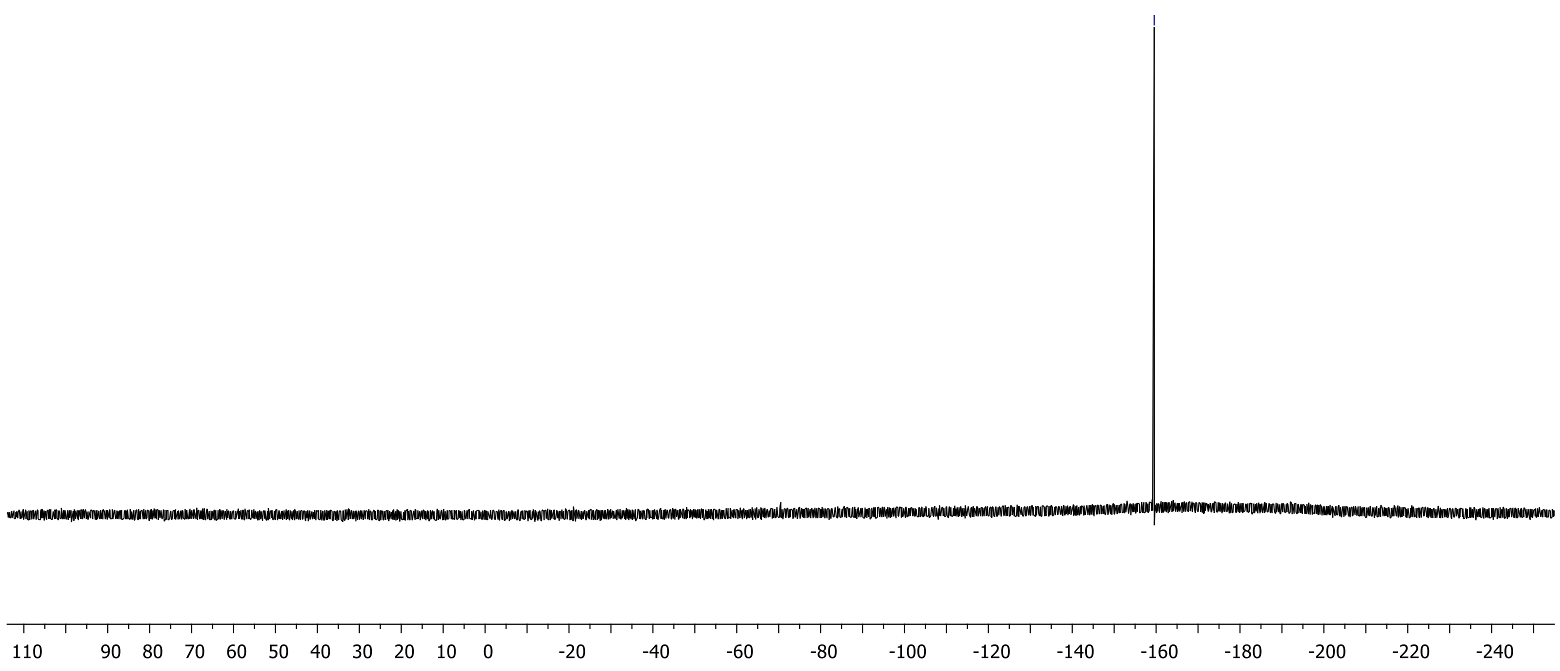

Compound (±)-10b

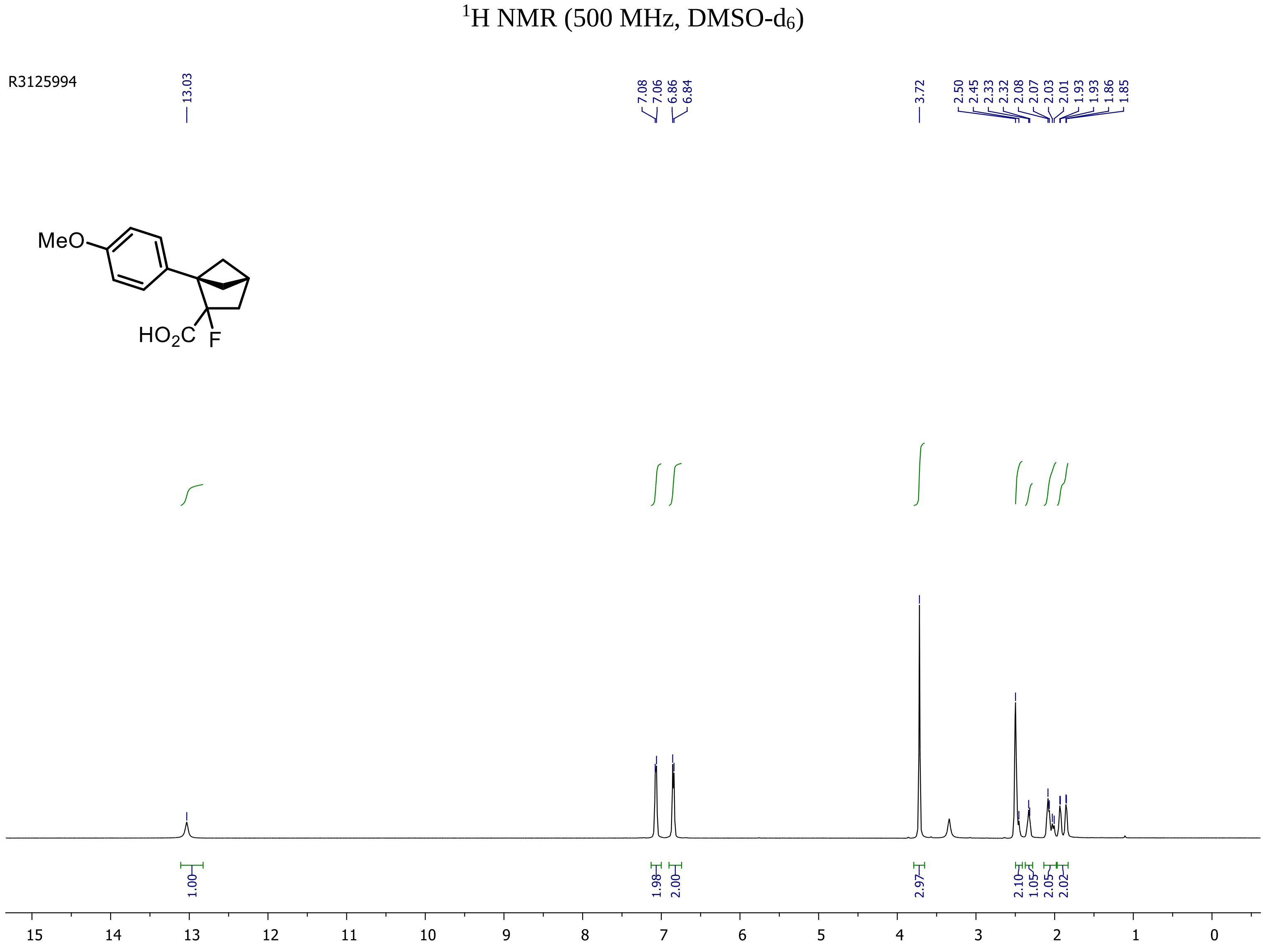

$^{13}C\{^{1}H\}$ NMR (126 MHz, $CDCl_3$)

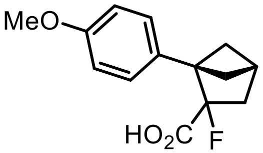

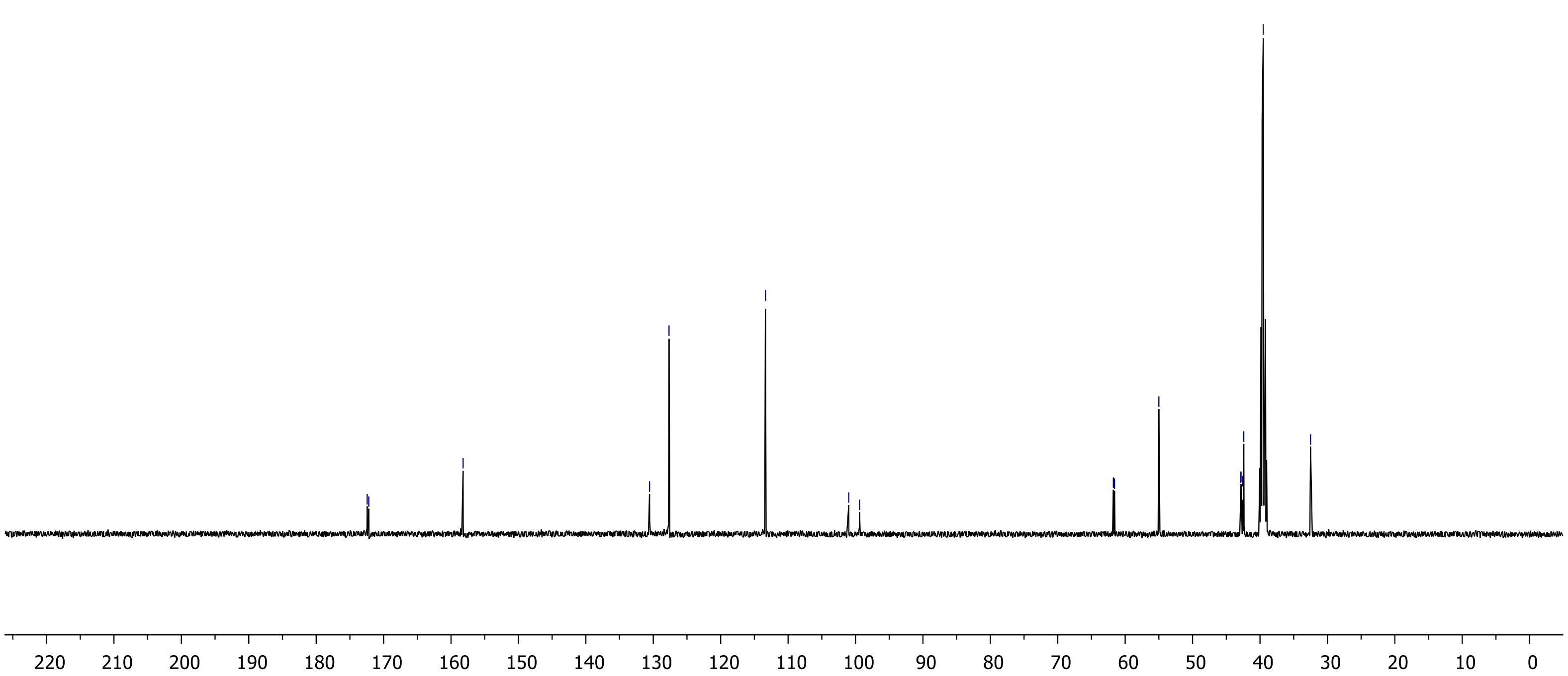

$^{19}F\{^{1}H\}$ NMR (376 MHz, DMSO-d_6)

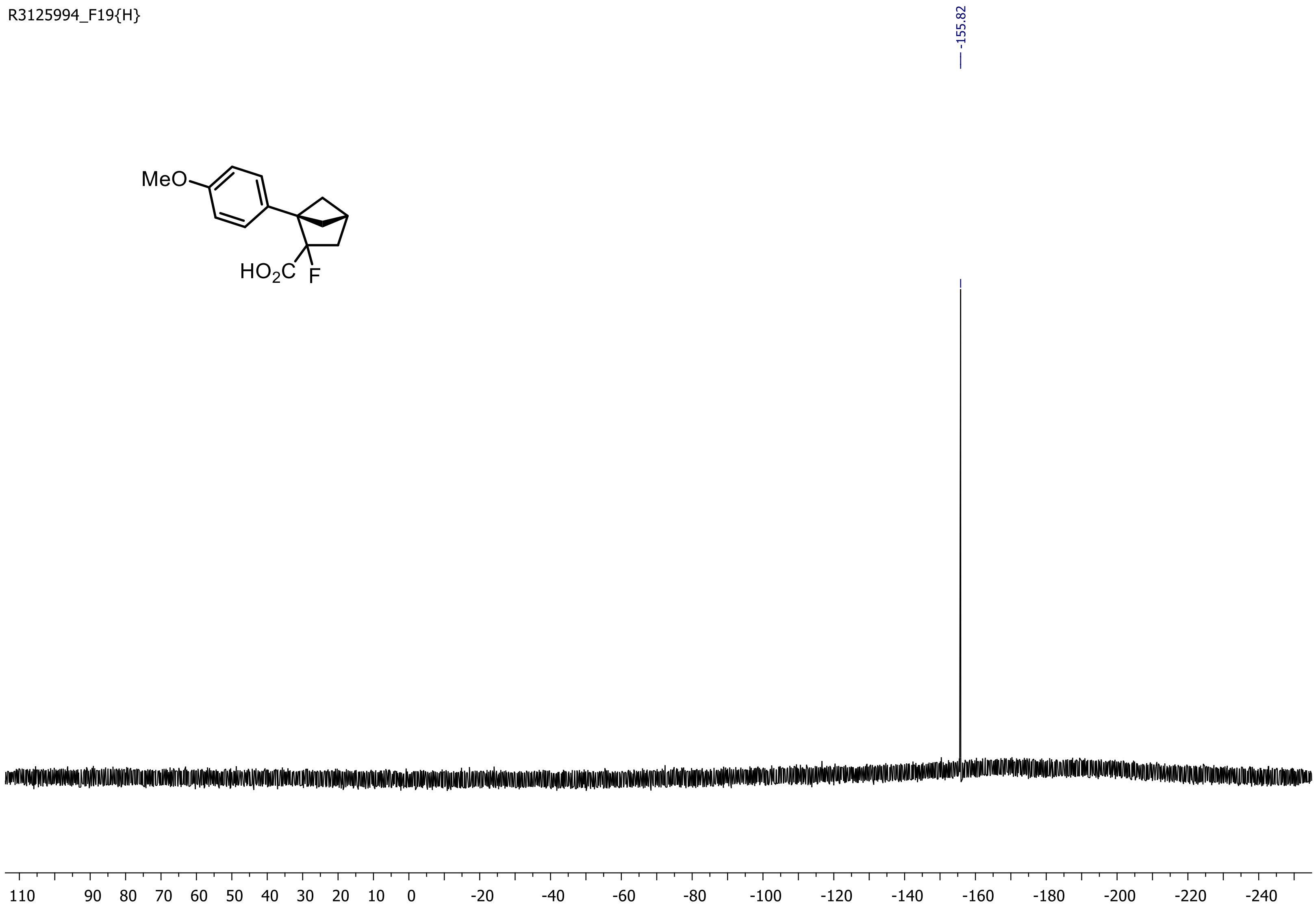

Ethyl-3-(3-bromophenyl)but-2-enoate

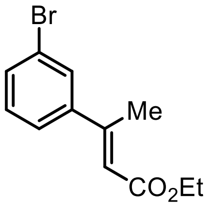

^{1}H NMR (500 MHz, $CDCl_3$)

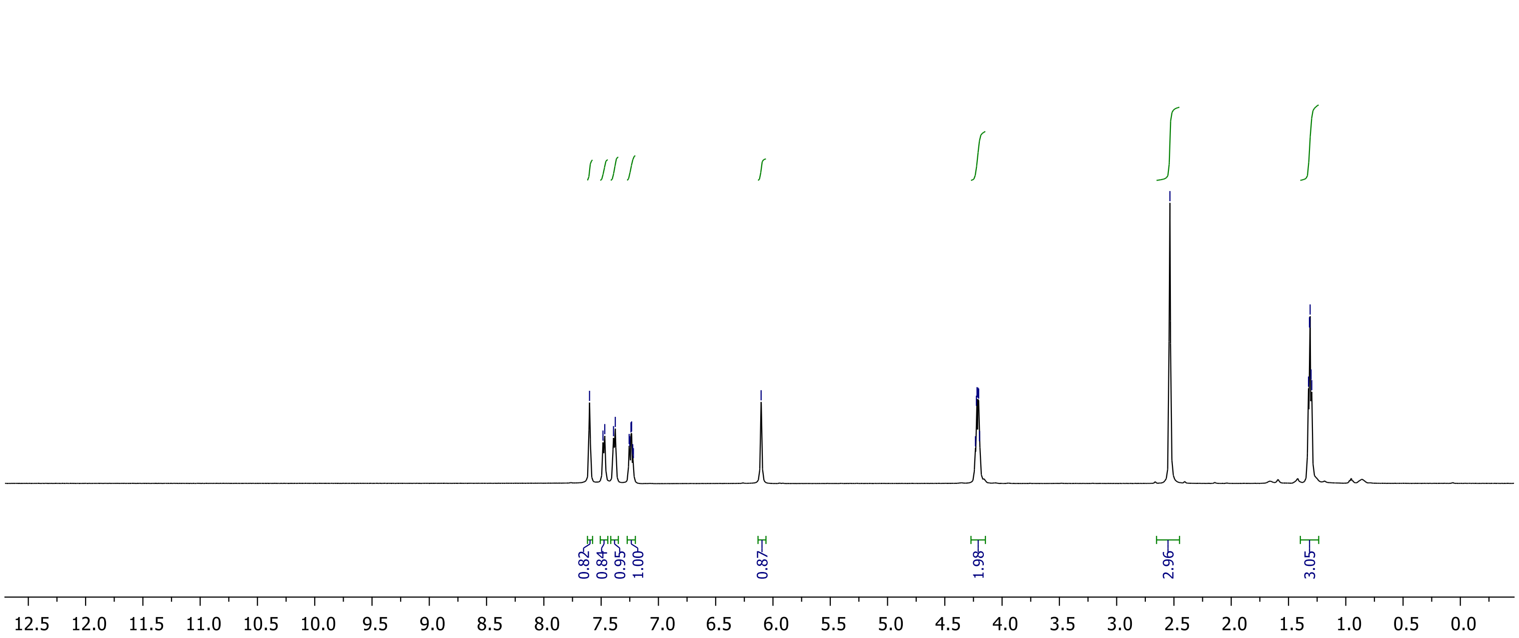

$^{13}C\{^1H\}$ NMR (126 MHz, $CDCl_3$)

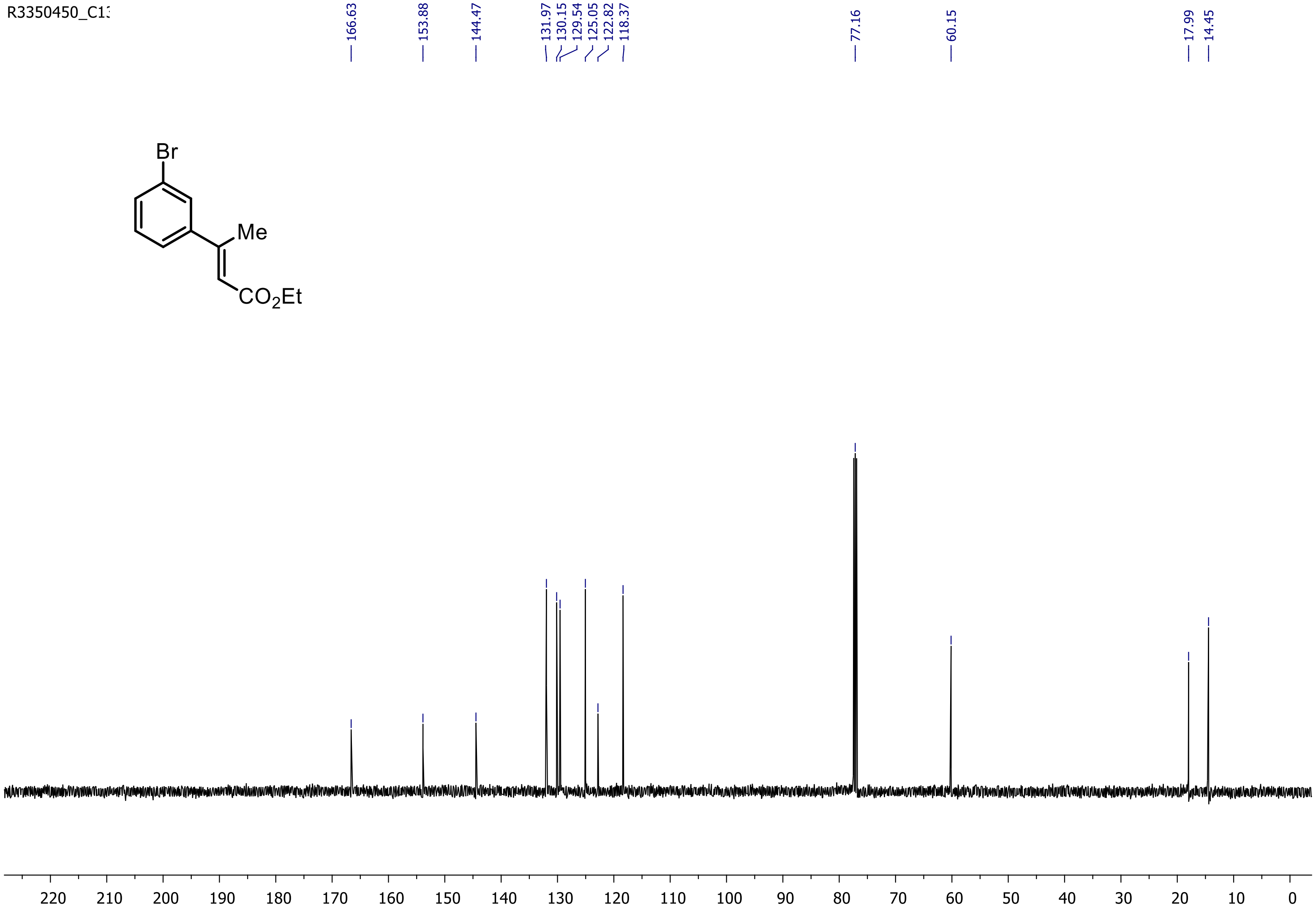

Compound 11

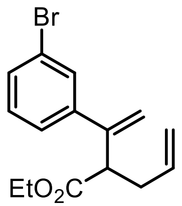

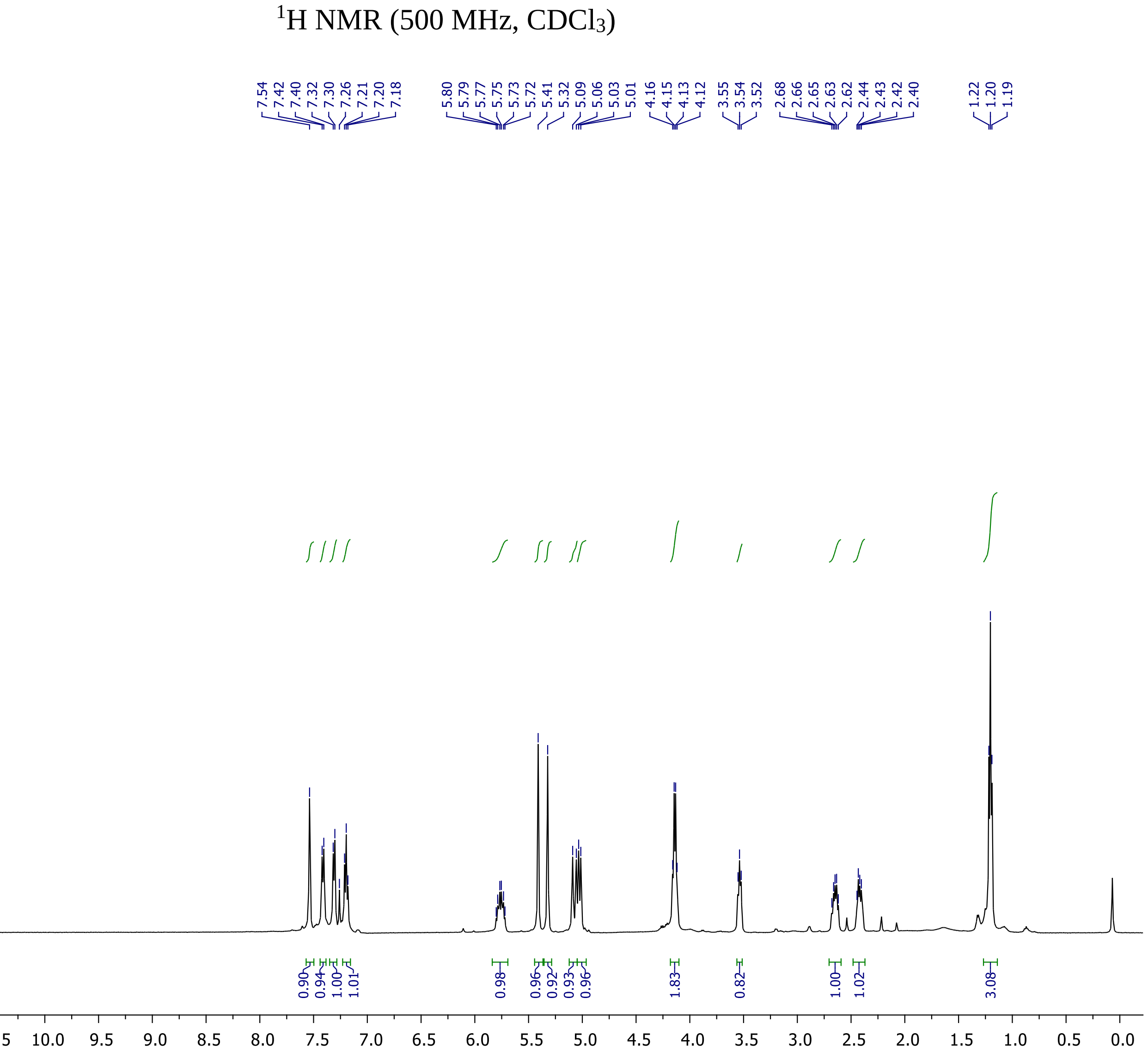

^{1}H NMR (500 MHz, CDCl$_3$)
R3331647
7.54 7.42 7.40 7.32 7.30 7.26 7.21 7.20 7.18 5.80 5.79 5.77 5.75 5.73 5.72 5.41 5.32 5.09 5.06 5.03 5.01 4.16 4.15 4.13 4.12 3.55 3.54 3.52 2.68 2.66 2.65 2.63 2.62 2.44 2.43 2.42 2.40 1.22 1.20 1.19
0.90 0.94 1.00 1.01 0.98 0.96 0.92 0.93 0.96 1.83 0.82 1.00 1.02 3.08
12.5 12.0 11.5 11.0 10.5 10.0 9.5 9.0 8.5 8.0 7.5 7.0 6.5 6.0 5.5 5.0 4.5 4.0 3.5 3.0 2.5 2.0 1.5 1.0 0.5 0.0

$^{13}C\{^{1}H\}$ NMR (126 MHz, $CDCl_3$)

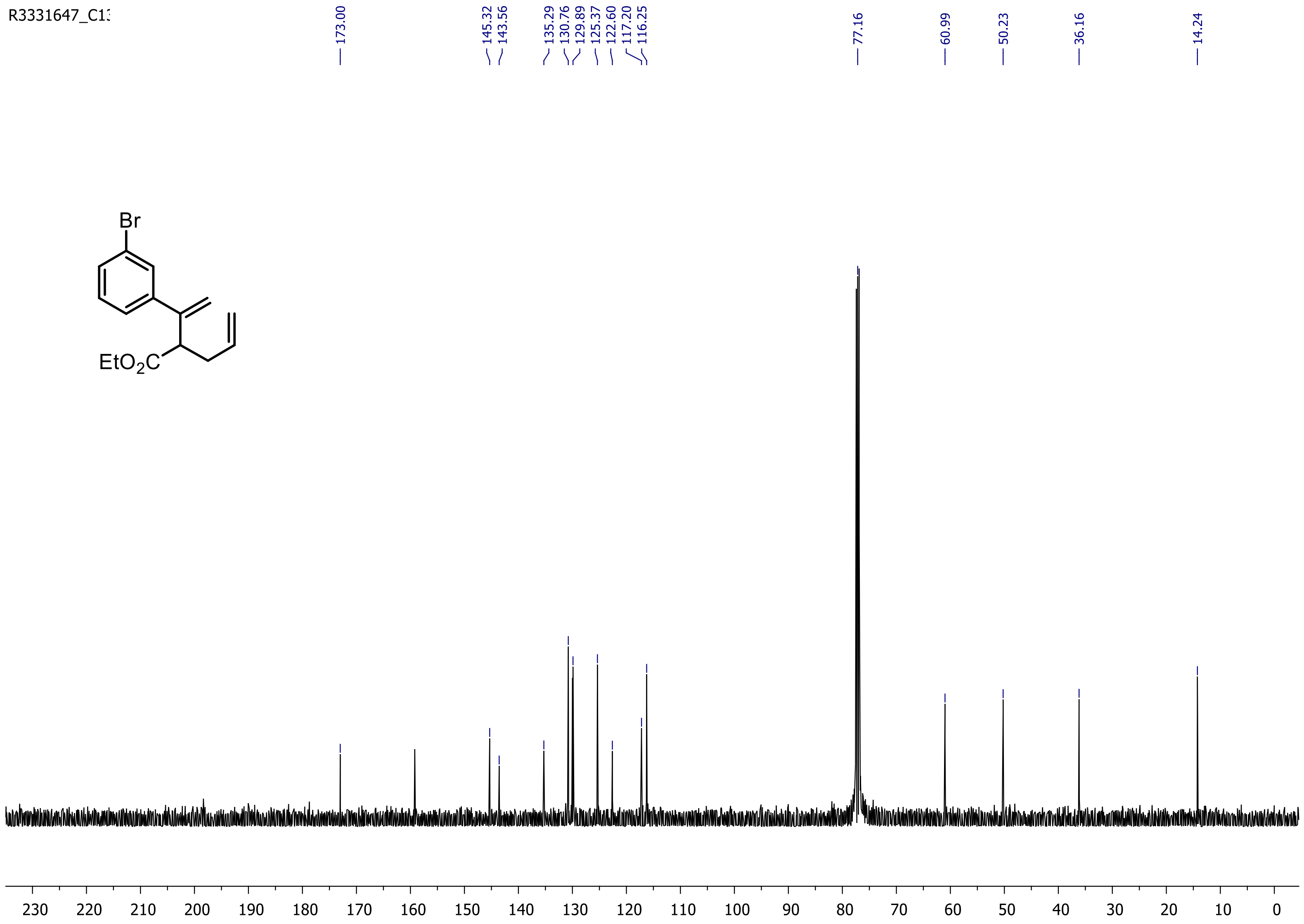

Compound (±)-11a

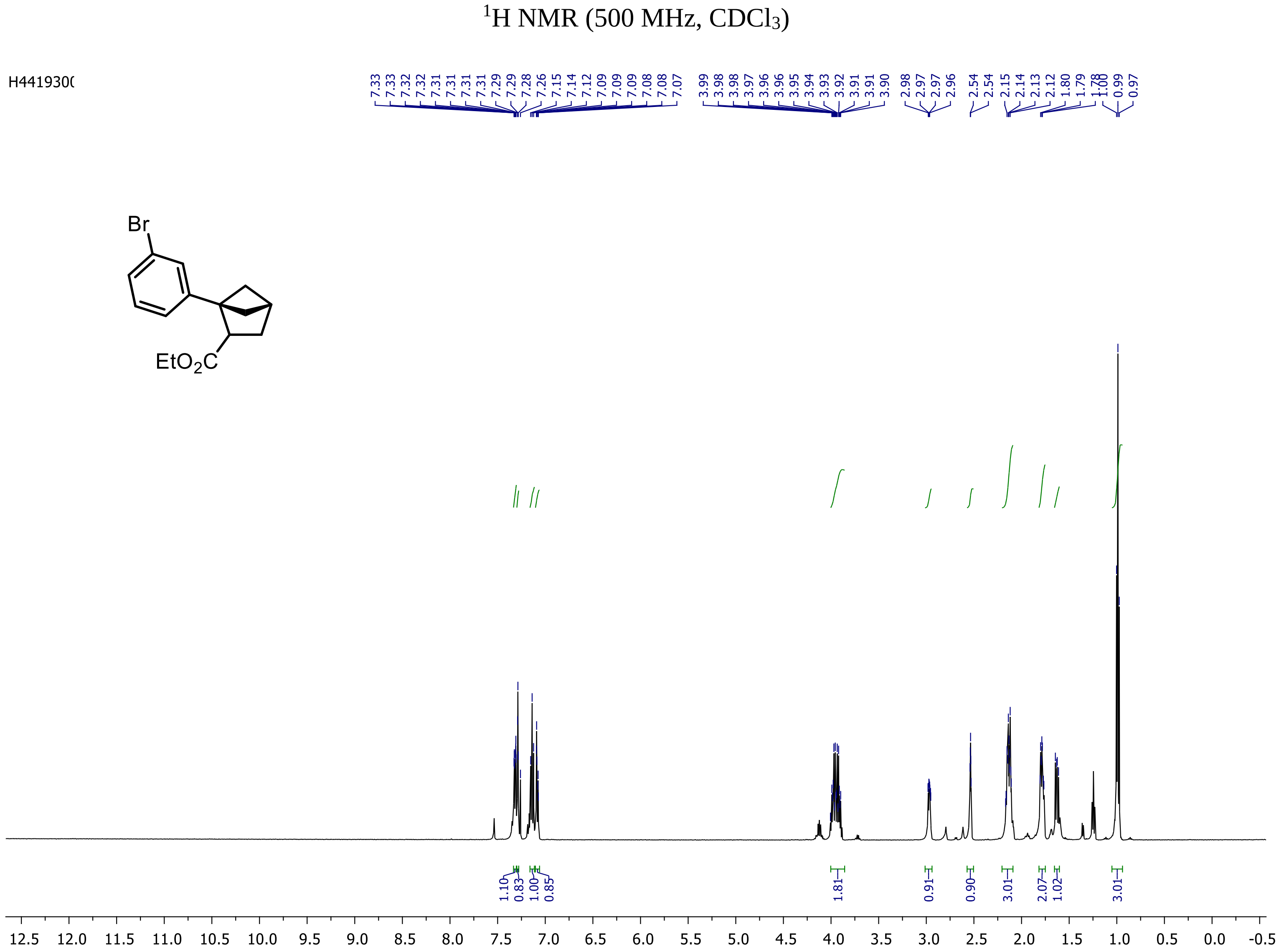

$^{13}C\{^1H\}$ NMR (126 MHz, $CDCl_3$)

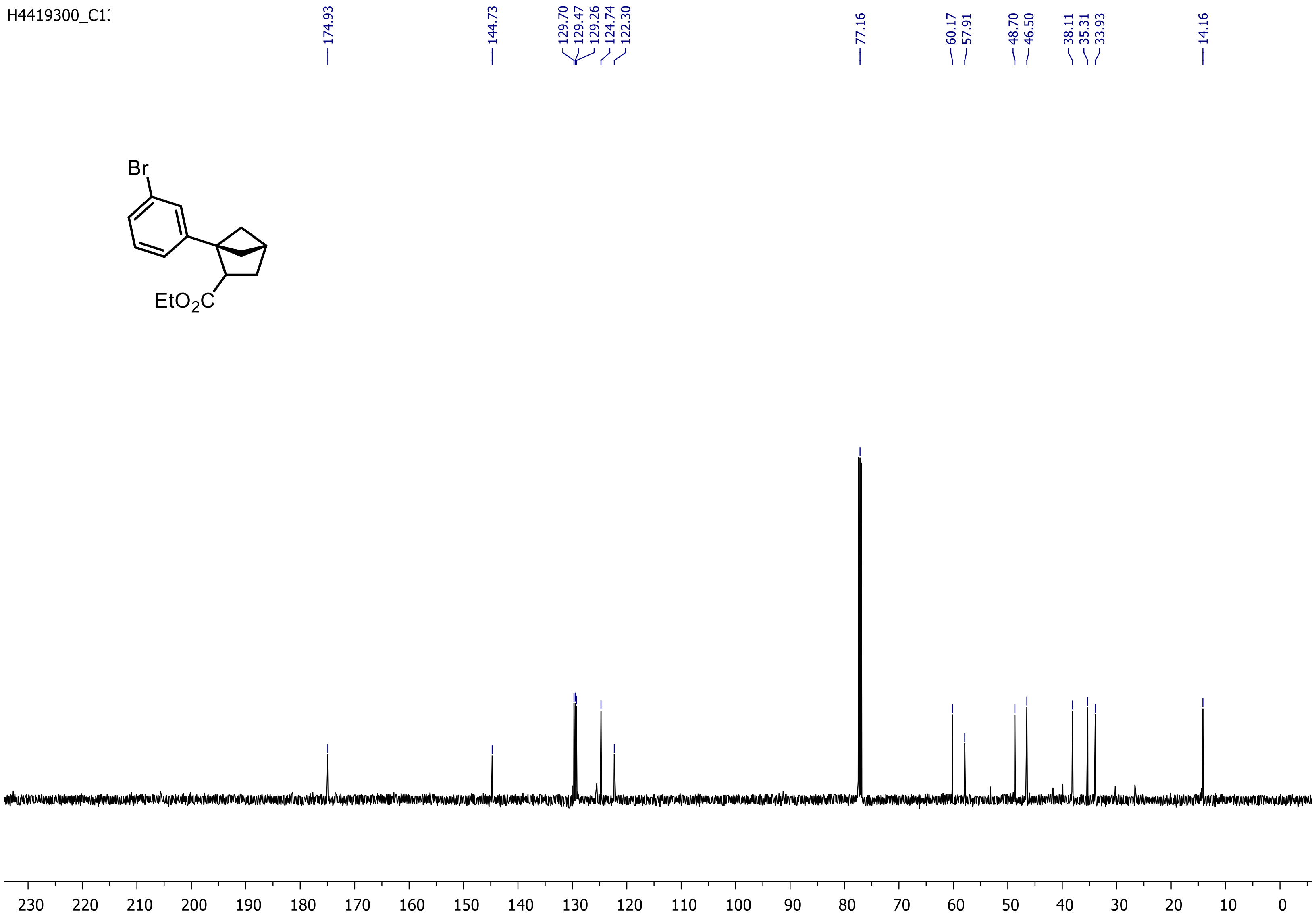

Compound (±)-11b

^{1}H NMR (500 MHz, DMSO-d_6)

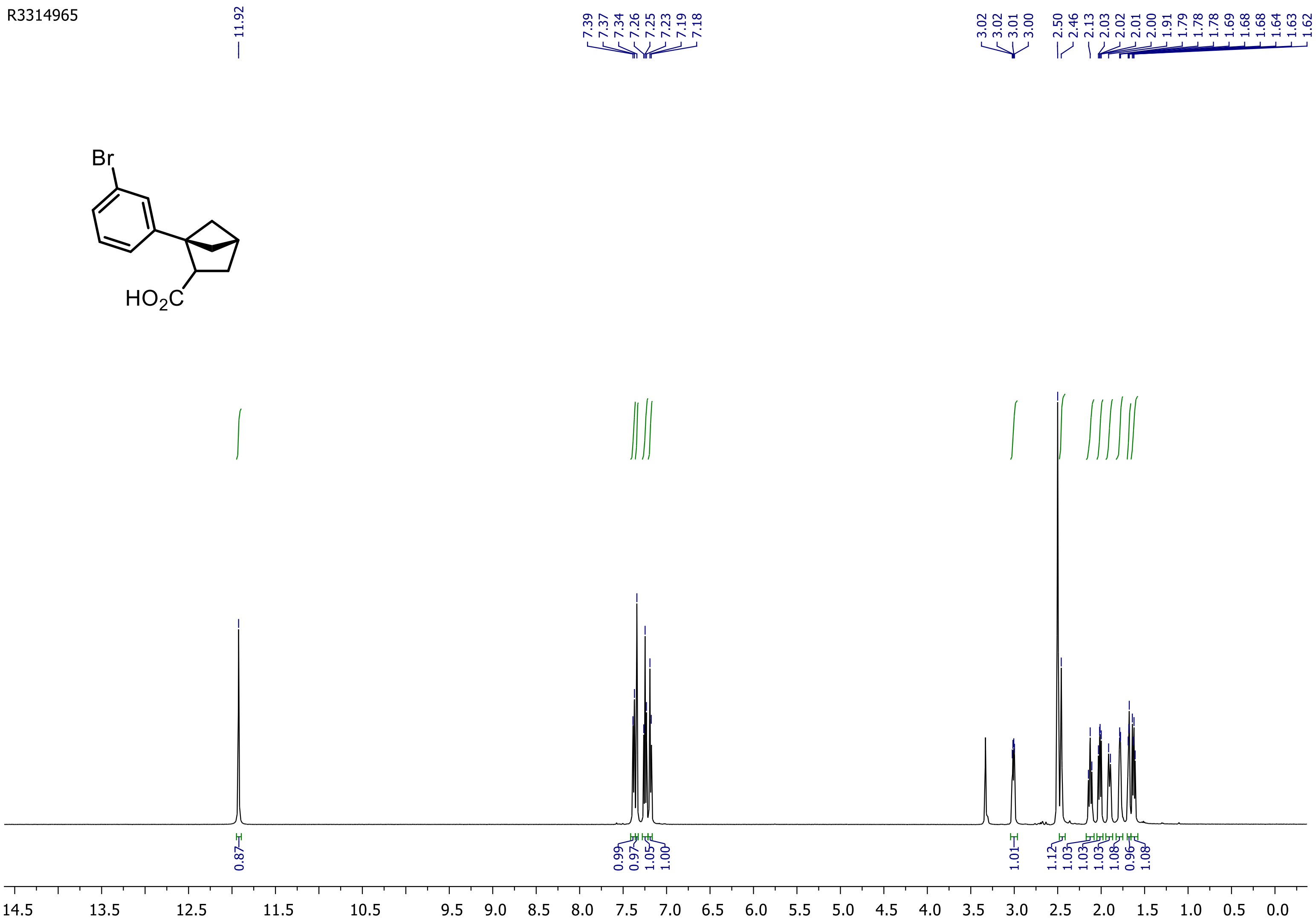

$^{13}C\{^1H\}$ NMR (101 MHz, DMSO-d$_6$)

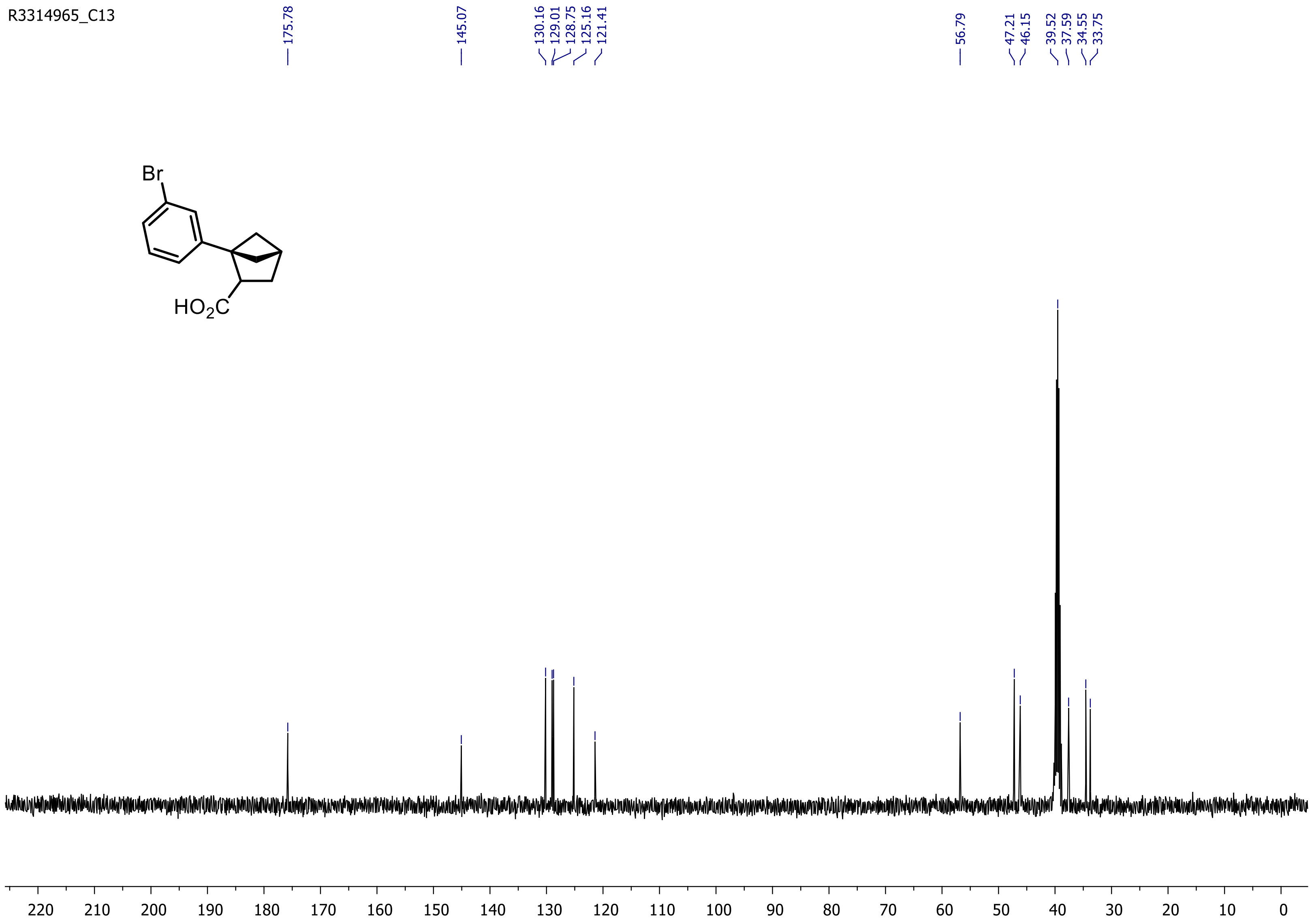

Ethyl-3-(3-(trifluoromethyl)phenyl)but-2-enoate

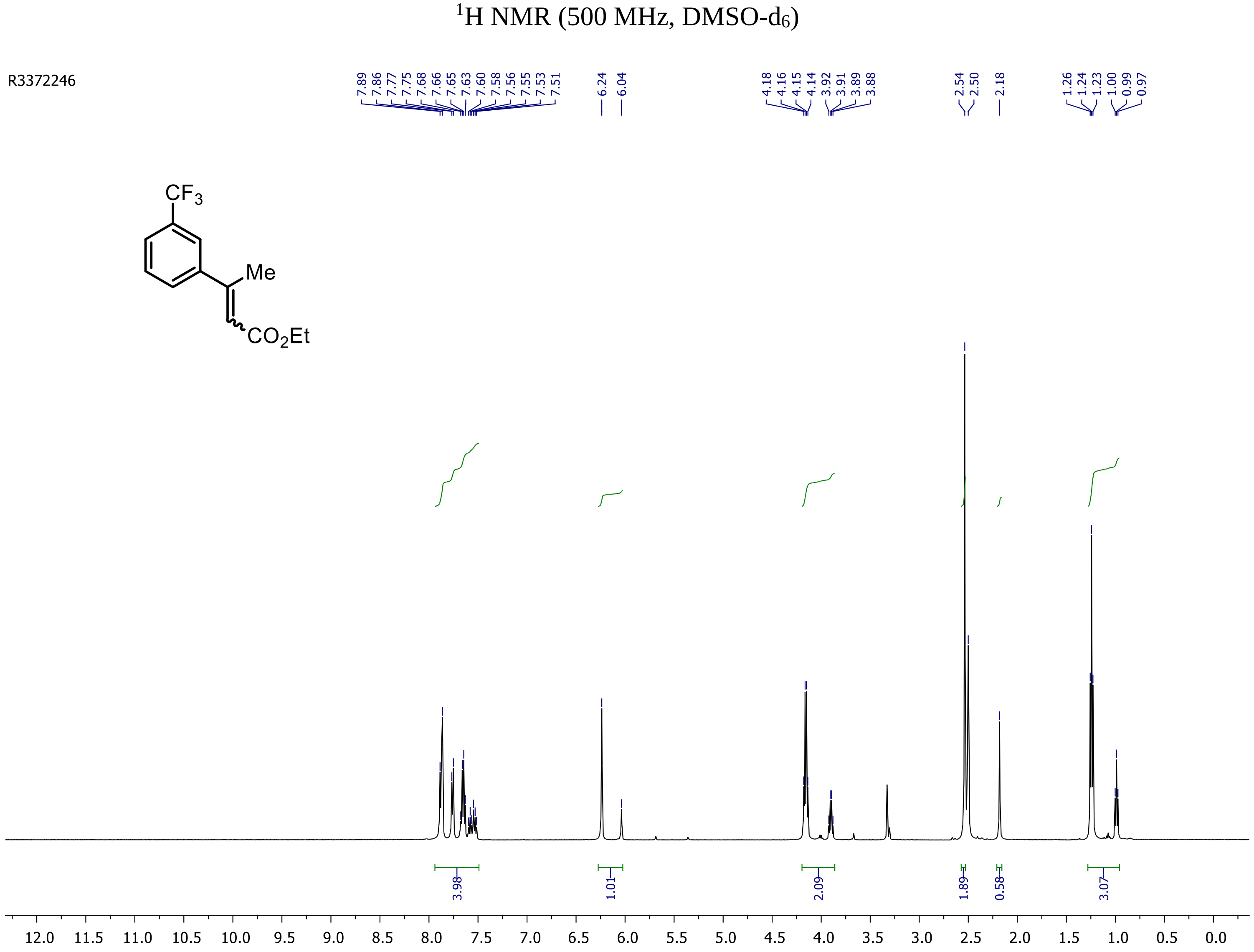

$^{13}C\{^{1}H\}$ NMR (151 MHz, DMSO-d$_6$)

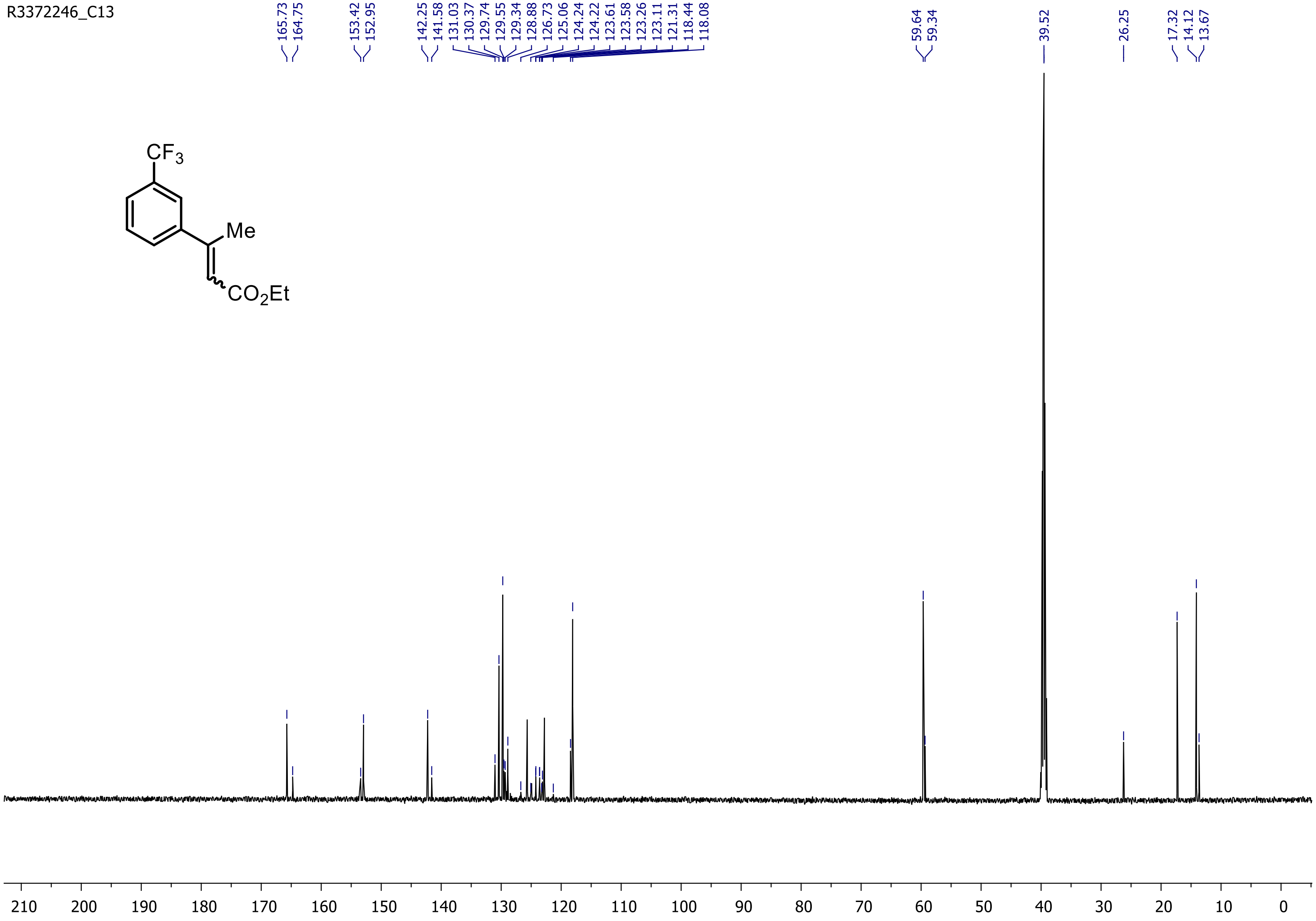

$^{19}F\{^{1}H\}$ NMR (376 MHz, DMSO-d_6)

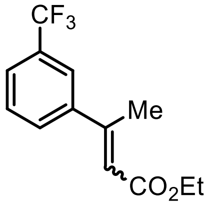

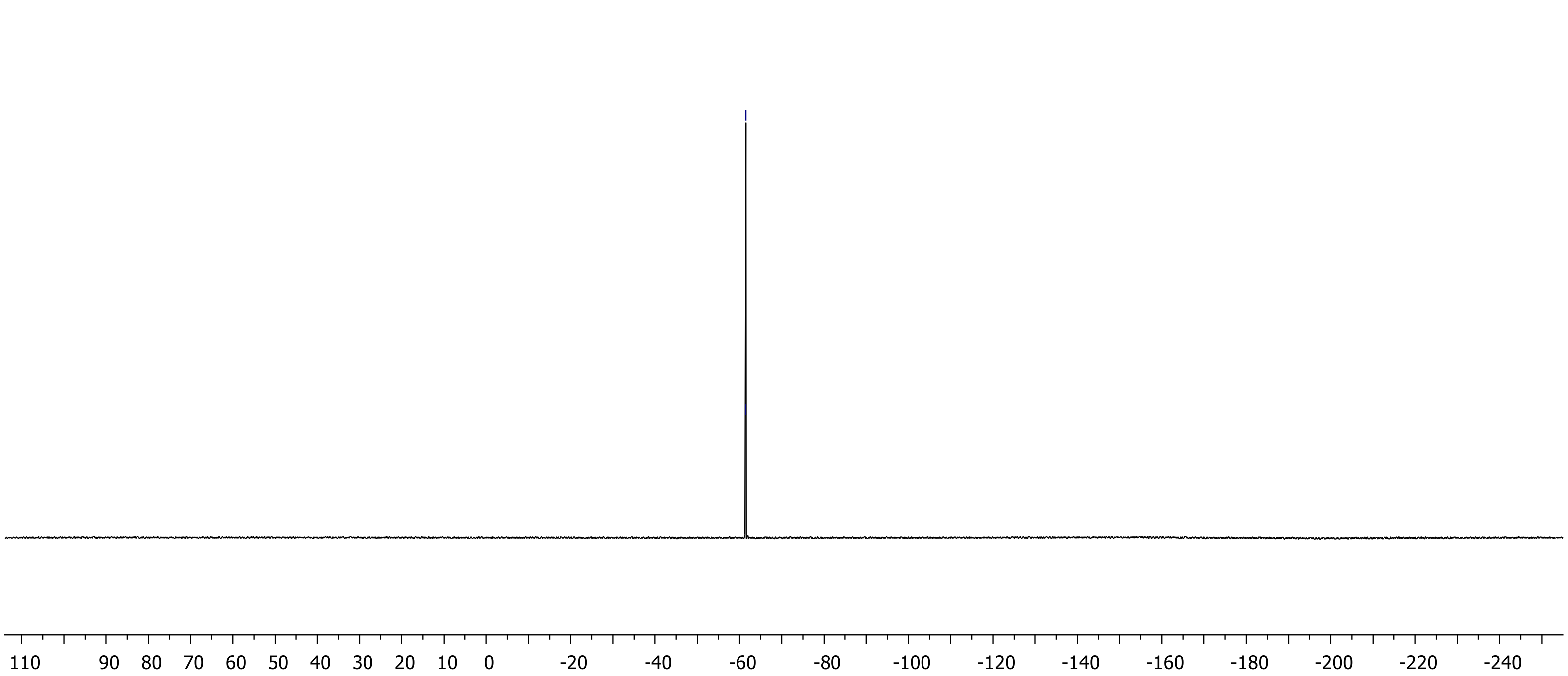

Compound 12

^{1}H NMR (500 MHz, $CDCl_3$)

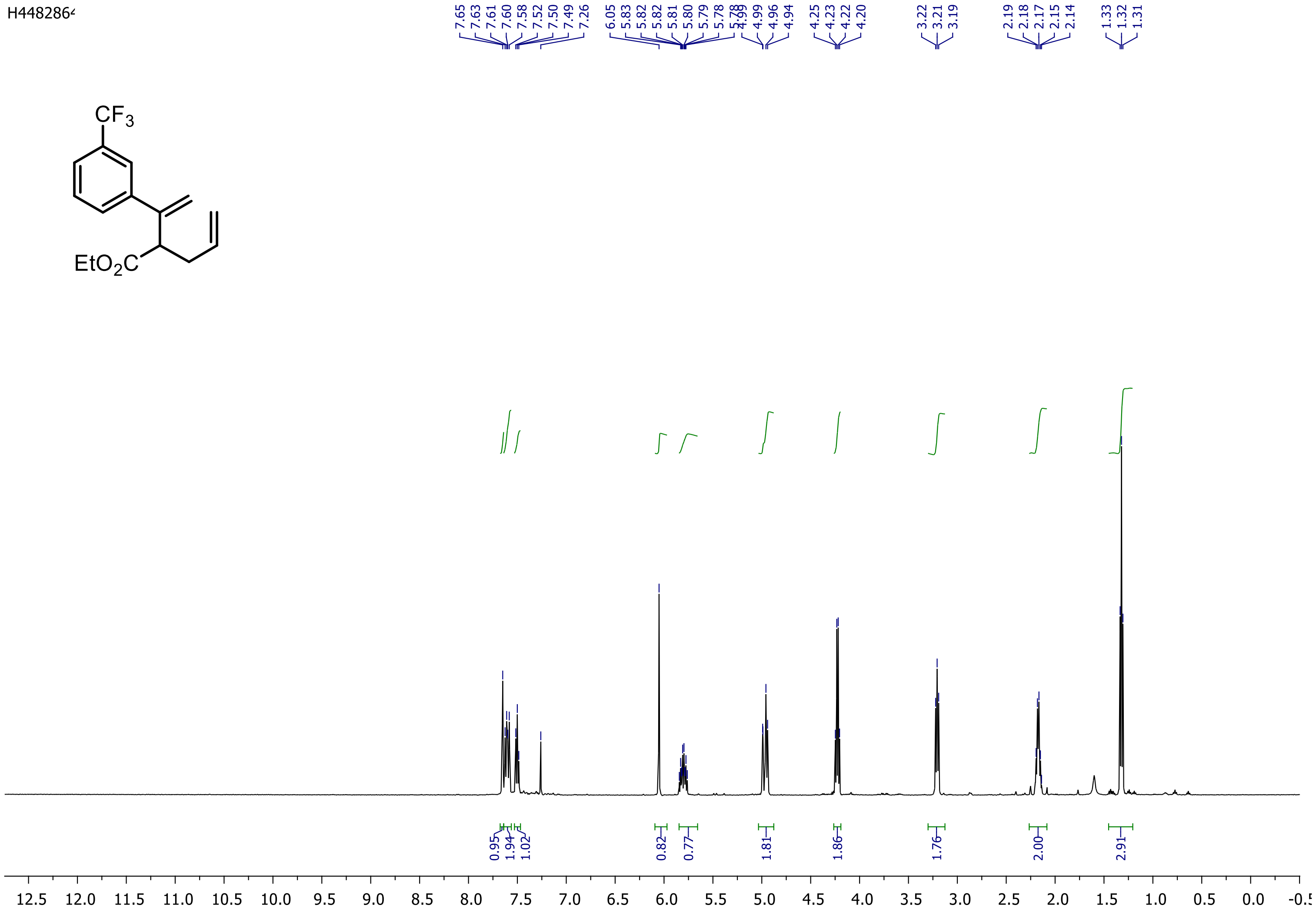

$^{13}C\{^1H\}$ NMR (126 MHz, $CDCl_3$)

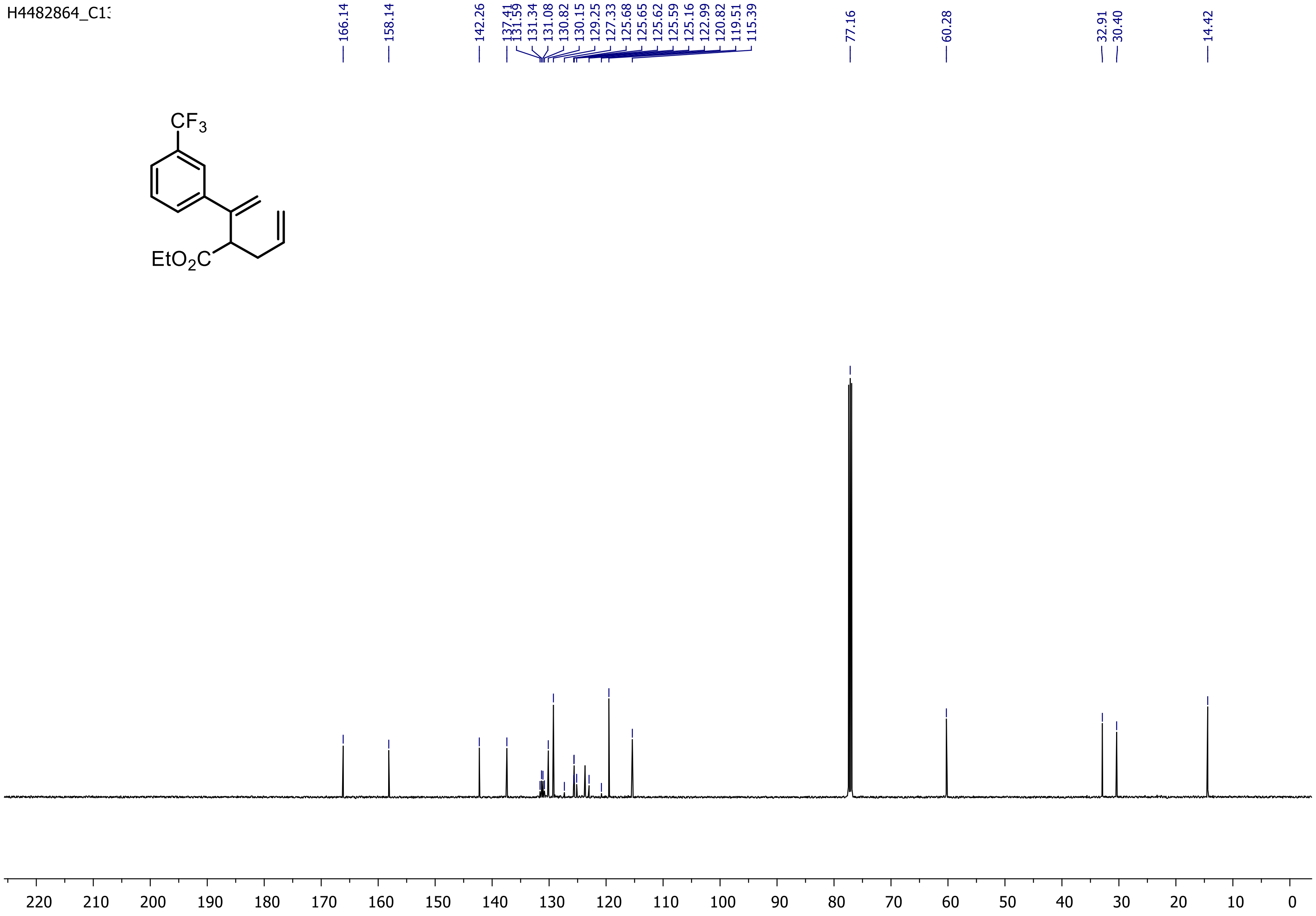

$^{19}F\{^1H\}$ NMR (376 MHz, $CDCl_3$)

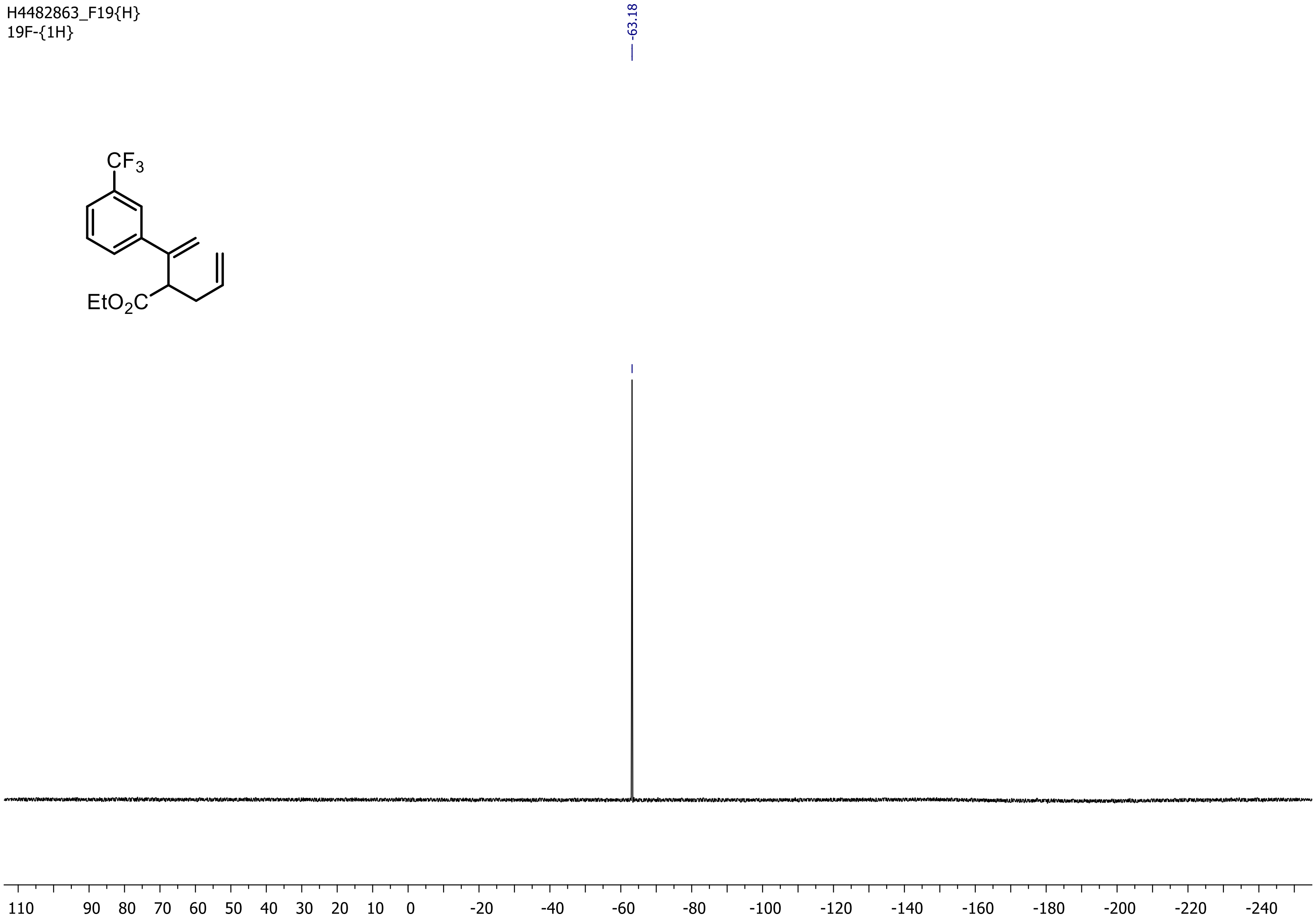

Compound (±)-12a (the sample contains ca. 10% of the isomeric 1,5-disubstituted bicyclo[2.1.1]hexane)

^{1}H NMR (500 MHz, $CDCl_3$)

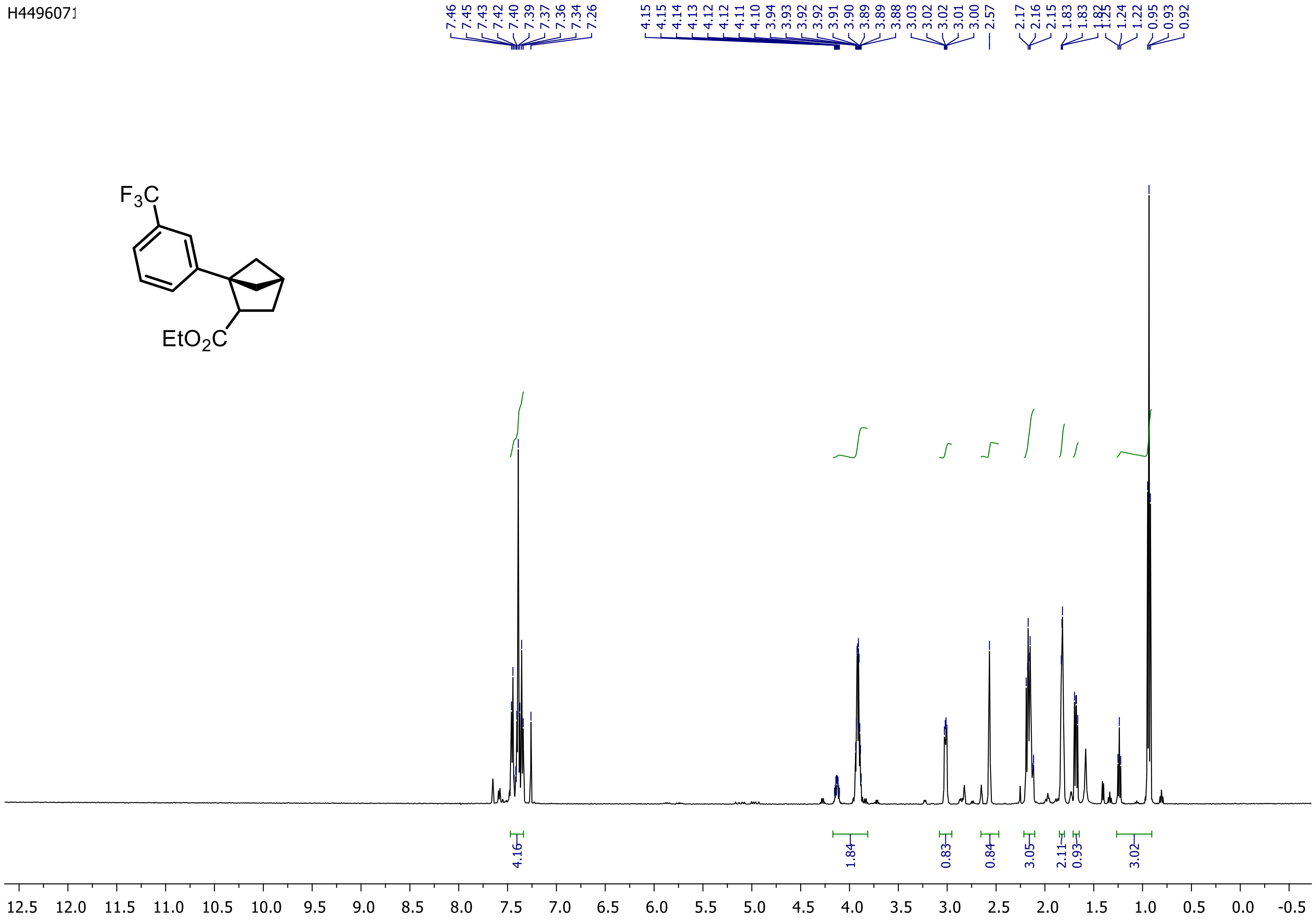

$^{13}C\{^1H\}$ NMR (126 MHz, $CDCl_3$)

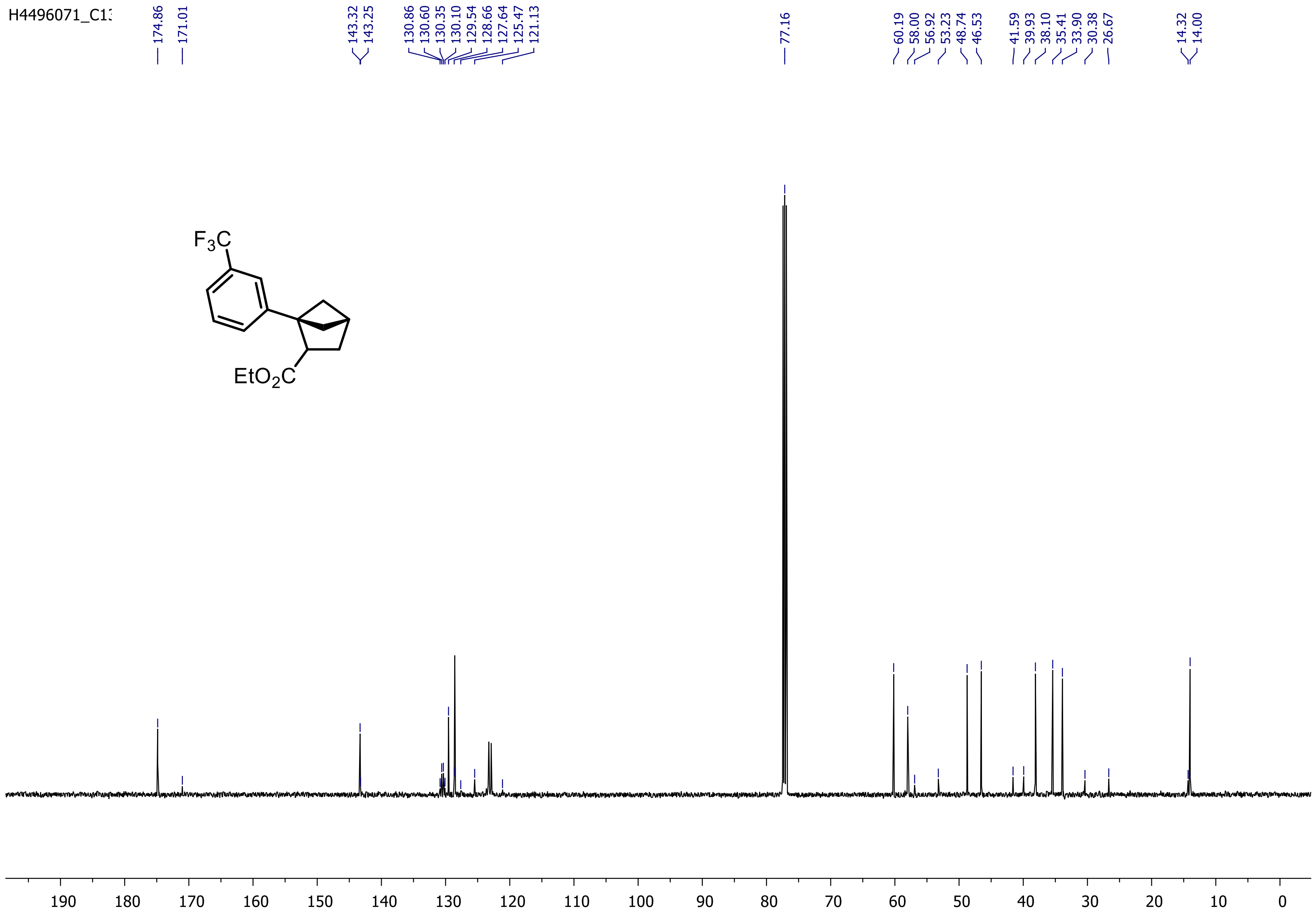

$^{19}F\{^{1}H\}$ NMR (376 MHz, $CDCl_3$)

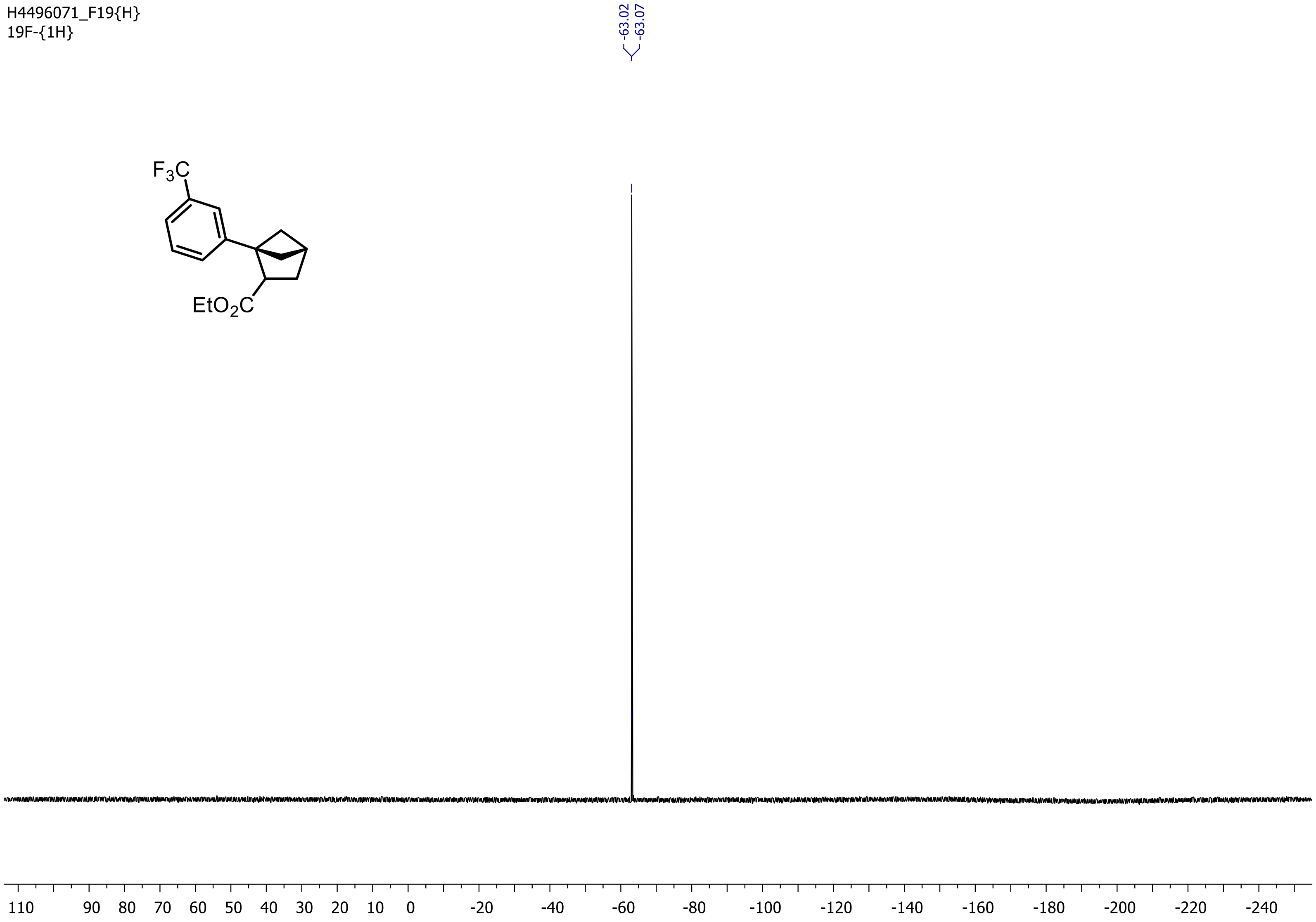

Compound (±)-12b

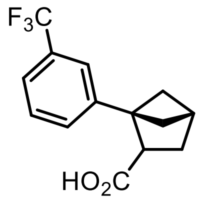

^{1}H NMR (500 MHz, DMSO-d$_6$)

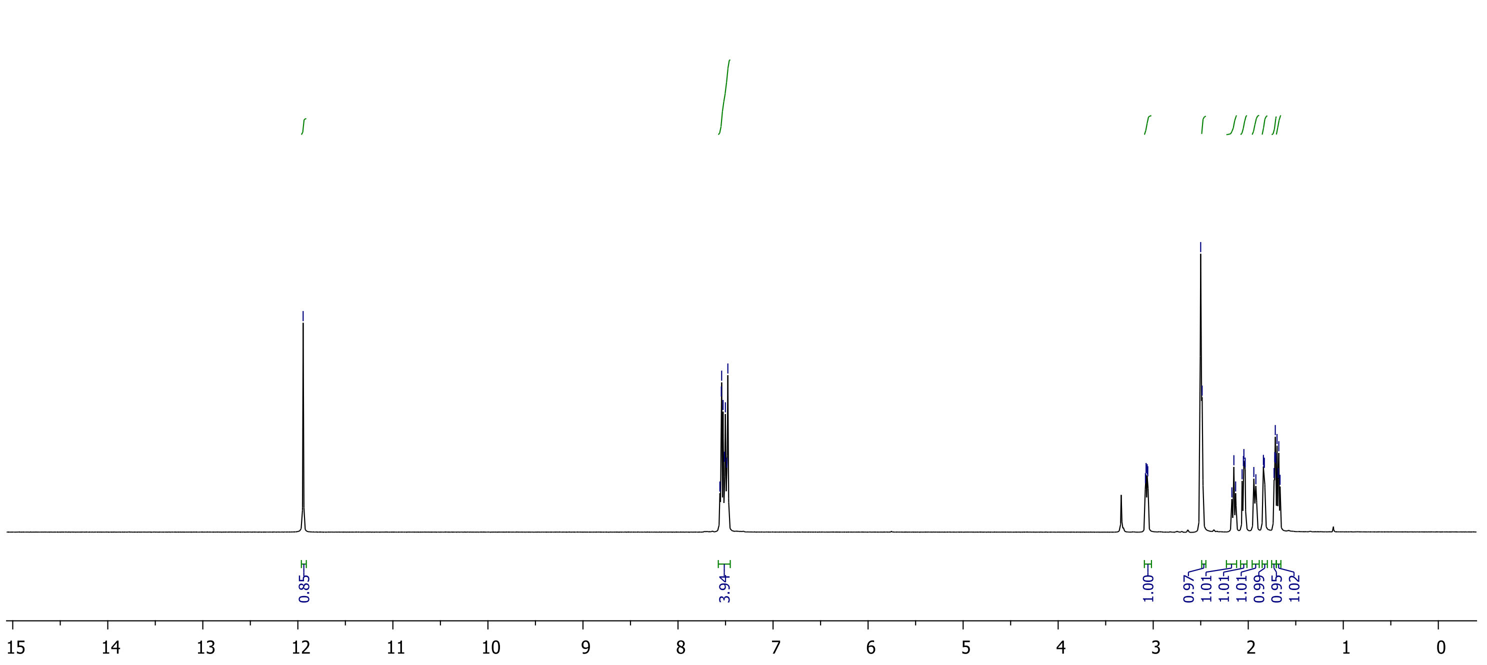

$^{13}C\{^{1}H\}$ NMR (126 MHz, DMSO-d_6)

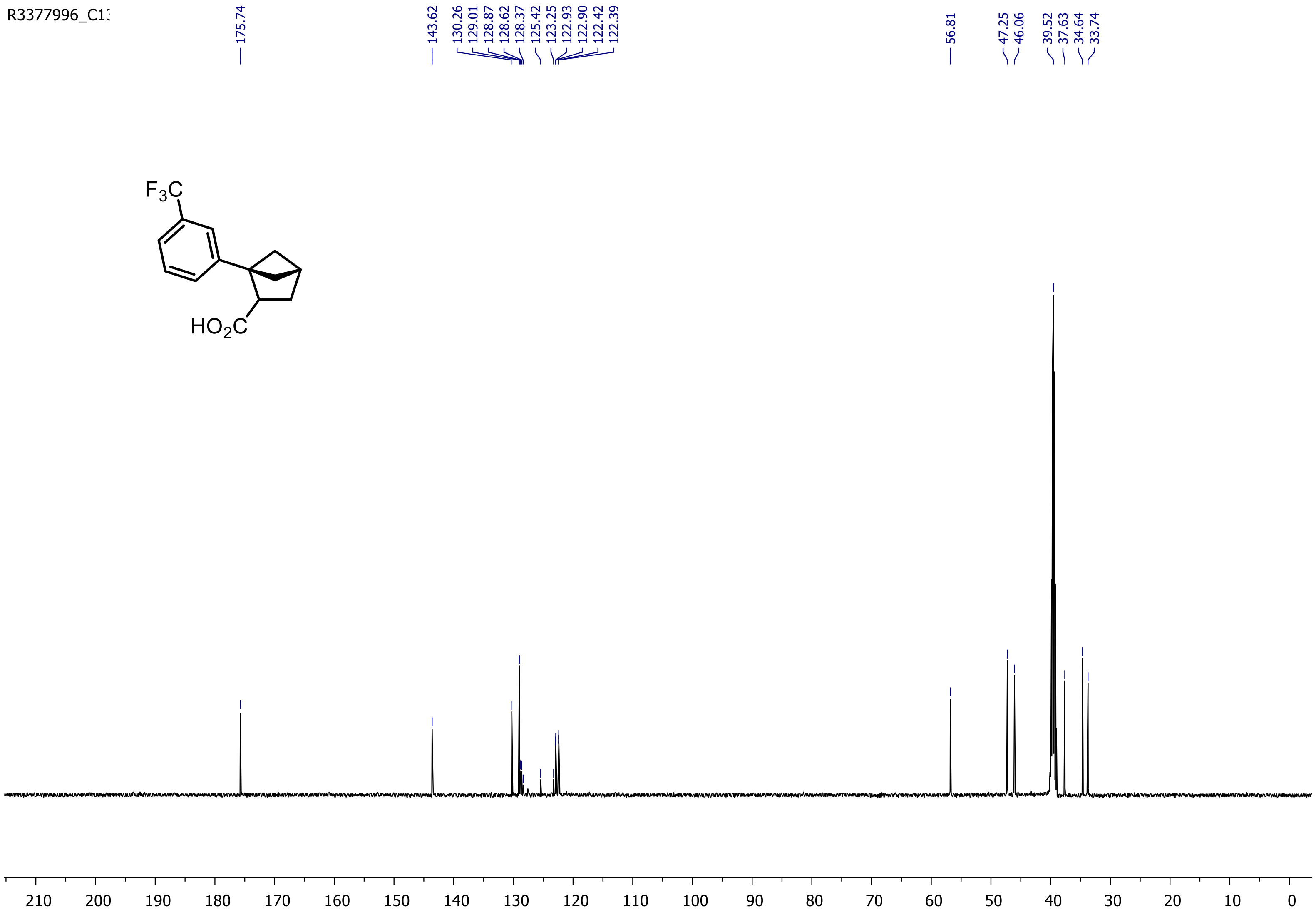

$^{19}F\{^{1}H\}$ NMR (376 MHz, DMSO-d_6)

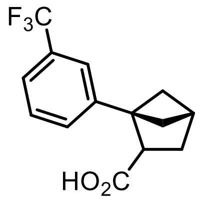

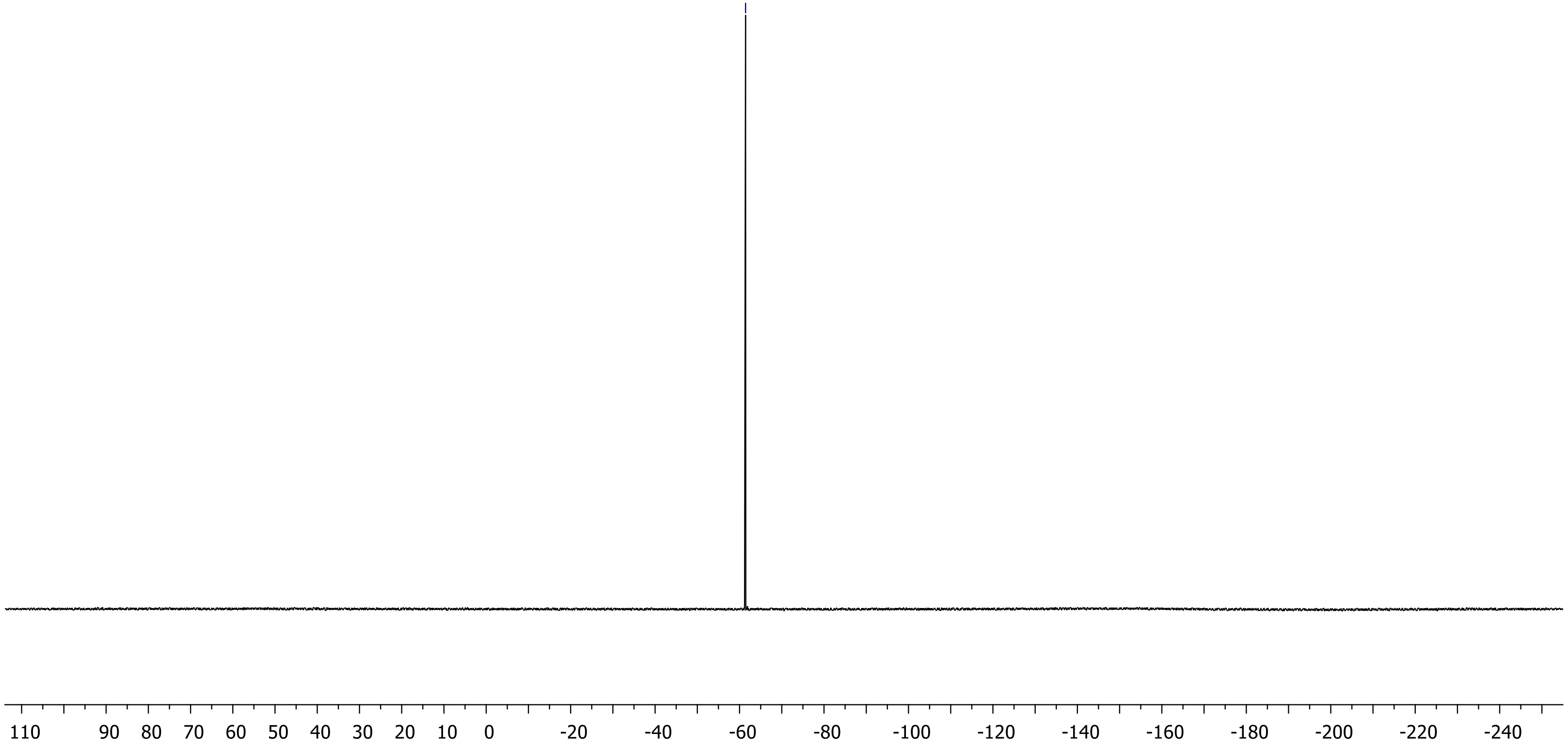

Ethyl-3-(3,4-dichlorophenyl)but-2-enoate

^{1}H NMR (500 MHz, $CDCl_3$)

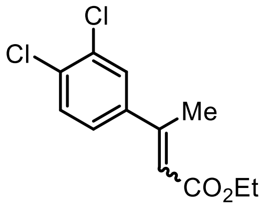

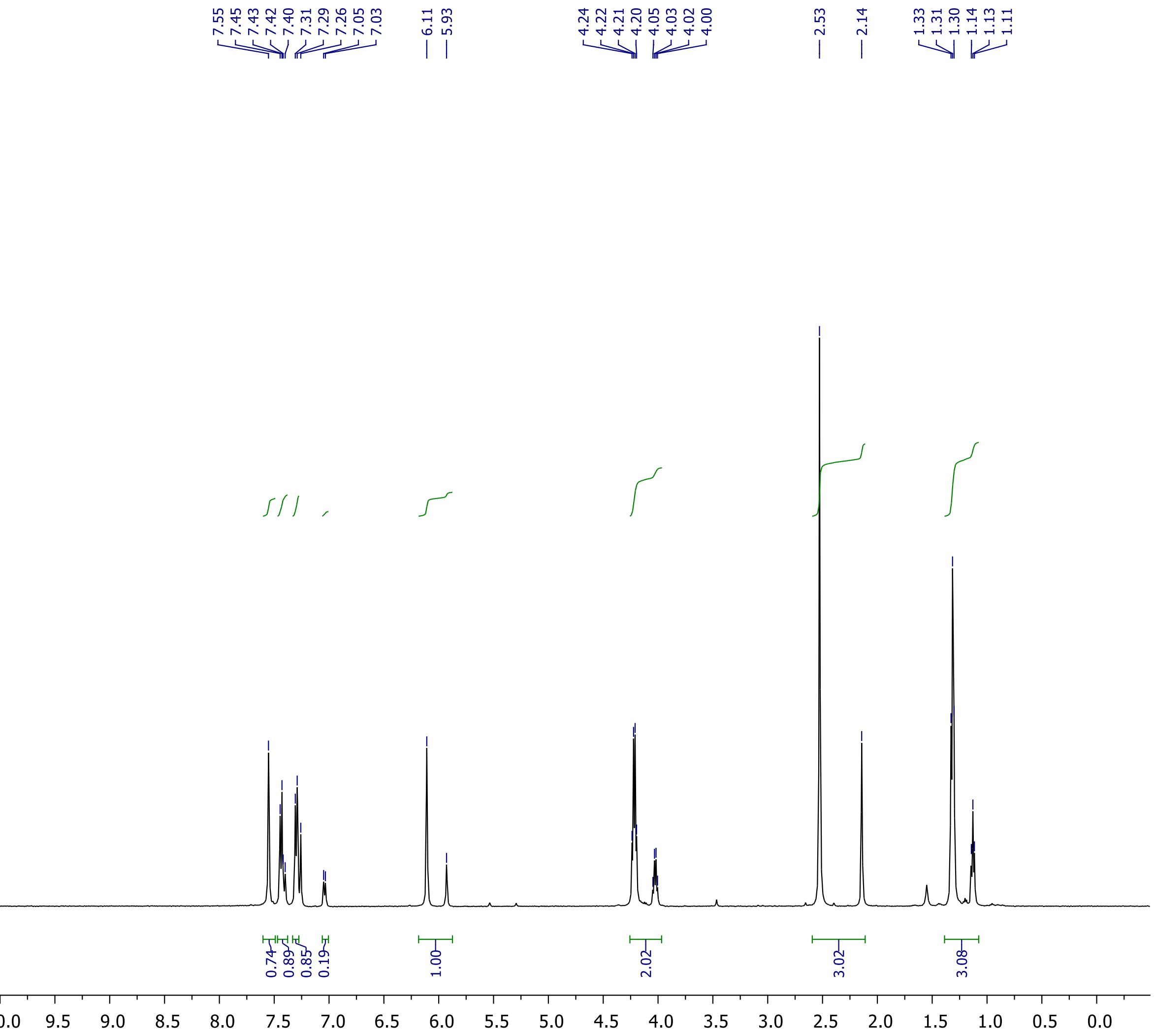

$^{13}C\{^{1}H\}$ NMR (151 MHz, $CDCl_3$)

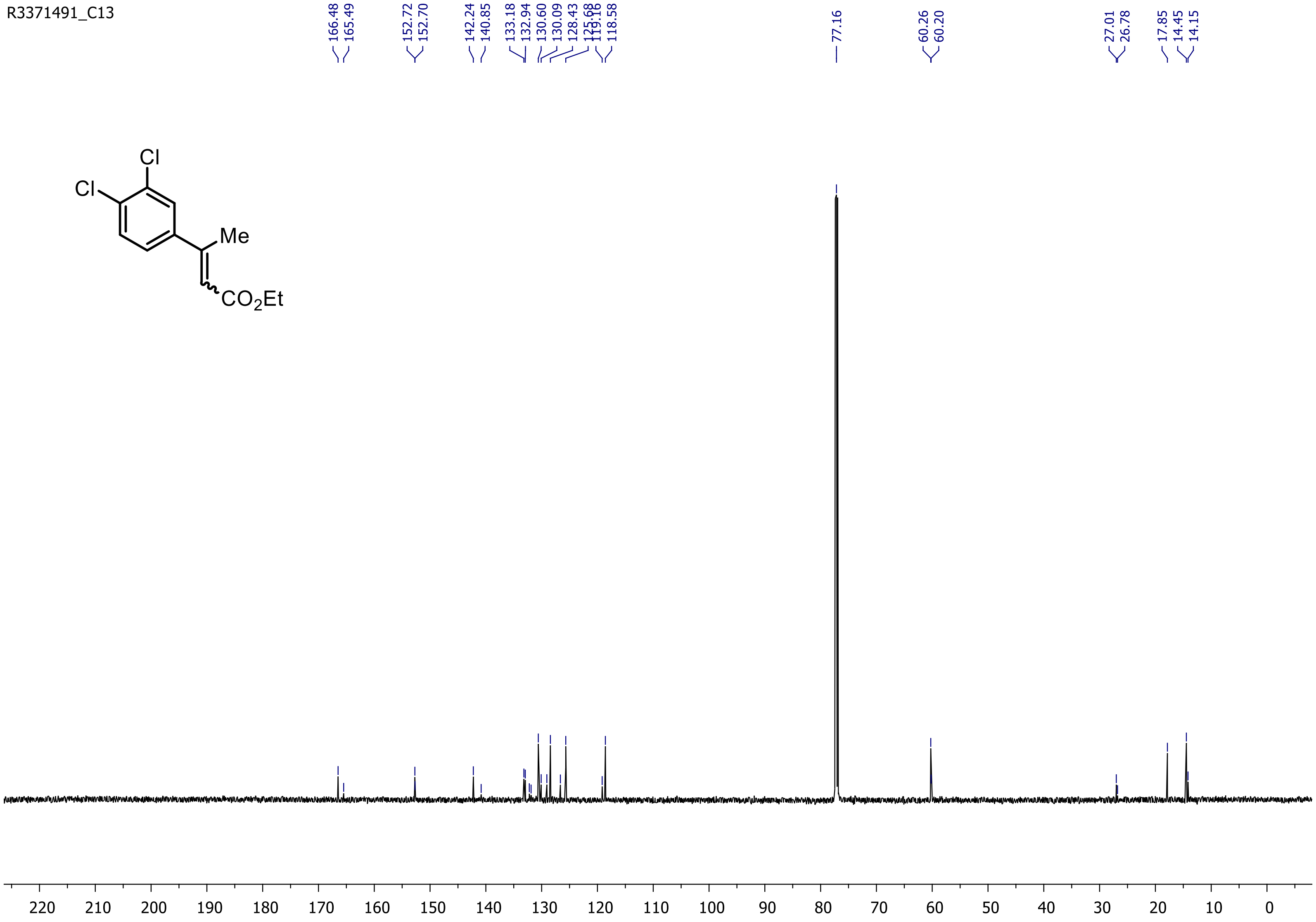

Compound 13

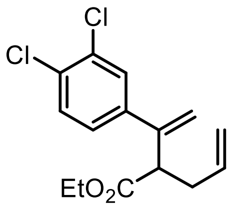

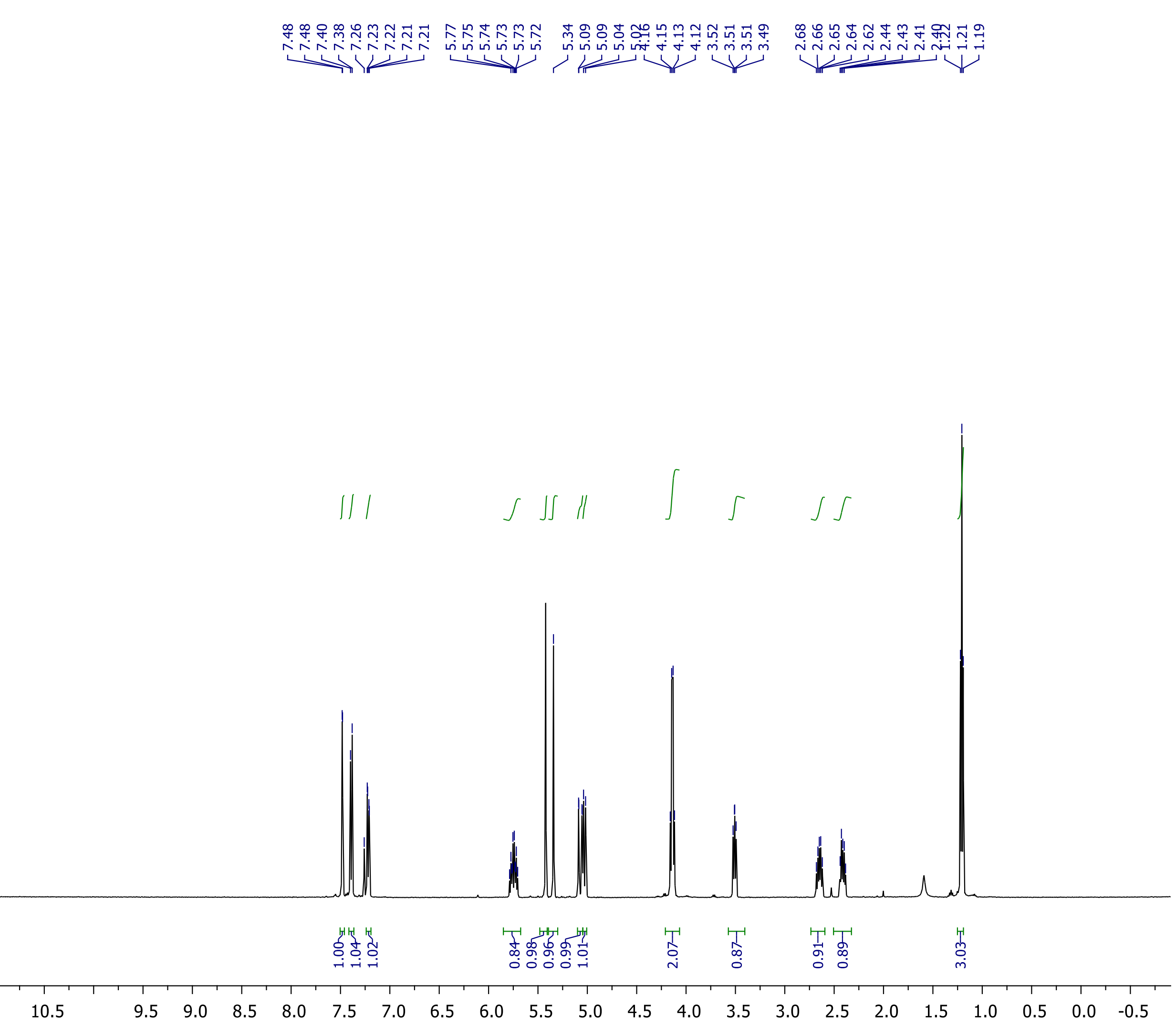

$^{13}C\{^1H\}$ NMR (126 MHz, $CDCl_3$)

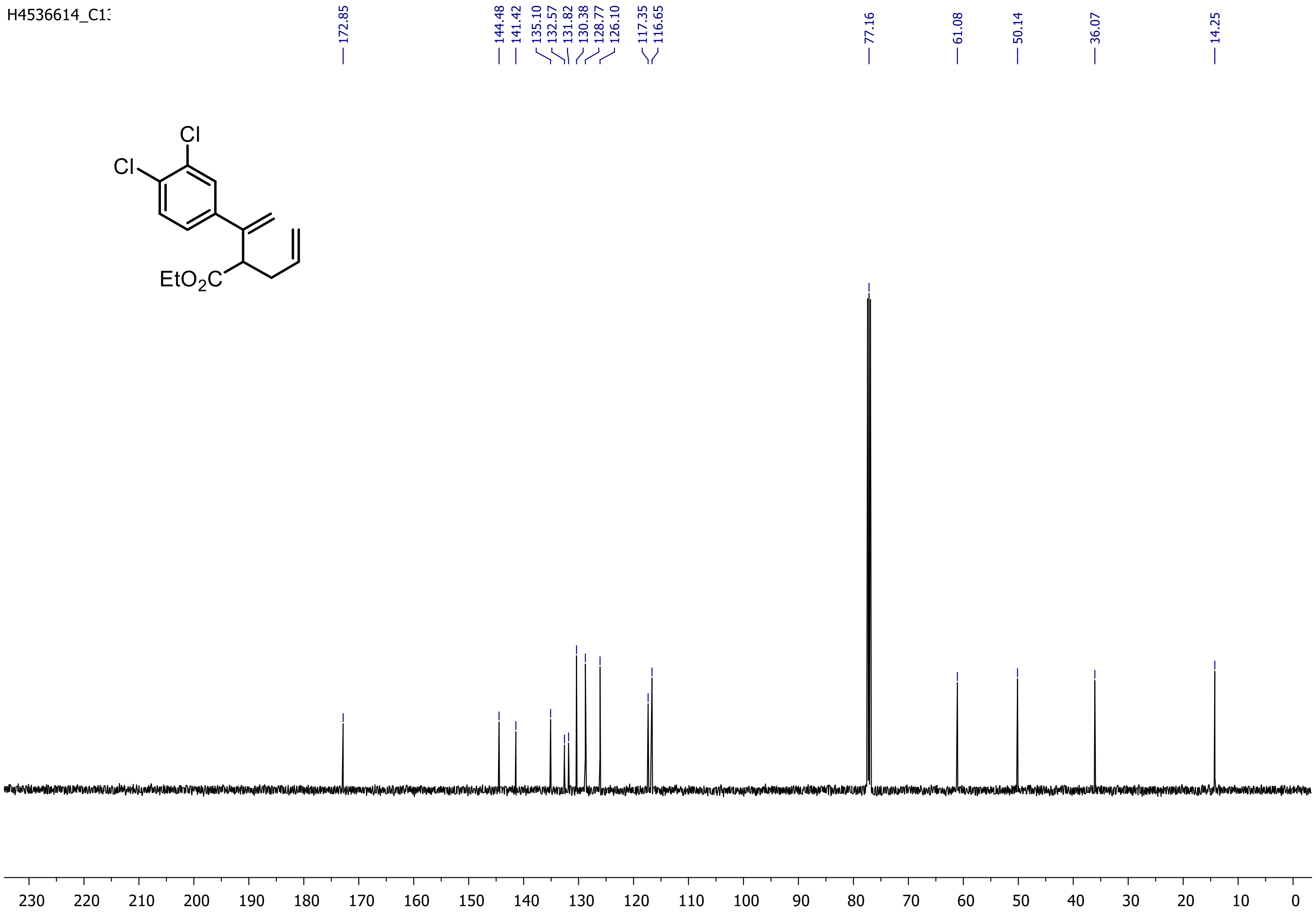

Compound (±)-13a

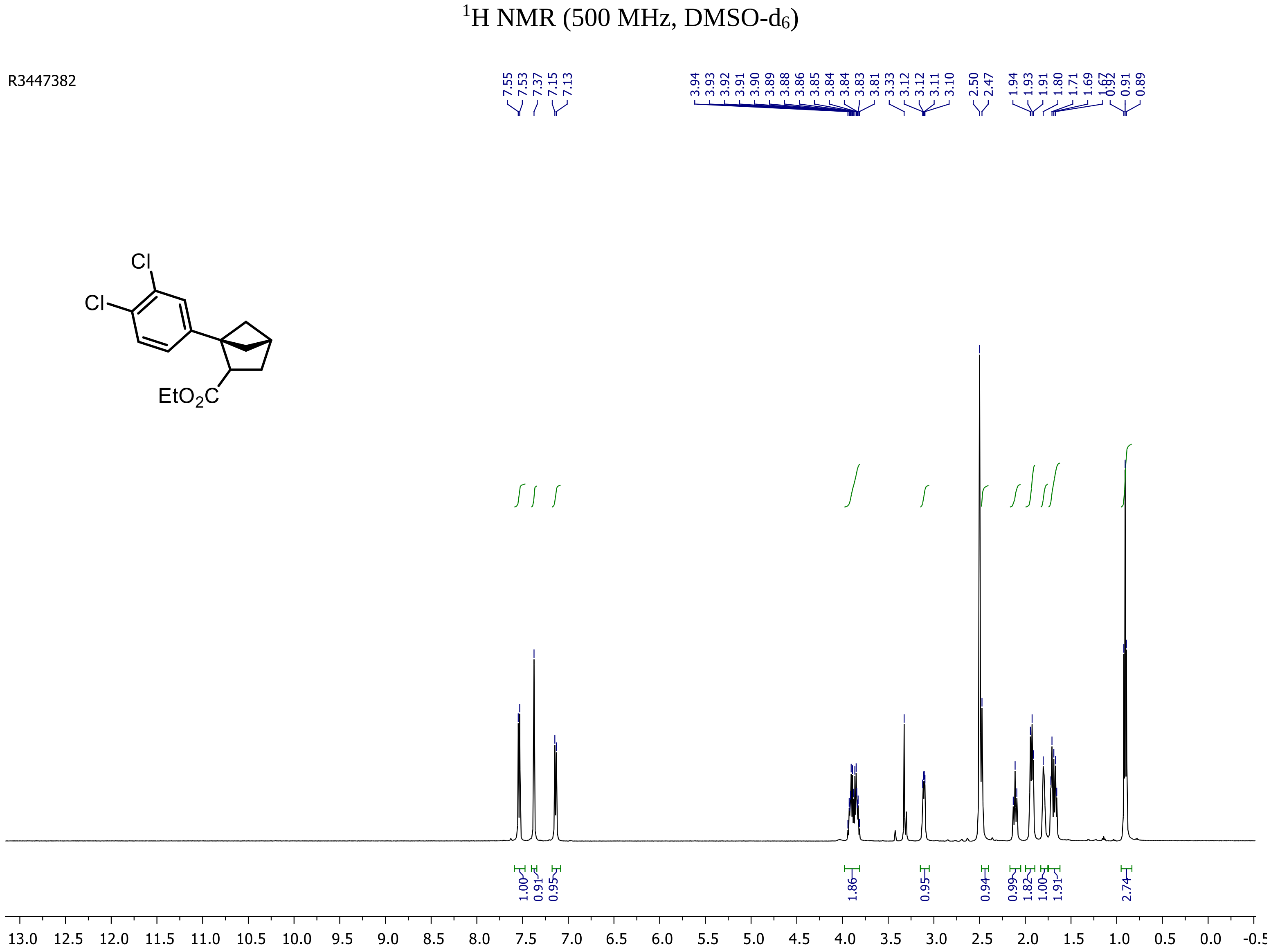

$^{13}C\{^1H\}$ NMR (126 MHz, DMSO-d_6)

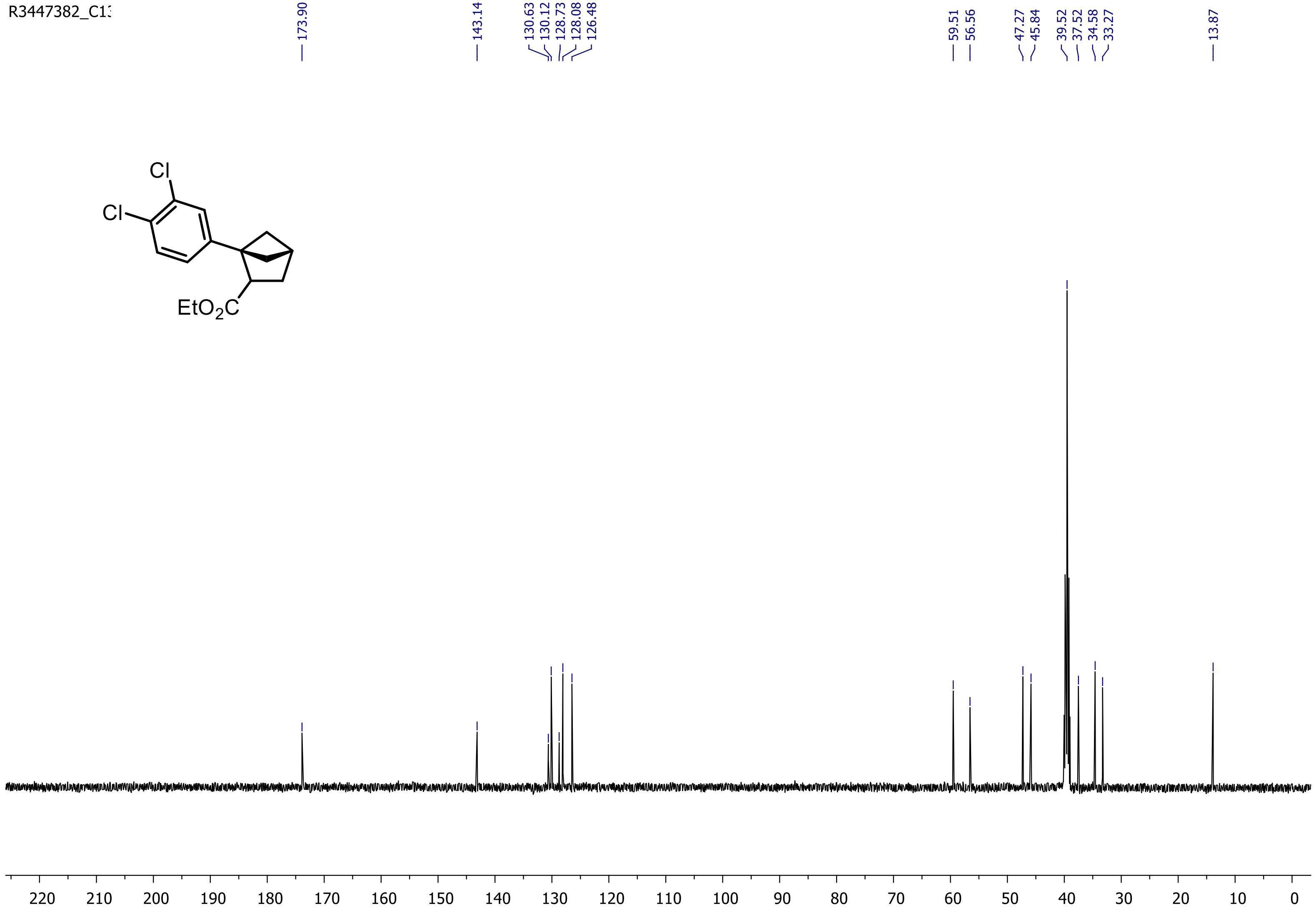

Compound (±)-13b

^{1}H NMR (500 MHz, DMSO-d$_6$)

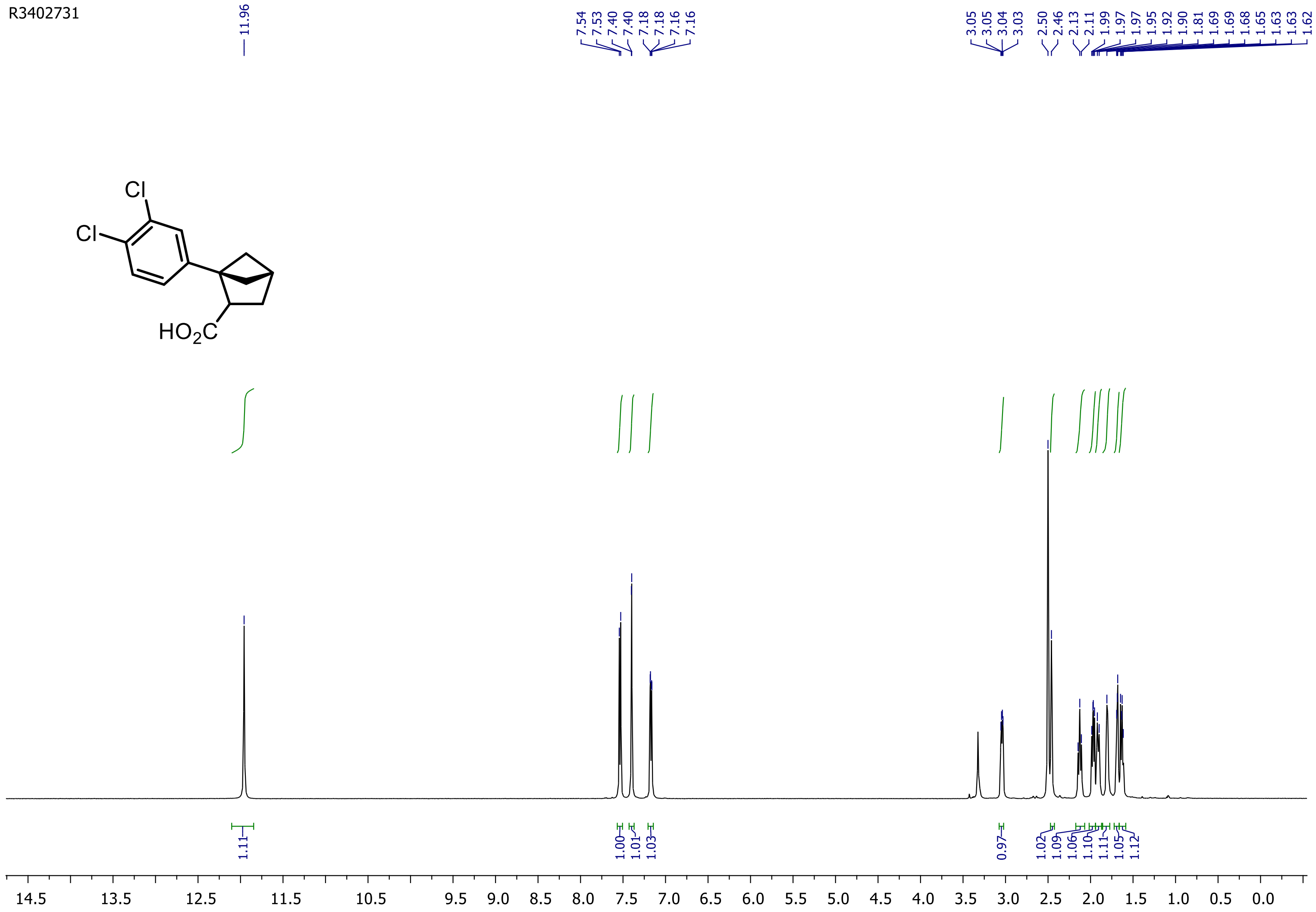

$^{13}C\{^1H\}$ NMR (151 MHz, DMSO-d_6)

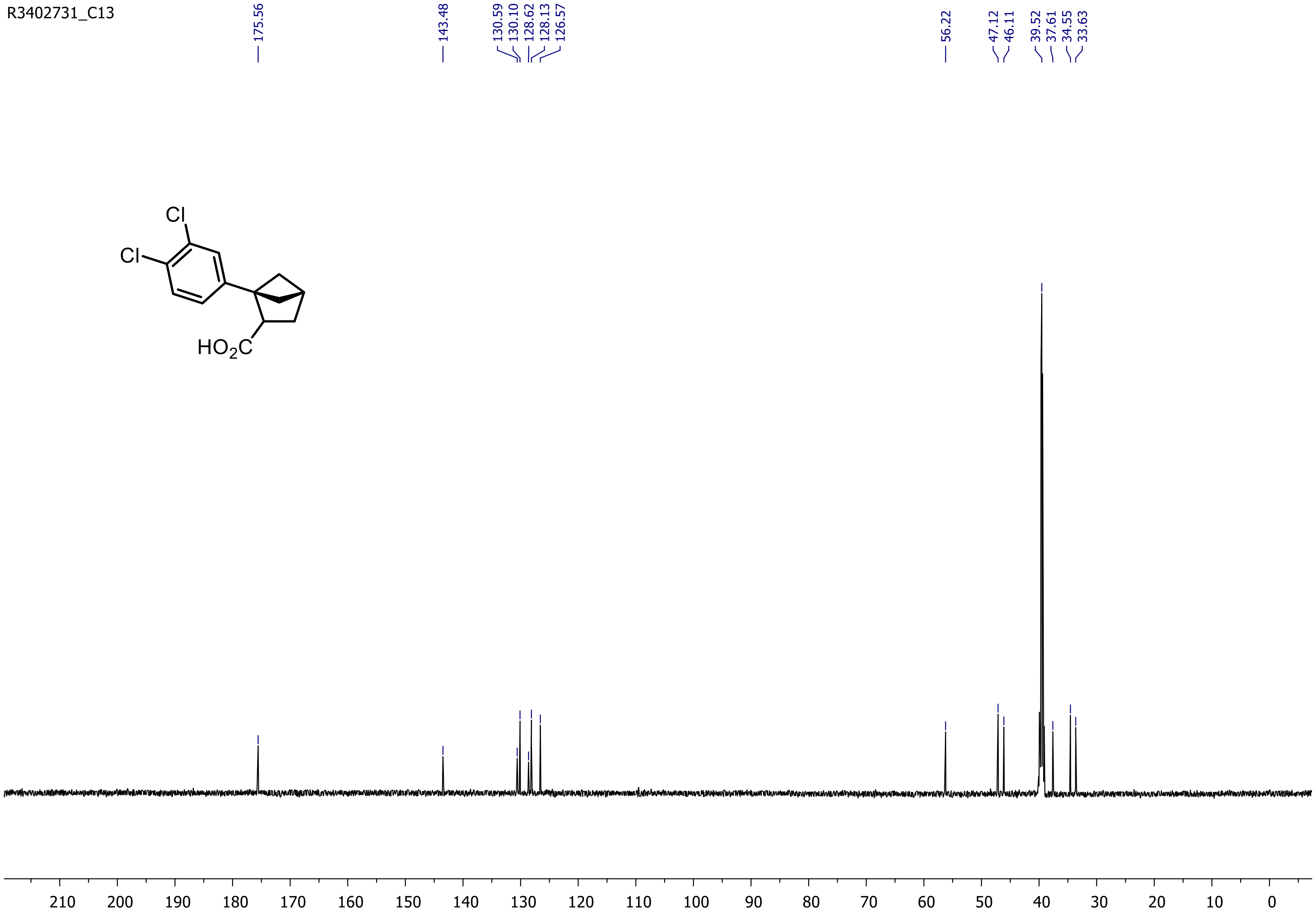

Ethyl-3-(3,4,5-trifluorophenyl)but-2-enoate

^{1}H NMR (500 MHz, $CDCl_3$)

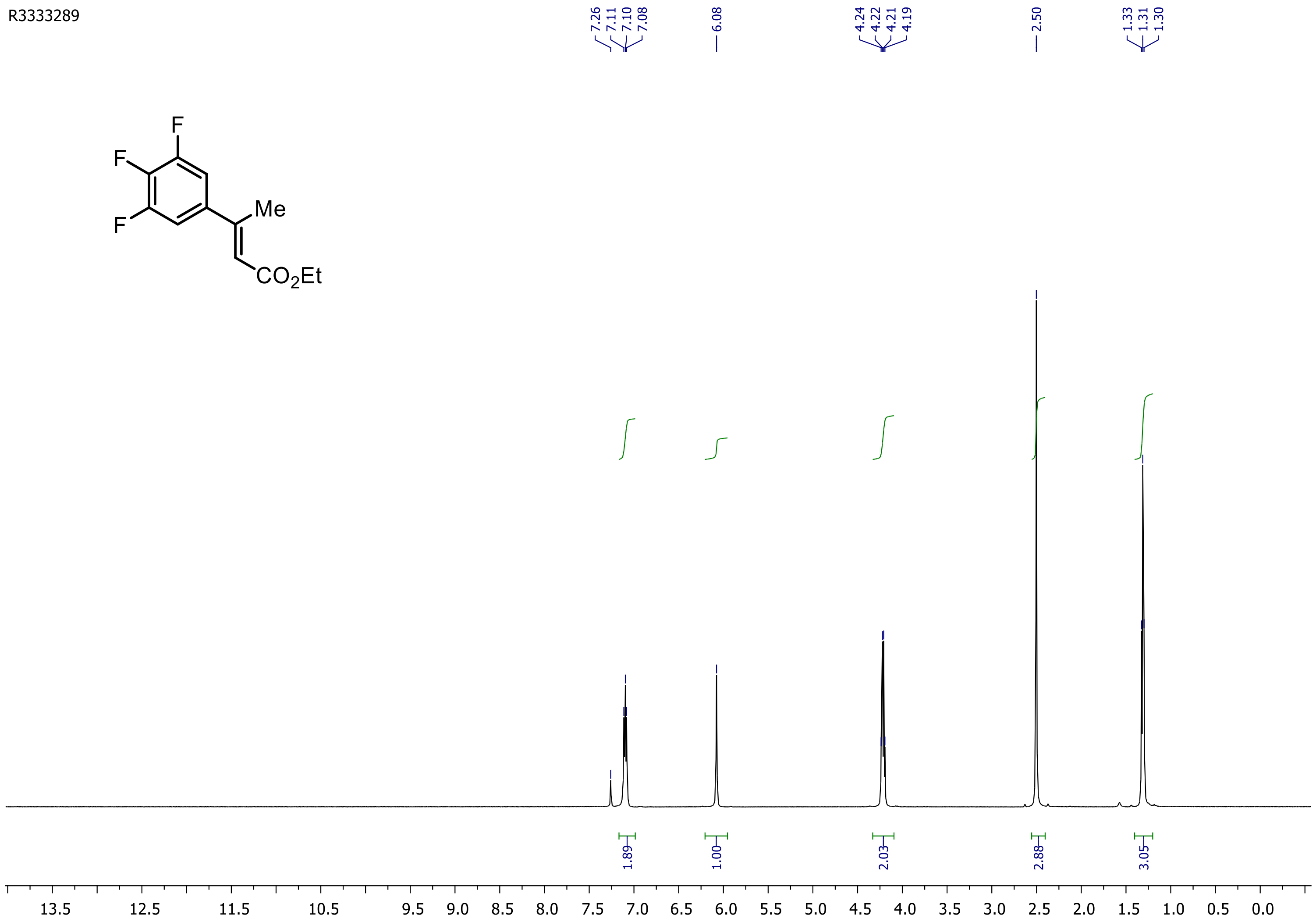

$^{13}C\{^{1}H\}$ NMR (151 MHz, $CDCl_3$)

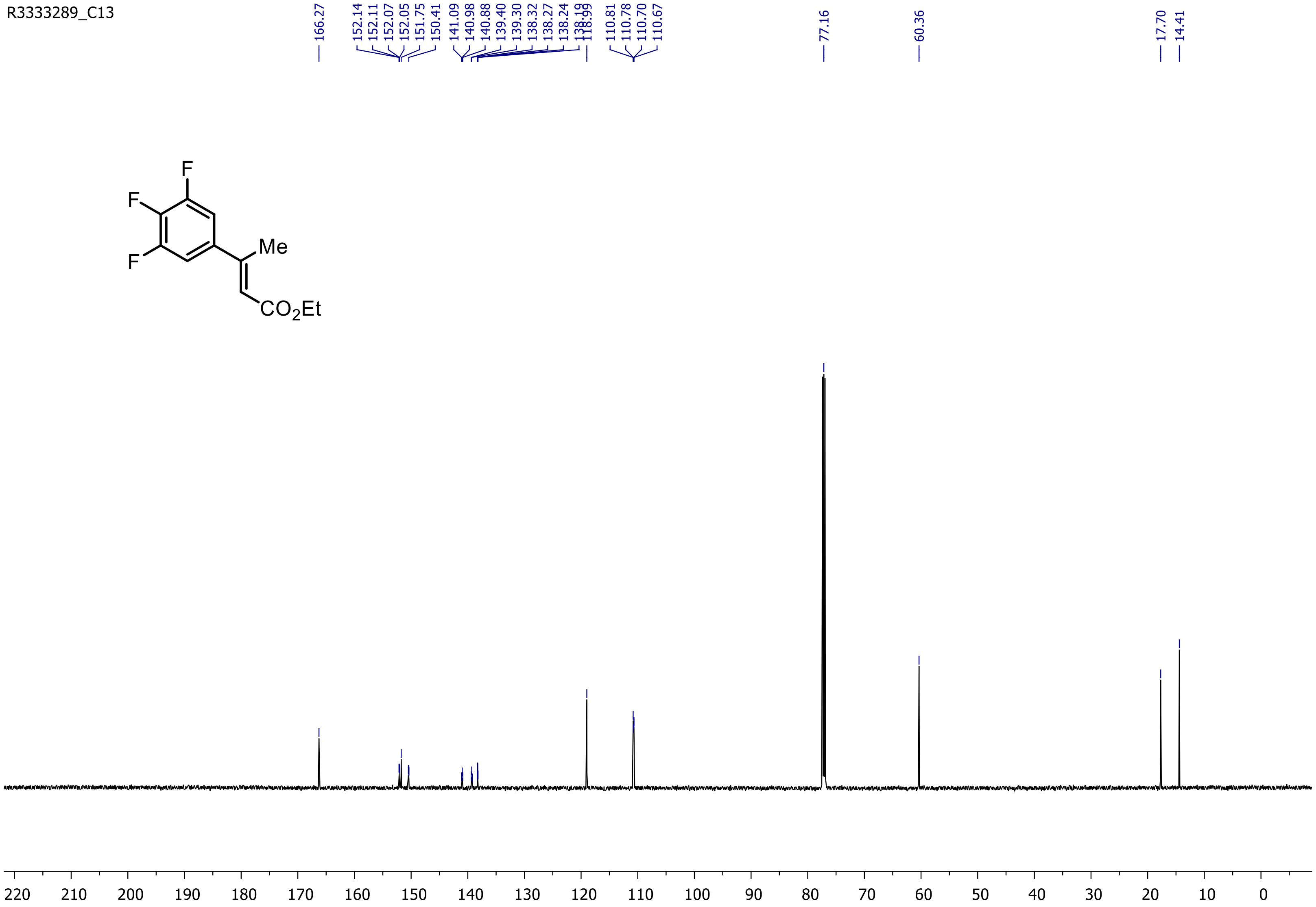

$^{19}F\{^{1}H\}$ NMR (376 MHz, $CDCl_3$)

R3333289_F19{H}

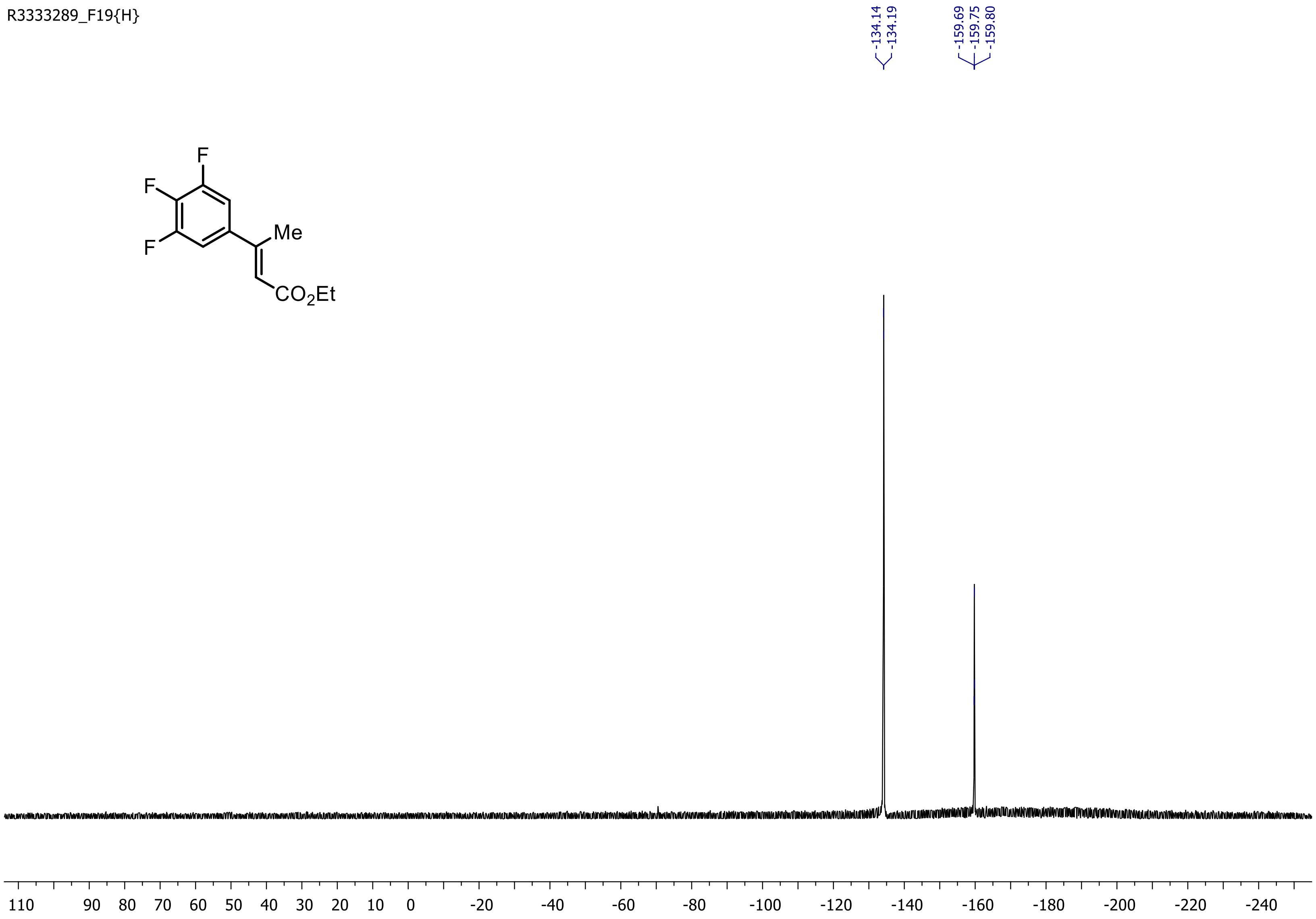

Compound 14

^{1}H NMR (500 MHz, $CDCl_3$)

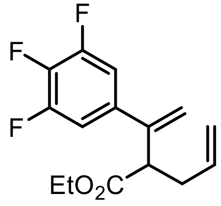

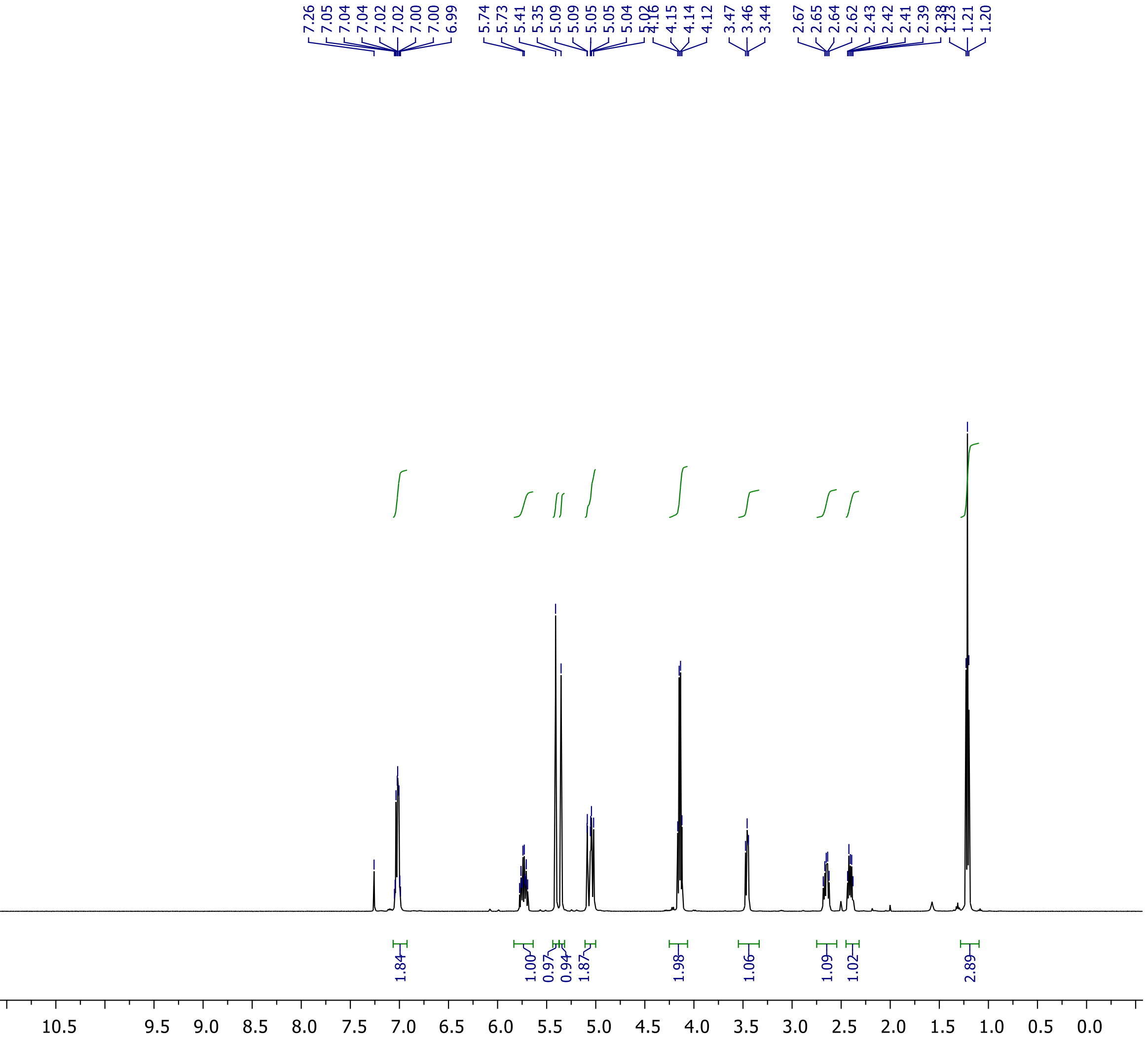

$^{13}C\{^{1}H\}$ NMR (151 MHz, $CDCl_3$)

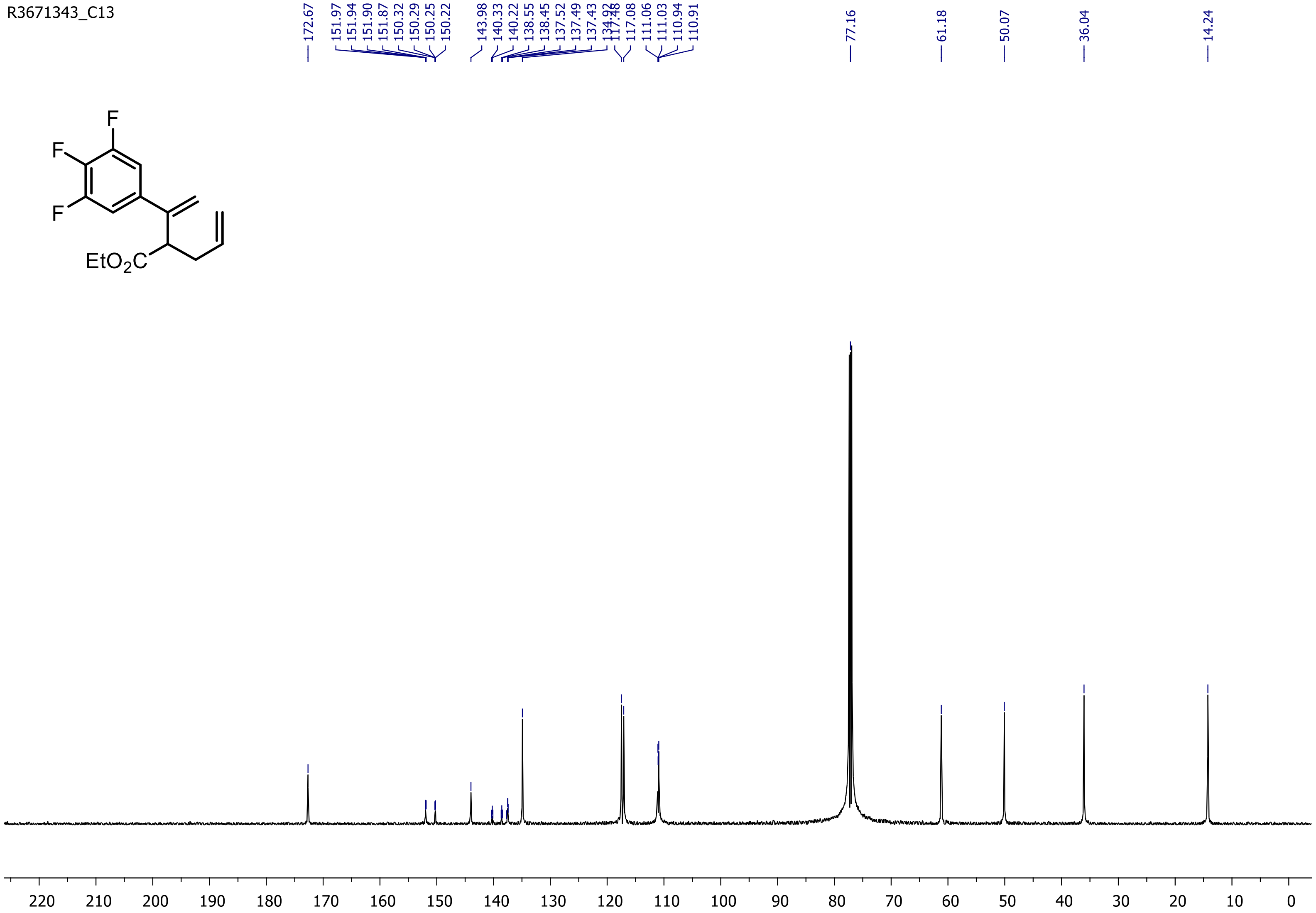

$^{19}F\{^1H\}$ NMR (376 MHz, $CDCl_3$)

R3671343_F19{H}

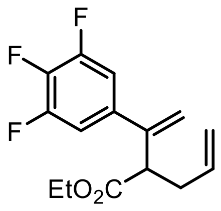

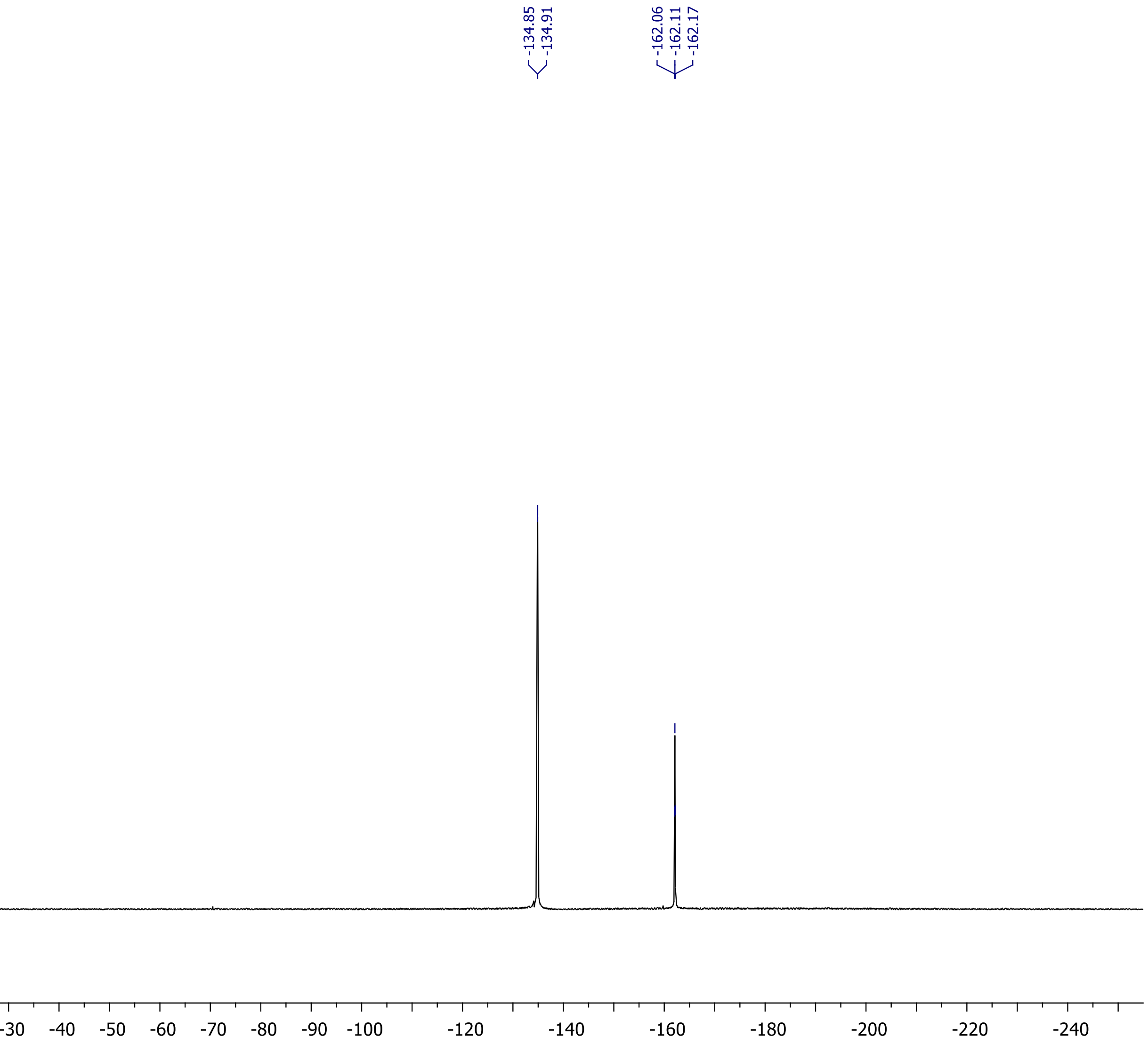

Compound (±)-14a

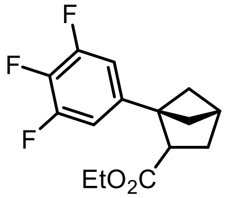

^{1}H NMR (500 MHz, $CDCl_3$)

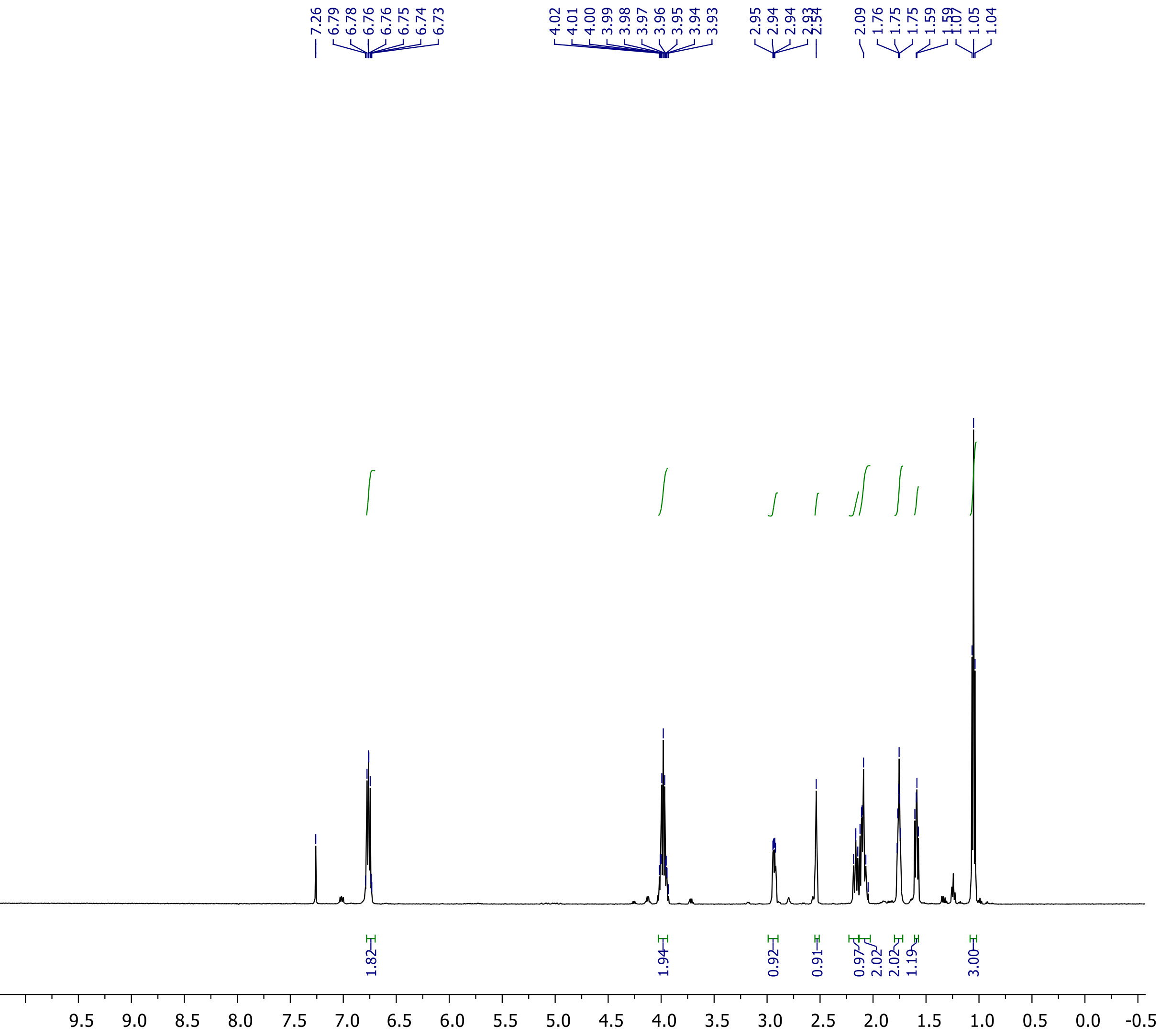

$^{13}C\{^{1}H\}$ NMR (126 MHz, $CDCl_3$)

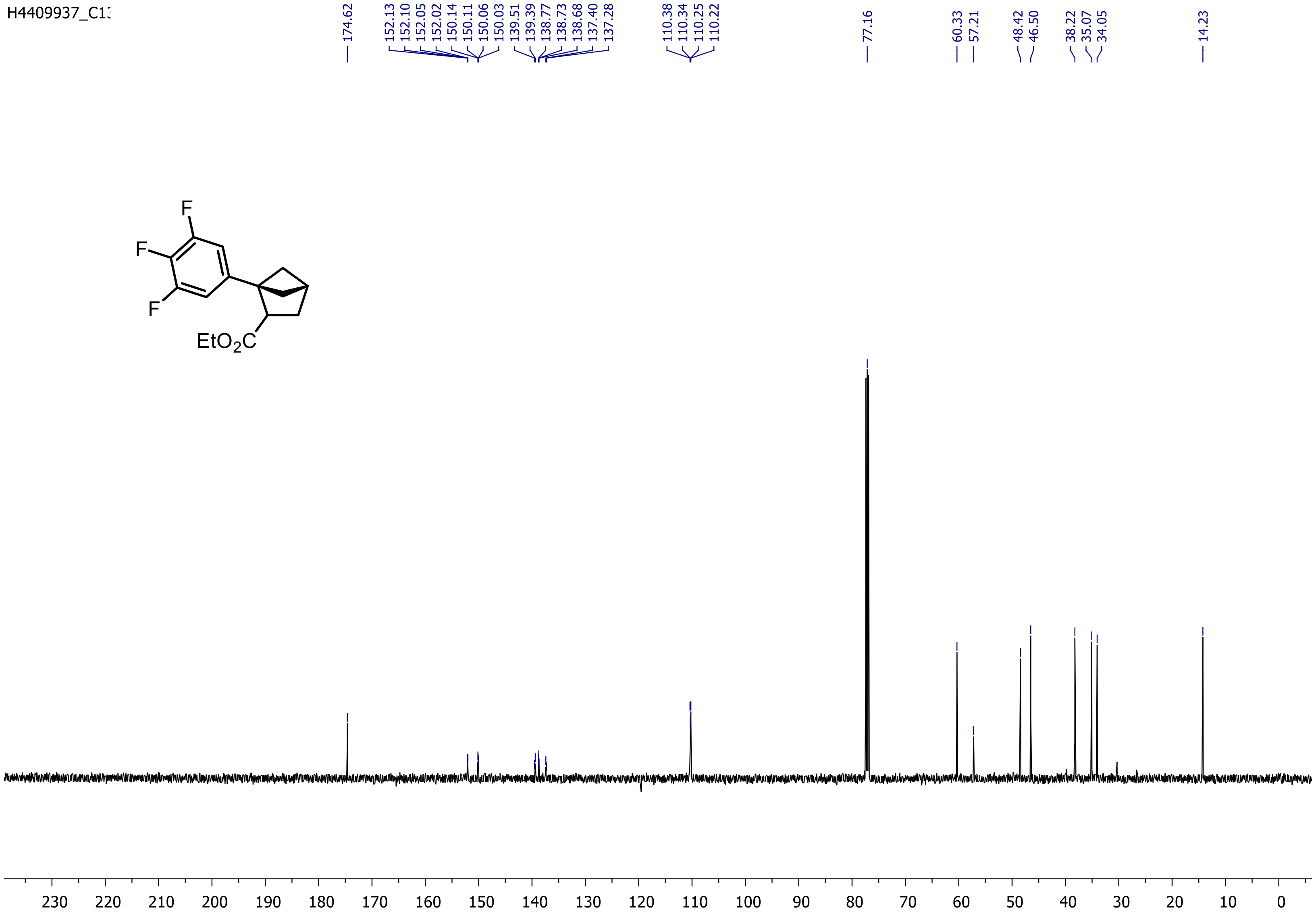

$^{19}F\{^{1}H\}$ NMR (376 MHz, $CDCl_3$)

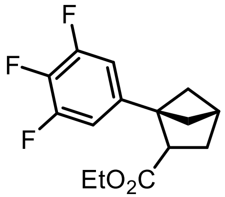

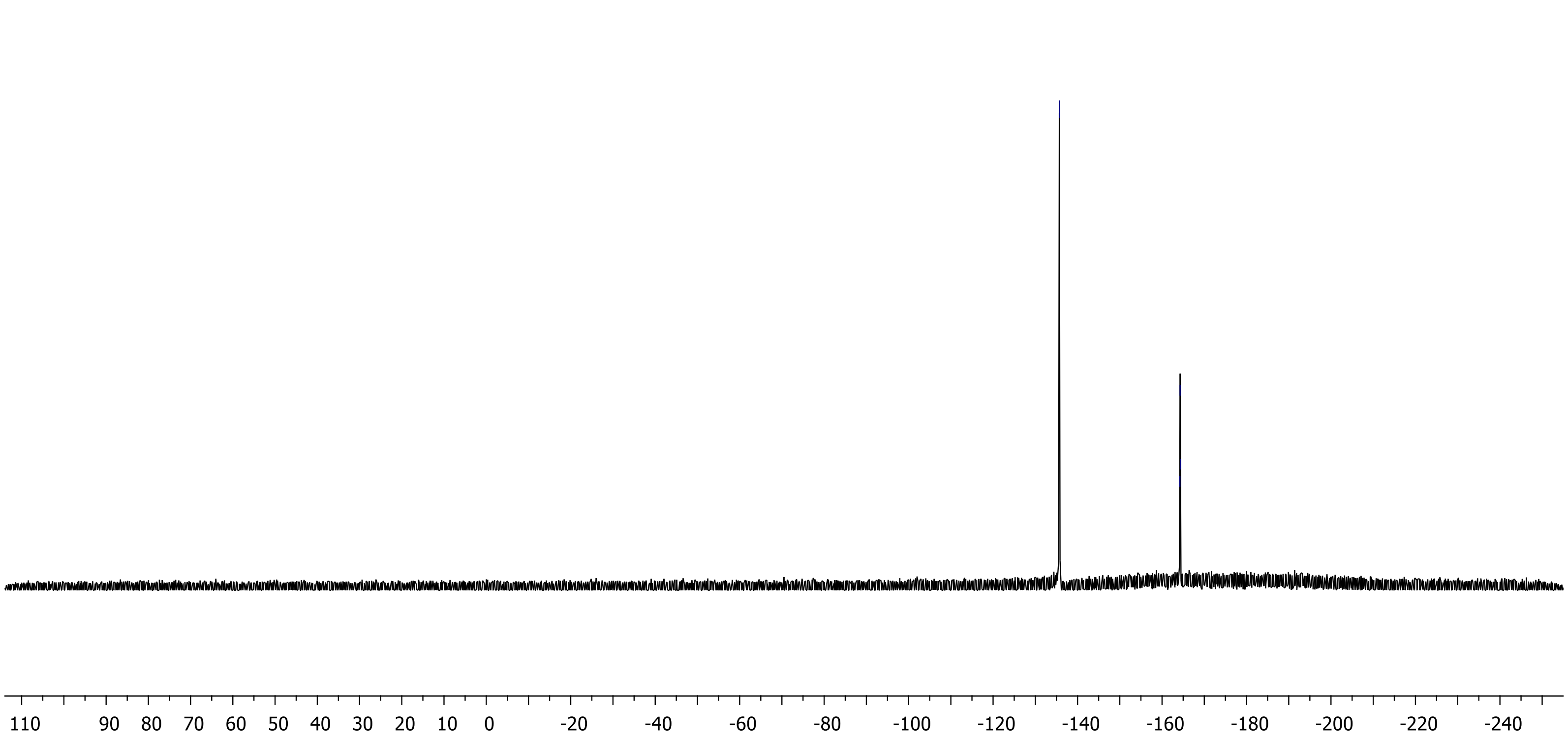

Compound (±)-14b

^{1}H NMR (500 MHz, DMSO-d_6)

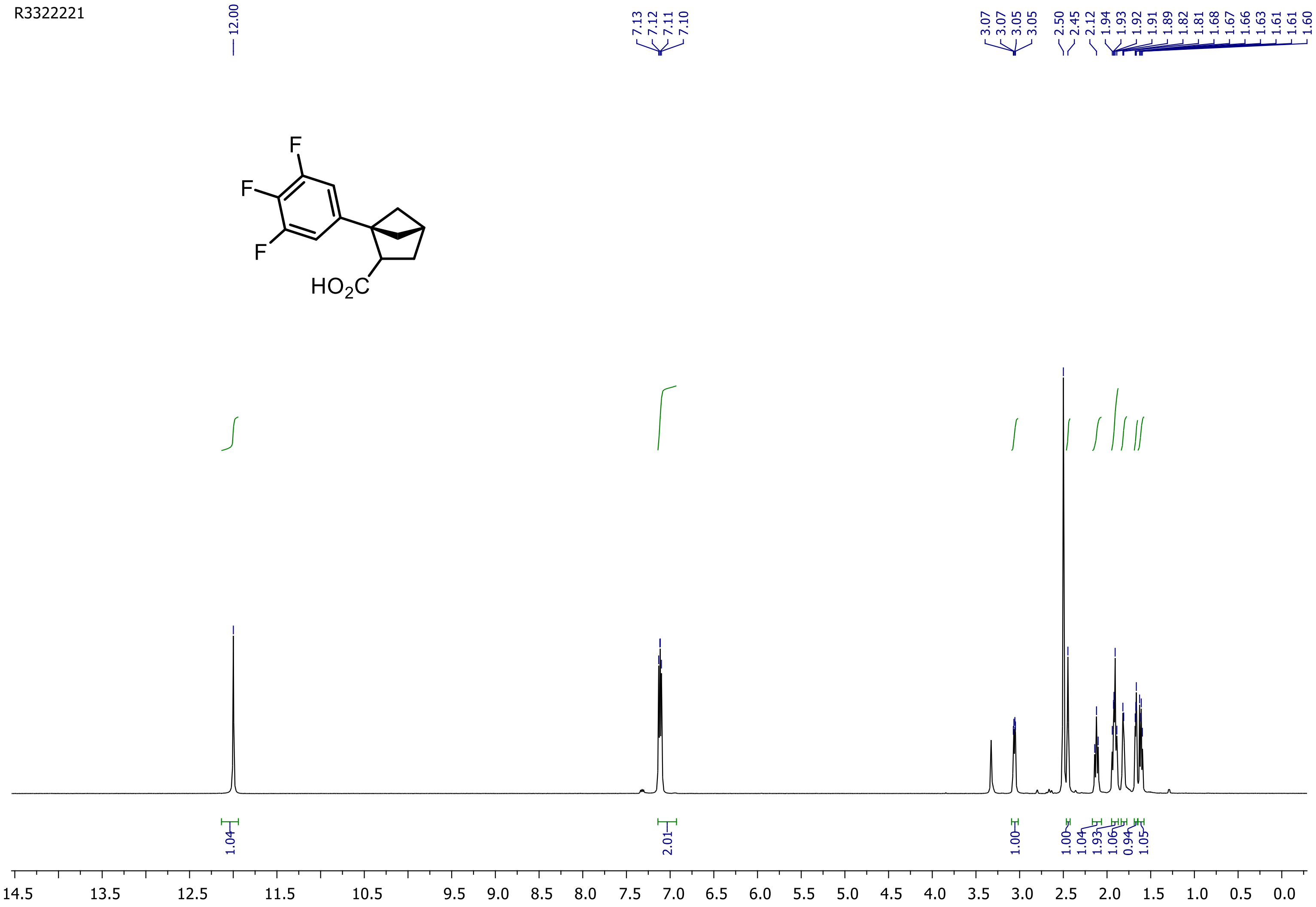

$^{13}C\{^{1}H\}$ NMR (151 MHz, DMSO-d_6)

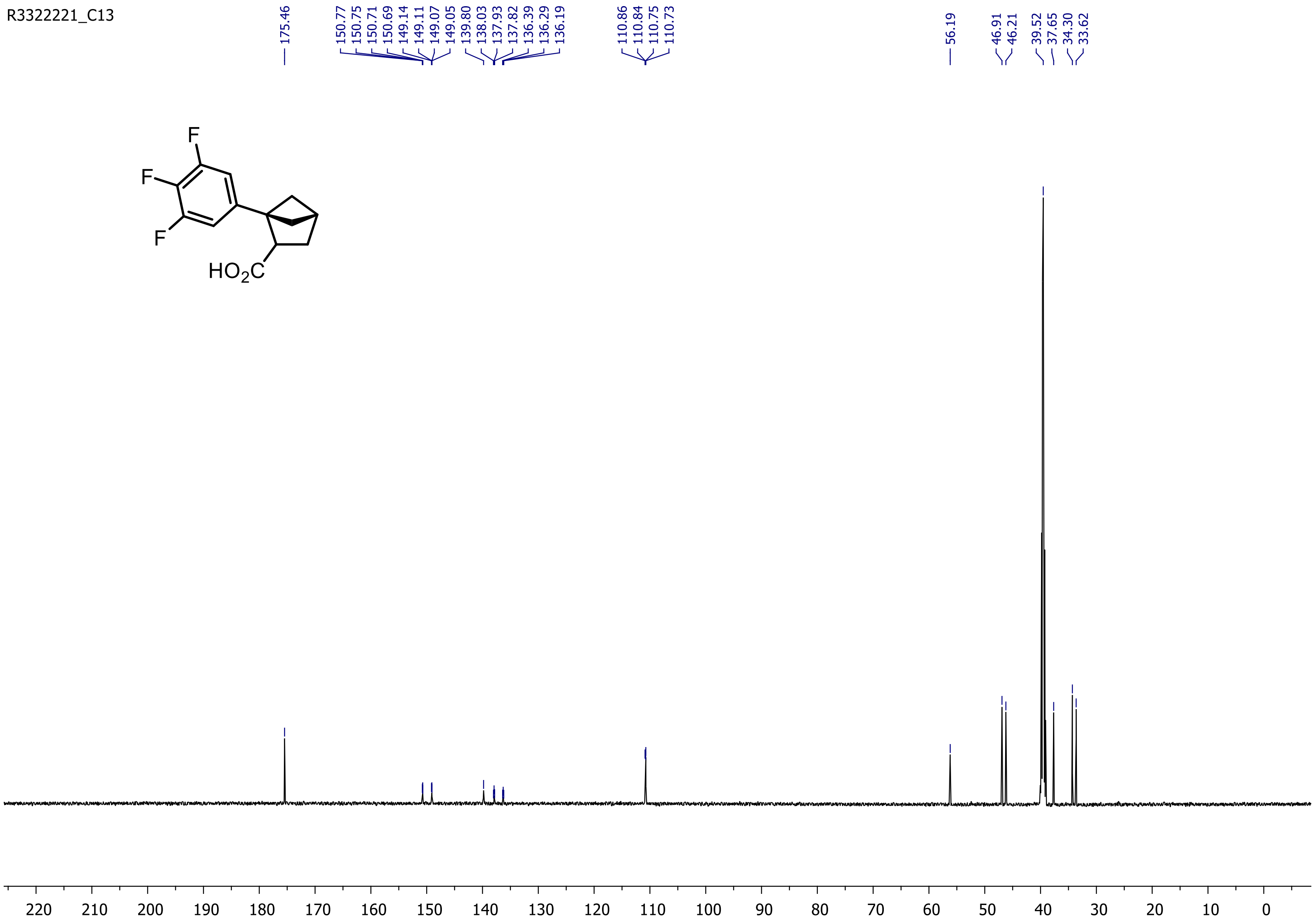

$^{19}F\{^{1}H\}$ NMR (376 MHz, DMSO-d_6)

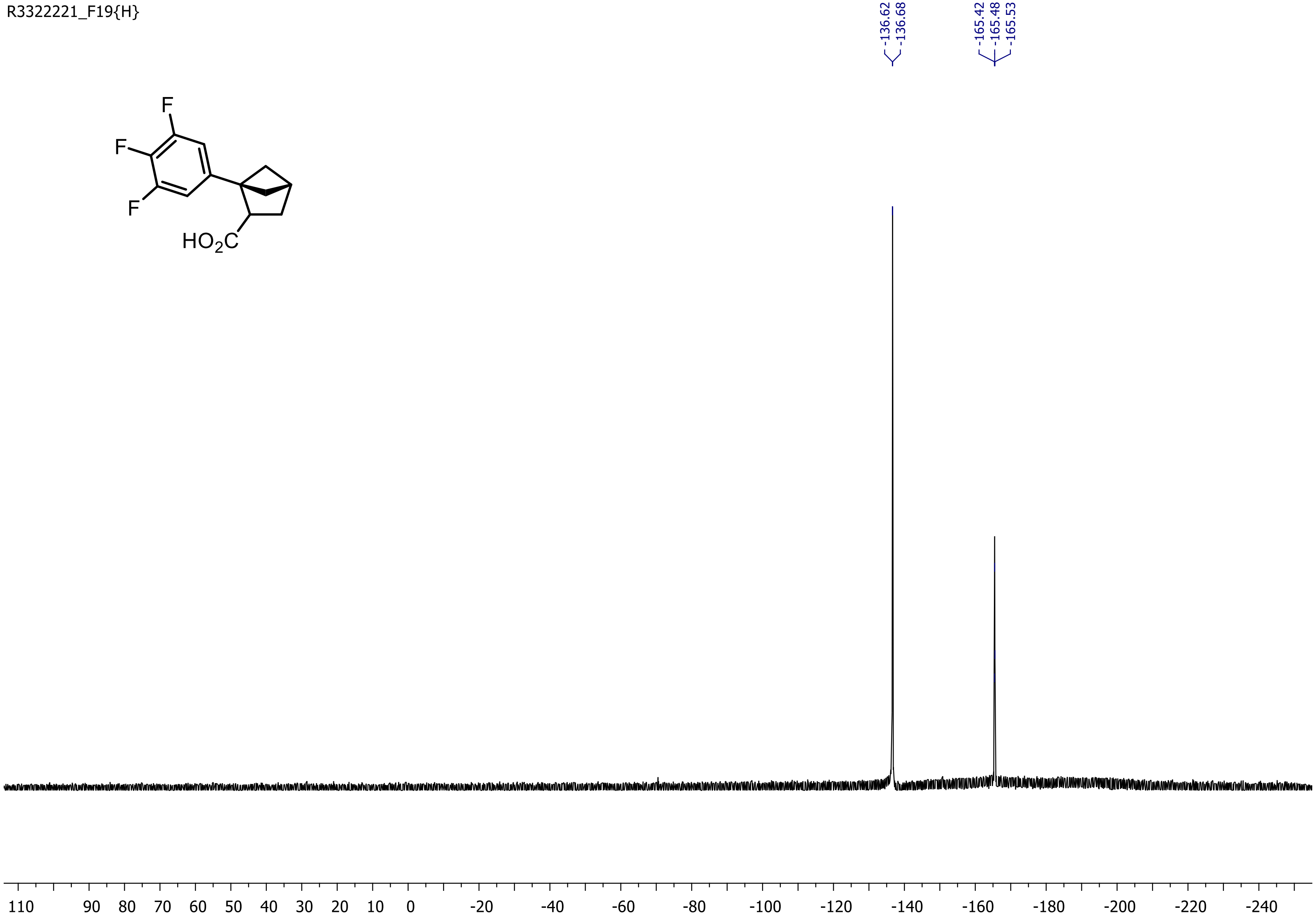

Ethyl-3-(2-fluorophenyl)but-2-enoate

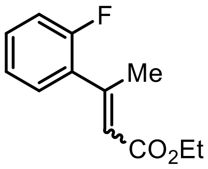

^{1}H NMR (500 MHz, $CDCl_3$)

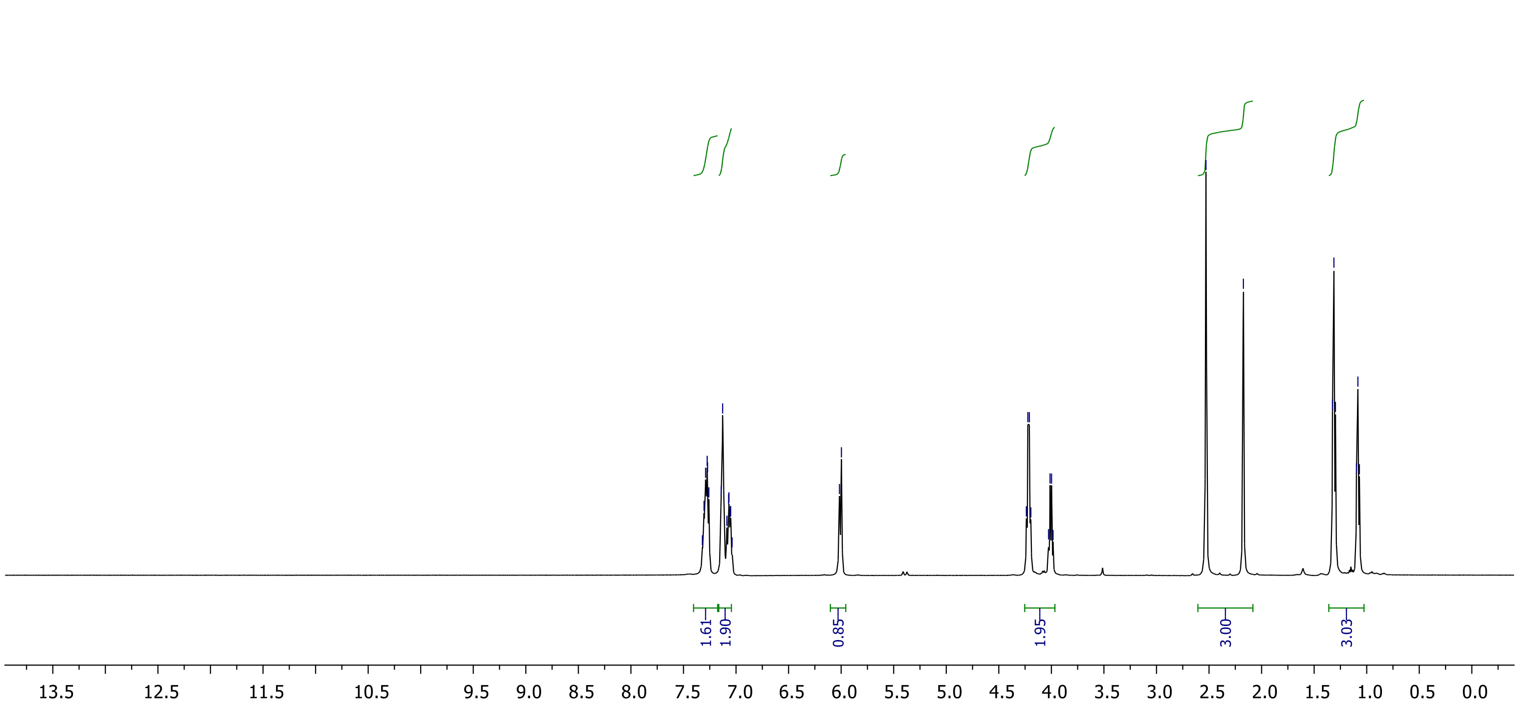

$^{13}C\{^{1}H\}$ NMR (101 MHz, $CDCl_3$)

R3390241_C13

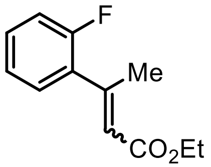

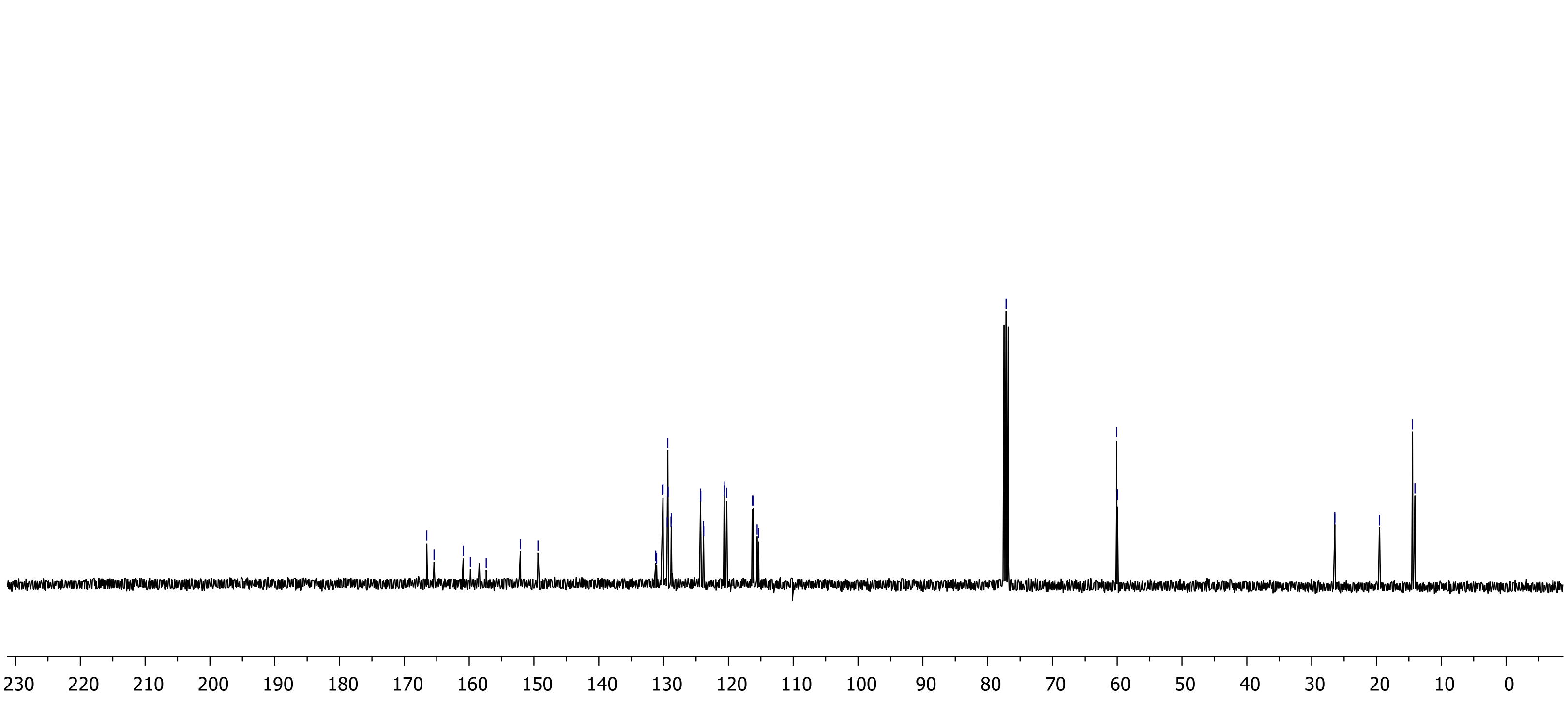

$^{19}F\{^1H\}$ NMR (376 MHz, $CDCl_3$)

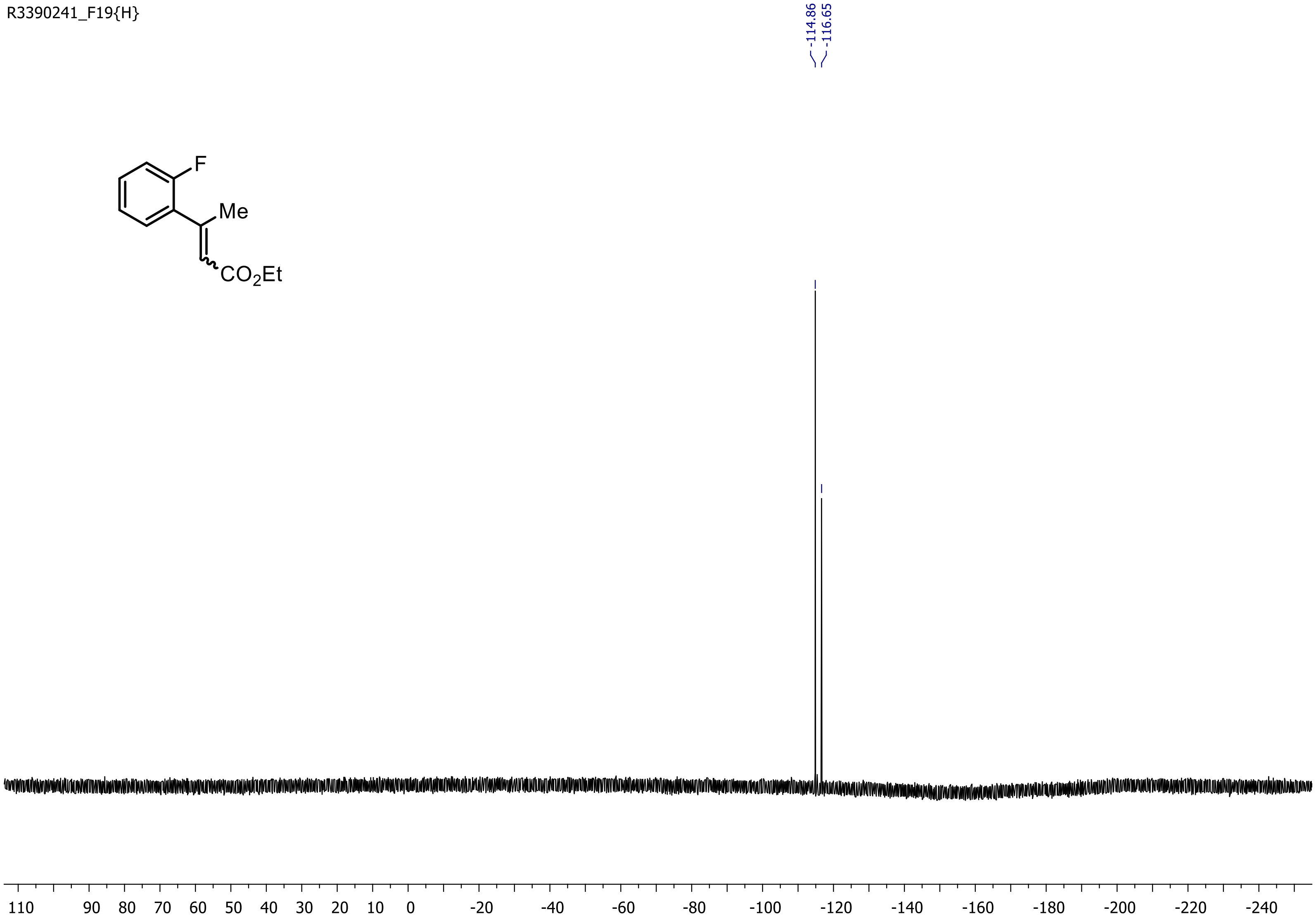

Compound 15

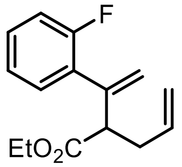

^1H NMR (500 MHz, $CDCl_3$)

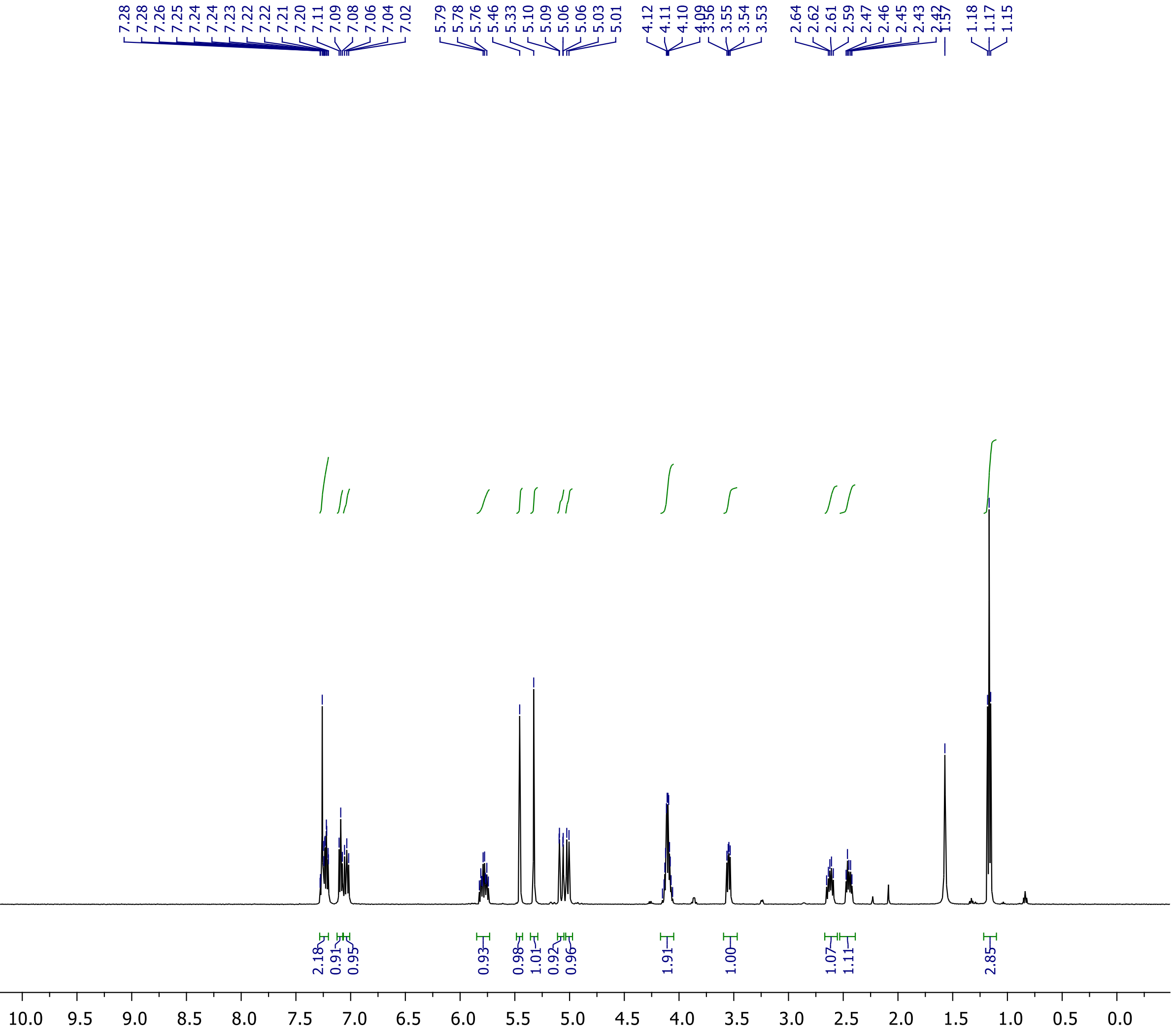

$^{13}C\{^1H\}$ NMR (151 MHz, $CDCl_3$)

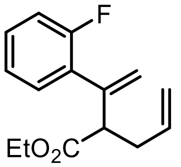

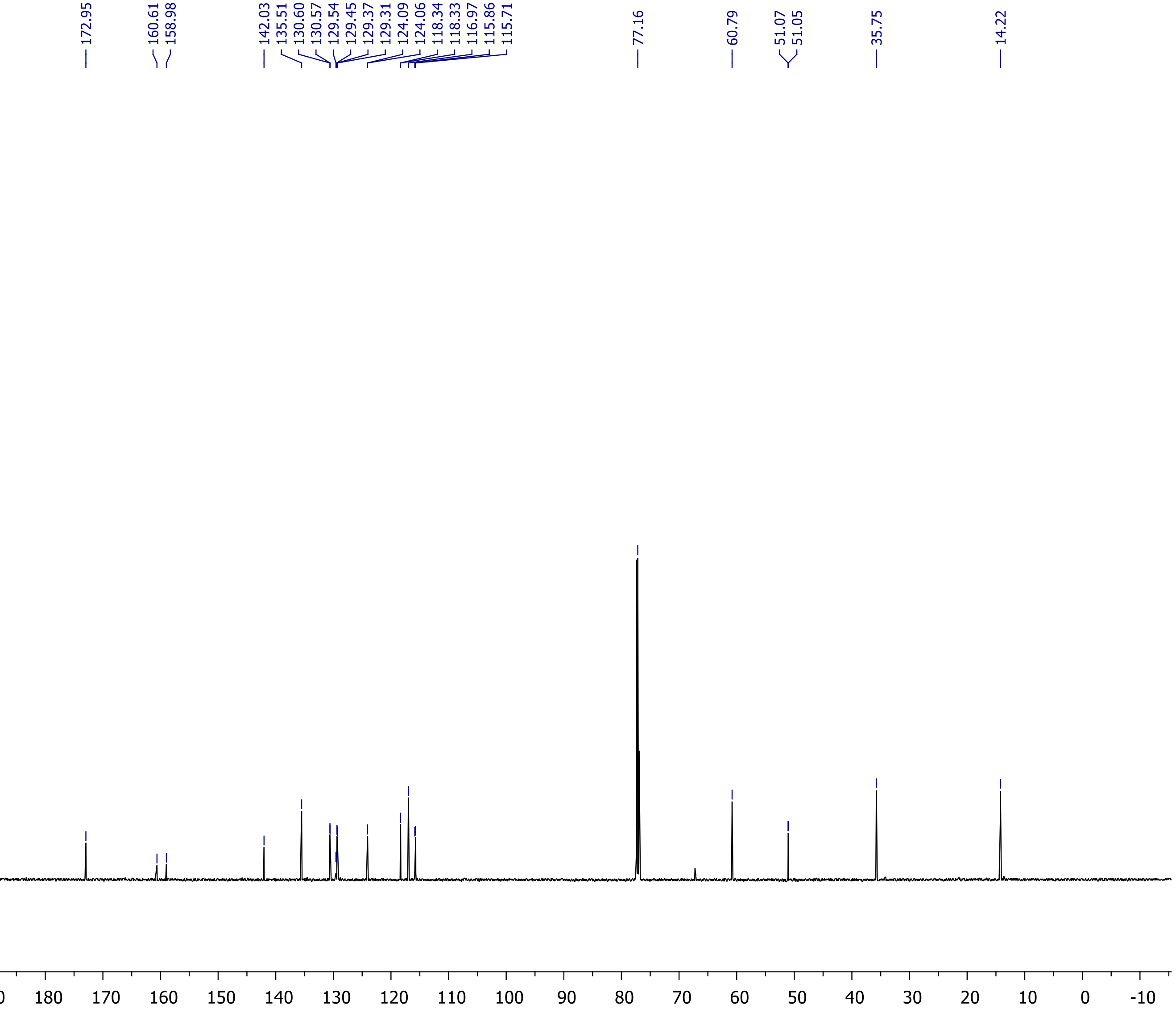

$^{19}F\{^{1}H\}$ NMR (376 MHz, $CDCl_3$)

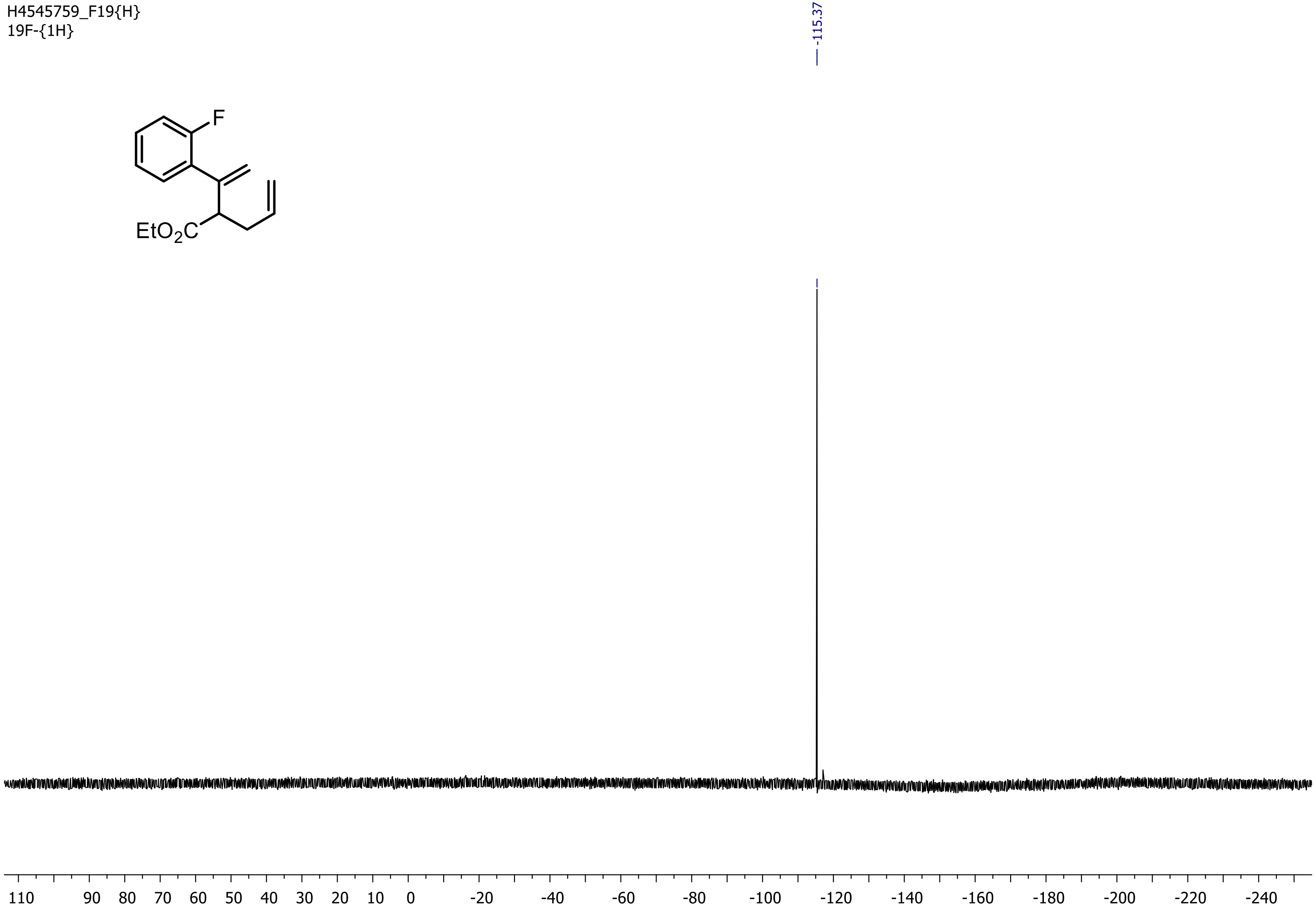

Compound (±)-15a

^{1}H NMR (500 MHz, $CDCl_3$)

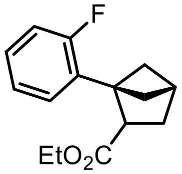

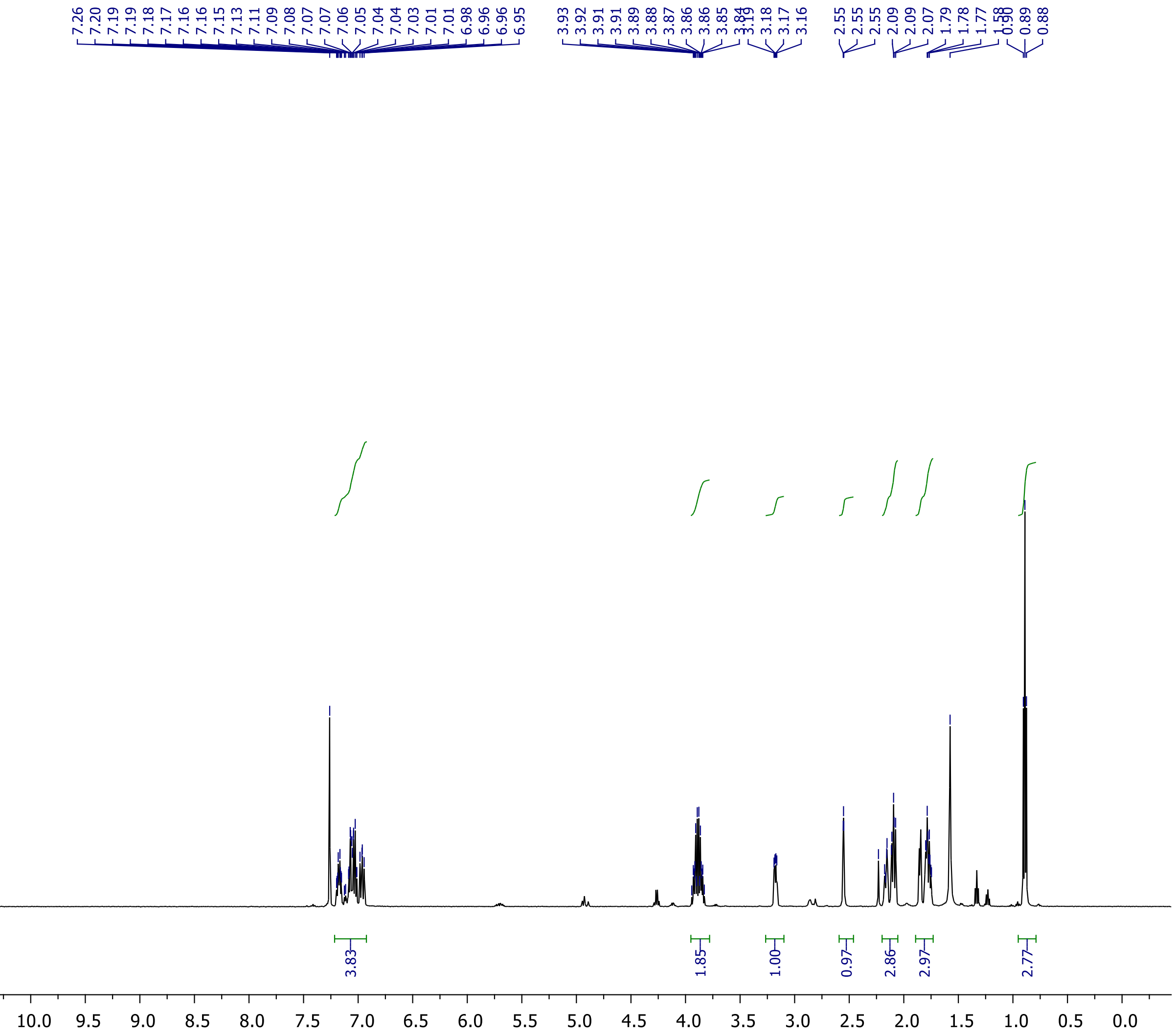

$^{13}C\{^{1}H\}$ (151 MHz, $CDCl_3$)

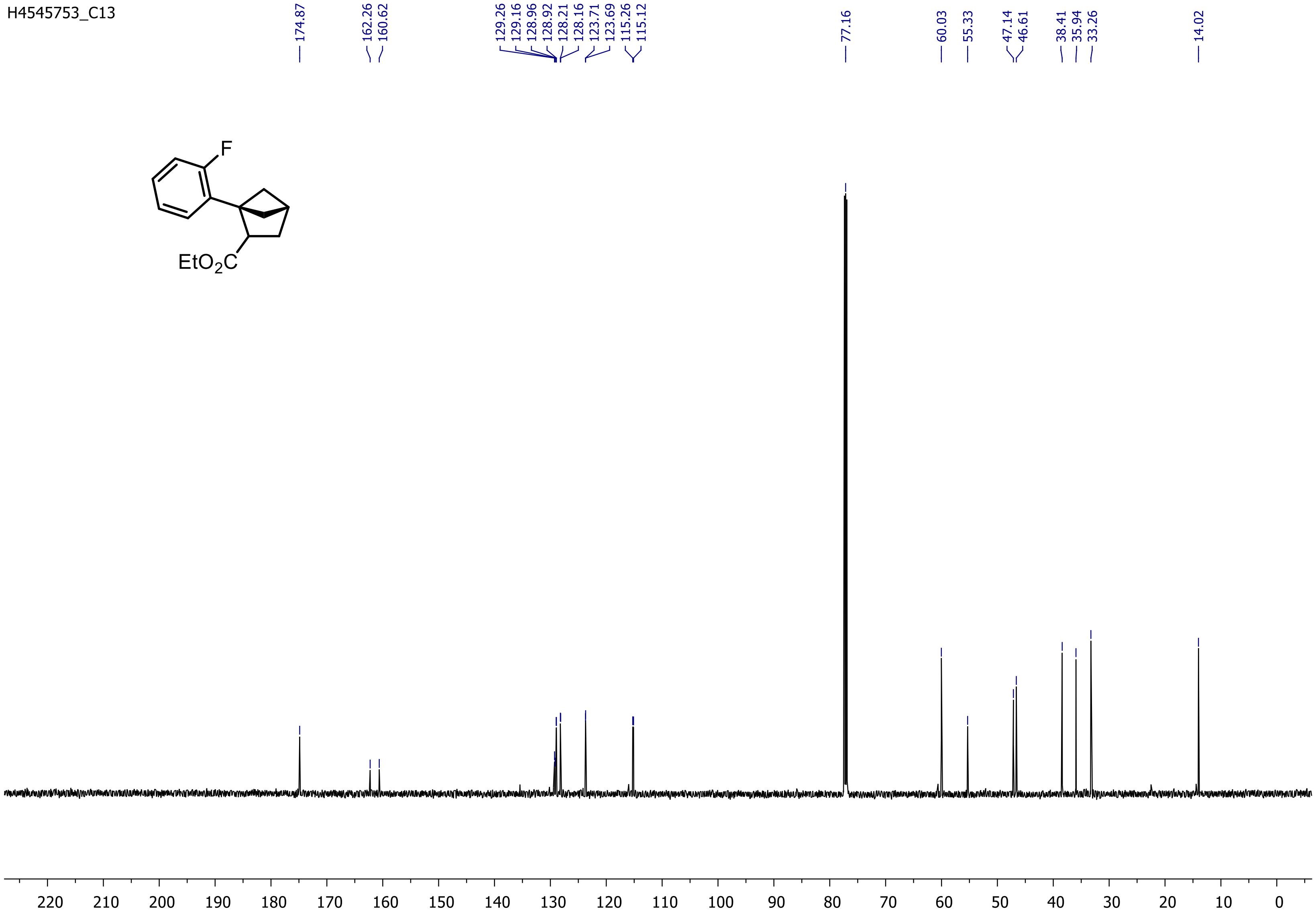

$^{19}F\{^{1}H\}$ NMR (376 MHz, $CDCl_3$)

H4545753_F19{H}
19F-{1H}

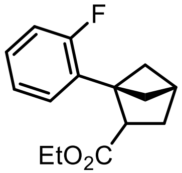

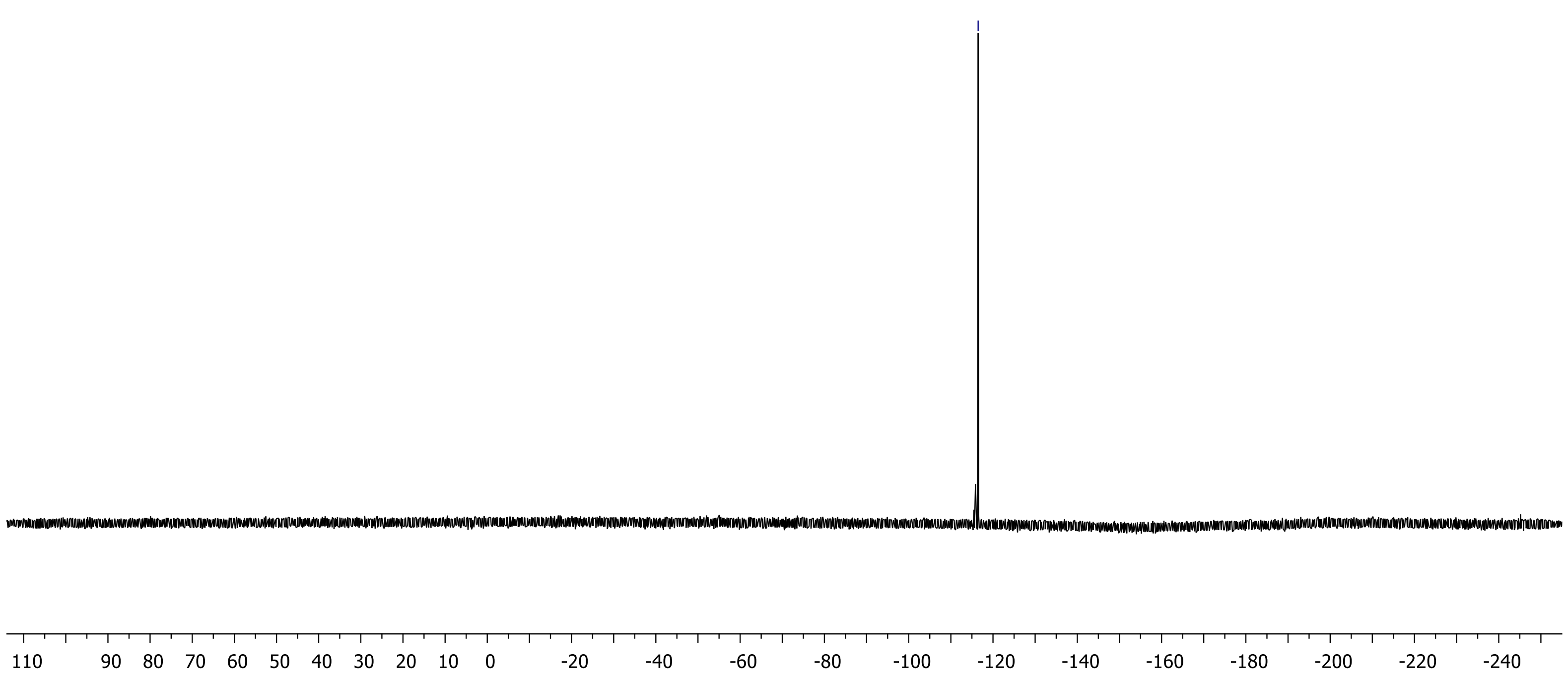

Compound (±)-15b

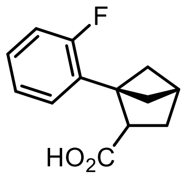

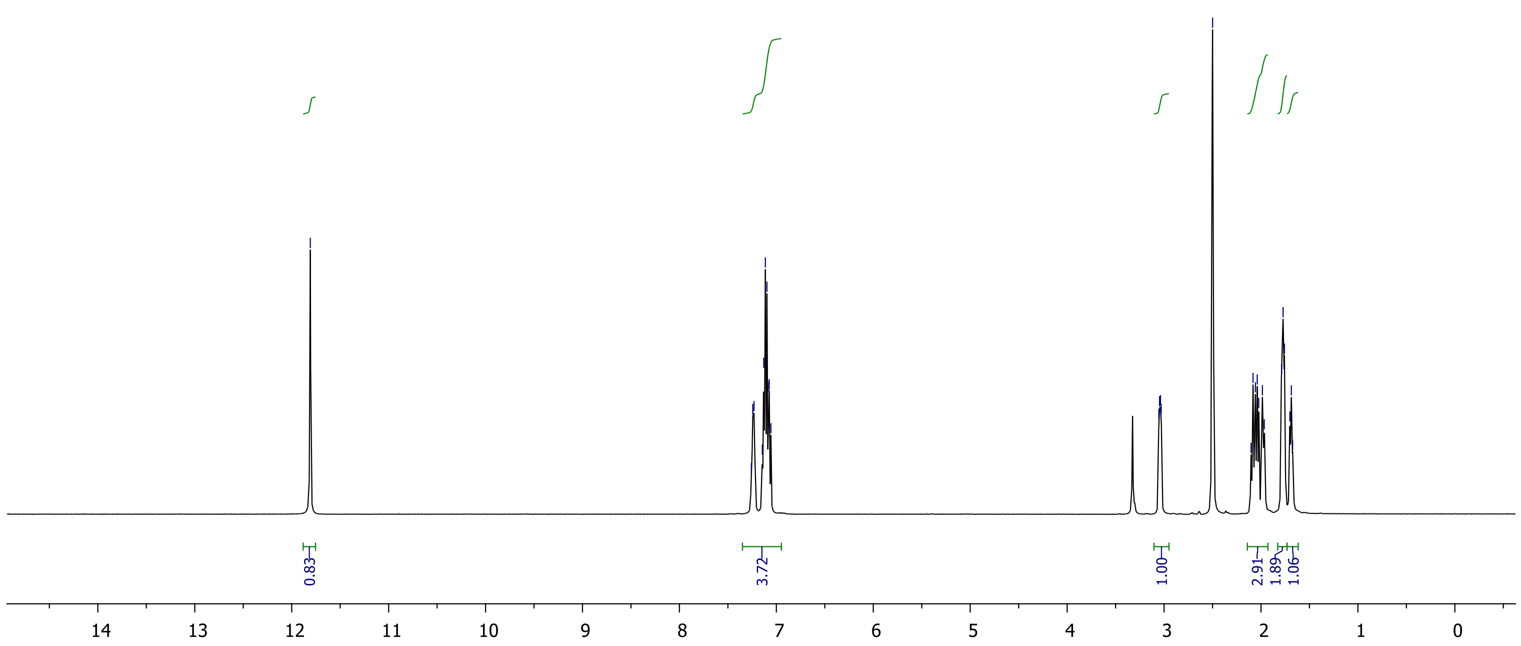

$^{13}C\{^{1}H\}$ NMR (151 MHz, DMSO-d$_6$)

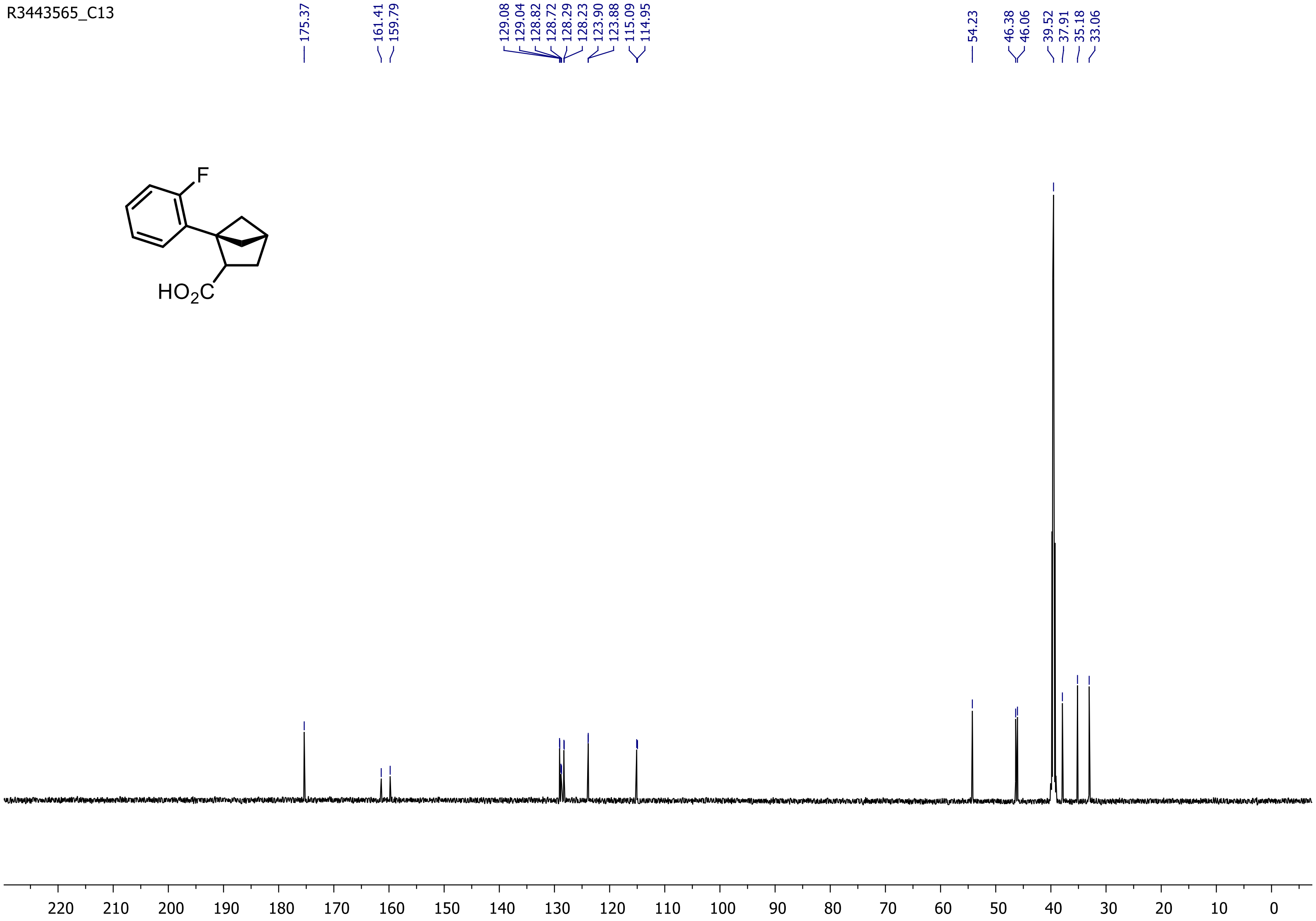

$^{19}F\{^{1}H\}$ NMR (376 MHz, DMSO-d$_6$)

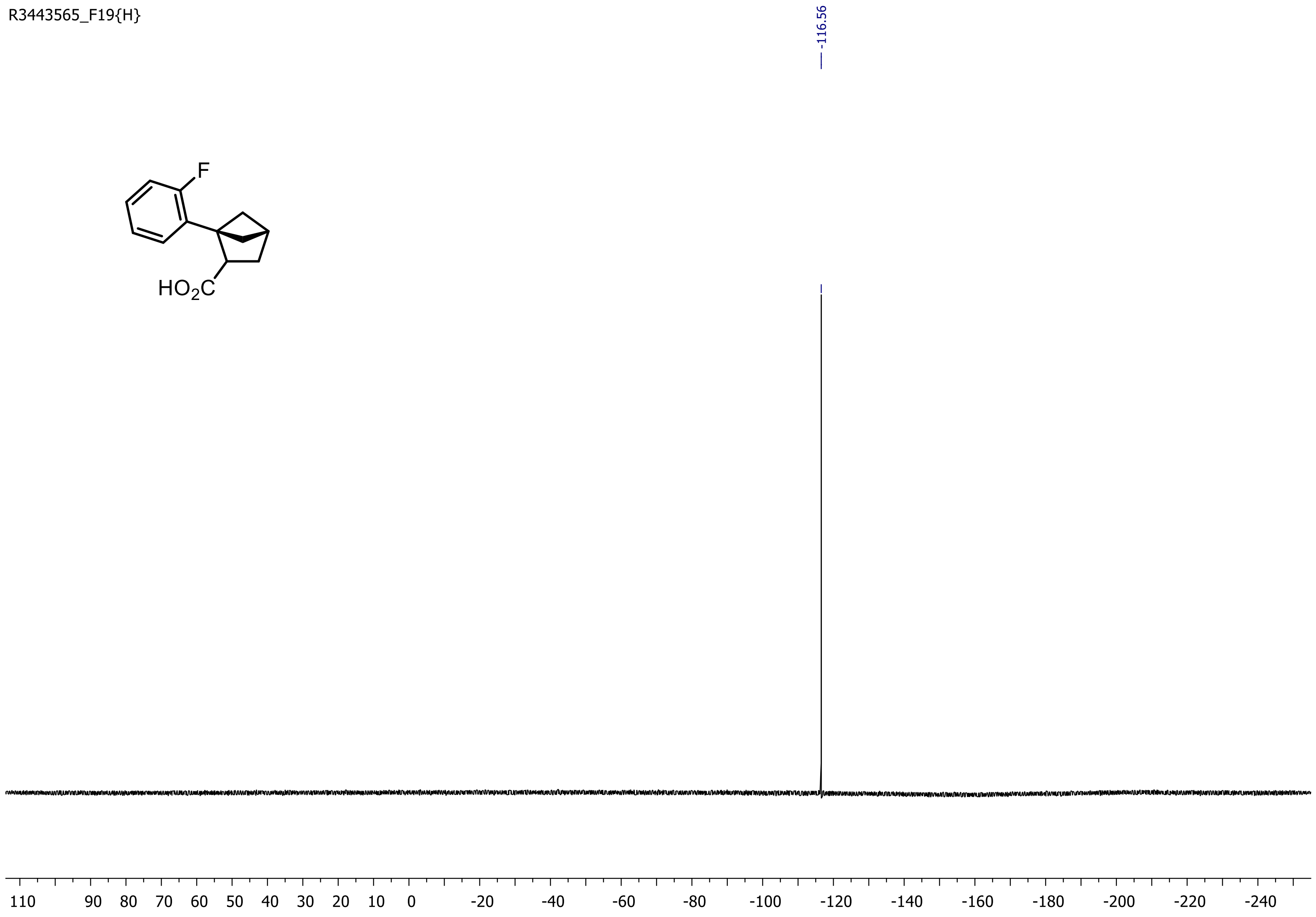

Ethyl-3-(1-methyl-1*H*-pyrazol-4-yl)but-2-enoate

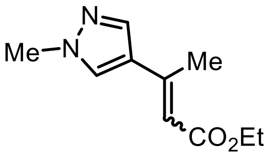

^1H NMR (500 MHz, $CDCl_3$)

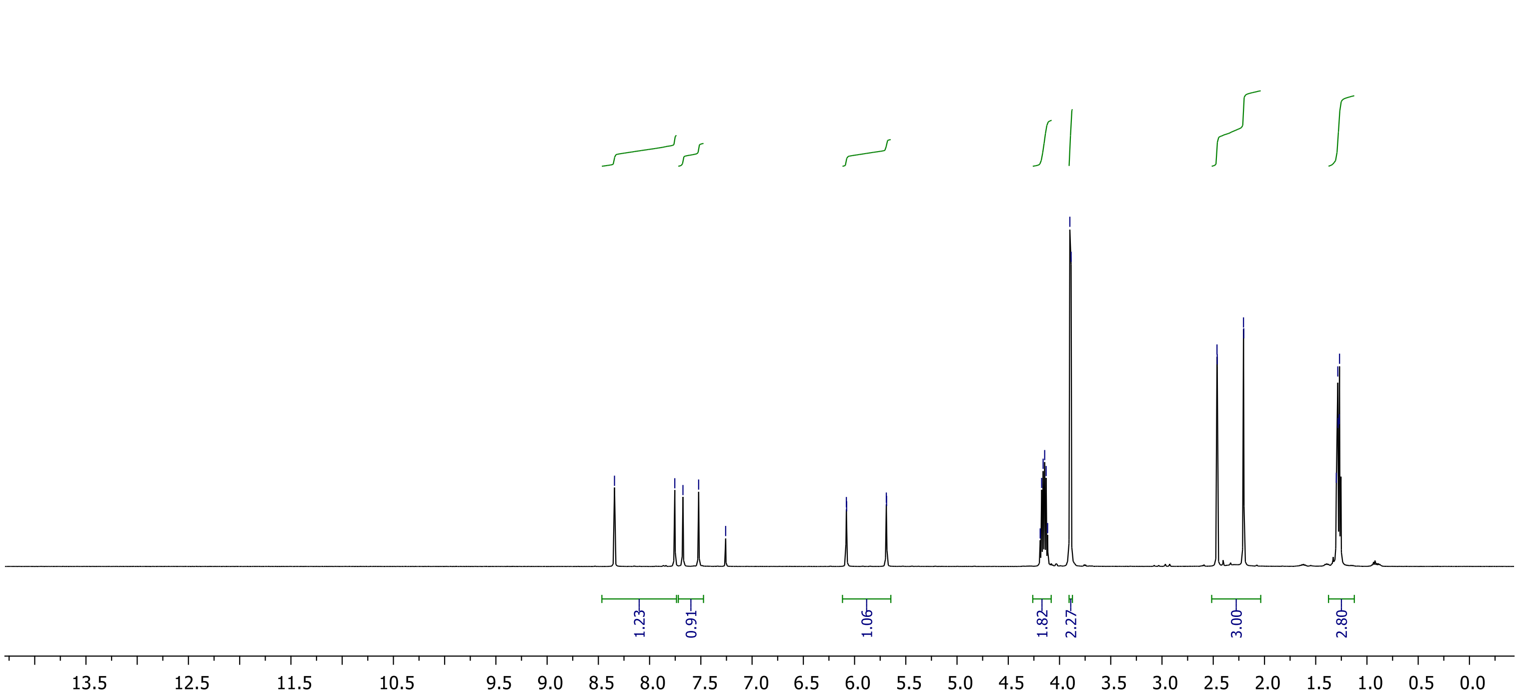

$^{13}C\{^1H\}$ NMR (126 MHz, $CDCl_3$)

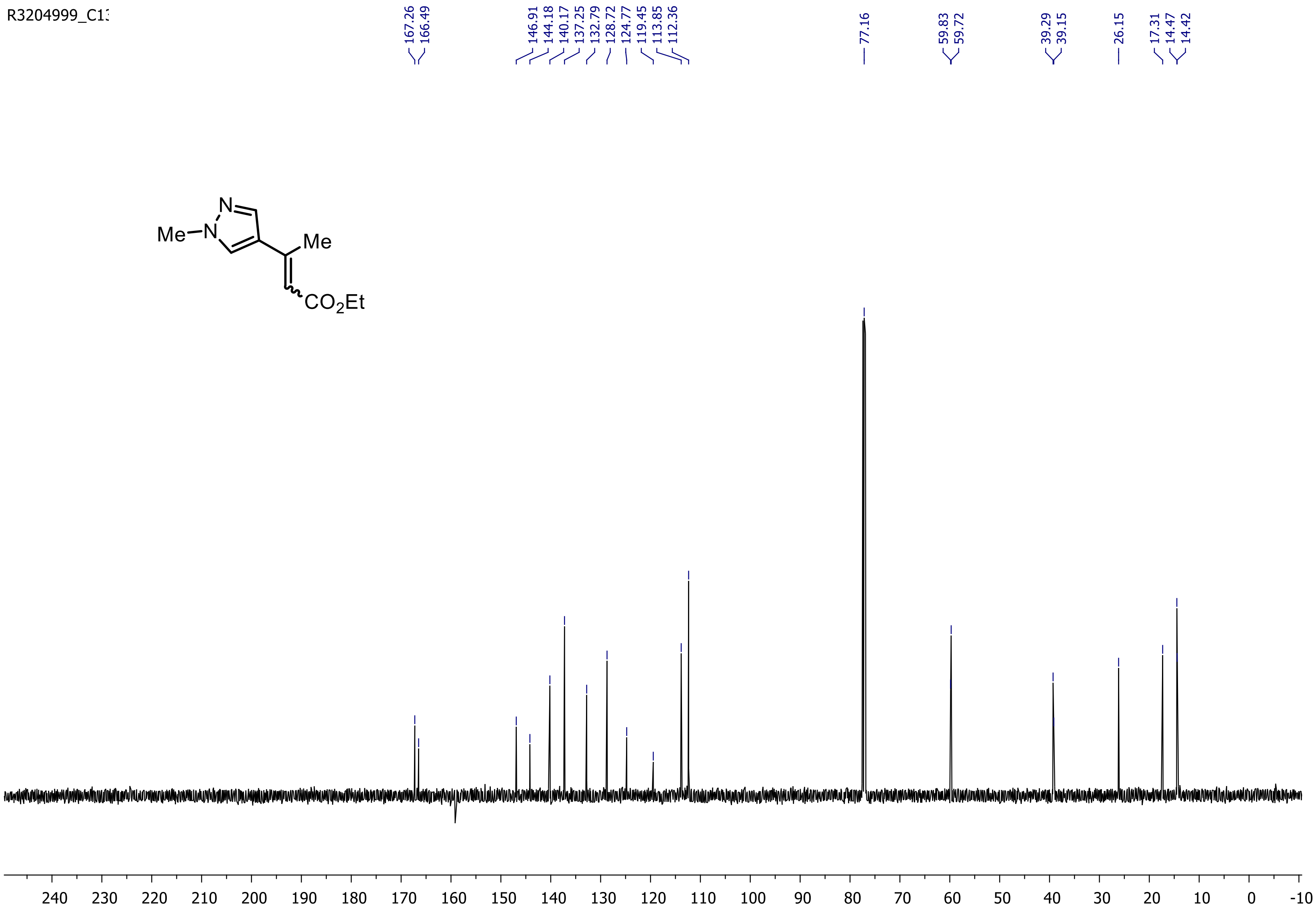

Compound 16

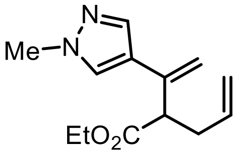

Me
N
N
EtO2C

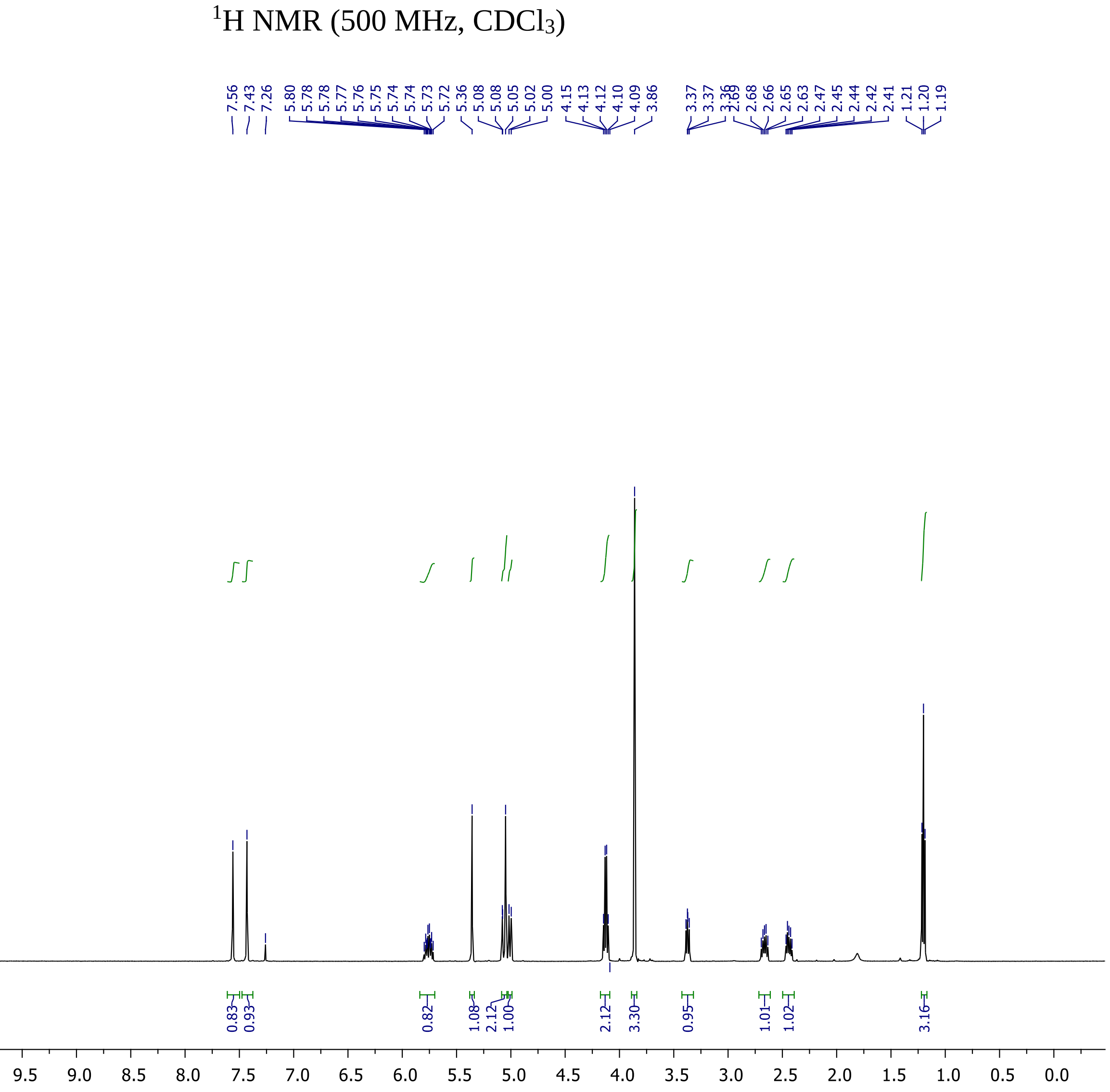

^{1}H NMR (500 MHz, CDCl3)
H4542732
7.56
7.43
7.26
5.80
5.78
5.78
5.77
5.76
5.75
5.74
5.74
5.73
5.72
5.36
5.08
5.08
5.05
5.02
5.00
4.15
4.13
4.12
4.10
4.09
3.86
3.37
3.37
3.36
2.69
2.68
2.66
2.65
2.63
2.47
2.45
2.44
2.42
2.41
1.21
1.20
1.19
0.83
0.93
0.82
1.08
2.12
1.00
2.12
3.30
0.95
1.01
1.02
3.16
12.5
12.0
11.5
11.0
10.5
10.0
9.5
9.0
8.5
8.0
7.5
7.0
6.5
6.0
5.5
5.0
4.5
4.0
3.5
3.0
2.5
2.0
1.5
1.0
0.5
0.0

$^{13}C\{^{1}H\}$ NMR (126 MHz, $CDCl_3$)

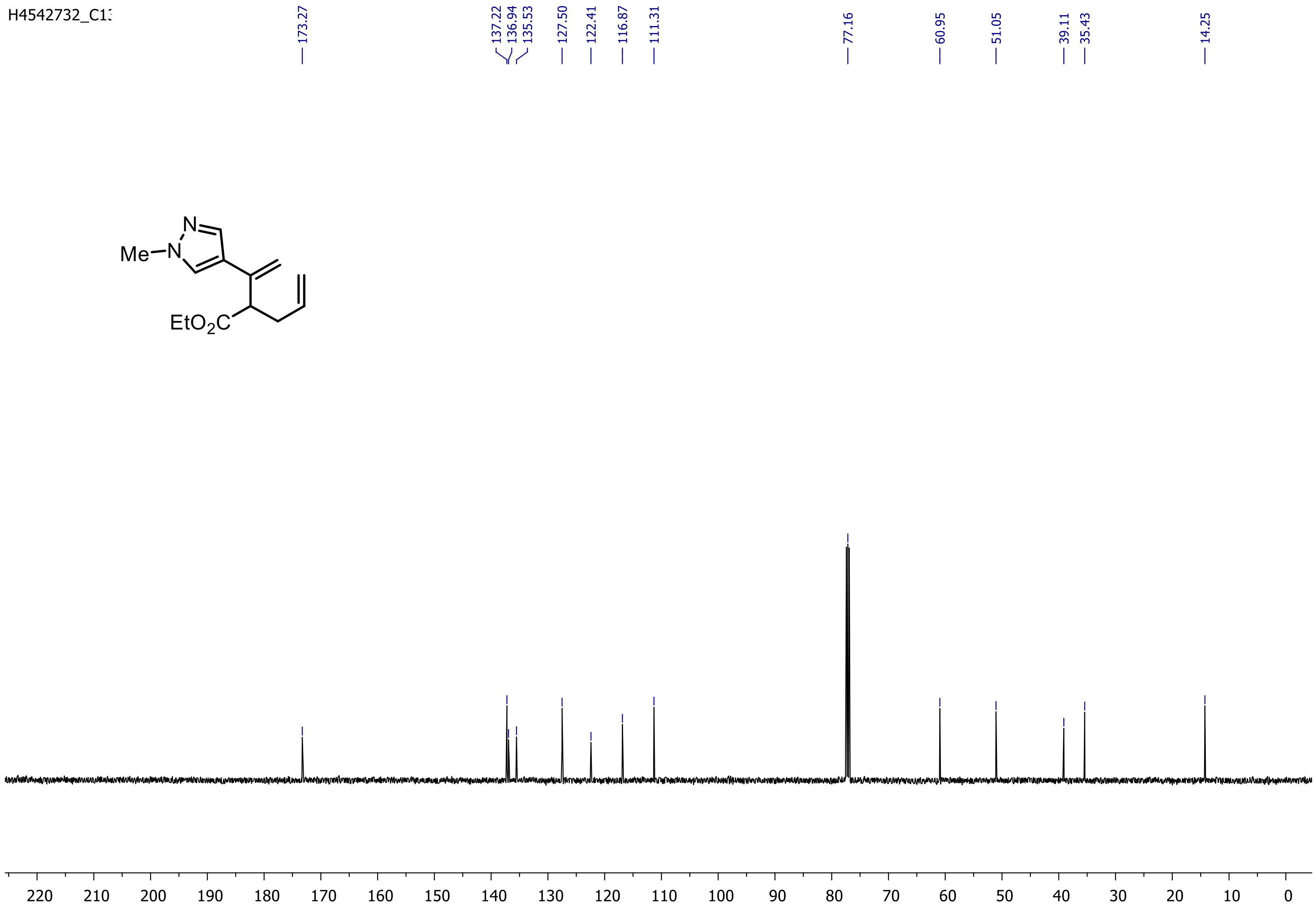

Compound (±)-16a (the sample contains ca. 10% of the isomeric 1,5-disubstituted bicyclo[2.1.1]hexane)

^{1}H NMR (500 MHz, $CDCl_3$)

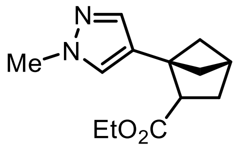

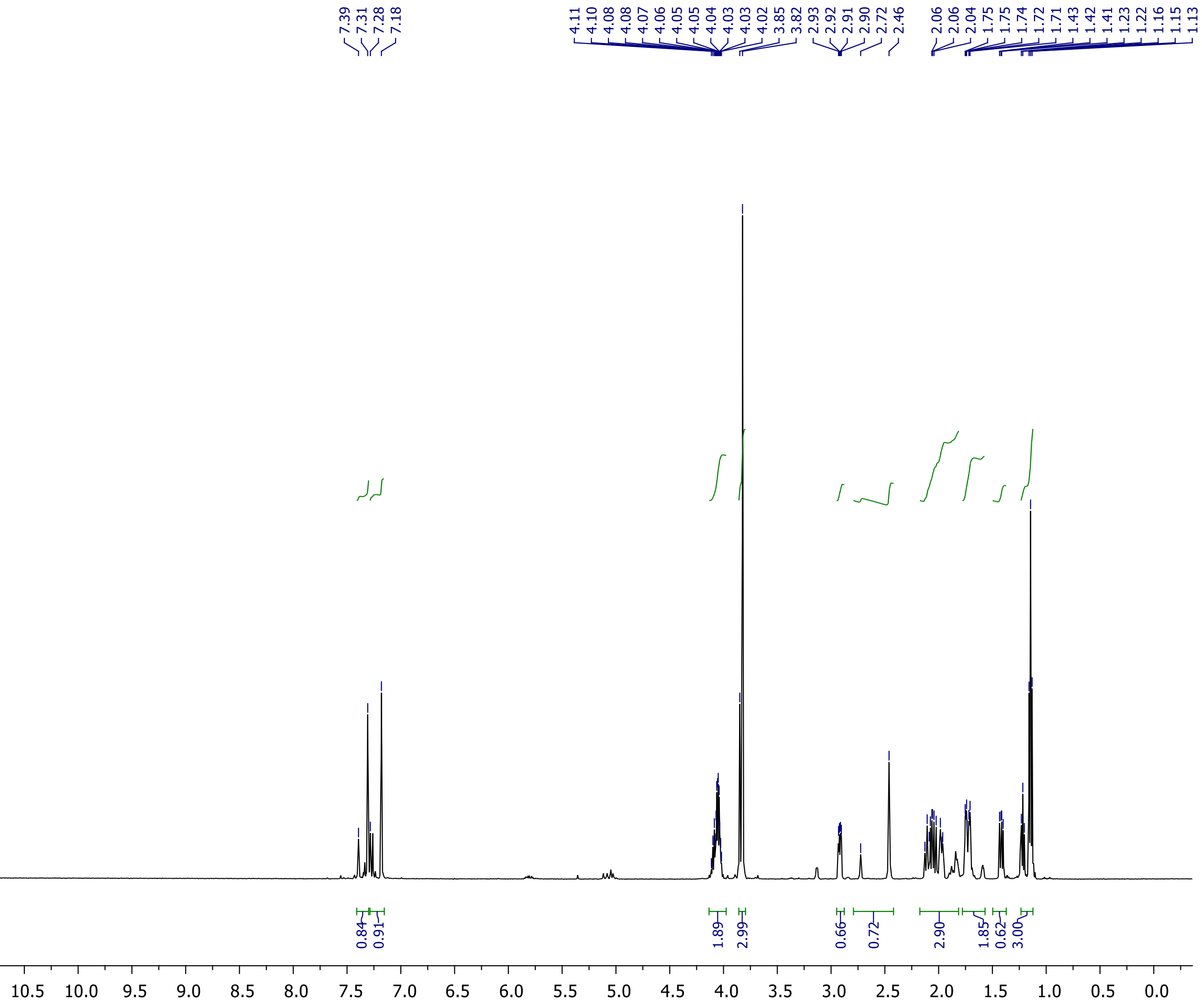

$^{13}C\{^{1}H\}$ NMR (126 MHz, $CDCl_3$)

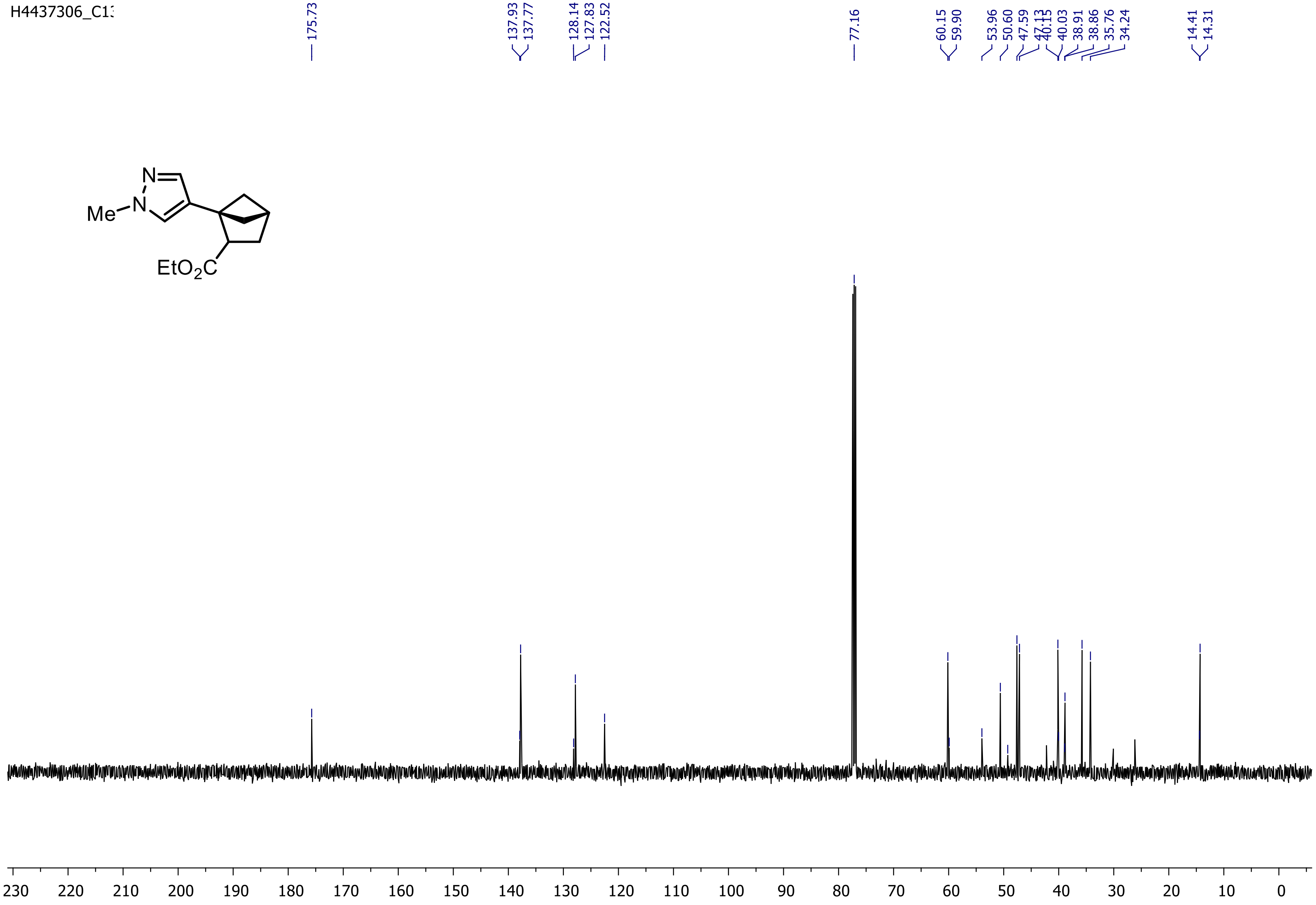

Compound (±)-16b

^{1}H NMR (500 MHz, DMSO-d$_6$)

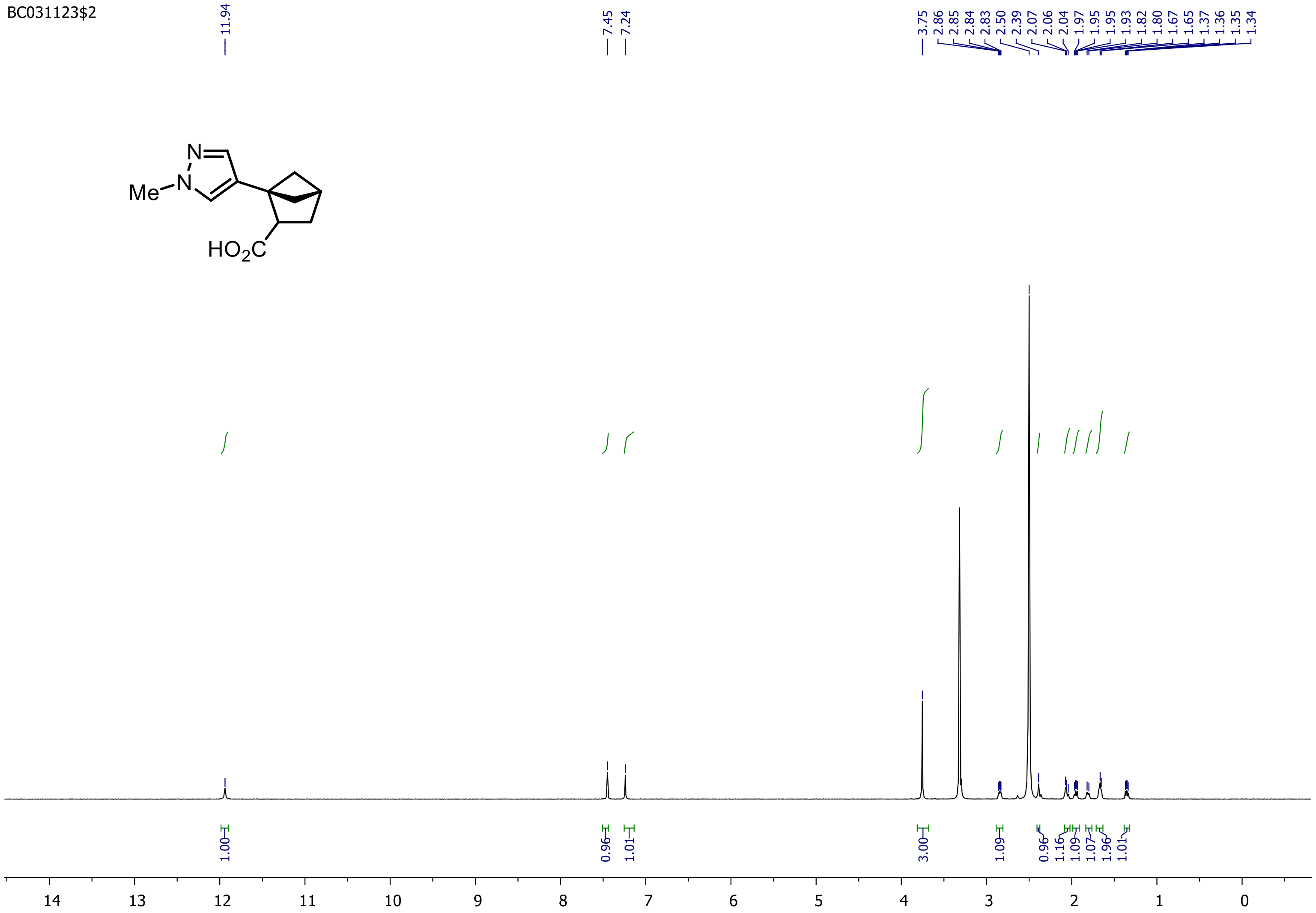

$^{13}C\{^{1}H\}$ NMR (126 MHz, DMSO-d_6)

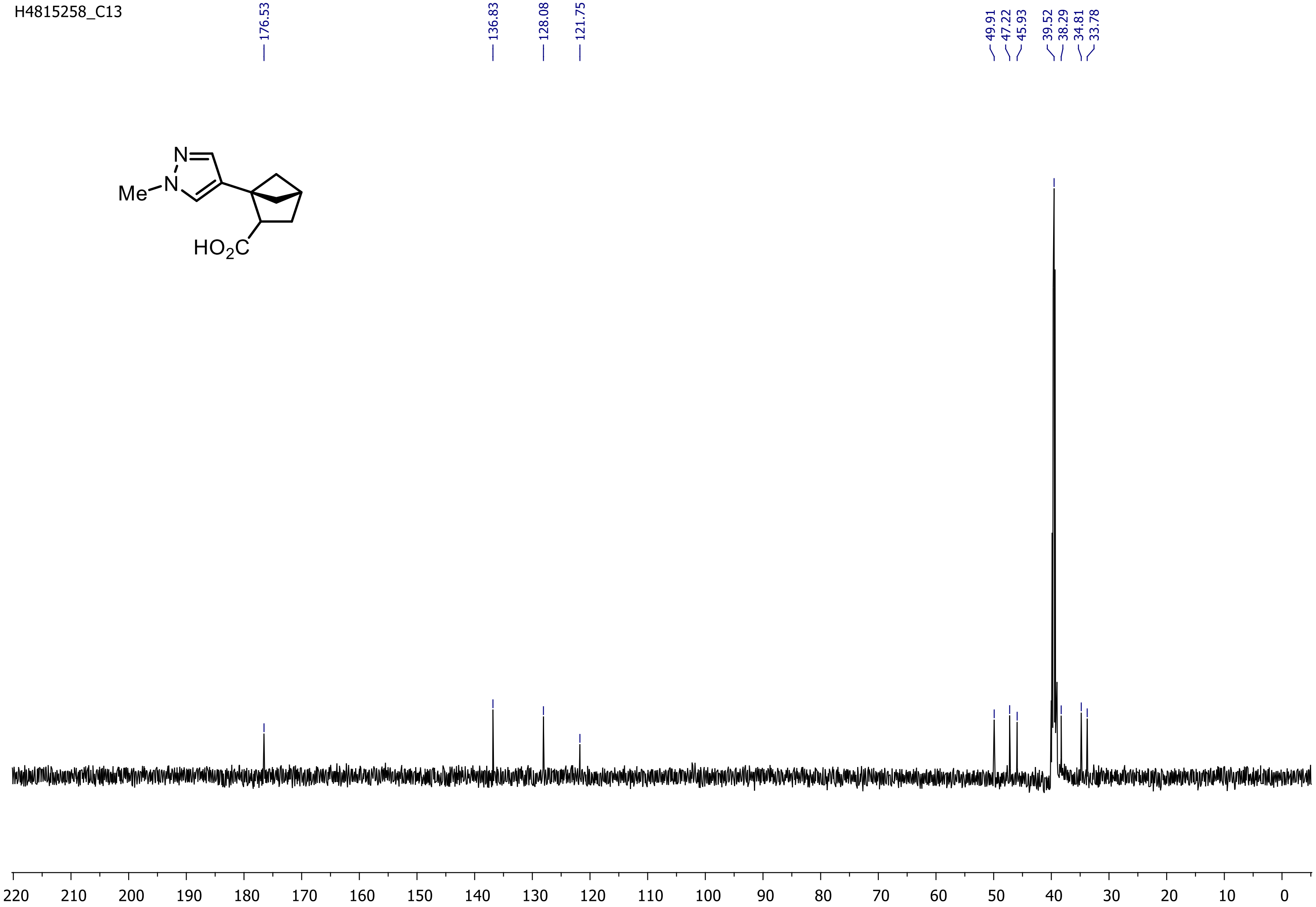

Ethyl-3-(1-methyl-1*H*-pyrazol-5-yl)but-2-enoate

^{1}H NMR (500 MHz, $CDCl_3$)

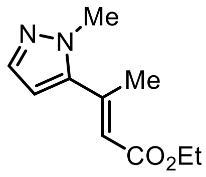

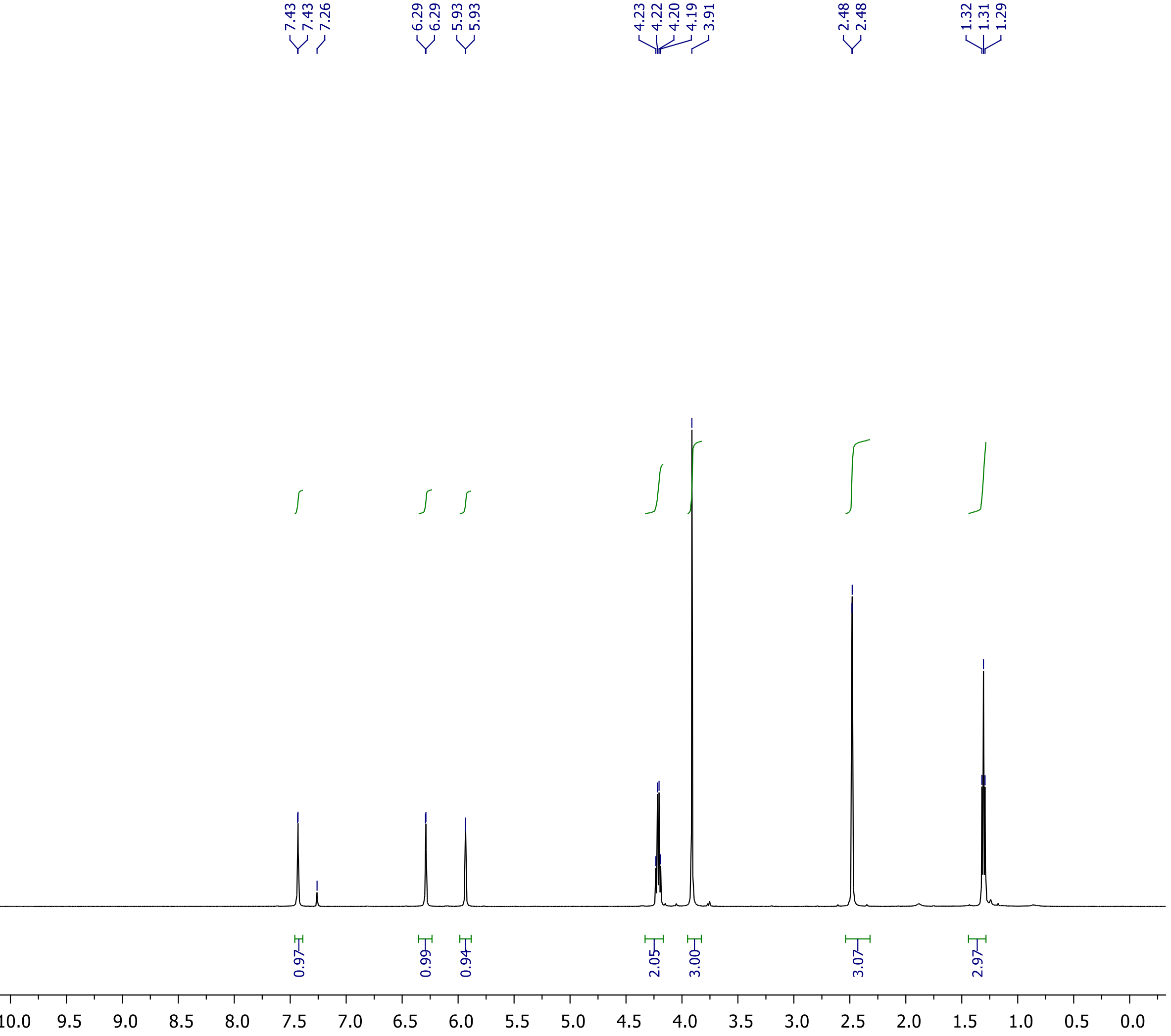

$^{13}C\{^{1}H\}$ NMR (151 MHz, $CDCl_3$)

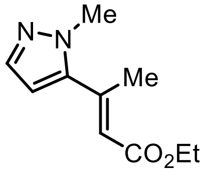

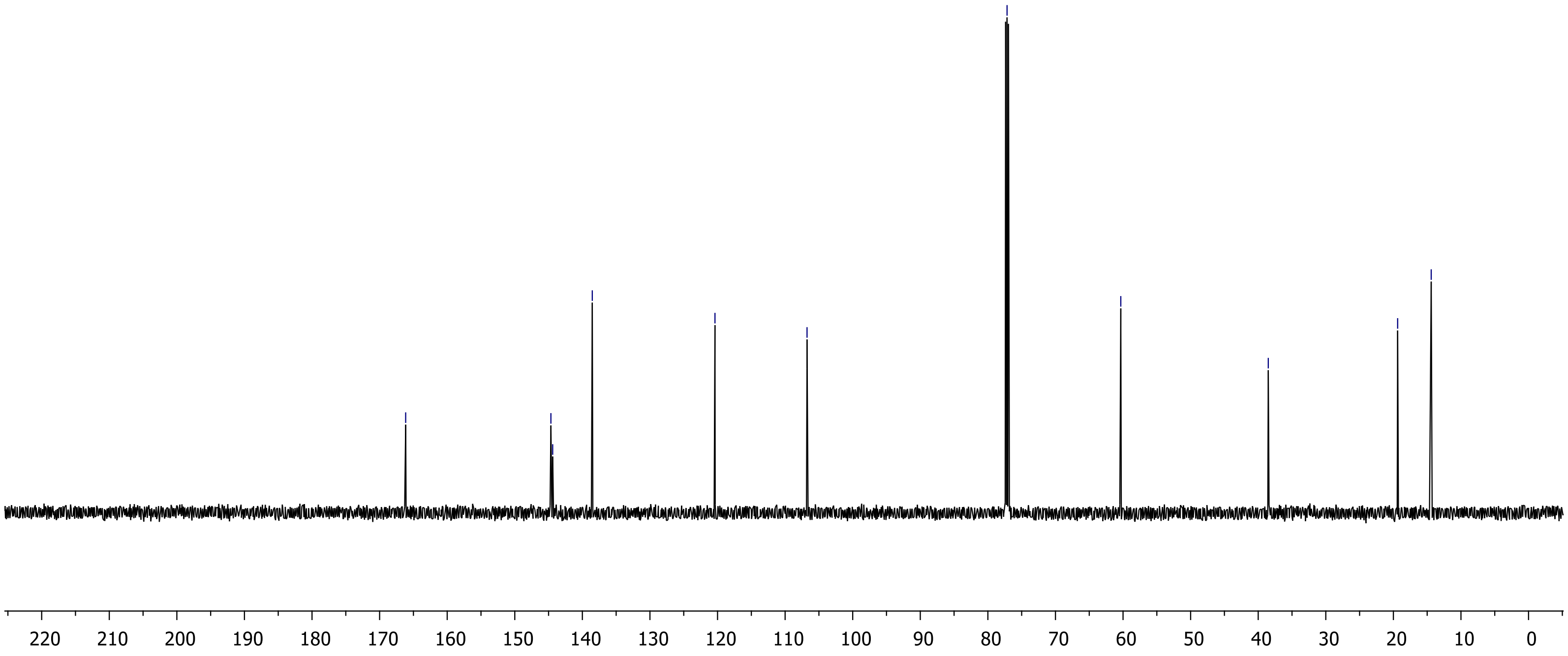

Compound 17

^{1}H NMR (500 MHz, $CDCl_3$)

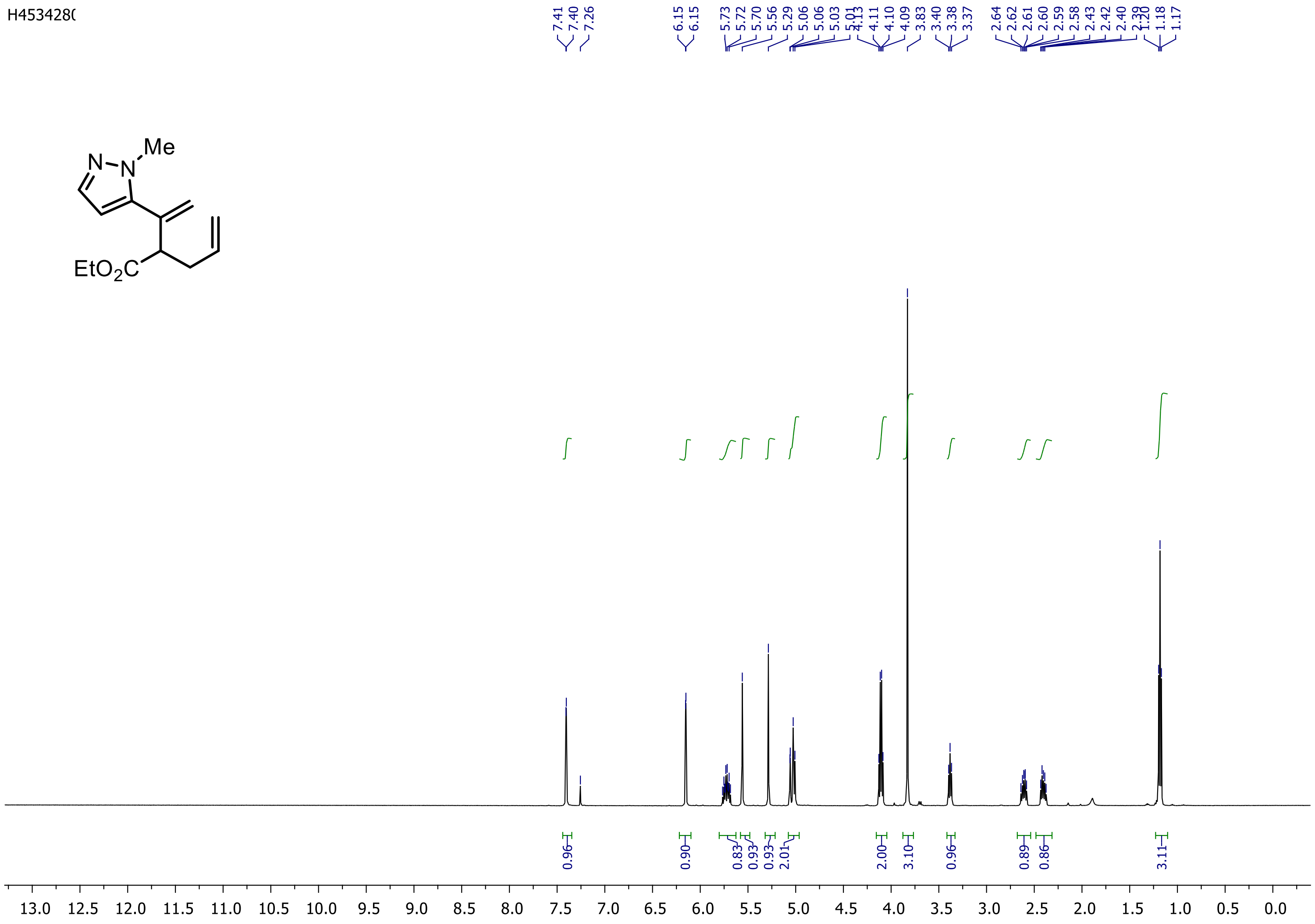

$^{13}C\{^{1}H\}$ NMR (126 MHz, $CDCl_3$)

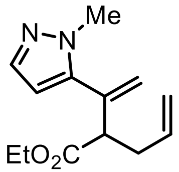

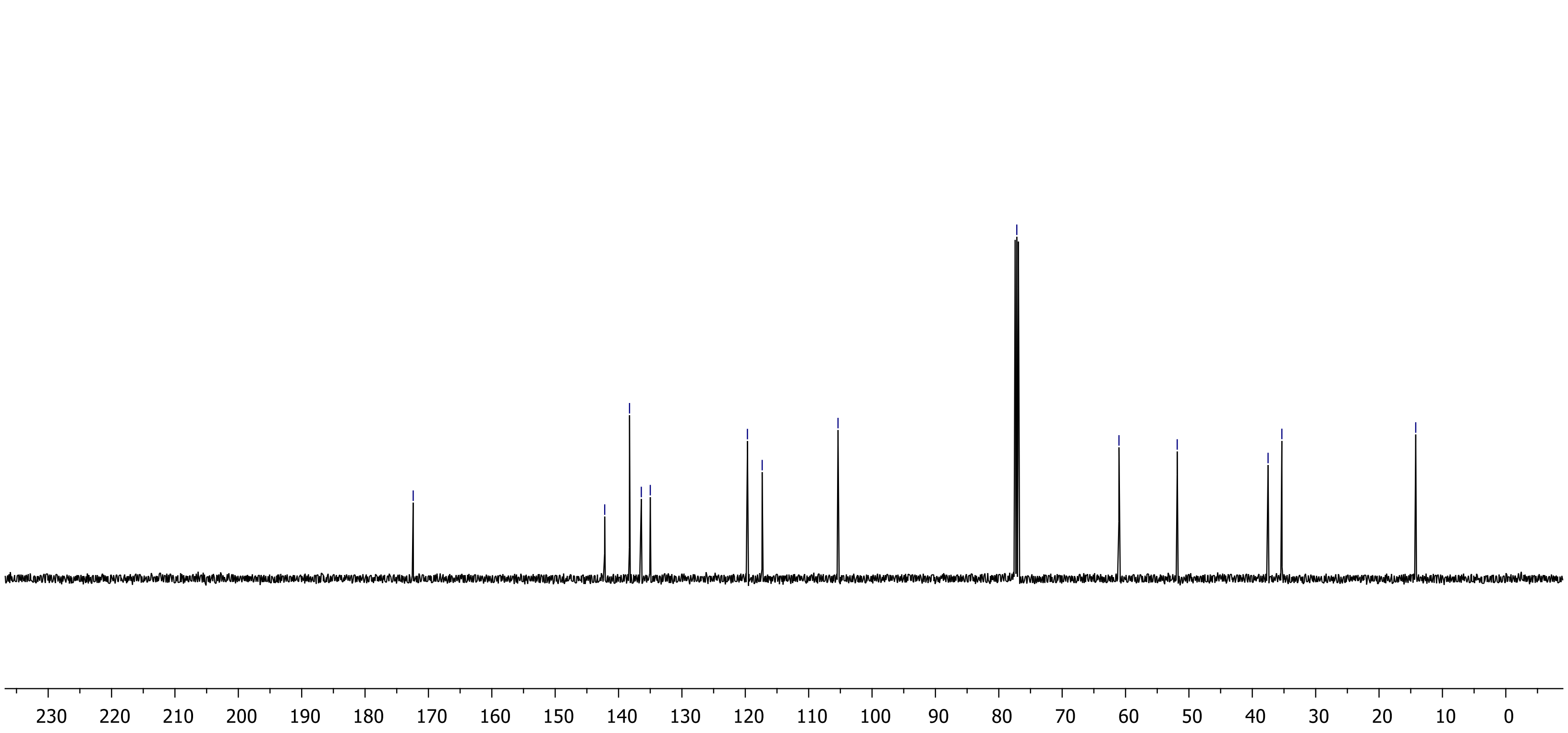

Compound (±)-17a (the sample contains ca. 20% of the isomeric 1,5-disubstituted bicyclo[2.1.1]hexane)

^{1}H NMR (500 MHz, DMSO-d_6)

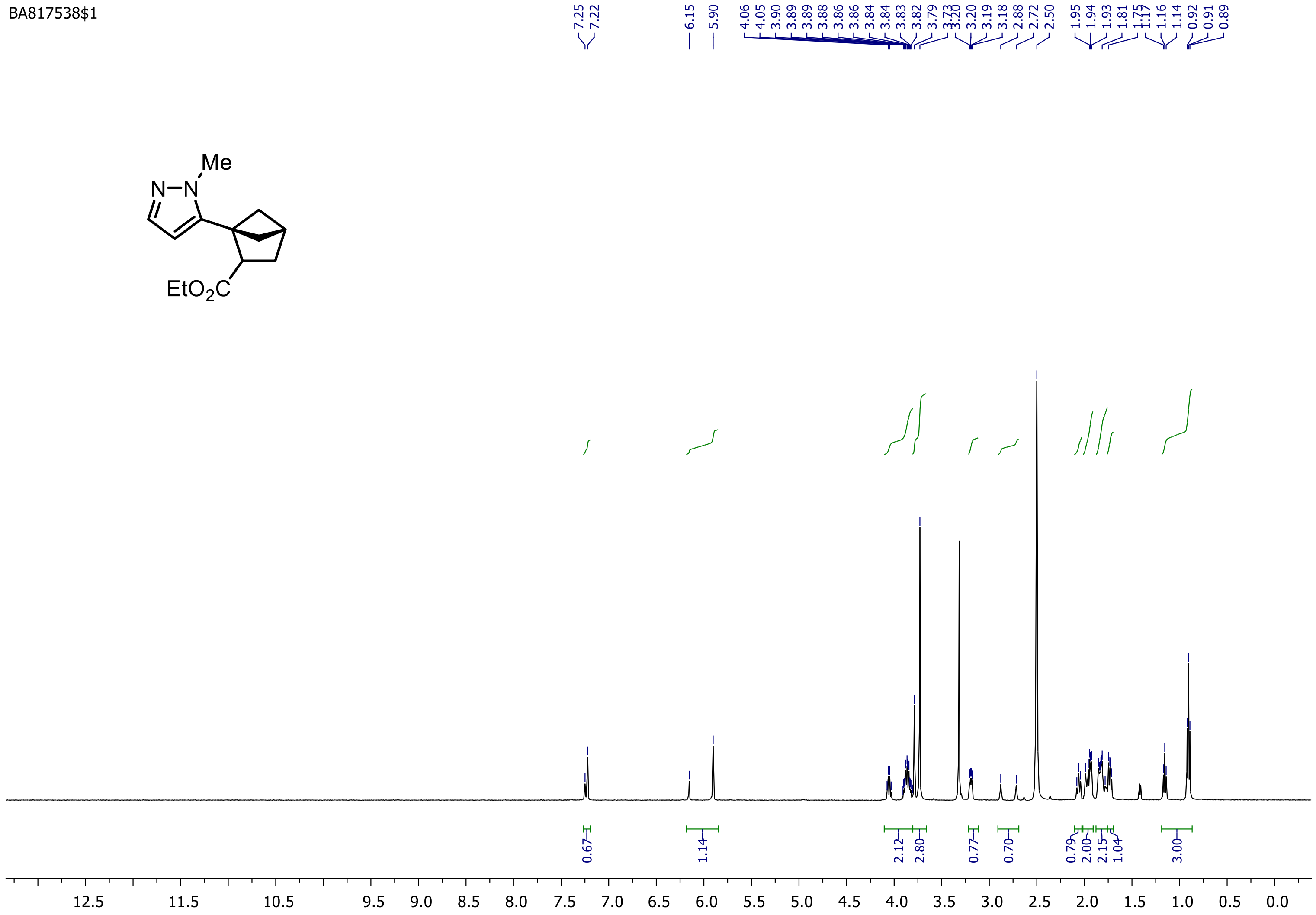

$^{13}C\{^{1}H\}$ NMR (151 MHz, $CDCl_3$)

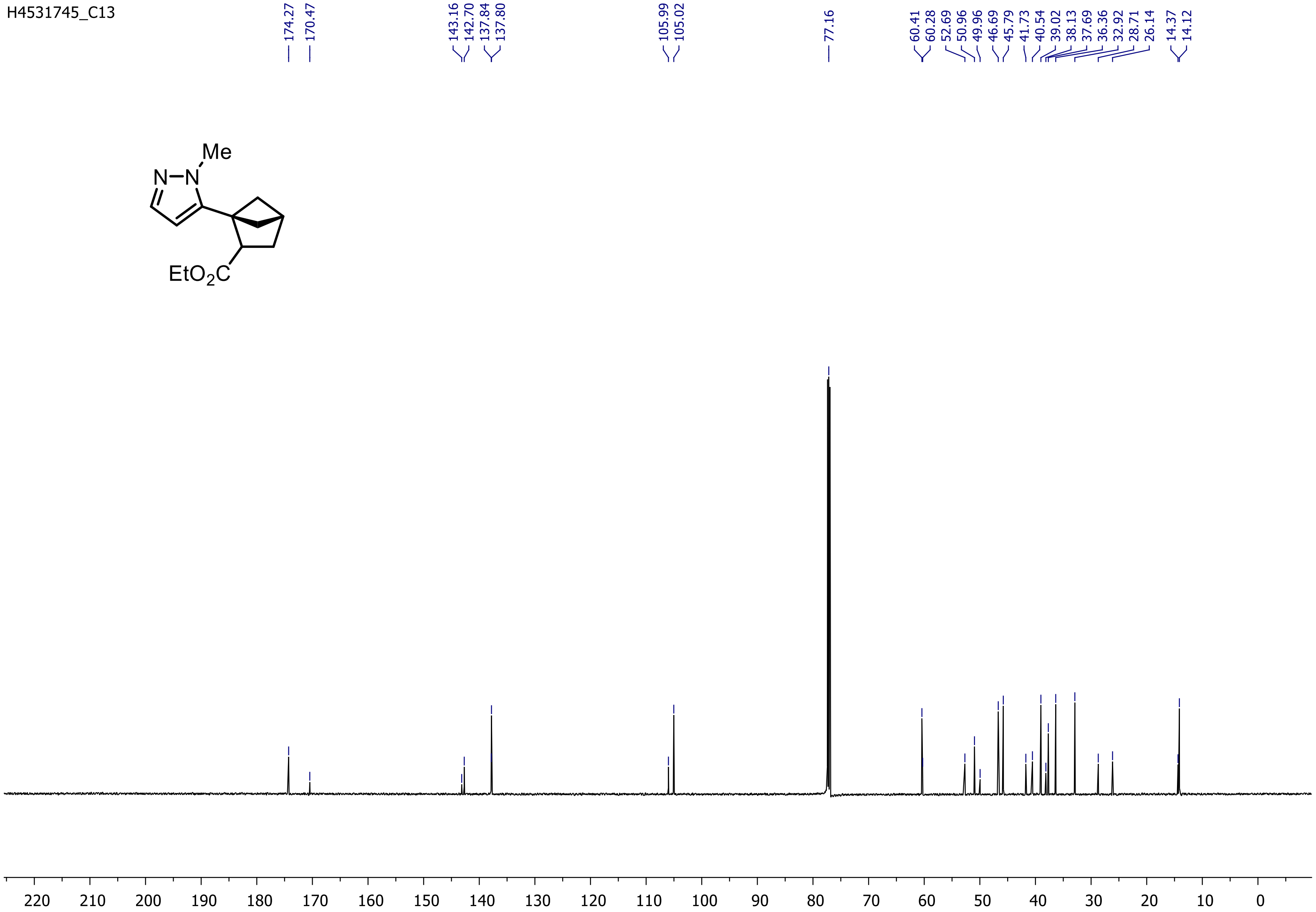

Compound (±)-17b

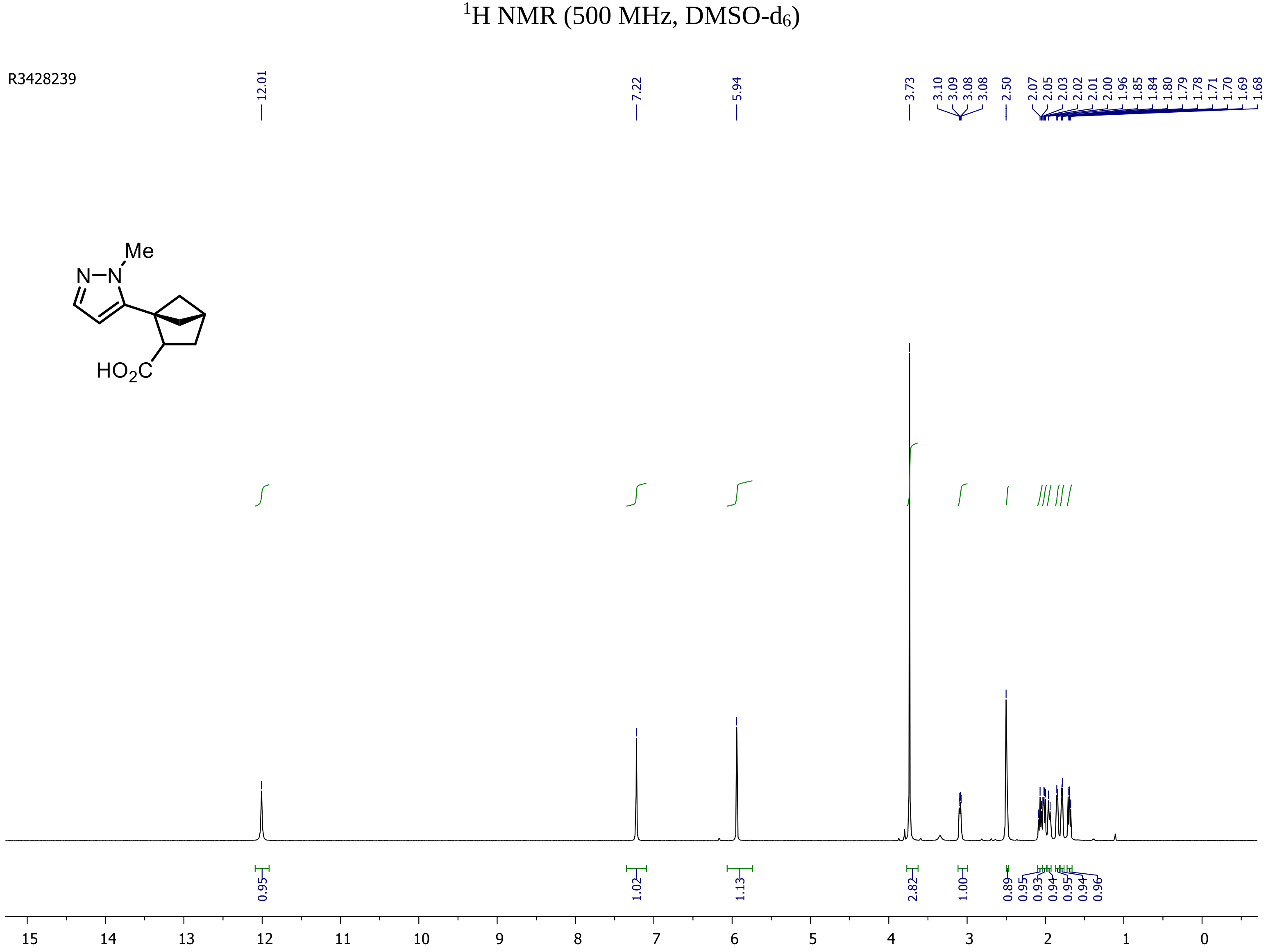

$^{13}C\{^1H\}$ NMR (126 MHz, DMSO-d$_6$)

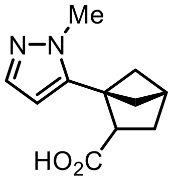

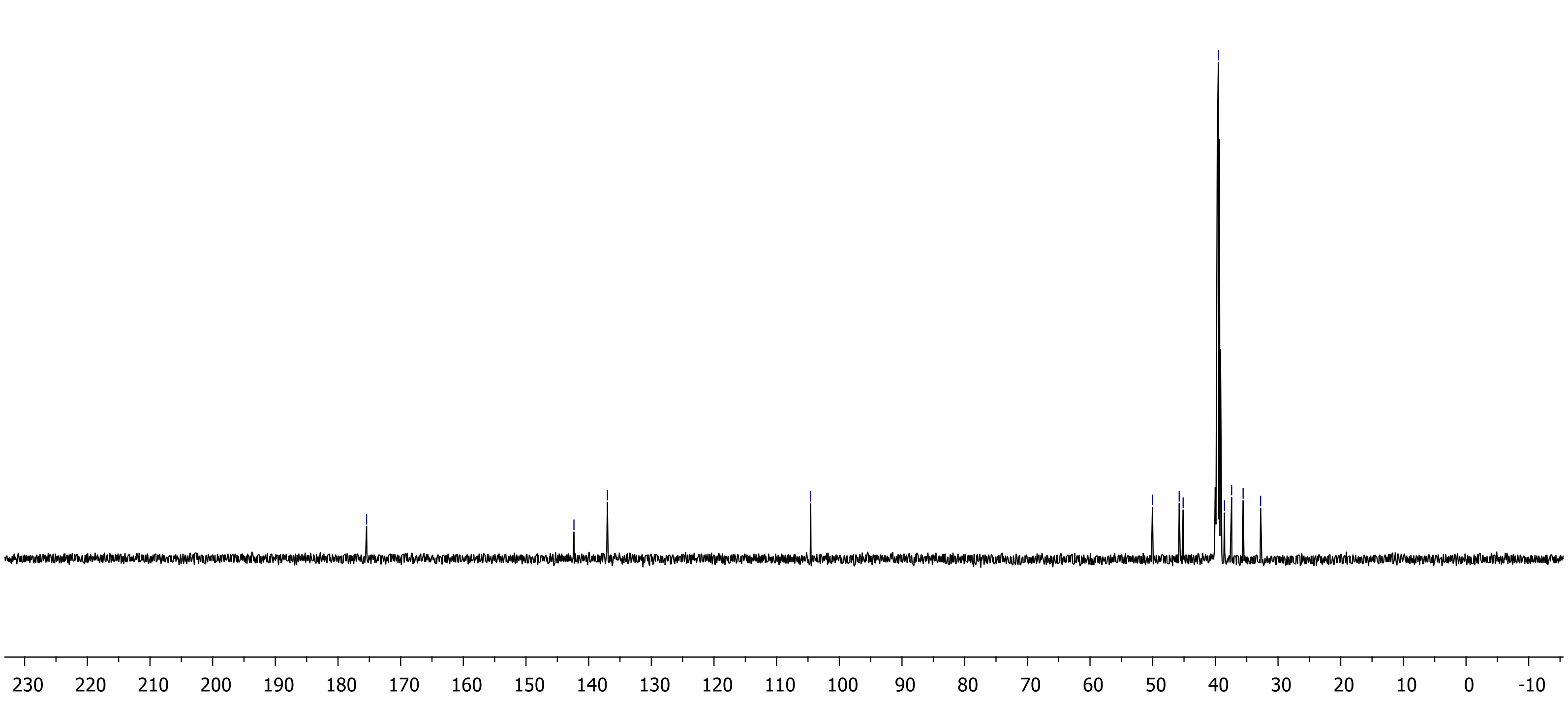

Ethyl-3-(1-methyl-1*H*-imidazol-2-yl)but-2-enoate

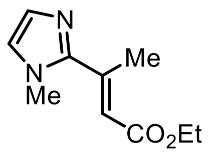

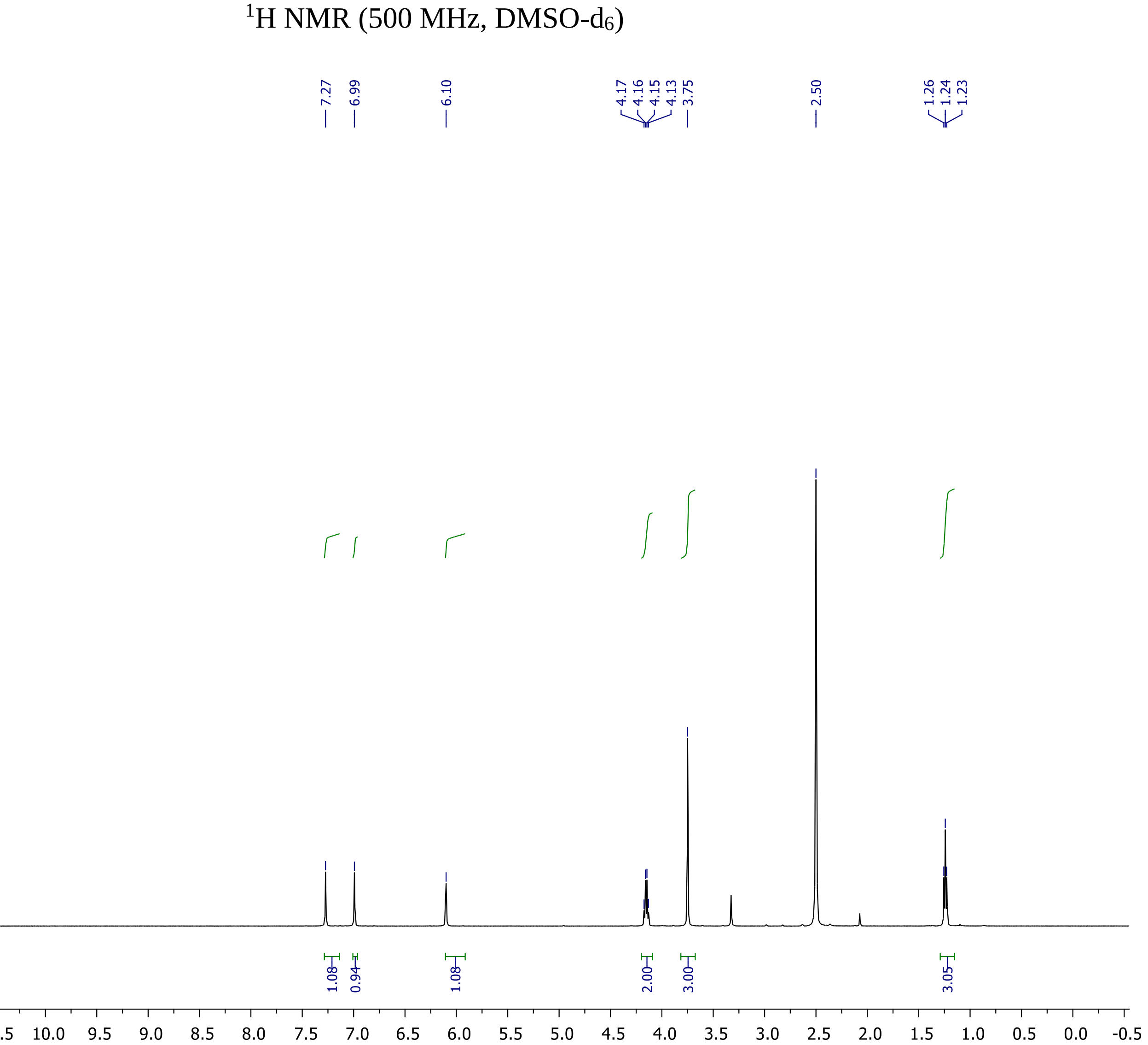

$^{13}C\{^1H\}$ NMR (151 MHz, $CDCl_3$)

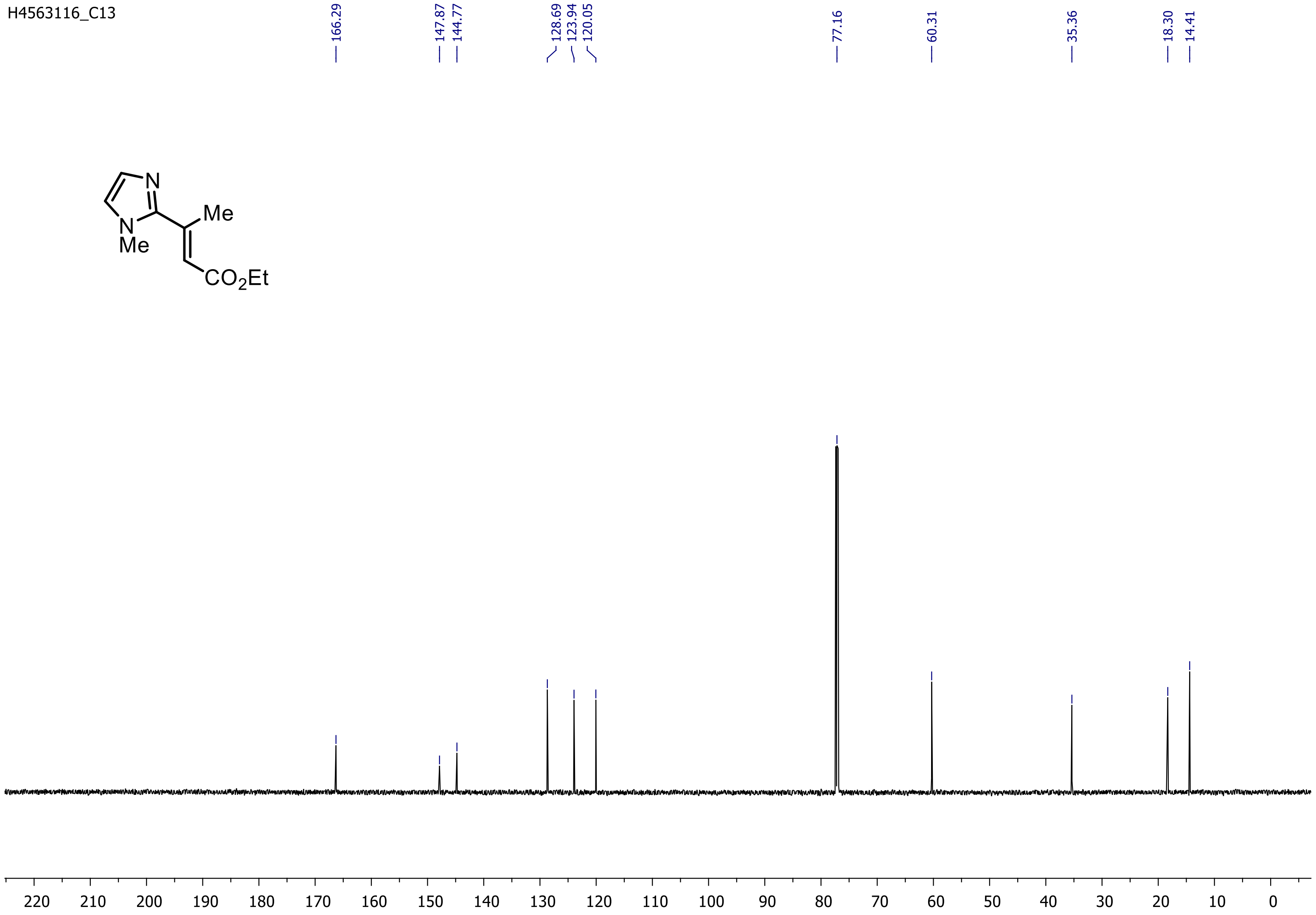

Compound 18

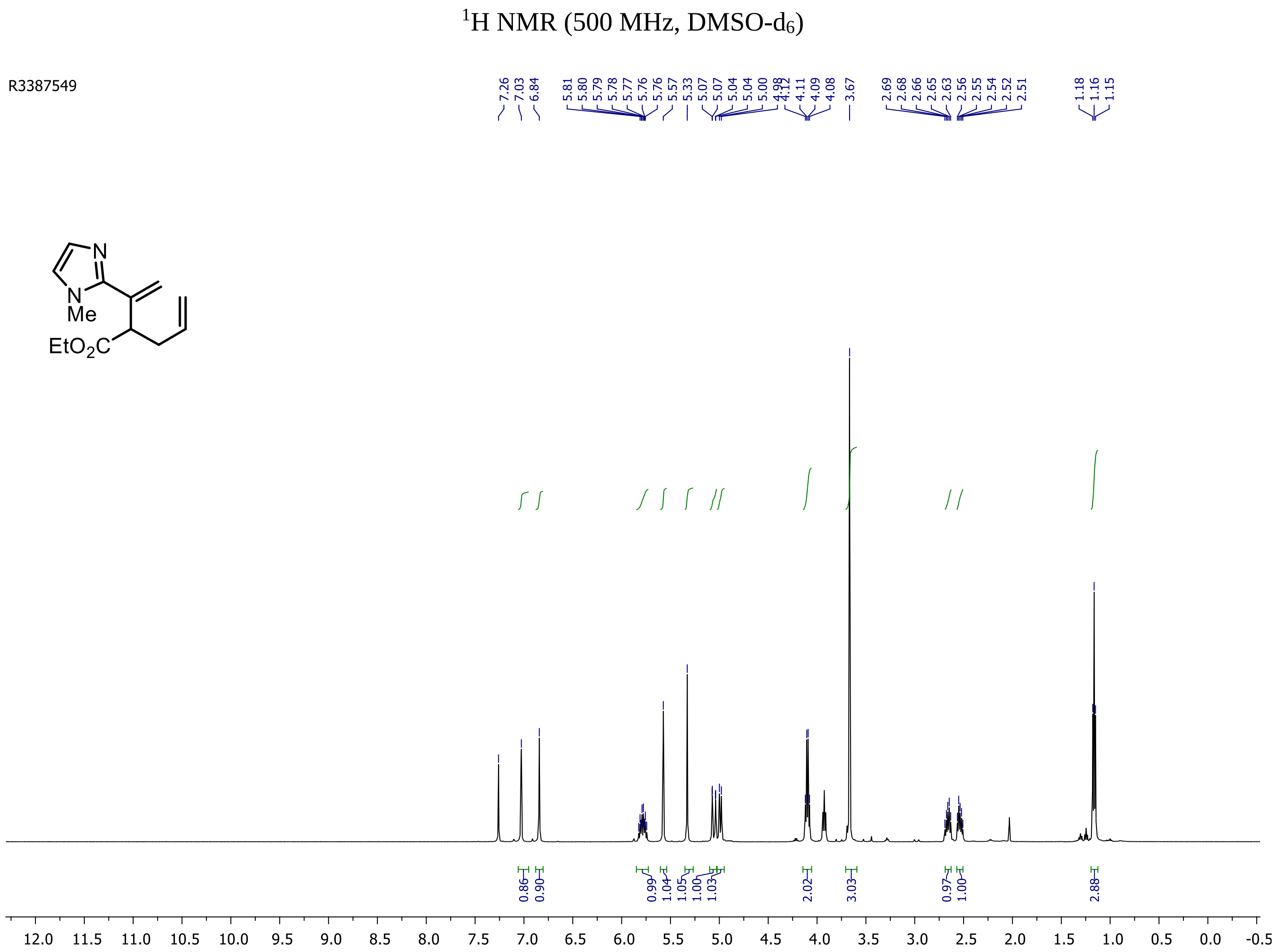

^{1}H NMR (500 MHz, DMSO-d$_6$)
R3387549
7.26
7.03
6.84
5.81
5.80
5.79
5.78
5.77
5.76
5.76
5.57
5.33
5.07
5.07
5.04
5.04
5.00
4.98
4.12
4.11
4.09
4.08
3.67
2.69
2.68
2.66
2.65
2.63
2.56
2.55
2.54
2.52
2.51
1.18
1.16
1.15
0.86
0.90
0.99
1.04
1.05
1.00
1.03
2.02
3.03
0.97
1.00
2.88
12.0 11.5 11.0 10.5 10.0 9.5 9.0 8.5 8.0 7.5 7.0 6.5 6.0 5.5 5.0 4.5 4.0 3.5 3.0 2.5 2.0 1.5 1.0 0.5 0.0 -0.5

$^{13}C\{^1H\}$ NMR (126 MHz, $CDCl_3$)

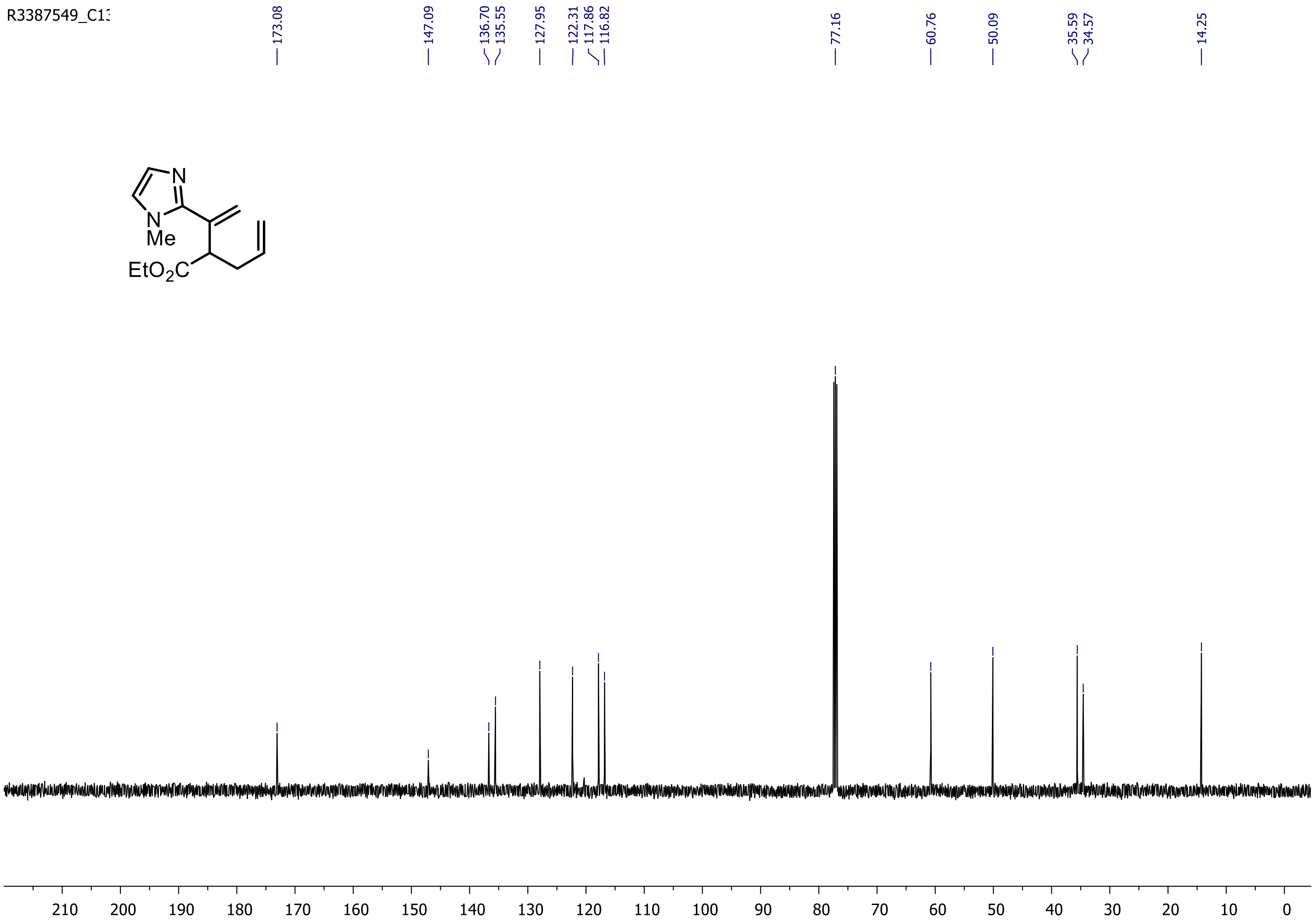

Compound (±)-18a

^{1}H NMR (500 MHz, $CDCl_3$)

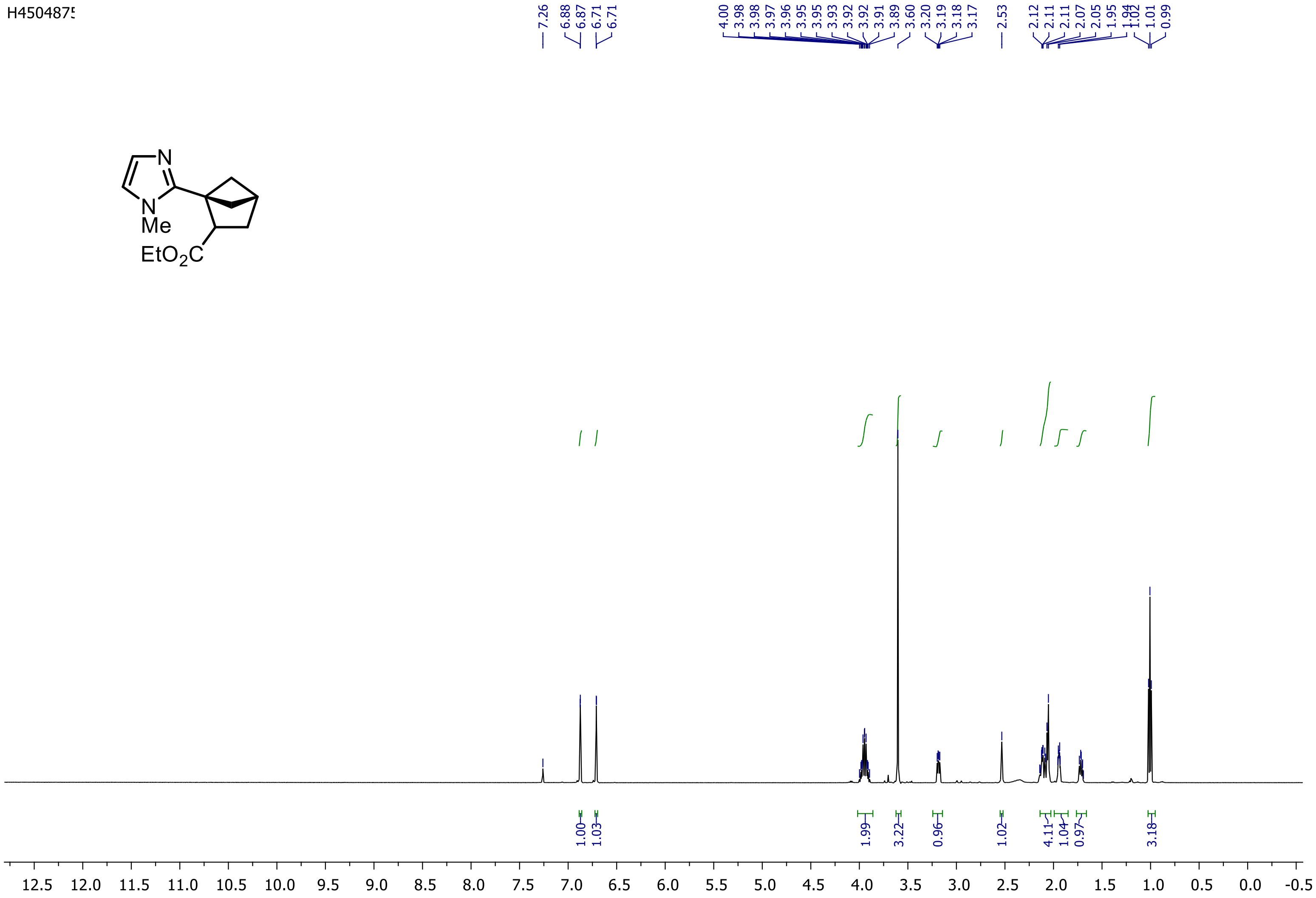

$^{13}C\{^1H\}$ NMR (126 MHz, $CDCl_3$)

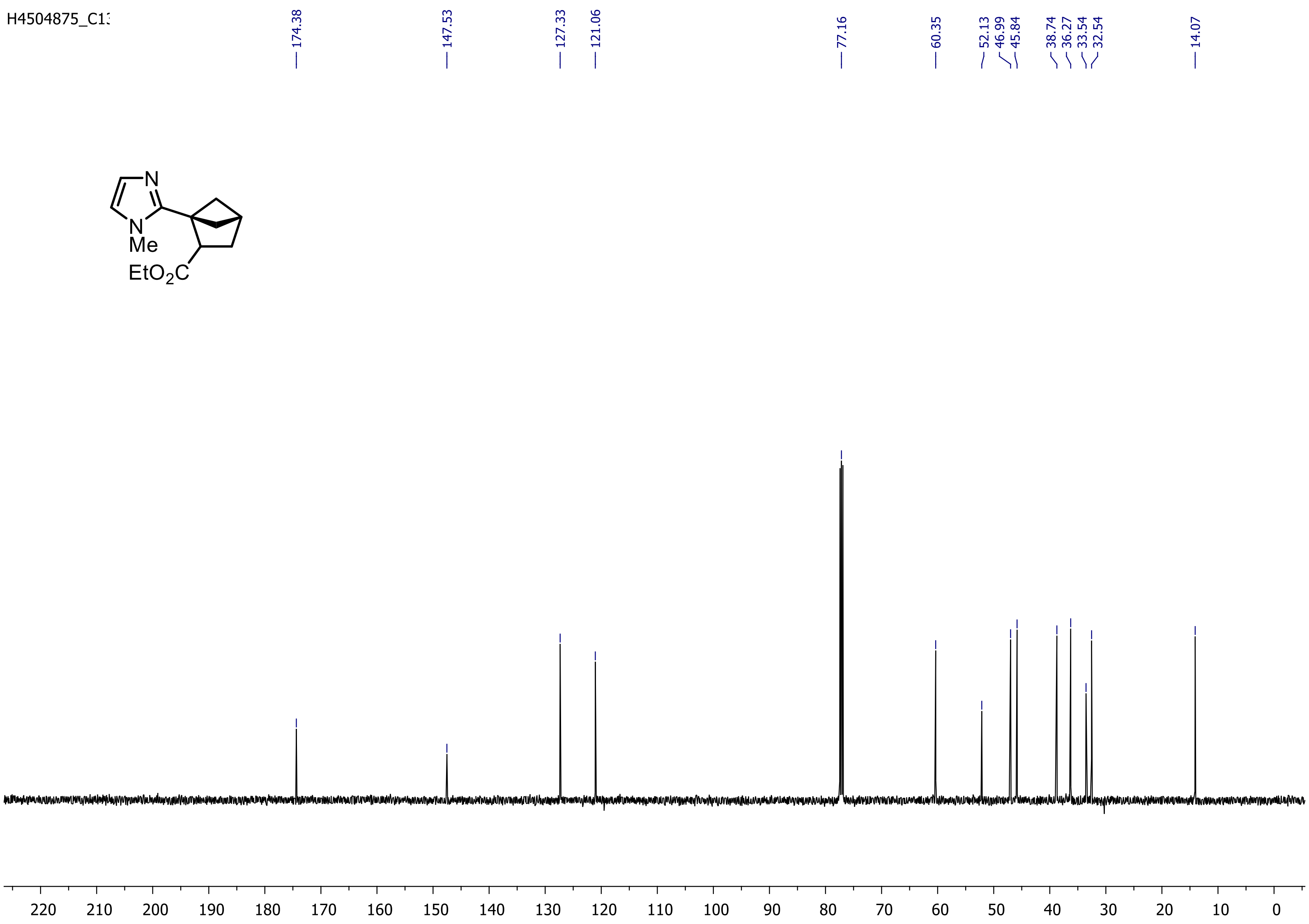

Compound (±)-18b

^{1}H NMR (500 MHz, DMSO-d_6)

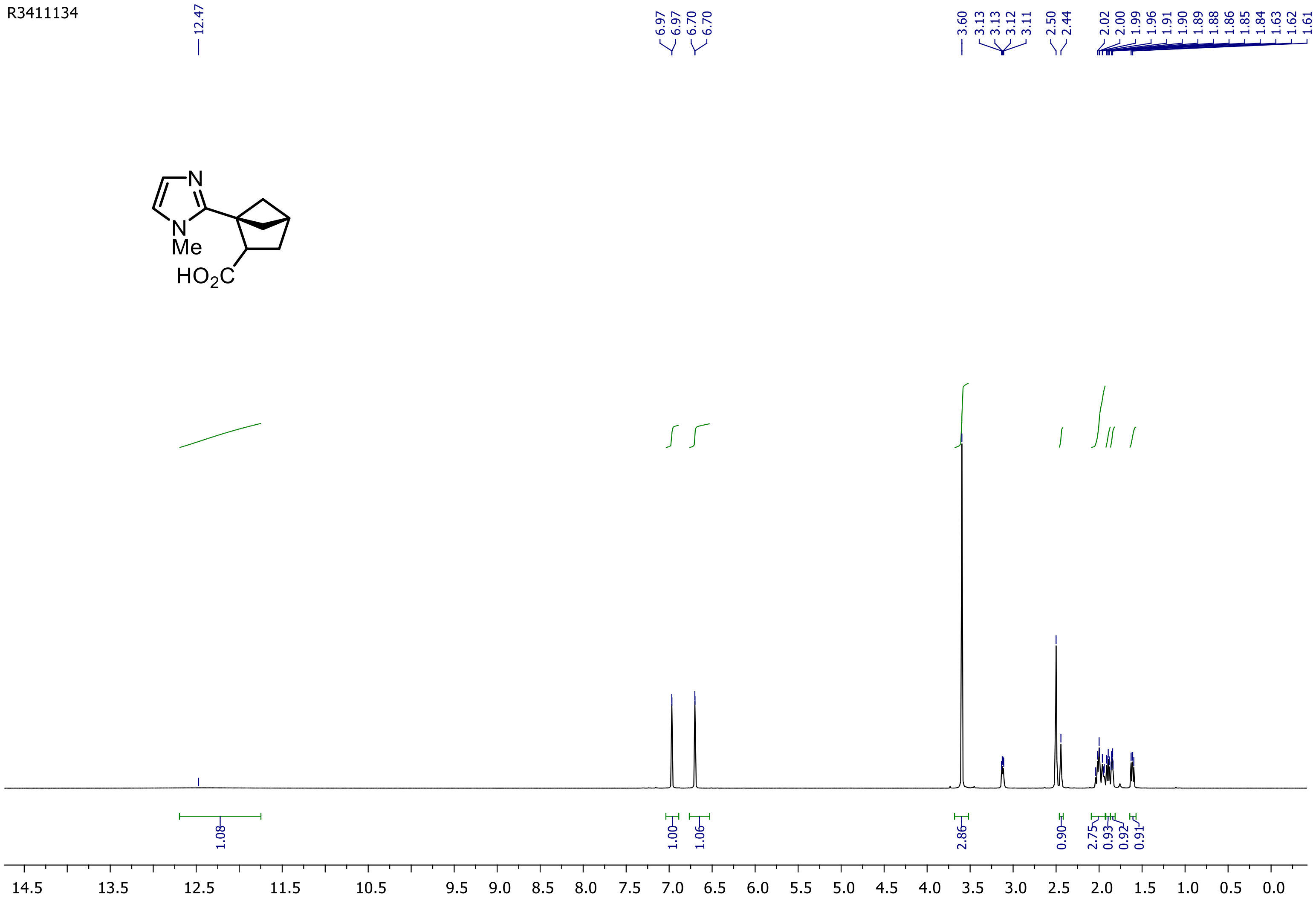

$^{13}C\{^{1}H\}$ NMR (126 MHz, DMSO-d_6)

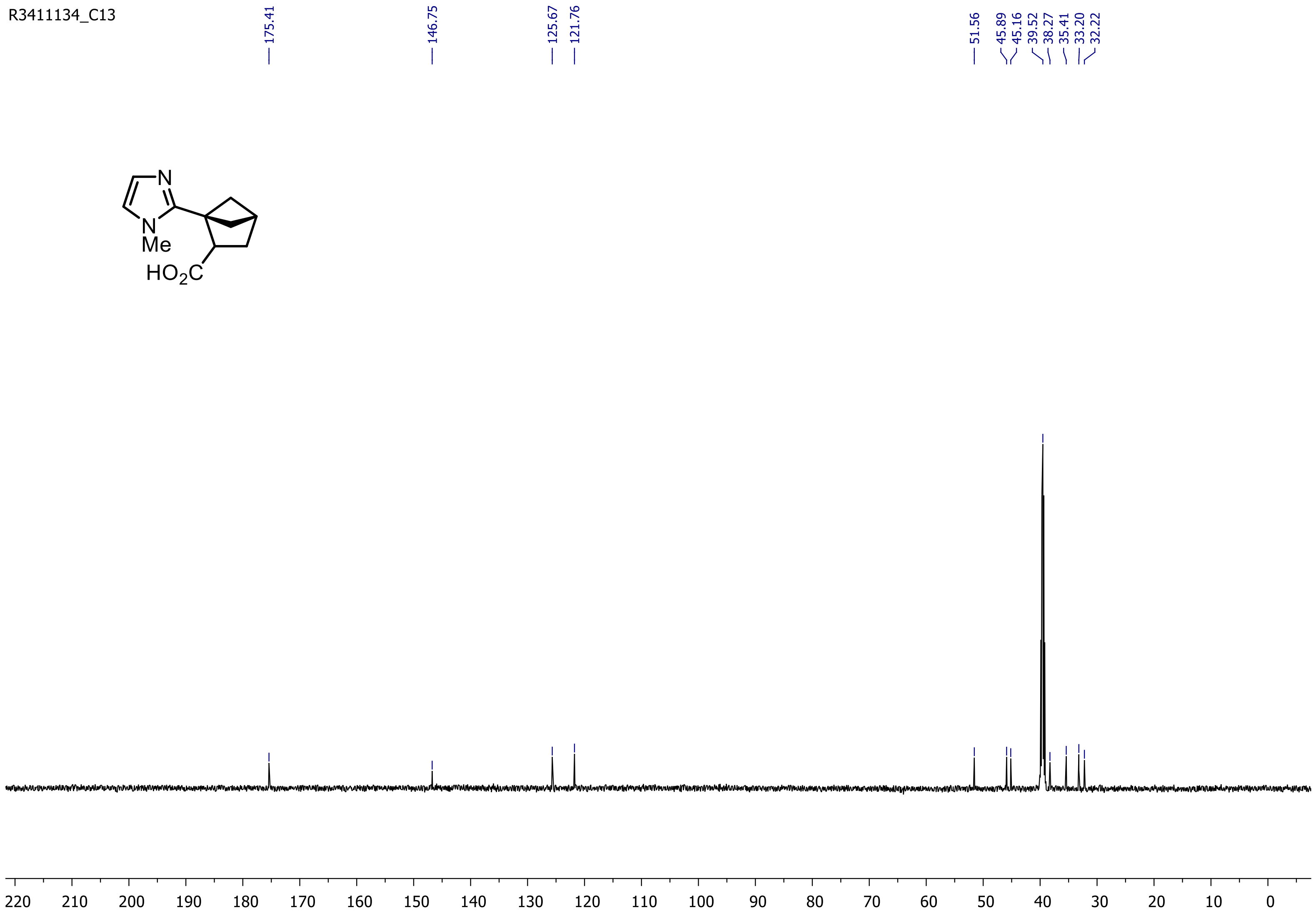

Ethyl-3-(thiophen-2-yl)but-2-enoate

^{1}H NMR (500 MHz, $CDCl_3$)

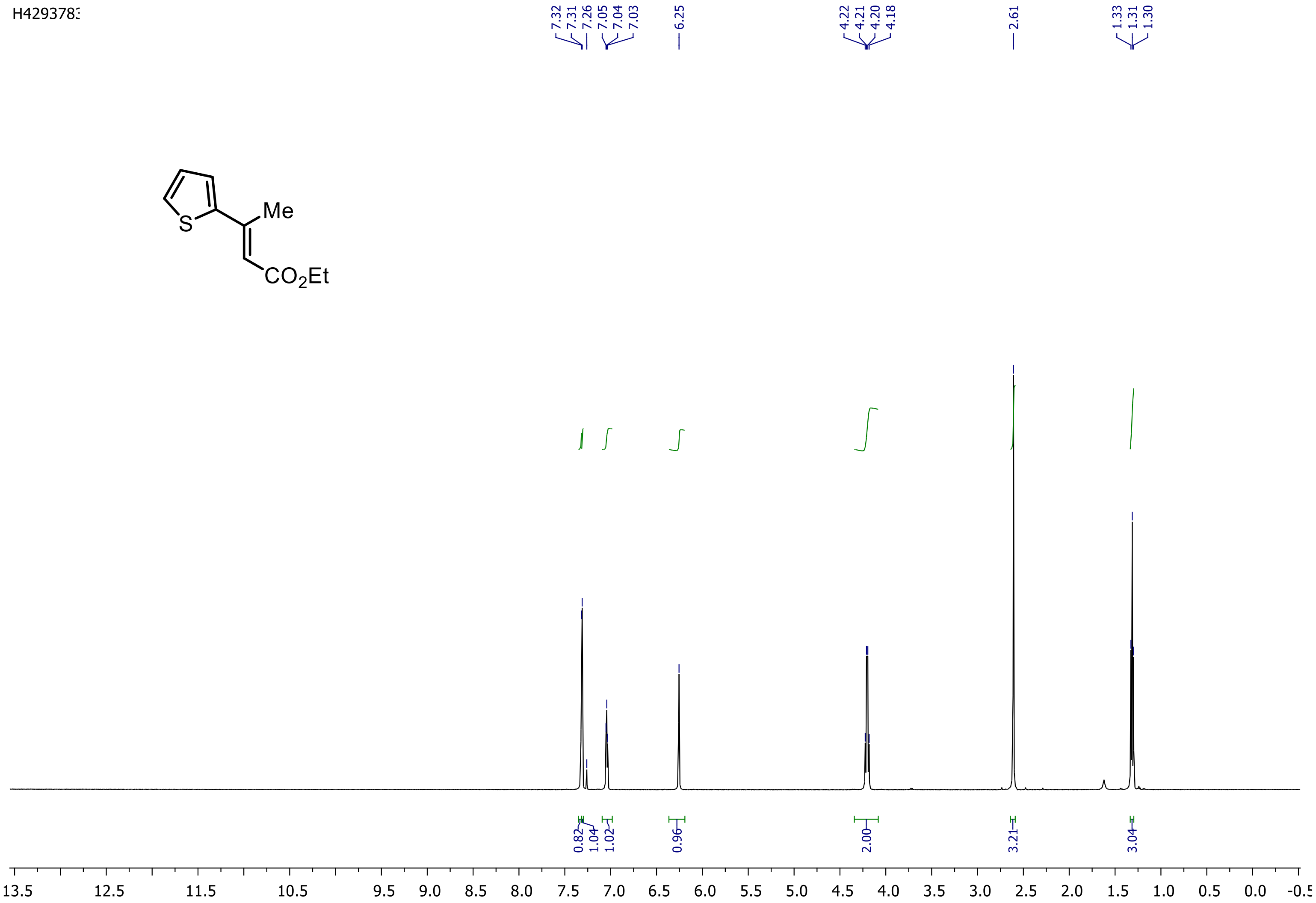

$^{13}C\{^{1}H\}$ NMR (126 MHz, $CDCl_3$)

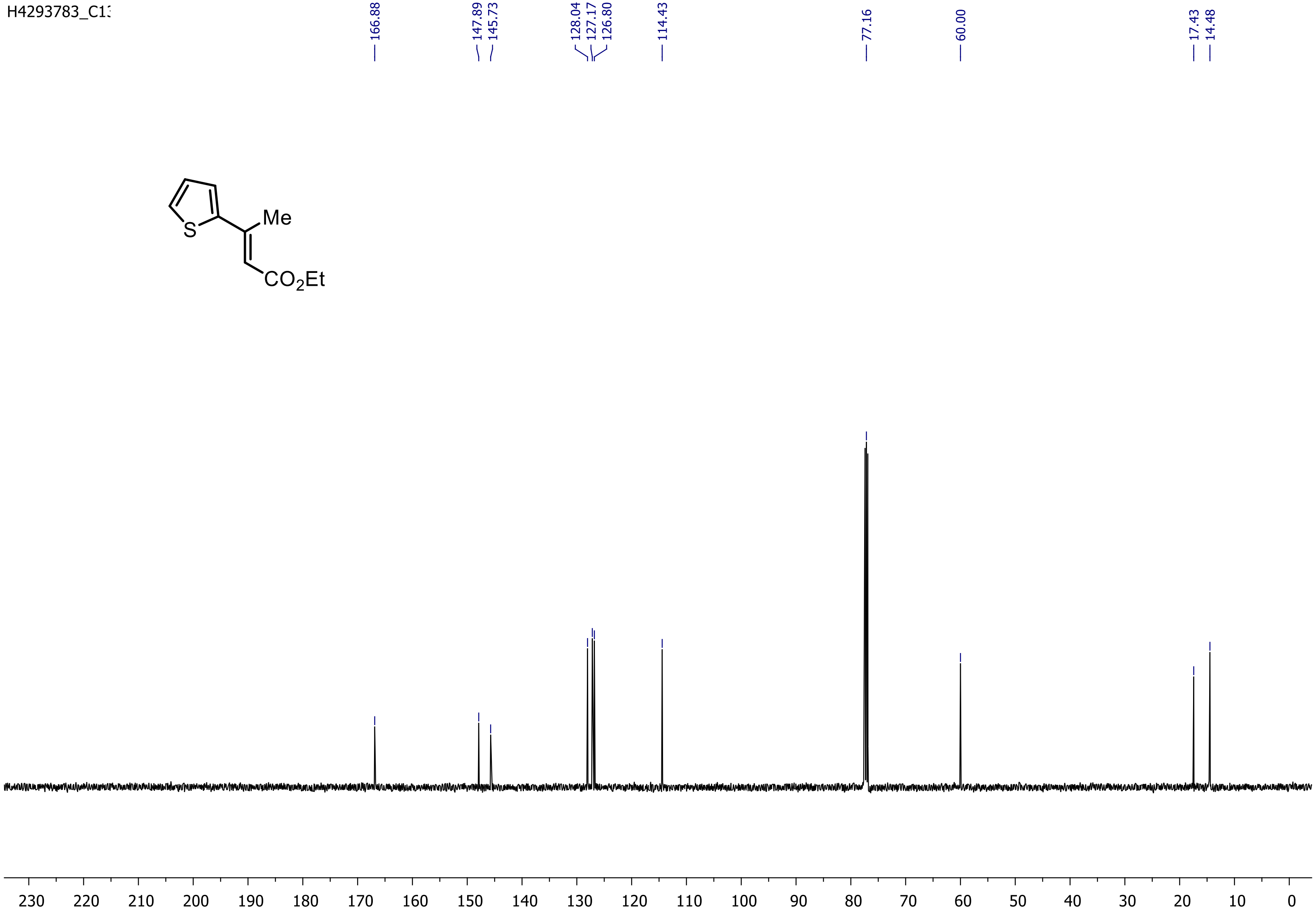

Compound 19

^{1}H NMR (500 MHz, $CDCl_3$)

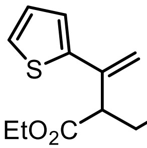

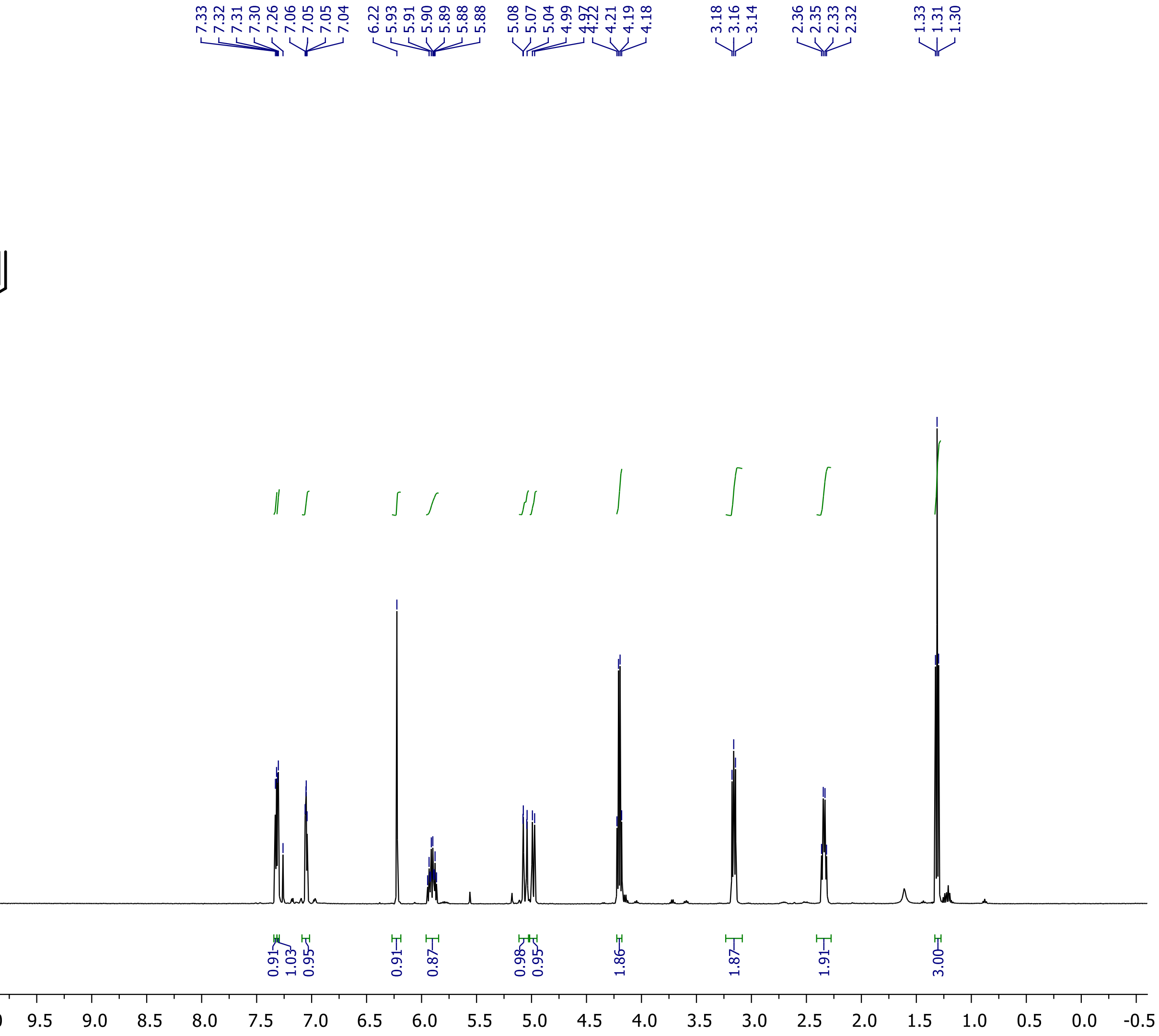

$^{13}C\{^{1}H\}$ NMR (126 MHz, $CDCl_3$)

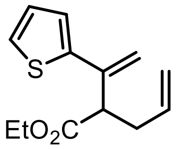

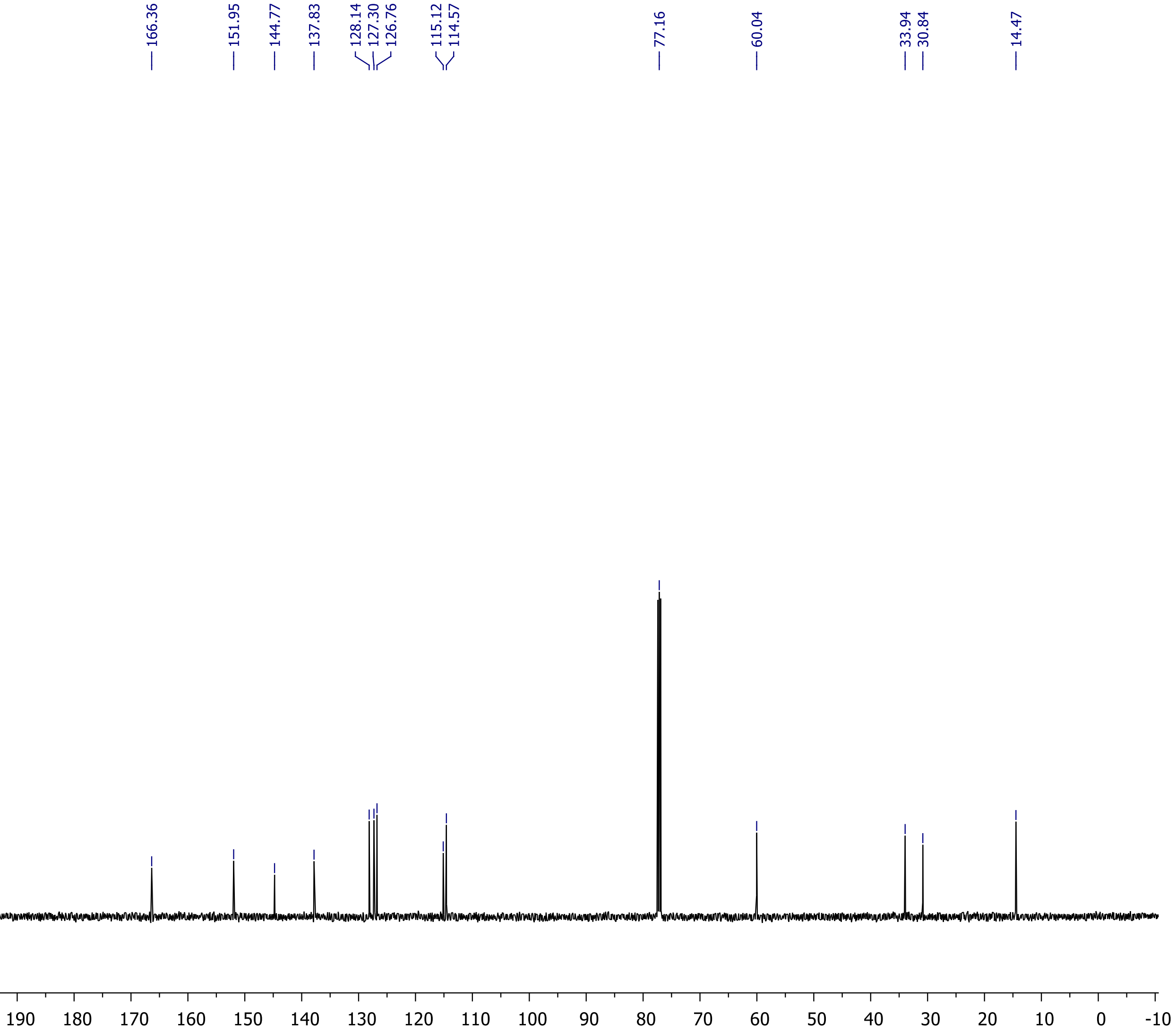

Compound (±)-19a

^{1}H NMR (500 MHz, $CDCl_3$)

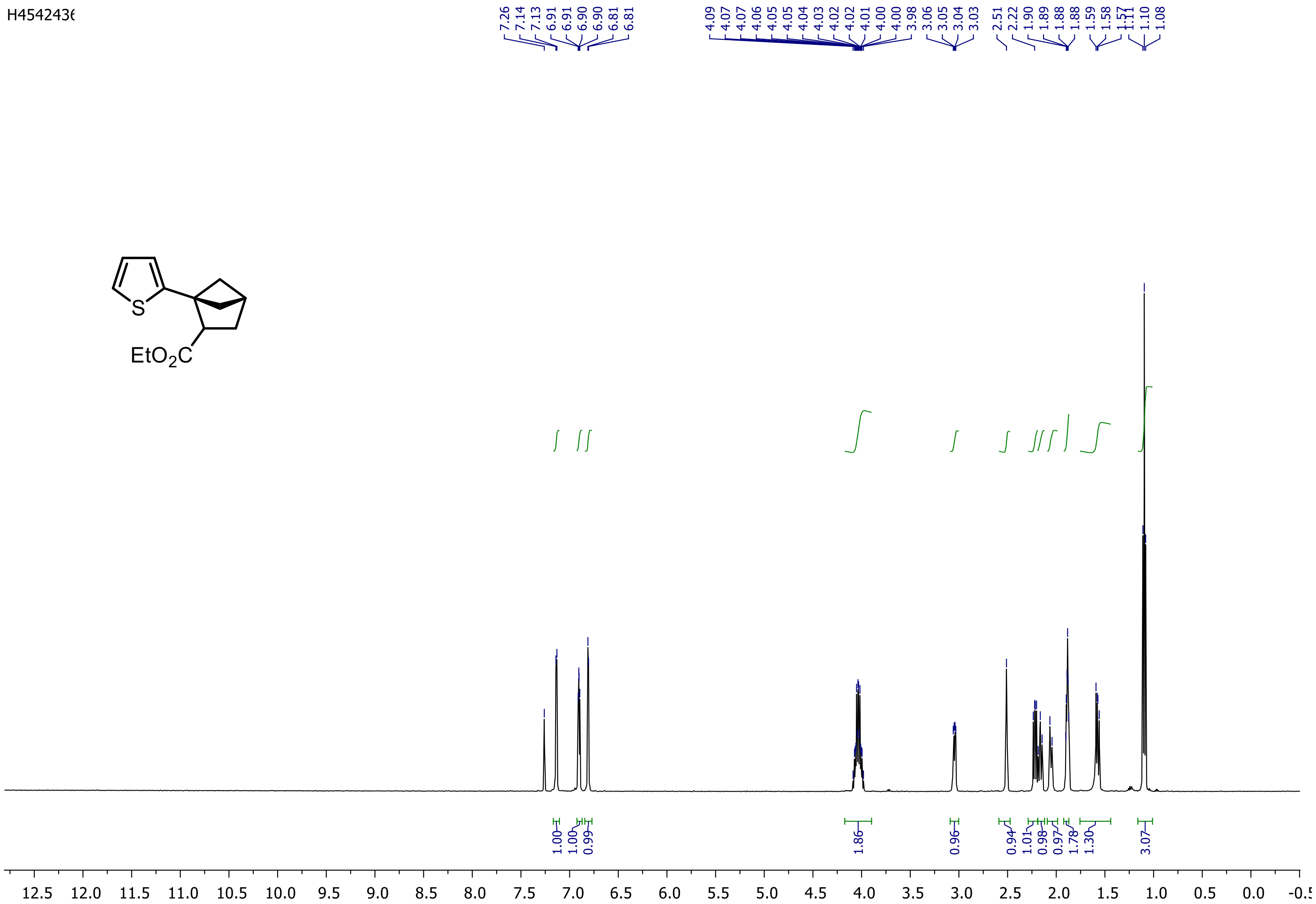

$^{13}C\{^1H\}$ NMR (126 MHz, $CDCl_3$)

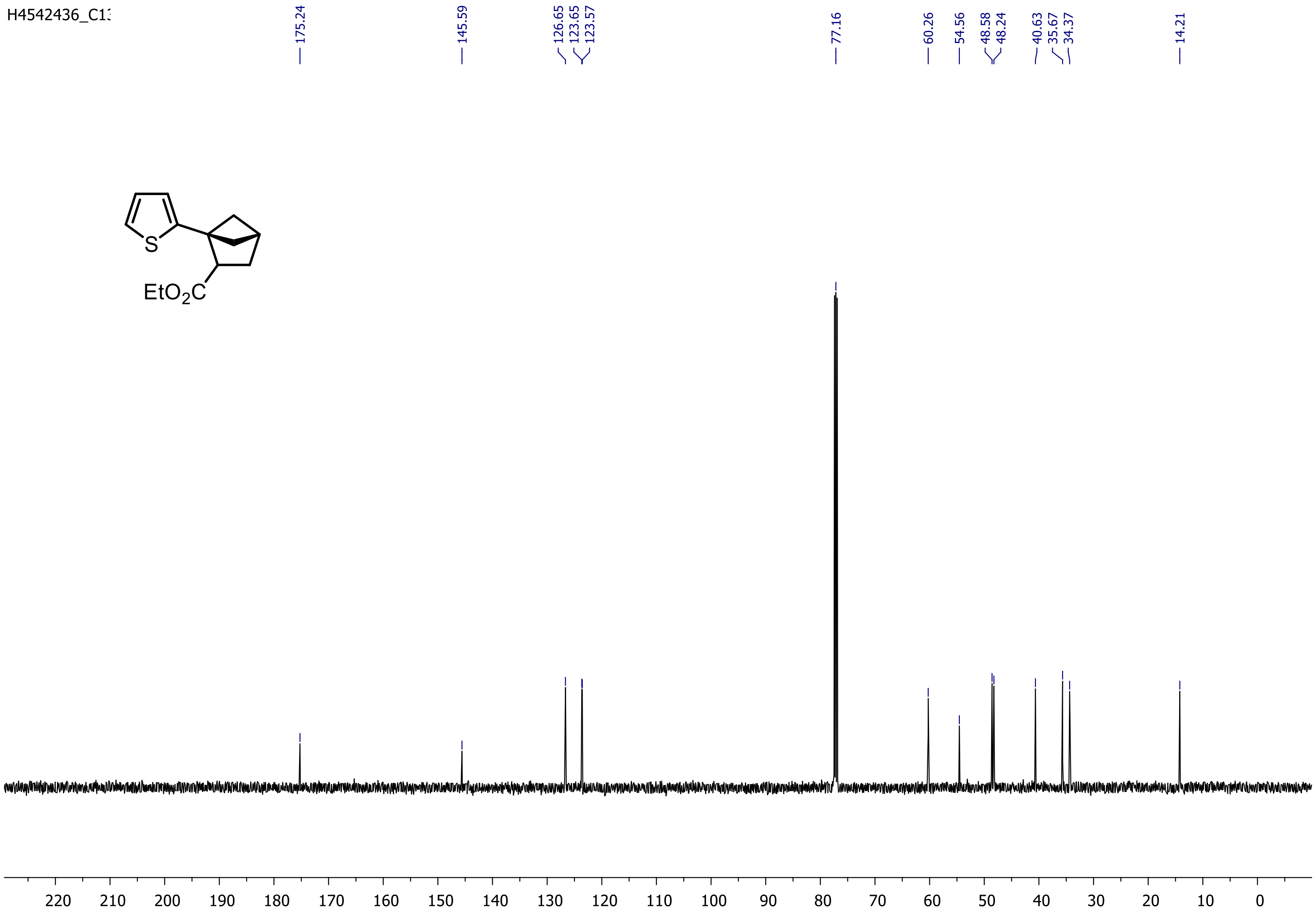

Compound (±)-19b

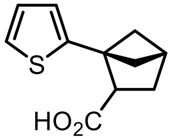

^{1}H NMR (500 MHz, DMSO-d_6)

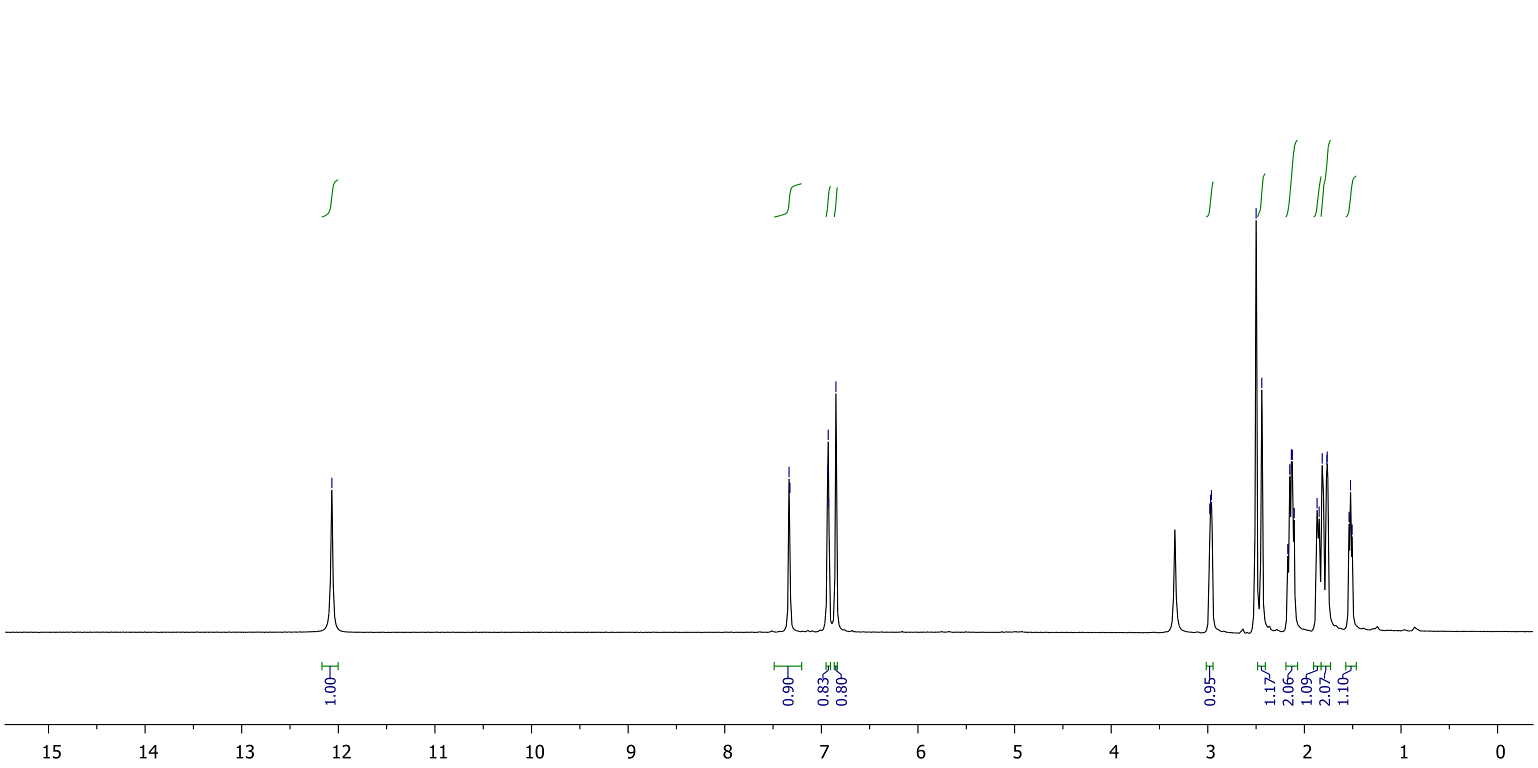

$^{13}C\{^{1}H\}$ NMR (126 MHz, DMSO-d_6)

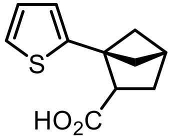

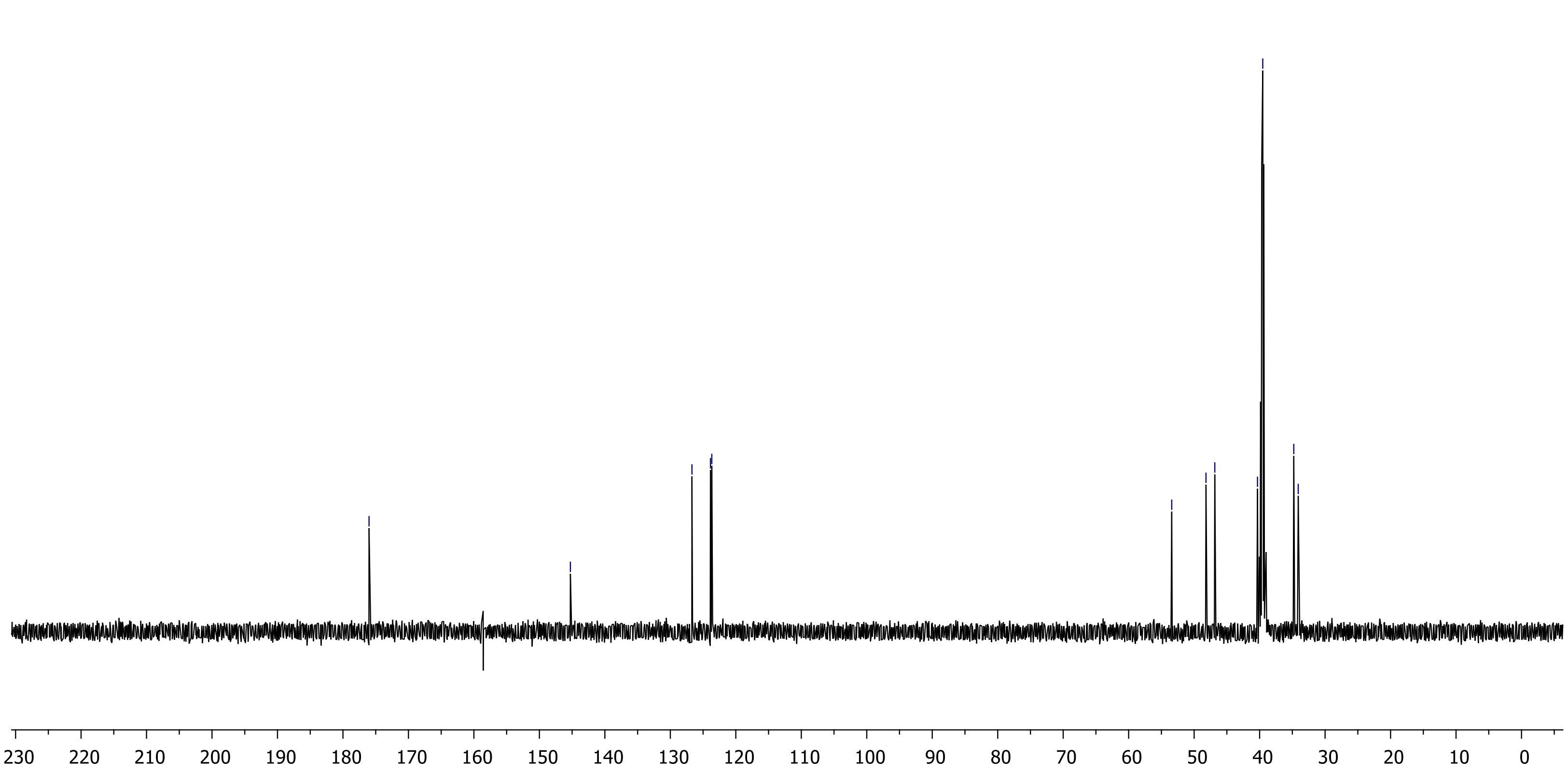

Ethyl-3-(furan-2-yl)but-2-enoate

^{1}H NMR (500 MHz, $CDCl_3$)

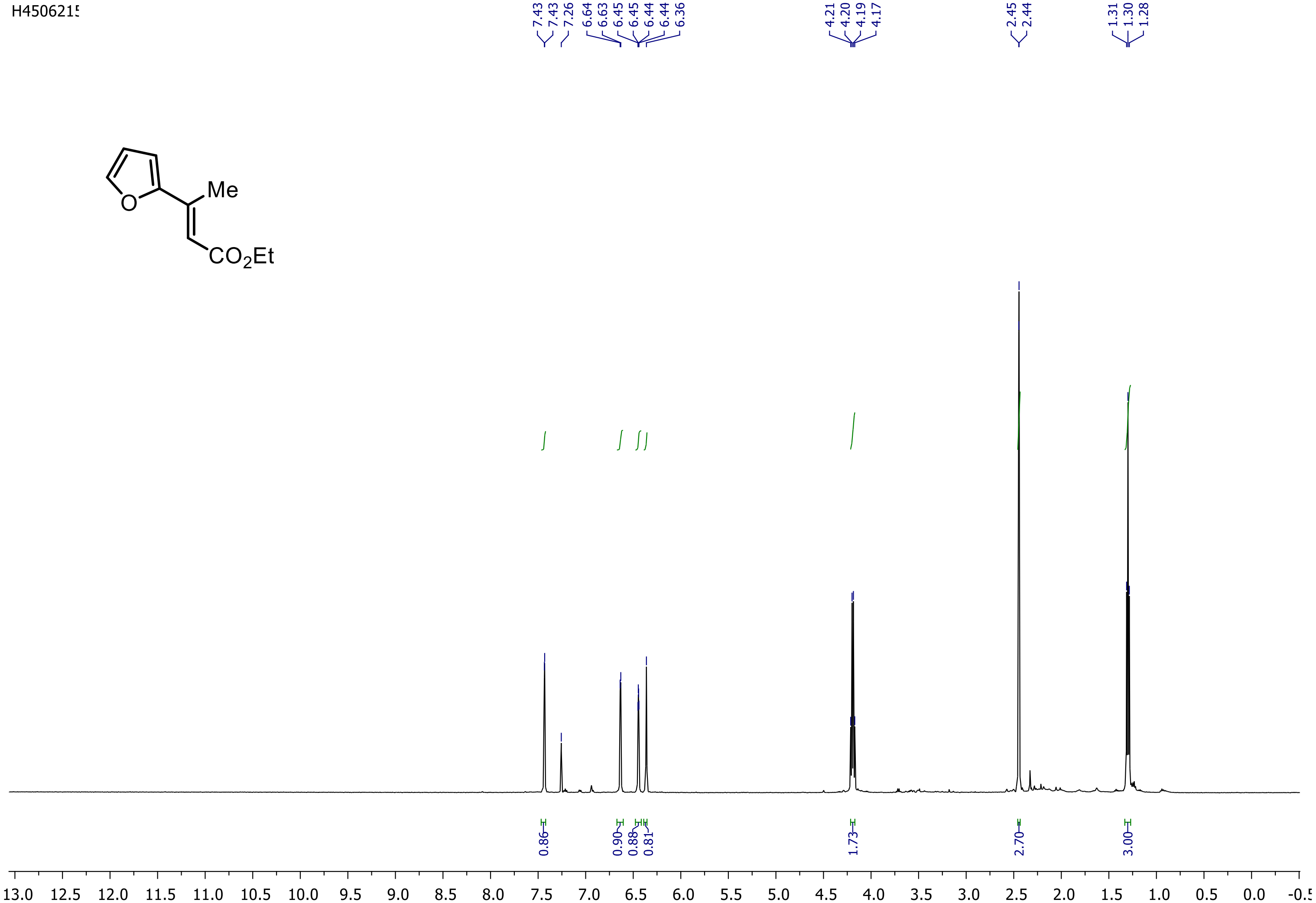

$^{13}C\{^{1}H\}$ NMR (126 MHz, $CDCl_3$)

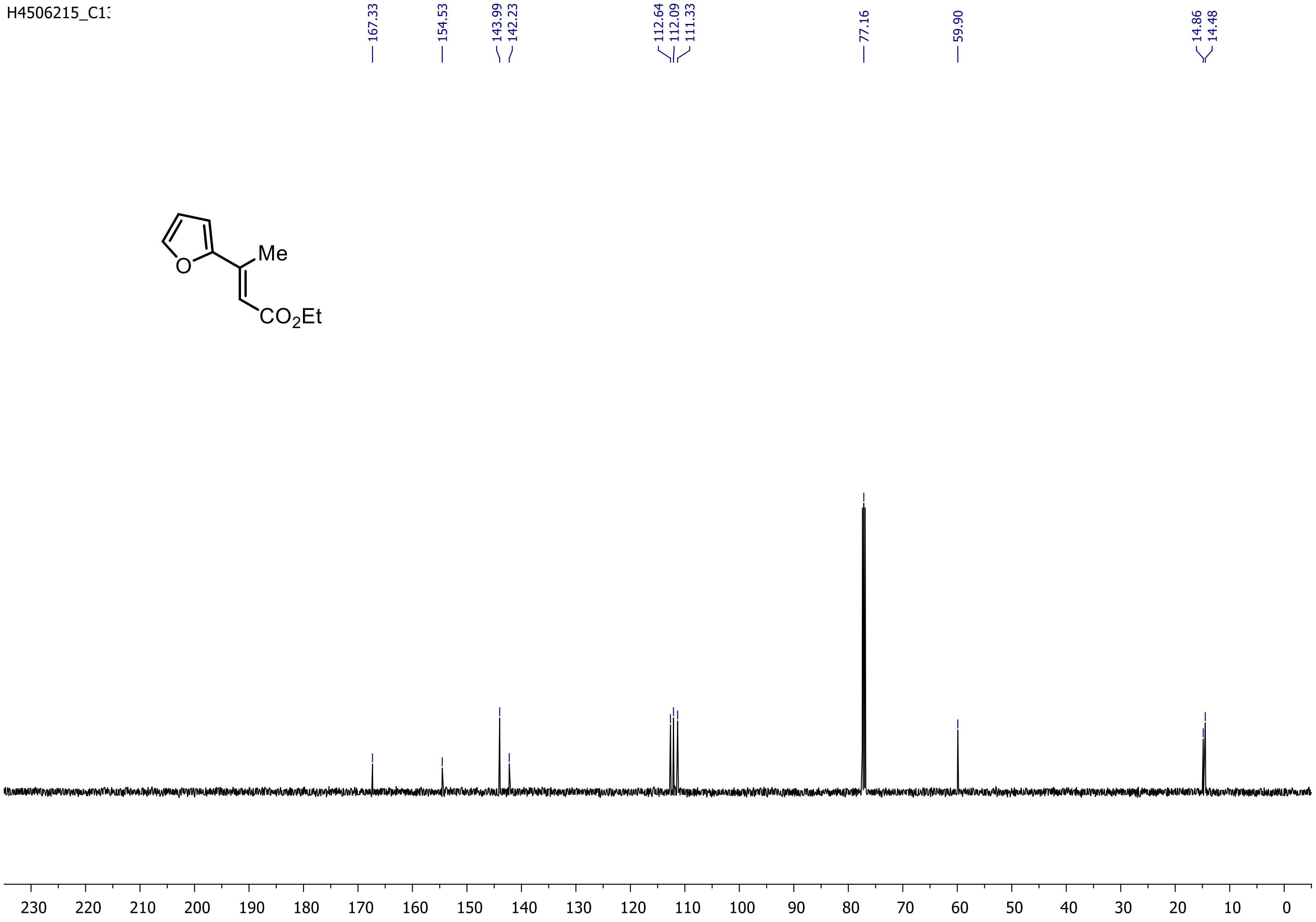

Compound (±)-20a

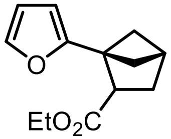

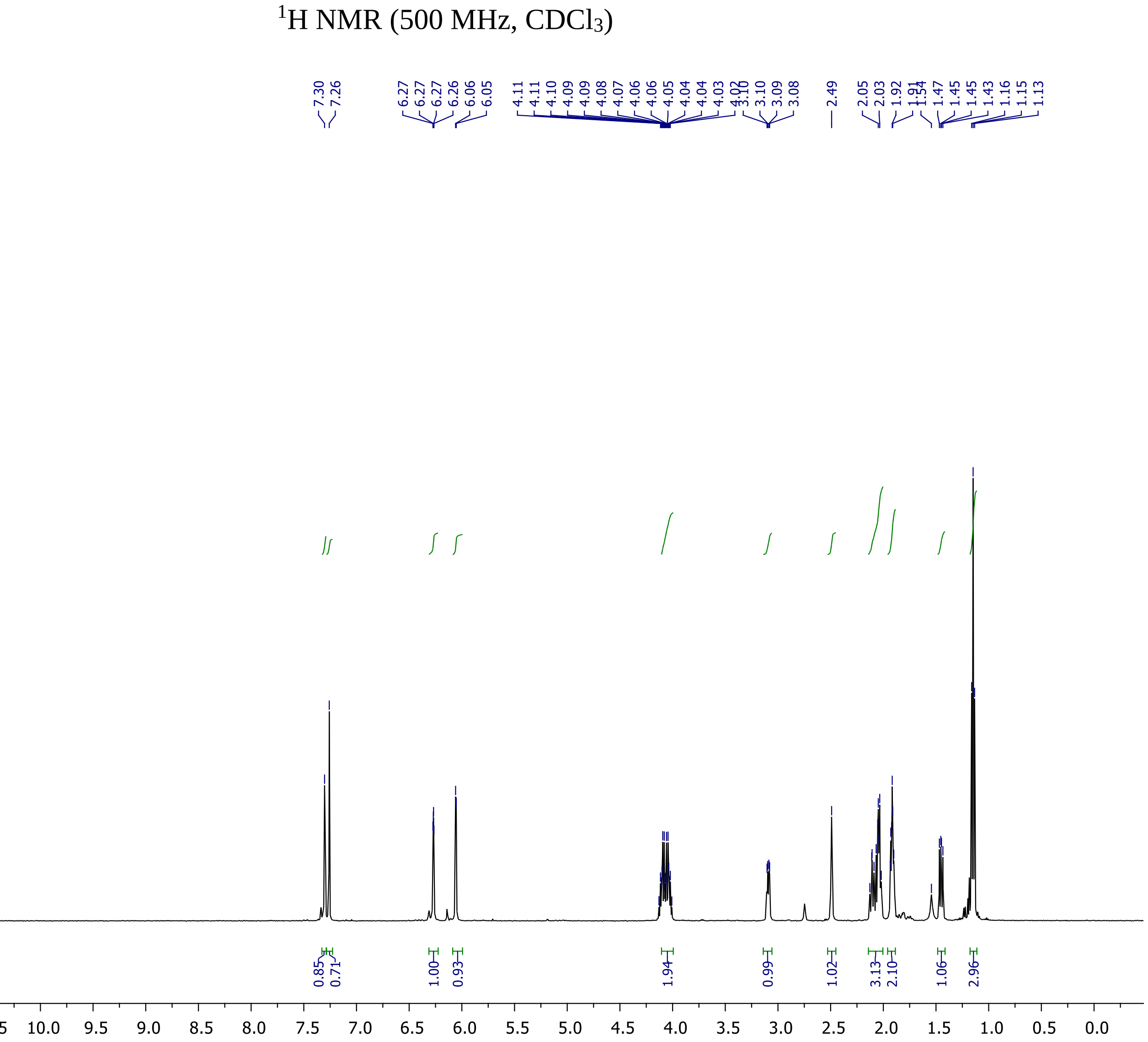

$^{13}C \{^1H\}$ NMR (151 MHz, $CDCl_3$)

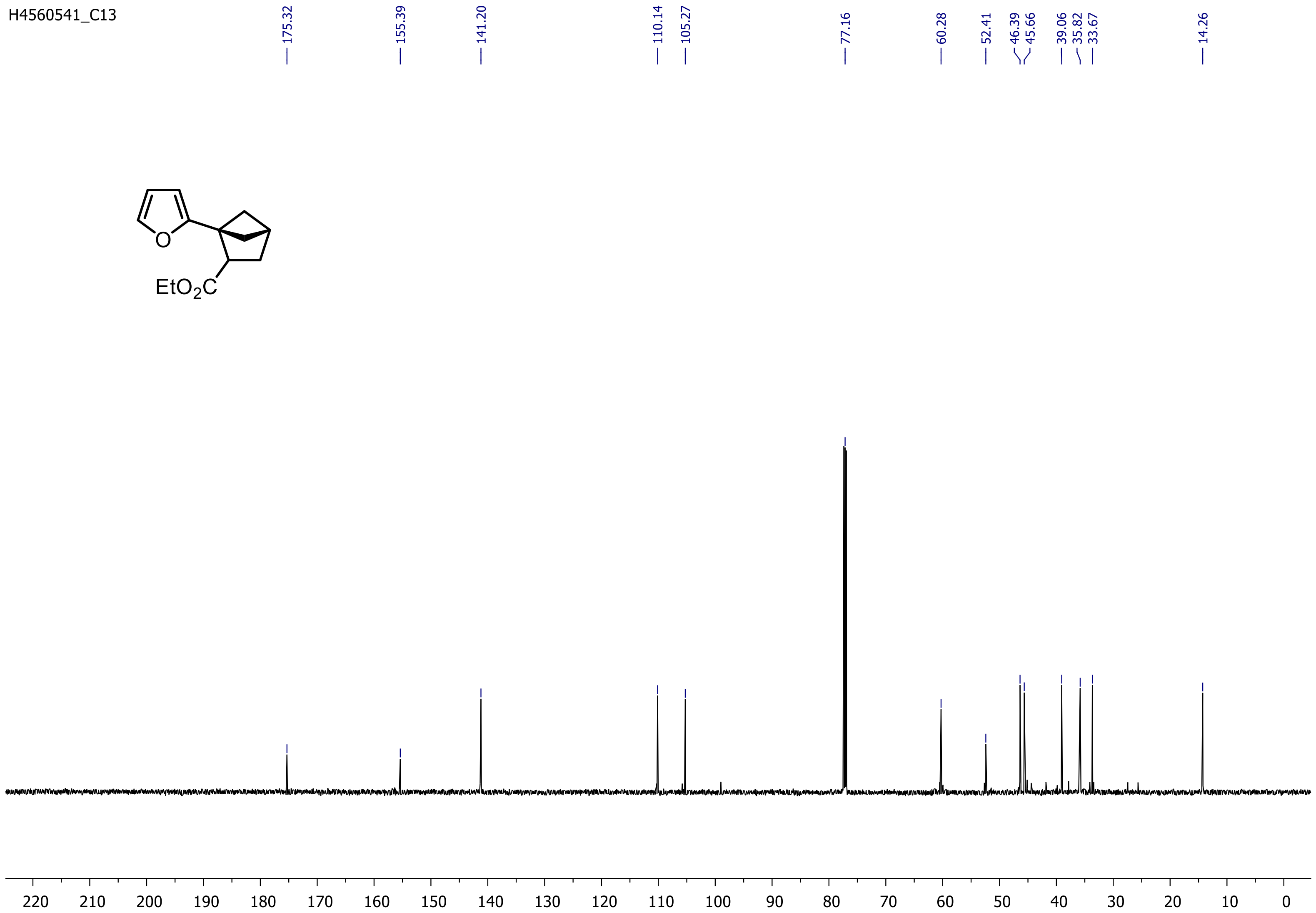

Compound (±)-20b

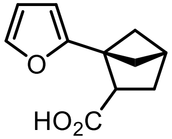

^{1}H NMR (500 MHz, DMSO-d$_6$)

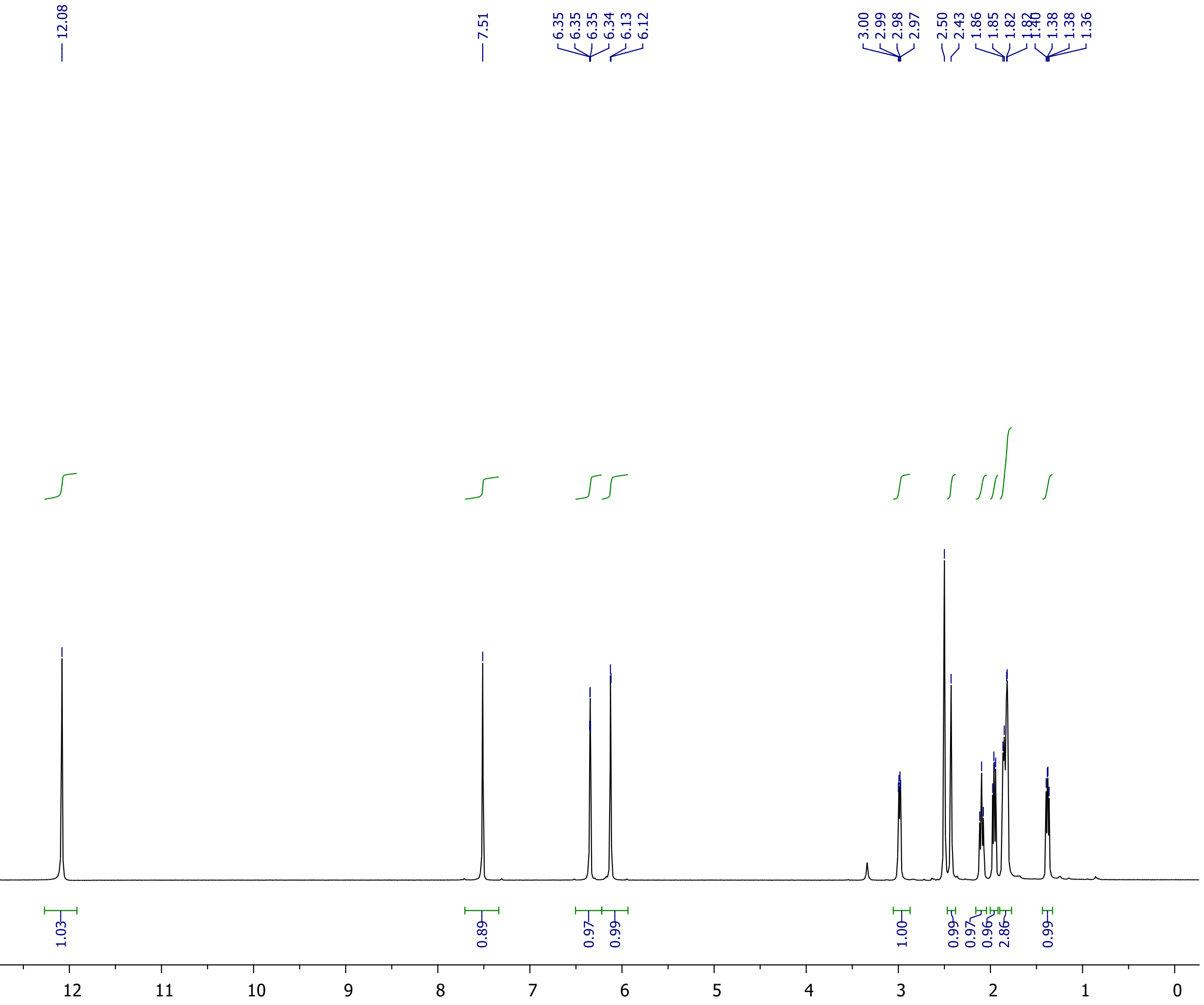

$^{13}C\{^{1}H\}$ NMR (126 MHz, DMSO-d_6)

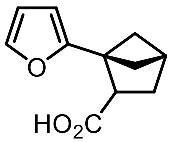

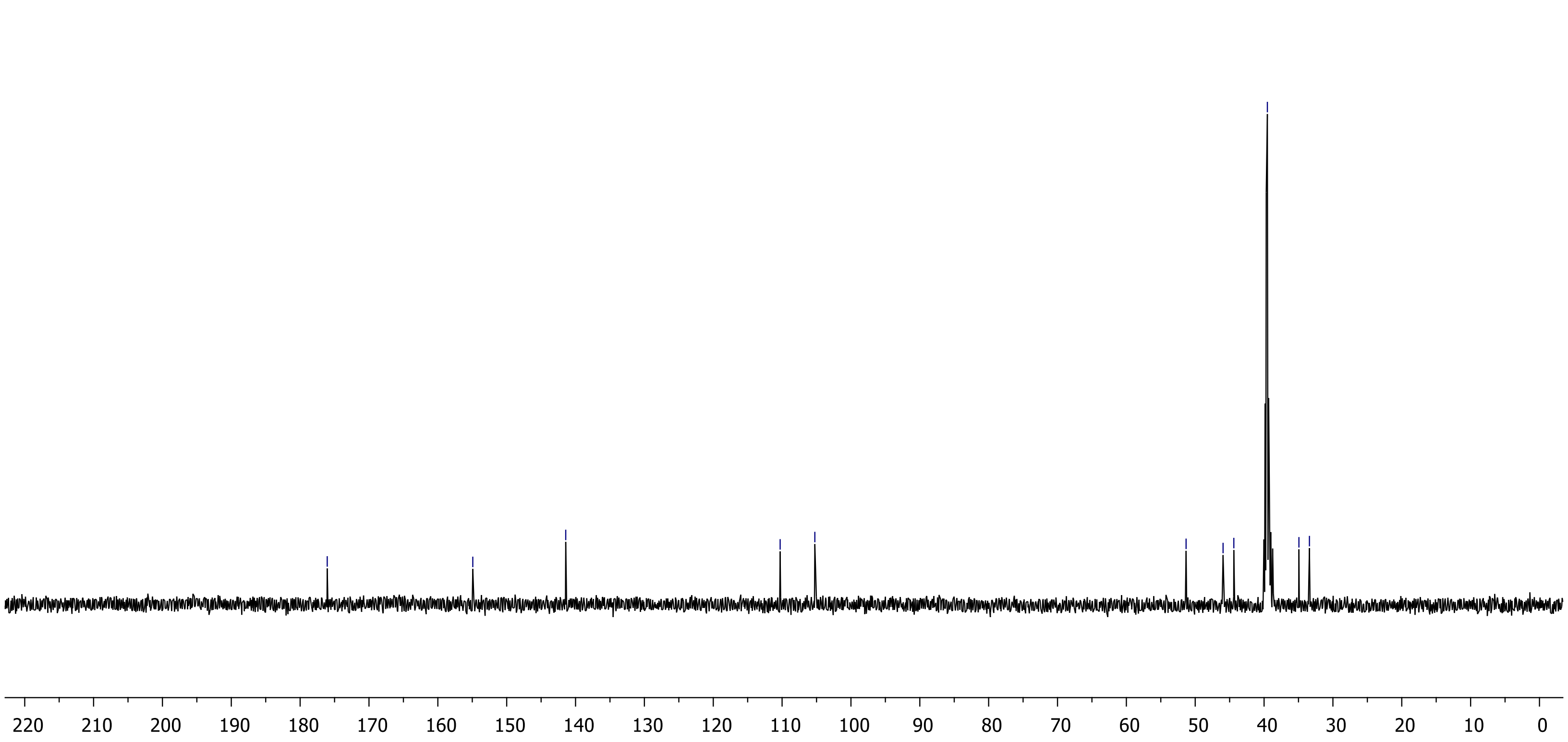

Ethyl-3-(pyridin-4-yl)but-2-enoate

^{1}H NMR (500 MHz, DMSO-d$_6$)

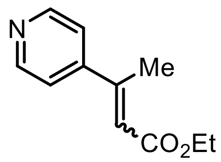

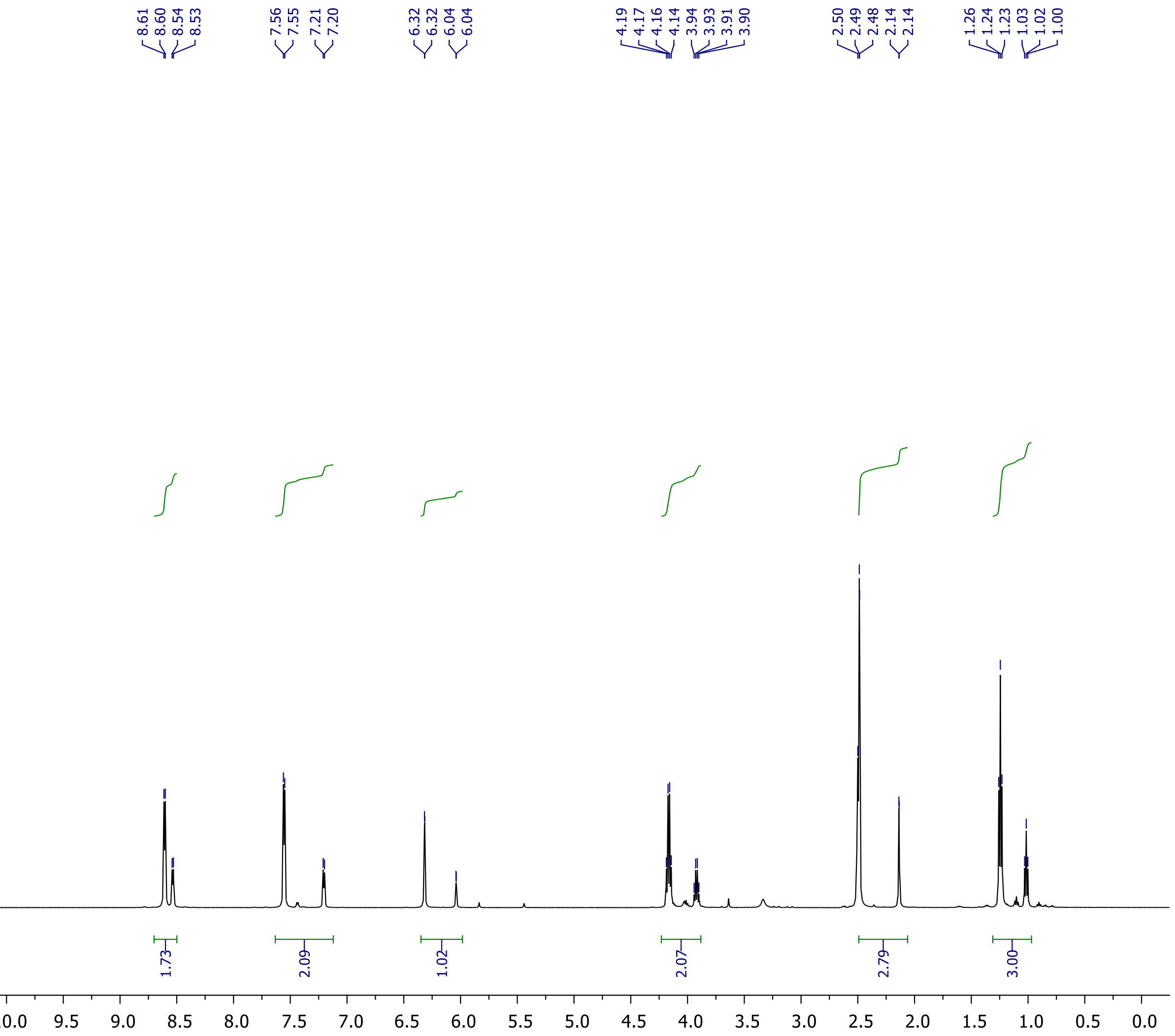

$^{13}C\{^{1}H\}$ NMR (126 MHz, DMSO-d_6)

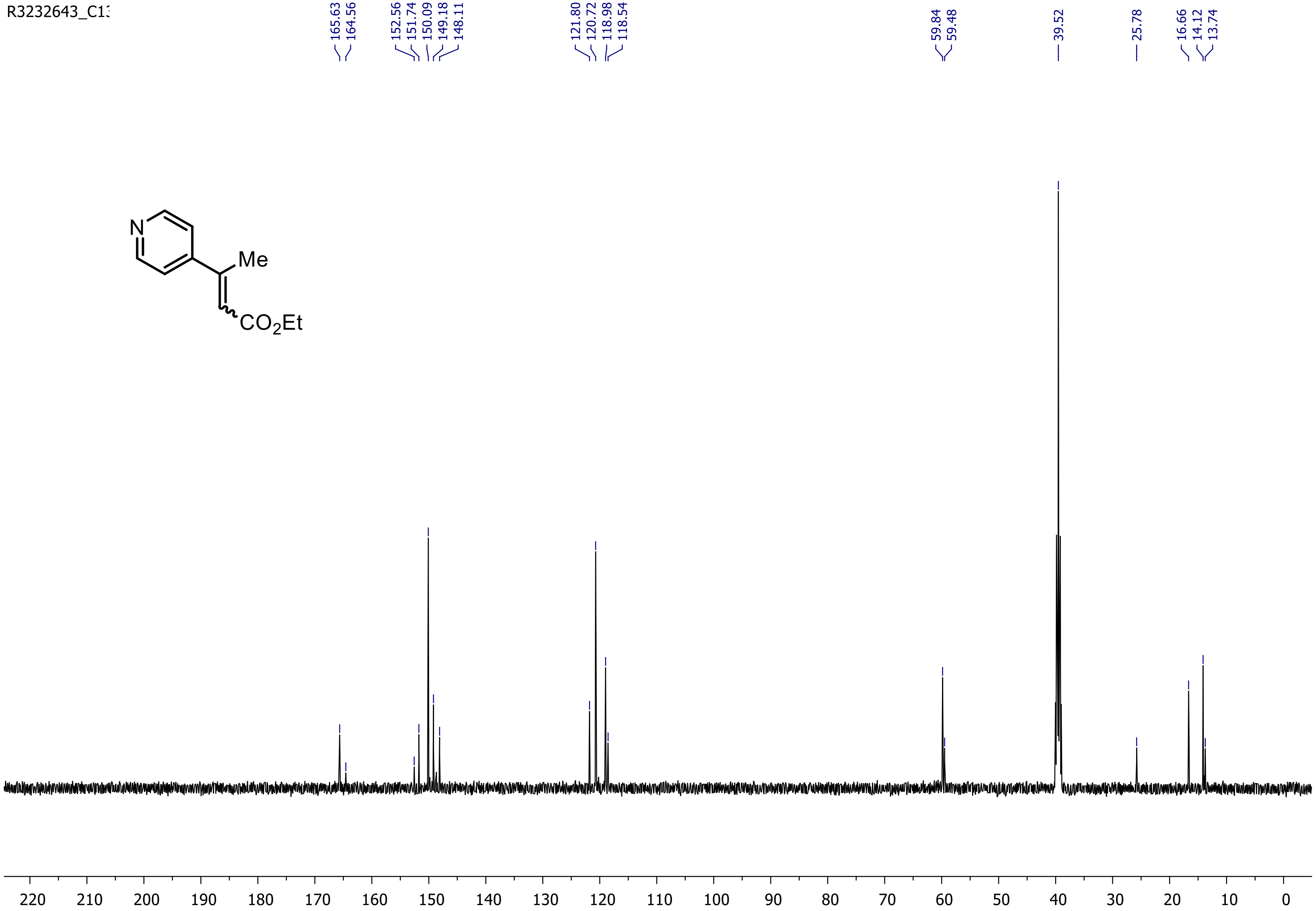

Compound 21

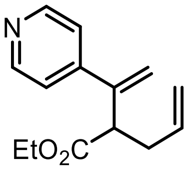

^{1}H NMR (500 MHz, $CDCl_3$)

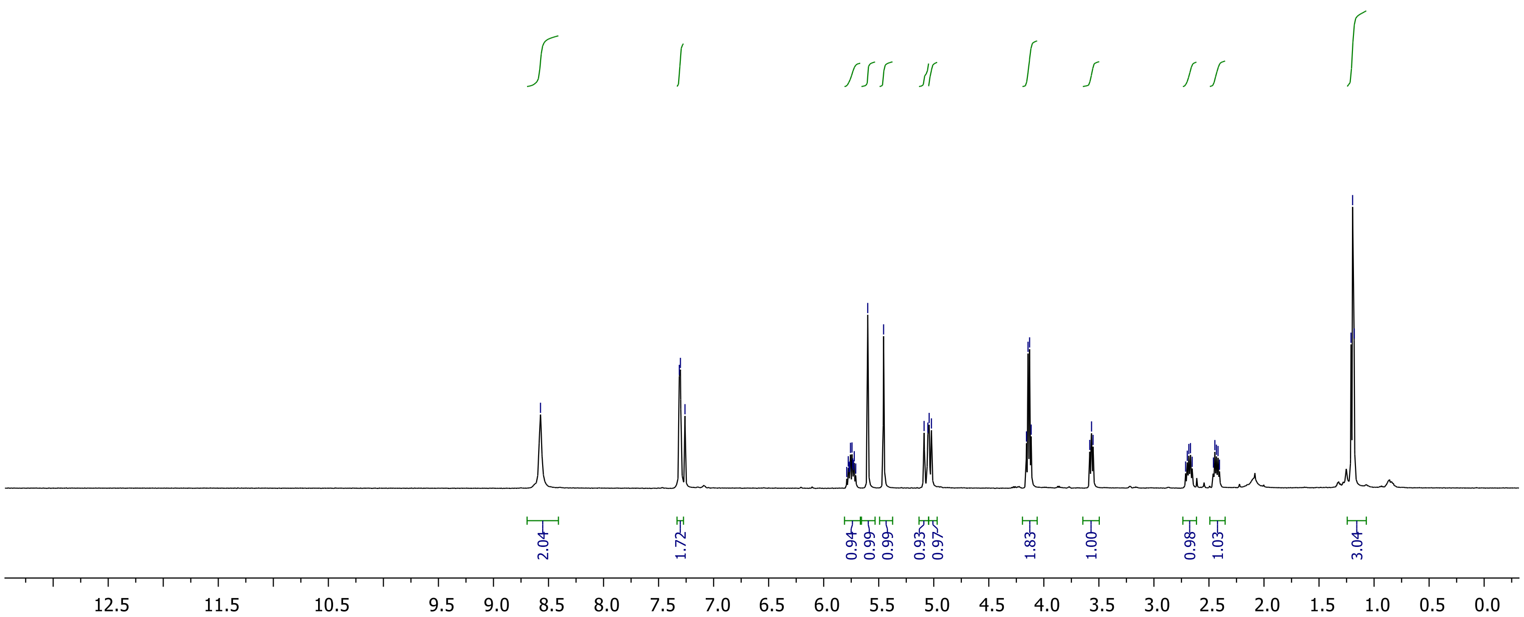

$^{13}C\{^{1}H\}$ NMR (151 MHz, CDCl$_3$)

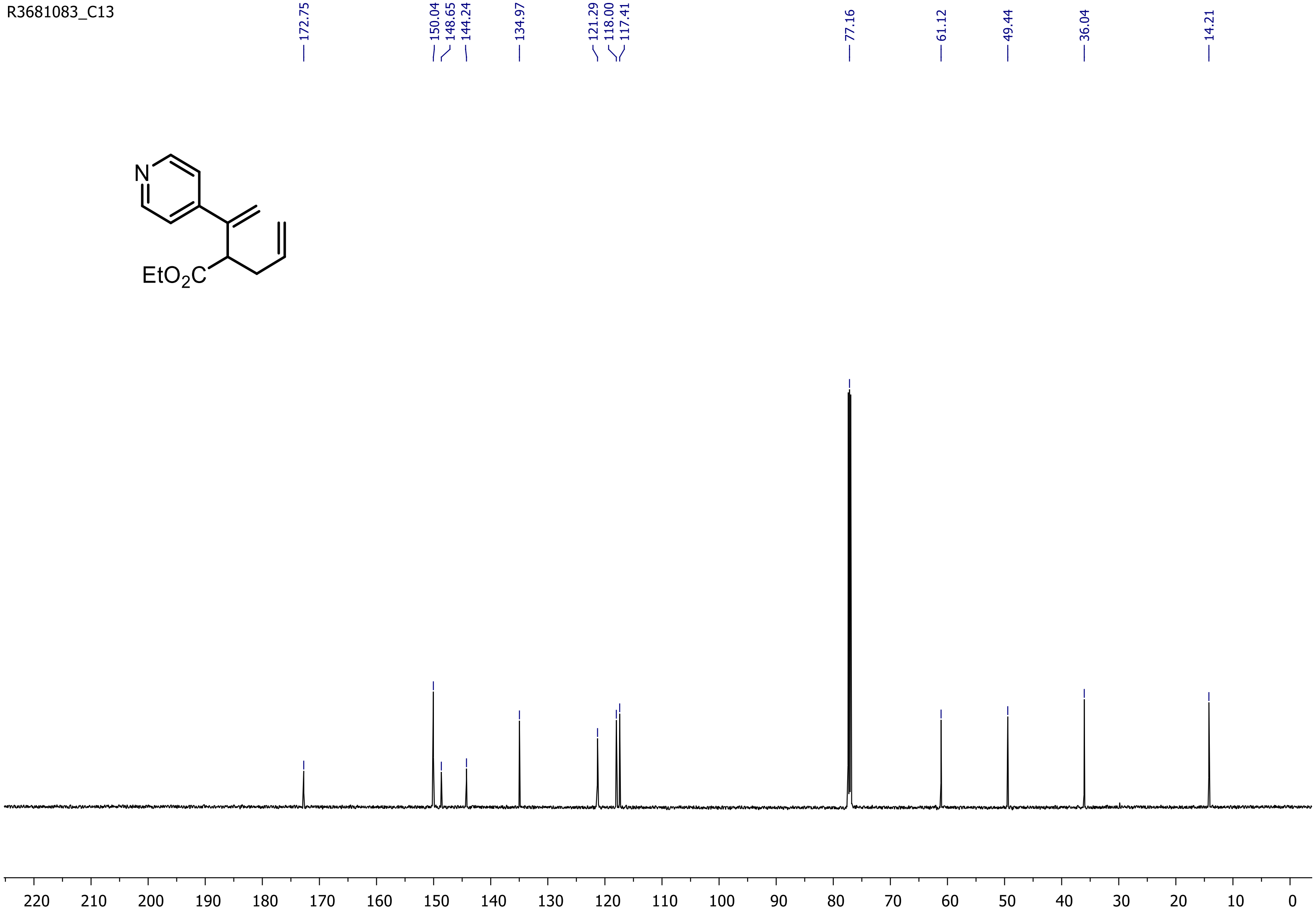

Compound (±)-21a

^{1}H NMR (500 MHz, $CDCl_3$)

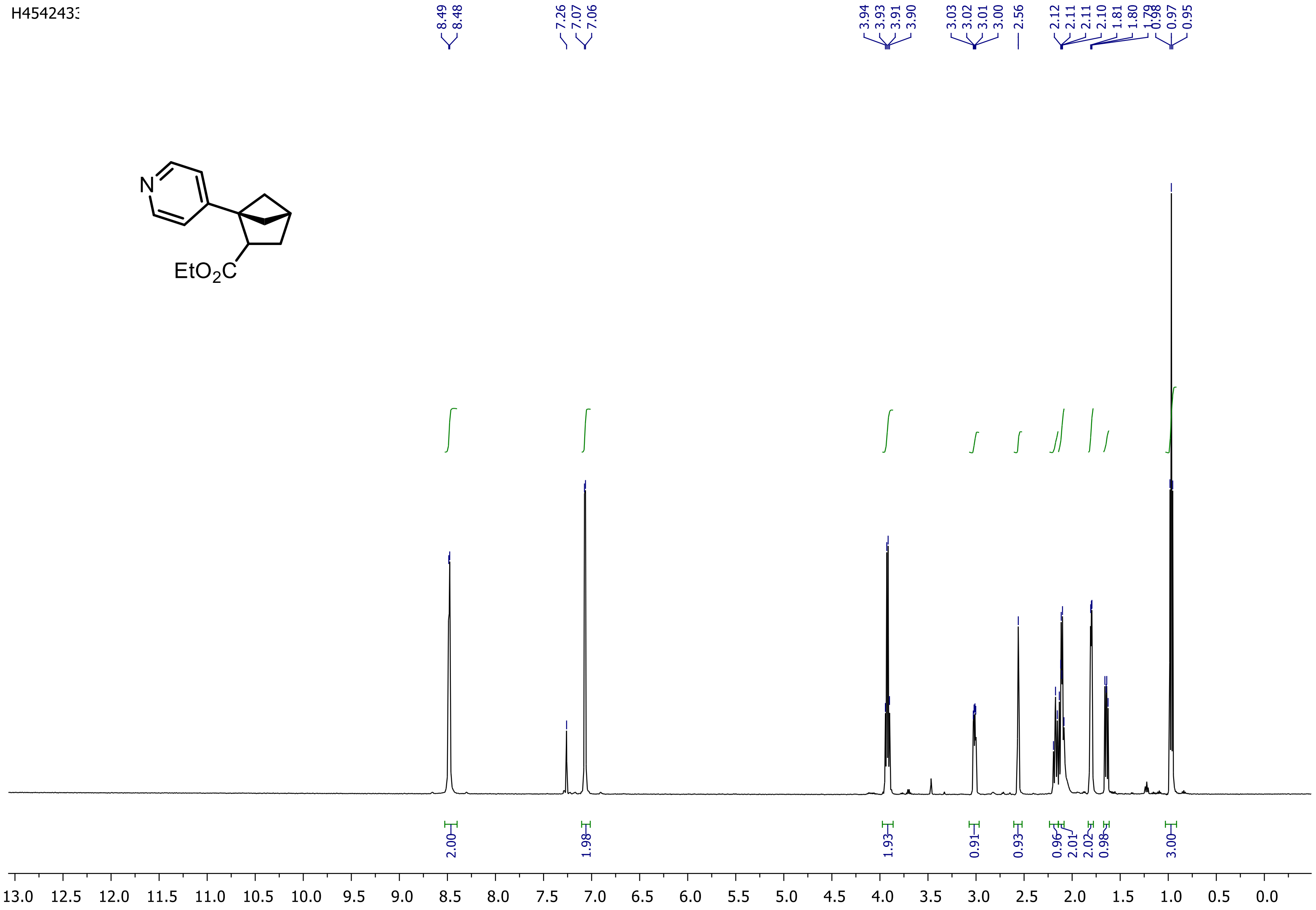

$^{13}C\{^{1}H\}$ NMR (126 MHz, $CDCl_3$)

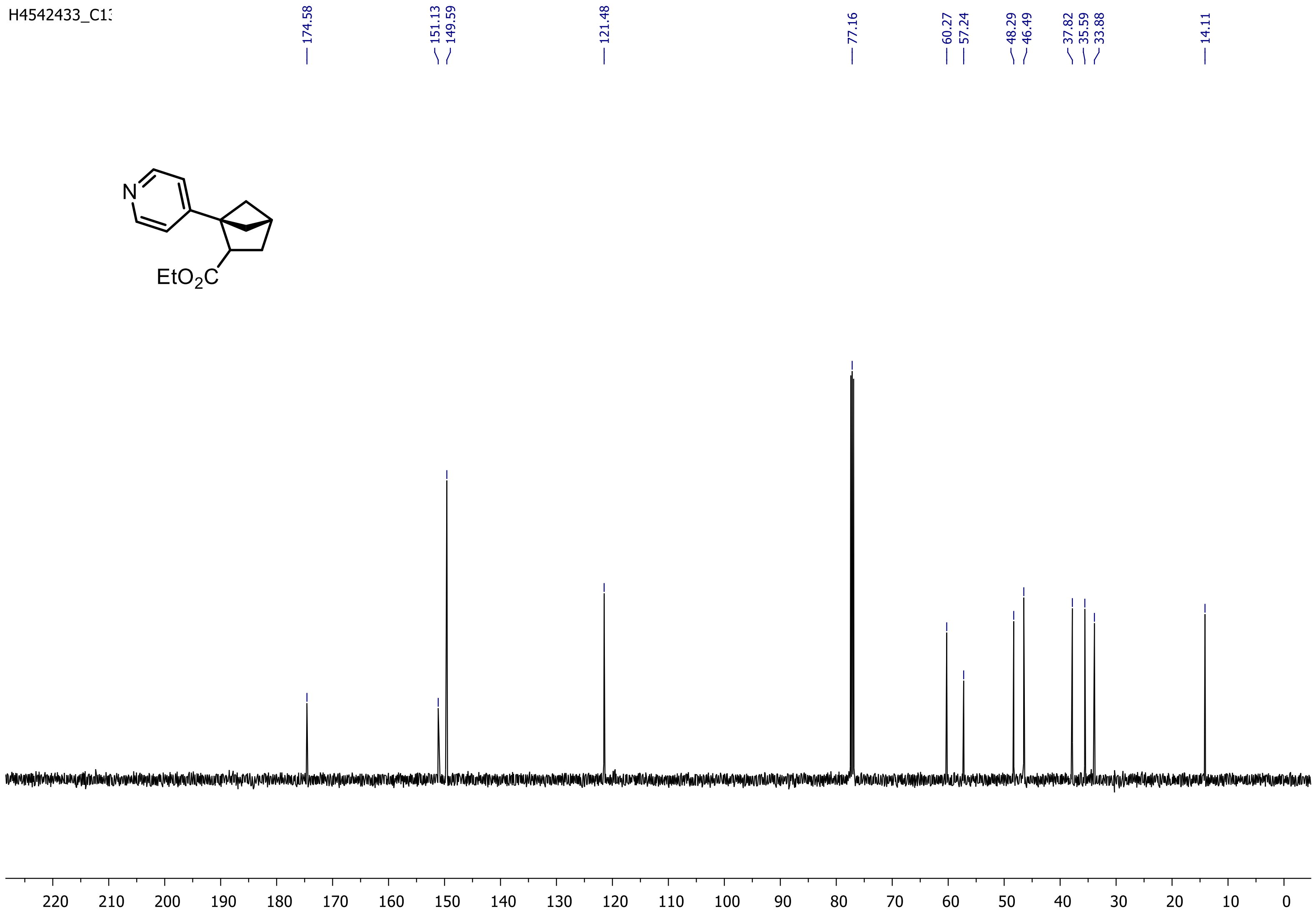

Compound (±)-21b

^{1}H NMR (500 MHz, DMSO-d_6)

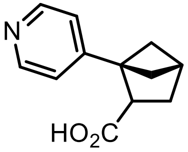

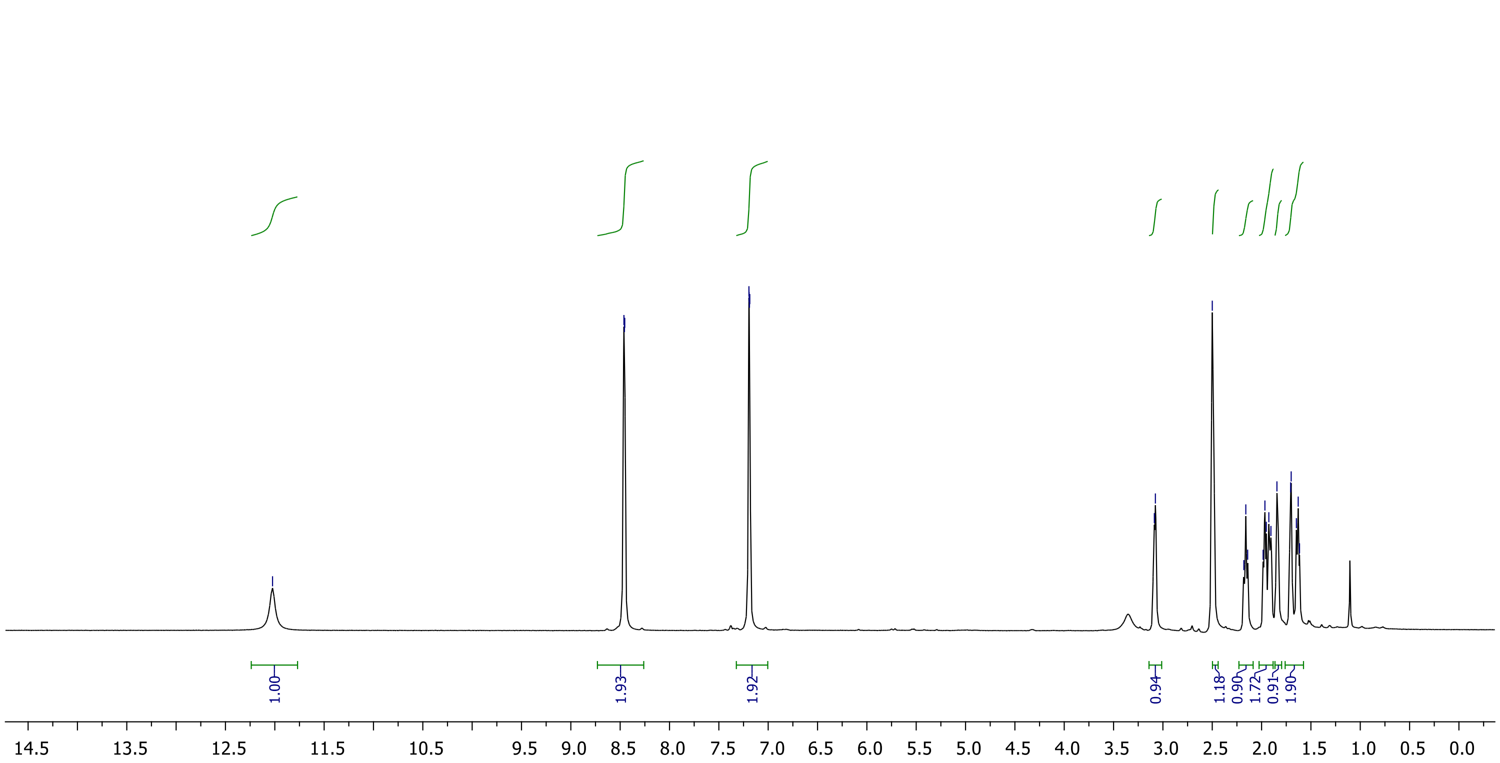

$^{13}C\{^{1}H\}$ NMR (126 MHz, DMSO-d$_6$)

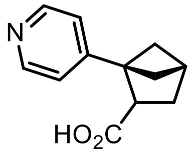

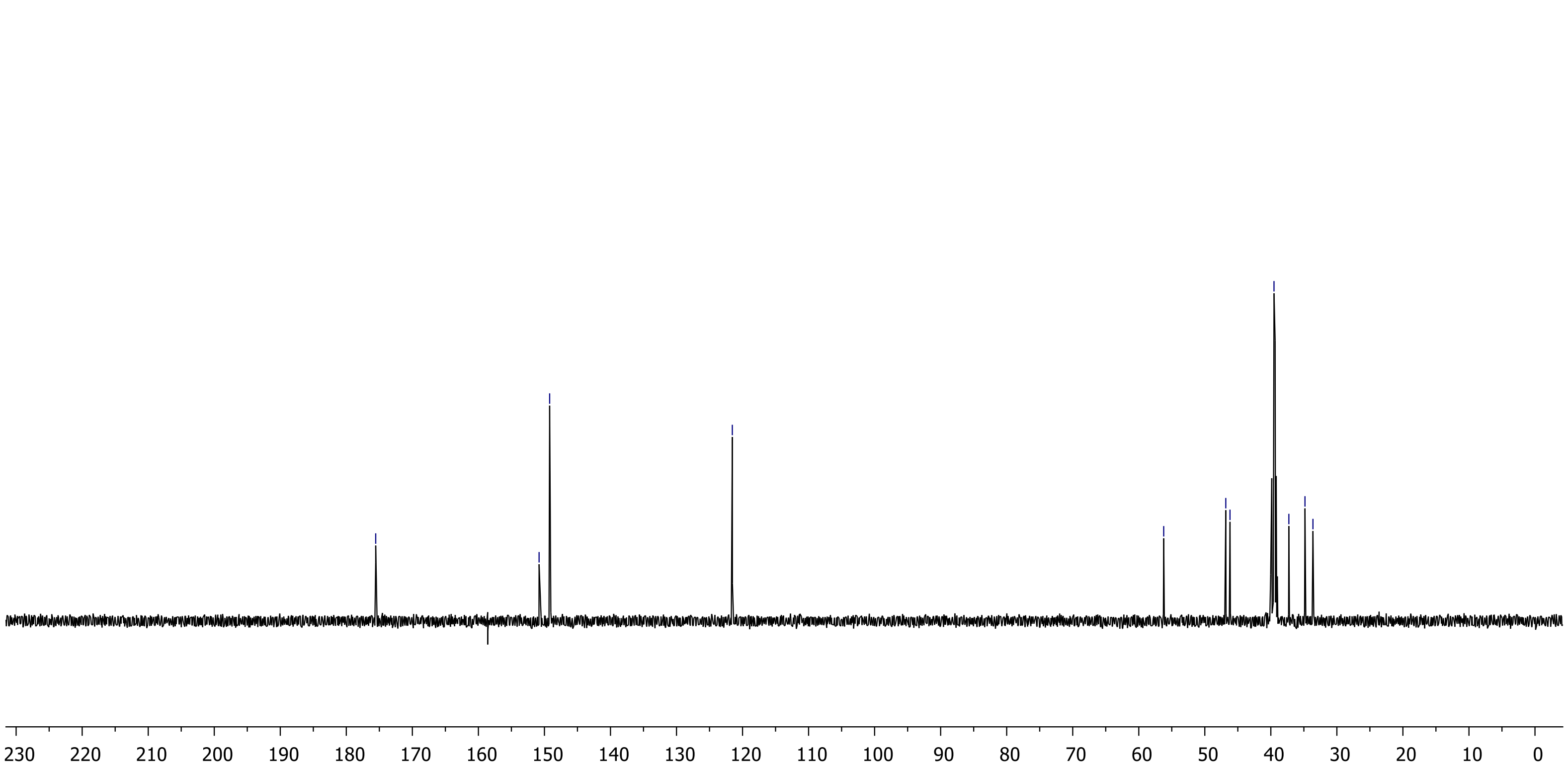

Ethyl-3-(pyridin-3-yl)but-2-enoate

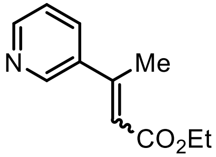

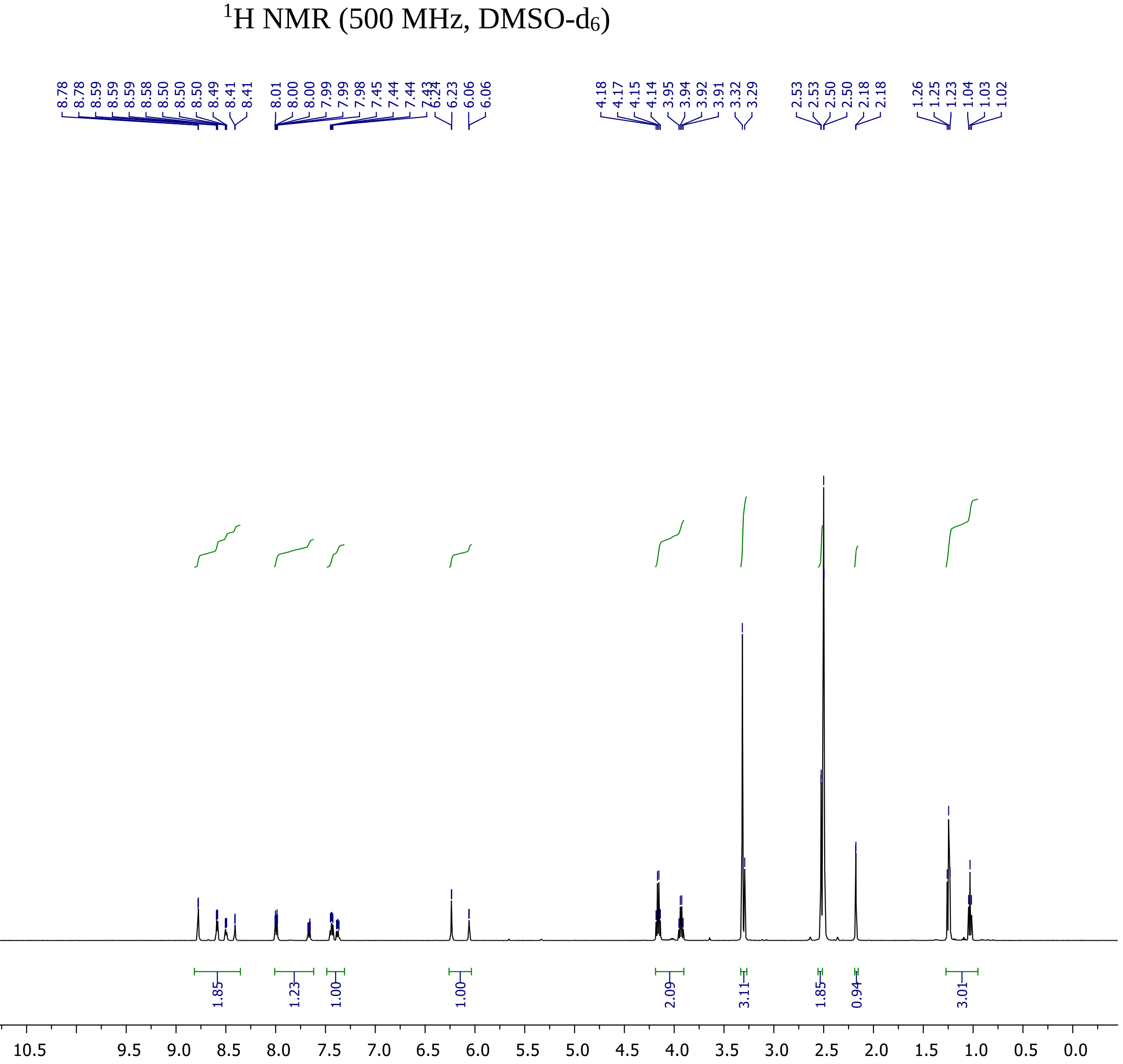

$^{13}C\{^{1}H\}$ NMR (126 MHz, $CDCl_3$)

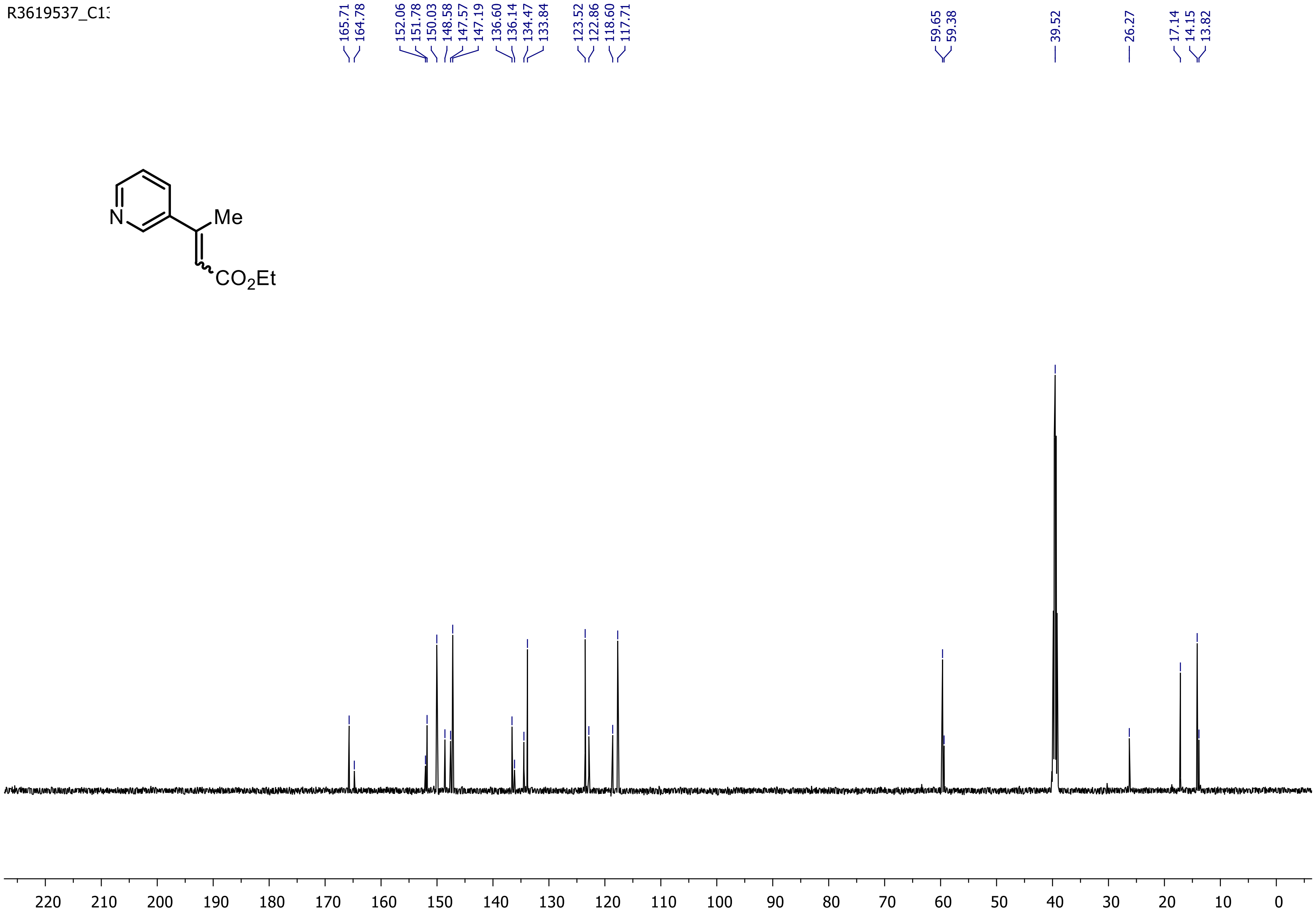

Compound 22

^{1}H NMR (500 MHz, DMSO-d$_6$)

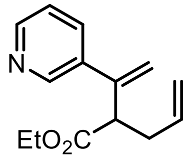

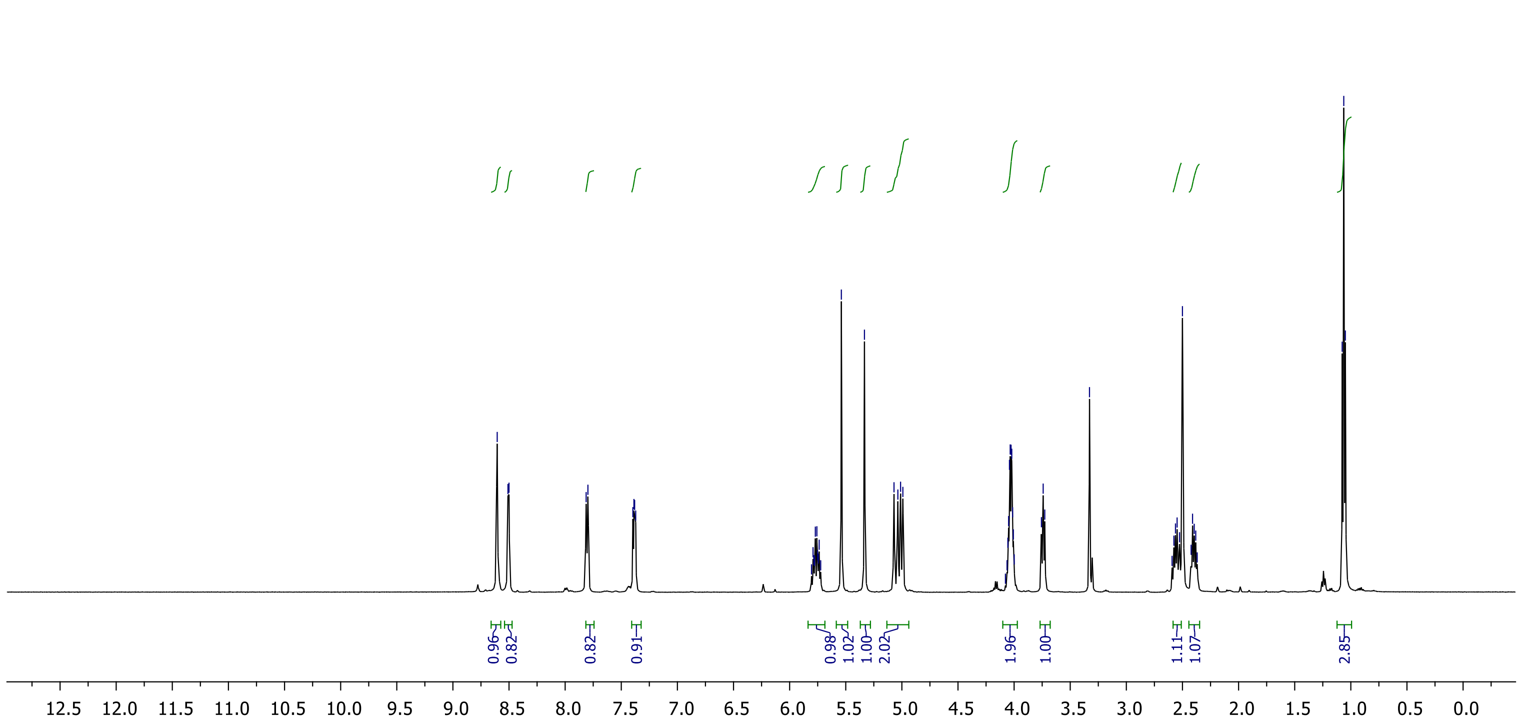

$^{13}C\{^{1}H\}$ NMR (126 MHz, DMSO-d_6)

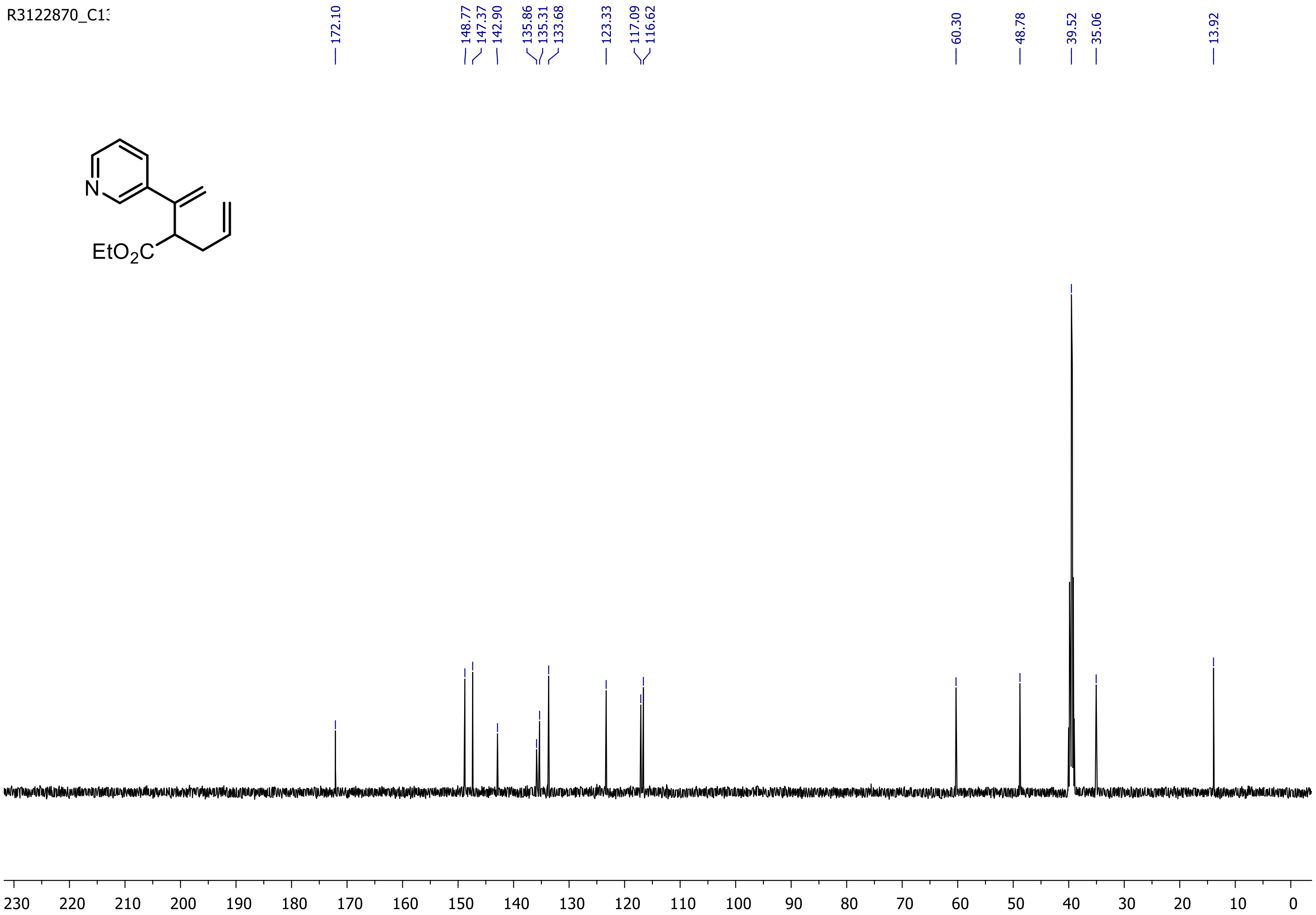

Compound (±)-22a

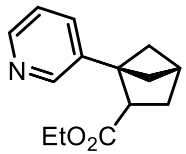

^{1}H NMR (500 MHz, DMSO-d$_6$)

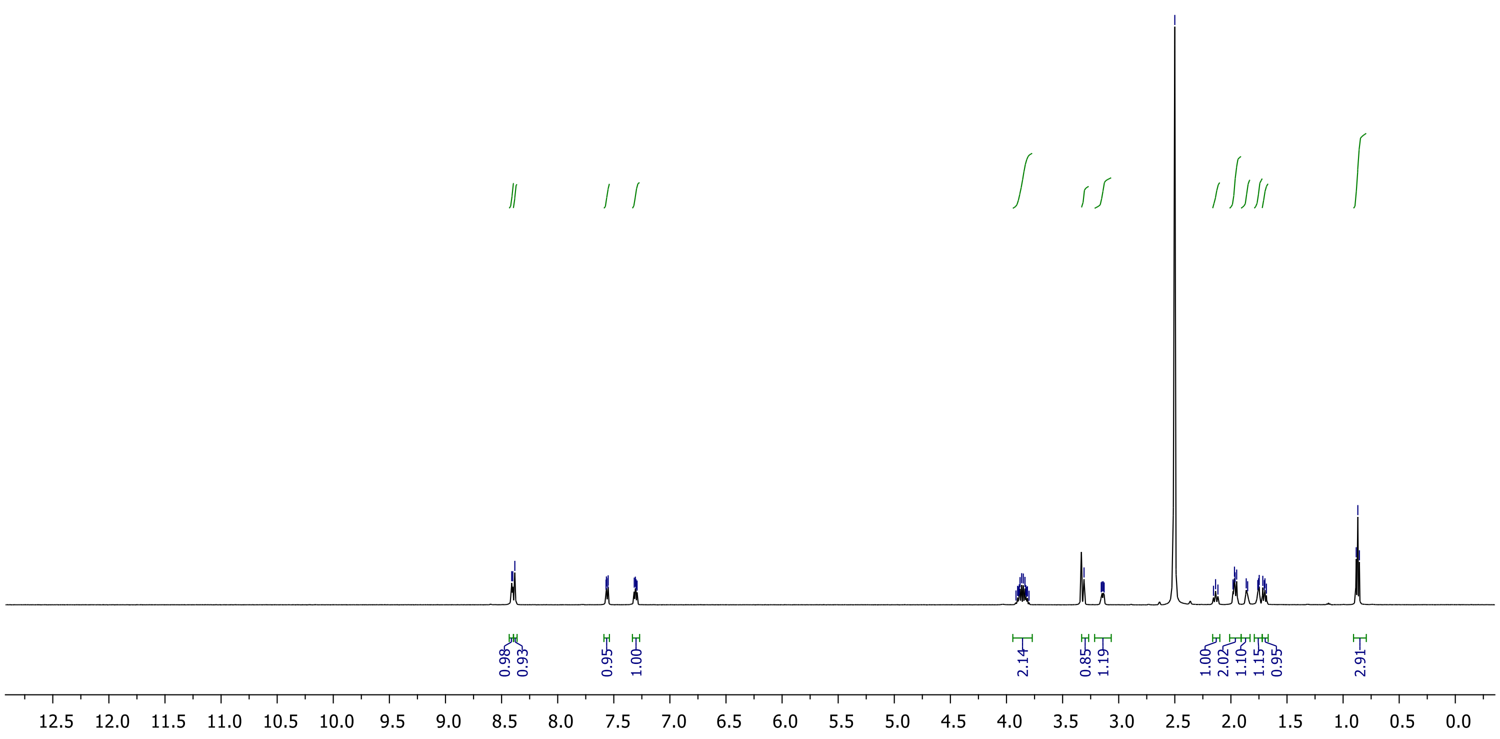

$^{13}C\{^{1}H\}$ NMR (151 MHz, $CDCl_3$)

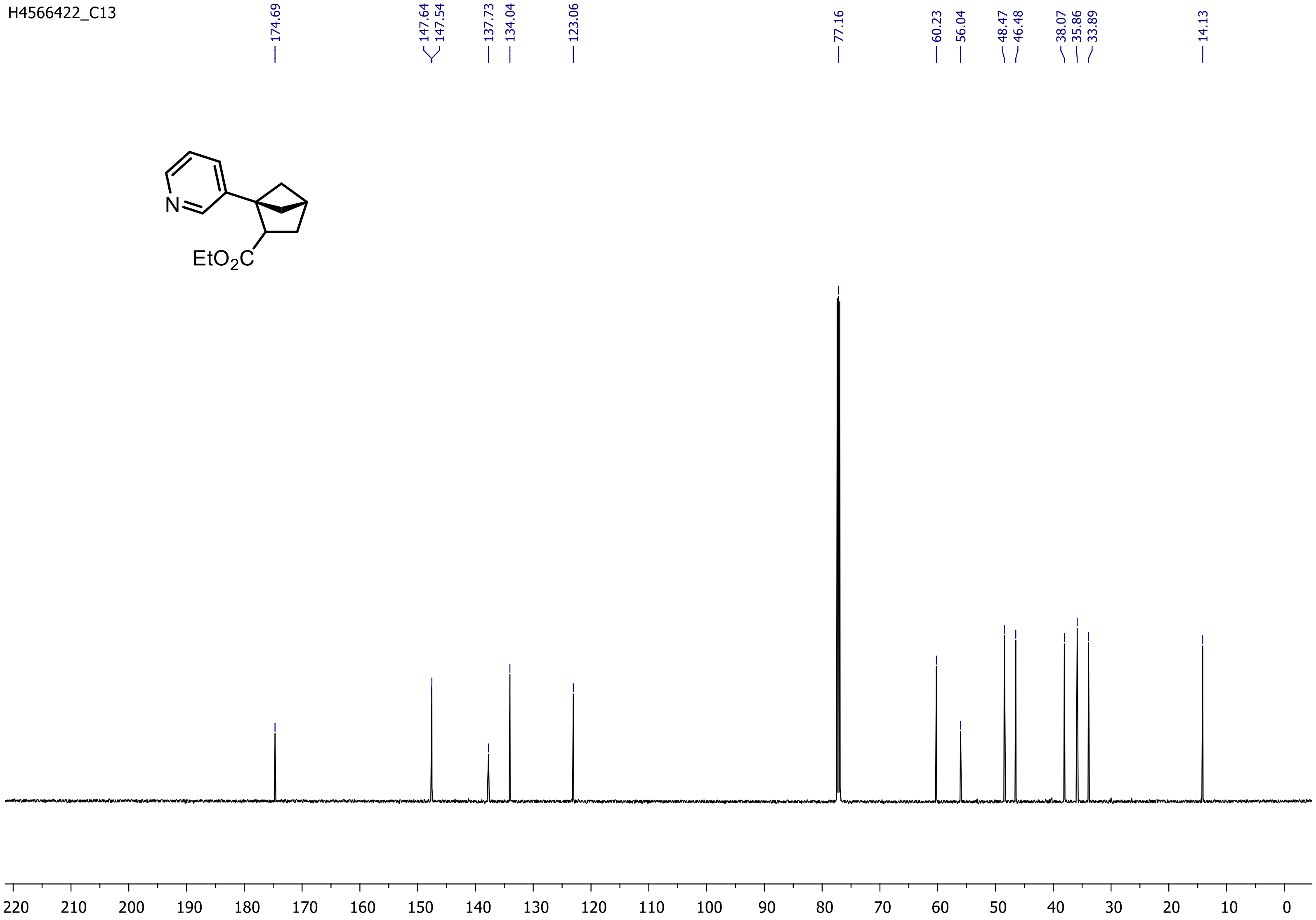

Compound (±)-22b

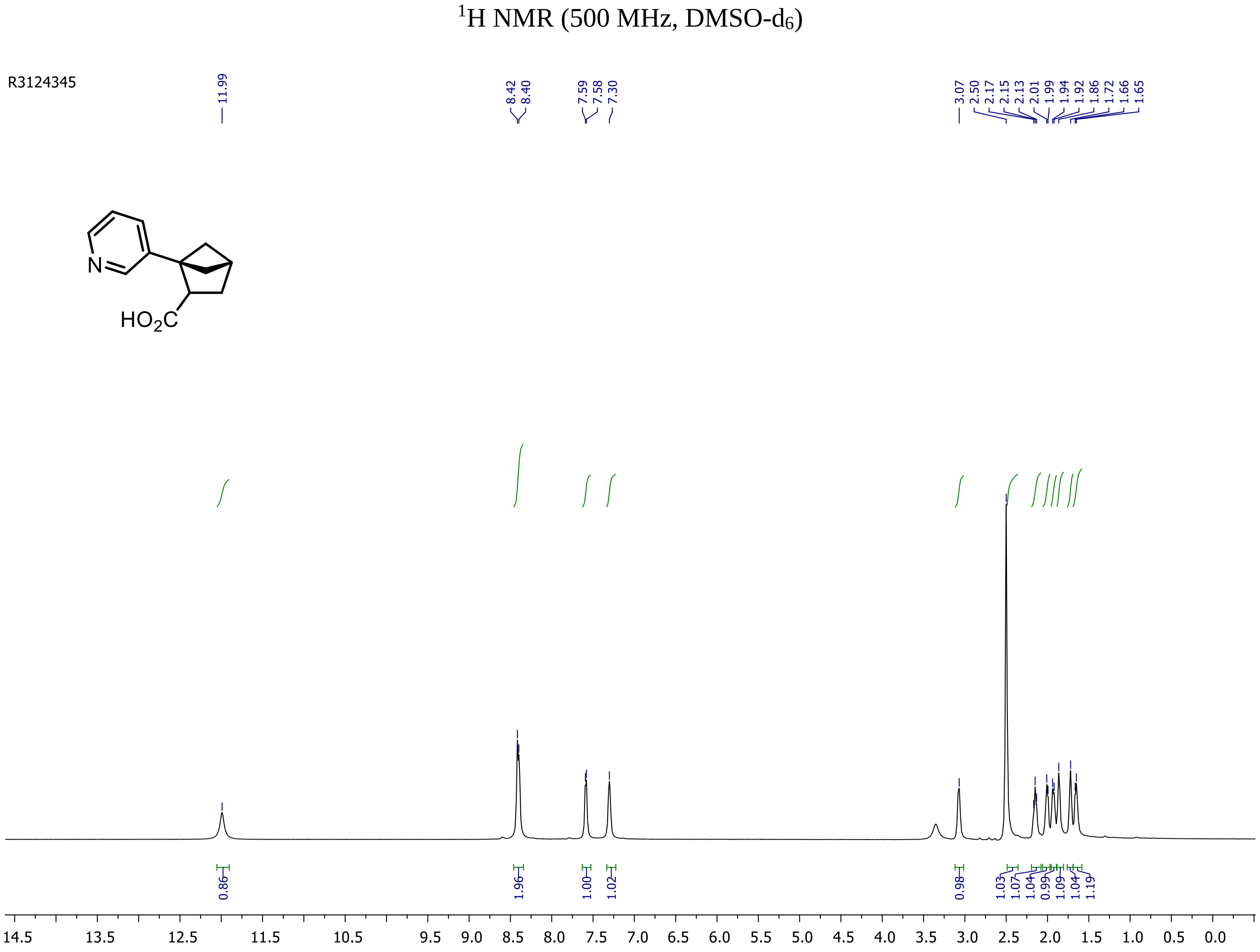

$^{13}C\{^{1}H\}$ NMR (126 MHz, DMSO-d_6)

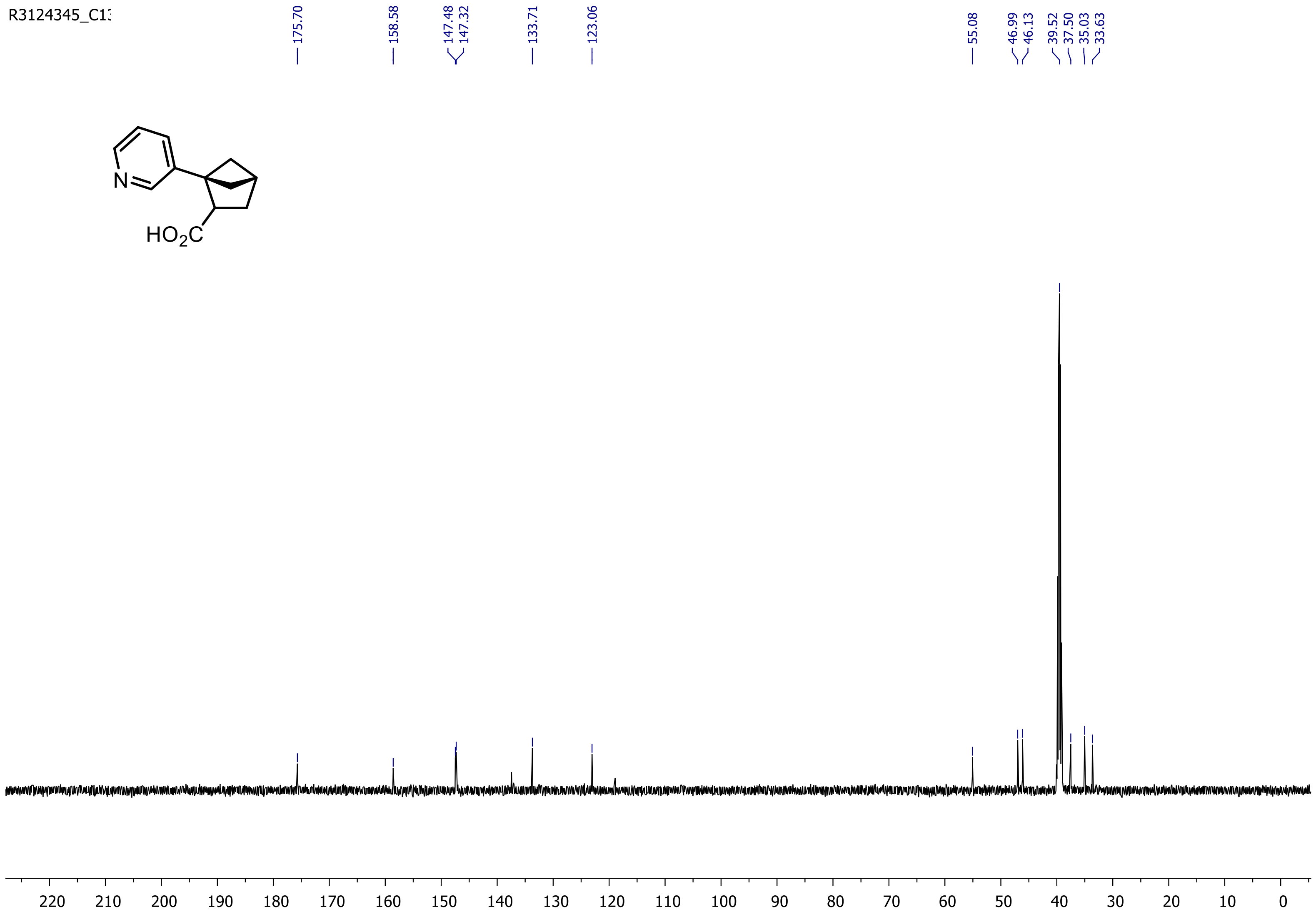

Ethyl-3-(pyridin-2-yl)but-2-enoate

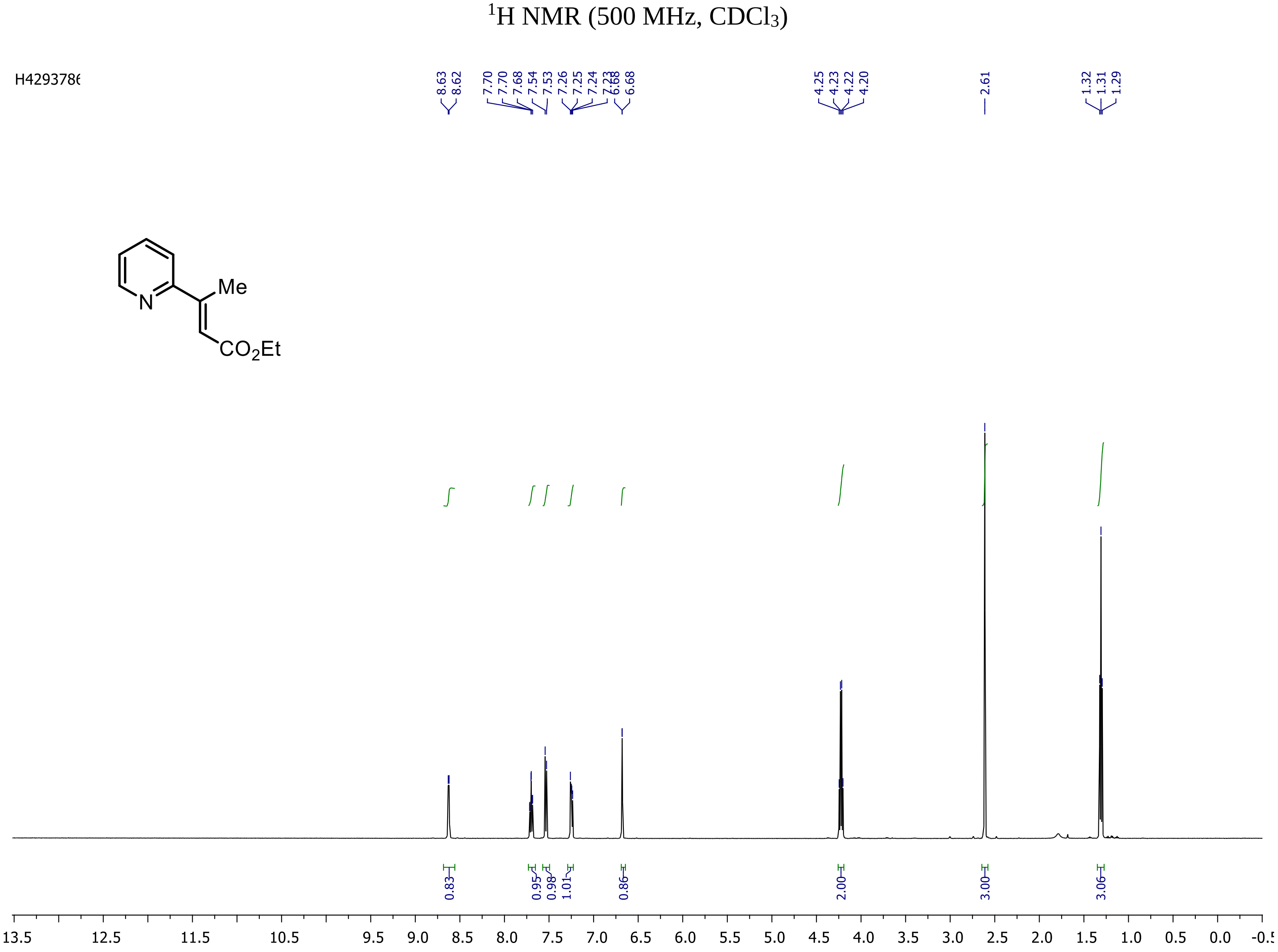

$^{13}C\{^{1}H\}$ NMR (126 MHz, $CDCl_3$)

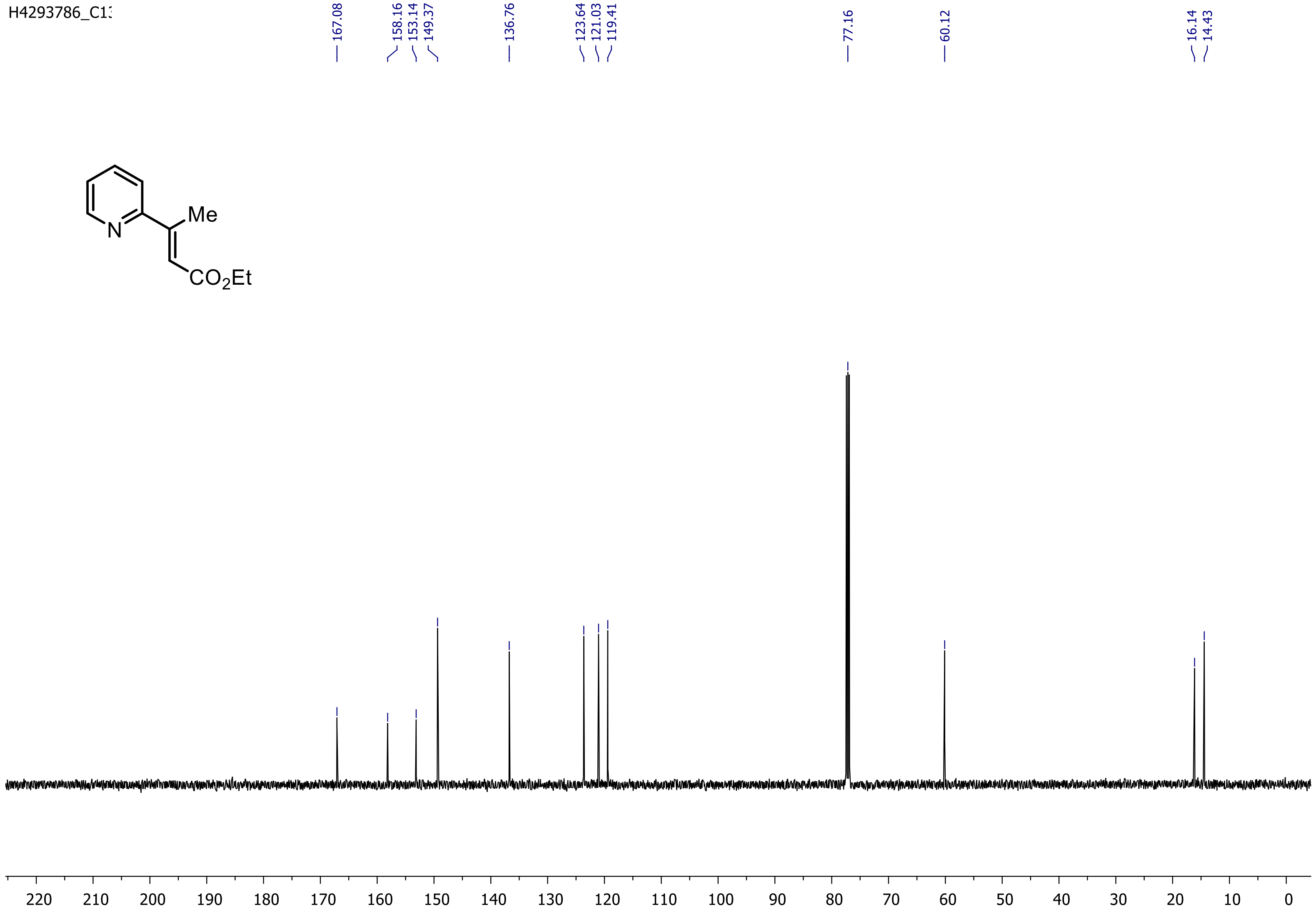

Compound 23

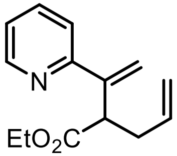

N
EtO2C

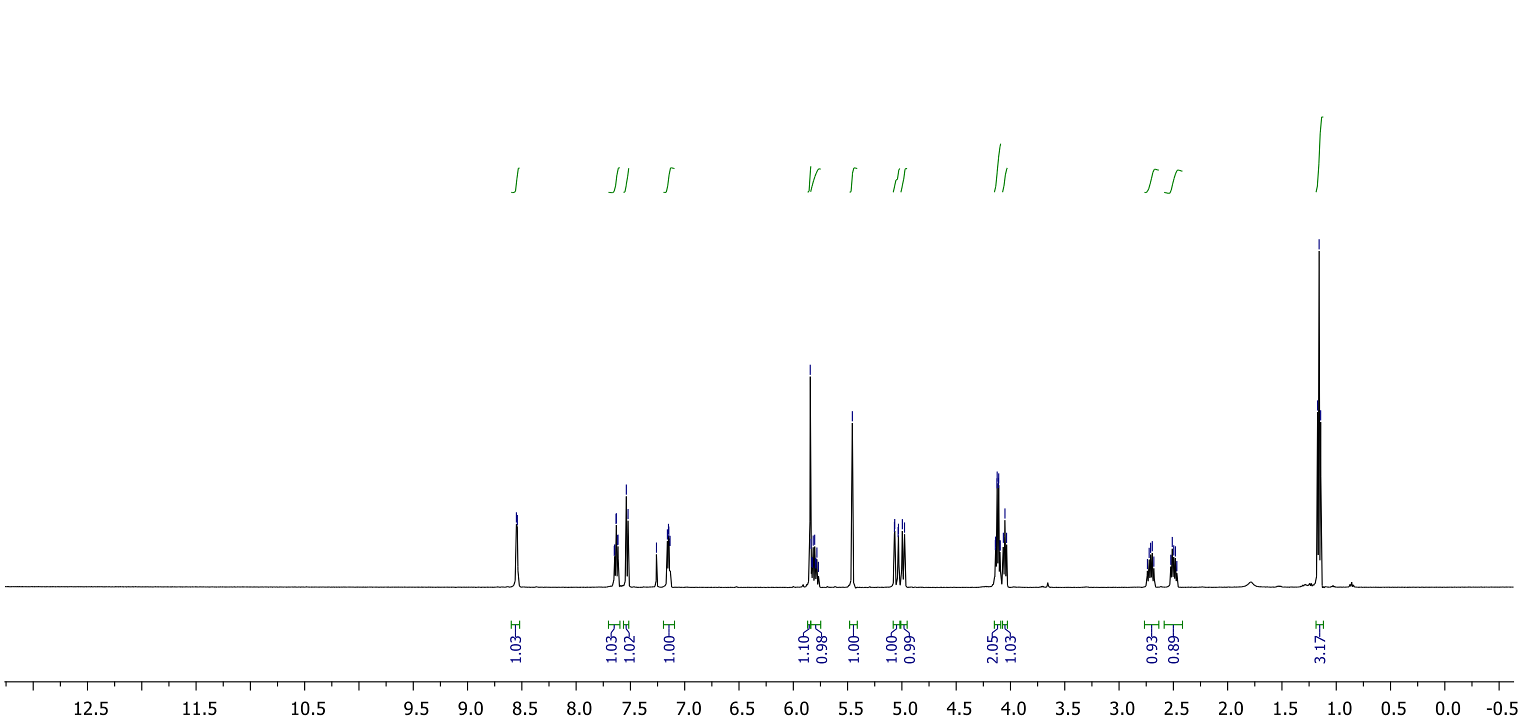

^{1}H NMR (500 MHz, CDCl$_3$)
H4273873
8.55 8.54 7.63 7.63 7.62 7.61 7.54 7.52 7.26 7.16 7.15 7.15 7.13 5.84 5.84 5.83 5.82 5.80 5.78 5.46 5.07 5.07 5.00 4.98 4.13 4.12 4.11 4.11 4.07 4.05 2.72 2.71 2.69 2.68 2.52 2.51 2.50 2.48 2.47 1.17 1.16 1.14
1.03 1.03 1.02 1.00 1.10 0.98 1.00 1.00 0.99 2.05 1.03 0.93 0.89 3.17
12.5 11.5 10.5 9.5 9.0 8.5 8.0 7.5 7.0 6.5 6.0 5.5 5.0 4.5 4.0 3.5 3.0 2.5 2.0 1.5 1.0 0.5 0.0 -0.5

$^{13}C\{^{1}H\}$ NMR (126 MHz, $CDCl_3$)

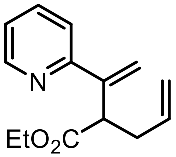

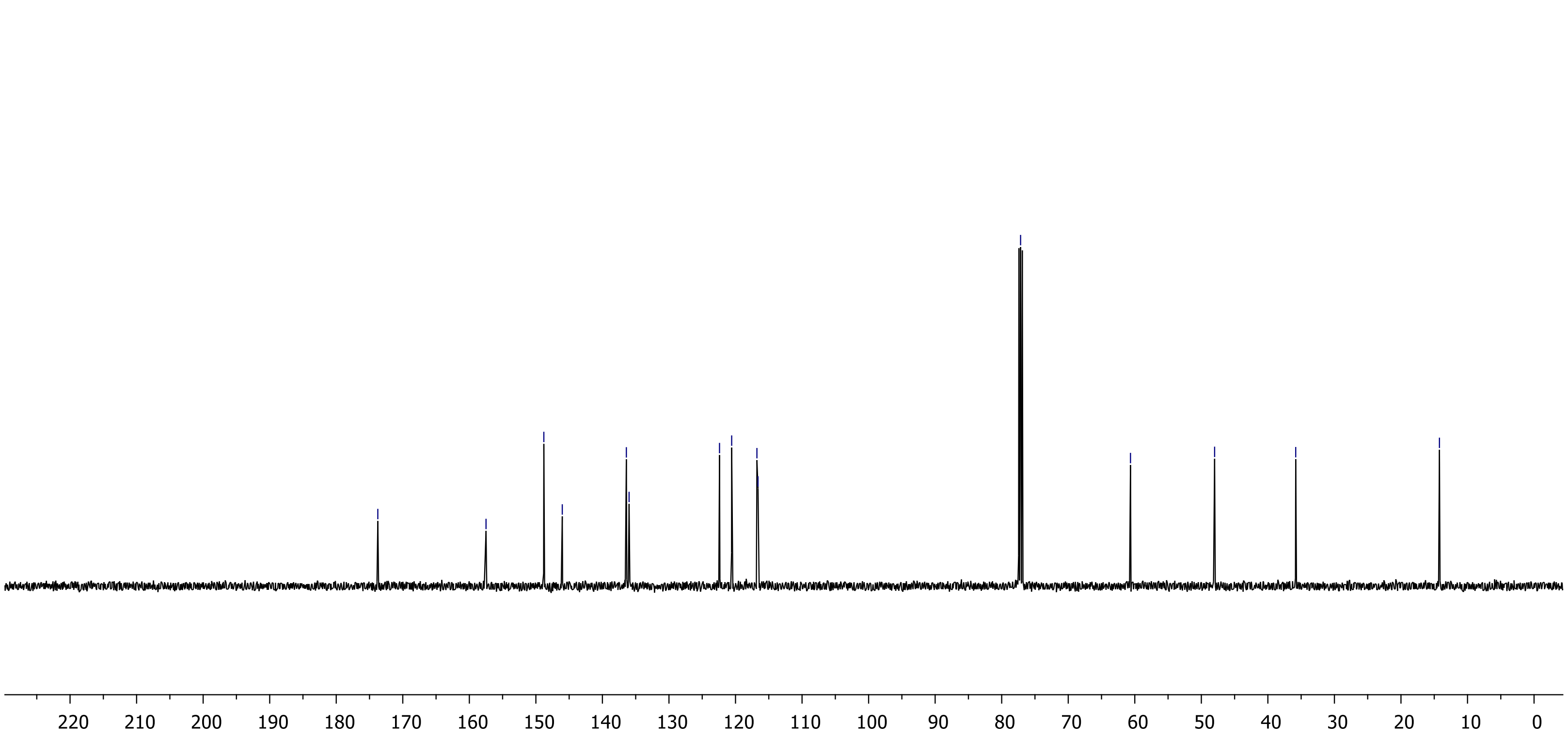

Compound (±)-23a

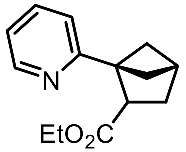

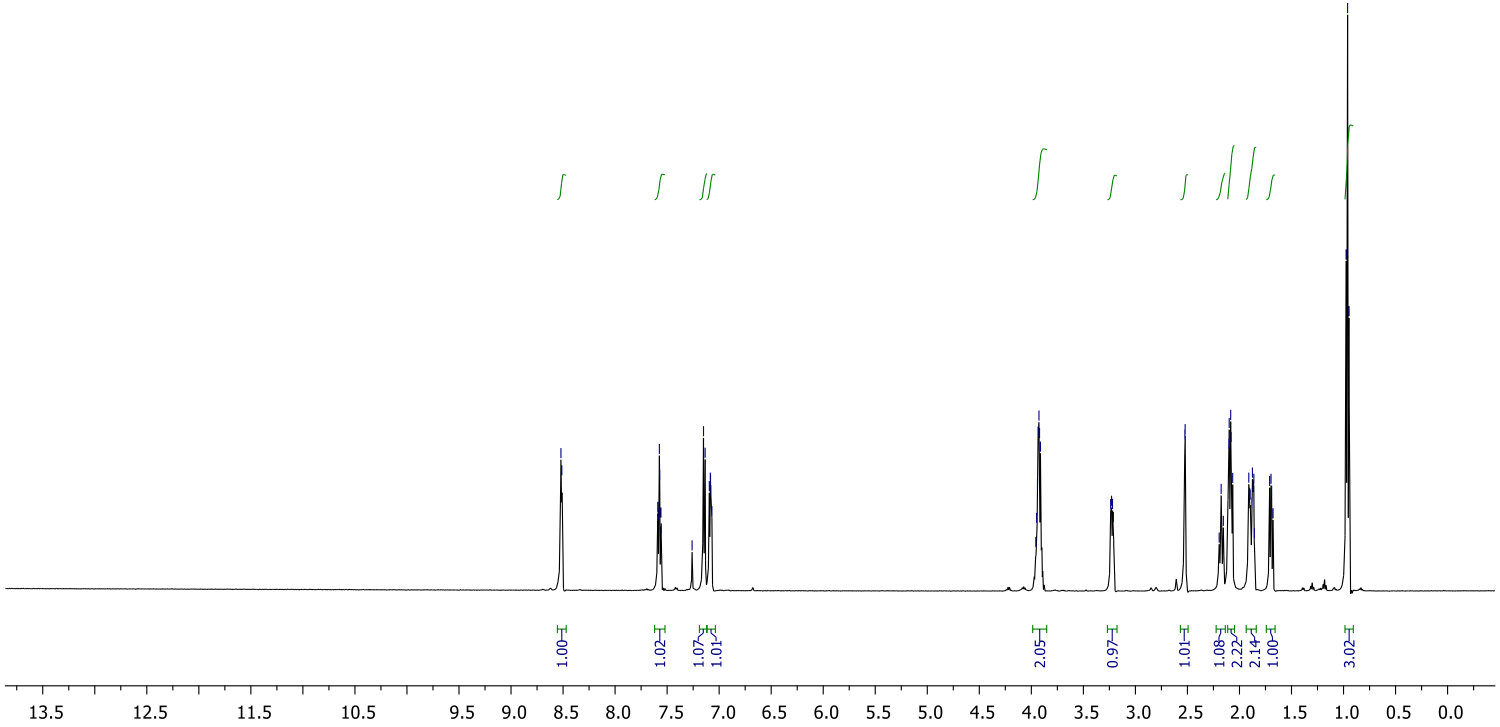

$^{13}C\{^{1}H\}$ NMR (126 MHz, $CDCl_3$)

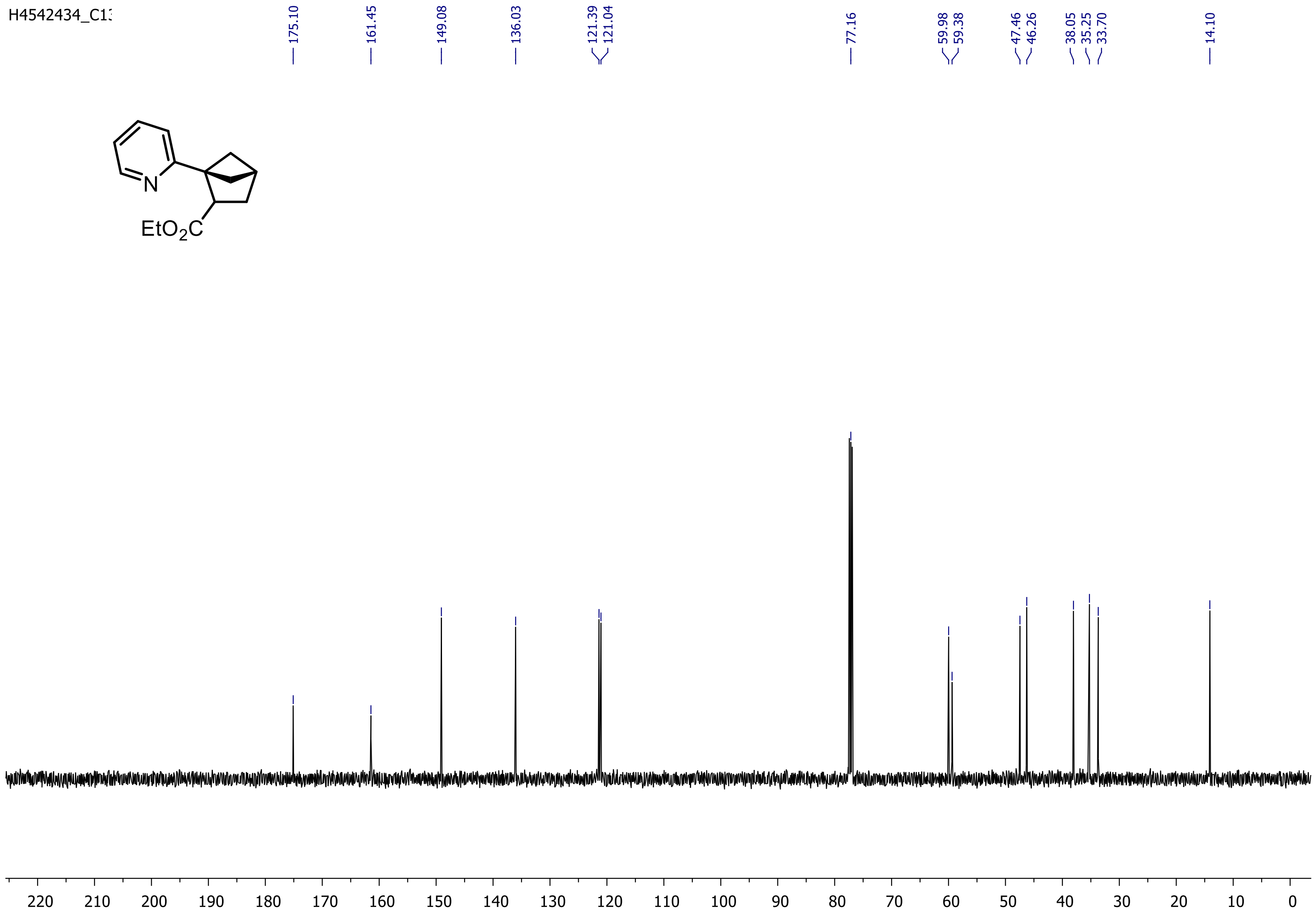

Compound (±)-23b

^{1}H NMR (500 MHz, DMSO-d_6)

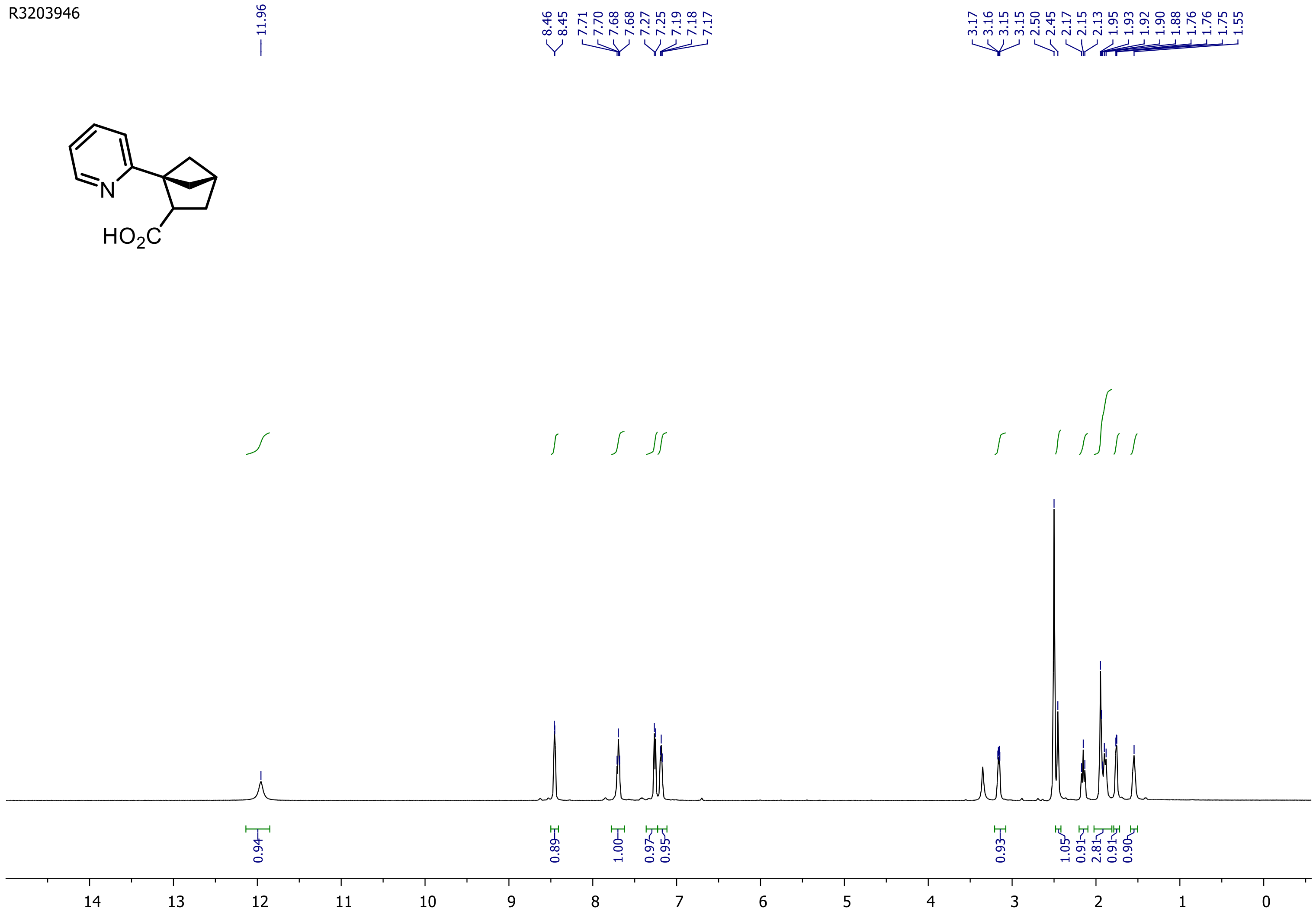

$^{13}C\{^{1}H\}$ NMR (126 MHz, DMSO-d$_6$)

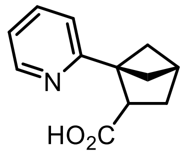

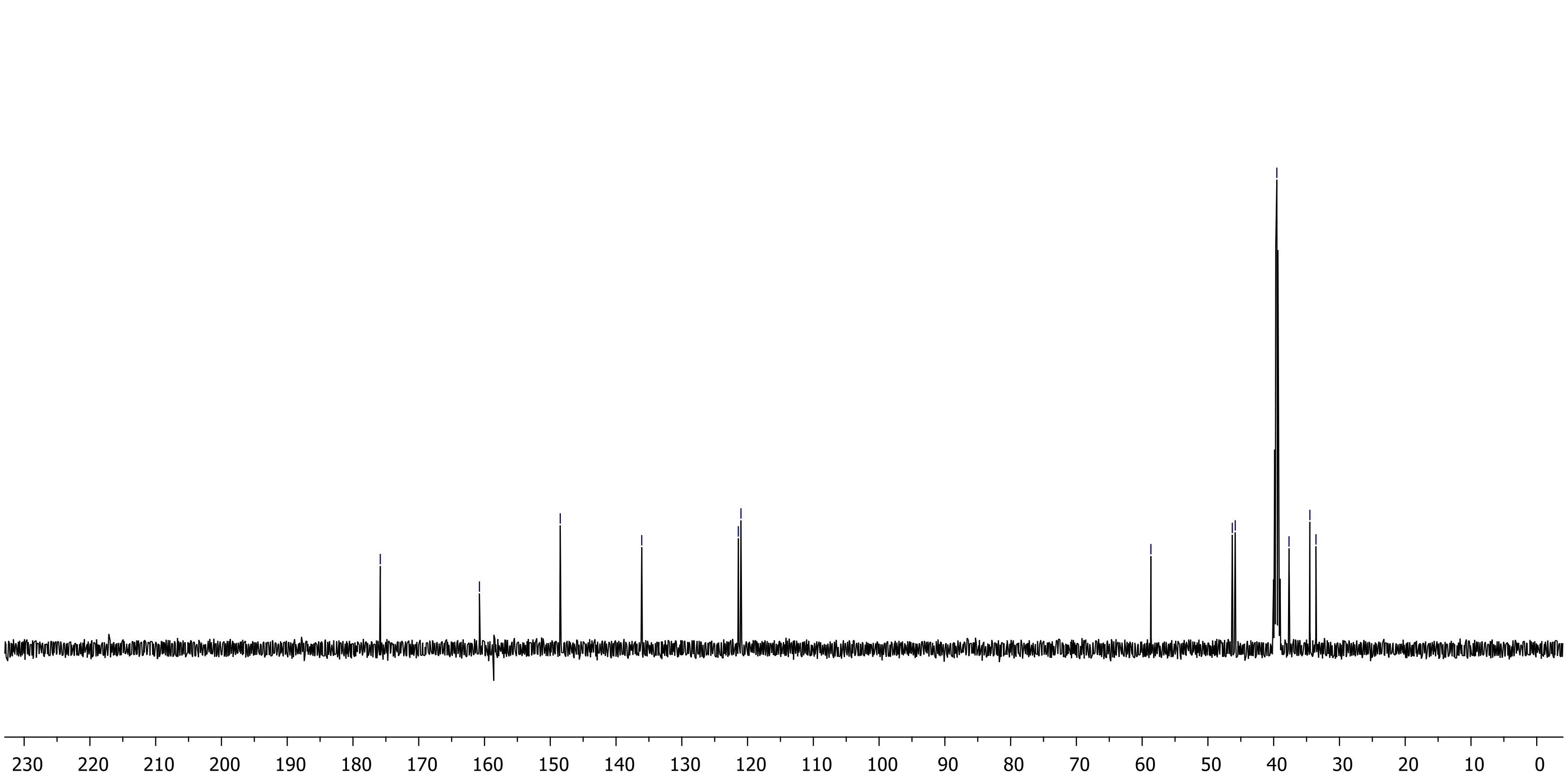

Compound (±)-24

^{1}H NMR (500 MHz, $CDCl_3$)

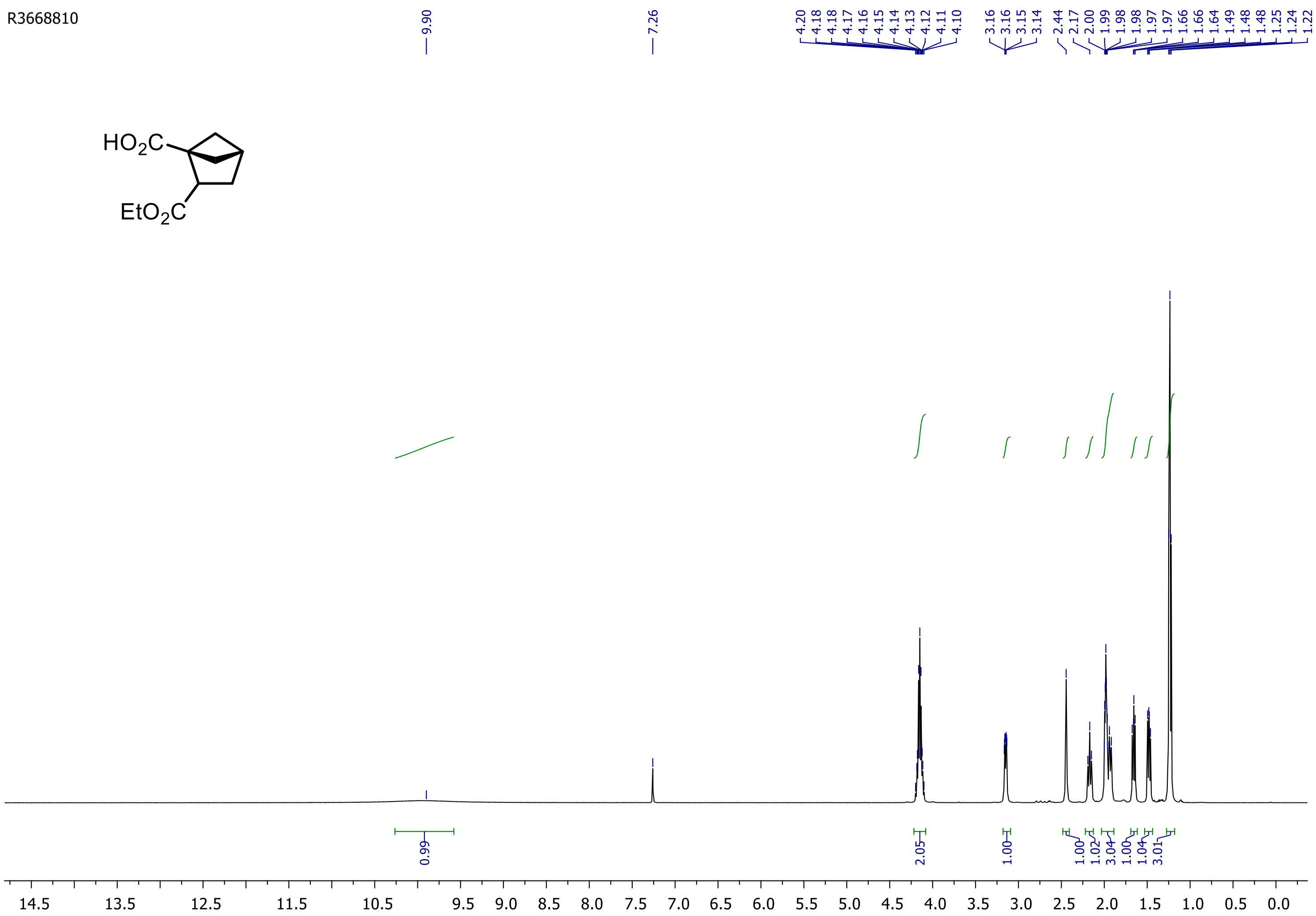

$^{13}C\{^1H\}$ NMR (126 MHz, $CDCl_3$)

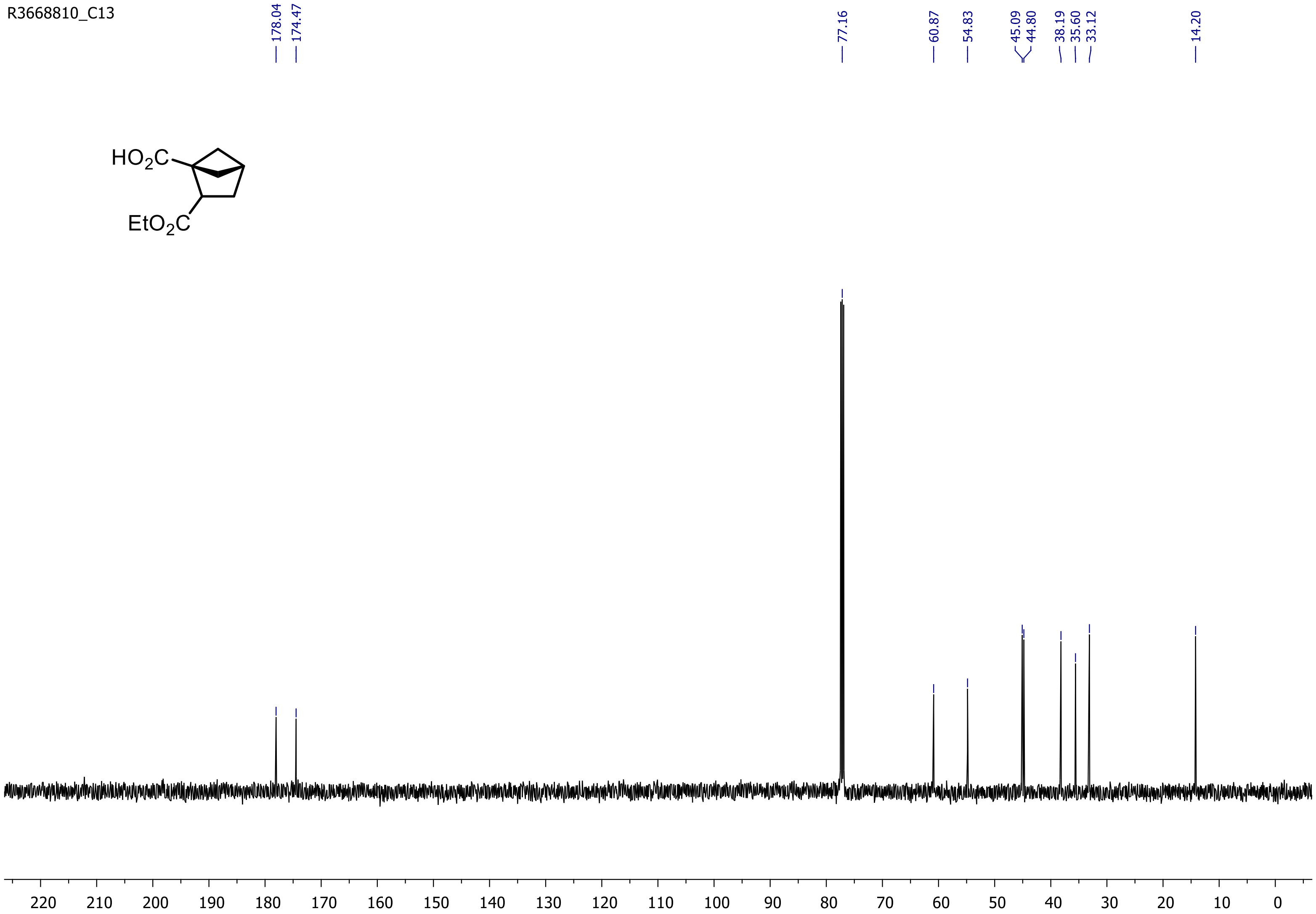

Compound (±)-25

^1H NMR (500 MHz, $CDCl_3$)

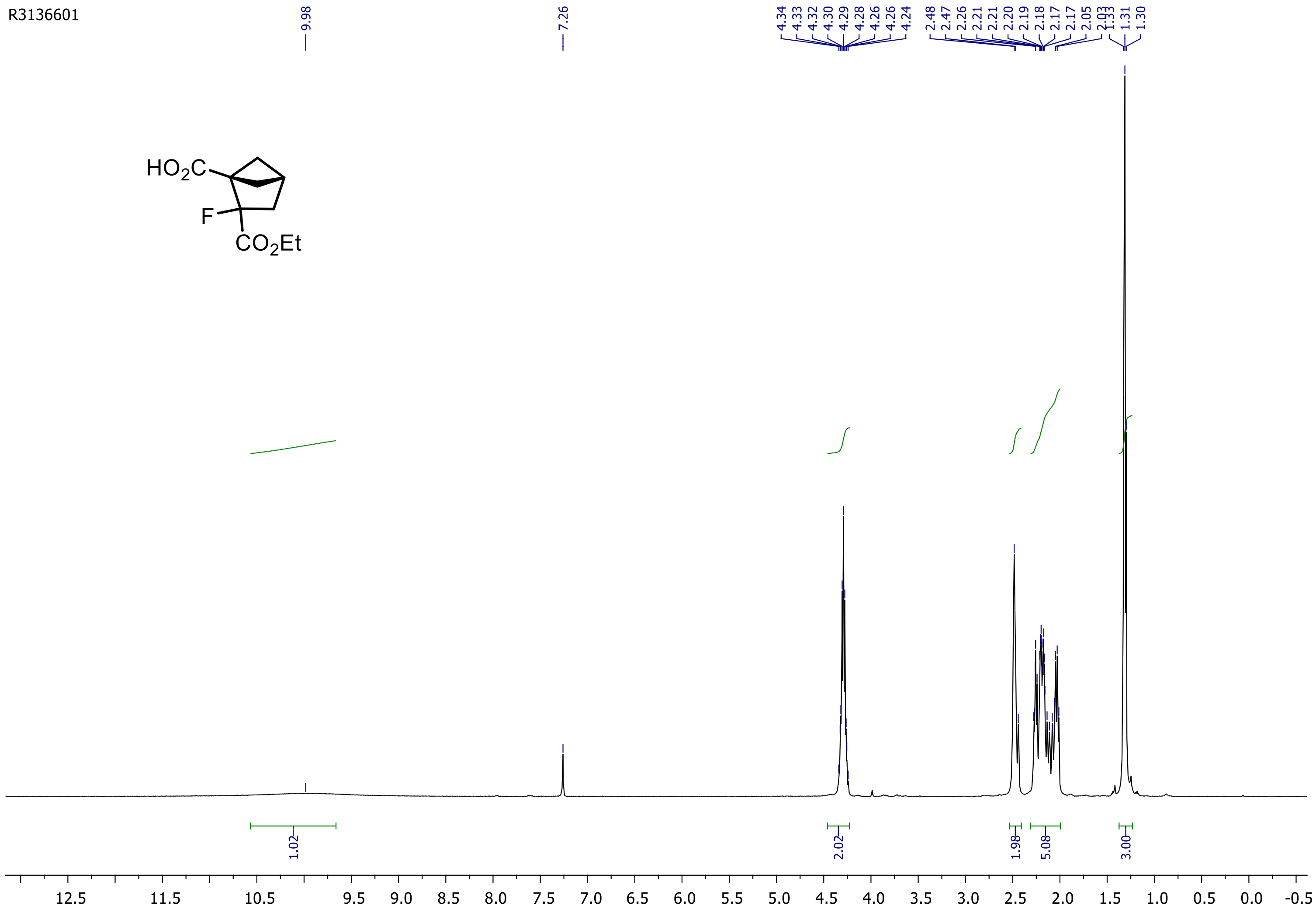

$^{13}C\{^1H\}$ NMR (126 MHz, $CDCl_3$)

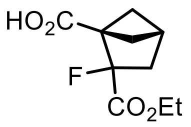

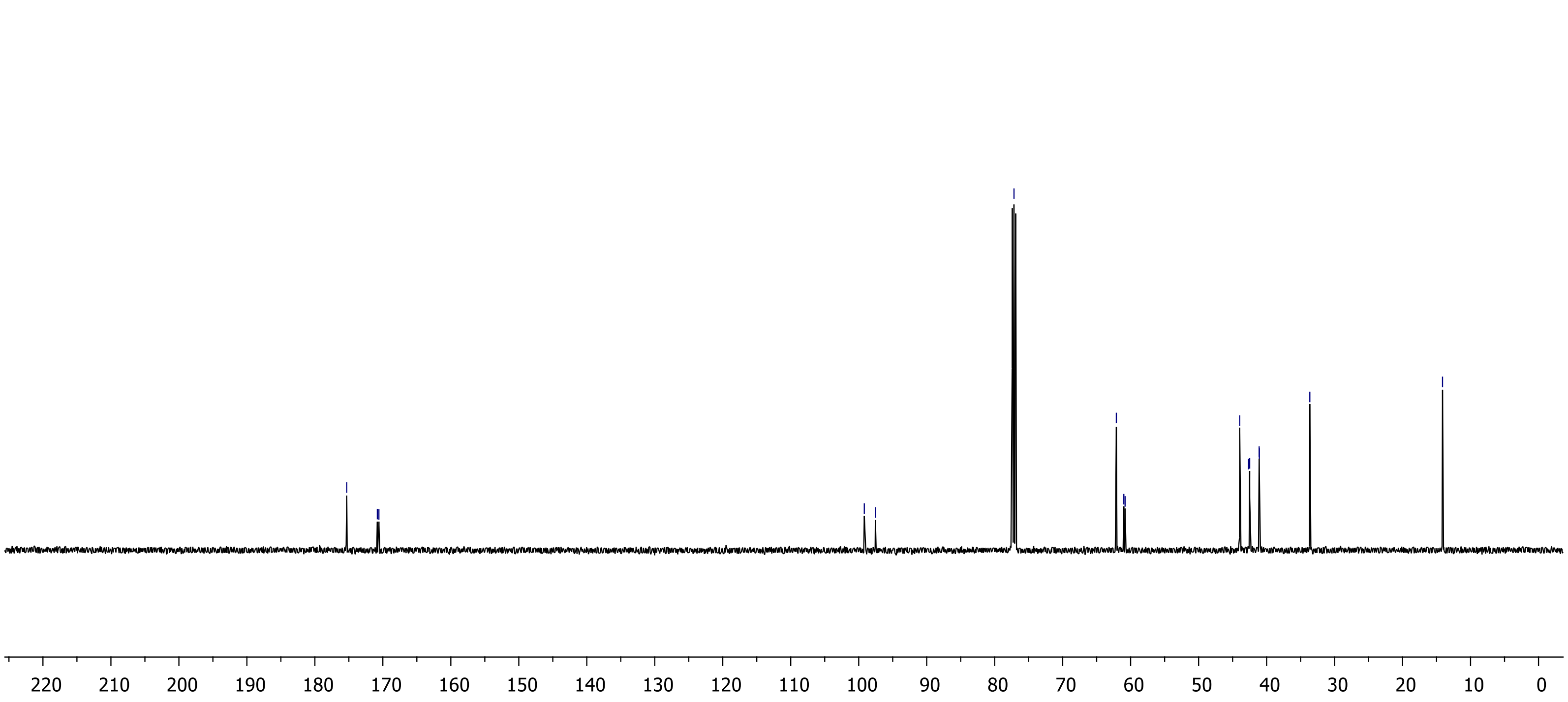

$^{19}F\{^{1}H\}$ NMR (376 MHz, $CDCl_3$)

R3136601_F19{H}

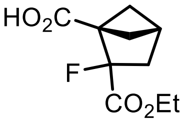

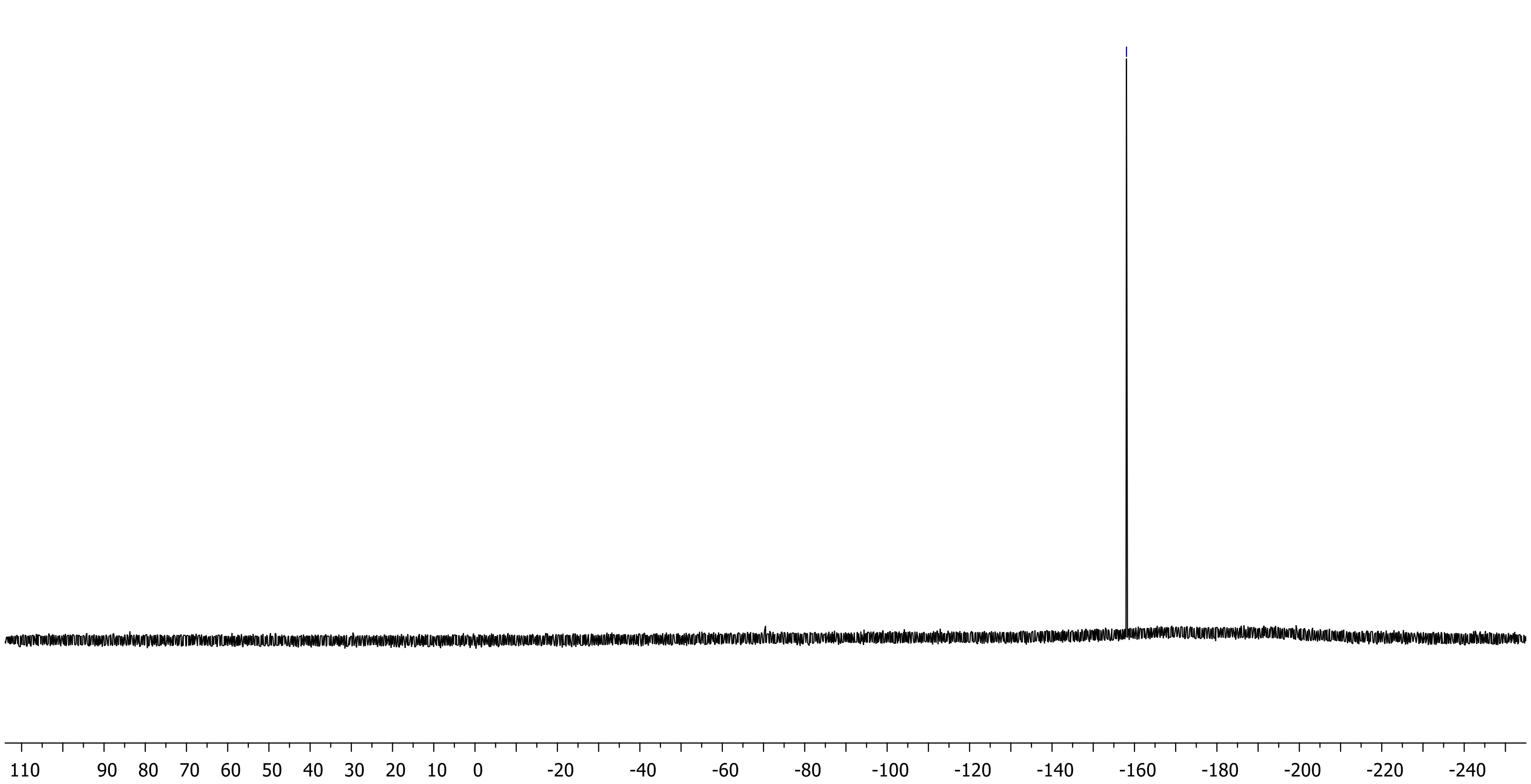

Compound (±)-26

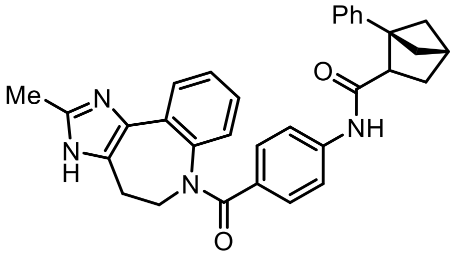

Ph
O
NH
Me
N
N
H
N
O

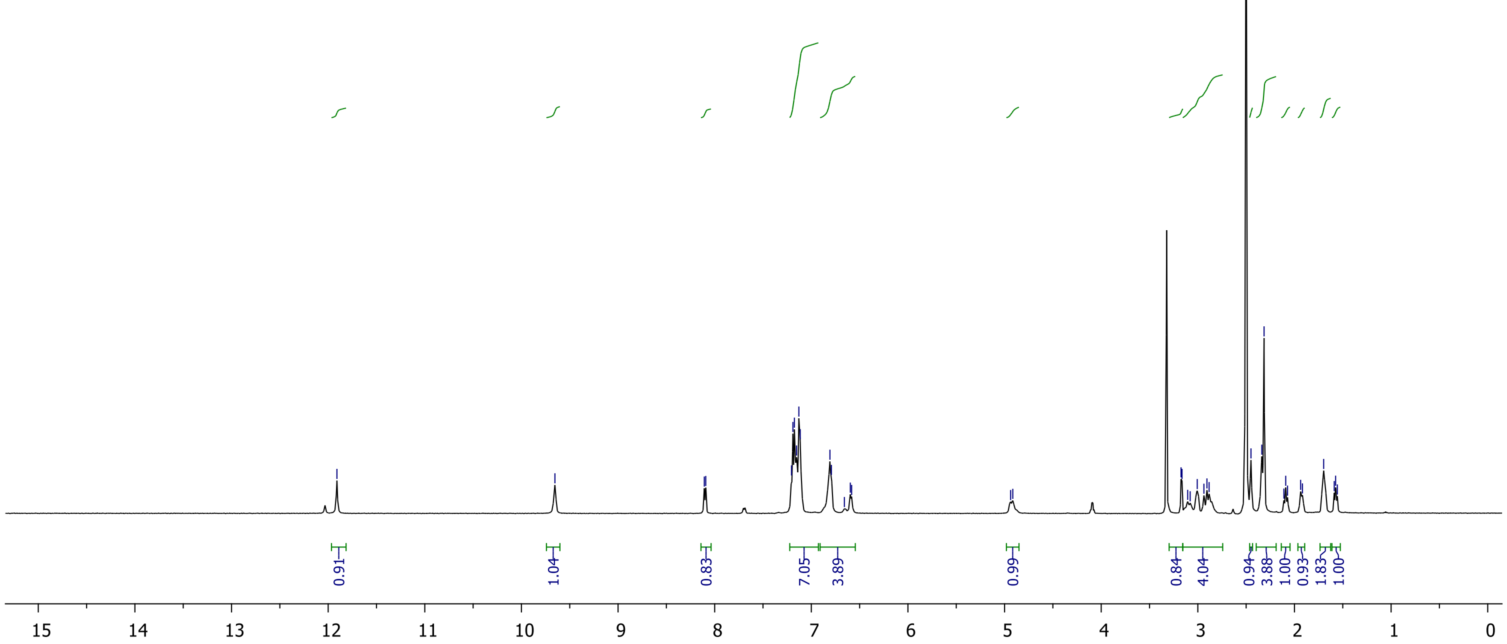

^{1}H NMR (500 MHz, DMSO-d$_6$)
BA682660$1
11.91
9.65
8.11
8.09
7.21
7.19
7.17
7.15
7.13
7.11
6.81
6.79
6.66
6.60
6.58
4.94
4.91
3.17
3.16
3.11
3.08
3.01
2.94
2.91
2.88
2.50
2.45
2.34
2.31
2.11
2.09
2.07
1.94
1.92
1.70
1.59
1.57
1.56
0.91
1.04
0.83
7.05
3.89
0.99
0.84
4.04
0.94
3.88
1.00
0.93
1.83
1.00
15
14
13
12
11
10
9
8
7
6
5
4
3
2
1
0

$^{13}C\{^{1}H\}$ NMR (151 MHz, DMSO-d$_6$)

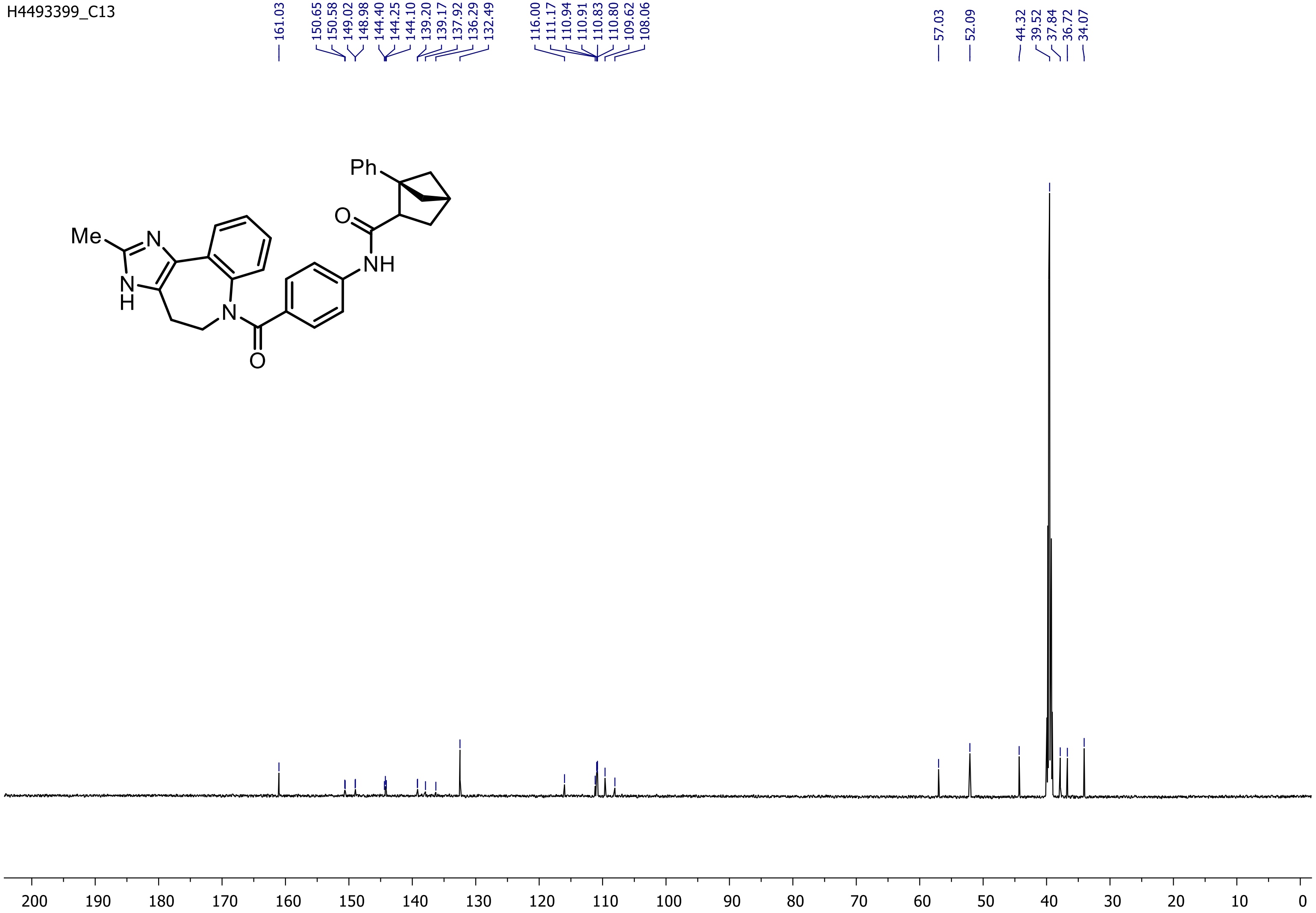

Compound (±)-27

^{1}H NMR (500 MHz, DMSO-d$_6$)

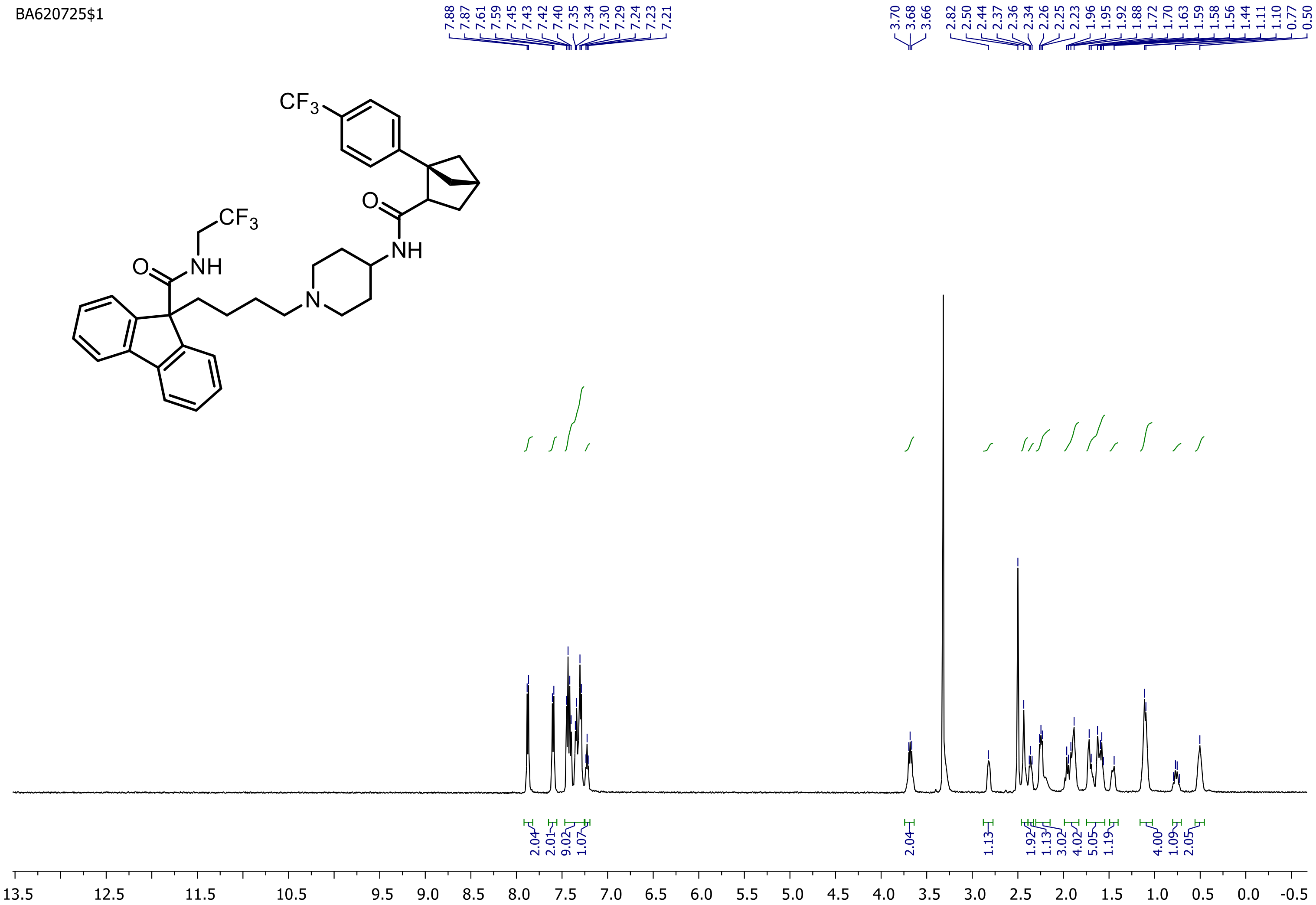

$^{13}C\{^{1}H\}$ NMR (151 MHz, DMSO-d_6)

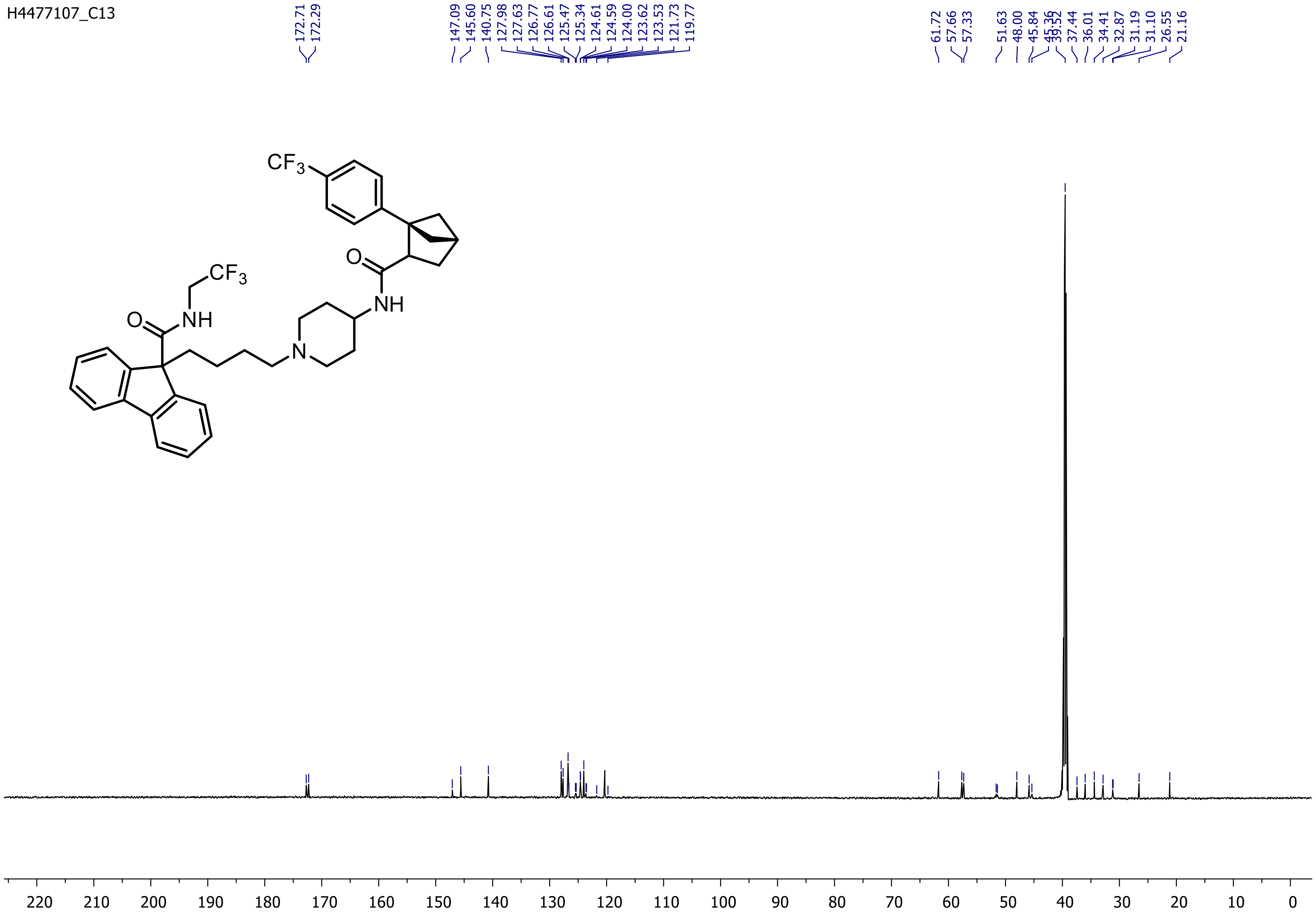

$^{19}F\{^{1}H\}$ NMR (376 MHz, DMSO-d$_6$)

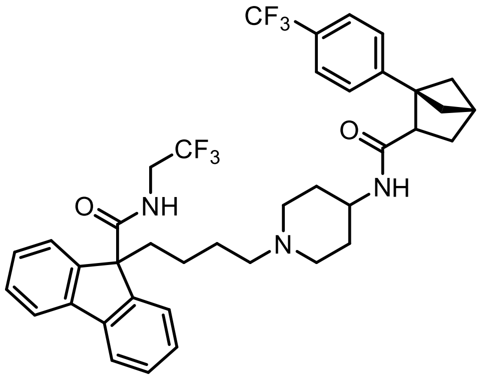

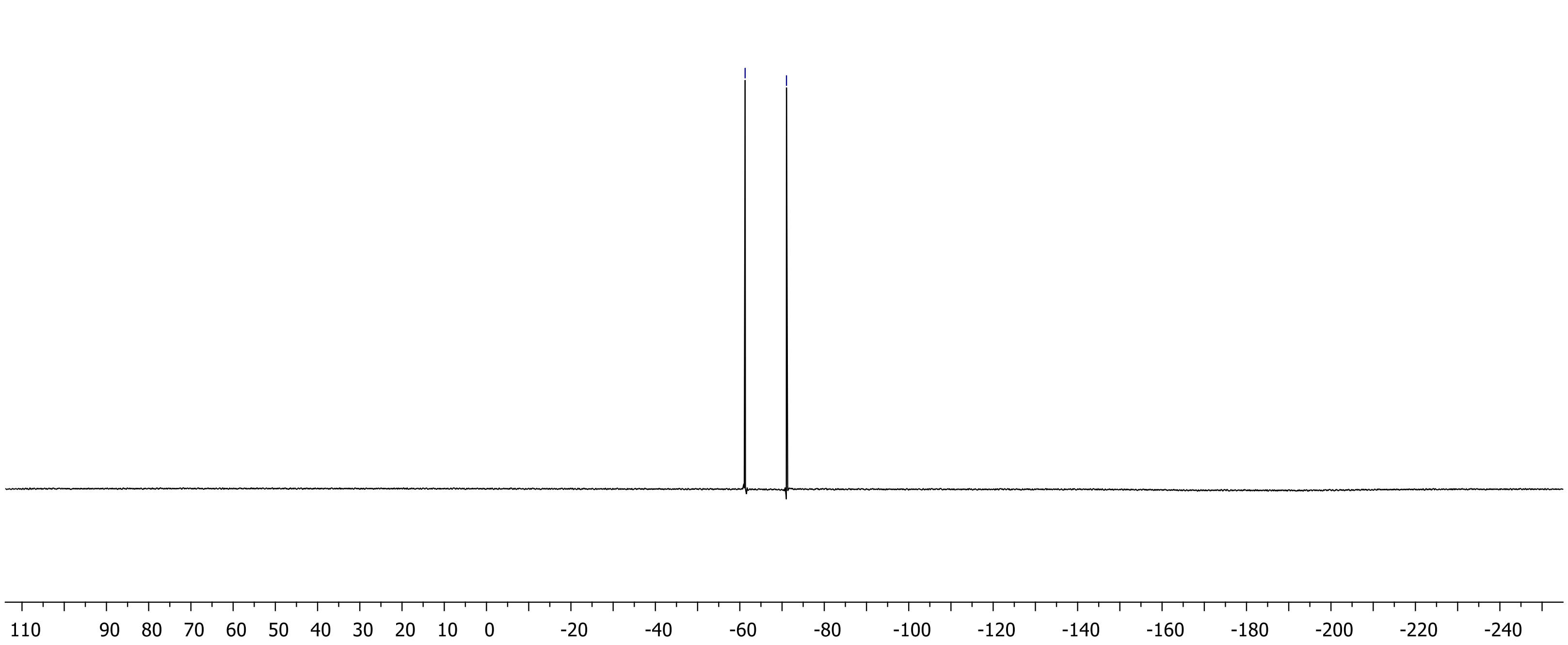

Tert-butyl (1-(4-chlorophenyl)bicyclo[2.1.1]hexan-2-yl)carbamate

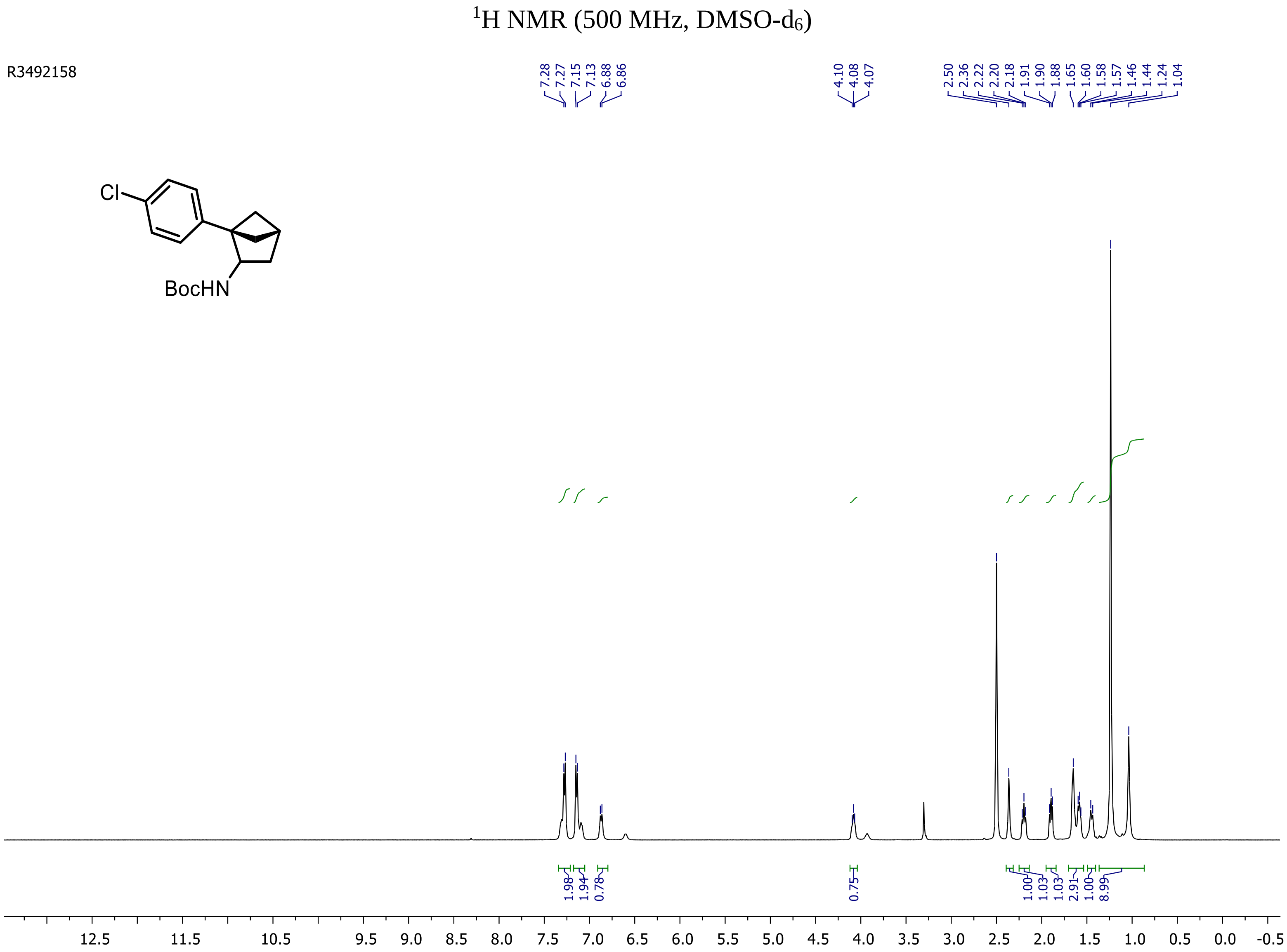

$^{13}C\{^1H\}$ NMR (151 MHz, DMSO-d$_6$)

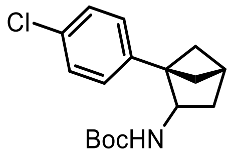

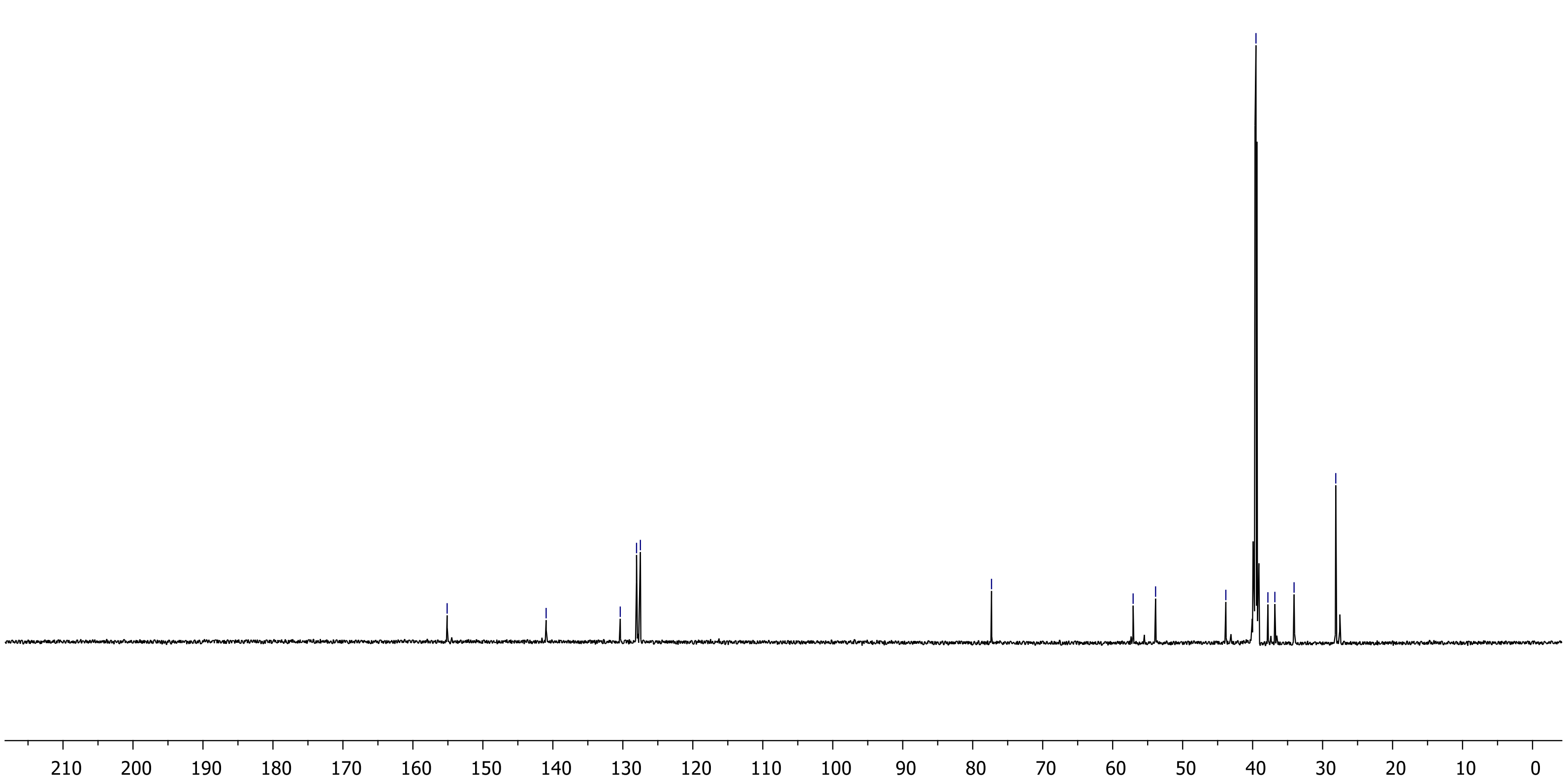

1-(4-Chlorophenyl)bicyclo[2.1.1]hexan-2-amine hydrochloride

^{1}H NMR (500 MHz, DMSO-d$_6$)

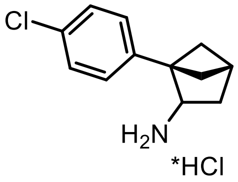

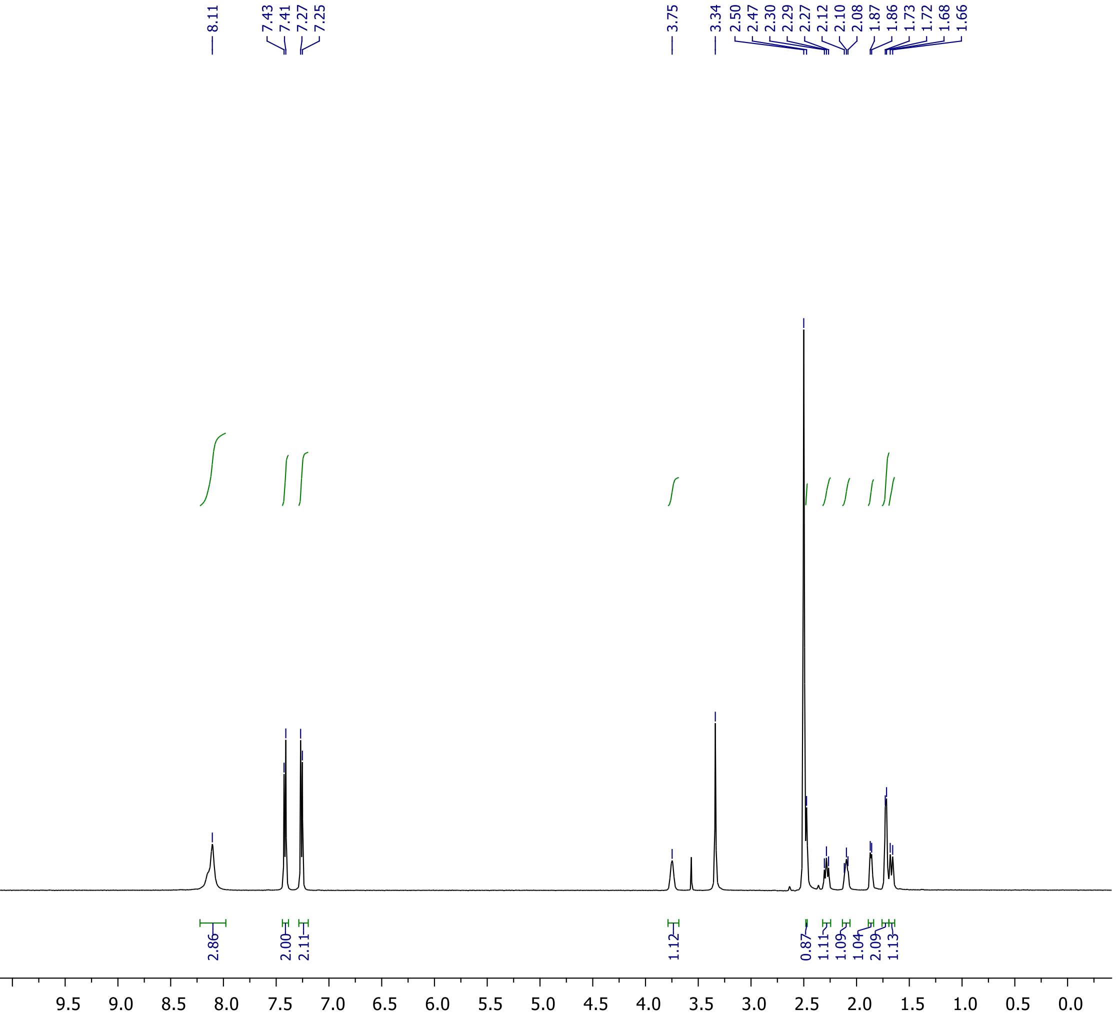

$^{13}C\{^{1}H\}$ NMR (151 MHz, DMSO-d_6)

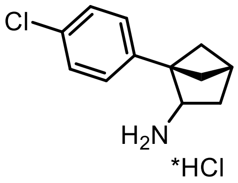

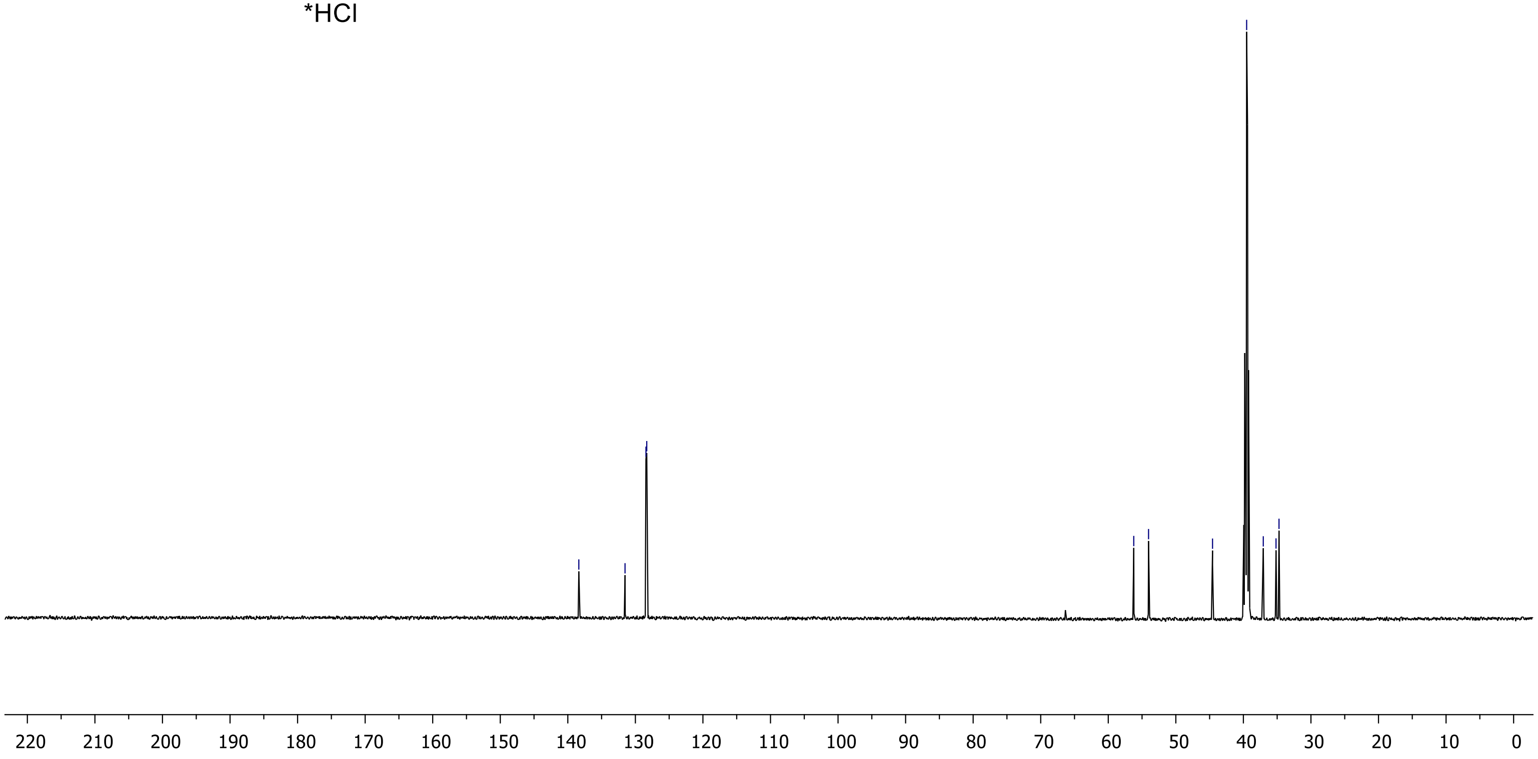

Compound (±)-28

^{1}H NMR (500 MHz, DMSO-d_6)

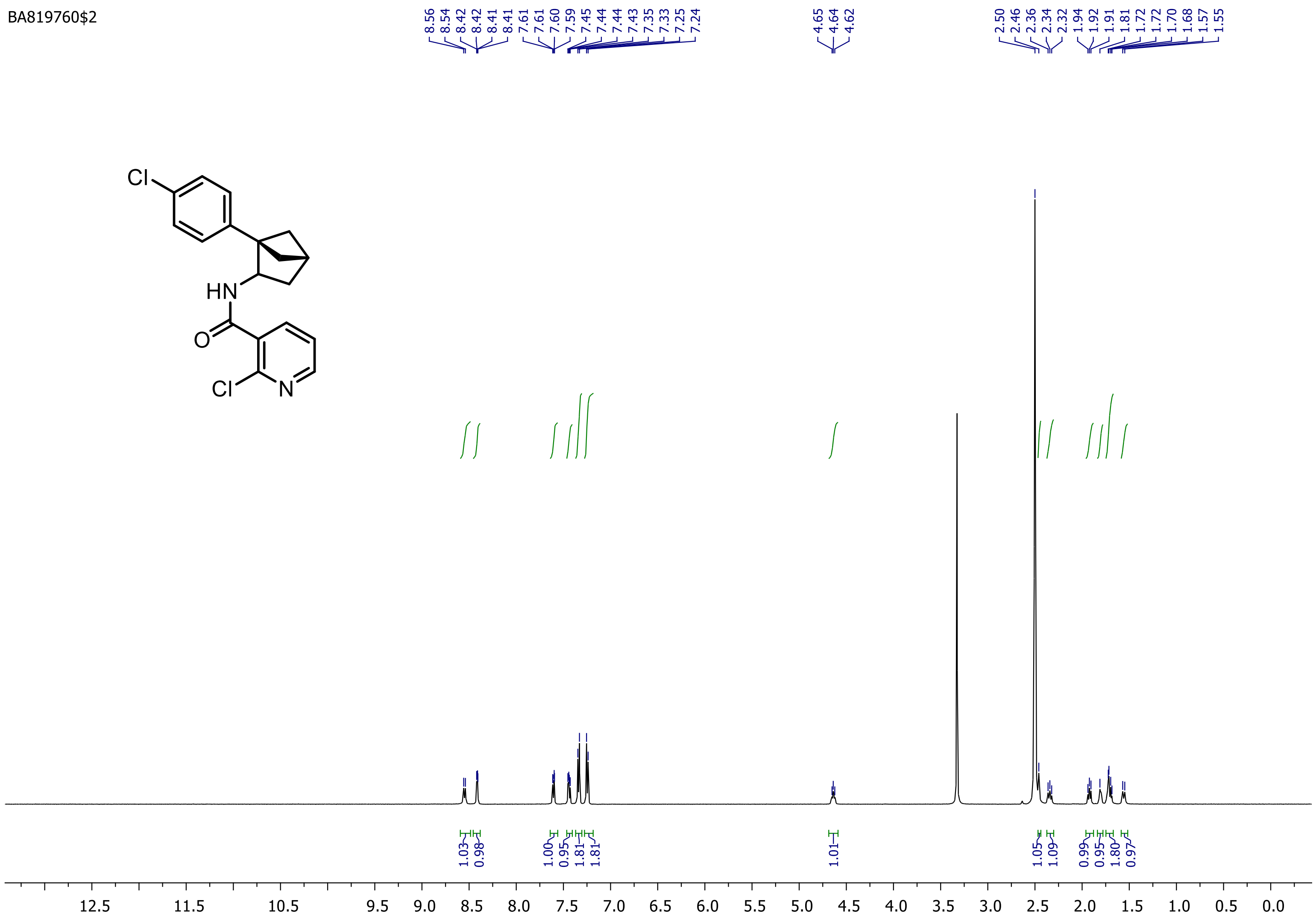

$^{13}C\{^{1}H\}$ NMR (151 MHz, DMSO-d_6)

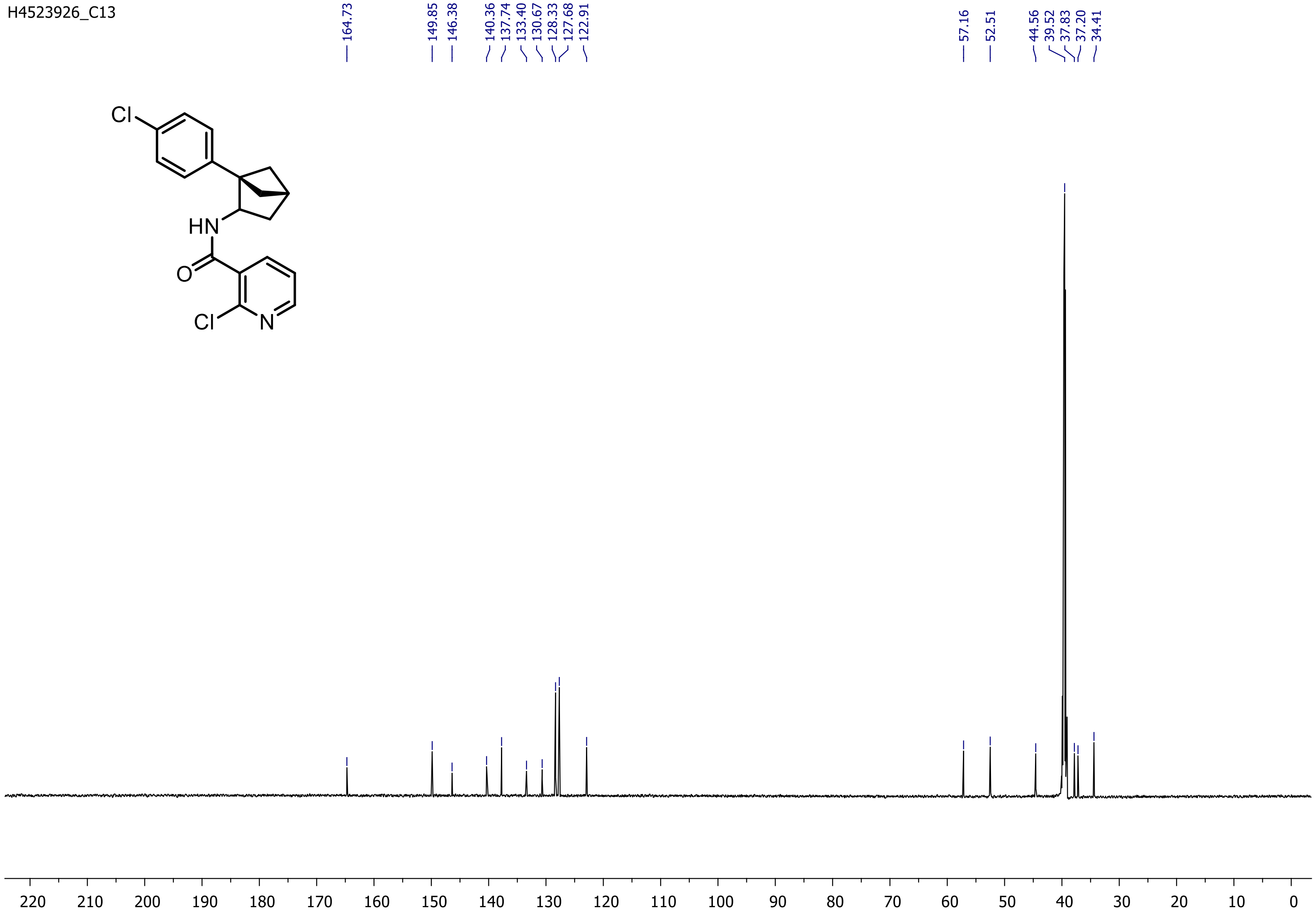

Tert-butyl (1-(3,4-dichlorophenyl)bicyclo[2.1.1]hexan-2-yl)carbamate

^{1}H NMR (500 MHz, DMSO-d$_6$)

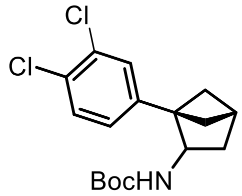

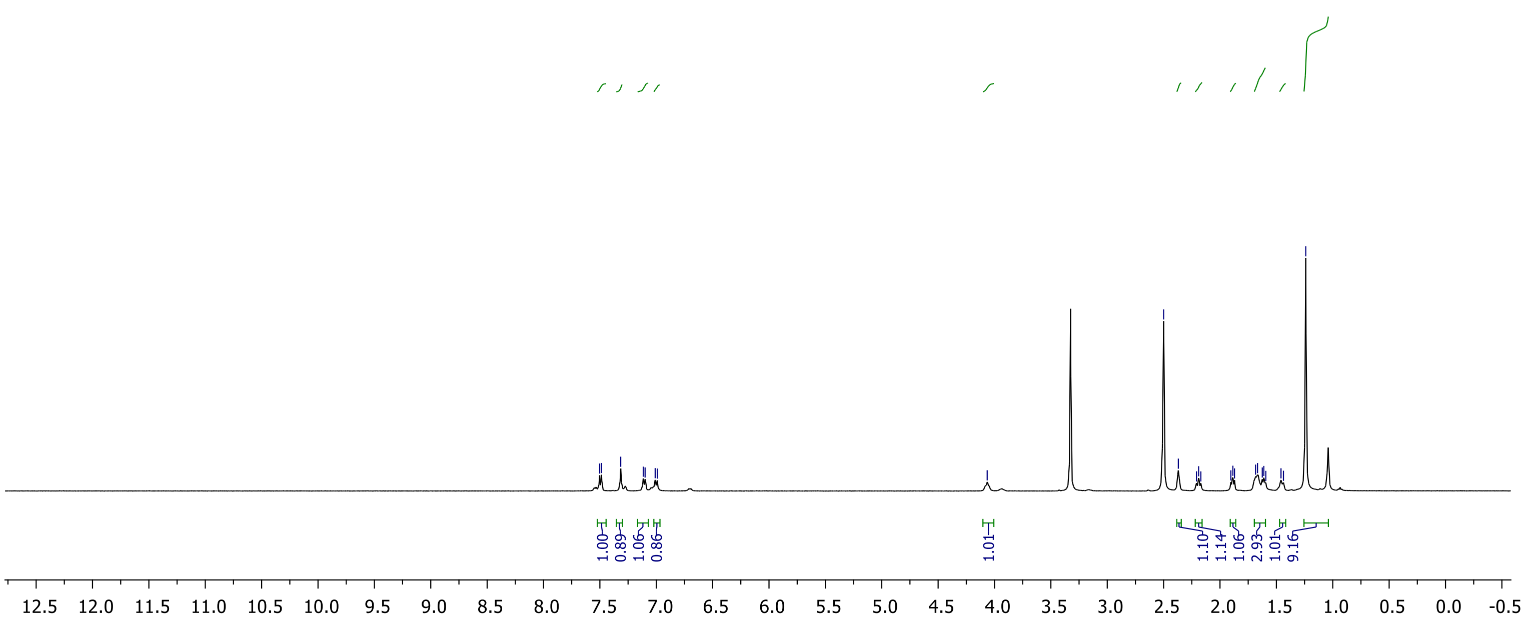

$^{13}C\{^{1}H\}$ NMR (101 MHz, DMSO-d_6)

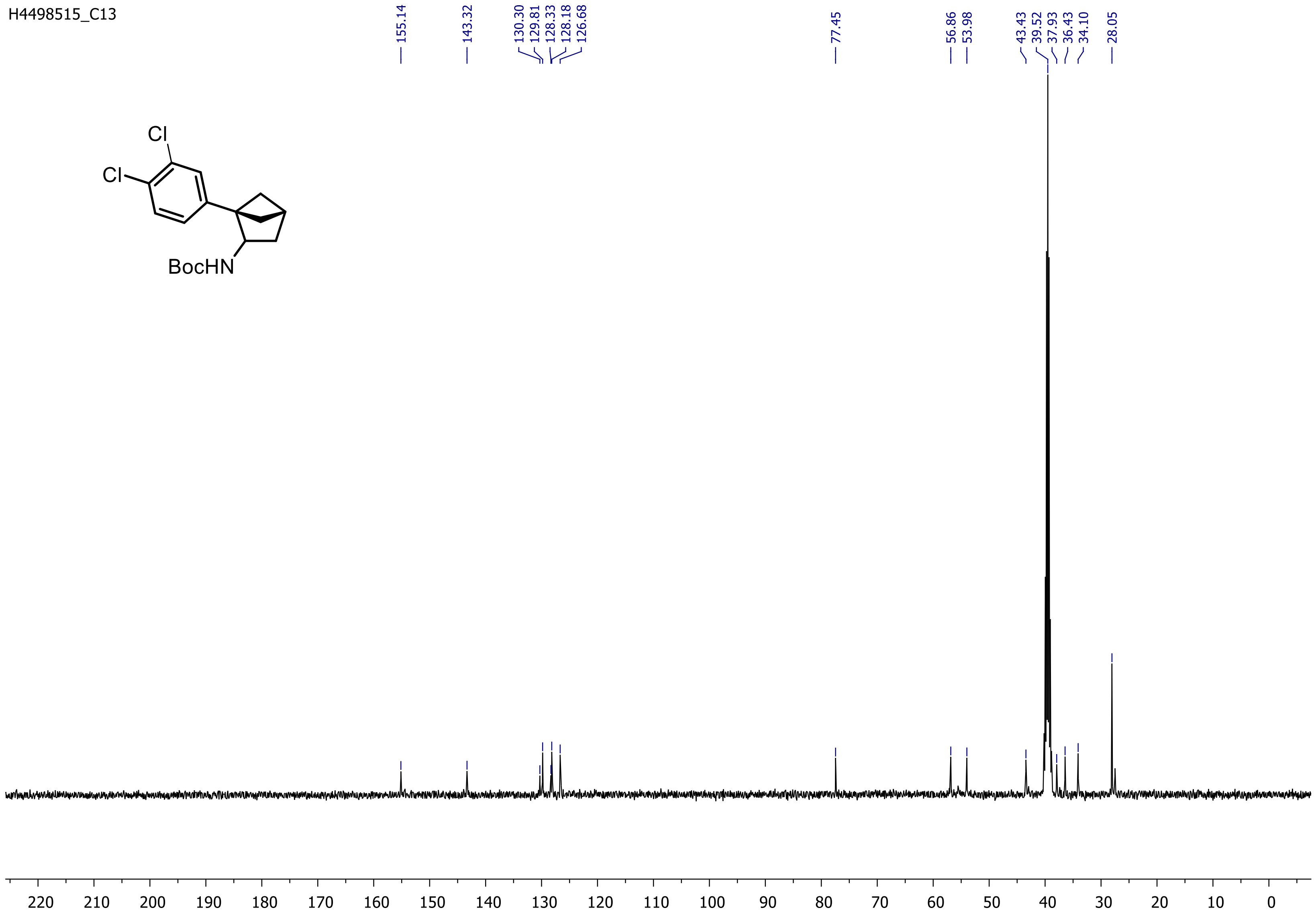

1-(3,4-Dichlorophenyl)bicyclo[2.1.1]hexan-2-amine hydrochloride

^{1}H NMR (500 MHz, DMSO-d$_6$)

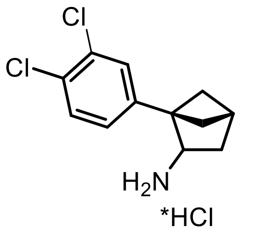

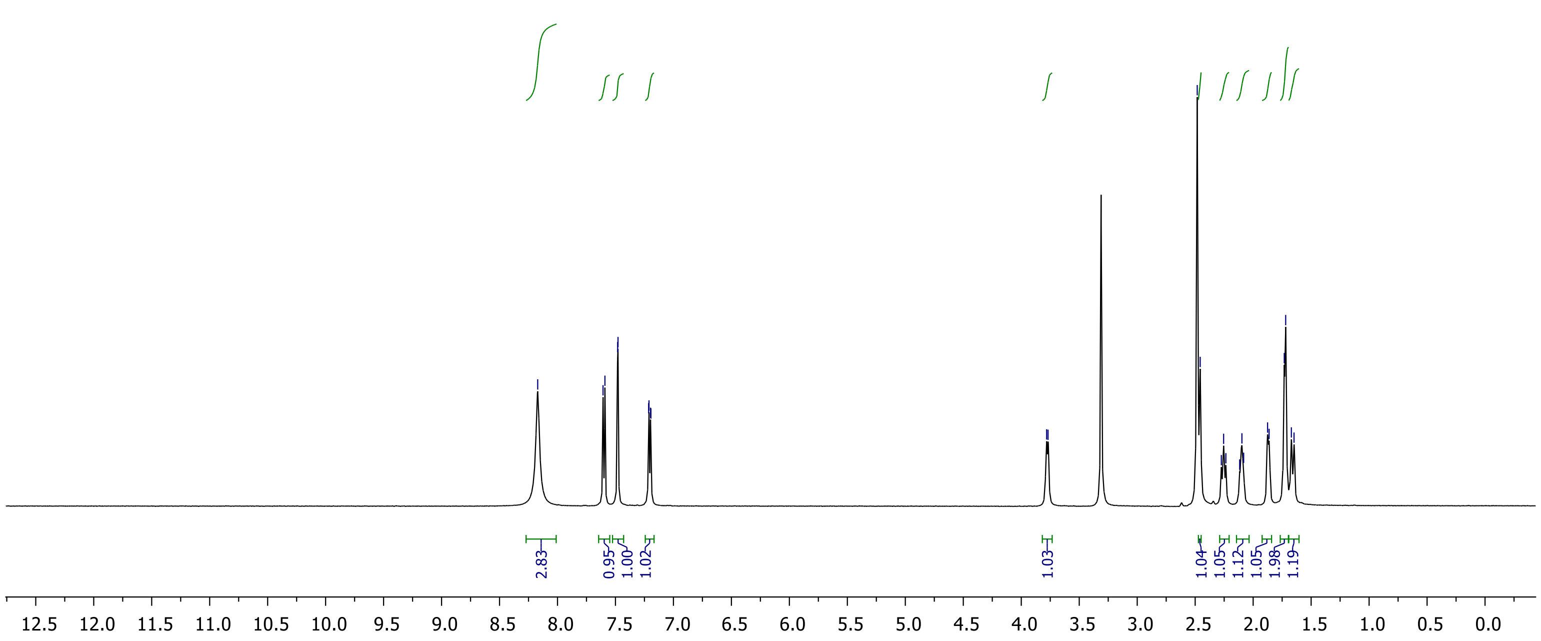

$^{13}C\{^{1}H\}$ (151 MHz, DMSO-d_6)

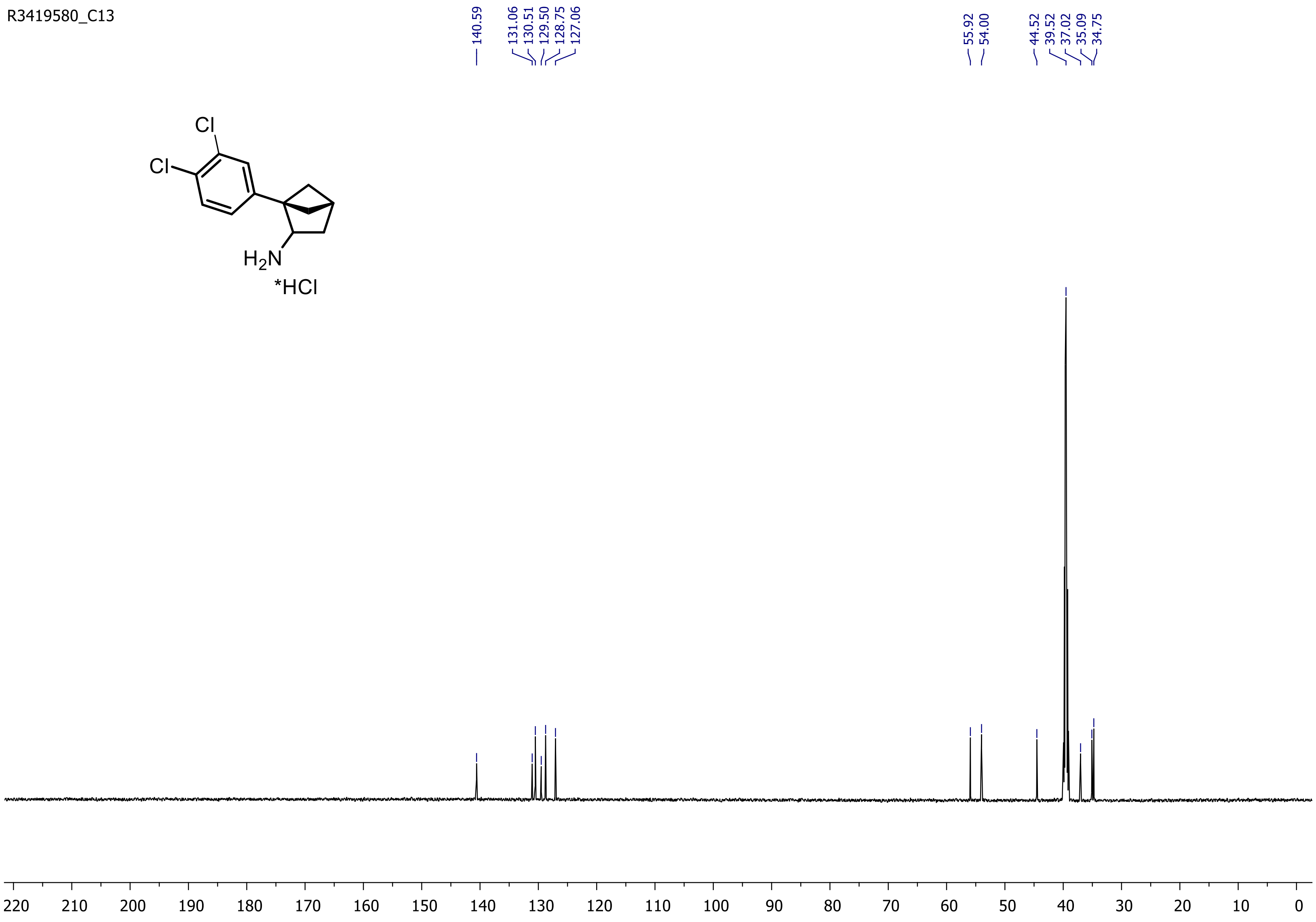

Compound (±)-29

^{1}H NMR (500 MHz, DMSO-d$_6$)

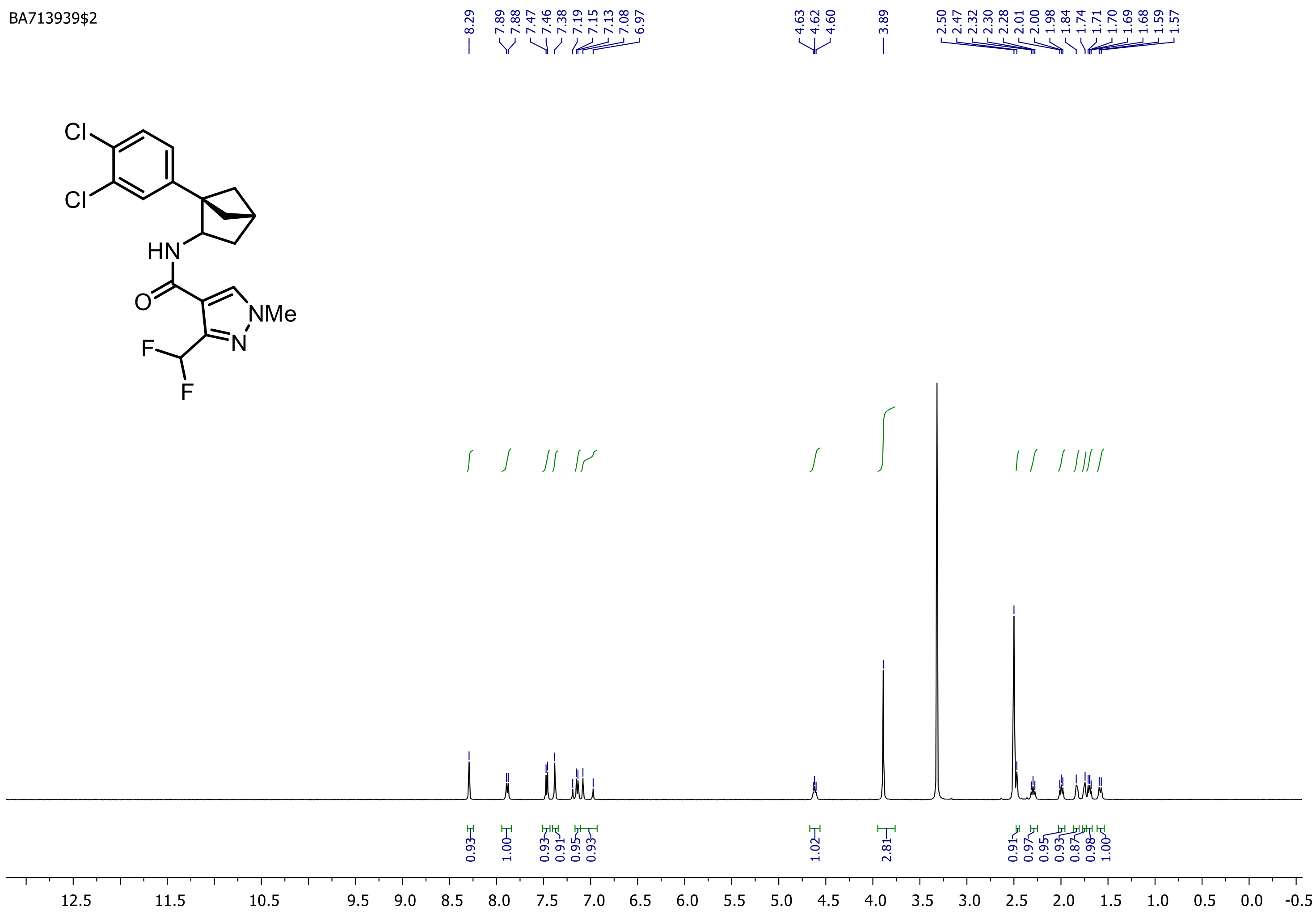

$^{13}C\{^1H\}$ NMR (151 MHz, DMSO-d_6)

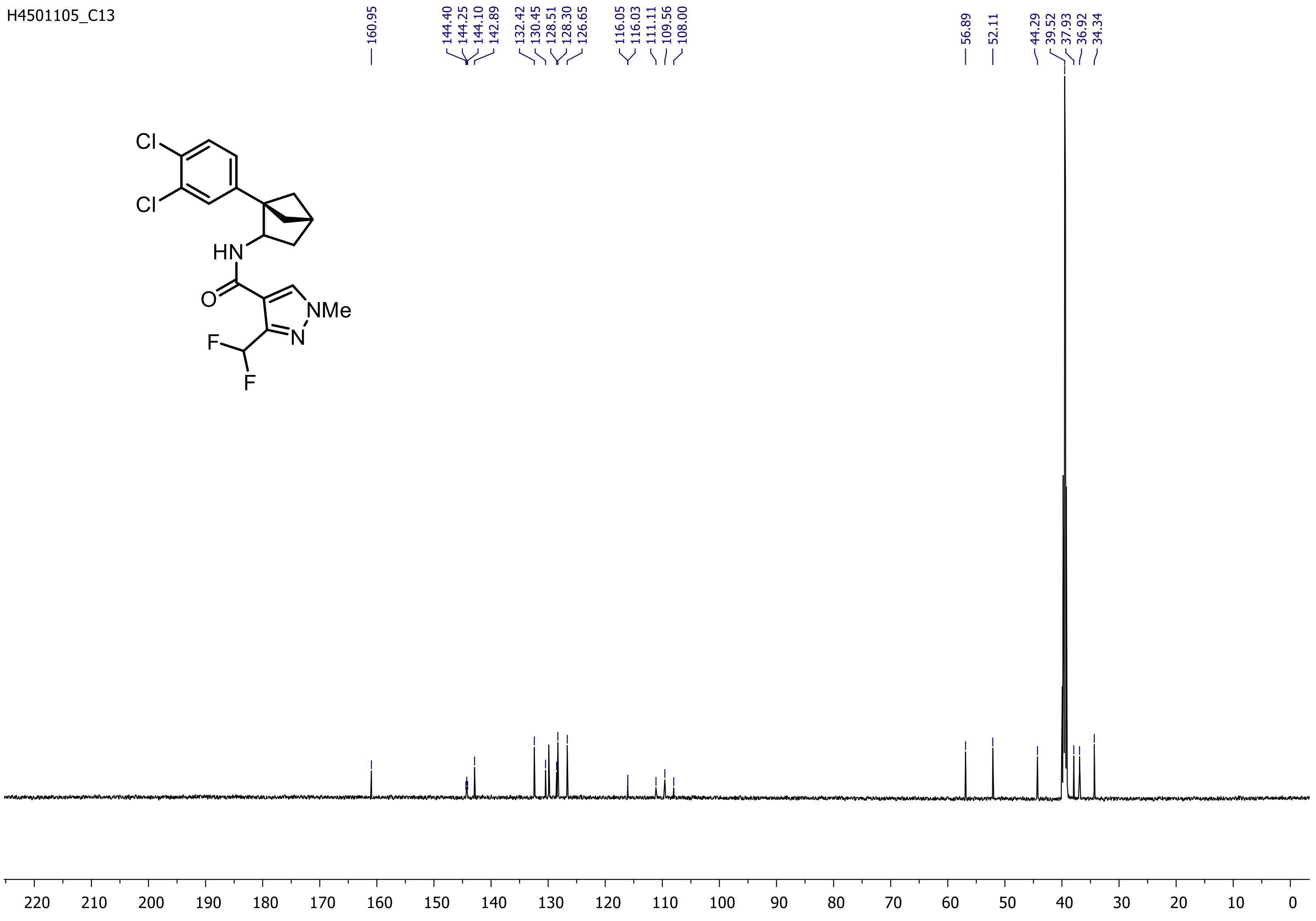

Tert-butyl (1-(3,4,5-trifluorophenyl)bicyclo[2.1.1]hexan-2-yl)carbamate

^{1}H NMR (500 MHz, DMSO-d_6)

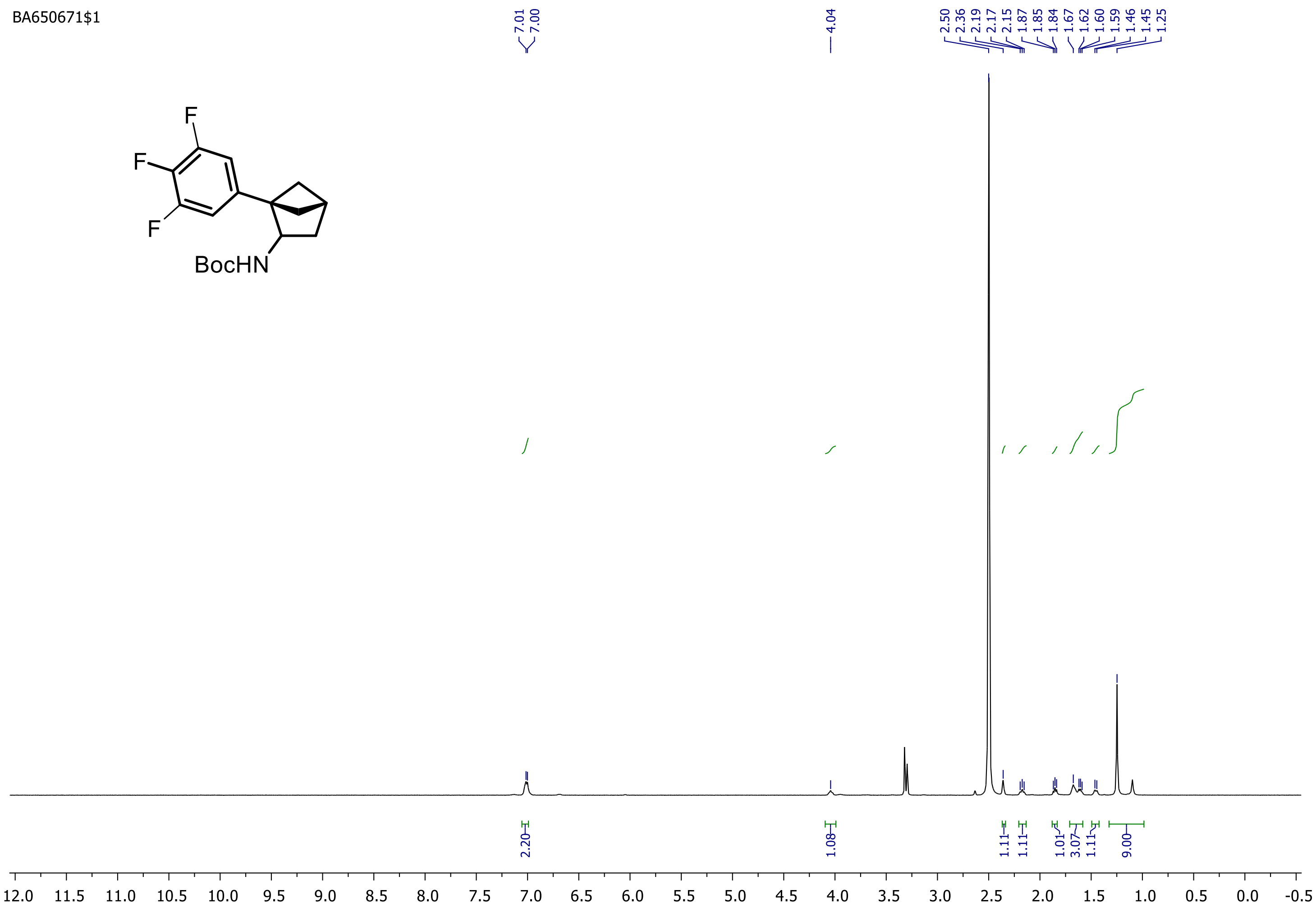

$^{13}C\{^{1}H\}$ NMR (151 MHz, DMSO-d_6)

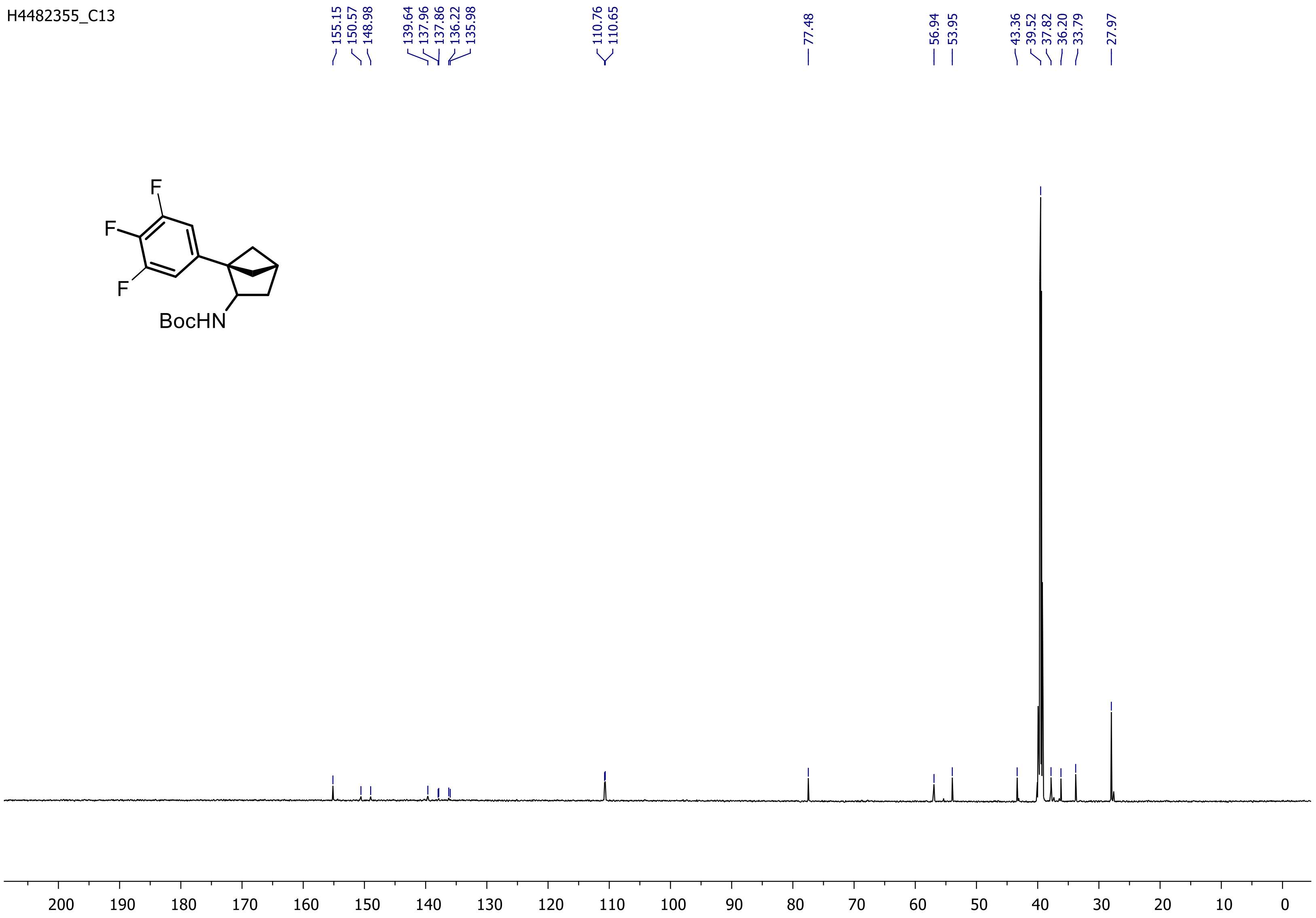

$^{19}F\{^{1}H\}$ NMR (376 MHz, DMSO-d_6)

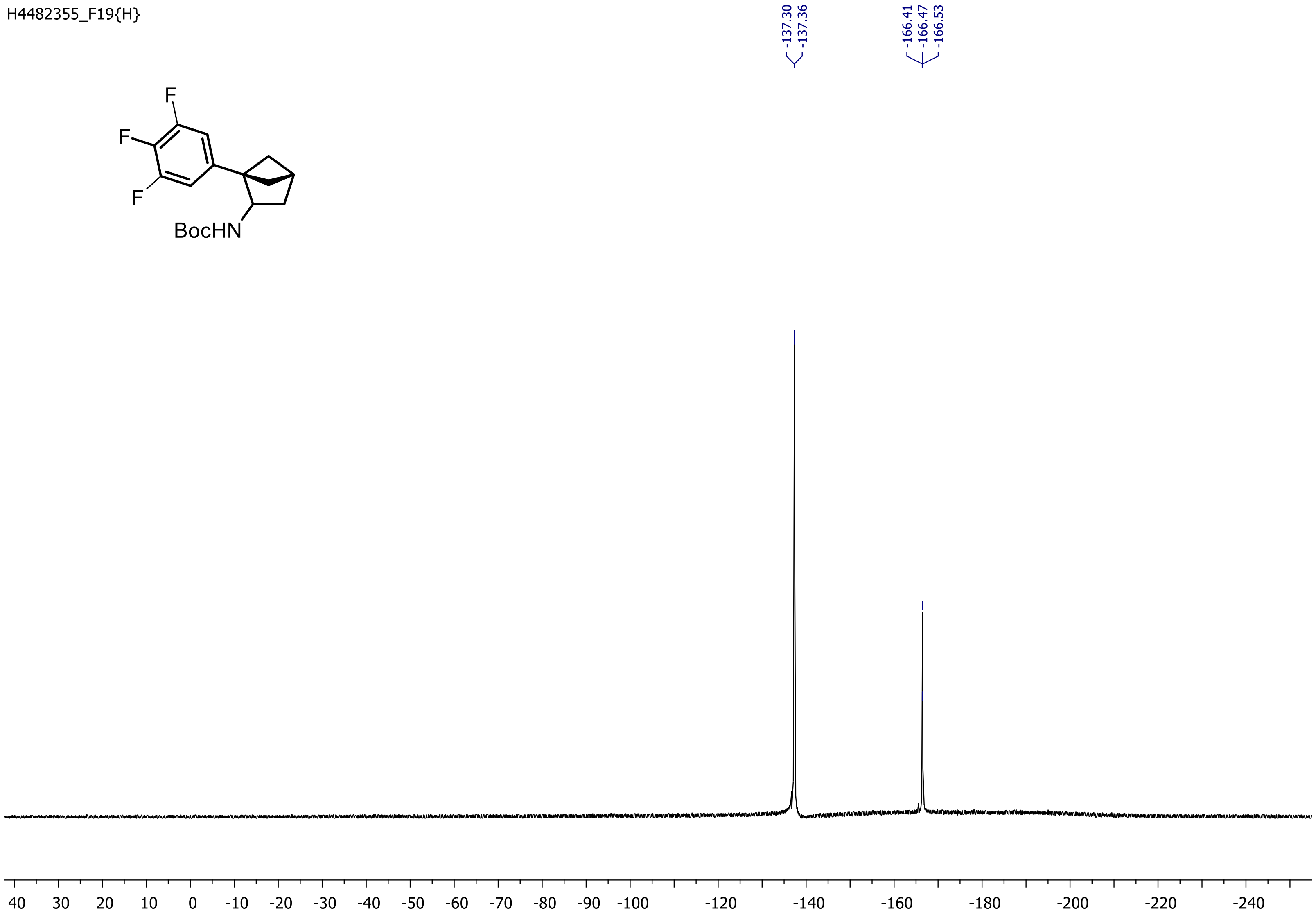

1-(3,4,5-Trifluorophenyl)bicyclo[2.1.1]hexan-2-amine hydrochloride

^{1}H NMR (500 MHz, DMSO-d_6)

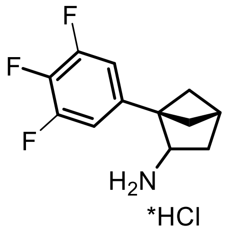

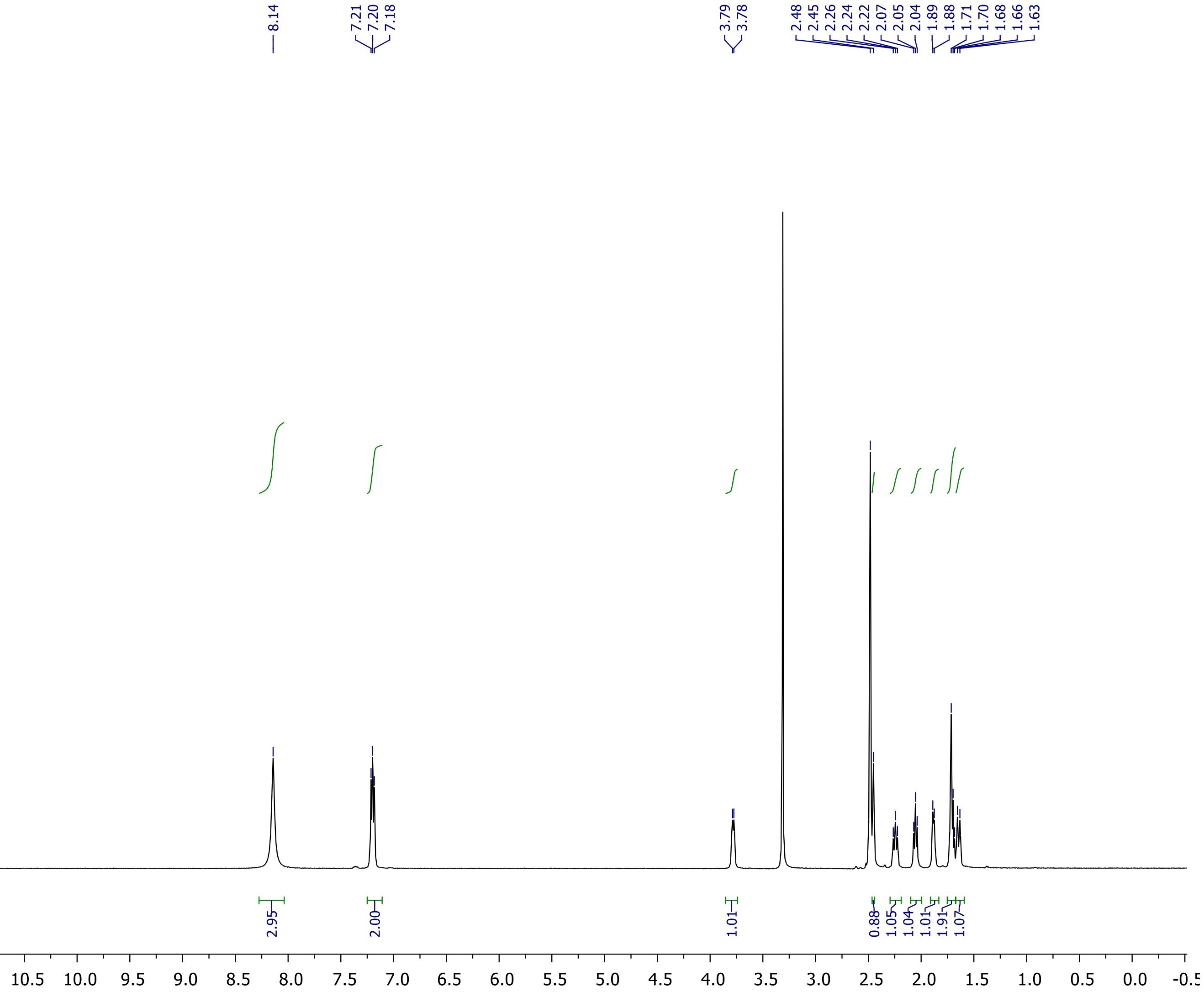

$^{13}C\{^1H\}$ NMR (151 MHz, DMSO-d_6)

H4493393_C13

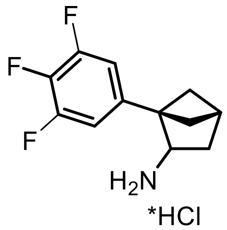

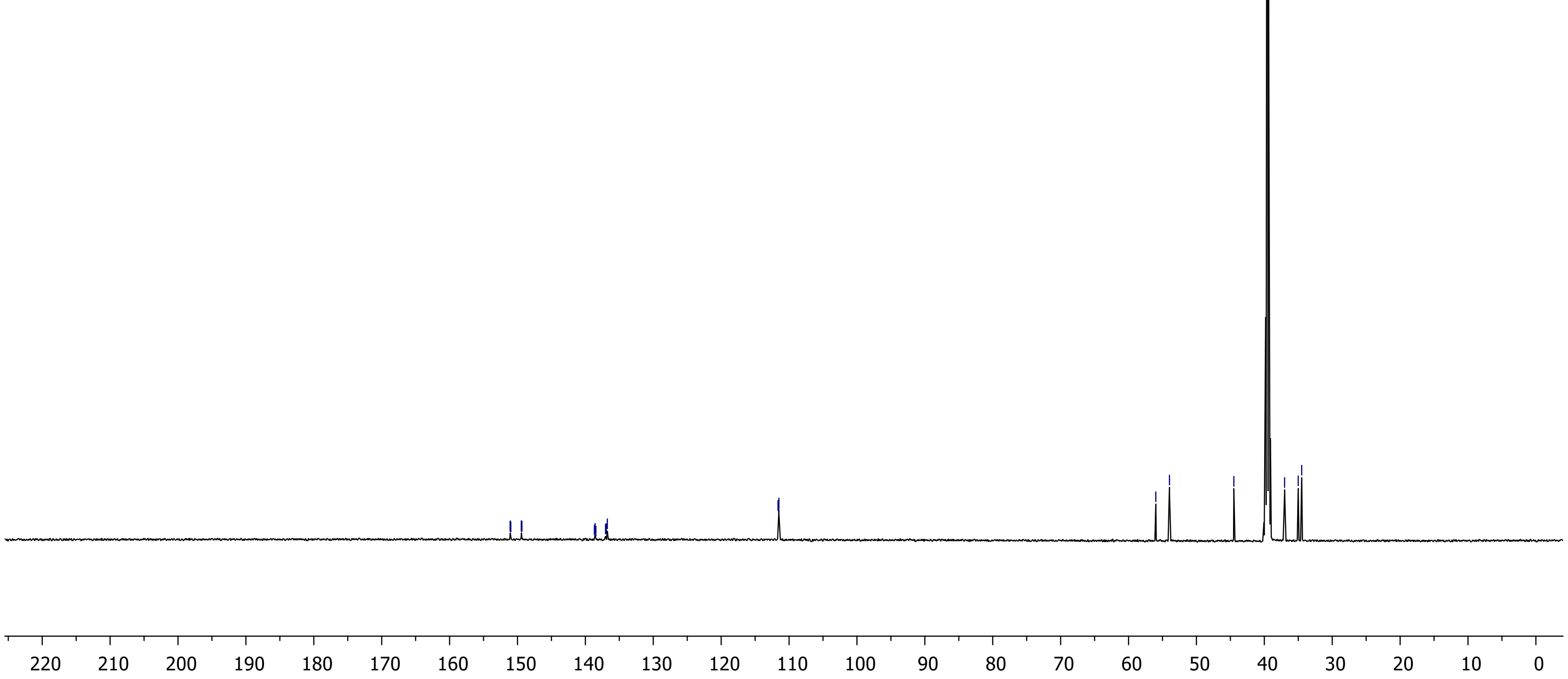

$^{19}F\{^{1}H\}$ NMR (376 MHz, DMSO-d_6)

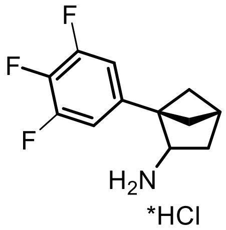

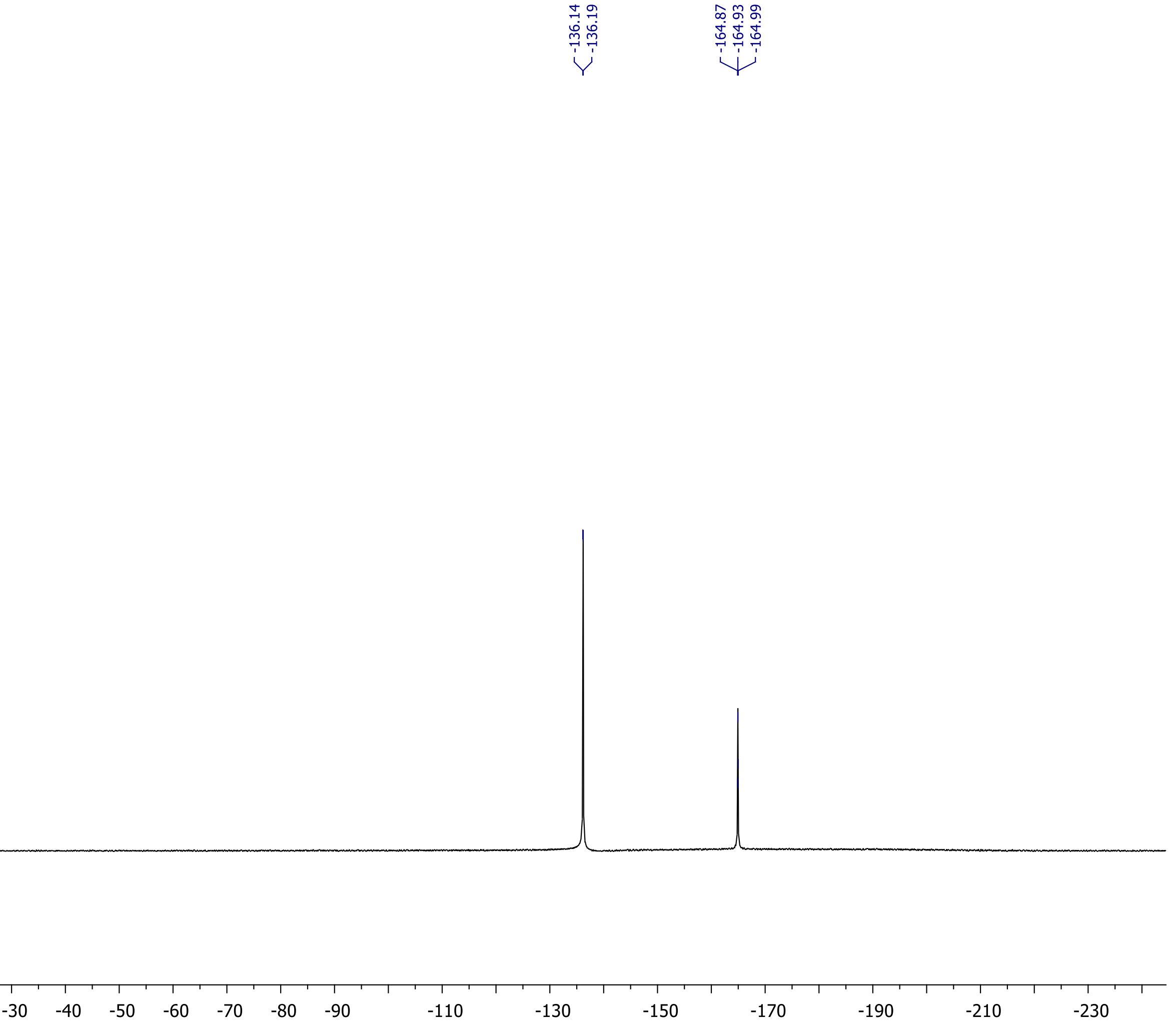

Compound (±)-30

^{1}H NMR (500 MHz, DMSO-d$_6$)

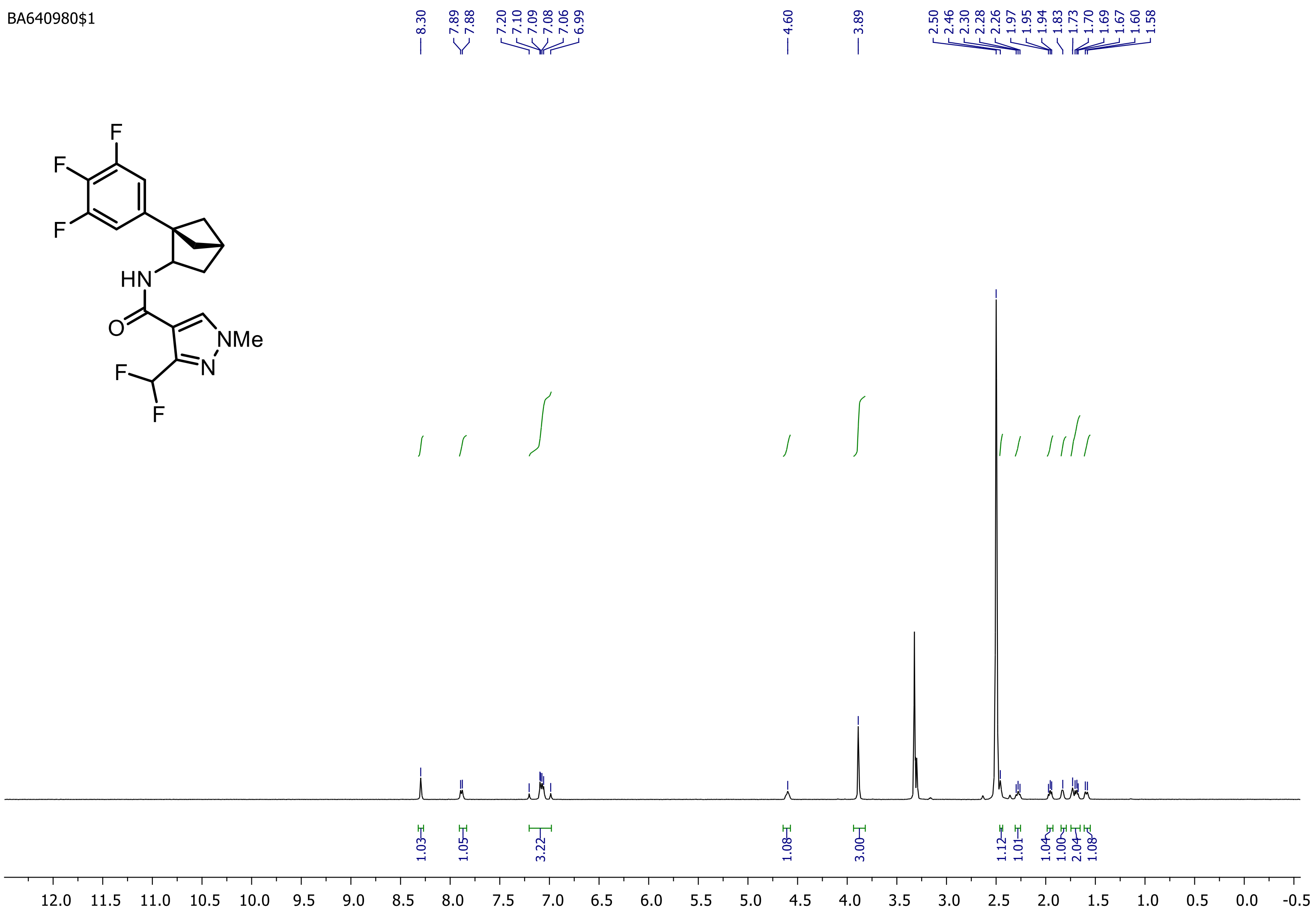

$^{13}C\{^{1}H\}$ NMR (151 MHz, DMSO-d$_6$)

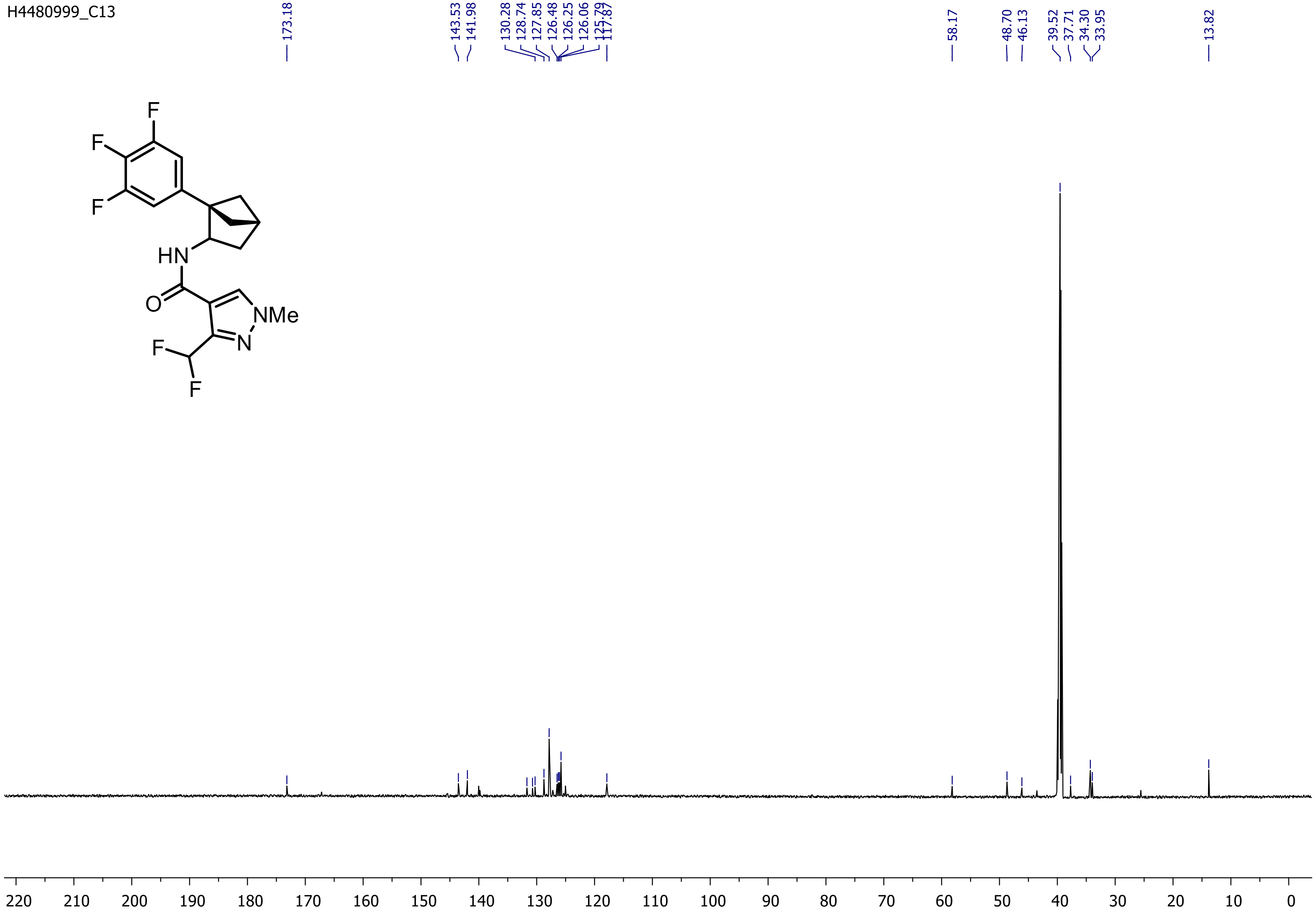

$^{19}F\{^{1}H\}$ NMR (376 MHz, DMSO-d_6)

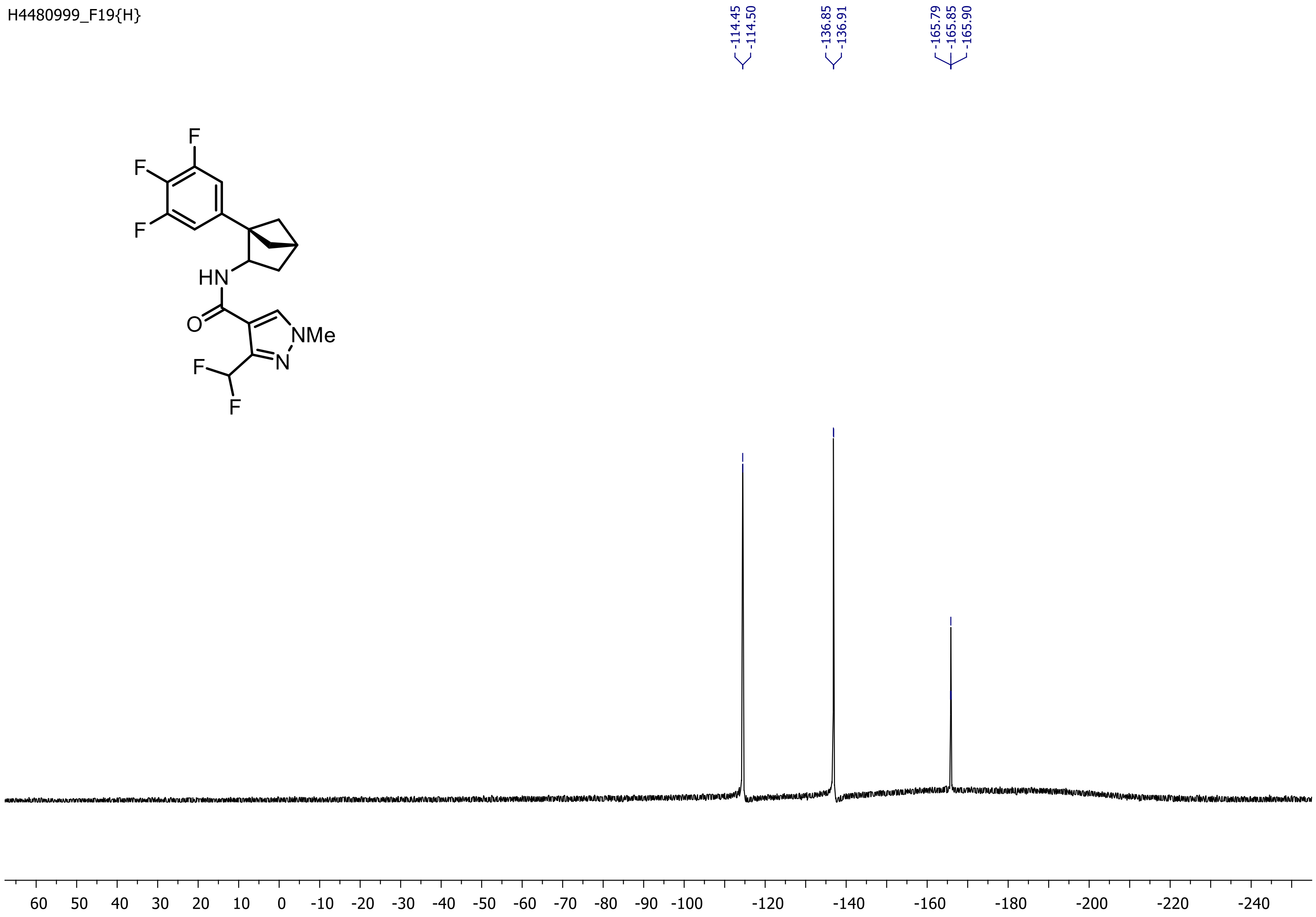

Crystallographic data (X-Ray)

Crystals of compounds **1b**, **3b**, **4b**, **10b**, **12b**, **28** and **29** suitable for X-Ray diffraction studies were obtained by a low evaporation of a solution of MeOH. Diffraction data were collected at room temperature on an Xcallibur-3 diffractometer with graphite-monochromated Mo Kα radiation (λ = 0.71073 Å) operating in the w-scans mode. The structure was solved by direct methods and refined by the full-matrix least-squares technique in the anisotropic approximation for non-hydrogen atoms using the SHELXTL program package. Crystallographic data for all structures in this paper have been deposited at Cambridge Crystallographic Data Centre. CCDC numbers: **1b** (2286523), **3b** (2286521), **4b** (2286526), **10b** (2286525), **12b** (2286524), **28** (2286523) and **29** (2286527). Copies of the data can be obtained, free of charge, on application to CCDC, 12 Union Road, Cambridge CB21EZ, UK, (fax: +44-(0)1223-336033 or e-mail: deposit@ccdc.cam.ac.uk).

Compound **1b**

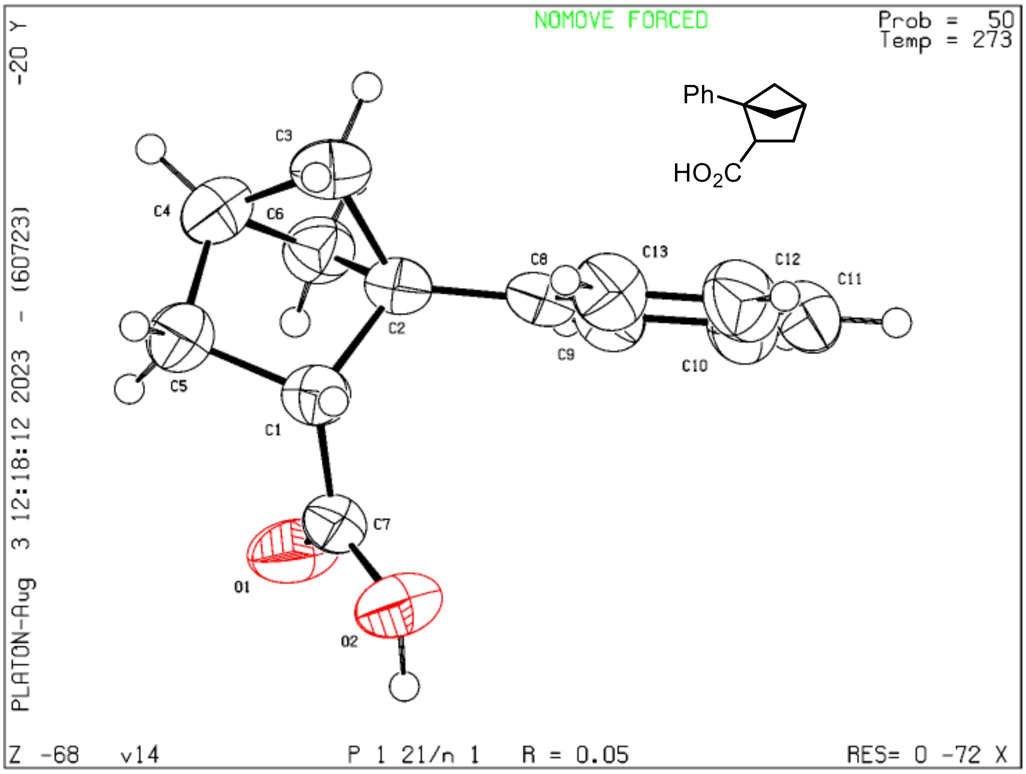

Figure S1. Molecular structure of **1b** according to X-Ray diffraction data. Thermal ellipsoids are shown at 50% probability level.

Crystal structure determination of 1b

data_v14

_chemical_formula_moiety	'C13 H14 O2'
_chemical_formula_weight	202.24

_space_group_crystal_system 'monoclinic'

_space_group_IT_number 14

_space_group_name_H-M_alt 'P 1 21/n 1'

_space_group_name_Hall '-P 2yn'

_cell_length_a 12.304(10)

_cell_length_b 6.165(5)

_cell_length_c 14.153(10)

_cell_angle_alpha 90

_cell_angle_beta 90.53(5)

_cell_angle_gamma 90

_cell_volume 1073.5(15)

_cell_formula_units_Z 4

_cell_measurement_reflns_used 1248

_cell_measurement_temperature 273.15

_cell_measurement_theta_max 18.77

_cell_measurement_theta_min 3.31

_shelx_estimated_absorpt_T_max 0.996

_shelx_estimated_absorpt_T_min 0.984

_exptl_absorpt_coefficient_mu 0.083

_exptl_crystal_colour colourless

_exptl_crystal_colour_primary colourless

_exptl_crystal_density_diffrn 1.251

_exptl_crystal_description block

_exptl_crystal_F_000 432

_exptl_crystal_size_max 0.2

_exptl_crystal_size_mid 0.05

_exptl_crystal_size_min 0.05

_diffrn_reflns_av_R_equivalents 0.0632

_diffrn_reflns_av_unetI/netI 0.0471

_diffrn_reflns_Laue_measured_fraction_full 1.000

_diffrn_reflns_Laue_measured_fraction_max 1.000

_diffrn_reflns_limit_h_max 14

_diffrn_reflns_limit_h_min -14

_diffrn_reflns_limit_k_max 7

_diffrn_reflns_limit_k_min -7

_diffrn_reflns_limit_l_max 16
_diffrn_reflns_limit_l_min -16
_diffrn_reflns_number 12492
_diffrn_reflns_point_group_measured_fraction_full 1.000
_diffrn_reflns_point_group_measured_fraction_max 1.000
_diffrn_reflns_theta_full 24.997
_diffrn_reflns_theta_max 24.997
_diffrn_reflns_theta_min 2.183
_diffrn_ambient_temperature 273.15
_diffrn_measured_fraction_theta_full 1.000
_diffrn_measured_fraction_theta_max 1.000
_diffrn_measurement_device_type 'Bruker APEX-II CCD'
_diffrn_radiation_type MoK\a
_diffrn_radiation_wavelength 0.71073
_diffrn_source_current 30.0
_diffrn_source_power 1.2
_diffrn_source_voltage 40.0

Compound **3b**

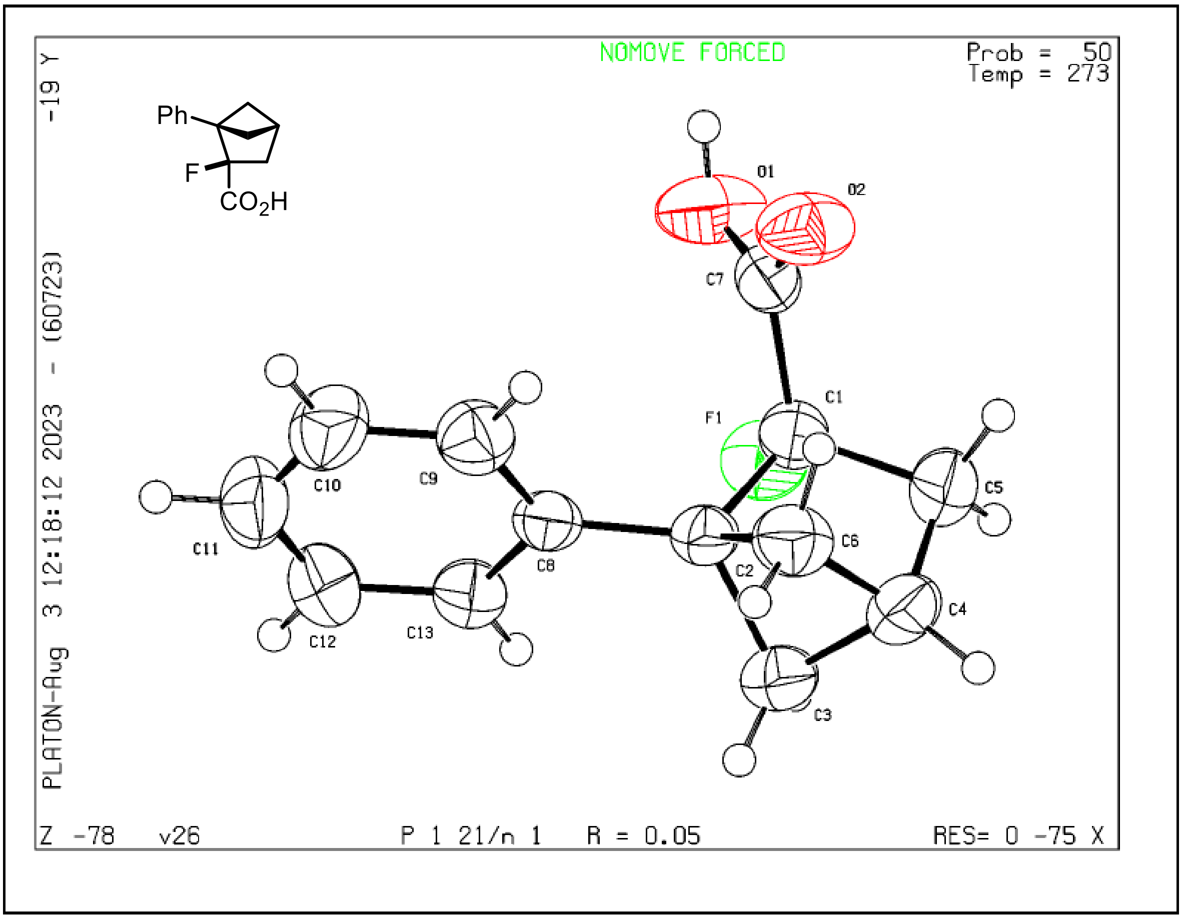

Figure S2. Molecular structure of **3b** according to X-Ray diffraction data. Thermal ellipsoids are shown at 50% probability level.

Crystal structure determination of 3b

data_v26

_chemical_formula_moiety	'C13 H13 F O2'
_chemical_formula_weight	220.23
_space_group_crystal_system	'monoclinic'
_space_group_IT_number	14
_space_group_name_H-M_alt	'P 1 21/n 1'
_space_group_name_Hall	'-P 2yn'
_cell_length_a	12.625(3)
_cell_length_b	6.2763(14)
_cell_length_c	14.050(3)
_cell_angle_alpha	90
_cell_angle_beta	95.590(14)
_cell_angle_gamma	90

_cell_volume 1108.0(4)
_cell_formula_units_Z 4
_cell_measurement_reflns_used 2379
_cell_measurement_temperature 273.15
_cell_measurement_theta_max 20.66
_cell_measurement_theta_min 2.91
_shelx_estimated_absorpt_T_max 0.990
_shelx_estimated_absorpt_T_min 0.980
_exptl_absorpt_coefficient_mu 0.099
_exptl_absorpt_correction_type none
_exptl_crystal_colour colourless
_exptl_crystal_colour_primary colourless
_exptl_crystal_density_diffrn 1.320
_exptl_crystal_description block
_exptl_crystal_F_000 464
_exptl_crystal_size_max 0.21
_exptl_crystal_size_mid 0.12
_exptl_crystal_size_min 0.1
_diffrn_reflns_av_R_equivalents 0.0520
_diffrn_reflns_av_unetI/netI 0.0351
_diffrn_reflns_Laue_measured_fraction_full 1.000
_diffrn_reflns_Laue_measured_fraction_max 1.000
_diffrn_reflns_limit_h_max 15
_diffrn_reflns_limit_h_min -12
_diffrn_reflns_limit_k_max 7
_diffrn_reflns_limit_k_min -7
_diffrn_reflns_limit_l_max 16
_diffrn_reflns_limit_l_min -16
_diffrn_reflns_number 13942
_diffrn_reflns_point_group_measured_fraction_full 1.000
_diffrn_reflns_point_group_measured_fraction_max 1.000
_diffrn_reflns_theta_full 24.996
_diffrn_reflns_theta_max 24.996
_diffrn_reflns_theta_min 2.071
_diffrn_ambient_temperature 273.15

_diffrn_measured_fraction_theta_full 1.000

_diffrn_measured_fraction_theta_max 1.000

_diffrn_measurement_device_type   'Bruker APEX-II CCD'

_diffrn_measurement_method   '\f and \w scans'

_diffrn_radiation_type   MoK\a

_diffrn_radiation_wavelength   0.71073

_diffrn_source_current   30.0

_diffrn_source_power   1.2

_diffrn_source_voltage   40.0

Compound **4b**

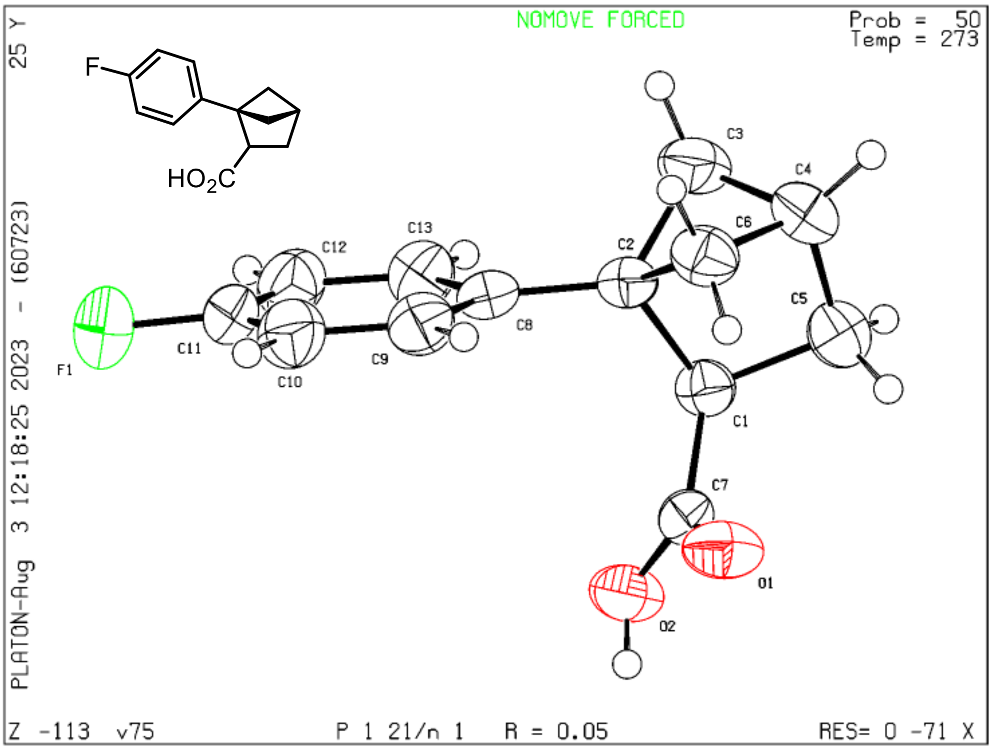

Figure S3. Molecular structure of **4b** according to X-Ray diffraction data. Thermal ellipsoids are shown at 50% probability level.

Crystal structure determination of 4b

data_v75

_chemical_formula_moiety	'C13 H13 F O2'
_chemical_formula_weight	220.23
_space_group_crystal_system	'monoclinic'
_space_group_IT_number	14
_space_group_name_H-M_alt	'P 1 21/n 1'
_space_group_name_Hall	'-P 2yn'
_cell_length_a	12.2300(9)
_cell_length_b	6.3052(5)
_cell_length_c	14.0798(10)
_cell_angle_alpha	90
_cell_angle_beta	90.886(5)
_cell_angle_gamma	90
_cell_volume	1085.60(14)
_cell_formula_units_Z	4
_cell_measurement_reflns_used	1457

_cell_measurement_temperature 273.15
_cell_measurement_theta_max 19.03
_cell_measurement_theta_min 2.19
_shelx_estimated_absorpt_T_max 0.995
_shelx_estimated_absorpt_T_min 0.985
_exptl_absorpt_coefficient_mu 0.101
_exptl_crystal_colour 'light yellow'
_exptl_crystal_colour_primary yellow
_exptl_crystal_density_diffrn 1.347
_exptl_crystal_description plate
_exptl_crystal_F_000 464
_exptl_crystal_size_max 0.15
_exptl_crystal_size_mid 0.12
_exptl_crystal_size_min 0.05
_diffrn_reflns_av_R_equivalents 0.0709
_diffrn_reflns_av_unetI/netI 0.0487
_diffrn_reflns_Laue_measured_fraction_full 1.000
_diffrn_reflns_Laue_measured_fraction_max 1.000
_diffrn_reflns_limit_h_max 14
_diffrn_reflns_limit_h_min -14
_diffrn_reflns_limit_k_max 7
_diffrn_reflns_limit_k_min -7
_diffrn_reflns_limit_l_max 16
_diffrn_reflns_limit_l_min -16
_diffrn_reflns_number 15155
_diffrn_reflns_point_group_measured_fraction_full 1.000
_diffrn_reflns_point_group_measured_fraction_max 1.000
_diffrn_reflns_theta_full 24.999
_diffrn_reflns_theta_max 24.999
_diffrn_reflns_theta_min 2.189
_diffrn_ambient_temperature 273.15
_diffrn_measured_fraction_theta_full 1.000
_diffrn_measured_fraction_theta_max 1.000
_diffrn_measurement_device_type 'Bruker APEX-II CCD'
_diffrn_measurement_method '\f and \w scans'

_diffrn_radiation_type MoK\a
_diffrn_radiation_wavelength 0.71073
_diffrn_source_current 30.0
_diffrn_source_power 1.2
_diffrn_source_voltage 40.0

Compound **10b**

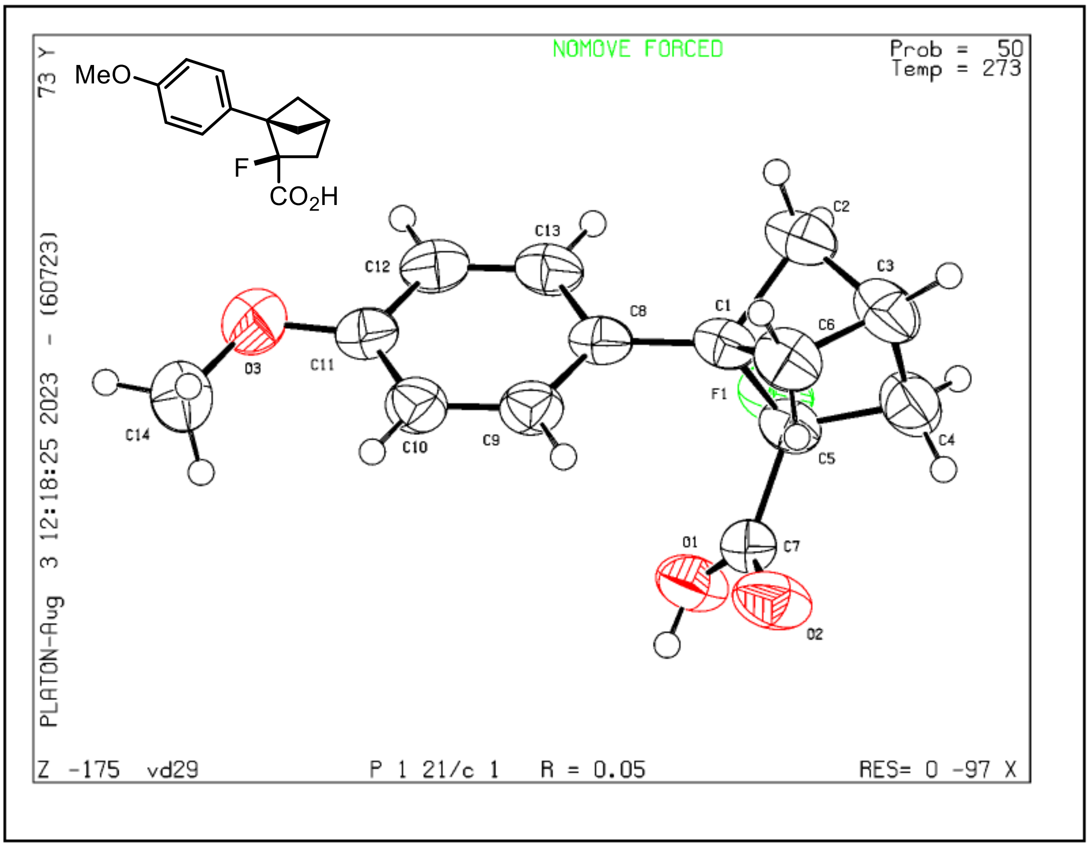

Figure S4. Molecular structure of **10b** according to X-Ray diffraction data. Thermal ellipsoids are shown at 50% probability level.

Crystal structure determination of 10b

data_vd29

_chemical_formula_sum	'C14 H15 F O3'
_chemical_formula_weight	250.26
_space_group_crystal_system	'monoclinic'
_space_group_IT_number	14
_space_group_name_H-M_alt	'P 1 21/c 1'
_space_group_name_Hall	'-P 2ybc'
_cell_length_a	9.5011(14)
_cell_length_b	11.9938(15)
_cell_length_c	11.5637(17)
_cell_angle_alpha	90
_cell_angle_beta	109.290(7)
_cell_angle_gamma	90
_cell_volume	1243.8(3)

_cell_formula_units_Z 4
_cell_measurement_reflns_used 3219
_cell_measurement_temperature 273.15
_cell_measurement_theta_max 22.30
_cell_measurement_theta_min 2.27
_shelx_estimated_absorpt_T_max 0.983
_shelx_estimated_absorpt_T_min 0.980
_exptl_absorpt_coefficient_mu 0.103
_exptl_crystal_colour colourless
_exptl_crystal_colour_primary colourless
_exptl_crystal_density_diffrn 1.336
_exptl_crystal_description block
_exptl_crystal_F_000 528
_exptl_crystal_size_max 0.2
_exptl_crystal_size_mid 0.18
_exptl_crystal_size_min 0.17
_diffrn_reflns_av_R_equivalents 0.0574
_diffrn_reflns_av_unetI/netI 0.0348
_diffrn_reflns_Laue_measured_fraction_full 1.000
_diffrn_reflns_Laue_measured_fraction_max 1.000
_diffrn_reflns_limit_h_max 11
_diffrn_reflns_limit_h_min -11
_diffrn_reflns_limit_k_max 14
_diffrn_reflns_limit_k_min -14
_diffrn_reflns_limit_l_max 13
_diffrn_reflns_limit_l_min -13
_diffrn_reflns_number 15158
_diffrn_reflns_point_group_measured_fraction_full 1.000
_diffrn_reflns_point_group_measured_fraction_max 1.000
_diffrn_reflns_theta_full 25.000
_diffrn_reflns_theta_max 25.000
_diffrn_reflns_theta_min 2.271
_diffrn_ambient_temperature 273.15
_diffrn_measured_fraction_theta_full 1.000
_diffrn_measured_fraction_theta_max 1.000

_diffrn_measurement_device_type   'Bruker APEX-II CCD'

_diffrn_measurement_method   '\f and \w scans'

_diffrn_radiation_type   MoK\a

_diffrn_radiation_wavelength   0.71073

_diffrn_source_current   30.0

_diffrn_source_power   1.2

_diffrn_source_voltage   40.0

Compound **12b**

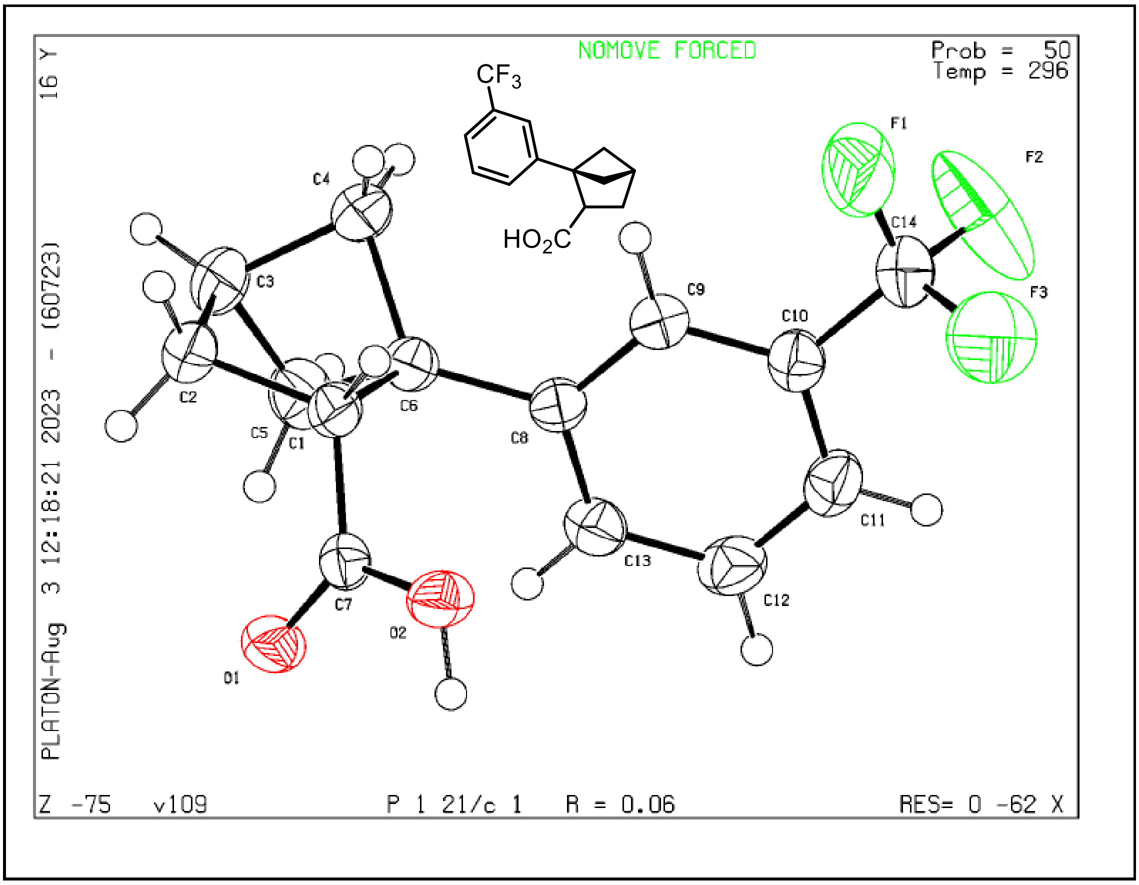

Figure S5. Molecular structure of **12b** according to X-Ray diffraction data. Thermal ellipsoids are shown at 50% probability level.

Crystal structure determination of 12b

data_v109

_chemical_formula_sum	'C14 H13 F3 O2'
_chemical_formula_weight	270.24
_space_group_crystal_system	'monoclinic'
_space_group_IT_number	14
_space_group_name_H-M_alt	'P 1 21/c 1'
_space_group_name_Hall	'-P 2ybc'
_cell_length_a	7.9641(4)
_cell_length_b	20.6944(9)
_cell_length_c	8.0024(4)
_cell_angle_alpha	90
_cell_angle_beta	104.190(3)
_cell_angle_gamma	90

_cell_volume 1278.65(11)

_cell_formula_units_Z 4

_cell_measurement_reflns_used 3974

_cell_measurement_temperature 296.15

_cell_measurement_theta_max 21.82

_cell_measurement_theta_min 2.64

_shelx_estimated_absorpt_T_max 0.981

_shelx_estimated_absorpt_T_min 0.973

_exptl_absorpt_coefficient_mu 0.122

_exptl_absorpt_correction_type none

_exptl_crystal_colour colourless

_exptl_crystal_colour_primary colourless

_exptl_crystal_density_diffrn 1.404

_exptl_crystal_description block

_exptl_crystal_F_000 560

_exptl_crystal_size_max 0.23

_exptl_crystal_size_mid 0.18

_exptl_crystal_size_min 0.16

_diffrn_reflns_av_R_equivalents 0.0411

_diffrn_reflns_av_unetI/netI 0.0254

_diffrn_reflns_Laue_measured_fraction_full 1.000

_diffrn_reflns_Laue_measured_fraction_max 1.000

_diffrn_reflns_limit_h_max 9

_diffrn_reflns_limit_h_min -9

_diffrn_reflns_limit_k_max 24

_diffrn_reflns_limit_k_min -24

_diffrn_reflns_limit_l_max 9

_diffrn_reflns_limit_l_min -9

_diffrn_reflns_number 17996

_diffrn_reflns_point_group_measured_fraction_full 1.000

_diffrn_reflns_point_group_measured_fraction_max 1.000

_diffrn_reflns_theta_full 24.999

_diffrn_reflns_theta_max 24.999

_diffrn_reflns_theta_min 1.968

_diffrn_ambient_temperature 296.15

_diffrn_measured_fraction_theta_full 1.000

_diffrn_measured_fraction_theta_max 1.000

_diffrn_measurement_device_type 'Bruker APEX-II CCD'

_diffrn_measurement_method '\f and \w scans'

_diffrn_radiation_type MoK\a

_diffrn_radiation_wavelength 0.71073

_diffrn_source_current 30.0

_diffrn_source_power 1.2

_diffrn_source_voltage 40.0

Compound **28**

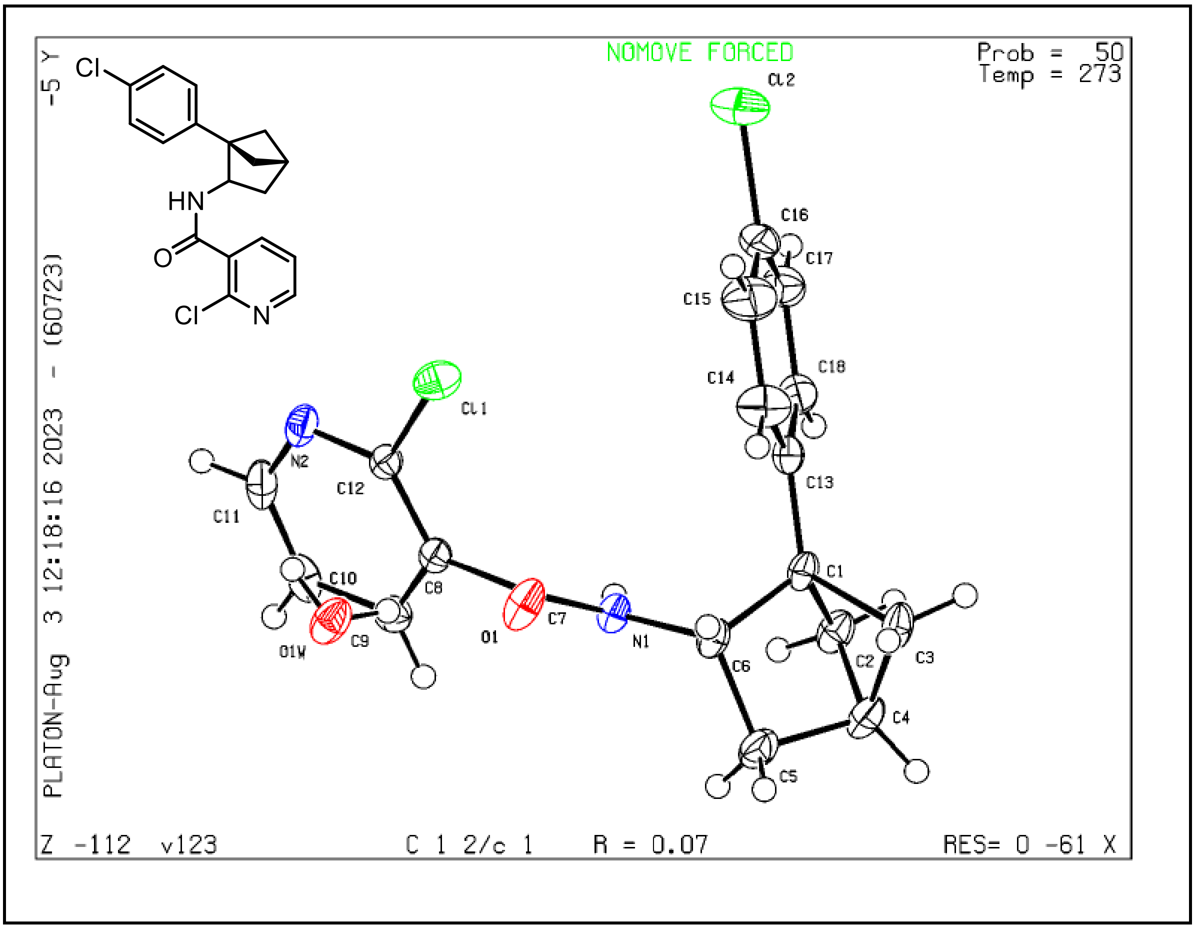

Figure S6. Molecular structure of **28** according to X-Ray diffraction data. Thermal ellipsoids are shown at 50% probability level.

Crystal structure determination of 28

data_v123

_chemical_formula_sum 'C9 H9 Cl N O'

_chemical_formula_weight 182.62

_space_group_crystal_system 'monoclinic'

_space_group_IT_number 15

_space_group_name_H-M_alt 'C 1 2/c 1'

_space_group_name_Hall '-C 2yc'

_cell_length_a 18.3768(13)

_cell_length_b 6.9898(4)

_cell_length_c 27.6891(17)

_cell_angle_alpha 90

_cell_angle_beta 103.554(7)

_cell_angle_gamma 90

_cell_volume 3457.6(4)
_cell_formula_units_Z 16
_cell_measurement_reflns_used 9067
_cell_measurement_temperature 273.15
_cell_measurement_theta_max 30.55
_cell_measurement_theta_min 2.28
_shelx_estimated_absorpt_T_max 0.955
_shelx_estimated_absorpt_T_min 0.926
_exptl_absorpt_coefficient_mu 0.388
_exptl_absorpt_correction_type none
_exptl_crystal_colour colourless
_exptl_crystal_colour_primary colourless
_exptl_crystal_density_diffrn 1.403
_exptl_crystal_description block
_exptl_crystal_F_000 1520
_exptl_crystal_size_max 0.2
_exptl_crystal_size_mid 0.2
_exptl_crystal_size_min 0.12
_diffrn_reflns_av_R_equivalents 0.0833
_diffrn_reflns_av_unetI/netI 0.0575
_diffrn_reflns_Laue_measured_fraction_full 0.999
_diffrn_reflns_Laue_measured_fraction_max 0.997
_diffrn_reflns_limit_h_max 25
_diffrn_reflns_limit_h_min -25
_diffrn_reflns_limit_k_max 9
_diffrn_reflns_limit_k_min -9
_diffrn_reflns_limit_l_max 38
_diffrn_reflns_limit_l_min -38
_diffrn_reflns_number 29127
_diffrn_reflns_point_group_measured_fraction_full 0.999
_diffrn_reflns_point_group_measured_fraction_max 0.997
_diffrn_reflns_theta_full 25.242
_diffrn_reflns_theta_max 29.999
_diffrn_reflns_theta_min 2.280
_diffrn_ambient_temperature 273.15

_diffrn_measured_fraction_theta_full 0.999

_diffrn_measured_fraction_theta_max 0.997

_diffrn_measurement_device_type 'Bruker APEX-II CCD'

_diffrn_measurement_method '\f and \w scans'

_diffrn_radiation_type MoK\a

_diffrn_radiation_wavelength 0.71073

_diffrn_source_current 30.0

_diffrn_source_power 1.2

_diffrn_source_voltage 40.0

Compound **29**

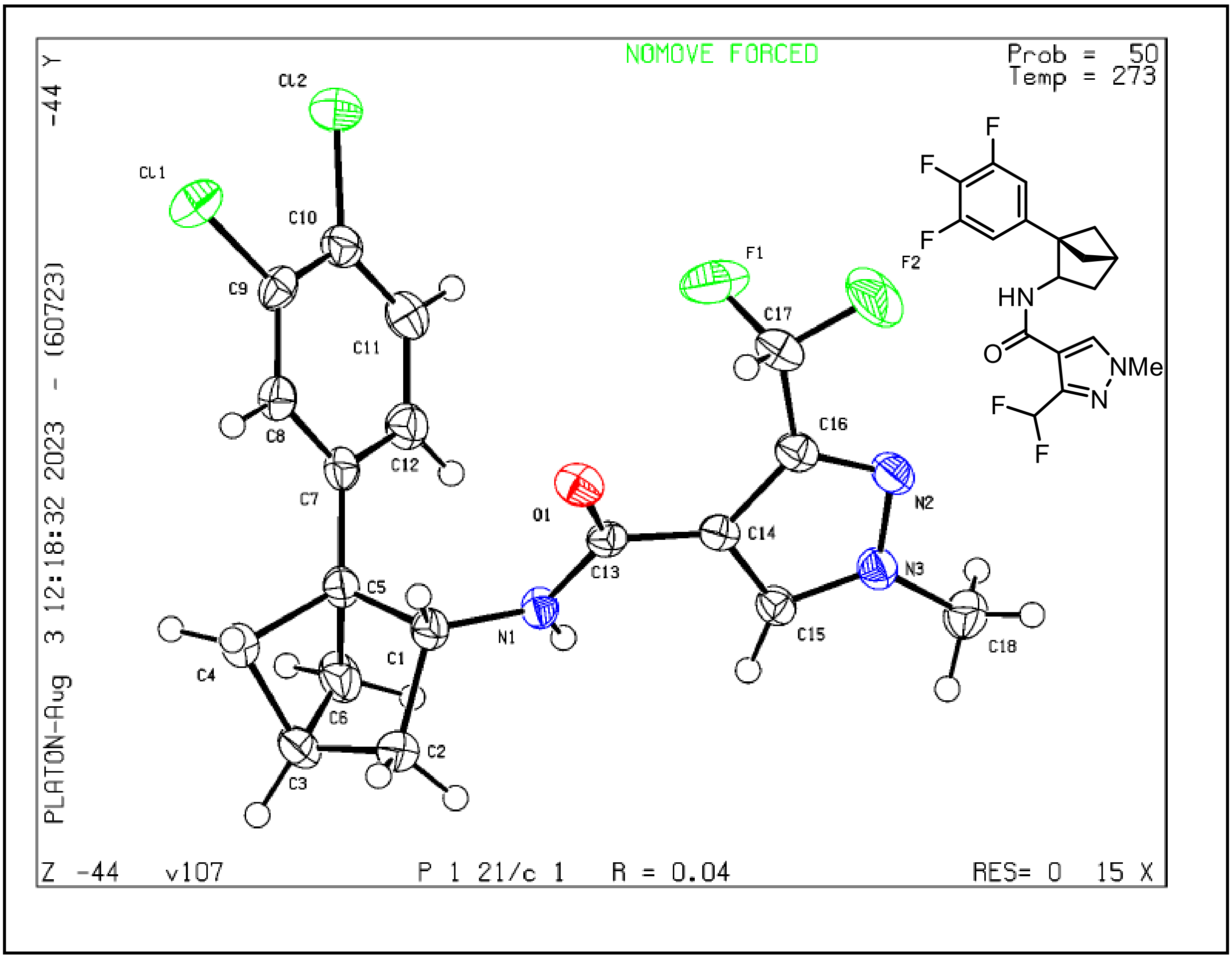

Figure S7. Molecular structure of **29** according to X-Ray diffraction data. Thermal ellipsoids are shown at 50% probability level.

Crystal structure determination of 29

data_v107

_chemical_formula_moiety	'C18 H17 Cl2 F2 N3 O'
_chemical_formula_weight	400.24
_space_group_crystal_system	'monoclinic'
_space_group_IT_number	14
_space_group_name_H-M_alt	'P 1 21/c 1'
_space_group_name_Hall	'-P 2ybc'
_cell_length_a	10.9193(6)
_cell_length_b	18.5629(12)
_cell_length_c	9.7993(5)
_cell_angle_alpha	90
_cell_angle_beta	115.272(3)

_cell_angle_gamma 90

_cell_volume 1796.16(18)

_cell_formula_units_Z 4

_cell_measurement_reflns_used 5641

_cell_measurement_temperature 273.15

_cell_measurement_theta_max 24.69

_cell_measurement_theta_min 2.19

_shelx_estimated_absorpt_T_max 0.984

_shelx_estimated_absorpt_T_min 0.911

_exptl_absorpt_coefficient_mu 0.394

_exptl_absorpt_correction_type none

_exptl_crystal_colour colourless

_exptl_crystal_colour_primary colourless

_exptl_crystal_density_diffrn 1.480

_exptl_crystal_description plate

_exptl_crystal_F_000 824

_exptl_crystal_size_max 0.24

_exptl_crystal_size_mid 0.17

_exptl_crystal_size_min 0.04

_diffrn_reflns_av_R_equivalents 0.0508

_diffrn_reflns_av_unetI/netI 0.0309

_diffrn_reflns_Laue_measured_fraction_full 1.000

_diffrn_reflns_Laue_measured_fraction_max 1.000

_diffrn_reflns_limit_h_max 12

_diffrn_reflns_limit_h_min -12

_diffrn_reflns_limit_k_max 22

_diffrn_reflns_limit_k_min -21

_diffrn_reflns_limit_l_max 11

_diffrn_reflns_limit_l_min -11

_diffrn_reflns_number 26745

_diffrn_reflns_point_group_measured_fraction_full 1.000

_diffrn_reflns_point_group_measured_fraction_max 1.000

_diffrn_reflns_theta_full 24.998

_diffrn_reflns_theta_max 24.998

_diffrn_reflns_theta_min 2.062

_diffrn_ambient_temperature 273.15

_diffrn_measured_fraction_theta_full 1.000

_diffrn_measured_fraction_theta_max 1.000

_diffrn_measurement_device_type 'Bruker APEX-II CCD'

_diffrn_measurement_method '\f and \w scans'

_diffrn_radiation_type MoK\a

_diffrn_radiation_wavelength 0.71073

_diffrn_source_current 30.0

_diffrn_source_power 1.2

_diffrn_source_voltage 40.0

Analysis of Aqueous Solubility

Test articles EN300-45199999 (**29**), EN300-45177924 (**27**), EN300-43350880 (**29**), EN300-43350881 (**30**), EN300-7392435 (**Conivaptan**), EN300-20331690 (**Lomitapide**), EN300-264529 (**Fluxapyroxad**), EN300-7394812 (**Boscalid**), EN300-20335148 (**Bixafen**) and reference compound (Ondansetron) were assessed for kinetic solubility in phosphate-buffered saline, pH 7.4.

Reagents and consumables

Phosphate buffered saline, pH 7.4 (Sigma-Aldrich, USA; Cat #P3813)
Acetonitrile Chromasolv, gradient grade, for HPLC, ≥99.9% (Sigma-Aldrich, USA; Cat #34851)
Methanol, for HPLC, ≥99.9% (Sigma-Aldrich, Cat #34860)
Ondansetron base powder (Enamine, Ukraine, Cat # EN300-117273)
DMSO (Sigma-Aldrich, USA; Cat # 34869)
Costar 96 Well Assay Blocks (Corning, USA; Cat # 3958)
MultiScreen HTS 96 Well Filter Plates (Millipore, Ireland; Cat # MSSLBPC10)
UV-Star® 96 Well Microplate (Greiner Bio-One, Germany; Cat #655801)
Matrix Disposable pipette tips (ThermoScientific, USA; Cat ## 8041, 7622, 7321)
Flex-Tubes Microcentrifuge Tubes, 1.5ml (Eppendorf, Germany; Cat # 22364111)
Matrix Storage tubes, 1.4 ml (ThermoScientific, USA; Cat # 4247)
Phenomenex Luna® C18 HPLC column, 2.1x50 mm, 5 µm (Cat #5291-126)

Equipment

Water purification system Millipore Milli-Q Gradient A10 (Millipore, France)
Thermomixer R Block, 1.5 mL (Eppendorf, Germany; Cat # 5355)
Matrix Multichannel Electronic Pipette 2-125 µL, 5-250 µL, 15-1250 µL (Thermo Scientific, USA; Cat ## 2011, 2012, 2004)
SpectraMax Plus Microplate Reader (Molecular Devices, USA; Product # 02196)
Multi-Well Plate Vacuum Manifold (Pall Corporation, USA; Product # 5014)
Vacuum pump (Millipore, USA; Model # XX5500000)
Analytical System
The measurements were performed using SpectraMax Plus reader in UV-Vis mode. Acquisition and analysis of the data were performed using SoftMax Pro v.5.4 (Molecular Devices) and Excel 2010 data analysis software.

Analytical System

The measurements were performed using SpectraMax Paradigm reader in UV-Vis mode. Acquisition and analysis of the data were performed using SoftMax Pro v.5.4 (Molecular Devices) and Excel 2010 data analysis software.

Methods

Kinetic solubility assay was performed according to the Enamine's aqueous solubility SOP. Briefly, using a 20 mM stock solution of the compound in 100% DMSO dilutions were prepared to a theoretical concentration of 400 µM in duplicates in phosphate-buffered saline pH 7.4 (138 mM NaCl, 2.7 mM KCl, 10 mM K-phosphate) with 2% final DMSO. The experimental compound dilutions in PBS were further allowed to equilibrate at 25 °C on a thermostatic shaker for two hours and then filtered through HTS filter plates using a vacuum manifold. The filtrates of test compounds were diluted 2-fold with acetonitrile with 2% DMSO before measuring.

In parallel, compound dilutions in 50% acetonitrile/PBS were prepared to theoretical concentrations of 0 µM (blank), 10 µM, 25 µM, 50 µM, 100 µM, and 200 µM with 2% final DMSO to generate calibration curves. Ondansetron was used as reference compound to control proper assay performance. 200 µl of each sample was transferred to 96-well plate and measured in 230-550 nm range with 5 nm step. The effective range of this assay is approximately 2-400 µM and the compounds returning values close to the upper limit of the range may have higher actual solubility (e.g. 5'-deoxy-5-fluorouridine). This method is not suitable for liquid (at 25 °C) substances (were not present among the tested compounds).

The concentrations of compounds in PBS filtrate are calculated using a dedicated Microsoft Excel calculation script. Proper absorbance wavelengths for calculations are selected for each compound manually based on absorbance maximums (absolute absorbance unit values for the minimum and maximum concentration points within the 0 – 3 OD range). Each final dataset is visually evaluated by the operator, and goodness of fit (R^2) is calculated for each calibration curve.

For EN300-43350880 **(29)** and EN300-43350881 **(30)** the calibration solutions and incubation samples were diluted 2-fold with acetonitrile containing internal standard and were analyzed using the HPLC system coupled with a tandem mass spectrometer. The effective range of this assay is approximately 2-400 µM (1-400 µM for EN300-43350880 **(29)** and EN300-43350881 **(30)**).

Results

The solubility data of the test and reference compounds are listed in the tables below. The calibration curves are shown in the Appendix*.

Table S1. Solubility data (1st batch)

Compound ID	PBS solubility, pH 7.4, µM			SE
	Incubation 1	Incubation 2	Mean	
Ondansetron	121	119	**120***	0.8
**EN300-7392435 (Conivaptan)**	4	7	**5**	1.5

Table S2. Solubility data (2st batch)

Compound ID	PBS solubility, pH 7.4, µM			SE
	Incubation 1	Incubation 2	Mean	
Ondansetron	126	126	**126****	0.1
**EN300-45199999 (26)**	15	14	**14**	0.4
**EN300-45177924 (27)**	18	19	**18**	0.4
**EN300-43359009 (28)**	35	34	**35**	0.5
**EN300-43350880 (29)**	4	4	**4**	0.1
**EN300-43350881 (30)**	25	28	**27**	1.5

Table S3. Solubility data (3st batch)

Compound ID	PBS solubility, pH 7.4, µM			SE
	Incubation 1	Incubation 2	Mean	
Ondansetron	120	116	**118****	1.2
**EN300-20331690 (Lomitapide)**	3	3	**3**	0.1
**EN300-264529 (Fluxapyroxad)**	24	27	**25**	1.1
**EN300-7394812 (Boscalid)**	9	13	**11**	2.0

Table S4. Solubility data (4nd batch)

Compound ID	PBS solubility, pH 7.4, µM			SE
	**Incubation 1**	**Incubation 2**	**Mean**	
Ondansetron	131	132	**132**	0.5
**EN300-20335148 (Bixafen)**	29	31	**30**	1.1

*Goodness of fit (R^2) in all titration curves as well as the variations between repeat measurements indicates high quality of the experimental data in the current batch of test articles.
**Ondansetron solubility data are consistent with previously obtained.

APPENDIX

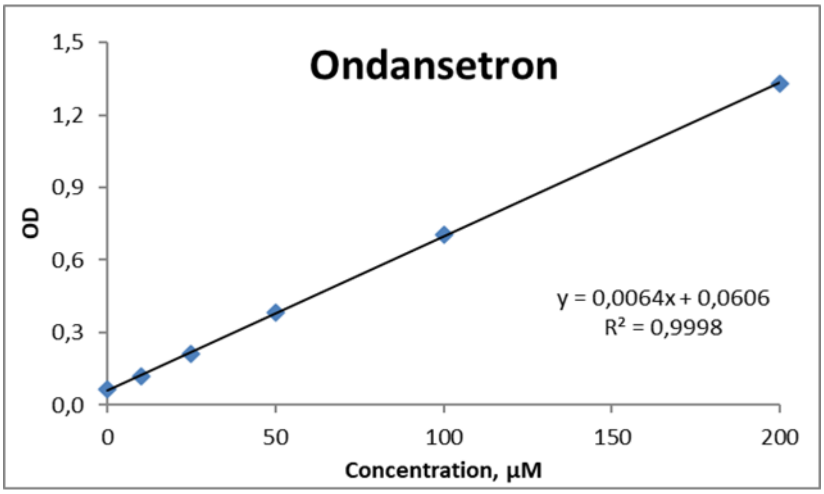

Figure S8. Calibration curve for **Ondansetron** (1st batch)

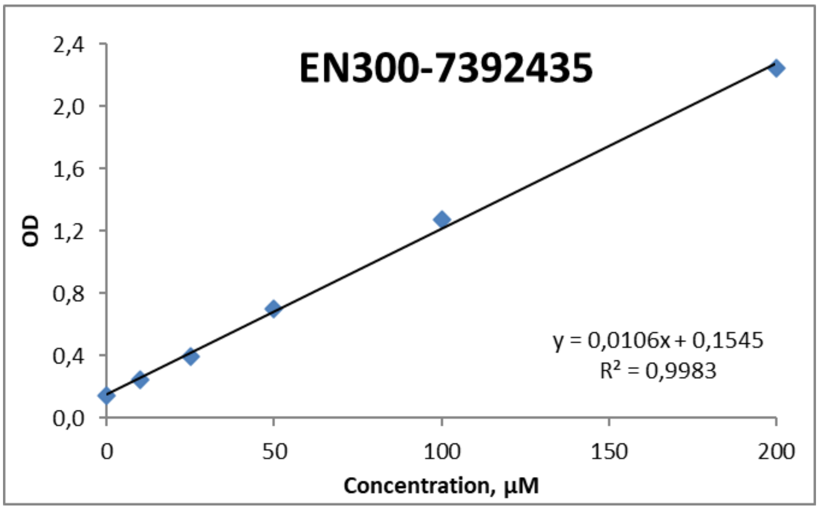

Figure S9. Calibration curve for **EN300-7392435 (Conivaptan)**

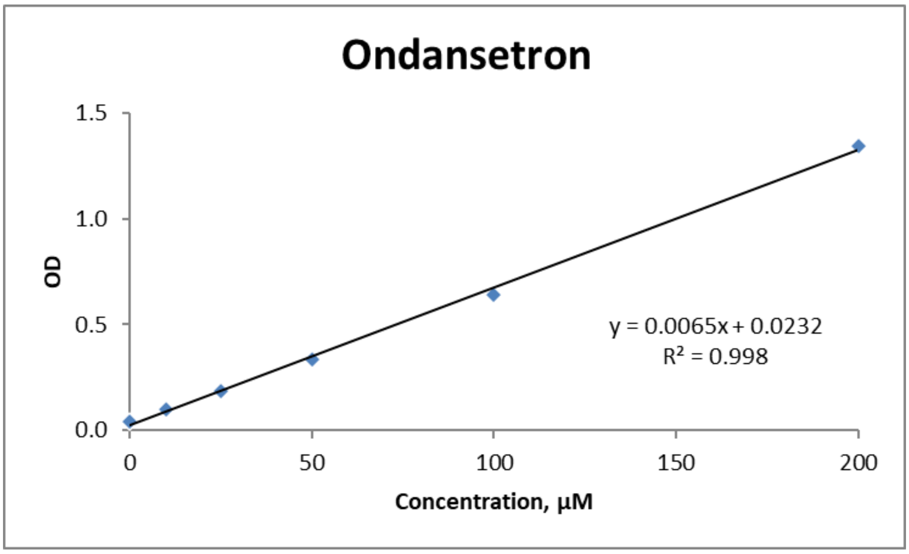

Figure S10. Calibration curve for **Ondansetron** (2st batch)

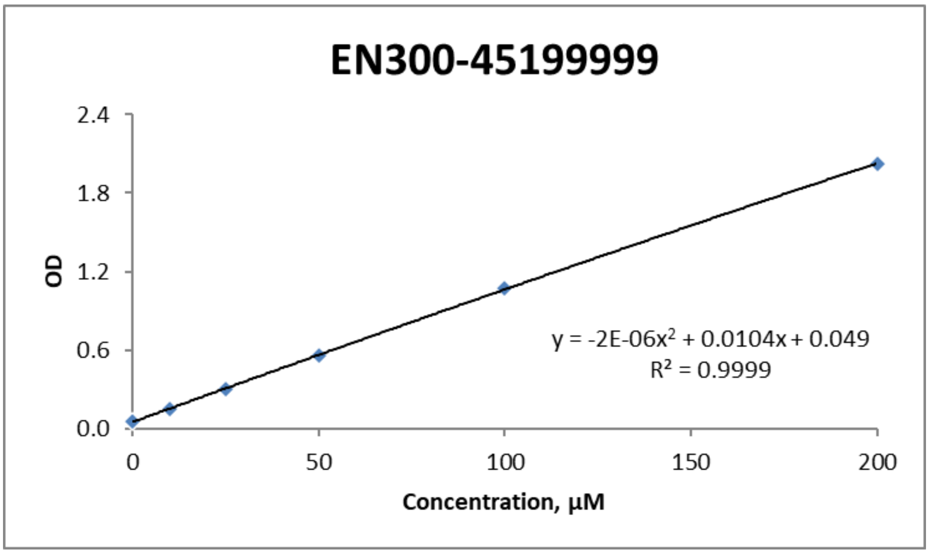

Figure S11. Calibration curve for **EN300-45199999 (26)**

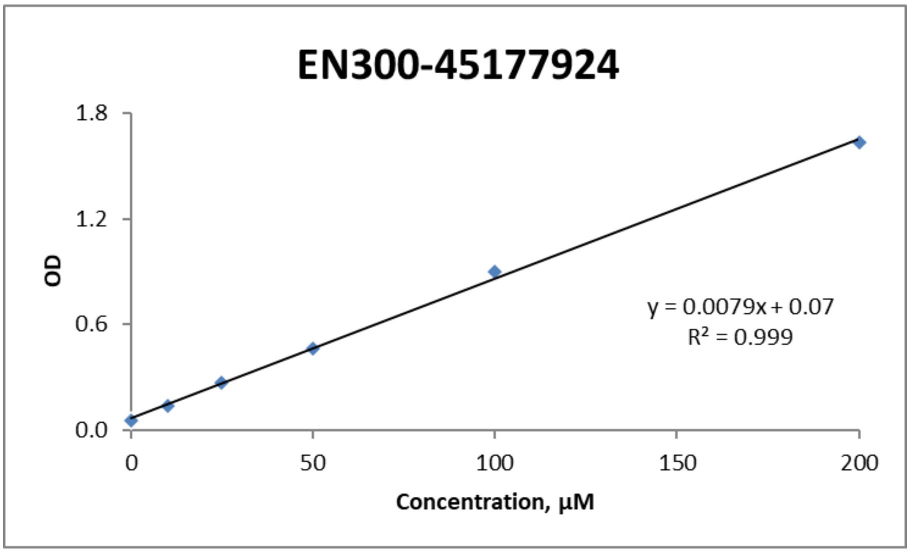

Figure S12. Calibration curve for **EN300-45177924 (27)**

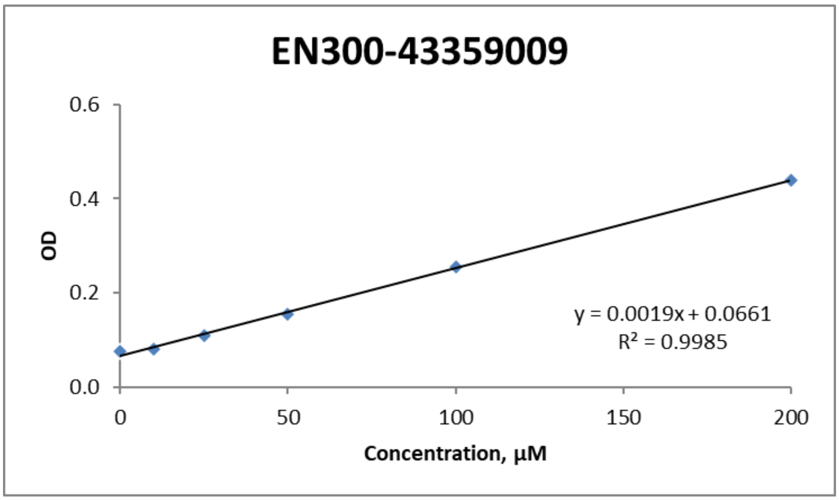

Figure S13. Calibration curve for **EN300-43359009 (28)**

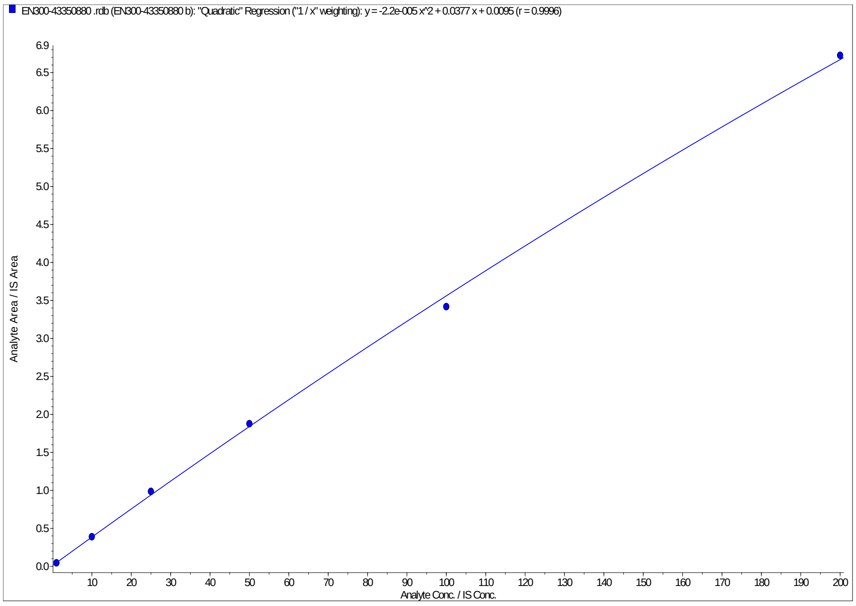

Figure S14. Calibration curve for **EN300-43350880 (29)**

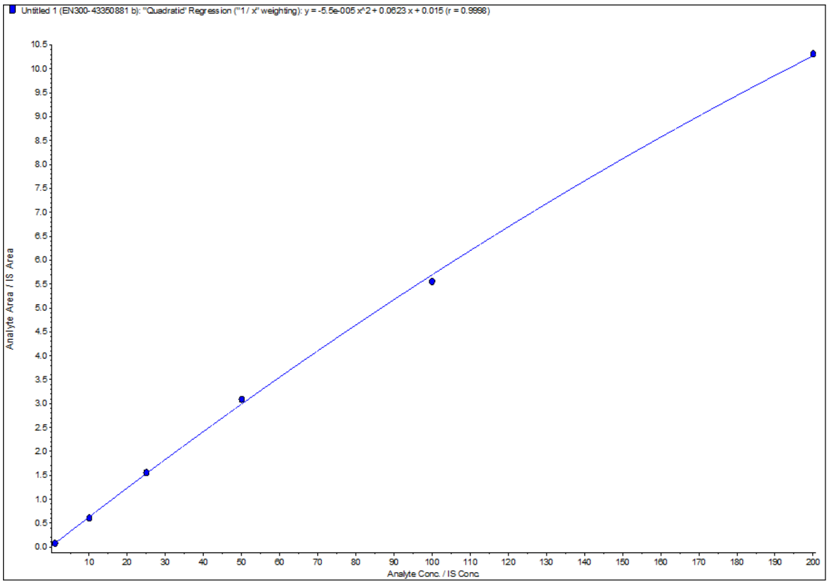

Figure S15. Calibration curve for **EN300-43350881 (30)**

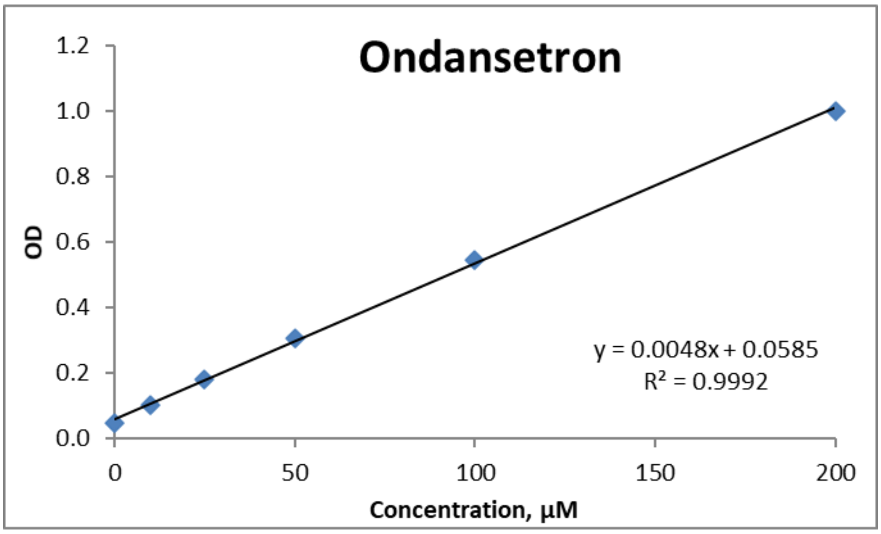

Figure S16. Calibration curve for **Ondansetron** (3st batch)

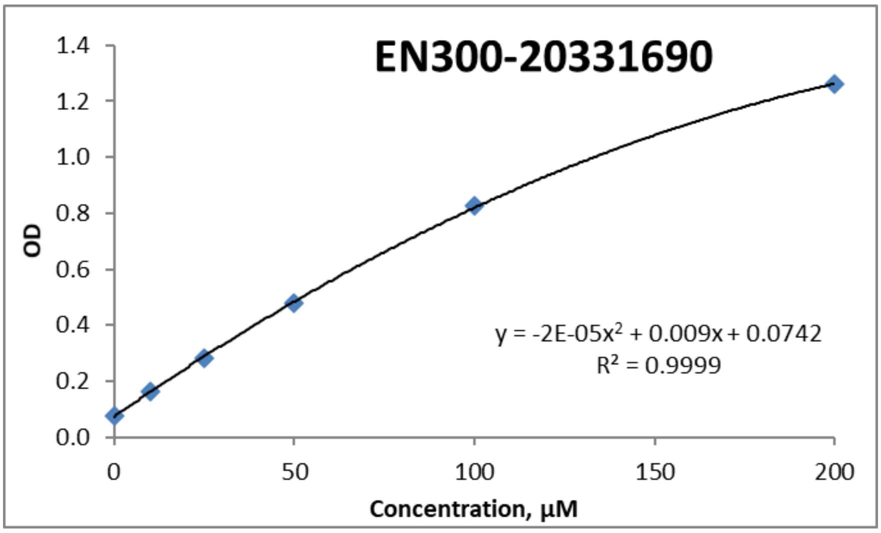

Figure S17. Calibration curve for **EN300-20331690 (Lomitapide)**

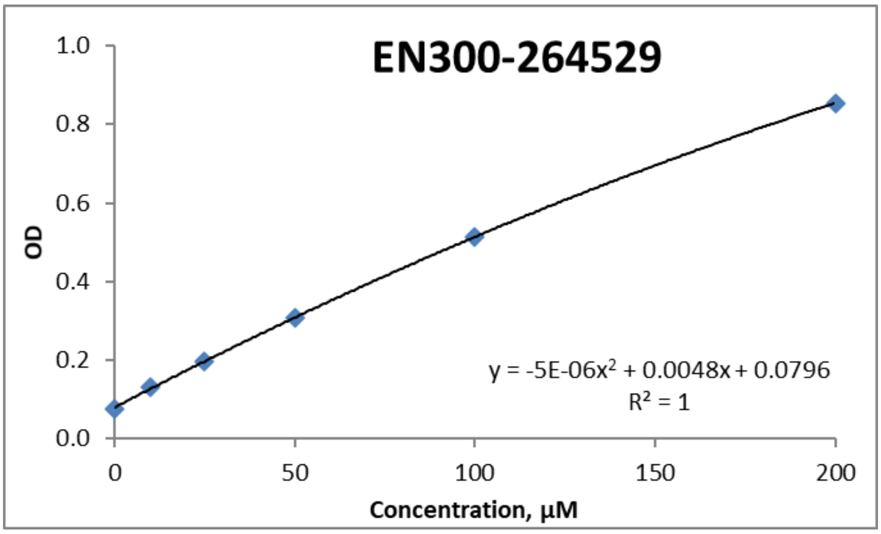

Figure S18. Calibration curve for **EN300-264529 (Fluxapyroxad)**

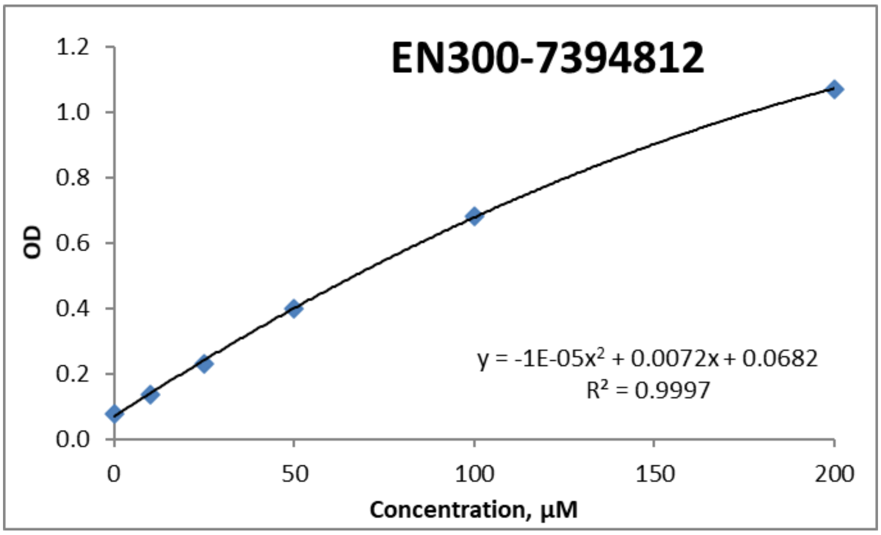

Figure S19. Calibration curve for **EN300-7394812 (Boscalid)**

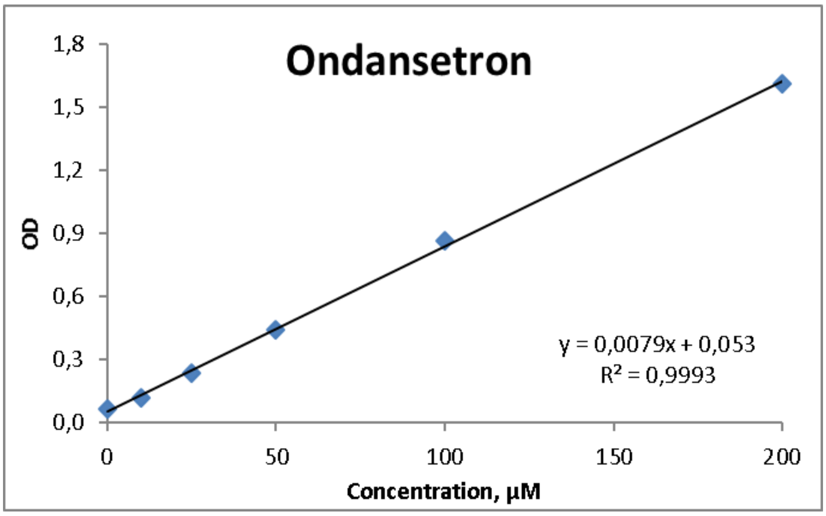

Figure S20. Calibration curve for **Ondansetron** (4nd batch)

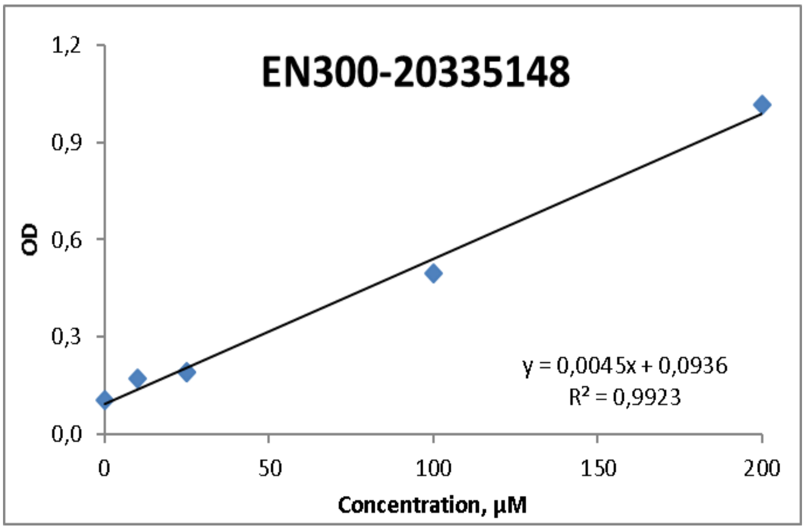

Figure S21. Calibration curve for **EN300-20335148 (Bixafen)**

Determination of Distribution Coefficient (LogD, pH 7.4)

Test articles EN300-45199999 (**26**), EN300-45177924 (**27**), EN300-43350880 (**29**), EN300-43350881 (**30**), EN300-7392435 (**Conivaptan**), EN300-20331690 (**Lomitapide**), EN300-264529 (**Fluxapyroxad**), EN300-7394812 (**Boscalid**), EN300-20335148 (**Bixafen**) and reference compound (Mebendazole) in *n*-octanol – phosphate buffered saline (PBS), pH 7.4. Distribution coefficient (or LogD) is a logarithm of the ratio of drug concentrations in two immiscible solvents, typically pH-buffered water and n-octanol. It is a measure of hydrophobic/hydrophilic properties of a given molecule. The partition of test compounds is determined using a shake-flask method, which involves mixing of a certain amount of the solute of interest in defined volumes of *n*-octanol and an aqueous buffer of choice followed by equilibration of the mixture by incubation with efficient mixing. Then, the distribution of the compounds in each solvent was controlled using LC-MS/MS.

Reagents and consumables

DMSO Chromasolv Plus, HPLC grade, ≥99.7% (Sigma-Aldrich, USA; Cat #34869)
Acetonitrile Chromasolv, gradient grade, for HPLC, ≥99.9% (Sigma-Aldrich, USA; Cat #34851)
Formic acid for mass spectrometry, ~98% (Fluka, USA; Cat #94318)
Phosphate buffered saline, tablet (Sigma-Aldrich, USA; Cat # P4417)
Acetic acid (Enamine, Ukraine)
1-Octanol ACS grade, ≥99% (Sigma-Aldrich, USA; Cat # 472328)
Mebendazole analytical standard, ≥98%, HPLC (Sigma-Aldrich, USA; Cat # M2523)
DMSO stock solutions of the test compounds 10mM
Phenomenex Luna® C18 HPLC column, 2.1 × 50 mm, 5 µm (Cat #5291-126)
1.1 mL microtubes in microracks, pipettor tips (Thermo Scientific, USA).
National Scientific MicroTube™ Rack (Thermo Fisher Scientific, USA; Cat # TN094612R)

Equipment

Gradient HPLC system (Shimadzu, Japan)
Triple quadrapole mass-detector API 3000 with TurboIonSpray Ion Source (AB Sciex, Canada)
VWR Membrane Nitrogen Generators N2-04-L1466, nitrogen purity 99%+ (VWR, USA)
MTR22 Multi Mix Rotator (UNICO, USA)
Laboratory Centrifuge, Sigma 4-15C, Qiagen (SIGMA GmbH, Germany)
Water purification system Millipore Milli-Q Gradient A10 (Millipore, France)
Multichannel Electronic Pipettes 0.5-12.5 µL, 2-125 µL, 5-250 µL, 15-1250 µL, Matrix (Thermo Scientific, USA; Cat ## 2009, 2001, 2002, 2004)

Analytical System

All measurements were performed using a Shimadzu Prominence HPLC system including a vacuum degasser, gradient pumps, a reverse phase column, a column oven and an autosampler. Mass spectrometric analysis was performed using an API 4000 QTRAP mass spectrometer from Applied Biosystems/MDS Sciex (AB Sciex) with Turbo V ion source and TurboIonspray interface. The TurboIonSpray ion source was used in both positive and negative ion modes. Acquisition and analysis of the data were performed using Analyst 1.6.3 software.

Methods

Incubations were carried out in Eppendorf-type polypropylene microtubes in triplicates. A 2.5 μL aliquot of 20 mM DMSO stock of a test compound was added into the previously mutually saturated mixture containing 500 μL of PBS (pH 7.4) and 500 μL of octanol. The solution was allowed to mix in a rotator for 1 hour at 30 rpm. Phase separation was assured by centrifugation for 2 min at 6000 rpm. The octanol phase was diluted 100-fold with 40% acetonitrile, and the aqueous phase (PBS buffer) was diluted 10-fold with 40% acetonitrile. The samples (both phases) were analyzed using an HPLC system coupled with a tandem mass spectrometer. Mebendazole was used as a reference compound.

Calculations of the partition ratios were carried out using the equation below.

$$D = \frac{d_o \cdot S_O}{d_p \cdot S_P}$$

where: S_0 – peak area of the analyte in octanol phase

S_P – peak area of the analyte in PBS buffer

d_o – dilution coefficient for octanol phase

d_p – dilution coefficient for aqueous phase

Results

LogD data for the reference compound (Mebendazole) and test compounds are provided in the table below.

Table S5. Experimental LogD, pH 7.4

Compound ID	Injection	S_P	S_O	D	LogD, pH 7.4	
**Mebendazole**	1	4.95E+04	3.26E+06	6.59E+02	2.819	**2.9**
	2	6.08E+04	4.83E+06	7.94E+02	2.901	
	3	6.54E+04	5.15E+06	7.87E+02	2.897	
**EN300-7392435 (Conivaptan)**	1	1.22E+04	2.41E+06	1.98E+04	4.30	**4.31**
	2	1.30E+04	2.75E+06	2.11E+04	4.33	
	3	1.35E+04	2.68E+06	1.99E+04	4.30	
**EN300-45199999 (26)**	1	2.88E+04	2.75E+06	9.55E+03	3.980	**4.1**
	2	3.43E+04	4.29E+06	1.25E+04	4.098	
	3	3.37E+04	4.57E+06	1.36E+04	4.133	
**EN300-20331690 (Lomitapide)**	1	4.09E-02	2.45E+05	5.98E+08	8.78	**6.39***
	2	5.92E+01	2.03E+05	3.42E+05	5.54	
	3	2.49E+02	1.69E+05	6.79E+04	4.83	
**EN300-45177924 (27)**	1	2.63E+03	2.05E+06	7.79E+03	3.892	**3.9**
	2	2.56E+03	1.91E+06	7.46E+03	3.873	
	3	2.66E+03	2.18E+06	8.20E+03	3.914	
**EN300-7394812 (Boscalid)**	1	1.25E+04	4.53E+05	3.62E+03	3.56	**3.55**
	2	1.53E+04	4.37E+05	2.86E+03	3.46	
	3	1.18E+04	4.81E+05	4.07E+03	3.61	
**EN300-43359009 (28)**	1	4.19E+03	1.61E+06	3.84E+03	3.585	**3.6**
	2	4.28E+03	1.63E+06	3.81E+03	3.581	
	3	4.98E+03	1.74E+06	3.49E+03	3.544	

Compound ID	Injection	S_P	S_O	D	LogD, pH 7.4	
**EN300-20335148 (Bixafen)**	1	4.38E+02	5.40E+04	1.23E+04	4.09	**4.22**
	2	3.81E+02	5.82E+04	1.53E+04	4.18	
	3	2.59E+02	5.96E+04	2.30E+04	4.36	
**EN300-43350880 (29)**	1	3.34E+03	4.21E+06	1.26E+04	4.101	**4.2**
	2	3.94E+03	5.43E+06	1.38E+04	4.140	
	3	3.09E+03	5.93E+06	1.92E+04	4.284	
**EN300-264529 (Fluxapyroxad)**	1	2.45E+03	8.27E+04	3.37E+03	3.53	**3.51**
	2	2.31E+03	6.90E+04	2.99E+03	3.48	
	3	2.56E+03	8.29E+04	3.24E+03	3.51	
**EN300-43350881 (30)**	1	5.90E+03	2.41E+06	4.08E+03	3.612	**3.6**
	2	5.91E+03	2.43E+06	4.11E+03	3.615	
	3	5.72E+03	2.54E+06	4.44E+03	3.648	

*Reliable measurable range is approximately -1 to 4.5

Assessment of Metabolic Stability in Human Liver Microsomes

The objective of this study was to determine metabolic stability of EN300-45199999 (**26**), EN300-45177924 (**27**), EN300-43350880 (**29**), EN300-43350881 (**30**), EN300-7392435 (**Conivaptan**), EN300-20331690 (**Lomitapide**), EN300-264529 (**Fluxapyroxad**), EN300-7394812 (**Boscalid**), EN300-20335148 (**Bixafen**) and and reference compounds in human liver microsomes at five time points over 40 minutes using HPLC-MS. Metabolic stability is defined as the percentage of parent compound lost over time in the presence of a metabolically active test system.

Materials

DMSO (Sigma-Aldrich, 34869 - Chromasolv Plus, for HPLC, ≥99.7%)

Acetonitrile (Sigma-Aldrich, 34851 - Chromasolv Plus, for HPLC, ≥99.9%)

K_2HPO_4 (Bio-Basic, Canada; Lot #MA7100050)

KH_2PO_4 (Bio-Basic, Canada; Lot #N9016010)

Magnesium chloride hexahydrate (Santa Cruz Biotechnology, Inc., USA; sc-203126A)

Human Liver Microsomes: pooled, mixed gender (XenoTech, H0630/lot N#1830003)

Glucose-6-phosphate dehydrogenase from baker's yeast, type XV (Sigma-Aldrich, USA; Cat #G6378)

D-Glucose-6-phosphate monosodium salt (Santa Cruz Biotechnology, Inc., USA; sc-210728)

NADPH tetrasodium salt (Biosynth, Cat # NN10871)

Formic acid (Sigma-Aldrich, USA; Cat #94318)

Verapamil hydrochloride (Sigma Aldrich, USA; Cat #V4629)

Niclosamide (Sigma-Aldrich, USA; Cat #N3510)

(+,-) Propranolol hydrochloride (Sigma-Aldrich, USA; Cat #P0884)

Diclofenac, 96% purity (Enamine, # EN300-119509)

Phenomenex Luna® C18 HPLC column, 2.1 × 50 mm, 5 μm (Cat #5291-126)

Matrix™ 0.75 mL blank tubes (Cat #4170), pipettor tips (Thermo Scientific).

Equipment

Gradient HPLC system (Shimadzu)

Triple quadrupole mass-detector API 5000 with Turbo V Ion Source (AB Sciex, Canada)

Nitrogen generator N2-04-L1466, nitrogen purity 99%+ (Whatman)

Incubator/Shaker Innova 4080 (New Brunswick Scientific, USA)

Water purification system Millipore Milli-Q Gradient A10 (Millipore, France)

Multichannel pipettors 1-30 μL, 2-125 μL, 30-850 μL (Thermo Scientific)

Analytical System

All measurements were performed using Shimadzu HPLC system including vacuum degasser, gradient pumps, reverse phase HPLC column, column oven, and autosampler. Mass spectrometric analysis was performed using a Triple quadrupole mass-detector API 5000 with Turbo V Ion Source (AB Sciex, Canada). The TurboIonSpray ion source was used in both positive and negative ion modes. The data acquisition and system control was performed using Analyst 1.6.3 software from AB Sciex.

Methods

Microsomal incubations were carried out in 96-well plates in 5 aliquots of 30 µL each (one for each time point). Liver microsomal incubation medium comprised of phosphate buffer (100 mM, pH 7.4), $MgCl_2$ (3.3 mM), NADPH (3 mM), glucose-6-phosphate (5.3 mM), glucose-6-phosphate dehydrogenase (0.67 units/mL) with 0.42 mg of liver microsomal protein per ml. In the control reactions the NADPH-cofactor system was substituted with phosphate buffer. Test compounds (2 µM, final solvent concentration 1.6%) were incubated with microsomes at 37 °C, shaking at 100 rpm. Each reaction was performed in duplicates. Five time points over 40 minutes were analyzed. The reactions were stopped by adding 5 volumes of acetonitrile containing internal standard to incubation aliquots, followed by protein sedimentation by centrifuging at 5500 rpm for 4 minutes. Supernatants were analyzed using the HPLC system coupled with tandem mass spectrometer.

The elimination constant (k_{el}), half-life ($t_{1/2}$) and intrinsic clearance (Cl_{int}) were determined in plot of ln(AUC) versus time, using linear regression analysis:[1]

$$k_{el} = -slope \qquad t_{1/2} = \frac{0.693}{k} \qquad Cl_{\text{int}} = \frac{0.693}{t_{1/2}} \times \frac{\mu l_{incubation}}{mg_{microsomes}}$$

[1] In order to indicate the quality of the linear regression analysis, the R (correlation coefficient) values are provided. In some cases, the last time point is excluded from the calculations to ensure acceptable logarithmic linearity of decay.

Results

Human microsomal stability data for reference and test compounds is provided in the table below.

Table S6. Human microsomal stability (1st batch)

Compound ID	Time, min	Peak Area Ratio		Peak Area Ratio, Mean of 2	% Remaining, Mean of 2	R	k_{el}, min^{-1}	$t_{1/2}$, min	Cl_{int}, µl/min/mg	% Remaining without cofactor, Mean of 2
		Inc. 1	Inc. 2							
1	2	3	4	5	6	7	8	9	10	11
Diclofenac human	0	3.59E-01	3.68E-01	3.63E-01	100	0.964	0.072	9.7	173	100
	7	1.84E-01	1.89E-01	1.86E-01	51	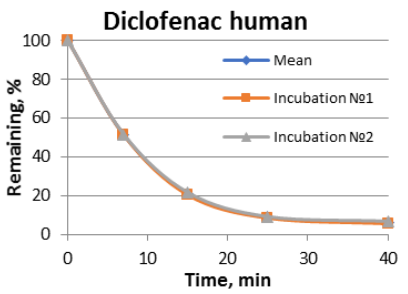				
	15	7.38E-02	7.91E-02	7.65E-02	21					
	25	3.12E-02	3.31E-02	3.21E-02	9					
	40	2.08E-02	2.44E-02	2.26E-02	6					103
Proprano-lol human	0	4.50E-01	4.53E-01	4.52E-01	100	0.976	0.010	70.9	24	100
	7	4.30E-01	4.66E-01	4.48E-01	99	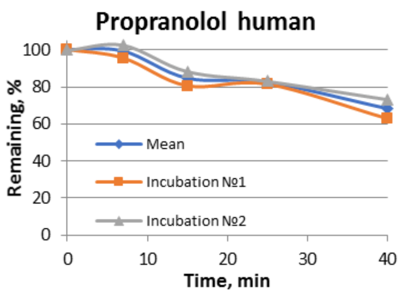				
	15	3.63E-01	4.01E-01	3.82E-01	85					
	25	3.67E-01	3.77E-01	3.72E-01	82					
	40	2.84E-01	3.32E-01	3.08E-01	68					105
**EN300-45177924 (27)** human	0	2.31E+01	2.41E+01	2.36E+01	100	0.983	0.077	9.0	186	100
	7	1.14E+01	1.07E+01	1.11E+01	47	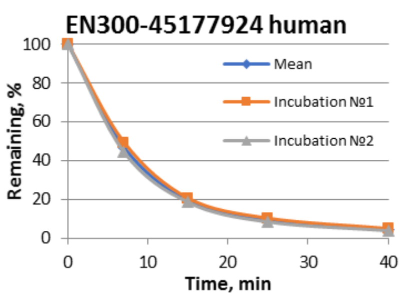				
	15	4.77E+00	4.50E+00	4.63E+00	20					
	25	2.39E+00	2.07E+00	2.23E+00	9					
	40	1.16E+00	9.82E-01	1.07E+00	5					106

1	2	3	4	5	6	7	8	9	10	11
**EN300-43350881 (30)** human	0	4.73E+00	4.17E+00	4.45E+00	100	0.966	0.025	27.3	61	100
	7	3.17E+00	3.09E+00	3.13E+00	70	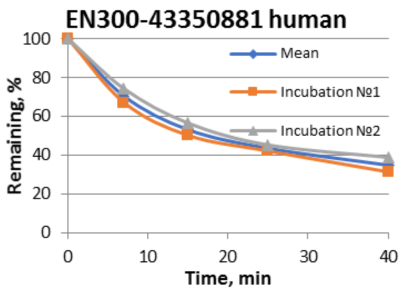				
	15	2.37E+00	2.35E+00	2.36E+00	53					
	25	1.99E+00	1.88E+00	1.94E+00	43					
	40	1.48E+00	1.61E+00	1.54E+00	35					109
**EN300-45199999 (26)** human	0	3.29E+01	3.14E+01	3.21E+01	100	0.776	0.005	134.8	12	100
	7	2.73E+01	2.50E+01	2.61E+01	81	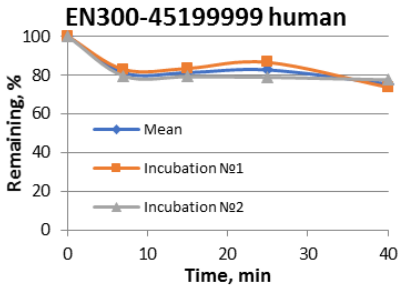				
	15	2.75E+01	2.49E+01	2.62E+01	81					
	25	2.85E+01	2.48E+01	2.66E+01	83					
	40	2.43E+01	2.44E+01	2.44E+01	76					109
**EN300-43350880 (29)** human	0	5.15E+00	4.95E+00	5.05E+00	100	0.987	0.015	47.4	35	100
	7	4.12E+00	4.25E+00	4.19E+00	83	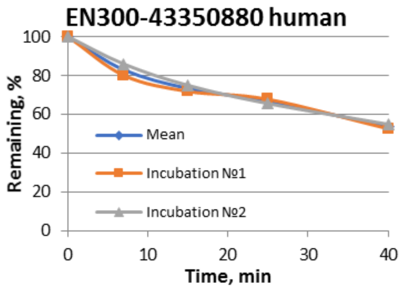				
	15	3.70E+00	3.71E+00	3.71E+00	73					
	25	3.49E+00	3.25E+00	3.37E+00	67					
	40	2.71E+00	2.72E+00	2.71E+00	54					106
**EN300-43359009 (28)** human	0	2.25E+00	2.21E+00	2.23E+00	100	0.996	0.012	57.6	29	100
	7	1.94E+00	2.00E+00	1.97E+00	88	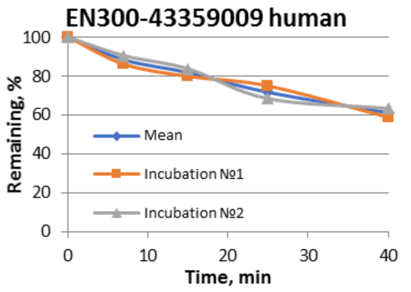				
	15	1.80E+00	1.85E+00	1.83E+00	82					
	25	1.69E+00	1.51E+00	1.60E+00	72					
	40	1.33E+00	1.40E+00	1.36E+00	61					116

Table S7. Human microsomal stability (2st batch)

Compound ID	Time, min	Peak Area Ratio		Peak Area Ratio, Mean of 2	% Remaining, Mean of 2	R	k_{el}, min^{-1}	$t_{1/2}$, min	Cl_{int}, µl/min/mg	% Remaining without cofactor, Mean of 2
		Inc. 1	Inc. 2							
1	2	3	4	5	6	7	8	9	10	11
Diclofenac human	0	9.19E-02	9.96E-02	9.58E-02	100	0.999	0.113	6.2	272	100
	7	4.17E-02	5.20E-02	4.69E-02	49	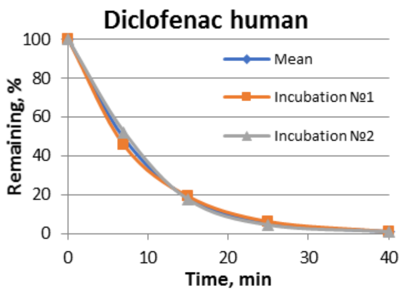				
	15	1.76E-02	1.76E-02	1.76E-02	18					
	25	5.80E-03	4.65E-03	5.22E-03	5					
	40	1.04E-03	1.21E-03	1.12E-03	1					91
Propranolol human	0	4.14E-02	3.63E-02	3.88E-02	100	0.980	0.015	46.7	36	100
	7	4.34E-02	3.55E-02	3.94E-02	102	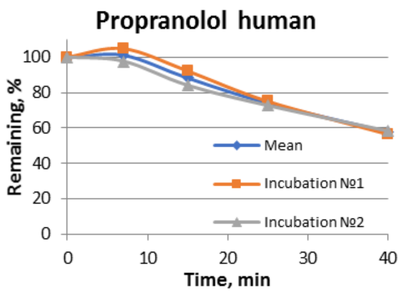				
	15	3.82E-02	3.06E-02	3.44E-02	89					
	25	3.11E-02	2.65E-02	2.88E-02	74					
	40	2.34E-02	2.13E-02	2.24E-02	58					96
**EN300-7392435 Conivaptan** human	0	4.47E-01	4.18E-01	4.32E-01	100	0.964	0.013	54.4	31	100
	7	4.14E-01	3.48E-01	3.81E-01	88	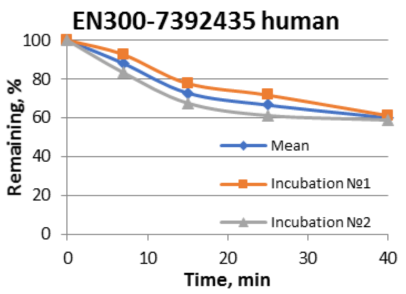				
	15	3.48E-01	2.82E-01	3.15E-01	73					
	25	3.21E-01	2.55E-01	2.88E-01	67					
	40	2.74E-01	2.45E-01	2.60E-01	60					

Table S8. Human microsomal stability (3st batch)

Compound ID	Time, min	Peak Area Ratio		Peak Area Ratio, Mean of 2	% Remaining, Mean of 2	R	k_{el} , min^{-1}	$t_{1/2}$, min	Cl_{int}, µl/min/mg	% Remaining without cofactor, Mean of 2
		Inc. 1	Inc. 2							
1	2	3	4	5	6	7	8	9	10	11
Diclofenac human	0	3.15E+00	3.07E+00	3.11E+00	100	0.995	0.118	5.9	285	100
	7	1.50E+00	1.67E+00	1.59E+00	51	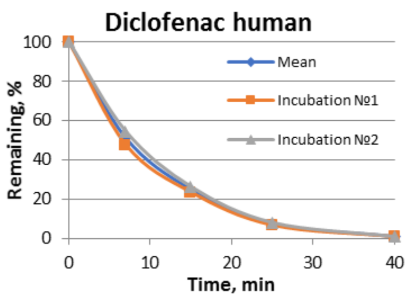				
	15	7.41E-01	8.05E-01	7.73E-01	25					
	25	2.13E-01	2.44E-01	2.29E-01	7					
	40	2.90E-02	2.60E-02	2.75E-02	1					90
Proprano-lol human	0	6.78E-01	6.54E-01	6.66E-01	100	0.959	0.007	95.7	17	100
	7	5.85E-01	6.07E-01	5.96E-01	89	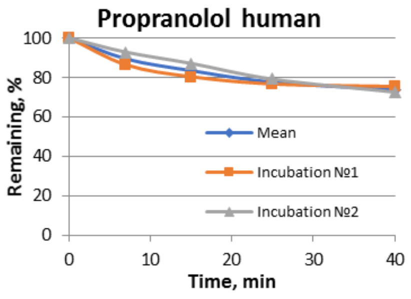				
	15	5.44E-01	5.70E-01	5.57E-01	84					
	25	5.19E-01	5.18E-01	5.19E-01	78					
	40	5.10E-01	4.75E-01	4.93E-01	74					84
**EN300-7394812 Boscalid** human	0	4.53E-02	4.46E-02	4.50E-02	100	0.994	0.011	63.8	26	100
	7	4.20E-02	4.35E-02	4.28E-02	95	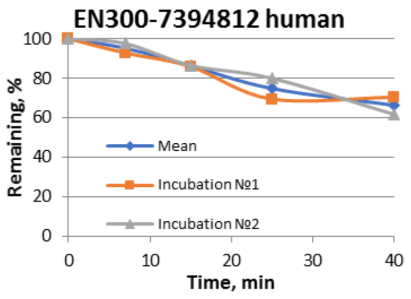				
	15	3.89E-02	3.86E-02	3.88E-02	86					
	25	3.14E-02	3.57E-02	3.36E-02	75					
	40	3.18E-02	2.76E-02	2.97E-02	66					59**

1	2	3	4	5	6	7	8	9	10	11
**EN300-264529 Fluxapyro-xad** human	0	9.53E-03	1.11E-02	1.03E-02	100	0.972	0.012	59.0	28	100
	7	1.01E-02	9.65E-03	9.88E-03	96	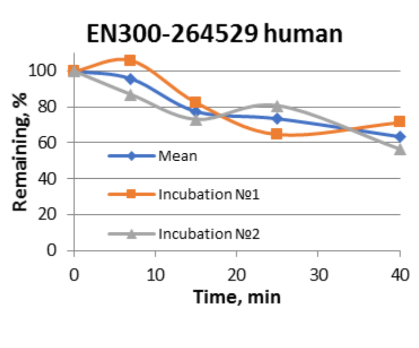				
	15	7.87E-03	8.10E-03	7.99E-03	77					
	25	6.19E-03	8.96E-03	7.58E-03	73					
	40	6.83E-03	6.25E-03	6.54E-03	63					79
**EN300-20331690 Lomitapide** human	0	3.38E-02	3.94E-02	3.66E-02	100	0.928	0.023	30.4	55	100
	7	3.17E-02	3.94E-02	3.56E-02	97	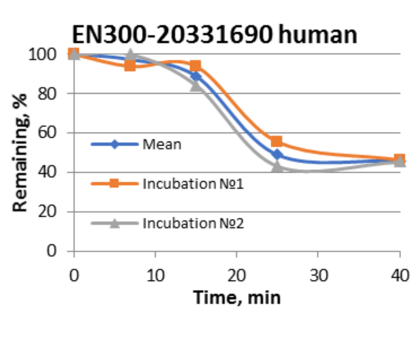				
	15	3.17E-02	3.32E-02	3.25E-02	89					
	25	1.88E-02	1.70E-02	1.79E-02	49					
	40	1.57E-02	1.79E-02	1.68E-02	46					88

Table S9. Human microsomal stability (4st batch)

Compound ID	Time, min	Peak Area Ratio		Peak Area Ratio, Mean of 2	% Remaining, Mean of 2	R	k_{el}, min^{-1}	$t_{1/2}$, min	Cl_{int}, µl/min/mg	% Remaining without cofactor, Mean of 2
		Inc. 1	Inc. 2							
1	2	3	4	5	6	7	8	9	10	11
Diclofenac human	0	1.00E+00	1.07E+00	1.04E+00	100	0.962	0.074	9.4	178	100
	7	5.83E-01	5.78E-01	5.81E-01	56	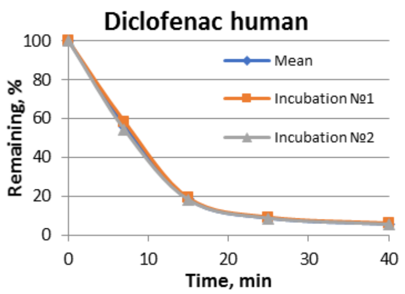				
	15	1.91E-01	1.93E-01	1.92E-01	19					
	25	9.27E-02	9.23E-02	9.25E-02	9					
	40	6.15E-02	5.84E-02	6.00E-02	6					97
Propranolol human	0	5.33E-01	5.47E-01	5.40E-01	100	0.715	0.002*	356.0*	5*	100
	7	5.54E-01	5.79E-01	5.67E-01	105	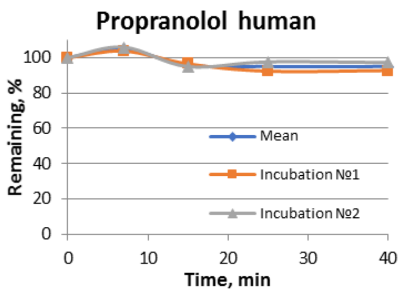				
	15	5.15E-01	5.21E-01	5.18E-01	96					
	25	4.92E-01	5.35E-01	5.14E-01	95					
	40	4.94E-01	5.33E-01	5.14E-01	95					89
**EN300-20335148 Bixafen** human	0	2.02E-01	2.20E-01	2.11E-01	100	0.919	0.004*	160.9*	10*	100
	7	2.11E-01	2.33E-01	2.22E-01	105	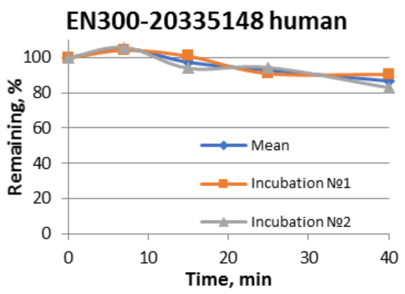				
	15	2.04E-01	2.07E-01	2.05E-01	97					
	25	1.84E-01	2.08E-01	1.96E-01	93					
	40	1.83E-01	1.83E-01	1.83E-01	87					101

*Parameter should be considered as approximate due to the high stability of the compound.
**"No cofactor" control data indicates that the instability of compound is partially or completely not determined by CYP450 activity

Interpretation of microsomal stability assay data

The test compounds can be classified in terms of their microsomal stability into low, medium and high clearance groups. The intrinsic clearance classification bands for mouse, rat, and human species are calculated according to the well stirred model equation:[1]

$$CL_{int} = \frac{CL_H}{fu \times (1 - E)}$$

where CL_H is a hepatic clearance (mL/min/kg), $CL_H = E \times Q_H$
Q_H = liver blood flow (mL/min/kg)[2]
E = extraction ratio, assumed at 0.3 for low clearance and at 0.7 for high clearance compounds
fu = fraction unbound in plasma, assumed at 1.

The CL_{int} classification values were calculated for mouse, rat, and human species using the literature data on liver weight[3] and microsomal protein concentration[3,4] and are represented in the following table.

Table S10. The intrinsic clearance groups for classification of test compounds

Classification group	Intrinsic clearance (µL/min/mg protein)		
	Mouse	Rat	Human
**Low clearance**	<8.6	<13	<8.8
**High clearance**	>48	>72	>48

[1]. Houston J.B., Utility of *in vitro* drug metabolism data in predicting *in vivo* metabolic clearance, Biochemical Pharmacology, **1994**, *47*, 1469-1479.

[2]. Davies B. and Morris T., Physiological parameters in laboratory animals and humans, *Pharmaceutical Research,* **1993**, *10*, 1093-1095.

[3]. Barter Z.E., *et al.*, Scaling factors for the extrapolation of *in vivo* metabolic drug clearance from *in vitro* data: reaching a consensus on values of human microsomal protein and hepatocellularity per gram of liver, *Current Drug Metabolism,* **2007**, *8,* 33-45.

[4]. Iwatsubo T., *et al.*, Prediction of species differences (rats, dogs, humans) in the *in vivo* metabolic clearance of YM796 by the liver from *in vitro* data, *Journal of Pharmacology and Experimental Therapeutics,* **1997**, *283*, 462-469.

Bioactivity

Antifungal activity of the synthetic compounds using disk diffusion methods

The compounds were tested for plant pathogens *Aspergillus niger* (strain VURV-F 822), which was received from Culture collection of microorganisms of Crop Research Institute (Prague, Czech Republic).

The synthetic compounds' antifungal activity was evaluated using a disk diffusion assay for testing filamentous fungi (CLSI M51-A) and broth microdilution antifungal susceptibility testing (EUCAST E.DEF 7.3.1; CLSI M38-A2)

[CLSI M51-A. Method for Antifungal Disk Diffusion Susceptibility Testing of Nondermatophyte Filamentous Fungi; Approved Guideline.CLSI document M51-A. Wayne, PA: Clinical and Laboratory Standards Institute; 2010. This method is standardized for Altenaria, Aspergillus, Bipolaris, Fusarium, order Mucorales, etc.]

[CLSI M38-A2. 2008. Reference method for broth dilution antifungal susceptibility testing of filamentous fungi, 2nd ed Approved standard M38-A2 Clinical and Laboratory Standards Institute, Wayne, PA.]

Antifungal disk diffusion method

The solutions of fungicides and their analogues were diluted in dimethyl sulfoxide (DMSO) to produce the following concentrations: 0.004, 0.008, 0.016, 0.031, 0.062, 0.125, 0.250, 0.5, 1, 2 mg/mL for each test component. Sterile paper disks (ASPECT, Ukraine) were soaked with 10 µl of a solution of the appropriate fungicide from the highest concentration to the lowest.

The fungal strains were cultured on Petri dishes with Saburo dextrose agar (Condalab, Spain) and incubated at 25 °C for 5 days. Sterile Dulbecco's phosphate-buffered saline (DPBS) with 0,1 % Tween 20 was used to cover colonies of fungus for obtained conidial suspension. Suspension of conidia was adjusted to inoculums 2×10^5 conidia/mL by sterile DPBS.

For antifungal disk diffusion test we used square Petri dishes (FALCON A Corning Brand, USA). The conidial suspension (0.5 mL) was added to Petri dishes with Saburo dextrose agar. The suspension was evenly distributed over the surface of the nutrient medium with a glass spatula. After the surface of the inoculated medium was dry, the disks with different concentrations of testing compounds were added to the Petri dishes. Each compound was tested in triplicate at different concentrations. Growth control was a disk with 10 µl of DMSO, which was used for test components dilution.

Petri dishes incubated at 25 °C for 72 h. The test compounds at known concentration into contact with an inoculated medium then exert a growth-inhibiting effect then a clear zone (the zone of

inhibition) appears around the test product. The diameter of a clear zone around the well is measured at the end of the incubation period in millimeters (disk diameter did not consider).

If the fungal strain is susceptible to the antifungal agent, then a zone of inhibition appears on the agar plate. If it is resistant to the test compound, then no zone is evident. The size of the zone of inhibition is usually related to the level of antifungal activity present in the compound – a larger zone of inhibition usually means that the antimicrobial is more potent.

The growth rate of all strains for each concentration of test compounds was determined visually and compared with the growth of control.

Broth microdilution antifungal susceptibility testing

A 96-well CELLSTAR plate (sterile, flat-bottomed, polystyrene, transparent) was used to determine MIC by broth microdilution method. Also, dabble straight RPMI-1640 medium with 2% glucose (2x RPMI 2%G) was used for this method. Spore suspension for plate inoculation was prepared according to the method described above for the disc diffusion method.

The each well in the plate contained 49 µl of medium (2x RPMI 2%G), 50 µl of inoculum (prepared in DPBS) and 1 µl of the stock solution of the test substance. Positive control wells contained 50 µl medium and 50 µl inoculum, and sterile control wells contained 50 µl medium and 50 µl DPBS.

To determine the MIC by the microdilution method, a series of stock solutions of fungicides and their analogues in DMSO with the following concentrations were prepared: 3.2 mg/mL, 1.6 mg/mL, 0.8 mg/mL, 0.4 mg/mL, 0.2 mg/mL, 0.1 mg/mL, 0.05 mg/mL, 0.025 mg/mL, 0.0125 mg/mL, 0.00625 mg/mL.

1 µl of the stock solutions of the test substances were added to each well to obtain the following final concentrations: 0.32 mg/mL, 0.16 mg/mL, 0.08 mg/mL, 0.04 mg/mL, 0.02 mg/mL, 0.01 mg/mL, 0.005 mg/mL, 0.0025 mg/mL, 0.00125 mg/mL, 0.000625 mg/mL.

Immediately after adding all the components to the plates, the optical density was measured at 490 nm on a spectrophotometer Safire2 (Tecan, Switzerland). After that, the plates were incubated at 28 °C for 48 hours and the optical density was measured again. The growth of the culture was determined as the difference between these dimensions.

Results

In the current research, we studied antifungal activity of three newly synthesized analogues of Fluxapyroxad, Boscalid, Bixafen, and compared their activity against 4 strains of fungi, relative to the original fungicides.

Aspergillus niger was to be the most sensitive strain to all tested compounds. However, its susceptibility to Fluxapyroxad, Boscalid and Bixafen was higher than their analogues (Fig. S22; Table. S12). Thus, in experiments with this strain of *A. niger*, the minimum inhibitory concentration of these three fungicides was 0.004 mg/mL. At this concentration, we observed small zones of inhibition, but it was noted the secondary mycelial growth in there (Fig. S23-S25). At the same time, the analogue of Fluxapyroxad was more effective than the analogue of Boscalid, which is evidenced by the larger size of the inhibition zones at higher concentrations of these compounds.

The broth microdilution method was not suitable for determining the MIC of Fluxapyroxad, Boscalid, and Bixafen analogues. This was due to two facts: 1) the test compounds did not inhibit growth by 100%, so visual reading of the plates was not possible; 2) the spectrometric reading of the results was not effective, the initial optical density (mixture of spores, fungicide analogues and medium) was higher than after 72 hours of cultivation, which was manifested as false positive results.

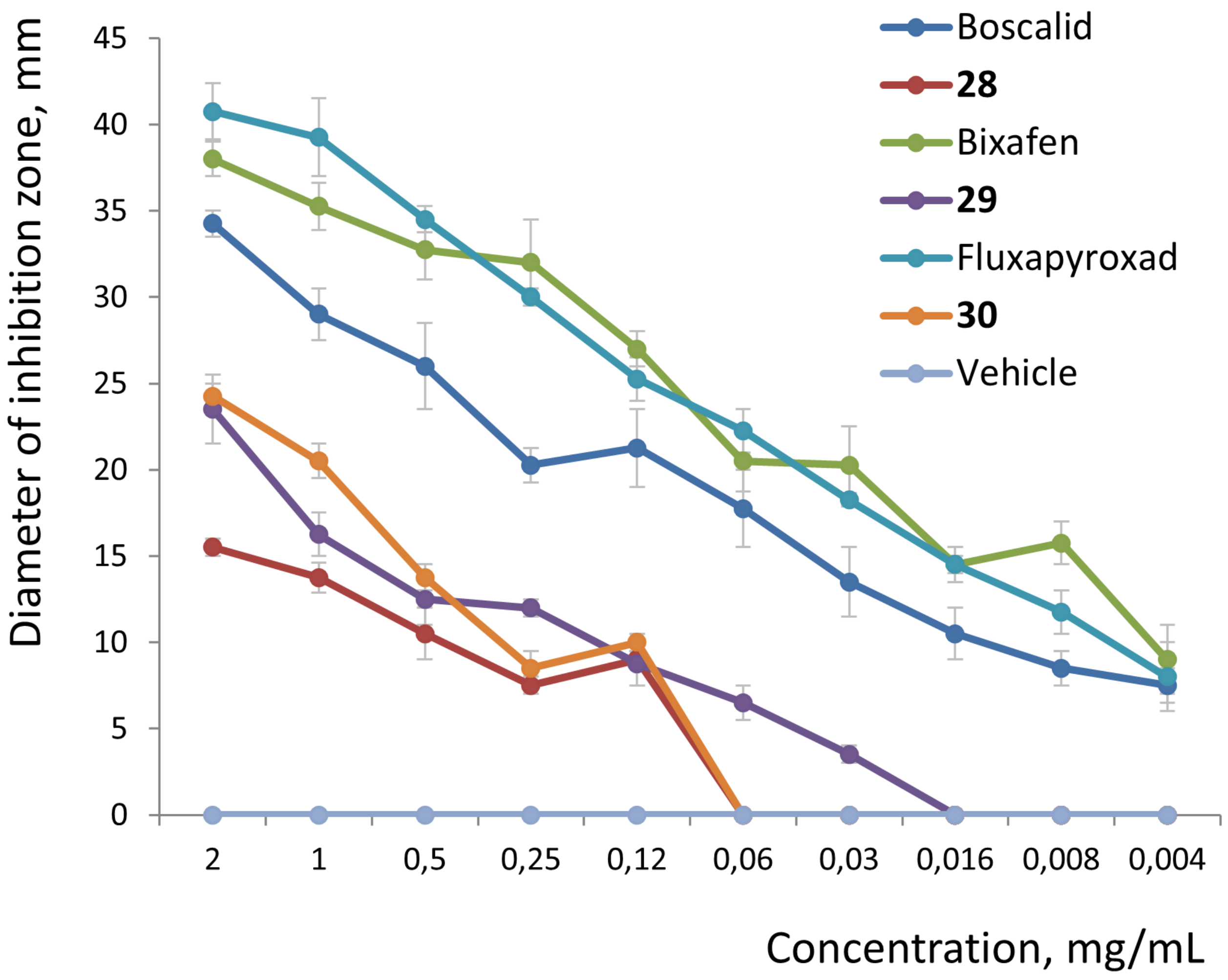

Figure S22. The antifungal activity of Boscalid, Bixafen, Fluxaporoxad, and their analogues **28-30** toward *Aspergillus niger* (strain VURV-F 822). Disk diffusion methods.

Table S11. The antifungal activity of studied compounds against *Aspergillus niger* (strain VURV-F 822)

Concentration mg/mL	Diameter of inhibition zone, mm					
	Boscalid	Analogue of Boscalid **(±)-28**	Fluxapyroxad	Analogue of Fluxapyroxad **(±)-30**	Bixafen	Analogue of Bixafen **(±)-29**
2	34.25±0.75	15.5±0.5	40.75±1.6	24.25±0.75	38±1	23.5±2
1	29±1.5	13.75±0.88	39.25±2.25	20.5±1	35.25±1.4	16.25±1.25
0.5	26±2.5	10.5±1.5	34.5±0.75	13.75±0.75	32.75±1.75	12.5±1.5
0.25	20.25±1	7.5±0.5 st	30±0.5	8.5±1	32±2.5	12±0.5
0.12	21.25±2.25	9±0.5 st	25.25±1.5	10±0.5	27±1	8.75±1.25
0.06	17.75±2.25	0	22.25±1.25 st	0	20.5±1.75 st	6.5±1 st
0.03	13.5±2	0	18.25±0.4	0	20.25±2.25	3.5±0.5 st
0.016	10.5±1.5	0	14.5±0.5	0	14.5±1	0
0.008	8.5±1	0	11.75±1.25	0	15.75±1.25	0
0.004	7.5±1 st	0	8±2 st	0	9±2 st	0

Abbreviation: 0 – absence of antifungal activity; st – static growth, when fungal mycelium has secondary growth

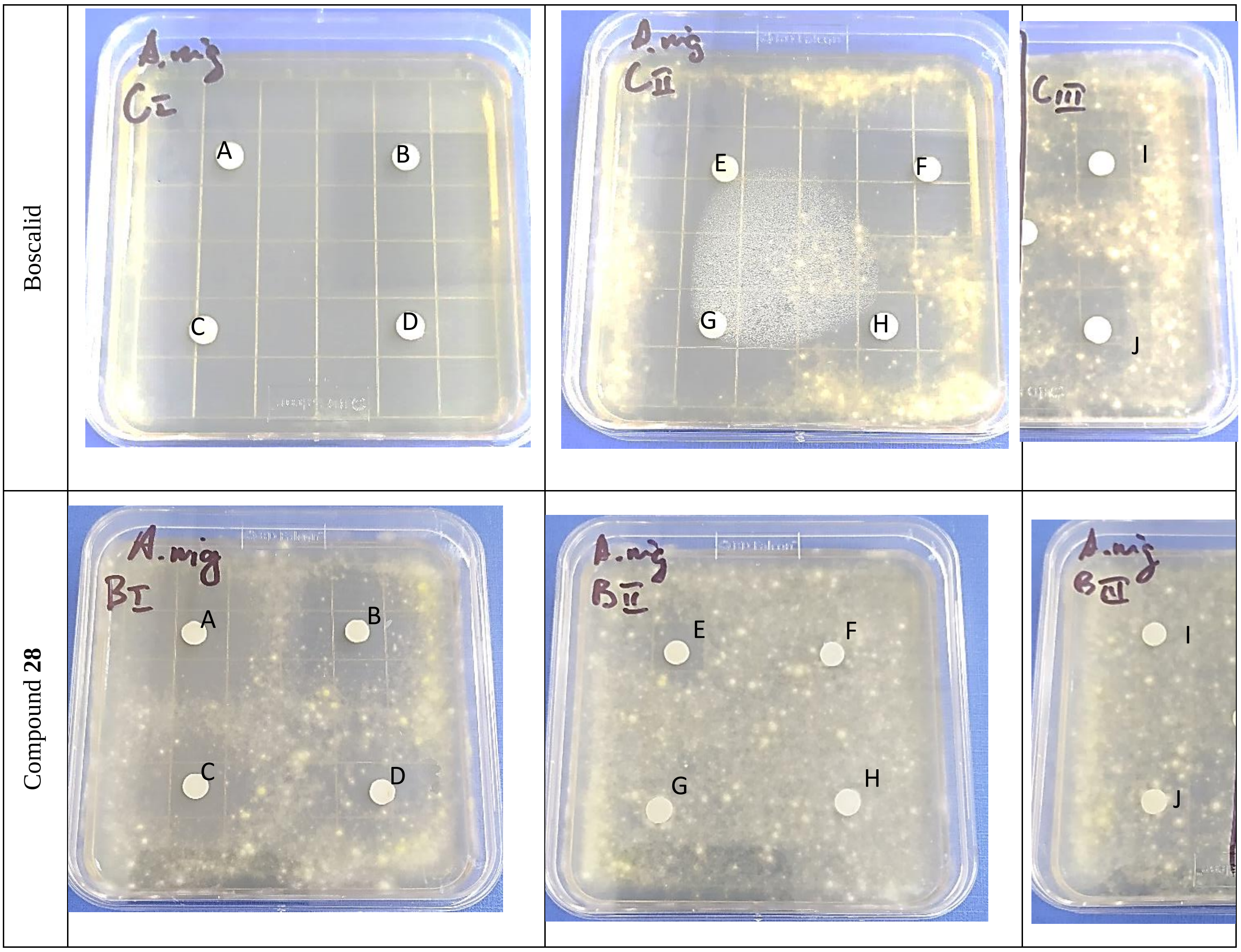

Figure S23. Activity of Boscalid and its analogue **(±)-28** toward *Aspergillus niger*, plates in 72 h. Concentration of compounds: A – 2 mg/mL; B – 1 mg/mL; C – 0.5 mg/mL; D – 0.25 mg/mL; E – 0.125 mg/mL; F – 0.06 mg/mL; G – 0.03 mg/mL; H – 0.016; I – 0.008 mg/mL; J – 0.004 mg/mL.

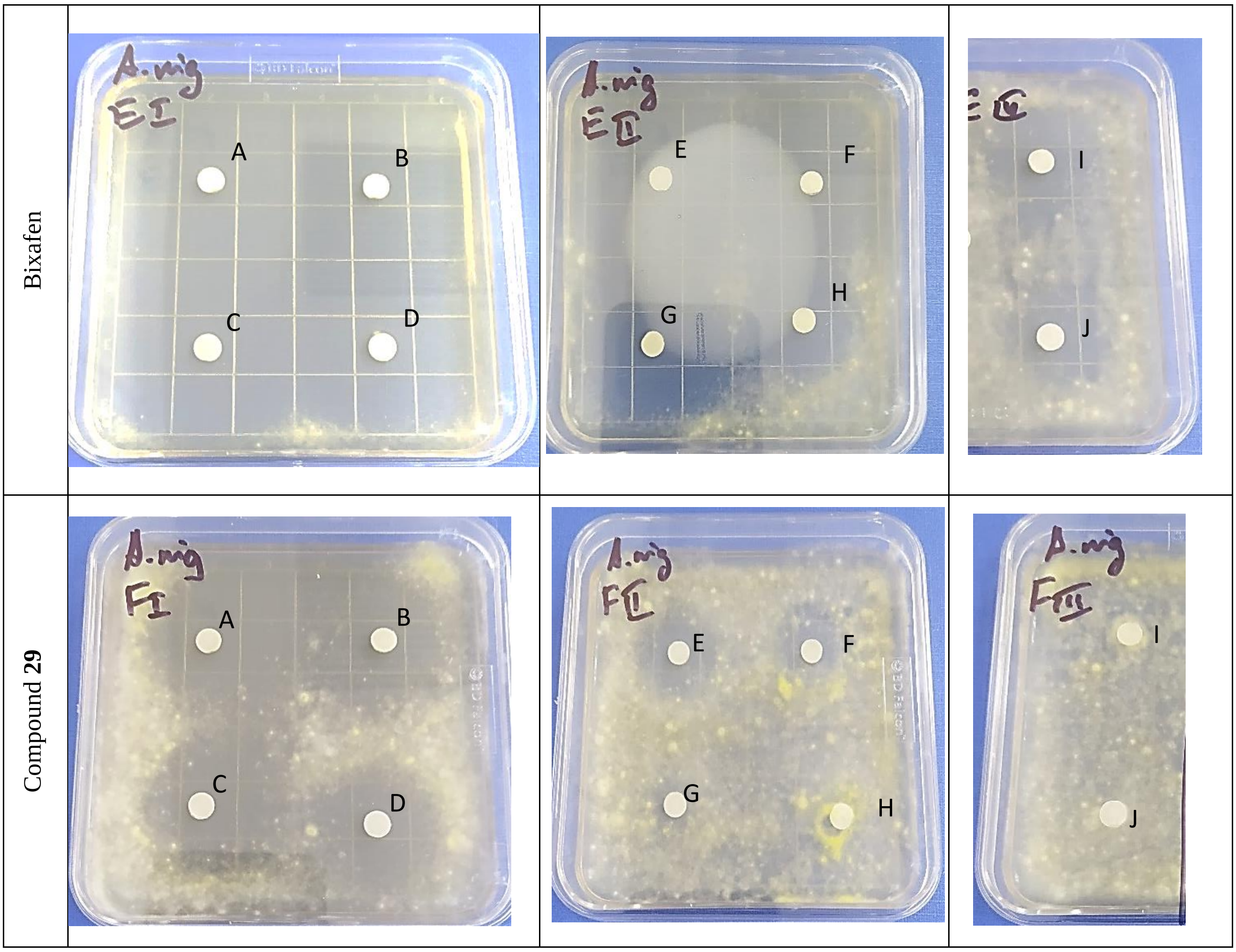

Figure S24. Activity of Bixafen and its analogue **(±)-29** toward *Aspergillus niger*, plates in 72 h. Concentration of compounds: A – 2 mg/mL; B – 1 mg/mL; C – 0.5 mg/mL; D – 0.25 mg/mL; E – 0.125 mg/mL; F – 0.06 mg/mL; G – 0.03 mg/mL; H – 0.016; I – 0.008 mg/mL; J – 0.004 mg/mL.

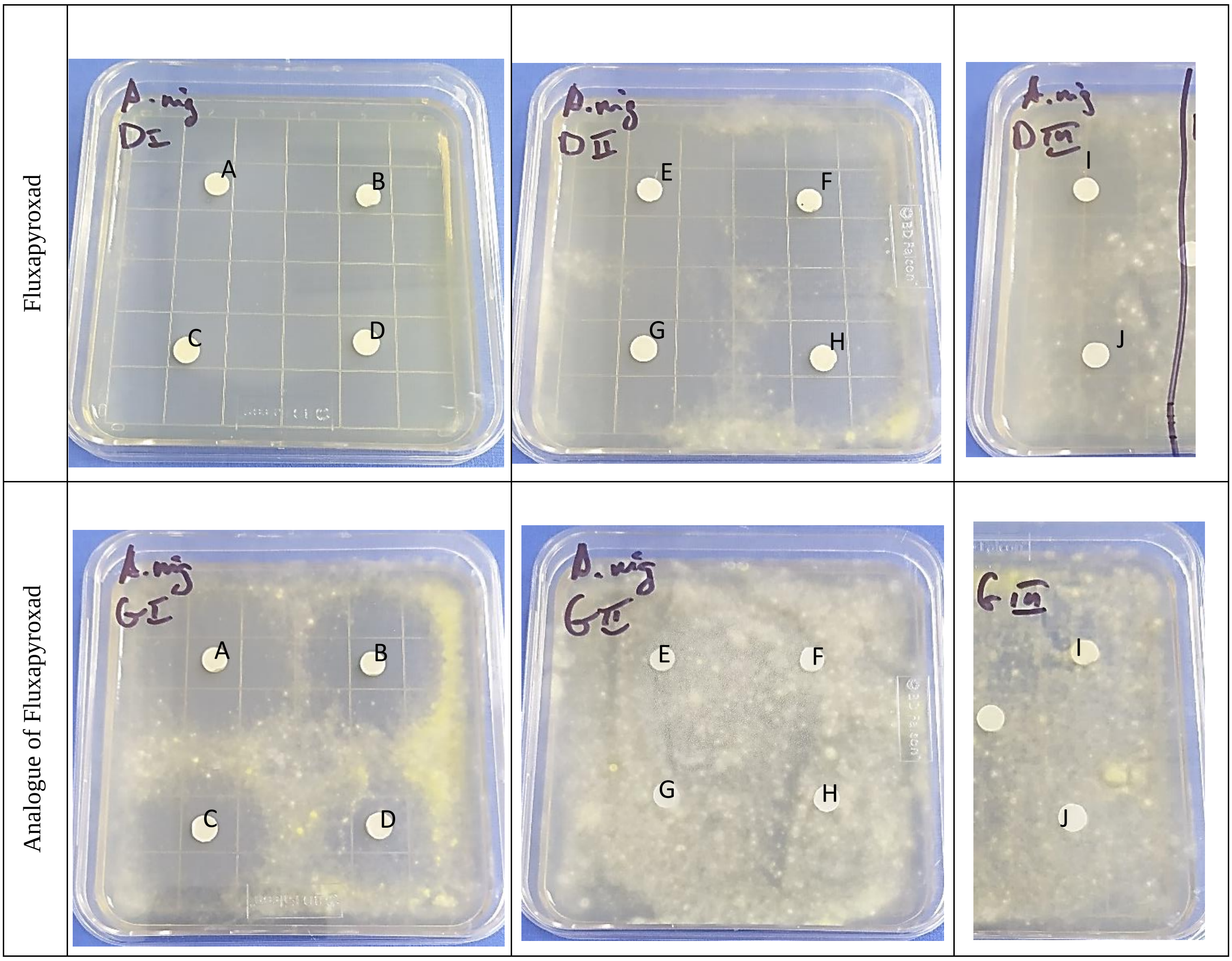

Figure S25. Activity of Fluxapyroxad and its analogue **(±)-30** toward *Aspergillus niger*, plates in 72 h. Concentration of compounds: A – 2 mg/mL; B – 1 mg/mL; C – 0.5 mg/mL; D – 0.25 mg/mL; E – 0.125 mg/mL; F – 0.06 mg/mL; G – 0.03 mg/mL; H – 0.016; I – 0.008 mg/mL; J – 0.004 mg/mL.
